# *MtNF-YA1*, A Central Transcriptional Regulator of Symbiotic Nodule Development, Is Also a Determinant of *Medicago truncatula* Susceptibility toward a Root Pathogen

**DOI:** 10.3389/fpls.2016.01837

**Published:** 2016-12-05

**Authors:** Thomas Rey, Philippe Laporte, Maxime Bonhomme, Marie-Françoise Jardinaud, Stéphanie Huguet, Sandrine Balzergue, Bernard Dumas, Andreas Niebel, Christophe Jacquet

**Affiliations:** ^1^Laboratoire de Recherche en Sciences Végétales, Université de Toulouse, CNRS, UPSCastanet Tolosan, France; ^2^Institut National de la Recherche Agronomique, Laboratoire des Interactions Plantes-Microorganismes, UMR441Castanet-Tolosan, France; ^3^Centre National de la Recherche Scientifique, Laboratoire des Interactions Plantes-Microorganismes, UMR2594Castanet-Tolosan, France; ^4^POPS Transcriptomic Platform – Institute of Plant Sciences Paris-Saclay IPS2, Centre National de la Recherche Scientifique, Institut National de la Recherche Agronomique, Université Paris-Sud, Université d’Évry Val-d’Essonne, Université Paris Diderot, Sorbonne Paris-Cite, Universite Paris-SaclayOrsay, France

**Keywords:** Medicago truncatula, Aphanomyces euteiches, symbiosis and immunity, Plant Roots, NF-Y transcription factor

## Abstract

Plant NF-Y transcription factors control a wide array of biological functions enabling appropriate reproductive and developmental processes as well as adaptation to various abiotic and biotic environments. In *Medicago truncatula*, *MtNF-YA1* was previously identified as a key determinant for nodule development and establishment of rhizobial symbiosis. Here, we highlight a new role for this protein in compatibility to *Aphanomyces euteiches*, a root pathogenic oomycete. The *Mtnf-ya1-1* mutant plants showed better survival rate, reduced symptoms, and increased development of their root apparatus as compared to their wild-type (WT) background A17. *MtNF-YA-1* was specifically up-regulated by *A. euteiches* in F83005.5, a highly susceptible natural accession of *M. truncatula* while transcript level remained stable in A17, which is partially resistant. The role of *MtNF-YA1* in F83005.5 susceptibility was further documented by reducing *MtNF-YA1* expression either by overexpression of the miR169q, a microRNA targeting *MtNF-YA1*, or by RNAi approaches leading to a strong enhancement in the resistance of this susceptible line. Comparative analysis of the transcriptome of WT and *Mtnf-ya1-1* led to the identification of 1509 differentially expressed genes. Among those, almost 36 defense-related genes were constitutively expressed in *Mtnf-ya1-1*, while 20 genes linked to hormonal pathways were repressed. In summary, we revealed an unexpected dual role for this symbiotic transcription factor as a key player in the compatibility mechanisms to a pathogen.

## Introduction

The NF-Y transcriptional regulator complex binds CCAAT boxes and is present in all eukaryotes (Mantovani, 1999; Matuoka and Chen, 2002). *NF-Y* complexes are heterotrimeric transcription factors composed of *NF-YA*, *NF-YB*, and *NF-YC* subunits. Mechanistic analysis of their transcriptional regulatory properties has been extensively investigated in animals (Dolfini et al., 2012). Briefly, *NF-YA* proteins located in the nucleus form a heterotrimeric complex with *NF-YB* and *NF-YC* subunits that interact in the cytoplasm before moving into the nucleus (Hackenberg et al., 2012). Among *NF-Y* proteins, the *A* subunit is thought to mediate the specificity of targets on genomic DNA by binding CCAAT motifs while *B* and *C* are thought to be involved in local chromatin decompaction (Calvenzani et al., 2012).

Unlike animals, plants possess multiple copies of NF-Y genes (Laloum et al., 2013). As an example *Medicago truncatula*, a relative of alfafa, possesses 8 *NF-YA*, 14 *NF-YB*, and 7 *NF-YC* subunits (Baudin et al., 2015). The resulting wealth of potential NF-Y subunit combinations opens the possibility for an extended spectrum of biological functions. In mammals, the nuclear factor Y complex is required to activate developmentally regulated genes, and is described as a key regulator of cell cycle progression (Bhattacharya et al., 2003; Benatti et al., 2011; Bungartz et al., 2012; Petroni et al., 2012). In plants, NF-YA, NF-YB, and NF-YC families of transcription factors have diversified and specialized to control plant–specific pathways including embryogenesis, germination, drought resistance, flowering, root development or nitrogen nutrition (Lotan et al., 1998; Wenkel et al., 2006; Laloum et al., 2013; Fornari et al., 2013).

Furthermore, the role of *NF-Y* genes in plant–microbe interactions is starting to be uncovered, especially in the frame of symbiotic interactions. In common bean (*Phaseolus vulgaris)*, *PvNF-YC1* subunit is up-regulated by efficient bacterial nitrogen fixing symbionts and promotes nodule development (Zanetti et al., 2010). In addition, MtNF-YC2 the ortholog of *PvNF-YC1* in *M. truncatula* was recently shown to form a functional trimer with MtNF-YA1 and MtNF-YB16 and to control nodule development (Baudin et al., 2015). The *LjNF-YA1* and *LjNF-YB1* subunits of *Lotus japonicus* are required for transcription of key genes acting in the nitrogen fixing nodule formation (Soyano et al., 2013). Another *NF-YC* subunit is specifically transcribed in *M. truncatula* cells forming arbuscules with mycorrhizal fungus (Hogekamp et al., 2011; Hogekamp and Küster, 2013; Gaude et al., 2012). Also, the knockdown of *NF-YA1a/b* in soybean (*Glycine max*), reduced the extent of mycorrhization (Schaarschmidt et al., 2013). However the best characterized *NF-Y* gene in plant–microbe interactions is probably *MtNF-YA1*, formerly named Mt*HAP2-1* (El Yahyaoui et al., 2004; Combier et al., 2006) which was shown to play a central role in the symbiosis between *M. truncatula* and *Sinorhizobium meliloti*. Its first identification in 2004 as an early and strong nodulin by a transcriptomic approach, suggested it is a highly specific regulator of nodulation (El Yahyaoui et al., 2004). Previous functional studies showed that *MtNF-YA1* controls late steps of nodule organogenesis under sequential control of two post transcriptional regulators (Combier et al., 2006, 2008). However, analyses of the *Mtnf-ya1-1* mutant also revealed the presence of abnormal infection threads (Laporte et al., 2014) suggesting that *MtNF-YA1* is implied in the early stages of symbiosis formation. Heterotrimeric complexes formed by *MtNF-YA1* as well as complementary roles of *MtNF-YA2* in nodulation and nodulin expression were subsequently documented (Baudin et al., 2015). In addition, using a fate map approach it was recently shown that *MtNF-YA1* is a key regulator of nodule meristem establishment and functioning (Xiao et al., 2014)

While the *NF-Y* complex has been involved in the regulation of plant development and symbiotic plant–microbe interactions, no study has addressed a potential function for *NF-Y* genes in plant–pathogen interactions. In this work, we used the *M. truncatula* – *Aphanomyces euteiches* pathosystem to assess a putative involvement of *MtNF-YA1* in plant responses to this pathogen. *A. euteiches* is a major pathogen of crop and forage legumes and is the causal agent of pea root rot disease (Gaulin et al., 2007). *M. truncatula* is a natural host for this biotrophic oomycete and accessions of this model legume have been shown to display a high level of variability in their colonization level by *A. euteiches* (Moussart et al., 2007; Bonhomme et al., 2014). Among them, F83005.5 is a natural accession displaying a high level of susceptibility. On the other end of the spectrum, A17 is a partially resistant line which was selected as the *M. truncatula* reference line for the genome sequencing project (Young et al., 2011) and mutant collections (Domonkos et al., 2013). While *A. euteiches* accomplishes a full life cycle in the root cortex of both lines, penetration in the vascular tissues of these plants differs. F83005.5 gets fully colonized whilst this phenomenon is hindered in A17 by immune mechanisms such as soluble phenolics production or lignification (Badis et al., 2015) and the development of supplementary pericycle cell layers and healthy lateral roots (LRs) (Djébali et al., 2009). In contrast to F83005.5, A17 plants usually survive to infection by *A. euteiches.*

Plant root colonization by beneficial and detrimental microbes shows an important degree of overlap (Rey and Schornack, 2013). Early steps in both types of interactions can involve signals and receptors that share structural homologies (Antolín-Llovera et al., 2012) and interfere with each other (Liang et al., 2013). In the last years, a dual role of several *M. truncatula* symbiotic genes in interaction with pathogens have been uncovered (Rey et al., 2014). Transmembrane receptor kinase such as the LysM-RLK *NFP* and the Histidine Kinase receptor *CRE1* that are involved in nitrogen fixing nodule formation participate to *M. truncatula* partial resistance to the oomycete *A. euteiches* (Rey et al., 2013; Laffont et al., 2015). *CRE1* along with the *EFD* transcription factor, both required for nitrogen fixing symbiosis, have been implied in the resistance to the bacterial pathogen *Ralstonia solanacearum* (Moreau et al., 2013). Finally, the glycerol phosphate acyl transferase *RAM2* produces cutin monomers promoting arbuscular mycorrhizal symbiosis as well as root invasion by the oomycetes *Phytophthora palmivora* and *A. euteiches* (Wang et al., 2012; Gobbato et al., 2013; Rey et al., 2014).

Because mutants in the Nod Factor perception and signaling pathway were also shown to affect susceptibility to *A. euteiches*, we investigated the role of another key symbiotic regulator: *MtNF-YA1*. For this purpose, we analyzed the *M. truncatula Mtnf-ya1-1* mutant belonging to the A17 genetic background upon *A. euteiches* inoculation. We show enhanced resistance of mutant plants suggesting that *MtNF-YA1* is a compatibility factor for the oomycete. This hypothesis was confirmed by loss of function analyses performed in the susceptible plant F83005.5. The expression pattern of *NF-YA1*, and microarray experiments pinpointed several unexpected functions for this transcription factor in the regulation of this plant pathogenic interaction.

## Materials and Methods

### Plant Material and Growth Conditions

A17 and F83005.5 *M. truncatula* accessions were used in each *A. euteiches* inoculation experiment as resistant and susceptible lines, respectively (Djébali et al., 2009). The *nf-ya-1-1* mutant was obtained from A17 EMS mutagenized seeds. A glutamine to stop codon substitution in position 137 out of 322 amino acids leads to non-sense mutation coding for a truncated non-functional protein depleted of DNA and protein-protein interactions domains (Laporte et al., 2014). Plants were grown *in vitro* with a 16-h light at 22°C and 8-h dark, 20°C, as previously described (Djébali et al., 2009). For root transformation, we used the ARqua1 strain of *Agrobacterium rhizogenes* as described by Boisson-Dernier et al. (2001, 2005) to produce composite plants. Composite plants were grown in the same condition than seedlings, as described in Djébali et al. (2009). Root organ cultures were produced using the same transformation pipeline and then propagated in absence of aerial parts on media supplemented with sucrose as described in Genre et al. (2013).

### Inoculation Procedures and Symptom Analysis

Zoospores of *A. euteiches*, a pea isolate, were produced as described by Badreddine et al. (2008) and inoculated according to methods described in Rey et al. (2013). Four independent *in vitro* infection assays were performed with 15 to 30 inoculated plants per line in each repeat. The calculated means for each parameter were compared through statistical ANOVA analyses.

### Sample Preparation for Microscopy and Image Analysis

For optical microscopy, 100 μm root sections were prepared from fresh infected seedlings and labeled with WGA-FITC, as described by Djébali et al. (2009) and Rey et al. (2013) to localize *A. euteiches* hyphae using epifluorescence illumination (excitation filter, BP 450–490 nm). Images were acquired using a CCD camera (color Coolview, Photonic Science, Robertsbridge, UK). Image J^1^ was used to perform hyphal counts of *A. euteiches* in root cross sections. Following image acquisition, the green channel of the pictures (which shows WGA-FITC signal) was transformed in a binary image where hyphae appear in black and then subjected to a particle analysis with standard settings.

### Constructions and Vectors

The miR169q overexpression was conducted with constructs designed by Combier et al. (2006). Briefly, a fragment of the pre-miR169q located just outside of the stem-loop structure was amplified and then cloned into pPex (Combier et al., 2006). Entry clones for the *GUS* open reading frame (Control) and the 3′UTR of *MtNF-YA1* were obtained in the Gateway vectors pDONR207 and pDONRP2R-P3, respectively, and recombined in pPEX-GUS. For p*MtNF-YA1* promoter analysis in *M. truncatula*, entry clones were recombined in the binary vector pK7m34GW (Laporte et al., 2014).

### RNA Extraction and Quantitative RT-PCR

Total RNAs were isolated from inoculated and non-inoculated roots with the “RNeasy for plant and fungi” kit (Qiagen). Following total RNA extraction and DNAse treatment, RNA integrity was checked using a Bioanalyzer (Agilent technologies) and reverse transcriptions were performed with 1 μg of total RNA for each sample using the High-Capacity cDNA Reverse Transcription Kit (Applied Biosystems) with random primers in a total volume of 20 μL. Gene specific primers were designed using the Quantprime software^2^ (Arvidsson et al., 2008). Then, the cDNAs were used for high-throughput qPCR using the BioMark^TM^ HD System (Fluidigm): first, 1.3 μL of a 1:40 dilution of the synthesized cDNA were submitted to specific target amplification (STA) by PCR amplification in a 5 μL reaction containing 96 *M. truncatula* specific primer pairs (50 nM each) and a twofold dilution of the TaqMan^®^ PreAmp Master Mix (Applied Biosystems). The PCR program consisted of 14 cycles of 15 s at 95°C followed by 4 min at 60°C. Then, 340 nL of preamplified cDNA were used for qPCR array analysis in a 6.7 μL reaction using EvaGreen chemistry (Applied Biosystems). Data were analyzed with the BioMark Real-Time PCR Analysis Software Version 2.0 (Fluidigm) (Vergnes et al., 2014). Calculations for comparing expression data were performed using the 2^-ΔCt^ and the 2^-ΔΔCt^ methods (Livak and Schmittgen, 2001) using means Ct values of three *M. truncatula* reference genes which were selected using the NormFinder software (Andersen et al., 2004): Histone-3-like (Medtr4g097170), Ubiquitin family protein (Medtr3g097170) and Translation elongation factor 1 (Medtr6g021800) were used to standardize expression in seedlings. Medtr4g097170 and Medtr6g021800 were used to normalize expression in composite plants whilst only Histone-3-like was analyzed in root organ culture experiments due to variable expression profile of the other housekeeping genes in these two types of biological material (Supplementary Figure 1). Gene identifier, primers, amplicons length and melting temperature are listed in (Supplementary Table 1). Gene encoding an *A. euteiches* tubuline transcript was used to assess the amount of pathogen biomass *in planta* at 6 dpi. For detection of miR169q overexpression, primers designed just outside the stem-loop of the pre-miR were used to detect transcription in plant cDNA.

**FIGURE 1:**
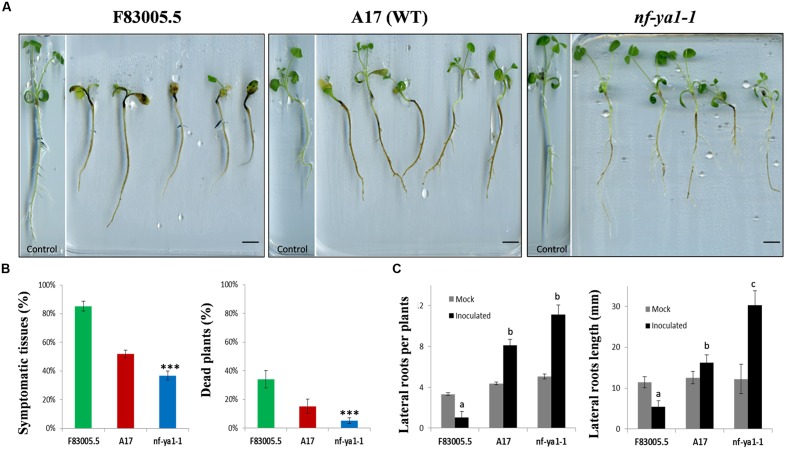
**Visual observations on *Mtnf-ya1-1* mutant, A17 partially tolerant wild-type (WT) and F83005.5 susceptible genotype along invasion by *Aphanomyces euteiches*.** **(A)** Visuals symptoms on F83005.5, A17 and nf-ya-1 mutants 14 dpi, bar = 1cm. **(B)** Symptoms extent (%) 14 dpi and dead plants 21 dpi (%) (*n* = 150 plants for each genotype). In all assays, error bars represent standard errors. Mean values for each line were compared by the *t*-test for symptoms extent; and by the χ^2^ test for proportion of dead plants. In all graphs asterisks indicate significant differences compared with WT results (^∗∗∗^*P* < 0.001). **(C)** Total root numbers and length (in millimeters) 21 days after germination in control conditions (gray bars) and 21 days post inoculation (dpi) by *Aphanomyces euteiches* (black bars) (*n* = 150 plants for each genotype). A pairwise Wilcoxon test was applied for number and length of lateral roots between A17 and the other lines results.

### Affymetrix Array Hybridization and Data Analyses

Total RNA was extracted using the RNeasy Kit (Qiagen). One microgram of total RNA was used to produce labeled cRNA and hybridize Affymetrix GeneChip^®^ *M. truncatula* genome arrays at INRA-URGV (Evry, France). Three independent biological replicates were performed for A17 wild-type (WT) and *Mtnf-ya1-1* mutants, non-inoculated or harvested 1 and 6 days post inoculation (dpi). For each biological repetition RNA samples were extracted from ten roots of 15-day-old plants. Following extraction, RNA samples were checked for their integrity on the Agilent 2100 bioanalyzer according to the Agilent Technologies (Waldbroon, Germany). One microgram of total RNA was used to synthesize biotin-labeled cRNAs with the One-cycle cDNA synthesis kit (Affymetrix, Santa Clara, CA, USA). Superscript II reverse transcriptase and T7-oligo (dT) primers were used to synthesize the single strand of cDNA at 42°C during 1 h followed by the synthesis of the double stranded cDNA by using DNA ligase, DNA polymerase I and RNaseH during 2 h at 16°C. Clean-up of the double-stranded cDNA was performed with Sample Cleanup Module (Affymetrix) followed by *in vitro* transcription (IVT) in presence of biotin-labeled UTP using GeneChip^®^ IVT labeling Kit (Affymetrix, Santa Clara, CA, USA). Quantity of the labeled-cRNA with RiboGreen^®^ RNA Quantification Reagent (Turner Biosystems, Sunnyvale, CA, USA) was determined after cleanup by the Sample Cleanup Module (Affymetrix). Fragmentation of 15 μg of labeled-cRNA was carried out for 35 min at 94°C, followed by hybridization during 16 h at 45°C to Affymetrix GeneChip^®^ *M. truncatula* Genome Array representing approximately 61,200 probes: 32,167 *M. truncatula* EST/mRNA-based probe sets; 18,733 *M. truncatula* IMGAG and phase 2/3 BAC prediction-based probes; 1,896 *M. sativa* EST/mRNA based probes; and 8,305 *S. meliloti* gene prediction-based probes. After hybridization, the arrays were washed with two different buffers (stringent: 6X SSPE, 0.01% Tween-20 and non-stringent: 100 mM MES, 0.1M [Na+], 0.01% Tween-20) and stained with a complex solution including Streptavidin R-Phycoerythrin conjugate (Invitrogen/molecular probes, Carlsbad, CA, USA) and anti-Streptavidin biotinylated antibody (Vectors laboratories, Burlingame, CA, USA). The washing and staining steps were performed in a GeneChip^®^ Fluidics Station 450 (Affymetrix). The Affymetrix GeneChip^®^ *M. truncatula* Genome Arrays were finally scanned with the GeneChip^®^ Scanner 3000 7G piloted by the Command Console Launcher Tool. The raw CEL files were imported in R software for data analysis^3^. The data were normalized with the gcrma algorithm, available in the Bioconductor package (Gentleman et al., 2004). Raw and normalized data are available through the CATdb database (AFFY_aphanomyces_*M. truncatula*) (Gagnot et al., 2008) and from the Gene Expression Omnibus (GEO) repository at the National Centre for Biotechnology Information (NCBI) (Turnpenny, 2008), A17 control and 1 day post inoculation data are available in GSE20587 and A17 6 dpi as well as *nf-ya1-1* control, 1 and 6 dpi are available in GSE26046.

### Statistical Analyses of Transcriptomic Data

Genes regulated depending on genotype and time post inoculation were determined by ANOVA according to Moreau et al. (2013). To determine differential gene expression subsequent to ANOVA, we performed a usual two group *t*-test that assumes equal variance between groups. The variance of the gene expression per group is a homoscedastic variance, where genes displaying extreme variance (*p* < 0.001) were excluded. The raw *p*-values were adjusted by the Bonferroni method, which controls the Family Wise Error Rate (FWER). A gene was declared differentially expressed if its Bonferroni *p*-value was lower than 0.001. Multi experiment Viewer (MeV^4^) was used for hierarchical clustering of gene expression profiles using euclidean distance and average linkage parameters (Eisen et al., 1998). Annotations used in this study are based on the work of published by Czaja et al. (2012) and Roux et al. (2014). Genes were also classified according to MapMan mapping Mt3.0_AFFY_0510^5^. Venn diagram was performed on Mapman tool.

## Results

### A Null-Mutation *in MtNF-YA-1* Increases Resistance to *A. euteiches* and Modifies Root Architecture upon Pathogen Infection

We used the *Mtnf-ya1-1* null mutant line obtained in the WT A17 genetic background (Laporte et al., 2014), to assess the role of *MtNF-YA-1* in the colonization by *A. euteiches* zoospores. Young seedlings of this *Mtnf-ya1-1* mutant line were inoculated by *A. euteiches* zoospores, along with A17 and F83005.5 seedlings which served as tolerant and susceptible lines, respectively. Phenotypes of both lines upon inoculation are shown in **Figure 1A**. At 14 dpi, most F83005.5 plants were severely attacked and their development impaired when compared to uninfected seedlings. A17 plants had similar size as control but a browning reflecting colonization of the whole roots cortical tissues was observed. Inoculated *Mtnf-ya1-1* plants displayed symptoms too, but parts of their primary roots as well as some newly formed secondary roots remained symptomless. The extent of rot symptoms and percentages of dead plants (**Figure 1B**) were measured for 150 seedlings of each genotype and both parameters support a significant decrease of symptomatic tissues and of death rate for *Mtnf-ya1-1* compared to A17 (*p*-value <0.001). We also assessed putative changes in root architecture of each line in control conditions and upon invasion by *A. euteiches*. The number and length of secondary roots was similar between the three lines in control conditions (*n* = 30 plants). In contrast, drastic modification of the root architecture was observed for all lines; 3 weeks post inoculation (**Figure 1C**). In F83005.5, a strong decrease in length and number of roots after inoculation was observed whereas A17 showed a two fold increase in the LR numbers with an average of their total length similar to control plants. In *Mtnf-ya1-1*, number of LRs (+29% – *p*-value <0.05) and their length (+37% – *p*-value <0.001) were both significantly increased compared to A17 WT. Taken together, these results suggest that (i) root branching and development are positively correlated to resistance to *A. euteiches* and (ii) *MtNF-YA-1* is a negative regulator of these responses in A17.

### Cytological Analyses Indicate a Lower Colonization of *A. euteiches* within *Mtnf-ya1-1* Root Tissues

**FIGURE 2:**
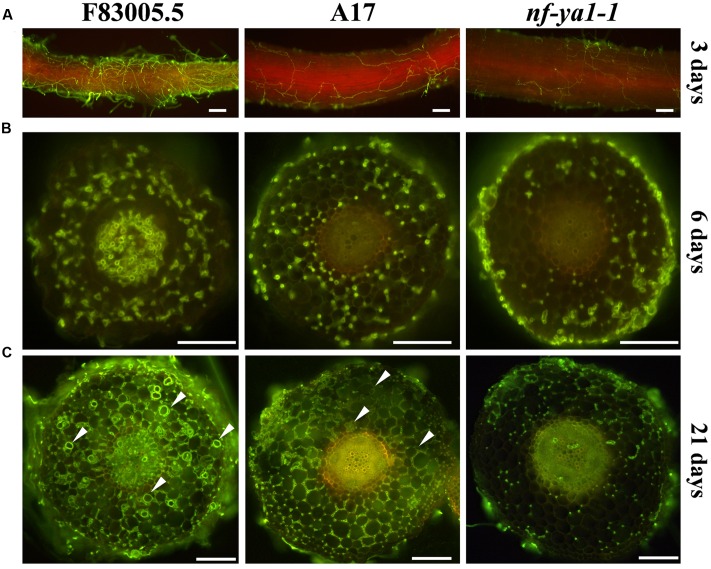
**Comparisons of spatiotemporal colonization pattern by *A. euteiches* in the F83005.5 susceptible line, A17 WT and *nf-ya-1-1.*** For cytological observations of *A. euteiches* was labeled in green with WGA-FITC (Bar = 100 μm). Infected seedlings were imaged with a GFP long pass filter enabling the oomycete detection in green and the root autofluorescence in red. Root cross sections are 100 μm thick. Oospores are indicated by white arrows. **(A)** Root surface observation 3 dpi, F83005.5 rhizodermis is fully colonized while a limited amount of hyphae is observed at the surface of A17 and its *nf-ya1-1* mutant. A bright red autofluorescence is observed in the last two lines but not in F83005.5. **(B)** Root sections 6 dpi. *A. euteiches* hyphae spread intercellularly within the cortex of all three lines. The oomycete colonizes the vascular tissues of F83005.5 as early as 6 days following inoculation whilst these tissues remained free of hyphae in A17 and *nf-ya1-1* 3 weeks after the inoculation. **(C)** Root sections 21 dpi. *A. euteiches* hyphae spread intercellularly within the cortex of all three lines and in the stele of F83005.5. Numerous oospores are being observed in F83005.5 (arrowheads mark some of them) after 21 days, showing the oomycete fulfilled it sexual lifecycle in the susceptible host.

As *Mtnf-ya1-1* plants showed reduced visual symptoms when challenged with *A. euteiches*, we decided to thoroughly observe the colonization process of the oomycete. To dissect the effect of *MtNF-YA1* knock out on the colonization behavior of the pathogen, its mycelium was stained using Wheat Germ Agglutinin-FITC (Badreddine et al., 2008). By this mean, we monitored the dynamics of the colonization from initial penetration events to complete invasion using epifluorescence microscopy. At 3 dpi, the rhizodermis of F83005.5 was completely surrounded by hyphae, while colonization was sparser in A17 and *Mtnf-ya1-1* (**Figure 2A**). Following initial superficial colonization, the mycelium crosses the epidermis and enters cortical tissues through wounds and intercellular spaces. Root cross sections were performed 6 days after inoculation to track pathogen progress. An extensive colonization of F83005.5 root tissues was recorded, notably within the inner cortex and vascular tissues (**Figure 2B**). Hyphae were spreading extracellularly from the root tip inoculation point, by following intercellular spaces. In A17, a similar behavior was observed with the exception that the central cylinder was not invaded. In comparison, the *Mtnf-ya1-1* roots harbored a reduced amount of hyphae mainly restricted to the outer cortex compared to A17 (**Figure 2B**). Twenty-one days post inoculation, root tissues were heavily colonized in F83005.5 and numerous oospores were produced inside dead cells, indicating that the pathogen fulfilled its life cycle (**Figure 2C**). In A17, numerous intercellular hyphae invaded the entire cortex but never entered the vascular tissues and only a few oospores were detected, in line with previous studies (Supplementary Figure 3A) (Djébali et al., 2009, 2011). The *Mtnf-ya1-1* mutant displayed consistently a reduced hyphal colonization mostly located in the outer root cortex whilst reduced colonization of the inner cortex was observed (Supplementary Figure 3B). *Aphanomyces* hyphae were excluded from the vascular system and almost no oospores were detected (**Figure 2B**; Supplementary Figures 3A,B). Taken together, these observations indicate that intra-radical colonization by *A. euteiches* along with its biological cycle were hampered in *Mtnf-ya1-1*.

### Description of Natural Allelic Variation and Transcriptional Regulation of *MtNF-YA1* in *M. truncatula* Roots Colonized by *A. euteiches*

**FIGURE 3:**
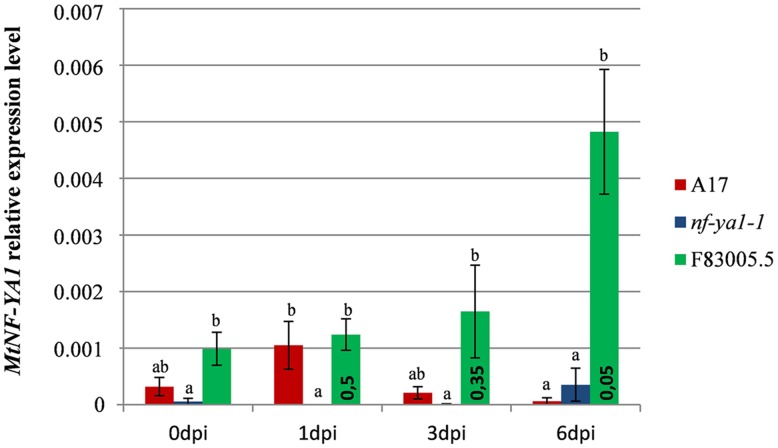
**Expression pattern of *MtNF-YA1* in A17, *nf-ya1-1*, and F83005.5 in control and *A. euteiches* inoculated conditions.** Root tissues from five seedlings were pooled in each individual sample and expression level at each datapoint is represented by a mean of three independent samples. The level of *MtNF-YA1* expression was analyzed by RT-qPCR and the 2^-Δ^*^C^*^T^ method by using three reference genes as described in “Materials and Methods.” Error bars display standard errors and significance of differential expression level was determined using a Mann–Whitney *U* test. Groups of expression levels were determined between genotypes at each individual time points, statistically similar expression level belongs to groups a, b or ab and bars which do not share any of these letters are significantly different at *p*-value threshold 0.05. In addition, a Mann–Whitney *U* test was performed between 0 dpi and 1, 3, and 6 dpi expression levels in F83005.5 to assess significance of the variations observed across the kinetic, *p*-values are indicated inside green bars.

To strengthen the role of *MtNF-YA1* during the accommodation of *A. euteiches*, its expression was analyzed by RT-qPCR in F83005.5, A17 and *Mtnf-ya1-1* at 0, 1, 3, and 6 dpi (**Figure 3**). We investigated the gene expression in the three lines grown in mock conditions and noticed a slightly stronger expression in F83005.5 compared to A17 albeit the difference was not significant. We then analyzed *MtNF-YA1* expression patterns at 1, 3, and 6 days following inoculation. We observed a late induction of *MtNF-YA1* expression in F83005.5 which peaked 6 days after inoculation at a fourfold level compared to non-inoculated samples. We assessed significance of this variation within F83005.5 and detected a *p*-value <0.05 between 6 dpi expression values and all the other time points of the kinetic in this genotype. In A17, *MtNF-YA1* expression was low and stable throughout the kinetic. We compared gene expression between genotypes at 6 dpi and observed that A17 expression significantly differed from F83005.5 and was reduced to 13% of the expression level recorded in this susceptible genotype. The *MtNF-YA1* expression observed in the *nf-ya1-1* mutant was significantly lower at 1 day post inoculation than in A17 but otherwise similar to the barely detectable levels of transcripts detected in A17. The *Mtnf-ya1-1* mutant produces an early stop codon transcript and Western blot analysis showed previously that the truncated protein was absent (Laporte et al., 2014). The very low level of *MtNF-YA1* transcripts measured at 1 dpi as compared to A17 (**Figure 3**) further supports the hypothesis that this might be a consequence of non-sense mediated mRNA decay (NMD) (Kervestin and Jacobson, 2012) and that *nf-ya1-1* does not express functional *MtNF-YA1* transcripts.

We then checked if allelic variation in the promoter sequences of *MtNF-YA1* between susceptible and resistant lines may explain the observed differential expression. We observed polymorphism both in the coding and promoter region of *MtNF-YA1* by investigating SNP data from the *M. truncatula* HapMap Project^6^, on a set of sequences from 226 *M. truncatula accessions* (see **Supplementary Data Sheet S1**; Supplementary Figure 2). More precisely, the genetic distance (Kimura 2-parameters) between A17 and F83005.5 for both regions, and even for the promoter region only (∼2 kb), was among the top 3% highest pairwise distance values calculated, based on a total of 25651 pairwise comparisons (Supplementary Figure 3; Supplementary Table 5). This finding thus supports the hypothesis that the differential expression of *MtNF-YA1* between A17 and F83005.5 could be due to increased divergence in the promoter region.

### Suppression of *MtNF-YA-1* Expression Increases Resistance of the Susceptible F83005.5 Line

**FIGURE 4:**
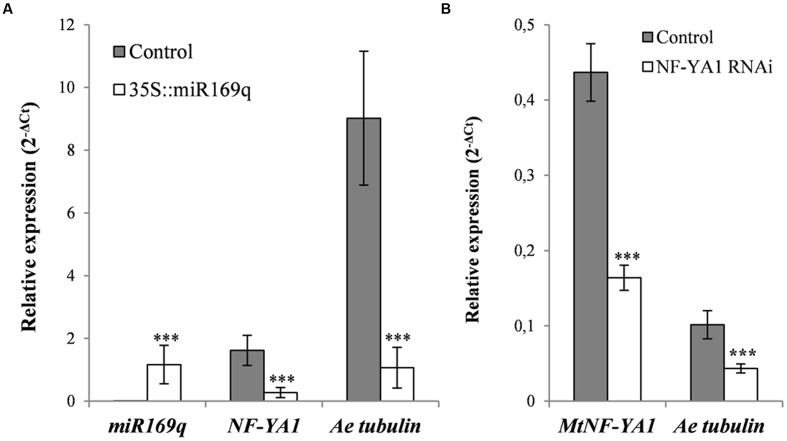
**Silencing approaches of *MtNF-YA1* in F83005.5 susceptible line.** The pre-*miR169q, MtNF-YA1* and *A. euteiches* tubulin transcripts were quantified by RT-qPCR and normalized with 2^-Δ^*^C^*^T^ methods. **(A)** Overexpression of pre-miR169q reduces *MtNF-YA1* transcripts accumulation 6 dpi and decrease *A. euteiches* development. Experiments were performed on three independent root organ culturelines for both control vector and pre-miR169q overexpressor. Expression values were standardized with the plant housekeeping gene Medtr4g097170. **(B)** Silencing of *MtNF-YA1* by the overexpressing construct of antisense *MtNF-YA1 3′UTR* decreases *A. euteiches* development. Experiments were performed with 12 independent samples containing each 10 composites plants for both control and silencing vector. Expression were standardized with the mean of two reference genes Medtr4g097170 and Medtr6g021800. Wilcoxon rank sum test was applied for root organ culturesqRT-PCR results (^∗∗∗^*p*-value <0.001) and ANOVA was applied for composite plants qRT-PCR results (^∗∗∗^*p*-value <0.001).

To validate the involvement of *MtNF-YA1* in disease susceptibility, we decided to suppress its expression in the susceptible genotype F83005.5. First, the miR169q that negatively regulates the *MtNF-YA1* transcript levels (Combier et al., 2006) was overexpressed in F83005.5 root organ cultures by *Agrobacterium rhizogenes* mediated transformation. To do so, a *35S::miR169q* construct overexpressing the precursor of the miR (pre-mir169q) was transformed in *M. truncatula* roots. Their susceptibility level to *A. euteiches* was compared to root organ cultures of the same line transformed with an empty vector control by assessing the amount of pathogen through RT-qPCR. Three independent hairy root lines for each construct were selected on the basis of their growth speed for further work. Both control and miR169q overexpressing root organ cultures were inoculated with *A. euteiches* and harvested at 6 dpi for subsequent analysis. Using RT-qPCR, we checked the overexpression of pre-miR169q in transgenic lines and detected on average a 305-fold increase in pre-miR169q transcript levels as compared to root organ cultures transformed with control vector (*p*-value <0.001) (**Figure 4A**). Consequently, *MtNF-YA1* transcript levels were reduced to 17% of the WT situation (*p*-value <0.001) (**Figure 4A**). Using the same samples, we quantified *A. euteiches* tubulin transcripts 6 days after root inoculation by using a RT-qPCR method as a proxy (Rey et al., 2013). This time point was chosen as it allowed detection of both pathogen and plant cDNAs and matches the timing of *MtNF-YA1* transcriptional induction in F83005.5. Strikingly, a strong reduction of 88% in the *A. euteiches* tubulin transcripts was observed for *35S::miR169q* root organ cultures compared to control root organ cultures (**Figure 4A**).

In a complementary approach, composite F83005.5 plants were transformed with control vector and an RNAi construct targeting specifically the 3′UTR of *MtNF-YA1* (*35S::aSIII’UTR*). One hundred and twenty transformants were obtained for each construct and subsequently inoculated with *A. euteiches.* Six days after inoculation, transgenic root systems were pooled in 12 samples for each constructs. The RNAi strategy led to a significant decrease of the *MtNF-YA1* transcript accumulation (63% lower than in controls). Overall colonization level of roots isolated from composite plants was lower than in the root organ culture experiments but the *A. euteiches* tubulin transcripts were also reduced in *NF-YA1* silenced roots to 43% of their level in control roots (**Figure 4B**).

In conclusion, both miRNA and RNAi-based strategies led to a significant down-regulation of *MtNF-YA1* expression and this correlated with an increased resistance of the susceptible genotype F83005.5 to *A. euteiches.*

### *NF-YA1* Controls a Subset of *M. truncatula* Disease Resistance Responses to *A. euteiches*

**FIGURE 5:**
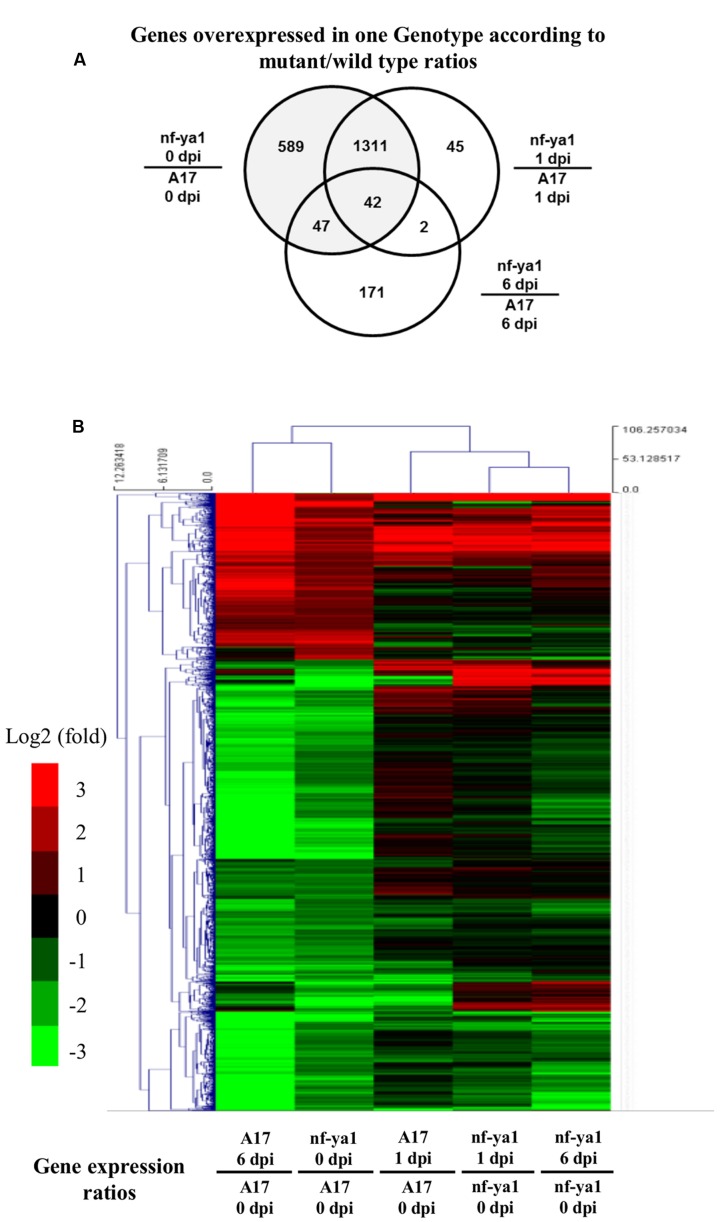
**Transcriptomic analysis of A17 and *nf-ya-1-1* in healthy plants and upon *A. euteiches* inoculation.** Analyses were performed on probes which were significantly regulated (Tuckey test, Bonferroni-corrected *p*-value <0.001). **(A)** Venn Diagram showing probes significantly regulated between genotypes at 0, 1 and 6 dpi. **(B)** Hierarchical clustering of genotype-dependent probes regulated upon *A. euteiches* inoculation. Induced probes (log2 ratio > 1) are in red, repressed probes (log2 ratio <-1) are in green. The detail of the gene annotation and their position in the cluster can be found in Supplementary Table 4.

To identify the gene network under control of the *MtNF-YA1* transcription factor in interaction with *A. euteiches*, we carried out transcriptome analyses using whole genome Affymetrix chips on A17 and *Mtnf-ya1-1. RNAs* extracted from whole root systems were harvested in control conditions and one or 6 dpi. An ANOVA analysis was performed to detect differential gene expression at each time point among the two genotypes (“Genotype effect“) and between controls and inoculated conditions (“Inoculation effect“). For each regulated gene (Bonferroni-corrected *p*-value <0.001), the factors conferring the changes in gene expression were determined and assigned to genotype or inoculation factors, for more details regarding this analysis pipeline, see Moreau et al. (2013). We then sorted genes displaying at least a twofold induction or repression compared to control (fold >1 or <-1 in log2). Five thousand three hundred and eighteen probes corresponding to 3970 individual genes were found to be regulated by one or both factors mentioned above, representing almost 10% of the overall Affymetrix chip probes (50900 probes on the chip) (Supplementary Table 4). We validated by RT-qPCR a selection of regulated genes of both genotype and inoculation effects and obtained an overall good correlation in the fold change obtained by the microarray and the qPCR (*R*^2^ = 0.78) (Supplementary Figure 1; Supplementary Table 1). In order to specifically assess the contribution of *MtNF-YA1* to these responses, we focused on the 1965 probes representing 1509 genes affected by Genotype effect as they display differential expression between A17 and the *Mtnf-ya1-1* mutant (Supplementary Tables 2 and 4). We produced a Venn diagram of probes overexpressed in *Mtnf-ya1-1* or A17 using ratios between mutant and WT expression levels at each time point (threshold = fold 2 between genotypes). A vast majority of probes were altered in the ratio between control 0 dpi mutant and wild type plants, (grayed circle) indicating major basal differences between the root transcriptome of *Mtnf-ya1-1* and A17 (**Figure 5A**). A hierarchical clustering of these 1965 probes revealed two patterns of gene regulation upon *A. euteiches* colonization either mostly induced (529 probes, 360 genes) or preferentially repressed (1436 probes, 1150 genes) (**Figure 5B**). Interestingly, expression of these 1965 probes in non-inoculated *Mtnf-ya1-1* was similar to the gene expression observed in A17 at 1 and 6 dpi. A similar observation can be drawn from genes constitutively repressed in the mutant (Supplementary Tables 3 and 4). This last finding implies that part of the A17 transcriptomic responses to *A. euteiches* is already constitutively expressed in *Mtnf-ya1-1* in non-challenged conditions. Hence, *NF-YA1* appears as a central regulator of responses triggered in WT roots by *A. euteiches* invasion.

We looked closer into the biological functions of genes deregulated in *Mtnf-ya1-1*. Among the 529 probes induced in non-inoculated mutant control and infected A17, 36 genes are unambiguously related to stress responses already known to contribute to resistance toward *A. euteiches* (Supplementary Tables 2 and 4) (Colditz et al., 2004, 2005, 2007; Djébali et al., 2009, 2011; Schenkluhn et al., 2010; Kiirika et al., 2012; Rey et al., 2013). The 1436 probes showing repression spanned more diverse processes (Supplementary Tables 3 and 4). Among these, 20 genes involved in hormonal biosynthesis and signaling, notably auxins, ethylene and abscisic acid (ABA) are down regulated in *Mtnf-ya1-1* already before inoculation and sometimes 1 and 6 dpi(Supplementary Table 3). These hormonal imbalances in the mutant may explain stress responses mentioned above or alternatively the modifications of root architecture observed upon invasion (De Smet, 2012; De Lucas and Brady, 2013; Tian et al., 2014).

## Discussion

The role of *MtNF-YA1*, a major regulator of nitrogen fixing nodule organogenesis was examined in the frame of the interaction between the model legume *M. truncatula* and the root pathogenic oomycete *A. euteiches*. So far, expression analysis using the *M. truncatula* gene atlas^7^ and qRT-PCR or p-GUS studies (Combier et al., 2006; Laloum et al., 2013; Laporte et al., 2014) had shown that *MtNF-YA1* was specifically expressed in root and nodule tissue challenged by symbiotic *S. meliloti* bacteria and not in any other organ or tissue. Here, we show that *MtNF-YA1* can also be up-regulated by *A. euteiches*, a root-pathogenic oomycete. This expression can only be detected in F83005.5, a *M. truncatula* accession that is susceptible to *A. euteiches*, while no induction could be detected in A17, a resistant accession (Djébali et al., 2009). In addition, phenotypic and cytological observations showed that *Mtnf-ya1-1* is more resistant to intra-radical invasion than the reference line A17 which is partially resistant. As in A17, *A. euteiches* mycelium is excluded from the root vascular tissues of the *Mtnf-ya1-1* mutant. Hyphal colonization is mostly located in the outer root cortex of the mutant, while important colonization of the inner cortex happens in A17. In addition, no oospores were detected in *Mtnf-ya1-1*, suggesting that *A. euteiches* cannot finalize its life cycle in this mutant. Strikingly, the very susceptible line F83005.5 can be turned into a more resistant plant by silencing *MtNF-YA1* by an RNAi strategy or miR169 overexpression. Taken together these data strongly suggest that in addition to being a central positive regulator of the symbiotic Rhizobium–legume interaction, MtNF-YA1 is also a positive determinant of susceptibility of *M. truncatula* toward a root pathogen. This unsuspected role aside from nitrogen fixing symbiosis sheds new light on the potential functions of *MtNF-YA1*.

We then performed a comparative transcriptome analysis in order to start to unravel by which mechanisms MtNF-YA1 might regulate susceptibility toward *A. euteiches*. Our transcriptomic analysis uncovered a gene network regulated by *MtNF-YA1*. More than 1500 genes were altered in their expression in *Mtnf-ya1-1* roots grown under control conditions. This finding was especially unexpected given the low expression level of *MtNF-YA1* in control conditions. The basal expression pattern of the transcription factor was recently documented in LR primordia (Laporte et al., 2014), suggesting that deregulated genes are potential targets of *MtNF-YA1* in these tissues where they may control both immune responses and development.

### *MtNF-YA1* Regulates Plant Immunity

Our transcriptomic approach revealed that 36 genes involved in resistance and stress mechanisms are constitutively expressed in the uninoculated *Mtnf-ya1-1* mutant, thereby indicating *MtNF-YA1* negatively regulates the expression of defense and stress-related genes in roots. The limitation of *A. euteiches* hyphal colonization in the root cortex observed after the knockout of *MtNF-YA1* in the *Mtnf-ya1-1* mutant as well as the knock down of this gene in the partially resistant A17 or the very sensitive F83005.5 could thus be explained by the constitutive expression of these defense and stress-related genes. Interestingly Laporte et al. (2014) also observed a negative effect of the knockout of *MtNF-YA1* in the *Mtnf-ya1-1* mutant on epidermal and cortical root colonization but by the symbiotic bacterium *Sinorhizobium meliloti*. This suggests that, despite the clear differences between root colonization by a pathogenic oomycete and by a beneficial bacterium some common mechanisms regulated by *MtNF-YA1* may exist. Rhizobial infection strongly up-regulates *MtNF-YA1* expression, especially in the tissues surrounding infection threads (Laporte et al., 2014). In the *Mtnf-ya1-1* mutant infection progression is severely disturbed, possibly because MtNF-YA1 does not downregulate defense mechanisms as in A17 WT. It is possible that *Aphanomyces euteiches* has evolved to exploit this potential symbiotic mechanism and thus downregulates defense mechanisms by up-regulating *MtNF-YA1* in susceptible ecotypes. How widespread this susceptibility mechanism is in the interaction with root filamentous pathogens or other root pathogens still has to be addressed.

### *MtNF-YA1* and Lateral Root Development

In *Arabidopsis thaliana*, overexpression of the closest homolog of MtNF-YA1, AtNF-YA2 leads to a significant increase in LR primordia density (Sorin et al., 2014) and the authors propose that AtNF-YA2 controls root architecture. Furthermore Laporte et al. (2014) reported that *MtNF-YA1* is also expressed transiently in LR primordia during LR initiation suggesting that this gene may also control LR growth in *M. truncatula*.

The resistance of *M. truncatula* roots to *A. euteiches* infection is correlated with the ability of the plant to generate new roots upon invasion by the pathogen (Djébali et al., 2009; Laporte et al., 2014; Laffont et al., 2015) (and this study). Here, we show that both the number and the length of LRs that the plant produces in response to an inoculation by *A. euteiches* are higher in the *Mtnf-ya1-1* mutant compared to the A17 accession and higher in the A17 as compared to the F83005.5. There is thus a negative correlation between the level of expression of *MtNF-YA1* and the capacity of the plant to initiate and elongate LRs upon inoculation.

Interestingly, the cytokinin receptor mutant *cre1* mutant is also more resistant to *A. euteiches* infection and displays more LRs upon infection, suggesting that this might be a cytokinin controlled mechanism (Laffont et al., 2015). Our transcriptome analysis showed that a certain number of cytokinin-related genes are mis-regulated in *Mtnf-ya1-1*. Among them 2 genes coding for cytokinin-*O*-glucosyltransferases (ZOG), i.e., *Medtr6g014660* and *Medtr3g111140.1* and a gene coding for a cytokinin oxidase, i.e., *Medtr4g118430.1* are significantly downregulated in *Mtnf-ya1-1* roots compared to WT (A17) roots, both before inoculation and one day after inoculation. In Rice the suppression of ZOGs leads to a decrease in fasciculated root production (Kudo et al., 2012) while contradicting reports about the positive and negative effect of cytokinin oxidase on root growth exist (Köllmer et al., 2014; Zalewski et al., 2014).

Importantly, ABA signaling is known to render *M. truncatula* susceptible to *A. euteiches* (Colditz et al., 2005; Schenkluhn et al., 2010). Among the genes down regulated in the *Mtnf-ya1-1* mutant compared to A17 both at day 0 and 1 day post inoculation, 6 encode ABA-related genes (Supplementary Table 3). Interestingly among them Medtr1g010210.1 encodes *LATERAL DEFICIENCY-LATD*. This nitrate transporter that could also transport ABA is required for nodule and LRs formation, (Bright et al., 2005; Liang et al., 2007; Harris and Dickstein, 2010; Bagchi et al., 2012).

Two auxin responsive genes, Medtr1g063950, encoding a SAUR protein and Medtr5g016320.1 encoding an indole-3-acetic acid-amido synthetase are also mis-regulated in *Mtnfya1-1* (Supplementary Table 3). However, these two genes are only differentially regulated at day 0, prior to inoculation and only Medtr1g063950 remained overexpressed in the mutant one day post inoculation whilst Medtr5g016320.1 is less expressed in mutant at 6 dpi.

Taken together we have shown correlations between the expression of genes from hormonal pathways potentially involved in LR development and the MtNF-YA1 transcription factor that need to be further studied to understand the role of this gene in root development upon *A. euteiches* invasion. Unlike the situation found during the nitrogen fixing symbiosis where *NF-YB* (Soyano et al., 2013) or mycorrhization where *NF-YC* (Hogekamp et al., 2011; Gaude et al., 2012; Hogekamp and Küster, 2013) genes have been described as up-regulated, we did not find any mis-regulated NF-YB and NF-YC subunit-encoding genes in our transcriptomic analysis. A number of *NF-Y* trimers formed with *MtNF-YA1* have been reported recently (Baudin et al., 2015). It is thus conceivable that the specificity of *MtNF-YA1* functions in symbiotic and pathogenic plant microbe interactions is achieved by the formation of different NF-Y trimmers. Also, it is likely that *MtNF-YA1* functions redundantly in the interaction with *A. euteiches*. This view is supported by the resistance triggered by *miR169q* overexpression in F83005.5 which is stronger than the one obtained via RNA interference. *miR169* is encoded by a large gene family in plants (14 genes in *Arabidopsis* leading to four different mature *miR169s*) and targets specifically the product of NF-YA genes with a certain degree of specificity (Sorin et al., 2014). It is thus likely that other *NF-YA* subunits such as MtNF-YA2 for example that is expressed in roots at a much higher level than *MtNF-YA1* are targeted by the miR169q in *M. truncatula* and play overlapping functions with *MtNF-YA1* in the susceptibility to *A. euteiches*.

### Genetic Engineering of Durable Disease Resistance with *MtNF-YA1*

Soils harbor diverse microbes engaging in contrasting interactions with plant roots (Rey and Schornack, 2013). Legumes establish mutualistic symbiosis with rhizobacteria or arbuscular mycorrhizal fungi or activate immune processes to fend off pathogens. How selectivity is achieved by the host plant to build up appropriate immune and symbiotic responses stays elusive, especially since many similarities exist in the colonization strategies of beneficial and detrimental partners (Evangelisti et al., 2014). In *M. truncatula, NF-YA1* knock out does not cause major developmental phenotypes for the plant but subtly modifies the associations with root inhabiting microbes. Knocking out *MtNF-YA1* to enhance resistance to *A. euteiches*, a major threat for pea and alfafa (Gaulin et al., 2007) is an appealing approach to engineer durable resistance (Le Fevre et al., 2015) but will preclude nitrogen fixing symbiosis. However, genome editing (Feng et al., 2013) in non-legume crops for orthologs of *MtNF-YA1* may become a fruitful biotech application of our findings.

## Author Contributions

TR, PL, M-FJ, MB, SH, and SB acquired and interpreted data. CJ, AN, BD, MB, and TR drafted the article. TR, CJ, and AN gave final approval of the version to be published.

## Conflict of Interest Statement

The authors declare that the research was conducted in the absence of any commercial or financial relationships that could be construed as a potential conflict of interest.

## Footnotes

1 https://imagej.nih.gov/ij/

2 http://www.quantprime.de

3 http://www.r-project.org/

4 http://mev.tm4.org/

5 http://mapman.gabipd.org/

6 http://medicagohapmap.org/

7 http://mtgea.noble.org/v3/

## Supplementary Material

**SUPPLEMENTARY DATA SHEET S1** | Reconstruction of *MtNF-YA1* sequences across HAPMAP accessions based on SNPs informations from the project (http://www.medicagohapmap.org/).
