# Supplementary material for: MtNF-YA1, A Central Transcriptional Regulator of Symbiotic Nodule Development, Is Also a Determinant of Medicago truncatula Susceptibility toward a Root Pathogen
Source: Front Plant Sci. 2016 Dec 5;7:1837. doi: 10.3389/fpls.2016.01837 (PMC5137509; doi:10.3389/fpls.2016.01837)
Supplement: Supplementary file 1 [file Data_Sheet_1.DOCX]

>A17

AAAAAAATAAAGGTTCTATACTCATTAAACAAAGTGCCAAATTCAGAGATACTACTTCCT

TTGGATAATTTGTATCGTCACCAACGATCTTTGAGTTATTTCAAAAATCACATTCTCAAT

TTTGTGGTCCAACATTCACAAAATGGTTTGTCGGGCTTTAAGTCTAGTAATTGTGAAACA

TGCATATGCATGAGGTAATTAATTGATAATCTGAAAAGGTTAAATAGTTAATCAACTGTA

CTTTGGTTATTTTTAATTTGTTCTCTATAATTTTTCAGTCAAAATTGAATTTGCTTTAAC

CATTTAGTGTAGTAGTATGATATTACTTGCTCTACATTCTTGGATCTAAAAGTCTAATTA

GTATCGTATAAAAAAAATTCTAGTTAATTAAGATACTTTTCTTCATTCTAATTATTCTTG

GATTACTTGTGAAACTTTTAGGGATGAACAAGAAAGTCCACTAAAGAGGCTTCTTTTTGC

TAGGAGTCACTAGTTAGTTTTTTTTTTTTTTGACAAAAGTTAACCATTTTTTTTTATCGC

ATGGTAGGAAAAAAAAAATAGAGATTTCCTTTGGTACTCATAAAAAAAAAAGTTGGATGG

ATAATGGATTAATCATAACTTATTTAACACAAAATGAATTAAGAGAACATTTGATATTTG

GGTTTATTTTGTAACATAAATATTAATATCAATGTTTGAAAGAGTTTTTAACAATAATTT

GTAAAAGAAAGCATAATATATCTATCAAAATAATTGTAAAAGAAAAAATTTCTTTTGGTG

GTATGACATGGAAAAATAATTGATTCTTATTAGATGAAAGTGTAAAATTAATTTGCATTA

ACAAAGCATATTTCATTAAACTCTTTTTAGTTTTTGTTGGAAAAAATAAATTTAATTTTA

CTCAAAAAATAAAAGTGTACAATTAATATATTTACTCAAAAGTCCCCTAAATGGGATGTA

ATTATCAAGGGATGTGAGCGGTTTAACTTTAAGTAATGAATCAACTGTTGCATAATCACT

TTGAAAGAAACTTGAGATTCTTCAATTAGCAAAATCTTGAATAGAATAATGAGATTATTC

CTAATGCTAATGATACCCACCTCTCCTATAAGTTGATGGGGCAGAACAAATTATGCACTC

AGGACAACTTTTCTCAAATTTTGAAAACTTACCACACGAAGAACACACTTCCACTTGCAC

AAGTAAGAATATAGTATAACAATAAATTTACCACTCATAGCTCAATGGATATTAAAATAT

TGCTAATCTTTTATAGACAGATGTTGGCAAATTAGTATAAATTAGTTATTATATTAGTTT

TTAAGGTTCAATCTACTTTTTAAAACTCTTATTAAGTTGTTTAGCTTGCCAACCAAAGAC

TAAATTATCTTTATTTGTTAGGAAATAAAAGCACTTATTTTATTTTCTCCATATTTGTTT

CCTCTTTGTTCCATTACTAAAAAAATTATAATATATTAGTAACAAAAAAATTTGGGGAGG

GAATGTTATTTTTAAAAAAGATGTTTTATTTATATTTAAGTGTTTTATTGAAATTCCTCG

TGAGTTTAGCTCAGTTGGTATGAACAATACATAATATATGCAAAATTCGAGGTTCAAACC

CTAAGCACCAAAAAAAAAAATGTTTTATTGAAAAATATCAAGAACTTGAAAACAAATTTC

CTTTTTATGAACTAGATATTGTCACATAGACAACTTAAGAATTGACATTTTTTTTAACAC

TATTGTATTTTCTGAACTCGACTTTAACTCGAGATCAATGAGTAAACTAAAAAAACTCGT

ATTATTTTATCTAAGTGTTCTTAGTGTGAGAAAACATTATAATTGTTTGCTTAGTCTATT

TCTCCCTATATTCTCACCCTCATTCTTGGTTTGCACATAGAAAGGAGAAAAAAAAGATTA

GAGAAGAGTTGAATGGAACAGAAAGAAAGAGCAAGCAAAATAATCACTAAAAAAAATTCC

CCTAAAAGAAAATCACTGAAAAATATTATCCACCTCTATTTTAAATAATAATAATAATGA

AAACAACAATATTTTTTTTTATAGATTTTCTCTACTAGAGGTAATTTATTTTAAACACTA

AATGTCCACACCTCTAACAAAAAAAATGCATAGTATTATAATAGATTATTGTAAGAAAGA

GTGACAAATGTTGAATTAGTTATGGAATGAAGAGTGAGAGAGATTATACAAATAAATAAA

TATAAATATAAATATAAATAAAGAATAGAGAAGAACATAGTACTTGAATGAAGAGATTTG

GCTTGTACTTCCATTACACTTGTAAGCTTTCTACTTCCTTCACATTCCTTCTCTTTCTCT

ATCTTTCCTTTTATGATATGTGTACTTTGTTATTGCTTCATTTACTACCGACATGACTCC

AACCTATTAGGGCTTTATCTGAATAATGTTAGTTATTTTCTCTCATTTTCTTTGCTTCTT

CTTATTCTTGGAACTTATAAAGGGTCTTGTCAATTCTATCATTCTCTTTTGTATACTTAC

AAATATAAATATTCTCTTTAGCTTCATTTTCATGTCTTCTTCTACTAATAAGCTATGGAA

ATTGAAGTTTTTGTTTTCATGTTTATTTTTCACTTCAGCTCCTCTTTTTTGATGTTGATG

CTGAAGATTGAATAAAAACTGAGGTGGTATGTATGAGAACAAGAACATTTTTCATTCATC

TCTGTTATTTGAACATCTAAATTTTAAACAACTATCGCGACAATTTTTTAAGTGGTTGAA

GTATTTTAAATAGGTGGTTAATTAAATTAAGTAGATAGTTAATTGATATATCAGGTGTCA

AAACTCATTAACCATCAACATAACTTCATTAGCCATGAATTTTTGACGTGATTAACCAAA

TATTGTAGATGCTCAATTACATGTAGATTTGATGGAGTTGTCCACAATTTCTCATATATC

TATTAACTAATAACCATCTACCGAATATAATTAACCATATATTAGAATATCATTAACCTC

TAAAACAGTTGTCACGAAAGTTGGTTGTACAAAAATCATTTCTATTTTTTACGCCACTAG

TTCTCTTGTTATTTCTCTTTGTTGGAAAGTTGTTGTTTTTAACTTTGAGCTTAGTCTTAT

TAATTAACTTGTAATGGTAACAACATTTTTCTATCTTTGTTTGAAGGAAGTAGTTACTGT

GTCAACTTTAGTAATTAATTTGGATCATTTTGGAGGCAAAATATGGCTATGCAACCTGTT

TATCTTAAAGAACATGAAGGAAATGTCCACAATTCTGTTGGACAGTTTTCATCTGTGACT

TCAGCACCATGGTGGAGTAATGCCTATGGATCTCAACCTGTTTATGGAGGAGACTCTTGT

GGCCAAATGAAACCTTTTTCACTAGAGCTTTCCAACTACATAGACCAACTTGCTCCGAGT

AAGAACTTAGTTCGAGGAGTTGAACAATTGTTTGATAAAGGGCATACAAACCAATTCACC

ATCTTTCCAGGTACTTGTTATTCAATATAATTCCGGTTTTGAACGAATTGAATTTTGTTA

AGTTGGTTCTGGCTAAACGTGCGTTAAATATAATATGATTTATGTTTGGATACGTTTATG

TAAAAGTGAGTTGAACATAAATTGGAGACTAAATATCAATTGCAGAGGCAAAAGCTTCAA

ATTCTAATTTCAAGTTAGAATCAAACCAATTCTACTCGTAAGCATCTAAATATTTCAAAA

CCAATTCTACATCTCTAGAACTAAATTTGCATTCAAACATTTTCTGCTACAGATGATTGT

AAGATGTCAGTTGATACACCAAATCATCAAGCAACCTTATCCCTGCAATCACCATTTGCT

GCCGAGCCACTTAATCGATTTGAGCTAGGTTTTAACCAGTCTATGGTAAATCTTCTTATT

AAAGCAATCCCTTTGATCATGTTTTAGTATTTTACTTTTGATGTACATAAATTCATTATG

TTTTTATTATCCTTTCCATGCTAGATCTGTGCAAAATATCCGTATATGGATCAATTTTAC

GGGCTCTTCTCGACTTATGGACCTCAAATCTCGGTTTGTCTTCACCTCGGCTATTTTATT

AATCTTTGTATATATCATGCAATGTCTATAAGTACTTGAATGTGAATTTGTTTTATTTAC

AGTGTGTATGCTTACATGAGAAAGCCAGAAATCTGTTTACAACTATCTTGTTAGTATTCT

TCTAAATCTTTCAAATGTAGAGTTGATTATGAAATTTGTTGTTAATGAGTTTTCGGCTTT

ATGAATGAATTGTAAGACAAGTAATGAAACAAAAACTAGAAGAAAATTGAATTATATGAT

GCAATCTGTGTTGAAGTTTTTGCATGTATTCAAGTTCTGCTTTTAGTTACTATTTGTTTA

TCTGCTGCAATATTTCATCAATTATTATTCATGTATGTACCAATATTTAATAAATTAGGG

GCGAATTATGCTTCCGCTTAGCATGACATCTGACGACGGACCAACATACGTGAATGCTAA

GCAATACCATGGAATCATCAGACGCAGGCATTCTCGTGCCAAAGCTGTGCTTCAGAATAA

ATTGATAAAGCGTAACAAGGTATGAAACTGAGTATTTTTCCTCACAACAATGTTCGAAAA

CTTGTGAAATAGTATATTTTTCCTTTTACCCTTTTTATACTTATATTAAGGTTTTGTATT

TGTCTTGCAGCCATATATGCACGAATCGCGTCATCTACATGCAATGCGTAGACCAAGAGG

ATGCGGTGGTCGTTTCTTGAACACAAAAGTTTCTGCTAATGGAAACGGTAAAAGCGGGAG

TGAAGAGAACGGAAACATTGGTGGCCTACAGCTGCAGTCCAGTGGTTCTCAGAGTTCCGA

AGTCTTACAATCTGAGGTTGGAACTTTAAATTCGTCGAAGGAGACAAACGGAGGCAGTCC

AAATGTCTCGGGGTCAGAGGTGACTAGCATGTATACACAGGGAGTTCTTGATAGCTTTAC

TGTCAATCATATCGGATCTACTGTCCACTCTTTGGGAGACATGATCGATACTGGACACGG

TATCGTCATGCCAACAAAATGGTTTGCAGCAGCTGGCAGACAGCTGCTGGAACCATAAGT

TTCGATTCAGAAAGGAAACAAGTGGGTTTGGTACAATGTGAAATATTTTGCACCAAACTC

ATCCTTTCCGAGACCAGATGAAGAAGCTATGTTTCAGTTTGTTGTGTTTACTACGACAAA

TTTAGTTTCGGAAGACTACTTTTCCATCTGGTGCTCAGGCAACTCATTCTTGGCTTATTC

TCAGGAAACTCATCCTTGGCTCGTAATATTTAGTAGTATTGTCATTGTCTTTCCGCGCAG

GCTTGCCGTGGCATGGTAGGCATGCTAATGACTTTGGTATTTTCATGCAGTTATAACTAT

GATGTGTCTTTGTTTGTTGTTAAAATAAAAAACATGAACTCTAGCTAGGTGCATGTGTGT

GTTTTTAATCTTGTCTACTAAGTTTGGTGTTTTGTAATGGATTTCTGACTTTATGGAGCA

ATGTATTGTAACTCTACTAAGAAGTGTAACATTTTATTTCTCCCTCTCTAAGGATTGTAT

AAGAACCTCTTATTTTCAGACTCTACTTAATCCTATTTTCTATGTCTGTATGATTTTTAT

ATTTCTAGGACAATCAAATTGGCTTGTAGAAGCTCAAAAGTATGCTCACAAAATAGGTAC

TTATGTAGGGAACTTCGTACTCTAATAATAACTGGTTATTAACGTTATAATTAAATGCAA

AATTTGATAAGTAGTAGGGTTTGGTAAGTAATATAACAACACCATGTTGGCTTGGGATTC

TGAATTGTGTTATTGGAGTACAACTCTTAAATAATGATCGTGATGTAAATAATTTAAATT

TGAAGTTACTAGATAAAAAGATAACATTATGTAATATATTATCAAATATATACGAAAAAT

ATAATTTTCATCTATCTCAATTGAAAAAATATCCGGGTTTTGGGTCTATTGTCAATGACG

A

>F83005.5

AAAAAAATAAAGGTTCTATACTCATTAAACAAAGTGCCAAATTCAGAGATACTACTTCCT

TTGGATAATTTGTATCGTCACCAACGATCTTTGAGTTATTTCAAAAATCACATTCTCAATT

TTGTGGTCCAACATTCACAAAATGGTTTGTCGAGCTTTAAGTCTAGTAATTGTGAAACATG

CATATGCATGAGGTAATTAATTGATAATCTGAAAAGGTTAAATAGTTAATCAATTGTACTT

TGGTTCTTTTTAATTTGTTCTCTATAATTTTTCAGTCAAAATTGAATTTGCTTTAACCATT

TAGTGTAGTAGTATGATATTACTTGCTCTACATTCTTGGATCTAAAGGTCTAATTAGTATC

GTATAAAAAATATTCTAGTTAATTAAGATACTTTTGTTCATTCTAATTATTCTTGGATTAC

TTGTGAAACTTTTAGGGATGAACAAGAAAGTCCACTAAAGAGGCTTCTTTTTGCTAGGAGT

CACTAGTTAGTTTTTTTTTTTTTTGACAAAAGTTAACCATTTTTTTTTATCGCATGGTAGG

AAAAAAAAAAAAGAGATTTCCTTTGGTACTCATAAAAAAAAAAGTTGGATGGATAATGGAT

TAATCATAACTTATTTAACATAAAATGAACTAAGAGAACATTTCATATTTGGGTTTATTTT

GTAACATAAATATTAATATCAATGTTTGAAAGAGTTTTTAACAATAATTTGTAAAAGAAAG

CATAATATATCTATCAAAATAATTATAAAAGAAAAAATTTCTTTTGGTGGTATGACATGGA

AATATAATTAATTCTTATTAGATGAAAGTGTAAAATTAATTTGCATTAACAAAGCATATTT

CATTAAACTCCATTTAGTTTTTGTTGGAAAAAATAAATTTATTGTTACTCAAAATATAAAA

GTGTACAATTAATATATTTACTCAAAAGTCCCCTAAATGGGATGTAATTATCAAGGGATGT

GAGCGGTTTAACTTTAAGTAATGAATCAACTGTTGCATAATCACTTTGAAAGAAACTTGAG

ATTCTTCAATTAGCAAAATCTTGAATAGAATAATGAGATTATTCCTAATGCTAATGATACC

CACCTCTCCTATAAGTTGATGGGGCAGAACAAATTATGCACTCAGGACAACTTTTCTCAAA

TTTTGAAAACTTACCACACGAAGAACACACTTCCACTTGCACAAGTAAGAATATAGTATAA

CAATAAATTTACCACTCATAGCTCAATGGATATTAAAATATTGCTAATCTTTTATAGACAG

ATGTTGGCAAATTAGTATAAATTAGTTATTATATTAGTTTTTAAGGTTCAATATACTTTTT

AAAACTCTTATTAAGTTGTTTAGCTTGCCAACCAAAGACTAAATTATCTTTATTTGTTAGG

AAATAAAAGCACTTATTTTATTTTCTCCATATTTGTTTCCTCTTTGTTCCATTACTAAAAA

AATTATAATATATTAATAACAAAAAAATTTGGGGAGGGAATGTTATTTTTAAAAAAGATGT

TTTATTTATATTTAAGTGTTTTATTGAAATTCCTCGTGAGTTTAGCTCAATTGGTATGGAC

AATACATAATATATGCAAAATTCGAGGTTCAAACCTTGAGCACCACAAAAAAAAGTGTTTT

ATTGAAAAATATCAAGAACTTGAAAACAAATTTCCTTTTTATGAACTAAATATTGTCACAT

AGACAACTTAAGAATTGACTTTTTTTTTAACACTATTGTATTTTCTGAACTCGACTTTAAC

TCGAGATCAATGAGTAAACTAAAAAAACTCGTATTATTTTATCTAAGTGTTCTTAGTGTGA

GAAAACATTATAATTGTTTGCTTAGTCTATTTCTCCCTATATTCTCACCCTCATTCTTGGT

TTGCACATAGAAAGGAGAAAAAAAAAATTAGAGAAGAGTTGAATGGAACAGAAAGAAAGAG

CAAGCAAAATAATCACTAAAAAAAATTCCCCTAAAAGAAAATCACTGAAAAATATTATCCA

CCTCTATTTTAAATAATAATAATAATGAAAACAACAATATATTTTTTTATAGATTTTCTCT

ACTAGAGGTAATTTATTTTAAACACTAAATGTCCACACCTCTAACAAAAAAAATGCATAAT

ATTATAATATATTATTGTAAGAAAGAGTGACAAATGTTGAATTAGTTATGGAATGAAGAGT

GAGAGAGATTATACAAATAAATAAATATAAATATAAATATAAATAAAGAATAGAGAAGAAC

ATAGTACTTGAATGAAGAGATTTGGCTTGTACTTCCATTACACTTGTAAGCTTTCTACTTC

CTTCACATTCCTTCTCTTTCTCTATCTTTCCTTTTATGATATGTGTACTTTGTTATTGCTT

CATTTACTACCGACATGACTCCAACCTATTAGGGCTTTATCTGAATAATGTTAGTTATTTT

CTCTCATTTTCTTTGCTTCTTCTTATTCTTGGAACTTATAAAGGGTCTTGTCAATTCTATC

GTTCTCTTTTGTATACTTACAAATATAAATATTCTCTTTAGCTTCATTTTCATGTCTTCTT

CTACTAATAAGCTATGGAAATTGAAGTATTTGTTTTCATGTTTATTTTTCACTTCAGCTCC

TCTTTTTTGATGTTGATGCTGAAGATTGAATAAAAACTGAGGTGGTATGTATGAGAACAAG

AACATGTTTCATTCATCTCTGTTATTTGAACATCTAAATTTTAAACAACTATCGCGACAAT

TTTTTAAGTGGTTGAAGTATTTTAAATAGGTGGTTAATTACATTAAGTAGATAGTTAATTG

ATATATCAGGTGTCAAAACTCATTAACCATCAACATAACTTCATTAGCCATAAATTTTTGA

CGTGATTAACCAAATATTGTAGATGCTCAATTACATGTAGATTTGATGGAGTTGTCCACAA

TTTCTCATATATATATTAACTAATAACCATCTACCGAATATAATTAACCATATATTAGAAT

ATCATTAACCTCTAAAACAGTTGTCGCGAAAATTGGTTGTAAAAAAATCATTTCTATTTTT

TACGCCACTAGTTCTCTTGTTATTTCTCTTTGTTGGAAAGTTGTTGTTTTTAACTTTGAGC

TTAGTCTTATTAATTAACTTGTAATGGTAACAACATTTTTCTATCTTTGTTTGAAGGAAGT

AGTTACTGTGTCAACTTTAGTAATTAATTTGGATCATTTTGGAGGCAAAATATGGCTATGC

AACCTGTTTATTTTAAAGAACATGAAGGAAATGTCCACAATTCTGTTGGACAGTTTTCATC

TGTGACTTCAGCACCATGGTGGAGTAATGCCTATGGATCTCAACCTTTTTATGGAGGAGAC

TCTTGTGGCCAAATGAAACCTTTTTCACTAGAGCTTTCCAACTACATAGACCAACTTGCTC

CGAGTAAGAACTTAGTTCGAGGAGTTGAACAATTGTTTGATAAAGGGCATACAAACCAATT

CACCATCTTTCCAGGTACTTGTTATTCAATATAATTCCGGTTTTGAATGAATTGATTTTTG

TTAAGTTGGTTCTGGATAAACGTGCGTTAAATATAATATGATTTATGTTTGGATACGTTTA

TGTTAAAGTGAGTTGAACATAAATTGGAGACTAAATATCAATTGCAGAGGCAAAAGCTTCA

AATTCTAATTTCAAGTTAGAATCAAATCAATTCTACTCGTAAGCATCTAAATGTTTCAAAA

CCAATTCTACATCTCTAGAACTAAATTTGCATTCAAACATTTTCTGCTACAGATGATTGTA

AGATGTCAGTTGATACACCAAATCATCAGGCAACCTTATCCCTGCAATCACCATTTGCTGC

CGAGCCACTTAATCGATTTGAGCTAGGTTTTAACCAGTCTATGGTAAATCTTCTTATTAAA

GCAATCCCTTTGATCATGTTTTAGTATTTTACTTTTGATGTACATAAATTCATTATGTTTT

TATTATCCTTTCCATGCTAGATCTGTGCAAAATATCCGTATATAGATCAATTTTACGGGCT

CTTCTCGACTTATGGACCTCAAATCTCGGTTTGTCTTCACCTCGGCTATTTTATTAATCTT

TGTATATATCATGCAATGTCTATAAGTACTTGAATGTGAATTTGTTTTATTTACAGTGTGT

ATGCTTACATGAGAAAGCCAGAAATCTGTTTACAACTATCTTGTTAGTATTCTTCTAAATC

TTTCAAATGTAGAGTTGATTATGAAATTTGTTGTTAATGAGTTTTCGGCTTTATGAATGAA

TTGTAAGACAAGTAATGAAACAAAAACTAGAAGAAAATTGAATTATATGATGCAATCTGTG

TTGAAGTTTTTGCATGTATTCAAGTTCTGCTTTTAGTTACTATTTGTTTATCTGCTGCAAT

ATTTCATCAATTATTATTCATGTATGTACCAATATTTAATAAATTAGGGGCGAATTATGCT

TCCGCTTAGCATGACATCTGACGACGGACCAACATACGTGAATGCTAAGCAATACCATGGA

ATCATCAGACGCAGGCATTCTCGTGCCAAAGCTGTGCTTCAGAATAAATTGATAAAGCGTA

ACAAGGTATGAAACTGAGTATTTTTCCTCACAACAATGTTCGAAAACTTGTGAAATAGTAT

ATTTTTCCTTTTACCCTTTTTATACCTATATTAAAGTTTTGTATTTGTCTTGCAGCCATAT

ATGCACGAATCGCGTCATCTACATGCAATGCGTAGACCAAGAGGATGCGGTGGTCGTTTCT

TGAACACAAAAGTTTCTGCTAATGGAAACGGTAAAAGTGGGAGTGAAGAGAACGGAAACAT

TGGTGGCCTACAGCTGCAGTCCAGTGGTTCTCAGAGTTCCGAAGTCTTACAATCTGAGGTT

GGAACTTTAAATTCGTCGAAGGAGACAAACGGAGGCAGTCCAAATGTCTCGGGGTCAGAGG

TGACTAGCATGTATACACAGGGAGGTCTTGATAGCTTTACTGTCAATCATATCGGATCTAC

TGTCCACTCTTTGGGAGACATGATCGATACTGGACACGGTATCGTCATGCCAACAAAATGG

TTTGCAGCAGCTGGCAGACAGCTGCTGGAACCATAAGTTTCGATTCAGAAAGGAAACAAGT

GGGTTTGGTACAATGTGAAATATTTTGCACCAAACTCATCCTTTCCGAGACCAGATGAAGA

AGCTATGTTTCAGTTTGTTGTGTTTACTACGACAAATTTAGTTTTGGAAGACTACTTTTCC

ATCTGGTGCTCAGGCAACTCATTCTTGGCTTATTCTCAGGAAACTCATCCTTGGCTCGTAA

TATTTAGTAGTATTGTCATCGTCTTCCCGCGCAGGCTTTCCGTGGCATGGTAGGCATGCTA

ATGACTTTGGTATTTTCATGCAGTTATAACTATGATGTGTCTTTGTTTGTTGTTAAAATAA

AAAACATGAACTCTAGCTAGGTGCATGTGTGTGTTTTTAATCTTGTCTACTAAGTTTGGTG

TTTTGTAATGGATTTCTGACTTTATGGAGCAATGTATTGTAACTCTACTAAGAAGTGTAAC

ATTTTATTTCTCCCTCTCTAAGGATTGTATAAGAACCTCTTATTTTCAGACTCTACTTAAT

CCTATTTTCTATGTCTGTATGATTTTTATATTTCTAGGACAATCAAATTGGCTTGTAGAAG

CTCAAAAGCATGCTCACAAAGTAGGTACTTATGTAGGGAACTTCGTACTCTAATAATAACT

GGTTATTAACGTTATAATTAAATGCAAAATTTGATAAGTAGTAGGGTTTCGTAAGTAATAT

AACAACACCATATTGGCTTGGGATTCTGAATTGTGTTATTAGAGTACAACTTTTAAATAAT

GATCGTAATGTAAATAACTTAAATTTGAAGTTACTAGATAAAAAGATAATATTATGTAATA

TATTATCAAATATGTACTAAAAATGTAATTTTCATCTATCTCAATTGAAAAAATATATGAG

TTTTGAGTCTATTGTCATGGACGA

>HM001

AAAAAAATAAAGGTTCTATACTCATTAAACAAAGTGCCAAATTCAGAGATACAACTTCCT

TTGGATAATTTGTATCGTCACCAACGATCTTTGAGTTATTTCAAAAATCACATTCTCAATT

TTGTGGTCCAACATTCACAAAATGGTTTGTCGAGCTTTAAGTCTAGTAATTGTGAAACATG

CATATGCATGAGGTAATTAATTGATAATCTGAAAAGGTTAAATAGTTAATCAATTGTACTT

TCGTTCTTTTTAATTTGTTCTCTATAATTTTTCAGTCAAAATTGAATTTGCTTTAACCATT

TAGTGTAGTAGTATGATATTACTTGCTCTACATTCTTGGATCTAAAGGTCTAATTCGTATC

GTATAAAAAATATTCTAGTTAATTAAGATACTTTTGTTCATTCTAATTATTCTTGGATTAC

TTGTGAAACTTTTAGGGATGAACAAGAAAGTCCACTAAAGAGGCTTCTTTTTGCTAGGAGT

CACTAGTTAGTTTTTTTTTTTTTTGACAAAAGTTAACCATTTTATTATGACGCATGGTAGG

AAAAAAAAAAAAGAGATTTCCTTTGGTACTCATAAAAAAAAAAATTGGATGGATAATGGAT

TAATCATAACTTATTTAACATAAAATGAACTAAGAGGGCATTTCATATTTGGGTTTATTTT

GTAACATAAATATTAATATCAATGTTTGAAAGAGTTTTTAACAATAATTTGTAAAAGAAAG

CATAATATATCTATCAAAATAATTATAAAAGAAAAAATTTCTTTTGGTGGTATGACATGGA

AATATAATTAATTCTTATTAGATGAAAGTGTAAAATTAATTTGCATTAACAAAGCATATTT

CATTAAACTCCATTTAGTTTTTGTTGGAAAAAATAAATTTATTGTTACTCAAAATATAAAA

ATGTACAATTAATATATTTACTCAAAAGTCCCCTAAATGGGATGTAATTATCAAGGGATGT

GAGCGGTTTAACTTTAAGTAATGAATCAACTGTTGCATAATCACTTTGAAAGAAACTTGAG

ATTCTTCAATTAGCAAAATCTTGAATAGAATAATGAGATTATTCCTAATGCTAATGATACC

CACCTCTCCTATAAGTTGATGGGGCAGAACAAATTATGCACTCAGGACAACTTTTCTCAAA

TTTTGAAAACTTACCACACGAAGAACACACTTCCACTTGCACAAGTAAGAATAGAGTATAA

CAATAAAATTACCACTCATAGCTCAATGGATATTAAAATATTGCTAATCTTTTATAGACAG

ATGTTGGCAAATTAGTATAAATAAGTTATTATATTAGTTTTTAAGGTTCAGTATACTTTTT

AAAACTCTTATTAAGTTGTTTAGGTTACCAACCAAAGACTAAATTATCTTTATTTGTTAGG

AAATAAAAGCACTTATTTTATTTTCTCCATATTTGTTTCCTCTTTGTTCCATTACTAAAAA

AATTATAATATATTAATAACAAAAAATTTTGGGGAGGGAATGTTATTTTTAAACAAGATGT

TTTATTTATATTTAAGTGTTTTATTGAAATTCCTCGTGAGTTTAGCTCAATTGGTATGGAC

AATACATAATATATGCAAGATTCGAGGTTCAAACCTTGAACACCACCAAAAAATGTGTTTT

ATTAAAAAATATCAAGAACTTGAAAACAAATTTTCTTTTTATGAACTAAATATTGTCACAT

AGACAACTTAAGAACTGACTTTTTTTTTAACACTATTGTATTTTCTGAACTCGACTTTAAC

TCGAGATCAATGAGTAAACTAAAAAAACTCGTATTATTTTATTCAAGTATTCTTAATGTGA

GAAAACATTATAATTGTTTGCTTAGTCTATTTCTCCCTATATTCTCACCCTCATTCTTGGT

TTGCACATAGAAAGGAGAAAAAAAAAATTAGAGAAGAGTTGAATGGAACAGAAAGAAAGAG

CAAGCAAAATAATCACTAAAAAAAATTCCCCTAAAAGAAAATCACTGAAAAATATTATCCA

CCTCTATTTTAAATAATAATAATAATGAAAACAACAATTTATTTTTTTATAGATTTTCTCT

ACTAGAGGTAATTTATTTTAAACACTAAATGTCCACACCTCTAACAAAAAAAATGCATAAT

ATTATAATATATTATTGTAAGAAAGAGTGACAAATGTTGAATTAGTTATGGAATGAAGAGT

GAGAGAGATTATACAAATAAATAAATATAAATATAAATATAAATAAAGAATAGAGAAGAAC

ATAGTACTTGAATGAAGAGATTTGGCTTGTACTTCCATTACACTTGTAAGCTTTCTACTTC

CTTCACATTCCTTCTCTTTCTCTATCTTTCCTTTTATGATATGTGTACTTTGTTATTGCTT

CATTTACTACCGACATGACTCCAACCTATTAGGGCTTTATCTGAATAACGTTAGTTATTTT

CTCTCATTTTCTTTGCTTCTTCTTATTCTTGGAACTTATAAAGGGTCTTGTCAATTCTATC

GTTCTCTTTTGTATACTTACAAATATAAATATTCTCTTTAGCTTCATTTTCATGTCTTCTT

CTACTAATAAGCTATGGAAATTGAAGTATTTGTTTCCATGTTTATTTTTCACTTCAGCTCC

TCTTTTTTTATGTTGATGCTGAAGATTGAATAAAAACTGAGGTGGTATGTATGAGAACAAG

AACATGTTTCATTCATCTCTGTCATTTGAACATCTAAATTTTAAACAATTATCGCAACAAT

TTTTTAAGTGGTTGAAGTATTTTAAATAGGTGGTTAATTACATTAAGTAGATAGTTAATTG

ATATATCAGGTGTCAAAACTCATTAACCATCAACATAACTTCATTAGCCATAAATTTTTGA

CGTGATTAACCAAATAGTGTAGATGCTCAATTACATGTAGATTTGATGGAGTTGTCCACAA

TTTCTCATATATATATTAACTAATAACCATCTACCGAATATAATTAACCATATATGAGAAT

AACATTAACCTCTAAAACAGTTGTCGTGAAAATTGATTGTGAAAAAATCATTTCTATTTTT

TACGCCATTAGTTCTCTTGTTATTTCTGTTTGTTGGAAAGTTGTTGTTTTTAACTTTGAGC

TTAGTCTTATTAATTAACTTGTAATGGTAACAACATTTTTCTATCTTTGTTTGAAGGAAGT

AGTTACTGTGTCAACTTTAGTAATTAATTTGGACTATTTTGGAGGAAAAATATGGCTATGC

AACCTGTTTATTTTAAAGAACATGAAGGAAATGTCCACAATTCTGTTGGACAGTTTTCATC

TGTGACTTCAGCACCATGGTGGAGTAATGCCTATGGATCTCAACCTTTTTATGGAGGAGAC

TCTTGTGGCCAAATGAAACCTTTTTCGCTAGAGCTTTCCAACTACATAGACCAACTTGCTC

CGAGTAAGAACTTAGTTCGAGGAGTTGAACAATTGTTTGATAAAGGGCATACAAACCAATT

CACCATCTTTCCAGGTACTTGTTATTCAATATAATTCCGGTTTTGAATGAATTGATTTTTG

TTAAGTTTGTTCTGGATAAACGTGCATTAAATATAATATAATTTATGTTTGGATACGTTTA

TGTTAAAGTGAGTTGAACATAAATTGGAGTCTAAATATCAATTGCAGAGGCAAAAGCTTCA

AATTCTAATTTCAAGTTAGAATCAAATCAATTCTACTCGTAAGCCTATAAATGTTTCAGAA

CCAATTCTACATGTCTAGAACTAAATTTGCATTCAAACATTTTCTGCTACAGATGATTGTA

AGATGTCAGTTGATACACCAAATCATCAGGCAACCTTATCCCTGCAATCACCATTTGCTGC

TGAGCCACTTAATCGATTTGAGCTAGGTTTTAACCAGTCTGTGGTAAATCTTCTTATTAAA

GCAATCCCTTTGATCATGTTTTAGTATTTTACTTTTGATGTACATAAATTCATTATGTTTT

TATTATCCTTTCCATGCTAGATCTGTGCAAAATATCCGTATATAGATCAATTTTACGGGCT

CTTCTCGACTTATGGACCTCAAATCTCGGTTTGTCTTCACCTCGGCTCTTTTATTAATCTT

TGTATATATCATGCAATGTCTATAAGTACTTGAATGTGAATTTGTTTTATTTACAGTGTGT

ATGCTTACATGAGAAAGCCAGAAATCTGTTTACAACTATCTTGTTAGTATTCTTCTAAATC

TTTCAAATGTAGAGTTGATTATGAAATTTGTTGTTAATGAGTTTTCGGCTTTATGAATAAA

TTGTAAGACAAGTAATGAAACAAAAACTAGAAGAAAATTGAATTATATGATGCAATCTGTG

TTGAAGTTTTTGCATGTATTCAAGTTCTGCTTTTAGTTACTATTTGTTTATCTGCTGCAAT

ATTTCATCAATTATTATTCATGTATGCACCAATACTTAATAAATTAGGGGCGAATTATGCT

TCCGCTTAGCATGACATCTGACGACGGACCAACATATGTGAATGCTAAGCAATACCACGGA

ATCATCAGACGCAGGCATTCTCGGGCCAAAGCTGTGCTTCAGAATAAATTGATAAAGCGTA

ACAAGGTAGGAACATGAGTATTTTCCCTCACAGCAATGTTTGAAAACCTGTGAAATAGTAT

ATTTTTCCTTTTACCCTTTTTATACCTATATTAAAGTTTTGTATTTTTCTTGCAGCCATAT

ATGCACGAATCGCGTCATCTACATGCAATGCGTAGACCAAGAGGATGCGGTGGTCGTTTCT

TGAACACAAAAGTTTCTGCTAATGGAAACGGTAAAAGTGGGAGTGAAGAGAACGGAAACAT

TGGTGGCCTACAGCTGCAGTCCAGTGGTTCTCAGAGTTCCGAAGTCTTACAATCTGAGGTT

GGAACTTTAAATTCGTCGAAGGAGACAAACGGAGGCAGTCCAAATGTCTCGGGGTCAGAGG

TGACTAGCATGTATACACAGGGAGGTCTTGATAGCTTTACTGTCAACCATATCGGATCTAC

TGTCCACTCTTTGGGAGACATGATCGATACTGGACACGGTATCGTCATGCCAACAAAATGG

TTTTCAGCAGCTGGCAGACAGCTGCTGGAACCTTAAGTTTCGATTCAGAAAGGAAACAAGT

GGGTTTGGTACAATGTGAAACATTTTGCACCAAACTCATCCTTTCCGAGACCGGATGAAGA

AGCTATGGTTCAGTTTGTTGTGTTTACTACGACAAATTTAGTATTGGAAGCCTGCTTTTCC

ATCTGGTGCTCAGGCAACTCATTCTTGGCTTATTCTCAGGAAACTTATCCTTGGCTCGTAA

TATTTAGTAGTATTGTCATCGTCTTCCTACGCAGGCTTTCCGTGGCATGGTAGGCATGCTA

ATGACTTTGGTATTTGCATGCAGTTATAACTATGATGTGTGTTTGTTTGTTGTTAAAATAA

AAAACATGAACTCTAGCTAGGTGCATGTGTGTGTTTTTAATCTTGTCTAGTAAGTTTGGTG

TTTTGTAATGGATTTCTGACTTTATGGAGCAATGCATTGTAACTCAACTAAGAAGTGTACC

ATTTTATATCTCCCTCTCTAAGGATTGTATAAGAACCTCTTATTTTCAGACTCTACTTAAT

CCTATTTTCTATGTCTGTATGATTTTTATATTTCTAGGACAATCAAATTGGCTTGTAGAAG

CCCAAAAGCATGCTCACAAAGTAGGTACATATGTAGGGAACTTCGTACTCTAATAATAACT

GGCTATTAACGTTATAATTAAATGCAAAATTTGATAAGTAGTTGGGTTTCGTAAGTAATAT

AACAACACCATATTTACTTGGGATTCTGAATTGTGTTATTAGAGTATAACTTTTAAATAAT

GATCCTAATGTAAATAACCTAAATTTGAAGTTAATAGATAAAAAGATAATATTATGTAATA

TATTATCAAATATGTACTAAAAATGTAATTTTCATCTATCTCAATTGAAAAAATATATGAA

TTTTAAGTCTATTGTCATGGACGG

>HM002

AAAAAAATAAAGGTTCTATACTCATTAAACAAAGTGCCAAATTCAGAGATACTACTTCCT

TTGGATAATTTGTATCGTCACCAACGATCTTTGAGTTATTTCAAAAATCACATTCTCAATT

TTGTGGTCCAACATTCACAAAATGGTTTGTCGAGCTTTAAGTCTAGTAATTGTGAAACATG

CATATGCATGAGGTAATTAATTGATAATCTGAAAAGGTTAAATAGTTAATCAATTGTACTT

TCGTTCTTTTTAATTTGTTCTCTATAATTTTTCAGTCAAAATTGAATTTGCTTTAACCATT

TAGTGTAGTAGTATGATATTACTTGCTCTACATTCTTGGATCTAAAGGTCTAATTCGTATC

GTATAAAAAATATTCTAGTTAATTAAGATACTTTTGTTCATTCTAATTATTCTTGGATTAC

TTGTGAAACTTTTAGGGATGAACAAGAAAGTCCACTAAAGAGGCTTCTTTTTGCTAGGAGT

CACTAGTTAGTTTTTTTTTTTTTTGACAAAAGTTAACCATTTTATTATGACGCATGGTAGG

AAAAAAAAAAAAGAGATTTCCTTTGGTACTCATAAAAAAAAAAATTGGATGGATAATGGAT

TAATCATAACTTATTTAACATAAAATGAACTAAGAGAACATTTCATATTTGGGTTTATTTT

GTAACATAAATATTAATATCAATGTTTGAAAGAGTTTTTAACAATAATTTGTAAAAGAAAG

CATAATATATCTATCAAAATAATTATAAAAGAAAAAATTTCTTTTGGTGGTATGACATGGA

AATATAATTAATTCTTATTAGATGAAAGTGTAAAATTAATTTGCATTAACAAAGCATATTT

CATTAAACTCCATTTAGTTTTTGTTGGAAAAAATAAATTTATTGTTACTCAAAATATAAAA

GTGTACAATTAATATATTTACTCAAAAGTCCCCTAAATGGGATGTAATTATCAAGGGATGT

GAGCGGTTTAACTTTAAGTAATGAATCAACTGTTGCATAATCACTTTGAAAGAAACTTGAG

ATTCTTCAATTAGCAAAATCTTGAATAGAATAATGAGATTATTCCTAATGCTAATGATACC

CACCTCTCCTATAAGTTGATGGGGCAGAACAAATTATGCACTCAGGACAACTTTTCTCAAA

TTTTGAAAACTTACCACACGAAGAACACACTTCCACTTGCACAAGTAAGAATAGAGTATAA

CAATAAATTTACCACTCATAGCTCAATGGATATTAAAATATTGCTAATCTTTTATAGACAG

ATGTTGGCAAATTAGTATAAATTAGTTATTATATTAGTTTTTAAGGTTCAGTATACTTTTT

AAAACTCTTATTAAGTTGTTTAGCTTGCCAACCAAAGACTAAATTATCTTTATTTGTTAGG

AAATAAAAGCACTTATTTTATTTTCTCCATATTTGTTTCCTCTTTGTTCCATTACTAAAAA

AATTATAATATATTAATAACAAAAAAATTTGGGGAGGGAATGTTATTTTTAAACAAGATGT

TTTATTTATATTTAAGTGTTTTATTGAAATTCCTCGTGAGTTTAGCTCAATTGGTATGGAC

AATACATAATATATGCAAGATTCGAGGTTCAAACCTTGAACACCACAAAAAAATGTGTTTT

ATTGAAAAATATCAAGAACTTGAAAACAAATTTCCTTTTTATGAACTAAATATTGTCACAT

AGACAACTTAAGAATTGACTTTTTTTTTAACACTATTGTATTTTCTGAACTCGACTTTAAC

TCGAGATCAATGAGTAAACTAAAAAAACTCGTATTATTTTATCCAAGTATTCTTAGTGTGA

GAAAACATTATAATTGTTTGCTTAGTCTATTTCTCCCTATATTCTCACCCTCATTCTTGGT

TTGCACATAGAAAGGAGAAAAAAAAAATTAGAGAAGAGTTGAATGGAACAGAAAGAAAGAG

CAAGCAAAATAATCACTAAAAAAAATTCCCCTAAAAGAAAATCACTGAAAAATATTATCCA

CCTCTATTTTAAATAATAATAATAATGAAAACAACAATATATTTTTTTATAGATTTTCTCT

ACTAGAGGTAATTTATTTTAAACACTAAATGTCCACACCTCTAACAAAAAAAATGCATAAT

ATTATAATATATTATTGTAAGAAAGAGTGACAAATGTTGAATTAGTTATGGAATGAAGAGT

GAGAGAGATTATACAAATAAATAAATATAAATATAAATATAAATAAAGAATAGAGAAGAAC

ATAGTACTTGAATGAAGAGATTTGGCTTGTACTTCCATTACACTTGTAAGCTTTCTACTTC

CTTCACATTCCTTCTCTTTCTCTATCTTTCCTTTTATGATATGTGTACTTTGTTATTGCTT

CATTTACTACCGACATGACTCCAACCTATTAGGGCTTTATCTGAATAACGTTAGTTATTTT

CTCTCATTTTCTTTGCTTCTTCTTATTCTTGGAACTTATAAAGGGTCTTGTCAATTCTATC

GTTCTCTTTTGTATACTTACAAATATAAATATTCTCTTTAGCTTCATTTTCATGTCTTCTT

CTACTAATAAGCTATGGAAATTGAAGTATTTGTTTCCATGTTTATTTTTCACTTCAGCTCC

TCTTTTTTTATGTTGATGCTGAAGATTGAATAAAAACTGAGGTGGTATGTATGAGAACAAG

AACATGTTTCATTCATCTCTGTTATTTGAACATCTAAATTTTAAACAACTATCGCGACAAT

TTTTTAAGTGGTTGAAGTATTTTAAATAGGTGGTTAATTACATTAAGTAGATAGTTAATTG

ATATATCAGGTGTCAAAACTCATTAACCATCAACATAACTTCATTAGCCATAAATTTTTGA

CGTGATTAACCAAATAGTGTAGATGCTCAATTACATGTAGATTTGATGGAGTTGTCCACAA

TTTCTCATATATATATTAACTAATAACCATCTACCGAATATAATTAACCATATATTAGAAT

ATCATTAACCTCTAAAACAGTTGTCGTGAAAATTGATTGTGAAAAAATCATTTCTATTTTT

TACGCCACTAGTTCTCTTGTTATTTCTGTTTGTTGGAAAGTTGTTGTTTTTAACTTTGAGC

TTAGTCTTATTAATTAACTTGTAATGGTAACAACATTTTTCTATCTTTGTTTGAAGGAAGT

AGTTACTGTGTCAACTTTAGTAATTAATTTGGATCATTTTGGAGGAAAAATATGGCTATGC

AACCTGTTTATTTTAAAGAACATGAAGGAAATGTCCACAATTCTGTTGGACAGTTTTCATC

TGTGACTTCAGCACCATGGTGGAGTAATGCCTATGGATCTCAACCTTTTTATGGAGGAGAC

TCTTGTGGCCAAATGAAACCTTTTTCGCTAGAGCTTTCCAACTACATAGACCAACTTGCTC

CGAGTAAGAACTTAGTTCGAGGAGTTGAACAATTGTTTGATAAAGGGCATACAAACCAATT

CACCATCTTTCCAGGTACTTGTTATTCAATATAATTCCGGTTTTGAATGAATTGATTTTTG

TTAAGTTGGTTCTGGATAAACGTGCATTAAATATAATATGATTTATGTTTGGATACGTTTA

TGTTAAAGTGAGTTGAACATAAATTGGAGACTAAATATCAATTGCAGAGGCAAAAGCTTCA

AATTCTAATTTCAAGTTAGAATCAAATCAATTCTACTCGTAAGCCTATAAATGTTTCAGAA

CCAATTCTACATGTCTAGAACTAAATTTGCATTCAAACATTTTCTGCTACAGATGATTGTA

AGATGTCAGTTGATACACCAAATCATCAGGCAACCTTATCCCTGCAATCACCATTTGCTGC

CGAGCCACTTAATCGATTTGAGCTAGGTTTTAACCAGTCTGTGGTAAATCTTCTTATTAAA

GCAATCCCTTTGATCATGTTTTAGTATTTTACTTTTGATGTACATAAATTCATTATGTTTT

TATTATCCTTTCCATGCTAGATCTGTGCAAAATATCCGTATATAGATCAATTTTACGGGCT

CTTCTCGACTTATGGACCTCAAATCTCGGTTTGTCTTCACCTCGGCTATTTTATTAATCTT

TGTATATATCATGCAATGTCTATAAGTACTTGAATGTGAATTTGTTTTATTTACAGTGTGT

ATGCTTACATGAGAAAGCCAGAAATCTGTTTACAACTATCTTGTTAGTATTCTTCTAAATC

TTTCAAATGTAGAGTTGATTATGAAATTTGTTGTTAATGAGTTTTCGGCTTTATGAATGAA

TTGTAAGACAAGTAATGAAACAAAAACTAGAAGAAAATTGAATTATATGATGCAATCTGTG

TTGAAGTTTTTGCATGTATTCAAGTTCTGCTTTTAGTTACTATTTGTTTATCTGCTGCAAT

ATTTCATCAATTATTATTCATGTATGTACCAATATTTAATAAATTAGGGGCGAATTATGCT

TCCGCTTAGCATGACATCTGACGACGGACCAACATATGTGAATGCTAAGCAATACCATGGA

ATCATCAGACGCAGGCATTCTCGTGCCAAAGCTGTGCTTCAGAATAAATTGATAAAGCGTA

ACAAGGTAGGAAACTGAGTATTTTTCCTCACAGCAATGTTTGAAAACCTGTGAAATAGTAT

ATTTTTCCTTTTACCCTTTTTATACCTATATTAAAGTTTTGTATTTGTCTTGCAGCCATAT

ATGCACGAATCGCGTCATCTACATGCAATGCGTAGACCAAGAGGATGCGGTGGTCGTTTCT

TGAACACAAAAGTTTCTGCTAATGGAAACGGTAAAAGTGGGAGTGAAGAGAACGGAAACAT

TGGTGGCCTACAGCTGCAGTCCAGTGGTTCTCAGAGTTCCGAAGTCTTACAATCTGAGGTT

GGAACTTTAAATTCGTCGAAGGAGACAAACGGAGGCAGTCCAAATGTCTCGGGGTCAGAGG

TGACTAGCATGTATACACAGGGAGGTCTTGATAGCTTTACTGTCAACCATATCGGATCTAC

TGTCCACTCTTTGGGAGACATGATCGATACTGGACACGGTATCGTCATGCCAACAAAATGG

TTTGCAGCAGCTGGCAGACAGCTGCTGGAACCTTAAGTTTCGATTCAGAAAGGAAACAAGT

GGGTTTGGTACAATGTGAAACATTTTGCACCAAACTCATCCTTTCCGAGACCAGATGAAGA

AGCTATGTTTCAGTTTGTTGTGTTTACTACGACAAATTTAGTTTTGGAAGCCTGCTTTTCC

ATCTGGTGCTCAGGCAACTCATTCTTGGCTTATTCTCAGGAAACTCATCCTTGGCTCGTAA

TATTTAGTAGTATTGTCATCGTCTTCCTACGCAGGCTTTCCGTGGCATGGTAGGCATGCTA

ATGACTTTGGTATTTTCATGCAGTTATAACTATGATGTGTCTTTGTTTGTTGTTAAAATAA

AAAACATGAACTCTAGCTAGGTGCATGTGTGTGTTTTTAATCTTGTCTAGTAAGTTTGGTG

TTTTGTAATGGATTTCTGACTTTATGGAGCAATGTATTGTAACTCTACTAAGAAGTGTACC

ATTTTATATCTCCCTCTCTAAGGATTGTATAAGAACCTCTTATTTTCAGACTCTACTTAAT

CCTATTTTCTATGTCTGTATGATTTTTATATTTCTAGGACAATCAAATTGGCTTGTAGAAG

CCCAAAAGCATGCTCACAAAGTAGGTACTTATGTAGGGAACTTCGTACTCTAATAATAACT

GGTTATTAACGTTATAATTAAATGCAAAATTTGATAAGTAGTAGGGTTTCGTAAGTAATAT

AACAACACCATATTTGCTTGGGATTCTGAATTGTGTTATTAGAGTACAACTTTTAAATAAT

GATCCTAATGTAAATAACTTAAATTTGAAGTTACTAGATAAAAAGATAATATTATGTAATA

TATTATCAAATATGTACTAAAAATGTAATTTTCATCTATCTCAATTGAAAAAATATATGAA

TTTTGAGTCTATTGTCATGGACGG

>HM003

AAAAAAATAAAGGTTCTATACTCATTAAACAAAGTGCCAAATTCAGAGATACTACTTCCT

TTGGATAATTTGTATCGTCACCAACGATCTTTGAGTTATTTCAAAAATCACATTCTCAATT

TTGTGGTCCAACATTCACAAAATGGTTTGTCGAGCTTTAAGTCTAGTAATTGTGAAACATG

CATATGCATGAGGTAATTAATTGATAATCTGAAAAGGTTAAATAGTTAATCAATTGTACTT

TCGTTCTTTTTAATTTGTTCTCTATAATTTTTCAGTCAAAATTGAATTTGCTTTAACCATT

TAGTGTAGTAGTATGATATTACTTGCTCTACATTCTTGGATCTAAAGGTCTAATTCGTATC

GTATAAAAAATATTCTAGTTAATTAAGATACTTTTGTTCATTCTAATTATTCTTGGATTAC

TTGTGAAACTTTTAGGGATGAACAAGAAAGTCCACTAAAGAGGCTTCTTTTTGCTAGGAGT

CACTAGTTAGTTTTTTTTTTTTTTGACAAAAGTTAACCATTTTTTTTTAACGCATGGTAGG

AAAAAAAAAAAAGAGATTTCCTTTGGTACTCATAAAAAAAAAAATTGGATGGATAATGGAT

TAATCATAACTTATTTAACATAAAATGAACTAAGAGAACATTTCATATTTGGGTTTATTTT

GTAACATAAATATTAATATCAATGTTTGAAAGAGTTTTTAACAATAATTTGTAAAAGAAAG

CATAATATATCTATCAAAATAATTATAAAAGAAAAAATTTCTTTTGGTGGTATGACATGGA

AATATAATTAATTCTTATTAGATGAAAGTGTAAAATTAATTTGCATTAACAAAGCATATTT

CATTAAACTCCATTTAGTTTTTGTTGGAAAAAATAAATTTATTGTTACTCAAAATATAAAA

GTGTACAATTAATATATTTACTCAAAAGTCCCCTAAATGGGATGTAATTATCAAGGGATGT

GAGCGGTTTAACTTTAAGTAATGAATCAACTGTTGCATAATCACTTTGAAAGAAACTTGAG

ATTCTTCAATTAGCAAAATCTTGAATAGAATAATGAGATTATTCCTAATGCTAATGATACC

CACCTCTCCTATAAGTTGATGGGGCAGAACAAATTATGCACTCAGGACAACTTTTCTCAAA

TTTTGAAAACTTACCACACGAAGAACACACTTCCACTTGCACAAGTAAGAATAGAGTATAA

CAATAAATTTACCACTCATAGCTCAATGGATATTAAAATATTGCTAATCTTTTATAGACAG

ATGTTGGCAAATTAGTATAAATTAGTTATTATATTAGTTTTTAAGGTTCAGTATACTTTTT

AAAACTCTTATTAAGTTGTTTAGCTTGCCAACCAAAGACTAAATTATCTTTATTTGTTAGG

AAATAAAAGCACTTATTTTATTTTCTCCATATTTGTTTCCTCTTTGTTCCATTACTAAAAA

AATTATAATATATTAATAACAAAAAAATTTGGGGAGGGAATGTTATTTTTAAACAAGATGT

TTTATTTATATTTAAGTGTTTTATTGAAATTCCTCGTGAGTTTAGCTCAATTGGTATGGAC

AATACATAATATATGCAAAATTCGAGGTTCAAACCTTGAGCACCACAAAAAAATGTGTTTT

ATTGAAAAATATCAAGAACTTGAAAACAAATTTCCTTTTTATGAACTAAATATTGTCACAT

AGACAACTTAAGAATTGACTTTTTTTTTAACACTATTGTATTTTCTGAACTCGACTTTAAC

TCGAGATCAATGAGTAAACTAAAAAAACTCGTATTATTTTATCCAAGTGTTCTTAGTGTGA

GAAAACATTATAATTGTTTGCTTAGTCTATTTCTCCCTATATTCTCACCCTCATTCTTGGT

TTGCACATAGAAAGGAGAAAAAAAAAATTAGAGAAGAGTTGAATGGAACAGAAAGAAAGAG

CAAGCAAAATAATCACTAAAAAAAATTCCCCTAAAAGAAAATCACTGAAAAATATTATCCA

CCTCTATTTTAAATAATAATAATAATGAAAACAACAATATATTTTTTTATAGATTTTCTCT

ACTAGAGGTAATTTATTTTAAACACTAAATGTCCACACCTCTAACAAAAAAAATGCATAAT

ATTATAATATATTATTGTAAGAAAGAGTGACAAATGTTGAATTAGTTATGGAATGAAGAGT

GAGAGAGATTATACAAATAAATAAATATAAATATAAATATAAATAAAGAATAGAGAAGAAC

ATAGTACTTGAATGAAGAGATTTGGCTTGTACTTCCATTACACTTGTAAGCTTTCTACTTC

CTTCACATTCCTTCTCTTTCTCTATCTTTCCTTTTATGATATGTGTACTTTGTTATTGCTT

CATTTACTACCGACATGACTCCAACCTATTAGGGCTTTATCTGAATAACGTTAGTTATTTT

CTCTCATTTTCTTTGCTTCTTCTTATTCTTGGAACTTATAAAGGGTCTTGTCAATTCTATC

GTTCTCTTTTGTATACTTACAAATATAAATATTCTCTTTAGCTTCATTTTCATGTCTTCTT

CTACTAATAAGCTATGGAAATTGAAGTATTTGTTTTCATGTTTATTTTTCACTTCAGCTCC

TCTTTTTTGATGTTGATGCTGAAGATTGAATAAAAACTGAGGTGGTATGTATGAGAACAAG

AACATGTTTCATTCATCTCTGTTATTTGAACATCTAAATTTTAAACAACTATCGCGACAAT

TTTTTAAGTGGTTGAAGTATTTTAAATAGGTGGTTAATTACATTAAGTAGATAGTTAATTG

ATATATCAGGTGTCAAAACTCATTAACCATCAACATAACTTCATTAGCCATAAATTTTTGA

CGTGATTAACCAAATATTGTAGATGCTCAATTACATGTAGATTTGATGGAGTTGTCCACAA

TTTCTCATATATATATTAACTAATAACCATCTACCGAATATAATTAACCATATATTAGAAT

ATCATTAACCTCTAAAACAGTTGTCGCGAAAATTGATTGTAAAAAAATCATTTCTATTTTT

TACGCCACTAGTTCTCTTGTTATTTCTCTTTGTTGGAAAGTTGTTGTTTTTAACTTTGAGC

TTAGTCTTATTAATTAACTTGTAATGGTAACAACATTTTTCTATCTTTGTTTGAAGGAAGT

AGTTACTGTGTCAACTTTAGTAATTAATTTGGATCATTTTGGAGGAAAAATATGGCTATGC

AACCTGTTTATTTTAAAGAACATGAAGGAAATGTCCACAATTCTGTTGGACAGTTTTCATC

TGTGACTTCAGCACCATGGTGGAGTAATGCCTATGGATCTCAACCTTTTTATGGAGGAGAC

TCTTGTGGCCAAATGAAACCTTTTTCGCTAGAGCTTTCCAACTACATAGACCAACTTGCTC

CGAGTAAGAACTTAGTTCGAGGAGTTGAACAATTGTTTGATAAAGGGCATACAAACCAATT

CACCATCTTTCCAGGTACTTGTTATTCAATATAATTCCGGTTTTGAATGAATTGATTTTTG

TTAAGTTGGTTCTGGATAAACGTGCATTAAATATAATATGATTTATGTTTGGATACGTTTA

TGTTAAAGTGAGTTGAACATAAATTGGAGACTAAATATCAATTGCAGAGGCAAAAGCTTCA

AATTCTAATTTCAAGTTAGAATCAAATCAATTCTACTCGTAAGCATATAAATGTTTCAGAA

CCAATTCTACATCTCTAGAACTAAATTTGCATTCAAACATTTTCTGCTACAGATGATTGTA

AGATGTCAGTTGATACACCAAATCATCAGGCAACCTTATCCCTGCAATCACCATTTGCTGC

CGAGCCACTTAATCGATTTGAGCTAGGTTTTAACCAGTCTATGGTAAATCTTCTTATTAAA

GCAATCCCTTTGATCATGTTTTAGTATTTTACTTTTGATGTACATAAATTCATTATGTTTT

TATTATCCTTTCCATGCTAGATCTGTGCAAAATATCCGTATATAGATCAATTTTACGGGCT

CTTCTCGACTTATGGACCTCAAATCTCGGTTTGTCTTCACCTCGGCTATTTTATTAATCTT

TGTATATATCATGCAATGTCTATAAGTACTTGAATGTGAATTTGTTTTATTTACAGTGTGT

ATGCTTACATGAGAAAGCCAGAAATCTGTTTACAACTATCTTGTTAGTATTCTTCTAAATC

TTTCAAATGTAGAGTTGATTATGAAATTTGTTGTTAATGAGTTTTCGGCTTTATGAATGAA

TTGTAAGACAAGTAATGAAACAAAAACTAGAAGAAAATTGAATTATATGATGCAATCTGTG

TTGAAGTTTTTGCATGTATTCAAGTTCTGCTTTTAGTTACTATTTGTTTATCTGCTGCAAT

ATTTCATCAATTATTATTCATGTATGTACCAATATTTAATAAATTAGGGGCGAATTATGCT

TCCGCTTAGCATGACATCTGACGACGGACCAACATACGTGAATGCTAAGCAATACCATGGA

ATCATCAGACGCAGGCATTCTCGTGCCAAAGCTGTGCTTCAGAATAAATTGATAAAGCGTA

ACAAGGTAGGAAACTGAGTATTTTTCCTCACAGCAATGTTTGAAAACTTGTGAAATAGTAT

ATTTTTCCTTTTACCCTTTTTATACCTATATTAAAGTTTTGTATTTGTCTTGCAGCCATAT

ATGCACGAATCGCGTCATCTACATGCAATGCGTAGACCAAGAGGATGCGGTGGTCGTTTCT

TGAACACAAAAGTTTCTGCTAATGGAAACGGTAAAAGTGGGAGTGAAGAGAACGGAAACAT

TGGTGGCCTACAGCTGCAGTCCAGTGGTTCTCAGAGTTCCGAAGTCTTACAATCTGAGGTT

GGAACTTTAAATTCGTCGAAGGAGACAAACGGAGGCAGTCCAAATGTCTCGGGGTCAGAGG

TGACTAGCATGTATACACAGGGAGGTCTTGATAGCTTTACTGTCAACCATATCGGATCTAC

TGTCCACTCTTTGGGAGACATGATCGATACTGGACACGGTATCGTCATGCCAACAAAATGG

TTTGCAGCAGCTGGCAGACAGCTGCTGGAACCTTAAGTTTCGATTCAGAAAGGAAACAAGT

GGGTTTGGTACAATGTGAAATATTTTGCACCAAACTCATCCTTTCCGAGACCAGATGAAGA

AGCTATGTTTCAGTTTGTTGTGTTTACTACGACAAATTTAGTTTTGGAAGACTACTTTTCC

ATCTGGTGCTCAGGCAACTCATTCTTGGCTTATTCTCAGGAAACTCATCCTTGGCTCGTAA

TATTTAGTAGTATTGTCATCGTCTTCCTACGCAGGCTTTCCGTGGCATGGTAGGCATGCTA

ATGACTTTGGTATTTTCATGCAGTTATAACTATGATGTGTCTTTGTTTGTTGTTAAAATAA

AAAACATGAACTCTAGCTAGGTGCATGTGTGTGTTTTTAATCTTGTCTACTAAGTTTGGTG

TTTTGTAATGGATTTCTGACTTTATGGAGCAATGTATTGTAACTCTACTAAGAAGTGTACC

ATTTTATTTCTCCCTCTCTAAGGATTGTATAAGAACCTCTTATTTTCAGACTCTACTTAAT

CCTATTTTCTATGTCTGTATGATTTTTATATTTCTAGGACAATCAAATTGGCTTGTAGAAG

CTCAAAAGCATGCTCACAAAGTAGGTACTTATGTAGGGAACTTCGTACTCTAATAATAACT

GGTTATTAACGTTATAATTAAATGCAAAATTTGATAAGTAGTAGGGTTTCGTAAGTAATAT

AACAACACCATATTGGCTTGGGATTCTGAATTGTGTTATTAGAGTACAACTTTTAAATAAT

GATCCTAATGTAAATAACTTAAATTTGAAGTTACTAGATAAAAAGATAATATTATGTAATA

TATTATCAAATATGTACTAAAAATGTAATTTTCATCTATCTCAATTGAAAAAATATATGAA

TTTTGAGTCTATTGTCATGGACGA

>HM004

AAAAAAATAAAGGTTCTATACTCATTAAACAAAGTGCCAAATTCAGAGATACTACTTCCT

TTGGATAATTTGTATCGTCACCAACGATCTTTGAGTTATTTCAAAAATCACATTCTCAATT

TTGTGGTCCAACATTCACAAAATGGTTTGTCGAGCTTTAAGTCTAGTAATTGTGAAACATG

CATATGCATGAGGTAATTAATTGATAATCTGAAAAGGTTAAATAGTTAATCAATTGTACTT

TCGTTCTTTTTAATTTGTTCTCTATAATTTTTCAGTCAAAATTGAATTTGCTTTAACCATT

TAGTGTAGTAGTATGATATTACTTGCTCTACATTCTTGGATCTAAAGGTCTAATTAGTATC

GTATAAAAAATATTCTAGTTAATTAAGATACTTTTGTTCATTCTAATTATTCTTGGATTAC

TTGTGAAACTTTTAGGGATGAACAAGAAAGTCCACTAAAGAGGCTTCTTTTTGCTAGGAGT

CACTAGTTAGTTTTTTTTTTTTTTGACAAAAGTTAACCATTTTTTTTTATCGCATGGTAGG

AAAAAAAAAAAAGAGATTTCCTTTGGTACTCATAAAAAAAAAAGTTGGATGGATAATGGAT

TAATCATAACTTATTTAACATAAAATGAACTAAGAGAACATTTCATATTTGGGTTTATTTT

GTAACATAAATATTAATATCAATGTTTGAAAGAGTTTTTAACAATAATTTGTAAAAGAAAG

CATAATATATCTATCAAAATAATTATAAAAGAAAAAATTTCTTTTGGTGGTATGACATGGA

AATATAATTAATTCTTATTAGATGAAAGTGTAAAATTAATTTGCATTAACAAAGCATATTT

CATTAAACTCCATTTAGTTTTTGTTGGAAAAAATAAATTTATTGTTACTCAAAATATAAAA

GTGTACAATTAATATATTTACTCAAAAGTCCCCTAAATGGGATGTAATTATCAAGGGATGT

GAGCGGTTTAACTTTAAGTAATGAATCAACTGTTGCATAATCACTTTGAAAGAAACTTGAG

ATTCTTCAATTAGCAAAATCTTGAATAGAATAATGAGATTATTCCTAATGCTAATGATACC

CACCTCTCCTATAAGTTGATGGGGCAGAACAAATTATGCACTCAGGACAACTTTTCTCAAA

TTTTGAAAACTTACCACACGAAGAACACACTTCCACTTGCACAAGTAAGAATATAGTATAA

CAATAAATTTACCACTCATAGCTCAATGGATATTAAAATATTGCTAATCTTTTATAGACAG

ATGTTGGCAAATTAGTATAAATTAGTTATTATATTAGTTTTTAAGGTTCAATATACTTTTT

AAAACTCTTATTAAGTTGTTTAGCTTGCCAACCAAAGACTAAATTATCTTTATTTGTTAGG

AAATAAAAGCACTTATTTTATTTTCTCCATATTTGTTTCCTCTTTGTTCCATTACTAAAAA

AATTATAATATATTAATAACAAAAAAATTTGGGGAGGGAATGTTATTTTTAAACAAGATGT

TTTATTTATATTTAAGTGTTTTATTGAAATTCCTCGTGAGTTTAGCTCAATTGGTATGGAC

AATACATAATATATGCAAAATTCGAGGTTCAAACCTTGAGCACCACAAAAAAATGTGTTTT

ATTGAAAAATATCAAGAACTTGAAAACAAATTTCCTTTTTATGAACTAAATATTGTCACAT

AGACAACTTAAGAATTGACTTTTTTTTTAACACTATTGTATTTTCTGAACTCGACTTTAAC

TCGAGATCAATGAGTAAACTAAAAAAACTCGTATTATTTTATCTAAGTGTTCTTAGTGTGA

GAAAACATTATAATTGTTTGCTTAGTCTATTTCTCCCTATATTCTCACCCTCATTCTTGGT

TTGCACATAGAAAGGAGAAAAAAAAAATTAGAGAAGAGTTGAATGGAACAGAAAGAAAGAG

CAAGCAAAATAATCACTAAAAAAAATTCCCCTAAAAGAAAATCACTGAAAAATATTATCCA

CCTCTATTTTAAATAATAATAATAATGAAAACAACAATATATTTTTTTATAGATTTTCTCT

ACTAGAGGTAATTTATTTTAAACACTAAATGTCCACACCTCTAACAAAAAAAATGCATAAT

ATTATAATATATTATTGTAAGAAAGAGTGACAAATGTTGAATTAGTTATGGAATGAAGAGT

GAGAGAGATTATACAAATAAATAAATATAAATATAAATATAAATAAAGAATAGAGAAGAAC

ATAGTACTTGAATGAAGAGATTTGGCTTGTACTTCCATTACACTTGTAAGCTTTCTACTTC

CTTCACATTCCTTCTCTTTCTCTATCTTTCCTTTTATGATATGTGTACTTTGTTATTGCTT

CATTTACTACCGACATGACTCCAACCTATTAGGGCTTTATCTGAATAATGTTAGTTATTTT

CTCTCATTTTCTTTGCTTCTTCTTATTCTTGGAACTTATAAAGGGTCTTGTCAATTCTATC

GTTCTCTTTTGTATACTTACAAATATAAATATTCTCTTTAGCTTCATTTTCATGTCTTCTT

CTACTAATAAGCTATGGAAATTGAAGTATTTGTTTTCATGTTTATTTTTCACTTCAGCTCC

TCTTTTTTGATGTTGATGCTGAAGATTGAATAAAAACTGAGGTGGTATGTATGAGAACAAG

AACATGTTTCATTCATCTCTGTTATTTGAACATCTAAATTTTAAACAACTATCGCGACAAT

TTTTTAAGTGGTTGAAGTATTTTAAATAGGTGGTTAATTACATTAAGTAGATAGTTAATTG

ATATATCAGGTGTCAAAACTCATTAACCATCAACATAACTTCATTAGCCATAAATTTTTGA

CGTGATTAACCAAATATTGTAGATGCTCAATTACATGTAGATTTGATGGAGTTGTCCACAA

TTTCTCATATATATATTAACTAATAACCATCTACCGAATATAATTAACCATATATTAGAAT

ATCATTAACCTCTAAAACAGTTGTCGCGAAAATTGATTGTAAAAAAATCATTTCTATTTTT

TACGCCACTAGTTCTCTTGTTATTTCTCTTTGTTGGAAAGTTGTTGTTTTTAACTTTGAGC

TTAGTCTTATTAATTAACTTGTAATGGTAACAACATTTTTCTATCTTTGTTTGAAGGAAGT

AGTTACTGTGTCAACTTTAGTAATTAATTTGGATCATTTTGGAGGCAAAATATGGCTATGC

AACCTGTTTATTTTAAAGAACATGAAGGAAATGTCCACAATTCTGTTGGACAGTTTTCATC

TGTGACTTCAGCACCATGGTGGAGTAATGCCTATGGATCTCAACCTTTTTATGGAGGAGAC

TCTTGTGGCCAAATGAAACCTTTTTCGCTAGAGCTTTCCAACTACATAGACCAACTTGCTC

CGAGTAAGAACTTAGTTCGAGGAGTTGAACAATTGTTTGATAAAGGGCATACAAACCAATT

CACCATCTTTCCAGGTACTTGTTATTCAATATAATTCCGGTTTTGAATGAATTGATTTTTG

TTAAGTTGGTTCTGGATAAACGTGCATTAAATATAATATGATTTATGTTTGGATACGTTTA

TGTTAAAGTGAGTTGAACATAAATTGGAGACTAAATATCAATTGCAGAGGCAAAAGCTTCA

AATTCTAATTTCAAGTTAGAATCAAATCAATTCTACTCGTAAGCATATAAATGTTTCAAAA

CCAATTCTACATCTCTAGAACTAAATTTGCATTCAAACATTTTCTGCTACAGATGATTGTA

AGATGTCAGTTGATACACCAAATCATCAGGCAACCTTATCCCTGCAATCACCATTTGCTGC

CGAGCCACTTAATCGATTTGAGCTAGGTTTTAACCAGTCTATGGTAAATCTTCTTATTAAA

GCAATCCCTTTGATCATGTTTTAGTATTTTACTTTTGATGTACATAAATTCATTATGTTTT

TATTATCCTTTCCATGCTAGATCTGTGCAAAATATCCGTATATAGATCAATTTTACGGGCT

CTTCTCGACTTATGGACCTCAAATCTCGGTTTGTCTTCACCTCGGCTATTTTATTAATCTT

TGTATATATCATGCAATGTCTATAAGTACTTGAATGTGAATTTGTTTTATTTACAGTGTGT

ATGCTTACATGAGAAAGCCAGAAATCTGTTTACAACTATCTTGTTAGTATTCTTCTAAATC

TTTCAAATGTAGAGTTGATTATGAAATTTGTTGTTAATGAGTTTTCGGCTTTATGAATGAA

TTGTAAGACAAGTAATGAAACAAAAACTAGAAGAAAATTGAATTATATGATGCAATCTGTG

TTGAAGTTTTTGCATGTATTCAAGTTCTGCTTTTAGTTACTATTTGTTTATCTGCTGCAAT

ATTTCATCAATTATTATTCATGTATGTACCAATATTTAATAAATTAGGGGCGAATTATGCT

TCCGCTTAGCATGACATCTGACGACGGACCAACATACGTGAATGCTAAGCAATACCATGGA

ATCATCAGACGCAGGCATTCTCGTGCCAAAGCTGTGCTTCAGAATAAATTGATAAAGCGTA

ACAAGGTAGGAAACTGAGTATTTTTCCTCACAGCAATGTTTGAAAACTTGTGAAATAGTAT

ATTTTTCCTTTTACCCTTTTTATACCTATATTAAAGTTTTGTATTTGTCTTGCAGCCATAT

ATGCACGAATCGCGTCATCTACATGCAATGCGTAGACCAAGAGGATGCGGTGGTCGTTTCT

TGAACACAAAAGTTTCTGCTAATGGAAACGGTAAAAGTGGGAGTGAAGAGAACGGAAACAT

TGGTGGCCTACAGCTGCAGTCCAGTGGTTCTCAGAGTTCCGAAGTCTTACAATCTGAGGTT

GGAACTTTAAATTCGTCGAAGGAGACAAACGGAGGCAGTCCAAATGTCTCGGGGTCAGAGG

TGACTAGCATGTATACACAGGGAGGTCTTGATAGCTTTACTGTCAACCATATCGGATCTAC

TGTCCACTCTTTGGGAGACATGATCGATACTGGACACGGTATCGTCATGCCAACAAAATGG

TTTGCAGCAGCTGGCAGACAGCTGCTGGAACCATAAGTTTCGATTCAGAAAGGAAACAAGT

GGGTTTGGTACAATGTGAAATATTTTGCACCAAACTCATCCTTTCCGAGACCAGATGAAGA

AGCTATGTTTCAGTTTGTTGTGTTTACTACGACAAATTTAGTTTTGGAAGACTACTTTTCC

ATCTGGTGCTCAGGCAACTCATTCTTGGCTTATTCTCAGGAAACTCATCCTTGGCTCGTAA

TATTTAGTAGTATTGTCATCGTCTTCCCACGCAGGCTTTCCGTGGCATGGTAGGCATGCTA

ATGACTTTGGTATTTTCATGCAGTTATAACTATGATGTGTCTTTGTTTGTTGTTAAAATAA

AAAACATGAACTCTAGCTAGGTGCATGTGTGTGTTTTTAATCTTGTCTACTAAGTTTGGTG

TTTTGTAATGGATTTCTGACTTTATGGAGCAATGTATTGTAACTCTACTAAGAAGTGTAAC

ATTTTATTTCTCCCTCTCTAAGGATTGTATAAGAACCTCTTATTTTCAGACTCTACTTAAT

CCTATTTTCTATGTCTGTATGATTTTTATATTTCTAGGACAATCAAATTGGCTTGTAGAAG

CTCAAAAGCATGCTCACAAAGTAGGTACTTATGTAGGGAACTTCGTACTCTAATAATAACT

GGTTATTAACGTTATAATTAAATGCAAAATTTGATAAGTAGTAGGGTTTCGTAAGTAATAT

AACAACACCATATTGGCTTGGGATTCTGAATTGTGTTATTAGAGTACAACTTTTAAATAAT

GATCCTAATGTAAATAACTTAAATTTGAAGTTACTAGATAAAAAGATAATATTATGTAATA

TATTATCAAATATGTACTAAAAATGTAATTTTCATCTATCTCAATTGAAAAAATATATGAG

TTTTGAGTCTATTGTCATGGACGA

>HM005

AAAAAAATAAAGGTTCTATACTCATTAAACAAAGTGCCAAATTCAGAGATACTACTTCCT

TTGGATAATTTGTATCGTCACCAACGATCTTTGAGTTATTTCAAAAATCACATTCTCAATT

TTGTGGTCCAACATTCACAAAATGGTTTGTCGAGCTTTAAGTCTAGTAATTGTGAAACATG

CATATGCATGAGGTAATTAATTGATAATCTGAAAAGGTTAAATAGTTAATCAATTGTACTT

TGGTTCTTTTTAATTTGTTCTCTATAATTTTTCAGTCAAAATTGAATTTGCTTTAACCATT

TAGTGTAGTAGTATGATATTACTTGCTCTACATTCTTGGATCTAAAGGTCTAATTAGTATC

GTATAAAAAATATTCTAGTTAATTAAGATACTTTTGTTCATTCTAATTATTCTTGGATTAC

TTGTGAAACTTTTAGGGATGAACAAGAAAGTCCACTAAAGAGGCTTCTTTTTGCTAGGAGT

CACTAGTTAGTTTTTTTTTTTTTTGACAAAAGTTAACCATTTTTTTTTATCGCATGGTAGG

AAAAAAAAAAAAGAGATTTCCTTTGGTACTCATAAAAAAAAAAGTTGGATGGATAATGGAT

TAATCATAACTTATTTAACATAAAATGAACTAAGAGAACATTTCATATTTGGGTTTATTTT

GTAACATAAATATTAATATCAATGTTTGAAAGAGTTTTTAACAATAATTTGTAAAAGAAAG

CATAATATATCTATCAAAATAATTATAAAAGAAAAAATTTCTTTTGGTGGTATGACATGGA

AATATAATTAATTCTTATTAGATGAAAGTGTAAAATTAATTTGCATTAACAAAGCATATTT

CATTAAACTCCATTTAGTTTTTGTTGGAAAAAATAAATTTATTGTTACTCAAAATATAAAA

GTGTACAATTAATATATTTACTCAAAAGTCCCCTAAATGGGATGTAATTATCAAGGGATGT

GAGCGGTTTAACTTTAAGTAATGAATCAACTGTTGCATAATCACTTTGAAAGAAACTTGAG

ATTCTTCAATTAGCAAAATCTTGAATAGAATAATGAGATTATTCCTAATGCTAATGATACC

CACCTCTCCTATAAGTTGATGGGGCAGAACAAATTATGCACTCAGGACAACTTTTCTCAAA

TTTTGAAAACTTACCACACGAAGAACACACTTCCACTTGCACAAGTAAGAATATAGTATAA

CAATAAATTTACCACTCATAGCTCAATGGATATTAAAATATTGCTAATCTTTTATAGACAG

ATGTTGGCAAATTAGTATAAATTAGTTATTATATTAGTTTTTAAGGTTCAATATACTTTTT

AAAACTCTTATTAAGTTGTTTAGCTTGCCAACCAAAGACTAAATTATCTTTATTTGTTAGG

AAATAAAAGCACTTATTTTATTTTCTCCATATTTGTTTCCTCTTTGTTCCATTACTAAAAA

AATTATAATATATTAATAACAAAAAAATTTGGGGAGGGAATGTTATTTTTAAAAAAGATGT

TTTATTTATATTTAAGTGTTTTATTGAAATTCCTCGTGAGTTTAGCTCAATTGGTATGGAC

AATACATAATATATGCAAAATTCGAGGTTCAAACCTTGAGCACCACAAAAAAAAGTGTTTT

ATTGAAAAATATCAAGAACTTGAAAACAAATTTCCTTTTTATGAACTAAATATTGTCACAT

AGACAACTTAAGAATTGACTTTTTTTTTAACACTATTGTATTTTCTGAACTCGACTTTAAC

TCGAGATCAATGAGTAAACTAAAAAAACTCGTATTATTTTATCTAAGTGTTCTTAGTGTGA

GAAAACATTATAATTGTTTGCTTAGTCTATTTCTCCCTATATTCTCACCCTCATTCTTGGT

TTGCACATAGAAAGGAGAAAAAAAAAATTAGAGAAGAGTTGAATGGAACAGAAAGAAAGAG

CAAGCAAAATAATCACTAAAAAAAATTCCCCTAAAAGAAAATCACTGAAAAATATTATCCA

CCTCTATTTTAAATAATAATAATAATGAAAACAACAATATATTTTTTTATAGATTTTCTCT

ACTAGAGGTAATTTATTTTAAACACTAAATGTCCACACCTCTAACAAAAAAAATGCATAAT

ATTATAATATATTATTGTAAGAAAGAGTGACAAATGTTGAATTAGTTATGGAATGAAGAGT

GAGAGAGATTATACAAATAAATAAATATAAATATAAATATAAATAAAGAATAGAGAAGAAC

ATAGTACTTGAATGAAGAGATTTGGCTTGTACTTCCATTACACTTGTAAGCTTTCTACTTC

CTTCACATTCCTTCTCTTTCTCTATCTTTCCTTTTATGATATGTGTACTTTGTTATTGCTT

CATTTACTACCGACATGACTCCAACCTATTAGGGCTTTATCTGAATAATGTTAGTTATTTT

CTCTCATTTTCTTTGCTTCTTCTTATTCTTGGAACTTATAAAGGGTCTTGTCAATTCTATC

GTTCTCTTTTGTATACTTACAAATATAAATATTCTCTTTAGCTTCATTTTCATGTCTTCTT

CTACTAATAAGCTATGGAAATTGAAGTATTTGTTTTCATGTTTATTTTTCACTTCAGCTCC

TCTTTTTTGATGTTGATGCTGAAGATTGAATAAAAACTGAGGTGGTATGTATGAGAACAAG

AACATGTTTCATTCATCTCTGTTATTTGAACATCTAAATTTTAAACAACTATCGCGACAAT

TTTTTAAGTGGTTGAAGTATTTTAAATAGGTGGTTAATTACATTAAGTAGATAGTTAATTG

ATATATCAGGTGTCAAAACTCATTAACCATCAACATAACTTCATTAGCCATAAATTTTTGA

CGTGATTAACCAAATATTGTAGATGCTCAATTACATGTAGATTTGATGGAGTTGTCCACAA

TTTCTCATATATATATTAACTAATAACCATCTACCGAATATAATTAACCATATATTAGAAT

ATCATTAACCTCTAAAACAGTTGTCGCGAAAATTGATTGTAAAAAAATCATTTCTATTTTT

TACGCCACTAGTTCTCTTGTTATTTCTCTTTGTTGGAAAGTTGTTGTTTTTAACTTTGAGC

TTAGTCTTATTAATTAACTTGTAATGGTAACAACATTTTTCTATCTTTGTTTGAAGGAAGT

AGTTACTGTGTCAACTTTAGTAATTAATTTGGATCATTTTGGAGGCAAAATATGGCTATGC

AACCTGTTTATTTTAAAGAACATGAAGGAAATGTCCACAATTCTGTTGGACAGTTTTCATC

TGTGACTTCAGCACCATGGTGGAGTAATGCCTATGGATCTCAACCTTTTTATGGAGGAGAC

TCTTGTGGCCAAATGAAACCTTTTTCGCTAGAGCTTTCCAACTACATAGACCAACTTGCTC

CGAGTAAGAACTTAGTTCGAGGAGTTGAACAATTGTTTGATAAAGGGCATACAAACCAATT

CACCATCTTTCCAGGTACTTGTTATTCAATATAATTCCGGTTTTGAATGAATTGATTTTTG

TTAAGTTGGTTCTGGATAAACGTGCATTAAATATAATATGATTTATGTTTGGATACGTTTA

TGTTAAAGTGAGTTGAACATAAATTGGAGACTAAATATCAATTGCAGAGGCAAAAGCTTCA

AATTCTAATTTCAAGTTAGAATCAAATCAATTCTACTCGTAAGCATATAAATGTTTCAAAA

CCAATTCTACATCTCTAGAACTAAATTTGCATTCAAACATTTTCTGCTACAGATGATTGTA

AGATGTCAGTTGATACACCAAATCATCAGGCAACCTTATCCCTGCAATCACCATTTGCTGC

CGAGCCACTTAATCGATTTGAGCTAGGTTTTAACCAGTCTATGGTAAATCTTCTTATTAAA

GCAATCCCTTTGATCATGTTTTAGTATTTTACTTTTGATGTACATAAATTCATTATGTTTT

TATTATCCTTTCCATGCTAGATCTGTGCAAAATATCCGTATATAGATCAATTTTACGGGCT

CTTCTCGACTTATGGACCTCAAATCTCGGTTTGTCTTCACCTCGGCTATTTTATTAATCTT

TGTATATATCATGCAATGTCTATAAGTACTTGAATGTGAATTTGTTTTATTTACAGTGTGT

ATGCTTACATGAGAAAGCCAGAAATCTGTTTACAACTATCTTGTTAGTATTCTTCTAAATC

TTTCAAATGTAGAGTTGATTATGAAATTTGTTGTTAATGAGTTTTCGGCTTTATGAATGAA

TTGTAAGACAAGTAATGAAACAAAAACTAGAAGAAAATTGAATTATATGATGCAATCTGTG

TTGAAGTTTTTGCATGTATTCAAGTTCTGCTTTTAGTTACTATTTGTTTATCTGCTGCAAT

ATTTCATCAATTATTATTCATGTATGTACCAATATTTAATAAATTAGGGGCGAATTATGCT

TCCGCTTAGCATGACATCTGACGACGGACCAACATACGTGAATGCTAAGCAATACCATGGA

ATCATCAGACGCAGGCATTCTCGTGCCAAAGCTGTGCTTCAGAATAAATTGATAAAGCGTA

ACAAGGTATGAAACTGAGTATTTTTCCTCACAACAATGTTCGAAAACTTGTGAAATAGTAT

ATTTTTCCTTTTACCCTTTTTATACCTATATTAAAGTTTTGTATTTGTCTTGCAGCCATAT

ATGCACGAATCGCGTCATCTACATGCAATGCGTAGACCAAGAGGATGCGGTGGTCGTTTCT

TGAACACAAAAGTTTCTGCTAATGGAAACGGTAAAAGTGGGAGTGAAGAGAACGGAAACAT

TGGTGGCCTACAGCTGCAGTCCAGTGGTTCTCAGAGTTCCGAAGTCTTACAATCTGAGGTT

GGAACTTTAAATTCGTCGAAGGAGACAAACGGAGGCAGTCCAAATGTCTCGGGGTCAGAGG

TGACTAGCATGTATACACAGGGAGGTCTTGATAGCTTTACTGTCAATCATATCGGATCTAC

TGTCCACTCTTTGGGAGACATGATCGATACTGGACACGGTATCGTCATGCCAACAAAATGG

TTTGCAGCAGCTGGCAGACAGCTGCTGGAACCATAAGTTTCGATTCAGAAAGGAAACAAGT

GGGTTTGGTACAATGTGAAATATTTTGCACCAAACTCATCCTTTCCGAGACCAGATGAAGA

AGCTATGTTTCAGTTTGTTGTGTTTACTACGACAAATTTAGTTTTGGAAGACTACTTTTCC

ATCTGGTGCTCAGGCAACTCATTCTTGGCTTATTCTCAGGAAACTCATCCTTGGCTCGTAA

TATTTAGTAGTATTGTCATCGTCTTCCCACGCAGGCTTTCCGTGGCATGGTAGGCATGCTA

ATGACTTTGGTATTTTCATGCAGTTATAACTATGATGTGTCTTTGTTTGTTGTTAAAATAA

AAAACATGAACTCTAGCTAGGTGCATGTGTGTGTTTTTAATCTTGTCTACTAAGTTTGGTG

TTTTGTAATGGATTTCTGACTTTATGGAGCAATGTATTGTAACTCTACTAAGAAGTGTAAC

ATTTTATTTCTCCCTCTCTAAGGATTGTATAAGAACCTCTTATTTTCAGACTCTACTTAAT

CCTATTTTCTATGTCTGTATGATTTTTATATTTCTAGGACAATCAAATTGGCTTGTAGAAG

CTCAAAAGCATGCTCACAAAGTAGGTACTTATGTAGGGAACTTCGTACTCTAATAATAACT

GGTTATTAACGTTATAATTAAATGCAAAATTTGATAAGTAGTAGGGTTTCGTAAGTAATAT

AACAACACCATATTGGCTTGGGATTCTGAATTGTGTTATTAGAGTACAACTTTTAAATAAT

GATCCTAATGTAAATAACTTAAATTTGAAGTTACTAGATAAAAAGATAATATTATGTAATA

TATTATCAAATATGTACTAAAAATGTAATTTTCATCTATCTCAATTGAAAAAATATATGAG

TTTTGAGTCTATTGTCATGGACGA

>HM007

AAAAAAATAAAGGTTCTATACTCATTAAACAAAGTGCCAAATTCAGAGATACTACTTCCT

TTGGATAATTTGTATCGTCACCAACGATCTTTGAGTTATTTCAAAAATCACATTCTCAATT

TTGTGGTCCAACATTCACAAAATGGTTTGTCGAGCTTTAAGTCTAGTAATTGTGAAACATG

CATATGCATGAGGTAATTAATTGATAATCTGAAAAGGTTAAATAGTTAATCAATTGTACTT

TGGTTCTTTTTAATTTGTTCTCTATAATTTTTCAGTCAAAATTGAATTTGCTTTAACCATT

TAGTGTAGTAGTATGATATTACTTGCTCTACATTCTTGGATCTAAAGGTCTAATTAGTATC

GTATAAAAAATATTCTAGTTAATTAAGATACTTTTGTTCATTCTAATTATTCTTGGATTAC

TTGTGAAACTTTTAGGGATGAACAAGAAAGTCCACTAAAGAGGCTTCTTTTTGCTAGGAGT

CACTAGTTAGTTTTTTTTTTTTTTGACAAAAGTTAACCATTTTTTTTTATCGCATGGTAGG

AAAAAAAAAAAAGAGATTTCCTTTGGTACTCATAAAAAAAAAAGTTGGATGGATAATGGAT

TAATCATAACTTATTTAACATAAAATGAACTAAGAGAACATTTCATATTTGGGTTTATTTT

GTAACATAAATATTAATATCAATGTTTGAAAGAGTTTTTAACAATAATTTGTAAAAGAAAG

CATAATATATCTATCAAAATAATTATAAAAGAAAAAATTTCTTTTGGTGGTATGACATGGA

AATATAATTAATTCTTATTAGATGAAAGTGTAAAATTAATTTGCATTAACAAAGCATATTT

CATTAAACTCCATTTAGTTTTTGTTGGAAAAAATAAATTTATTGTTACTCAAAAAATAAAA

GTGTACAATTAATATATTTACTCAAAAGTCCCCTAAATGGGATGTAATTATCAAGGGATGT

GAGCGGTTTAACTTTAAGTAATGAATCAACTGTTGCATAATCACTTTGAAAGAAACTTGAG

ATTCTTCAATTAGCAAAATCTTGAATAGAATAATGAGATTATTCCTAATGCTAATGATACC

CACCTCTCCTATAAGTTGATGGGGCAGAACAAATTATGCACTCAGGACAACTTTTCTCAAA

TTTTGAAAACTTACCACACGAAGAACACACTTCCACTTGCACAAGTAAGAATATAGTATAA

CAATAAATTTACCACTCATAGCTCAATGGATATTAAAATATTGCTAATCTTTTATAGACAG

ATGTTGGCAAATTAGTATAAATTAGTTATTATATTAGTTTTTAAGGTTCAATATACTTTTT

AAAACTCTTATTAAGTTGTTTAGCTTGCCAACCAAAGACTAAATTATCTTTATTTGTTAGG

AAATAAAAGCACTTATTTTATTTTCTCCATATTTGTTTCCTCTTTGTTCCATTACTAAAAA

AATTATAATATATTAATAACAAAAAAATTTGGGGAGGGAATGTTATTTTTAAAAAAGATGT

TTTATTTATATTTAAGTGTTTTATTGAAATTCCTCGTGAGTTTAGCTCAATTGGTATGGAC

AATACATAATATATGCAAAATTCGAGGTTCAAACCTTGAGCACCACAAAAAAAAGTGTTTT

ATTGAAAAATATCAAGAACTTGAAAACAAATTTCCTTTTTATGAACTAAATATTGTCACAT

AGACAACTTAAGAATTGACTTTTTTTTTAACACTATTGTATTTTCTGAACTCGACTTTAAC

TCGAGATCAATGAGTAAACTAAAAAAACTCGTATTATTTTATCTAAGTGTTCTTAGTGTGA

GAAAACATTATAATTGTTTGCTTAGTCTATTTCTCCCTATATTCTCACCCTCATTCTTGGT

TTGCACATAGAAAGGAGAAAAAAAAAATTAGAGAAGAGTTGAATGGAACAGAAAGAAAGAG

CAAGCAAAATAATCACTAAAAAAAATTCCCCTAAAAGAAAATCACTGAAAAATATTATCCA

CCTCTATTTTAAATAATAATAATAATGAAAACAACAATATATTTTTTTATAGATTTTCTCT

ACTAGAGGTAATTTATTTTAAACACTAAATGTCCACACCTCTAACAAAAAAAATGCATAAT

ATTATAATATATTATTGTAAGAAAGAGTGACAAATGTTGAATTAGTTATGGAATGAAGAGT

GAGAGAGATTATACAAATAAATAAATATAAATATAAATATAAATAAAGAATAGAGAAGAAC

ATAGTACTTGAATGAAGAGATTTGGCTTGTACTTCCATTACACTTGTAAGCTTTCTACTTC

CTTCACATTCCTTCTCTTTCTCTATCTTTCCTTTTATGATATGTGTACTTTGTTATTGCTT

CATTTACTACCGACATGACTCCAACCTATTAGGGCTTTATCTGAATAATGTTAGTTATTTT

CTCTCATTTTCTTTGCTTCTTCTTATTCTTGGAACTTATAAAGGGTCTTGTCAATTCTATC

GTTCTCTTTTGTATACTTACAAATATAAATATTCTCTTTAGCTTCATTTTCATGTCTTCTT

CTACTAATAAGCTATGGAAATTGAAGTATTTGTTTTCATGTTTATTTTTCACTTCAGCTCC

TCTTTTTTGATGTTGATGCTGAAGATTGAATAAAAACTGAGGTGGTATGTATGAGAACAAG

AACATGTTTCATTCATCTCTGTTATTTGAACATCTAAATTTTAAACAACTATCGCGACAAT

TTTTTAAGTGGTTGAAGTATTTTAAATAGGTGGTTAATTACATTAAGTAGATAGTTAATTG

ATATATCAGGTGTCAAAACTCATTAACCATCAACATAACTTCATTAGCCATAAATTTTTGA

CGTGATTAACCAAATATTGTAGATGCTCAATTACATGTAGATTTGATGGAGTTGTCCACAA

TTTCTCATATATATATTAACTAATAACCATCTACCGAATATAATTAACCATATATTAGAAT

ATCATTAACCTCTAAAACAGTTGTCGCGAAAATTGGTTGTAAAAAAATCATTTCTATTTTT

TACGCCACTAGTTCTCTTGTTATTTCTCTTTGTTGGAAAGTTGTTGTTTTTAACTTTGAGC

TTAGTCTTATTAATTAACTTGTAATGGTAACAACATTTTTCTATCTTTGTTTGAAGGAAGT

AGTTACTGTGTCAACTTTAGTAATTAATTTGGATCATTTTGGAGGCAAAATATGGCTATGC

AACCTGTTTATTTTAAAGAACATGAAGGAAATGTCCACAATTCTGTTGGACAGTTTTCATC

TGTGACTTCAGCACCATGGTGGAGTAATGCCTATGGATCTCAACCTTTTTATGGAGGAGAC

TCTTGTGGCCAAATGAAACCTTTTTCACTAGAGCTTTCCAACTACATAGACCAACTTGCTC

CGAGTAAGAACTTAGTTCGAGGAGTTGAACAATTGTTTGATAAAGGGCATACAAACCAATT

CACCATCTTTCCAGGTACTTGTTATTCAATATAATTCCGGTTTTGAATGAATTGATTTTTG

TTAAGTTGGTTCTGGATAAACGTGCGTTAAATATAATATGATTTATGTTTGGATACGTTTA

TGTTAAAGTGAGTTGAACATAAATTGGAGACTAAATATCAATTGCAGAGGCAAAAGCTTCA

AATTCTAATTTCAAGTTAGAATCAAATCAATTCTACTCGTAAGCATCTAAATGTTTCAAAA

CCAATTCTACATCTCTAGAACTAAATTTGCATTCAAACATTTTCTGCTACAGATGATTGTA

AGATGTCAGTTGATACACCAAATCATCAGGCAACCTTATCCCTGCAATCACCATTTGCTGC

CGAGCCACTTAATCGATTTGAGCTAGGTTTTAACCAGTCTATGGTAAATCTTCTTATTAAA

GCAATCCCTTTGATCATGTTTTAGTATTTTACTTTTGATGTACATAAATTCATTATGTTTT

TATTATCCTTTCCATGCTAGATCTGTGCAAAATATCCGTATATAGATCAATTTTACGGGCT

CTTCTCGACTTATGGACCTCAAATCTCGGTTTGTCTTCACCTCGGCTATTTTATTAATCTT

TGTATATATCATGCAATGTCTATAAGTACTTGAATGTGAATTTGTTTTATTTACAGTGTGT

ATGCTTACATGAGAAAGCCAGAAATCTGTTTACAACTATCTTGTTAGTATTCTTCTAAATC

TTTCAAATGTAGAGTTGATTATGAAATTTGTTGTTAATGAGTTTTCGGCTTTATGAATGAA

TTGTAAGACAAGTAATGAAACAAAAACTAGAAGAAAATTGAATTATATGATGCAATCTGTG

TTGAAGTTTTTGCATGTATTCAAGTTCTGCTTTTAGTTACTATTTGTTTATCTGCTGCAAT

ATTTCATCAATTATTATTCATGTATGTACCAATATTTAATAAATTAGGGGCGAATTATGCT

TCCGCTTAGCATGACATCTGACGACGGACCAACATACGTGAATGCTAAGCAATACCATGGA

ATCATCAGACGCAGGCATTCTCGTGCCAAAGCTGTGCTTCAGAATAAATTGATAAAGCGTA

ACAAGGTATGAAACTGAGTATTTTTCCTCACAACAATGTTCGAAAACTTGTGAAATAGTAT

ATTTTTCCTTTTACCCTTTTTATACCTATATTAAGGTTTTGTATTTGTCTTGCAGCCATAT

ATGCACGAATCGCGTCATCTACATGCAATGCGTAGACCAAGAGGATGCGGTGGTCGTTTCT

TGAACACAAAAGTTTCTGCTAATGGAAACGGTAAAAGTGGGAGTGAAGAGAACGGAAACAT

TGGTGGCCTACAGCTGCAGTCCAGTGGTTCTCAGAGTTCCGAAGTCTTACAATCTGAGGTT

GGAACTTTAAATTCGTCGAAGGAGACAAACGGAGGCAGTCCAAATGTCTCGGGGTCAGAGG

TGACTAGCATGTATACACAGGGAGGTCTTGATAGCTTTACTGTCAATCATATCGGATCTAC

TGTCCACTCTTTGGGAGACATGATCGATACTGGACACGGTATCGTCATGCCAACAAAATGG

TTTGCAGCAGCTGGCAGACAGCTGCTGGAACCATAAGTTTCGATTCAGAAAGGAAACAAGT

GGGTTTGGTACAATGTGAAATATTTTGCACCAAACTCATCCTTTCCGAGACCAGATGAAGA

AGCTATGTTTCAGTTTGTTGTGTTTACTACGACAAATTTAGTTTTGGAAGACTACTTTTCC

ATCTGGTGCTCAGGCAACTCATTCTTGGCTTATTCTCAGGAAACTCATCCTTGGCTCGTAA

TATTTAGTAGTATTGTCATCGTCTTCCCGCGCAGGCTTTCCGTGGCATGGTAGGCATGCTA

ATGACTTTGGTATTTTCATGCAGTTATAACTATGATGTGTCTTTGTTTGTTGTTAAAATAA

AAAACATGAACTCTAGCTAGGTGCATGTGTGTGTTTTTAATCTTGTCTACTAAGTTTGGTG

TTTTGTAATGGATTTCTGACTTTATGGAGCAATGTATTGTAACTCTACTAAGAAGTGTAAC

ATTTTATTTCTCCCTCTCTAAGGATTGTATAAGAACCTCTTATTTTCAGACTCTACTTAAT

CCTATTTTCTATGTCTGTATGATTTTTATATTTCTAGGACAATCAAATTGGCTTGTAGAAG

CTCAAAAGCATGCTCACAAAGTAGGTACTTATGTAGGGAACTTCGTACTCTAATAATAACT

GGTTATTAACGTTATAATTAAATGCAAAATTTGATAAGTAGTAGGGTTTGGTAAGTAATAT

AACAACACCATATTGGCTTGGGATTCTGAATTGTGTTATTAGAGTACAACTTTTAAATAAT

GATCGTAATGTAAATAATTTAAATTTGAAGTTACTAGATAAAAAGATAATATTATGTAATA

TATTATCAAATATGTACTAAAAATGTAATTTTCATCTATCTCAATTGAAAAAATATATGAG

TTTTGAGTCTATTGTCATGGACGA

>HM008

AAAAAAATAAAGGTTCTATACTCATTAAACAAAGTGCCAAATTCAGAGATACTACTTCCT

TTGGATAATTTGTATCGTCACCAACGATCTTTGAGTTATTTCAAAAATCACATTCTCAATT

TTGTGGTCCAACATTCACAAAATGGTTTGTCGGGCTTTAAGTCTAGTAATTGTGAAACATG

CATATGCATGAGGTAATTAATTGATAATCTGAAAAGGTTAAATAGTTAATCAATTGTACTT

TGGTTCTTTTTAATTTGTTCTCTATAATTTTTCAGTCAAAATTGAATTTGCTTTAACCATT

TAGTGTAGTAGTATGATATTACTTGCTCTACATTCTTGGATCTAAAGGTCTAATTAGTATC

GTATAAAAAATATTCTAGTTAATTAAGATACTTTTGTTCATTCTAATTATTCTTGGATTAC

TTGTGAAACTTTTAGGGATGAACAAGAAAGTCCACTAAAGAGGCTTCTTTTTGCTAGGAGT

CACTAGTTAGTTTTTTTTTTTTTTGACAAAAGTTAACCATTTTTTTTTATCGCATGGTAGG

AAAAAAAAAAAAGAGATTTCCTTTGGTACTCATAAAAAAAAAAGTTGGATGGATAATGGAT

TAATCATAACTTATTTAACATAAAATGAACTAAGAGAACATTTCATATTTGGGTTTATTTT

GTAACATAAATATTAATATCAATGTTTGAAAGAGTTTTTAACAATAATTTGTAAAAGAAAG

CATAATATATCTATCAAAATAATTATAAAAGAAAAAATTTCTTTTGGTGGTATGACATGGA

AATATAATTAATTCTTATTAGATGAAAGTGTAAAATTAATTTGCATTAACAAAGCATATTT

CATTAAACTCCTTTTAGTTTTTGTTGGAAAAAATAAATTTATTGTTACTCAAAAAATAAAA

GTGTACAATTAATATATTTACTCAAAAGTCCCCTAAATGGGATGTAATTATCAAGGGATGT

GAGCGGTTTAACTTTAAGTAATGAATCAACTGTTGCATAATCACTTTGAAAGAAACTTGAG

ATTCTTCAATTAGCAAAATCTTGAATAGAATAATGAGATTATTCCTAATGCTAATGATACC

CACCTCTCCTATAAGTTGATGGGGCAGAACAAATTATGCACTCAGGACAACTTTTCTCAAA

TTTTGAAAACTTACCACACGAAGAACACACTTCCACTTGCACAAGTAAGAATATAGTATAA

CAATAAATTTACCACTCATAGCTCAATGGATATTAAAATATTGCTAATCTTTTATAGACAG

ATGTTGGCAAATTAGTATAAATTAGTTATTATATTAGTTTTTAAGGTTCAATATACTTTTT

AAAACTCTTATTAAGTTGTTTAGCTTGCCAACCAAAGACTAAATTATCTTTATTTGTTAGG

AAATAAAAGCACTTATTTTATTTTCTCCATATTTGTTTCCTCTTTGTTCCATTACTAAAAA

AATTATAATATATTAATAACAAAAAAATTTGGGGAGGGAATGTTATTTTTAAAAAAGATGT

TTTATTTATATTTAAGTGTTTTATTGAAATTCCTCGTGAGTTTAGCTCAATTGGTATGGAC

AATACATAATATATGCAAAATTCGAGGTTCAAACCTTGAGCACCACAAAAAAAAGTGTTTT

ATTGAAAAATATCAAGAACTTGAAAACAAATTTCCTTTTTATGAACTAAATATTGTCACAT

AGACAACTTAAGAATTGACTTTTTTTTTAACACTATTGTATTTTCTGAACTCGACTTTAAC

TCGAGATCAATGAGTAAACTAAAAAAACTCGTATTATTTTATCTAAGTGTTCTTAGTGTGA

GAAAACATTATAATTGTTTGCTTAGTCTATTTCTCCCTATATTCTCACCCTCATTCTTGGT

TTGCACATAGAAAGGAGAAAAAAAAAATTAGAGAAGAGTTGAATGGAACAGAAAGAAAGAG

CAAGCAAAATAATCACTAAAAAAAATTCCCCTAAAAGAAAATCACTGAAAAATATTATCCA

CCTCTATTTTAAATAATAATAATAATGAAAACAACAATATATTTTTTTATAGATTTTCTCT

ACTAGAGGTAATTTATTTTAAACACTAAATGTCCACACCTCTAACAAAAAAAATGCATAAT

ATTATAATATATTATTGTAAGAAAGAGTGACAAATGTTGAATTAGTTATGGAATGAAGAGT

GAGAGAGATTATACAAATAAATAAATATAAATATAAATATAAATAAAGAATAGAGAAGAAC

ATAGTACTTGAATGAAGAGATTTGGCTTGTACTTCCATTACACTTGTAAGCTTTCTACTTC

CTTCACATTCCTTCTCTTTCTCTATCTTTCCTTTTATGATATGTGTACTTTGTTATTGCTT

CATTTACTACCGACATGACTCCAACCTATTAGGGCTTTATCTGAATAATGTTAGTTATTTT

CTCTCATTTTCTTTGCTTCTTCTTATTCTTGGAACTTATAAAGGGTCTTGTCAATTCTATC

GTTCTCTTTTGTATACTTACAAATATAAATATTCTCTTTAGCTTCATTTTCATGTCTTCTT

CTACTAATAAGCTATGGAAATTGAAGTATTTGTTTTCATGTTTATTTTTCACTTCAGCTCC

TCTTTTTTGATGTTGATGCTGAAGATTGAATAAAAACTGAGGTGGTATGTATGAGAACAAG

AACATGTTTCATTCATCTCTGTTATTTGAACATCTAAATTTTAAACAACTATCGCGACAAT

TTTTTAAGTGGTTGAAGTATTTTAAATAGGTGGTTAATTACATTAAGTAGATAGTTAATTG

ATATATCAGGTGTCAAAACTCATTAACCATCAACATAACTTCATTAGCCATAAATTTTTGA

CGTGATTAACCAAATATTGTAGATGCTCAATTACATGTAGATTTGATGGAGTTGTCCACAA

TTTCTCATATATATATTAACTAATAACCATCTACCGAATATAATTAACCATATATTAGAAT

ATCATTAACCTCTAAAACAGTTGTCGCGAAAATTGGTTGTAAAAAAATCATTTCTATTTTT

TACGCCACTAGTTCTCTTGTTATTTCTCTTTGTTGGAAAGTTGTTGTTTTTAACTTTGAGC

TTAGTCTTATTAATTAACTTGTAATGGTAACAACATTTTTCTATCTTTGTTTGAAGGAAGT

AGTTACTGTGTCAACTTTAGTAATTAATTTGGATCATTTTGGAGGCAAAATATGGCTATGC

AACCTGTTTATTTTAAAGAACATGAAGGAAATGTCCACAATTCTGTTGGACAGTTTTCATC

TGTGACTTCAGCACCATGGTGGAGTAATGCCTATGGATCTCAACCTTTTTATGGAGGAGAC

TCTTGTGGCCAAATGAAACCTTTTTCACTAGAGCTTTCCAACTACATAGACCAACTTGCTC

CGAGTAAGAACTTAGTTCGAGGAGTTGAACAATTGTTTGATAAAGGGCATACAAACCAATT

CACCATCTTTCCAGGTACTTGTTATTCAATATAATTCCGGTTTTGAATGAATTGATTTTTG

TTAAGTTGGTTCTGGATAAACGTGCGTTAAATATAATATGATTTATGTTTGGATACGTTTA

TGTTAAAGTGAGTTGAACATAAATTGGAGACTAAATATCAATTGCAGAGGCAAAAGCTTCA

AATTCTAATTTCAAGTTAGAATCAAATCAATTCTACTCGTAAGCATCTAAATGTTTCAAAA

CCAATTCTACATCTCTAGAACTAAATTTGCATTCAAACATTTTCTGCTACAGATGATTGTA

AGATGTCAGTTGATACACCAAATCATCAAGCAACCTTATCCCTGCAATCACCATTTGCTGC

CGAGCCACTTAATCGATTTGAGCTAGGTTTTAACCAGTCTATGGTAAATCTTCTTATTAAA

GCAATCCCTTTGATCATGTTTTAGTATTTTACTTTTGATGTACATAAATTCATTATGTTTT

TATTATCCTTTCCATGCTAGATCTGTGCAAAATATCCGTATATAGATCAATTTTACGGGCT

CTTCTCGACTTATGGACCTCAAATCTCGGTTTGTCTTCACCTCGGCTATTTTATTAATCTT

TGTATATATCATGCAATGTCTATAAGTACTTGAATGTGAATTTGTTTTATTTACAGTGTGT

ATGCTTACATGAGAAAGCCAGAAATCTGTTTACAACTATCTTGTTAGTATTCTTCTAAATC

TTTCAAATGTAGAGTTGATTATGAAATTTGTTGTTAATGAGTTTTCGGCTTTATGAATGAA

TTGTAAGACAAGTAATGAAACAAAAACTAGAAGAAAATTGAATTATATGATGCAATCTGTG

TTGAAGTTTTTGCATGTATTCAAGTTCTGCTTTTAGTTACTATTTGTTTATCTGCTGCAAT

ATTTCATCAATTATTATTCATGTATGTACCAATATTTAATAAATTAGGGGCGAATTATGCT

TCCGCTTAGCATGACATCTGACGACGGACCAACATACGTGAATGCTAAGCAATACCATGGA

ATCATCAGACGCAGGCATTCTCGTGCCAAAGCTGTGCTTCAGAATAAATTGATAAAGCGTA

ACAAGGTATGAAACTGAGTATTTTTCCTCACAACAATGTTCGAAAACTTGTGAAATAGTAT

ATTTTTCCTTTTACCCTTTTTATACCTATATTAAGGTTTTGTATTTGTCTTGCAGCCATAT

ATGCACGAATCGCGTCATCTACATGCAATGCGTAGACCAAGAGGATGCGGTGGTCGTTTCT

TGAACACAAAAGTTTCTGCTAATGGAAACGGTAAAAGTGGGAGTGAAGAGAACGGAAACAT

TGGTGGCCTACAGCTGCAGTCCAGTGGTTCTCAGAGTTCCGAAGTCTTACAATCTGAGGTT

GGAACTTTAAATTCGTCGAAGGAGACAAACGGAGGCAGTCCAAATGTCTCGGGGTCAGAGG

TGACTAGCATGTATACACAGGGAGGTCTTGATAGCTTTACTGTCAATCATATCGGATCTAC

TGTCCACTCTTTGGGAGACATGATCGATACTGGACACGGTATCGTCATGCCAACAAAATGG

TTTGCAGCAGCTGGCAGACAGCTGCTGGAACCATAAGTTTCGATTCAGAAAGGAAACAAGT

GGGTTTGGTACAATGTGAAATATTTTGCACCAAACTCATCCTTTCCGAGACCAGATGAAGA

AGCTATGTTTCAGTTTGTTGTGTTTACTACGACAAATTTAGTTTTGGAAGACTACTTTTCC

ATCTGGTGCTCAGGCAACTCATTCTTGGCTTATTCTCAGGAAACTCATCCTTGGCTCGTAA

TATTTAGTAGTATTGTCATCGTCTTCCCGCGCAGGCTTTCCGTGGCATGGTAGGCATGCTA

ATGACTTTGGTATTTTCATGCAGTTATAACTATGATGTGTCTTTGTTTGTTGTTAAAATAA

AAAACATGAACTCTAGCTAGGTGCATGTGTGTGTTTTTAATCTTGTCTACTAAGTTTGGTG

TTTTGTAATGGATTTCTGACTTTATGGAGCAATGTATTGTAACTCTACTAAGAAGTGTAAC

ATTTTATTTCTCCCTCTCTAAGGATTGTATAAGAACCTCTTATTTTCAGACTCTACTTAAT

CCTATTTTCTATGTCTGTATGATTTTTATATTTCTAGGACAATCAAATTGGCTTGTAGAAG

CTCAAAAGCATGCTCACAAAGTAGGTACTTATGTAGGGAACTTCGTACTCTAATAATAACT

GGTTATTAACGTTATAATTAAATGCAAAATTTGATAAGTAGTAGGGTTTGGTAAGTAATAT

AACAACACCATATTGGCTTGGGATTCTGAATTGTGTTATTAGAGTACAACTTTTAAATAAT

GATCGTAATGTAAATAATTTAAATTTGAAGTTACTAGATAAAAAGATAATATTATGTAATA

TATTATCAAATATGTACTAAAAATGTAATTTTCATCTATCTCAATTGAAAAAATATATGAG

TTTTGAGTCTATTGTCATGGACGA

>HM009

AAAAAAATAAAGGTTCTATACTCATTAAACAAAGTGCCAAATTCAGAGATACTACTTCCT

TTGGATAATTTGTATCGTCACCAACGATCTTTGAGTTATTTCAAAAATCACATTCTCAATT

TTGTGGTCCAACATTCACAAAATGGTTTGTCGGGCTTTAAGTCTAGTAATTGTGAAACATG

CATATGCATGAGGTAATTAATTGATAATCTGAAAAGGTTAAATAGTTAATCAATTGTACTT

TGGTTCTTTTTAATTTGTTCTCTATAATTTTTCAGTCAAAATTGAATTTGCTTTAACCATT

TAGTGTAGTAGTATGATATTACTTGCTCTACATTCTTGGATCTAAAGGTCTAATTAGTATC

GTATAAAAAATATTCTAGTTAATTAAGATACTTTTGTTCATTCTAATTATTCTTGGATTAC

TTGTGAAACTTTTAGGGATGAACAAGAAAGTCCACTAAAGAGGCTTCTTTTTGCTAGGAGT

CACTAGTTAGTTTTTTTTTTTTTTGACAAAAGTTAACCATTTTTTTTTATCGCATGGTAGG

AAAAAAAAAAAAGAGATTTCCTTTGGTACTCATAAAAAAAAAAGTTGGATGGATAATGGAT

TAATCATAACTTATTTAACATAAAATGAACTAAGAGAACATTTCATATTTGGGTTTATTTT

GTAACATAAATATTAATATCAATGTTTGAAAGAGTTTTTAACAATAATTTGTAAAAGAAAG

CATAATATATCTATCAAAATAATTATAAAAGAAAAAATTTCTTTTGGTGGTATGACATGGA

AATATAATTAATTCTTATTAGATGAAAGTGTAAAATTAATTTGCATTAACAAAGCATATTT

CATTAAACTCCTTTTAGTTTTTGTTGGAAAAAATAAATTTATTGTTACTCAAAAAATAAAA

GTGTACAATTAATATATTTACTCAAAAGTCCCCTAAATGGGATGTAATTATCAAGGGATGT

GAGCGGTTTAACTTTAAGTAATGAATCAACTGTTGCATAATCACTTTGAAAGAAACTTGAG

ATTCTTCAATTAGCAAAATCTTGAATAGAATAATGAGATTATTCCTAATGCTAATGATACC

CACCTCTCCTATAAGTTGATGGGGCAGAACAAATTATGCACTCAGGACAACTTTTCTCAAA

TTTTGAAAACTTACCACACGAAGAACACACTTCCACTTGCACAAGTAAGAATATAGTATAA

CAATAAATTTACCACTCATAGCTCAATGGATATTAAAATATTGCTAATCTTTTATAGACAG

ATGTTGGCAAATTAGTATAAATTAGTTATTATATTAGTTTTTAAGGTTCAATATACTTTTT

AAAACTCTTATTAAGTTGTTTAGCTTGCCAACCAAAGACTAAATTATCTTTATTTGTTAGG

AAATAAAAGCACTTATTTTATTTTCTCCATATTTGTTTCCTCTTTGTTCCATTACTAAAAA

AATTATAATATATTAATAACAAAAAAATTTGGGGAGGGAATGTTATTTTTAAAAAAGATGT

TTTATTTATATTTAAGTGTTTTATTGAAATTCCTCGTGAGTTTAGCTCAATTGGTATGGAC

AATACATAATATATGCAAAATTCGAGGTTCAAACCTTGAGCACCACAAAAAAAAGTGTTTT

ATTGAAAAATATCAAGAACTTGAAAACAAATTTCCTTTTTATGAACTAGATATTGTCACAT

AGACAACTTAAGAATTGACTTTTTTTTTAACACTATTGTATTTTCTGAACTCGACTTTAAC

TCGAGATCAATGAGTAAACTAAAAAAACTCGTATTATTTTATCTAAGTGTTCTTAGTGTGA

GAAAACATTATAATTGTTTGCTTAGTCTATTTCTCCCTATATTCTCACCCTCATTCTTGGT

TTGCACATAGAAAGGAGAAAAAAAAAATTAGAGAAGAGTTGAATGGAACAGAAAGAAAGAG

CAAGCAAAATAATCACTAAAAAAAATTCCCCTAAAAGAAAATCACTGAAAAATATTATCCA

CCTCTATTTTAAATAATAATAATAATGAAAACAACAATATATTTTTTTATAGATTTTCTCT

ACTAGAGGTAATTTATTTTAAACACTAAATGTCCACACCTCTAACAAAAAAAATGCATAAT

ATTATAATATATTATTGTAAGAAAGAGTGACAAATGTTGAATTAGTTATGGAATGAAGAGT

GAGAGAGATTATACAAATAAATAAATATAAATATAAATATAAATAAAGAATAGAGAAGAAC

ATAGTACTTGAATGAAGAGATTTGGCTTGTACTTCCATTACACTTGTAAGCTTTCTACTTC

CTTCACATTCCTTCTCTTTCTCTATCTTTCCTTTTATGATATGTGTACTTTGTTATTGCTT

CATTTACTACCGACATGACTCCAACCTATTAGGGCTTTATCTGAATAATGTTAGTTATTTT

CTCTCATTTTCTTTGCTTCTTCTTATTCTTGGAACTTATAAAGGGTCTTGTCAATTCTATC

GTTCTCTTTTGTATACTTACAAATATAAATATTCTCTTTAGCTTCATTTTCATGTCTTCTT

CTACTAATAAGCTATGGAAATTGAAGTATTTGTTTTCATGTTTATTTTTCACTTCAGCTCC

TCTTTTTTGATGTTGATGCTGAAGATTGAATAAAAACTGAGGTGGTATGTATGAGAACAAG

AACATGTTTCATTCATCTCTGTTATTTGAACATCTAAATTTTAAACAACTATCGCGACAAT

TTTTTAAGTGGTTGAAGTATTTTAAATAGGTGGTTAATTACATTAAGTAGATAGTTAATTG

ATATATCAGGTGTCAAAACTCATTAACCATCAACATAACTTCATTAGCCATAAATTTTTGA

CGTGATTAACCAAATATTGTAGATGCTCAATTACATGTAGATTTGATGGAGTTGTCCACAA

TTTCTCATATATATATTAACTAATAACCATCTACCGAATATAATTAACCATATATTAGAAT

ATCATTAACCTCTAAAACAGTTGTCGCGAAAATTGGTTGTAAAAAAATCATTTCTATTTTT

TACGCCACTAGTTCTCTTGTTATTTCTCTTTGTTGGAAAGTTGTTGTTTTTAACTTTGAGC

TTAGTCTTATTAATTAACTTGTAATGGTAACAACATTTTTCTATCTTTGTTTGAAGGAAGT

AGTTACTGTGTCAACTTTAGTAATTAATTTGGATCATTTTGGAGGCAAAATATGGCTATGC

AACCTGTTTATTTTAAAGAACATGAAGGAAATGTCCACAATTCTGTTGGACAGTTTTCATC

TGTGACTTCAGCACCATGGTGGAGTAATGCCTATGGATCTCAACCTTTTTATGGAGGAGAC

TCTTGTGGCCAAATGAAACCTTTTTCACTAGAGCTTTCCAACTACATAGACCAACTTGCTC

CGAGTAAGAACTTAGTTCGAGGAGTTGAACAATTGTTTGATAAAGGGCATACAAACCAATT

CACCATCTTTCCAGGTACTTGTTATTCAATATAATTCCGGTTTTGAATGAATTGATTTTTG

TTAAGTTGGTTCTGGATAAACGTGCGTTAAATATAATATGATTTATGTTTGGATACGTTTA

TGTTAAAGTGAGTTGAACATAAATTGGAGACTAAATATCAATTGCAGAGGCAAAAGCTTCA

AATTCTAATTTCAAGTTAGAATCAAATCAATTCTACTCGTAAGCATCTAAATGTTTCAAAA

CCAATTCTACATCTCTAGAACTAAATTTGCATTCAAACATTTTCTGCTACAGATGATTGTA

AGATGTCAGTTGATACACCAAATCATCAAGCAACCTTATCCCTGCAATCACCATTTGCTGC

CGAGCCACTTAATCGATTTGAGCTAGGTTTTAACCAGTCTATGGTAAATCTTCTTATTAAA

GCAATCCCTTTGATCATGTTTTAGTATTTTACTTTTGATGTACATAAATTCATTATGTTTT

TATTATCCTTTCCATGCTAGATCTGTGCAAAATATCCGTATATAGATCAATTTTACGGGCT

CTTCTCGACTTATGGACCTCAAATCTCGGTTTGTCTTCACCTCGGCTATTTTATTAATCTT

TGTATATATCATGCAATGTCTATAAGTACTTGAATGTGAATTTGTTTTATTTACAGTGTGT

ATGCTTACATGAGAAAGCCAGAAATCTGTTTACAACTATCTTGTTAGTATTCTTCTAAATC

TTTCAAATGTAGAGTTGATTATGAAATTTGTTGTTAATGAGTTTTCGGCTTTATGAATGAA

TTGTAAGACAAGTAATGAAACAAAAACTAGAAGAAAATTGAATTATATGATGCAATCTGTG

TTGAAGTTTTTGCATGTATTCAAGTTCTGCTTTTAGTTACTATTTGTTTATCTGCTGCAAT

ATTTCATCAATTATTATTCATGTATGTACCAATATTTAATAAATTAGGGGCGAATTATGCT

TCCGCTTAGCATGACATCTGACGACGGACCAACATACGTGAATGCTAAGCAATACCATGGA

ATCATCAGACGCAGGCATTCTCGTGCCAAAGCTGTGCTTCAGAATAAATTGATAAAGCGTA

ACAAGGTATGAAACTGAGTATTTTTCCTCACAACAATGTTCGAAAACTTGTGAAATAGTAT

ATTTTTCCTTTTACCCTTTTTATACCTATATTAAGGTTTTGTATTTGTCTTGCAGCCATAT

ATGCACGAATCGCGTCATCTACATGCAATGCGTAGACCAAGAGGATGCGGTGGTCGTTTCT

TGAACACAAAAGTTTCTGCTAATGGAAACGGTAAAAGTGGGAGTGAAGAGAACGGAAACAT

TGGTGGCCTACAGCTGCAGTCCAGTGGTTCTCAGAGTTCCGAAGTCTTACAATCTGAGGTT

GGAACTTTAAATTCGTCGAAGGAGACAAACGGAGGCAGTCCAAATGTCTCGGGGTCAGAGG

TGACTAGCATGTATACACAGGGAGGTCTTGATAGCTTTACTGTCAATCATATCGGATCTAC

TGTCCACTCTTTGGGAGACATGATCGATACTGGACACGGTATCGTCATGCCAACAAAATGG

TTTGCAGCAGCTGGCAGACAGCTGCTGGAACCATAAGTTTCGATTCAGAAAGGAAACAAGT

GGGTTTGGTACAATGTGAAATATTTTGCACCAAACTCATCCTTTCCGAGACCAGATGAAGA

AGCTATGTTTCAGTTTGTTGTGTTTACTACGACAAATTTAGTTTTGGAAGACTACTTTTCC

ATCTGGTGCTCAGGCAACTCATTCTTGGCTTATTCTCAGGAAACTCATCCTTGGCTCGTAA

TATTTAGTAGTATTGTCATCGTCTTCCCGCGCAGGCTTTCCGTGGCATGGTAGGCATGCTA

ATGACTTTGGTATTTTCATGCAGTTATAACTATGATGTGTCTTTGTTTGTTGTTAAAATAA

AAAACATGAACTCTAGCTAGGTGCATGTGTGTGTTTTTAATCTTGTCTACTAAGTTTGGTG

TTTTGTAATGGATTTCTGACTTTATGGAGCAATGTATTGTAACTCTACTAAGAAGTGTAAC

ATTTTATTTCTCCCTCTCTAAGGATTGTATAAGAACCTCTTATTTTCAGACTCTACTTAAT

CCTATTTTCTATGTCTGTATGATTTTTATATTTCTAGGACAATCAAATTGGCTTGTAGAAG

CTCAAAAGCATGCTCACAAAGTAGGTACTTATGTAGGGAACTTCGTACTCTAATAATAACT

GGTTATTAACGTTATAATTAAATGCAAAATTTGATAAGTAGTAGGGTTTGGTAAGTAATAT

AACAACACCATATTGGCTTGGGATTCTGAATTGTGTTATTAGAGTACAACTTTTAAATAAT

GATCGTAATGTAAATAATTTAAATTTGAAGTTACTAGATAAAAAGATAATATTATGTAATA

TATTATCAAATATGTACTAAAAATGTAATTTTCATCTATCTCAATTGAAAAAATATCTGAG

TTTTGAGTCTATTGTCATGGACGA

>HM010

AAAAAAATAAAGGTTCTATACTCATTAAACAAAGTGCCAAATTCAGAGATACTACTTCCT

TTGGATAATTTGTATCGTCACCAACGATCTTTGAGTTATTTCAAAAATCACATTCTCAATT

TTGTGGTCCAACATTCACAAAATGGTTTGTCGGGCTTTAAGTCTAGTAATTGTGAAACATG

CATATGCATGAGGTAATTAATTGATAATCTGAAAAGGTTAAATAGTTAATCAATTGTACTT

TGGTTCTTTTTAATTTGTTCTCTATAATTTTTCAGTCAAAATTGAATTTGCTTTAACCATT

TAGTGTAGTAGTATGATATTACTTGCTCTACATTCTTGGATCTAAAGGTCTAATTAGTATC

GTATAAAAAATATTCTAGTTAATTAAGATACTTTTGTTCATTCTAATTATTCTTGGATTAC

TTGTGAAACTTTTAGGGATGAACAAGAAAGTCCACTAAAGAGGCTTCTTTTTGCTAGGAGT

CACTAGTTAGTTTTTTTTTTTTTTGACAAAAGTTAACCATTTTTTTTTATCGCATGGTAGG

AAAAAAAAAAAAGAGATTTCCTTTGGTACTCATAAAAAAAAAAGTTGGATGGATAATGGAT

TAATCATAACTTATTTAACATAAAATGAACTAAGAGAACATTTCATATTTGGGTTTATTTT

GTAACATAAATATTAATATCAATGTTTGAAAGAGTTTTTAACAATAATTTGTAAAAGAAAG

CATAATATATCTATCAAAATAATTATAAAAGAAAAAATTTCTTTTGGTGGTATGACATGGA

AATATAATTAATTCTTATTAGATGAAAGTGTAAAATTAATTTGCATTAACAAAGCATATTT

CATTAAACTCCTTTTAGTTTTTGTTGGAAAAAATAAATTTATTGTTACTCAAAAAATAAAA

GTGTACAATTAATATATTTACTCAAAAGTCCCCTAAATGGGATGTAATTATCAAGGGATGT

GAGCGGTTTAACTTTAAGTAATGAATCAACTGTTGCATAATCACTTTGAAAGAAACTTGAG

ATTCTTCAATTAGCAAAATCTTGAATAGAATAATGAGATTATTCCTAATGCTAATGATACC

CACCTCTCCTATAAGTTGATGGGGCAGAACAAATTATGCACTCAGGACAACTTTTCTCAAA

TTTTGAAAACTTACCACACGAAGAACACACTTCCACTTGCACAAGTAAGAATATAGTATAA

CAATAAATTTACCACTCATAGCTCAATGGATATTAAAATATTGCTAATCTTTTATAGACAG

ATGTTGGCAAATTAGTATAAATTAGTTATTATATTAGTTTTTAAGGTTCAATATACTTTTT

AAAACTCTTATTAAGTTGTTTAGCTTGCCAACCAAAGACTAAATTATCTTTATTTGTTAGG

AAATAAAAGCACTTATTTTATTTTCTCCATATTTGTTTCCTCTTTGTTCCATTACTAAAAA

AATTATAATATATTAATAACAAAAAAATTTGGGGAGGGAATGTTATTTTTAAAAAAGATGT

TTTATTTATATTTAAGTGTTTTATTGAAATTCCTCGTGAGTTTAGCTCAATTGGTATGGAC

AATACATAATATATGCAAAATTCGAGGTTCAAACCTTGAGCACCACAAAAAAAAGTGTTTT

ATTGAAAAATATCAAGAACTTGAAAACAAATTTCCTTTTTATGAACTAGATATTGTCACAT

AGACAACTTAAGAATTGACTTTTTTTTTAACACTATTGTATTTTCTGAACTCGACTTTAAC

TCGAGATCAATGAGTAAACTAAAAAAACTCGTATTATTTTATCTAAGTGTTCTTAGTGTGA

GAAAACATTATAATTGTTTGCTTAGTCTATTTCTCCCTATATTCTCACCCTCATTCTTGGT

TTGCACATAGAAAGGAGAAAAAAAAAATTAGAGAAGAGTTGAATGGAACAGAAAGAAAGAG

CAAGCAAAATAATCACTAAAAAAAATTCCCCTAAAAGAAAATCACTGAAAAATATTATCCA

CCTCTATTTTAAATAATAATAATAATGAAAACAACAATATATTTTTTTATAGATTTTCTCT

ACTAGAGGTAATTTATTTTAAACACTAAATGTCCACACCTCTAACAAAAAAAATGCATAAT

ATTATAATATATTATTGTAAGAAAGAGTGACAAATGTTGAATTAGTTATGGAATGAAGAGT

GAGAGAGATTATACAAATAAATAAATATAAATATAAATATAAATAAAGAATAGAGAAGAAC

ATAGTACTTGAATGAAGAGATTTGGCTTGTACTTCCATTACACTTGTAAGCTTTCTACTTC

CTTCACATTCCTTCTCTTTCTCTATCTTTCCTTTTATGATATGTGTACTTTGTTATTGCTT

CATTTACTACCGACATGACTCCAACCTATTAGGGCTTTATCTGAATAATGTTAGTTATTTT

CTCTCATTTTCTTTGCTTCTTCTTATTCTTGGAACTTATAAAGGGTCTTGTCAATTCTATC

GTTCTCTTTTGTATACTTACAAATATAAATATTCTCTTTAGCTTCATTTTCATGTCTTCTT

CTACTAATAAGCTATGGAAATTGAAGTATTTGTTTTCATGTTTATTTTTCACTTCAGCTCC

TCTTTTTTGATGTTGATGCTGAAGATTGAATAAAAACTGAGGTGGTATGTATGAGAACAAG

AACATGTTTCATTCATCTCTGTTATTTGAACATCTAAATTTTAAACAACTATCGCGACAAT

TTTTTAAGTGGTTGAAGTATTTTAAATAGGTGGTTAATTACATTAAGTAGATAGTTAATTG

ATATATCAGGTGTCAAAACTCATTAACCATCAACATAACTTCATTAGCCATAAATTTTTGA

CGTGATTAACCAAATATTGTAGATGCTCAATTACATGTAGATTTGATGGAGTTGTCCACAA

TTTCTCATATATATATTAACTAATAACCATCTACCGAATATAATTAACCATATATTAGAAT

ATCATTAACCTCTAAAACAGTTGTCGCGAAAATTGGTTGTAAAAAAATCATTTCTATTTTT

TACGCCACTAGTTCTCTTGTTATTTCTCTTTGTTGGAAAGTTGTTGTTTTTAACTTTGAGC

TTAGTCTTATTAATTAACTTGTAATGGTAACAACATTTTTCTATCTTTGTTTGAAGGAAGT

AGTTACTGTGTCAACTTTAGTAATTAATTTGGATCATTTTGGAGGCAAAATATGGCTATGC

AACCTGTTTATTTTAAAGAACATGAAGGAAATGTCCACAATTCTGTTGGACAGTTTTCATC

TGTGACTTCAGCACCATGGTGGAGTAATGCCTATGGATCTCAACCTTTTTATGGAGGAGAC

TCTTGTGGCCAAATGAAACCTTTTTCACTAGAGCTTTCCAACTACATAGACCAACTTGCTC

CGAGTAAGAACTTAGTTCGAGGAGTTGAACAATTGTTTGATAAAGGGCATACAAACCAATT

CACCATCTTTCCAGGTACTTGTTATTCAATATAATTCCGGTTTTGAATGAATTGATTTTTG

TTAAGTTGGTTCTGGATAAACGTGCGTTAAATATAATATGATTTATGTTTGGATACGTTTA

TGTTAAAGTGAGTTGAACATAAATTGGAGACTAAATATCAATTGCAGAGGCAAAAGCTTCA

AATTCTAATTTCAAGTTAGAATCAAATCAATTCTACTCGTAAGCATCTAAATGTTTCAAAA

CCAATTCTACATCTCTAGAACTAAATTTGCATTCAAACATTTTCTGCTACAGATGATTGTA

AGATGTCAGTTGATACACCAAATCATCAAGCAACCTTATCCCTGCAATCACCATTTGCTGC

CGAGCCACTTAATCGATTTGAGCTAGGTTTTAACCAGTCTATGGTAAATCTTCTTATTAAA

GCAATCCCTTTGATCATGTTTTAGTATTTTACTTTTGATGTACATAAATTCATTATGTTTT

TATTATCCTTTCCATGCTAGATCTGTGCAAAATATCCGTATATAGATCAATTTTACGGGCT

CTTCTCGACTTATGGACCTCAAATCTCGGTTTGTCTTCACCTCGGCTATTTTATTAATCTT

TGTATATATCATGCAATGTCTATAAGTACTTGAATGTGAATTTGTTTTATTTACAGTGTGT

ATGCTTACATGAGAAAGCCAGAAATCTGTTTACAACTATCTTGTTAGTATTCTTCTAAATC

TTTCAAATGTAGAGTTGATTATGAAATTTGTTGTTAATGAGTTTTCGGCTTTATGAATGAA

TTGTAAGACAAGTAATGAAACAAAAACTAGAAGAAAATTGAATTATATGATGCAATCTGTG

TTGAAGTTTTTGCATGTATTCAAGTTCTGCTTTTAGTTACTATTTGTTTATCTGCTGCAAT

ATTTCATCAATTATTATTCATGTATGTACCAATATTTAATAAATTAGGGGCGAATTATGCT

TCCGCTTAGCATGACATCTGACGACGGACCAACATACGTGAATGCTAAGCAATACCATGGA

ATCATCAGACGCAGGCATTCTCGTGCCAAAGCTGTGCTTCAGAATAAATTGATAAAGCGTA

ACAAGGTATGAAACTGAGTATTTTTCCTCACAACAATGTTCGAAAACTTGTGAAATAGTAT

ATTTTTCCTTTTACCCTTTTTATACCTATATTAAGGTTTTGTATTTGTCTTGCAGCCATAT

ATGCACGAATCGCGTCATCTACATGCAATGCGTAGACCAAGAGGATGCGGTGGTCGTTTCT

TGAACACAAAAGTTTCTGCTAATGGAAACGGTAAAAGTGGGAGTGAAGAGAACGGAAACAT

TGGTGGCCTACAGCTGCAGTCCAGTGGTTCTCAGAGTTCCGAAGTCTTACAATCTGAGGTT

GGAACTTTAAATTCGTCGAAGGAGACAAACGGAGGCAGTCCAAATGTCTCGGGGTCAGAGG

TGACTAGCATGTATACACAGGGAGGTCTTGATAGCTTTACTGTCAATCATATCGGATCTAC

TGTCCACTCTTTGGGAGACATGATCGATACTGGACACGGTATCGTCATGCCAACAAAATGG

TTTGCAGCAGCTGGCAGACAGCTGCTGGAACCATAAGTTTCGATTCAGAAAGGAAACAAGT

GGGTTTGGTACAATGTGAAATATTTTGCACCAAACTCATCCTTTCCGAGACCAGATGAAGA

AGCTATGTTTCAGTTTGTTGTGTTTACTACGACAAATTTAGTTTTGGAAGACTACTTTTCC

ATCTGGTGCTCAGGCAACTCATTCTTGGCTTATTCTCAGGAAACTCATCCTTGGCTCGTAA

TATTTAGTAGTATTGTCATCGTCTTCCCGCGCAGGCTTTCCGTGGCATGGTAGGCATGCTA

ATGACTTTGGTATTTTCATGCAGTTATAACTATGATGTGTCTTTGTTTGTTGTTAAAATAA

AAAACATGAACTCTAGCTAGGTGCATGTGTGTGTTTTTAATCTTGTCTACTAAGTTTGGTG

TTTTGTAATGGATTTCTGACTTTATGGAGCAATGTATTGTAACTCTACTAAGAAGTGTAAC

ATTTTATTTCTCCCTCTCTAAGGATTGTATAAGAACCTCTTATTTTCAGACTCTACTTAAT

CCTATTTTCTATGTCTGTATGATTTTTATATTTCTAGGACAATCAAATTGGCTTGTAGAAG

CTCAAAAGCATGCTCACAAAGTAGGTACTTATGTAGGGAACTTCGTACTCTAATAATAACT

GGTTATTAACGTTATAATTAAATGCAAAATTTGATAAGTAGTAGGGTTTGGTAAGTAATAT

AACAACACCATGTTGGCTTGGGATTCTGAATTGTGTTATTAGAGTACAACTTTTAAATAAT

GATCGTAATGTAAATAATTTAAATTTGAAGTTACTAGATAAAAAGATAATATTATGTAATA

TATTATCAAATATGTACTAAAAATGTAATTTTCATCTATCTCAATTGAAAAAATATCTGAG

TTTTGAGTCTATTGTCATGGACGA

>HM011

AAAAAAATAAAGGTTCTATACTCATTAAACAAAGTGCCAAATTCAGAGATACTACTTCCT

TTGGATAATTTGTATCGTCACCAACGATCTTTGAGTTATTTCAAAAATCACATTCTCAATT

TTGTGGTCCAACATTCACAAAATGGTTTGTCGGGCTTTAAGTCTAGTAATTGTGAAACATG

CATATGCATGAGGTAATTAATTGATAATCTGAAAAGGTTAAATAGTTAATCAATTGTACTT

TGGTTCTTTTTAATTTGTTCTCTATAATTTTTCAGTCAAAATTGAATTTGCTTTAACCATT

TAGTGTAGTAGTATGATATTACTTGCTCTACATTCTTGGATCTAAAGGTCTAATTAGTATC

GTATAAAAAATATTCTAGTTAATTAAGATACTTTTGTTCATTCTAATTATTCTTGGATTAC

TTGTGAAACTTTTAGGGATGAACAAGAAAGTCCACTAAAGAGGCTTCTTTTTGCTAGGAGT

CACTAGTTAGTTTTTTTTTTTTTTGACAAAAGTTAACCATTTTTTTTTATCGCATGGTAGG

AAAAAAAAAATAGAGATTTCCTTTGGTACTCATAAAAAAAAAAGTTGGATGGATAATGGAT

TAATCATAACTTATTTAACATAAAATGAACTAAGAGAACATTTCATATTTGGGTTTATTTT

GTAACATAAATATTAATATCAATGTTTGAAAGAGTTTTTAACAATAATTTGTAAAAGAAAG

CATAATATATCTATCAAAATAATTATAAAAGAAAAAATTTCTTTTGGTGGTATGACATGGA

AATATAATTAATTCTTATTAGATGAAAGTGTAAAATTAATTTGCATTAACAAAGCATATTT

CATTAAACTCCTTTTAGTTTTTGTTGGAAAAAATAAATTTATTGTTACTCAAAAAATAAAA

GTGTACAATTAATATATTTACTCAAAAGTCCCCTAAATGGGATGTAATTATCAAGGGATGT

GAGCGGTTTAACTTTAAGTAATGAATCAACTGTTGCATAATCACTTTGAAAGAAACTTGAG

ATTCTTCAATTAGCAAAATCTTGAATAGAATAATGAGATTATTCCTAATGCTAATGATACC

CACCTCTCCTATAAGTTGATGGGGCAGAACAAATTATGCACTCAGGACAACTTTTCTCAAA

TTTTGAAAACTTACCACACGAAGAACACACTTCCACTTGCACAAGTAAGAATATAGTATAA

CAATAAATTTACCACTCATAGCTCAATGGATATTAAAATATTGCTAATCTTTTATAGACAG

ATGTTGGCAAATTAGTATAAATTAGTTATTATATTAGTTTTTAAGGTTCAATATACTTTTT

AAAACTCTTATTAAGTTGTTTAGCTTGCCAACCAAAGACTAAATTATCTTTATTTGTTAGG

AAATAAAAGCACTTATTTTATTTTCTCCATATTTGTTTCCTCTTTGTTCCATTACTAAAAA

AATTATAATATATTAATAACAAAAAAATTTGGGGAGGGAATGTTATTTTTAAAAAAGATGT

TTTATTTATATTTAAGTGTTTTATTGAAATTCCTCGTGAGTTTAGCTCAATTGGTATGGAC

AATACATAATATATGCAAAATTCGAGGTTCAAACCTTGAGCACCACAAAAAAAAGTGTTTT

ATTGAAAAATATCAAGAACTTGAAAACAAATTTCCTTTTTATGAACTAGATATTGTCACAT

AGACAACTTAAGAATTGACTTTTTTTTTAACACTATTGTATTTTCTGAACTCGACTTTAAC

TCGAGATCAATGAGTAAACTAAAAAAACTCGTATTATTTTATCTAAGTGTTCTTAGTGTGA

GAAAACATTATAATTGTTTGCTTAGTCTATTTCTCCCTATATTCTCACCCTCATTCTTGGT

TTGCACATAGAAAGGAGAAAAAAAAAATTAGAGAAGAGTTGAATGGAACAGAAAGAAAGAG

CAAGCAAAATAATCACTAAAAAAAATTCCCCTAAAAGAAAATCACTGAAAAATATTATCCA

CCTCTATTTTAAATAATAATAATAATGAAAACAACAATATATTTTTTTATAGATTTTCTCT

ACTAGAGGTAATTTATTTTAAACACTAAATGTCCACACCTCTAACAAAAAAAATGCATAAT

ATTATAATATATTATTGTAAGAAAGAGTGACAAATGTTGAATTAGTTATGGAATGAAGAGT

GAGAGAGATTATACAAATAAATAAATATAAATATAAATATAAATAAAGAATAGAGAAGAAC

ATAGTACTTGAATGAAGAGATTTGGCTTGTACTTCCATTACACTTGTAAGCTTTCTACTTC

CTTCACATTCCTTCTCTTTCTCTATCTTTCCTTTTATGATATGTGTACTTTGTTATTGCTT

CATTTACTACCGACATGACTCCAACCTATTAGGGCTTTATCTGAATAATGTTAGTTATTTT

CTCTCATTTTCTTTGCTTCTTCTTATTCTTGGAACTTATAAAGGGTCTTGTCAATTCTATC

GTTCTCTTTTGTATACTTACAAATATAAATATTCTCTTTAGCTTCATTTTCATGTCTTCTT

CTACTAATAAGCTATGGAAATTGAAGTATTTGTTTTCATGTTTATTTTTCACTTCAGCTCC

TCTTTTTTGATGTTGATGCTGAAGATTGAATAAAAACTGAGGTGGTATGTATGAGAACAAG

AACATGTTTCATTCATCTCTGTTATTTGAACATCTAAATTTTAAACAACTATCGCGACAAT

TTTTTAAGTGGTTGAAGTATTTTAAATAGGTGGTTAATTACATTAAGTAGATAGTTAATTG

ATATATCAGGTGTCAAAACTCATTAACCATCAACATAACTTCATTAGCCATAAATTTTTGA

CGTGATTAACCAAATATTGTAGATGCTCAATTACATGTAGATTTGATGGAGTTGTCCACAA

TTTCTCATATATATATTAACTAATAACCATCTACCGAATATAATTAACCATATATTAGAAT

ATCATTAACCTCTAAAACAGTTGTCGCGAAAATTGGTTGTAAAAAAATCATTTCTATTTTT

TACGCCACTAGTTCTCTTGTTATTTCTCTTTGTTGGAAAGTTGTTGTTTTTAACTTTGAGC

TTAGTCTTATTAATTAACTTGTAATGGTAACAACATTTTTCTATCTTTGTTTGAAGGAAGT

AGTTACTGTGTCAACTTTAGTAATTAATTTGGATCATTTTGGAGGCAAAATATGGCTATGC

AACCTGTTTATTTTAAAGAACATGAAGGAAATGTCCACAATTCTGTTGGACAGTTTTCATC

TGTGACTTCAGCACCATGGTGGAGTAATGCCTATGGATCTCAACCTTTTTATGGAGGAGAC

TCTTGTGGCCAAATGAAACCTTTTTCACTAGAGCTTTCCAACTACATAGACCAACTTGCTC

CGAGTAAGAACTTAGTTCGAGGAGTTGAACAATTGTTTGATAAAGGGCATACAAACCAATT

CACCATCTTTCCAGGTACTTGTTATTCAATATAATTCCGGTTTTGAATGAATTGATTTTTG

TTAAGTTGGTTCTGGATAAACGTGCGTTAAATATAATATGATTTATGTTTGGATACGTTTA

TGTTAAAGTGAGTTGAACATAAATTGGAGACTAAATATCAATTGCAGAGGCAAAAGCTTCA

AATTCTAATTTCAAGTTAGAATCAAATCAATTCTACTCGTAAGCATCTAAATGTTTCAAAA

CCAATTCTACATCTCTAGAACTAAATTTGCATTCAAACATTTTCTGCTACAGATGATTGTA

AGATGTCAGTTGATACACCAAATCATCAAGCAACCTTATCCCTGCAATCACCATTTGCTGC

CGAGCCACTTAATCGATTTGAGCTAGGTTTTAACCAGTCTATGGTAAATCTTCTTATTAAA

GCAATCCCTTTGATCATGTTTTAGTATTTTACTTTTGATGTACATAAATTCATTATGTTTT

TATTATCCTTTCCATGCTAGATCTGTGCAAAATATCCGTATATAGATCAATTTTACGGGCT

CTTCTCGACTTATGGACCTCAAATCTCGGTTTGTCTTCACCTCGGCTATTTTATTAATCTT

TGTATATATCATGCAATGTCTATAAGTACTTGAATGTGAATTTGTTTTATTTACAGTGTGT

ATGCTTACATGAGAAAGCCAGAAATCTGTTTACAACTATCTTGTTAGTATTCTTCTAAATC

TTTCAAATGTAGAGTTGATTATGAAATTTGTTGTTAATGAGTTTTCGGCTTTATGAATGAA

TTGTAAGACAAGTAATGAAACAAAAACTAGAAGAAAATTGAATTATATGATGCAATCTGTG

TTGAAGTTTTTGCATGTATTCAAGTTCTGCTTTTAGTTACTATTTGTTTATCTGCTGCAAT

ATTTCATCAATTATTATTCATGTATGTACCAATATTTAATAAATTAGGGGCGAATTATGCT

TCCGCTTAGCATGACATCTGACGACGGACCAACATACGTGAATGCTAAGCAATACCATGGA

ATCATCAGACGCAGGCATTCTCGTGCCAAAGCTGTGCTTCAGAATAAATTGATAAAGCGTA

ACAAGGTATGAAACTGAGTATTTTTCCTCACAACAATGTTCGAAAACTTGTGAAATAGTAT

ATTTTTCCTTTTACCCTTTTTATACCTATATTAAGGTTTTGTATTTGTCTTGCAGCCATAT

ATGCACGAATCGCGTCATCTACATGCAATGCGTAGACCAAGAGGATGCGGTGGTCGTTTCT

TGAACACAAAAGTTTCTGCTAATGGAAACGGTAAAAGTGGGAGTGAAGAGAACGGAAACAT

TGGTGGCCTACAGCTGCAGTCCAGTGGTTCTCAGAGTTCCGAAGTCTTACAATCTGAGGTT

GGAACTTTAAATTCGTCGAAGGAGACAAACGGAGGCAGTCCAAATGTCTCGGGGTCAGAGG

TGACTAGCATGTATACACAGGGAGGTCTTGATAGCTTTACTGTCAATCATATCGGATCTAC

TGTCCACTCTTTGGGAGACATGATCGATACTGGACACGGTATCGTCATGCCAACAAAATGG

TTTGCAGCAGCTGGCAGACAGCTGCTGGAACCATAAGTTTCGATTCAGAAAGGAAACAAGT

GGGTTTGGTACAATGTGAAATATTTTGCACCAAACTCATCCTTTCCGAGACCAGATGAAGA

AGCTATGTTTCAGTTTGTTGTGTTTACTACGACAAATTTAGTTTTGGAAGACTACTTTTCC

ATCTGGTGCTCAGGCAACTCATTCTTGGCTTATTCTCAGGAAACTCATCCTTGGCTCGTAA

TATTTAGTAGTATTGTCATCGTCTTCCCGCGCAGGCTTTCCGTGGCATGGTAGGCATGCTA

ATGACTTTGGTATTTTCATGCAGTTATAACTATGATGTGTCTTTGTTTGTTGTTAAAATAA

AAAACATGAACTCTAGCTAGGTGCATGTGTGTGTTTTTAATCTTGTCTACTAAGTTTGGTG

TTTTGTAATGGATTTCTGACTTTATGGAGCAATGTATTGTAACTCTACTAAGAAGTGTAAC

ATTTTATTTCTCCCTCTCTAAGGATTGTATAAGAACCTCTTATTTTCAGACTCTACTTAAT

CCTATTTTCTATGTCTGTATGATTTTTATATTTCTAGGACAATCAAATTGGCTTGTAGAAG

CTCAAAAGCATGCTCACAAAGTAGGTACTTATGTAGGGAACTTCGTACTCTAATAATAACT

GGTTATTAACGTTATAATTAAATGCAAAATTTGATAAGTAGTAGGGTTTGGTAAGTAATAT

AACAACACCATGTTGGCTTGGGATTCTGAATTGTGTTATTAGAGTACAACTTTTAAATAAT

GATCGTAATGTAAATAATTTAAATTTGAAGTTACTAGATAAAAAGATAATATTATGTAATA

TATTATCAAATATGTACTAAAAATGTAATTTTCATCTATCTCAATTGAAAAAATATCTGAG

TTTTGAGTCTATTGTCATGGACGA

>HM012

AAAAAAATAAAGGTTCTATACTCATTAAACAAAGTGCCAAATTCAGAGATACTACTTCCT

TTGGATAATTTGTATCGTCACCAACGATCTTTGAGTTATTTCAAAAATCACATTCTCAATT

TTGTGGTCCAACATTCACAAAATGGTTTGTCGGGCTTTAAGTCTAGTAATTGTGAAACATG

CATATGCATGAGGTAATTAATTGATAATCTGAAAAGGTTAAATAGTTAATCAATTGTACTT

TGGTTCTTTTTAATTTGTTCTCTATAATTTTTCAGTCAAAATTGAATTTGCTTTAACCATT

TAGTGTAGTAGTATGATATTACTTGCTCTACATTCTTGGATCTAAAGGTCTAATTAGTATC

GTATAAAAAATATTCTAGTTAATTAAGATACTTTTGTTCATTCTAATTATTCTTGGATTAC

TTGTGAAACTTTTAGGGATGAACAAGAAAGTCCACTAAAGAGGCTTCTTTTTGCTAGGAGT

CACTAGTTAGTTTTTTTTTTTTTTGACAAAAGTTAACCATTTTTTTTTATCGCATGGTAGG

AAAAAAAAAATAGAGATTTCCTTTGGTACTCATAAAAAAAAAAGTTGGATGGATAATGGAT

TAATCATAACTTATTTAACATAAAATGAACTAAGAGAACATTTCATATTTGGGTTTATTTT

GTAACATAAATATTAATATCAATGTTTGAAAGAGTTTTTAACAATAATTTGTAAAAGAAAG

CATAATATATCTATCAAAATAATTATAAAAGAAAAAATTTCTTTTGGTGGTATGACATGGA

AATATAATTAATTCTTATTAGATGAAAGTGTAAAATTAATTTGCATTAACAAAGCATATTT

CATTAAACTCCTTTTAGTTTTTGTTGGAAAAAATAAATTTATTGTTACTCAAAAAATAAAA

GTGTACAATTAATATATTTACTCAAAAGTCCCCTAAATGGGATGTAATTATCAAGGGATGT

GAGCGGTTTAACTTTAAGTAATGAATCAACTGTTGCATAATCACTTTGAAAGAAACTTGAG

ATTCTTCAATTAGCAAAATCTTGAATAGAATAATGAGATTATTCCTAATGCTAATGATACC

CACCTCTCCTATAAGTTGATGGGGCAGAACAAATTATGCACTCAGGACAACTTTTCTCAAA

TTTTGAAAACTTACCACACGAAGAACACACTTCCACTTGCACAAGTAAGAATATAGTATAA

CAATAAATTTACCACTCATAGCTCAATGGATATTAAAATATTGCTAATCTTTTATAGACAG

ATGTTGGCAAATTAGTATAAATTAGTTATTATATTAGTTTTTAAGGTTCAATATACTTTTT

AAAACTCTTATTAAGTTGTTTAGCTTGCCAACCAAAGACTAAATTATCTTTATTTGTTAGG

AAATAAAAGCACTTATTTTATTTTCTCCATATTTGTTTCCTCTTTGTTCCATTACTAAAAA

AATTATAATATATTAATAACAAAAAAATTTGGGGAGGGAATGTTATTTTTAAAAAAGATGT

TTTATTTATATTTAAGTGTTTTATTGAAATTCCTCGTGAGTTTAGCTCAATTGGTATGGAC

AATACATAATATATGCAAAATTCGAGGTTCAAACCTTGAGCACCACAAAAAAAAGTGTTTT

ATTGAAAAATATCAAGAACTTGAAAACAAATTTCCTTTTTATGAACTAGATATTGTCACAT

AGACAACTTAAGAATTGACTTTTTTTTTAACACTATTGTATTTTCTGAACTCGACTTTAAC

TCGAGATCAATGAGTAAACTAAAAAAACTCGTATTATTTTATCTAAGTGTTCTTAGTGTGA

GAAAACATTATAATTGTTTGCTTAGTCTATTTCTCCCTATATTCTCACCCTCATTCTTGGT

TTGCACATAGAAAGGAGAAAAAAAAAATTAGAGAAGAGTTGAATGGAACAGAAAGAAAGAG

CAAGCAAAATAATCACTAAAAAAAATTCCCCTAAAAGAAAATCACTGAAAAATATTATCCA

CCTCTATTTTAAATAATAATAATAATGAAAACAACAATATATTTTTTTATAGATTTTCTCT

ACTAGAGGTAATTTATTTTAAACACTAAATGTCCACACCTCTAACAAAAAAAATGCATAAT

ATTATAATAGATTATTGTAAGAAAGAGTGACAAATGTTGAATTAGTTATGGAATGAAGAGT

GAGAGAGATTATACAAATAAATAAATATAAATATAAATATAAATAAAGAATAGAGAAGAAC

ATAGTACTTGAATGAAGAGATTTGGCTTGTACTTCCATTACACTTGTAAGCTTTCTACTTC

CTTCACATTCCTTCTCTTTCTCTATCTTTCCTTTTATGATATGTGTACTTTGTTATTGCTT

CATTTACTACCGACATGACTCCAACCTATTAGGGCTTTATCTGAATAATGTTAGTTATTTT

CTCTCATTTTCTTTGCTTCTTCTTATTCTTGGAACTTATAAAGGGTCTTGTCAATTCTATC

GTTCTCTTTTGTATACTTACAAATATAAATATTCTCTTTAGCTTCATTTTCATGTCTTCTT

CTACTAATAAGCTATGGAAATTGAAGTATTTGTTTTCATGTTTATTTTTCACTTCAGCTCC

TCTTTTTTGATGTTGATGCTGAAGATTGAATAAAAACTGAGGTGGTATGTATGAGAACAAG

AACATGTTTCATTCATCTCTGTTATTTGAACATCTAAATTTTAAACAACTATCGCGACAAT

TTTTTAAGTGGTTGAAGTATTTTAAATAGGTGGTTAATTACATTAAGTAGATAGTTAATTG

ATATATCAGGTGTCAAAACTCATTAACCATCAACATAACTTCATTAGCCATAAATTTTTGA

CGTGATTAACCAAATATTGTAGATGCTCAATTACATGTAGATTTGATGGAGTTGTCCACAA

TTTCTCATATATATATTAACTAATAACCATCTACCGAATATAATTAACCATATATTAGAAT

ATCATTAACCTCTAAAACAGTTGTCGCGAAAATTGGTTGTAAAAAAATCATTTCTATTTTT

TACGCCACTAGTTCTCTTGTTATTTCTCTTTGTTGGAAAGTTGTTGTTTTTAACTTTGAGC

TTAGTCTTATTAATTAACTTGTAATGGTAACAACATTTTTCTATCTTTGTTTGAAGGAAGT

AGTTACTGTGTCAACTTTAGTAATTAATTTGGATCATTTTGGAGGCAAAATATGGCTATGC

AACCTGTTTATTTTAAAGAACATGAAGGAAATGTCCACAATTCTGTTGGACAGTTTTCATC

TGTGACTTCAGCACCATGGTGGAGTAATGCCTATGGATCTCAACCTTTTTATGGAGGAGAC

TCTTGTGGCCAAATGAAACCTTTTTCACTAGAGCTTTCCAACTACATAGACCAACTTGCTC

CGAGTAAGAACTTAGTTCGAGGAGTTGAACAATTGTTTGATAAAGGGCATACAAACCAATT

CACCATCTTTCCAGGTACTTGTTATTCAATATAATTCCGGTTTTGAATGAATTGATTTTTG

TTAAGTTGGTTCTGGATAAACGTGCGTTAAATATAATATGATTTATGTTTGGATACGTTTA

TGTTAAAGTGAGTTGAACATAAATTGGAGACTAAATATCAATTGCAGAGGCAAAAGCTTCA

AATTCTAATTTCAAGTTAGAATCAAATCAATTCTACTCGTAAGCATCTAAATGTTTCAAAA

CCAATTCTACATCTCTAGAACTAAATTTGCATTCAAACATTTTCTGCTACAGATGATTGTA

AGATGTCAGTTGATACACCAAATCATCAAGCAACCTTATCCCTGCAATCACCATTTGCTGC

CGAGCCACTTAATCGATTTGAGCTAGGTTTTAACCAGTCTATGGTAAATCTTCTTATTAAA

GCAATCCCTTTGATCATGTTTTAGTATTTTACTTTTGATGTACATAAATTCATTATGTTTT

TATTATCCTTTCCATGCTAGATCTGTGCAAAATATCCGTATATAGATCAATTTTACGGGCT

CTTCTCGACTTATGGACCTCAAATCTCGGTTTGTCTTCACCTCGGCTATTTTATTAATCTT

TGTATATATCATGCAATGTCTATAAGTACTTGAATGTGAATTTGTTTTATTTACAGTGTGT

ATGCTTACATGAGAAAGCCAGAAATCTGTTTACAACTATCTTGTTAGTATTCTTCTAAATC

TTTCAAATGTAGAGTTGATTATGAAATTTGTTGTTAATGAGTTTTCGGCTTTATGAATGAA

TTGTAAGACAAGTAATGAAACAAAAACTAGAAGAAAATTGAATTATATGATGCAATCTGTG

TTGAAGTTTTTGCATGTATTCAAGTTCTGCTTTTAGTTACTATTTGTTTATCTGCTGCAAT

ATTTCATCAATTATTATTCATGTATGTACCAATATTTAATAAATTAGGGGCGAATTATGCT

TCCGCTTAGCATGACATCTGACGACGGACCAACATACGTGAATGCTAAGCAATACCATGGA

ATCATCAGACGCAGGCATTCTCGTGCCAAAGCTGTGCTTCAGAATAAATTGATAAAGCGTA

ACAAGGTATGAAACTGAGTATTTTTCCTCACAACAATGTTCGAAAACTTGTGAAATAGTAT

ATTTTTCCTTTTACCCTTTTTATACCTATATTAAGGTTTTGTATTTGTCTTGCAGCCATAT

ATGCACGAATCGCGTCATCTACATGCAATGCGTAGACCAAGAGGATGCGGTGGTCGTTTCT

TGAACACAAAAGTTTCTGCTAATGGAAACGGTAAAAGTGGGAGTGAAGAGAACGGAAACAT

TGGTGGCCTACAGCTGCAGTCCAGTGGTTCTCAGAGTTCCGAAGTCTTACAATCTGAGGTT

GGAACTTTAAATTCGTCGAAGGAGACAAACGGAGGCAGTCCAAATGTCTCGGGGTCAGAGG

TGACTAGCATGTATACACAGGGAGGTCTTGATAGCTTTACTGTCAATCATATCGGATCTAC

TGTCCACTCTTTGGGAGACATGATCGATACTGGACACGGTATCGTCATGCCAACAAAATGG

TTTGCAGCAGCTGGCAGACAGCTGCTGGAACCATAAGTTTCGATTCAGAAAGGAAACAAGT

GGGTTTGGTACAATGTGAAATATTTTGCACCAAACTCATCCTTTCCGAGACCAGATGAAGA

AGCTATGTTTCAGTTTGTTGTGTTTACTACGACAAATTTAGTTTTGGAAGACTACTTTTCC

ATCTGGTGCTCAGGCAACTCATTCTTGGCTTATTCTCAGGAAACTCATCCTTGGCTCGTAA

TATTTAGTAGTATTGTCATCGTCTTCCCGCGCAGGCTTTCCGTGGCATGGTAGGCATGCTA

ATGACTTTGGTATTTTCATGCAGTTATAACTATGATGTGTCTTTGTTTGTTGTTAAAATAA

AAAACATGAACTCTAGCTAGGTGCATGTGTGTGTTTTTAATCTTGTCTACTAAGTTTGGTG

TTTTGTAATGGATTTCTGACTTTATGGAGCAATGTATTGTAACTCTACTAAGAAGTGTAAC

ATTTTATTTCTCCCTCTCTAAGGATTGTATAAGAACCTCTTATTTTCAGACTCTACTTAAT

CCTATTTTCTATGTCTGTATGATTTTTATATTTCTAGGACAATCAAATTGGCTTGTAGAAG

CTCAAAAGCATGCTCACAAAGTAGGTACTTATGTAGGGAACTTCGTACTCTAATAATAACT

GGTTATTAACGTTATAATTAAATGCAAAATTTGATAAGTAGTAGGGTTTGGTAAGTAATAT

AACAACACCATGTTGGCTTGGGATTCTGAATTGTGTTATTAGAGTACAACTTTTAAATAAT

GATCGTAATGTAAATAATTTAAATTTGAAGTTACTAGATAAAAAGATAATATTATGTAATA

TATTATCAAATATGTACTAAAAATGTAATTTTCATCTATCTCAATTGAAAAAATATCTGAG

TTTTGAGTCTATTGTCATGGACGA

>HM013

AAAAAAATAAAGGTTCTATACTCATTAAACAAAGTGCCAAATTCAGAGATACTACTTCCT

TTGGATAATTTGTATCGTCACCAACGATCTTTGAGTTATTTCAAAAATCACATTCTCAATT

TTGTGGTCCAACATTCACAAAATGGTTTGTCGGGCTTTAAGTCTAGTAATTGTGAAACATG

CATATGCATGAGGTAATTAATTGATAATCTGAAAAGGTTAAATAGTTAATCAATTGTACTT

TGGTTCTTTTTAATTTGTTCTCTATAATTTTTCAGTCAAAATTGAATTTGCTTTAACCATT

TAGTGTAGTAGTATGATATTACTTGCTCTACATTCTTGGATCTAAAGGTCTAATTAGTATC

GTATAAAAAATATTCTAGTTAATTAAGATACTTTTGTTCATTCTAATTATTCTTGGATTAC

TTGTGAAACTTTTAGGGATGAACAAGAAAGTCCACTAAAGAGGCTTCTTTTTGCTAGGAGT

CACTAGTTAGTTTTTTTTTTTTTTGACAAAAGTTAACCATTTTTTTTTATCGCATGGTAGG

AAAAAAAAAATAGAGATTTCCTTTGGTACTCATAAAAAAAAAAGTTGGATGGATAATGGAT

TAATCATAACTTATTTAACATAAAATGAACTAAGAGAACATTTCATATTTGGGTTTATTTT

GTAACATAAATATTAATATCAATGTTTGAAAGAGTTTTTAACAATAATTTGTAAAAGAAAG

CATAATATATCTATCAAAATAATTATAAAAGAAAAAATTTCTTTTGGTGGTATGACATGGA

AATATAATTAATTCTTATTAGATGAAAGTGTAAAATTAATTTGCATTAACAAAGCATATTT

CATTAAACTCCTTTTAGTTTTTGTTGGAAAAAATAAATTTATTGTTACTCAAAAAATAAAA

GTGTACAATTAATATATTTACTCAAAAGTCCCCTAAATGGGATGTAATTATCAAGGGATGT

GAGCGGTTTAACTTTAAGTAATGAATCAACTGTTGCATAATCACTTTGAAAGAAACTTGAG

ATTCTTCAATTAGCAAAATCTTGAATAGAATAATGAGATTATTCCTAATGCTAATGATACC

CACCTCTCCTATAAGTTGATGGGGCAGAACAAATTATGCACTCAGGACAACTTTTCTCAAA

TTTTGAAAACTTACCACACGAAGAACACACTTCCACTTGCACAAGTAAGAATATAGTATAA

CAATAAATTTACCACTCATAGCTCAATGGATATTAAAATATTGCTAATCTTTTATAGACAG

ATGTTGGCAAATTAGTATAAATTAGTTATTATATTAGTTTTTAAGGTTCAATATACTTTTT

AAAACTCTTATTAAGTTGTTTAGCTTGCCAACCAAAGACTAAATTATCTTTATTTGTTAGG

AAATAAAAGCACTTATTTTATTTTCTCCATATTTGTTTCCTCTTTGTTCCATTACTAAAAA

AATTATAATATATTAATAACAAAAAAATTTGGGGAGGGAATGTTATTTTTAAAAAAGATGT

TTTATTTATATTTAAGTGTTTTATTGAAATTCCTCGTGAGTTTAGCTCAATTGGTATGGAC

AATACATAATATATGCAAAATTCGAGGTTCAAACCTTGAGCACCACAAAAAAAAGTGTTTT

ATTGAAAAATATCAAGAACTTGAAAACAAATTTCCTTTTTATGAACTAGATATTGTCACAT

AGACAACTTAAGAATTGACTTTTTTTTTAACACTATTGTATTTTCTGAACTCGACTTTAAC

TCGAGATCAATGAGTAAACTAAAAAAACTCGTATTATTTTATCTAAGTGTTCTTAGTGTGA

GAAAACATTATAATTGTTTGCTTAGTCTATTTCTCCCTATATTCTCACCCTCATTCTTGGT

TTGCACATAGAAAGGAGAAAAAAAAAATTAGAGAAGAGTTGAATGGAACAGAAAGAAAGAG

CAAGCAAAATAATCACTAAAAAAAATTCCCCTAAAAGAAAATCACTGAAAAATATTATCCA

CCTCTATTTTAAATAATAATAATAATGAAAACAACAATATATTTTTTTATAGATTTTCTCT

ACTAGAGGTAATTTATTTTAAACACTAAATGTCCACACCTCTAACAAAAAAAATGCATAAT

ATTATAATAGATTATTGTAAGAAAGAGTGACAAATGTTGAATTAGTTATGGAATGAAGAGT

GAGAGAGATTATACAAATAAATAAATATAAATATAAATATAAATAAAGAATAGAGAAGAAC

ATAGTACTTGAATGAAGAGATTTGGCTTGTACTTCCATTACACTTGTAAGCTTTCTACTTC

CTTCACATTCCTTCTCTTTCTCTATCTTTCCTTTTATGATATGTGTACTTTGTTATTGCTT

CATTTACTACCGACATGACTCCAACCTATTAGGGCTTTATCTGAATAATGTTAGTTATTTT

CTCTCATTTTCTTTGCTTCTTCTTATTCTTGGAACTTATAAAGGGTCTTGTCAATTCTATC

GTTCTCTTTTGTATACTTACAAATATAAATATTCTCTTTAGCTTCATTTTCATGTCTTCTT

CTACTAATAAGCTATGGAAATTGAAGTTTTTGTTTTCATGTTTATTTTTCACTTCAGCTCC

TCTTTTTTGATGTTGATGCTGAAGATTGAATAAAAACTGAGGTGGTATGTATGAGAACAAG

AACATGTTTCATTCATCTCTGTTATTTGAACATCTAAATTTTAAACAACTATCGCGACAAT

TTTTTAAGTGGTTGAAGTATTTTAAATAGGTGGTTAATTACATTAAGTAGATAGTTAATTG

ATATATCAGGTGTCAAAACTCATTAACCATCAACATAACTTCATTAGCCATAAATTTTTGA

CGTGATTAACCAAATATTGTAGATGCTCAATTACATGTAGATTTGATGGAGTTGTCCACAA

TTTCTCATATATATATTAACTAATAACCATCTACCGAATATAATTAACCATATATTAGAAT

ATCATTAACCTCTAAAACAGTTGTCGCGAAAATTGGTTGTAAAAAAATCATTTCTATTTTT

TACGCCACTAGTTCTCTTGTTATTTCTCTTTGTTGGAAAGTTGTTGTTTTTAACTTTGAGC

TTAGTCTTATTAATTAACTTGTAATGGTAACAACATTTTTCTATCTTTGTTTGAAGGAAGT

AGTTACTGTGTCAACTTTAGTAATTAATTTGGATCATTTTGGAGGCAAAATATGGCTATGC

AACCTGTTTATTTTAAAGAACATGAAGGAAATGTCCACAATTCTGTTGGACAGTTTTCATC

TGTGACTTCAGCACCATGGTGGAGTAATGCCTATGGATCTCAACCTTTTTATGGAGGAGAC

TCTTGTGGCCAAATGAAACCTTTTTCACTAGAGCTTTCCAACTACATAGACCAACTTGCTC

CGAGTAAGAACTTAGTTCGAGGAGTTGAACAATTGTTTGATAAAGGGCATACAAACCAATT

CACCATCTTTCCAGGTACTTGTTATTCAATATAATTCCGGTTTTGAATGAATTGATTTTTG

TTAAGTTGGTTCTGGATAAACGTGCGTTAAATATAATATGATTTATGTTTGGATACGTTTA

TGTTAAAGTGAGTTGAACATAAATTGGAGACTAAATATCAATTGCAGAGGCAAAAGCTTCA

AATTCTAATTTCAAGTTAGAATCAAATCAATTCTACTCGTAAGCATCTAAATGTTTCAAAA

CCAATTCTACATCTCTAGAACTAAATTTGCATTCAAACATTTTCTGCTACAGATGATTGTA

AGATGTCAGTTGATACACCAAATCATCAAGCAACCTTATCCCTGCAATCACCATTTGCTGC

CGAGCCACTTAATCGATTTGAGCTAGGTTTTAACCAGTCTATGGTAAATCTTCTTATTAAA

GCAATCCCTTTGATCATGTTTTAGTATTTTACTTTTGATGTACATAAATTCATTATGTTTT

TATTATCCTTTCCATGCTAGATCTGTGCAAAATATCCGTATATAGATCAATTTTACGGGCT

CTTCTCGACTTATGGACCTCAAATCTCGGTTTGTCTTCACCTCGGCTATTTTATTAATCTT

TGTATATATCATGCAATGTCTATAAGTACTTGAATGTGAATTTGTTTTATTTACAGTGTGT

ATGCTTACATGAGAAAGCCAGAAATCTGTTTACAACTATCTTGTTAGTATTCTTCTAAATC

TTTCAAATGTAGAGTTGATTATGAAATTTGTTGTTAATGAGTTTTCGGCTTTATGAATGAA

TTGTAAGACAAGTAATGAAACAAAAACTAGAAGAAAATTGAATTATATGATGCAATCTGTG

TTGAAGTTTTTGCATGTATTCAAGTTCTGCTTTTAGTTACTATTTGTTTATCTGCTGCAAT

ATTTCATCAATTATTATTCATGTATGTACCAATATTTAATAAATTAGGGGCGAATTATGCT

TCCGCTTAGCATGACATCTGACGACGGACCAACATACGTGAATGCTAAGCAATACCATGGA

ATCATCAGACGCAGGCATTCTCGTGCCAAAGCTGTGCTTCAGAATAAATTGATAAAGCGTA

ACAAGGTATGAAACTGAGTATTTTTCCTCACAACAATGTTCGAAAACTTGTGAAATAGTAT

ATTTTTCCTTTTACCCTTTTTATACCTATATTAAGGTTTTGTATTTGTCTTGCAGCCATAT

ATGCACGAATCGCGTCATCTACATGCAATGCGTAGACCAAGAGGATGCGGTGGTCGTTTCT

TGAACACAAAAGTTTCTGCTAATGGAAACGGTAAAAGTGGGAGTGAAGAGAACGGAAACAT

TGGTGGCCTACAGCTGCAGTCCAGTGGTTCTCAGAGTTCCGAAGTCTTACAATCTGAGGTT

GGAACTTTAAATTCGTCGAAGGAGACAAACGGAGGCAGTCCAAATGTCTCGGGGTCAGAGG

TGACTAGCATGTATACACAGGGAGGTCTTGATAGCTTTACTGTCAATCATATCGGATCTAC

TGTCCACTCTTTGGGAGACATGATCGATACTGGACACGGTATCGTCATGCCAACAAAATGG

TTTGCAGCAGCTGGCAGACAGCTGCTGGAACCATAAGTTTCGATTCAGAAAGGAAACAAGT

GGGTTTGGTACAATGTGAAATATTTTGCACCAAACTCATCCTTTCCGAGACCAGATGAAGA

AGCTATGTTTCAGTTTGTTGTGTTTACTACGACAAATTTAGTTTTGGAAGACTACTTTTCC

ATCTGGTGCTCAGGCAACTCATTCTTGGCTTATTCTCAGGAAACTCATCCTTGGCTCGTAA

TATTTAGTAGTATTGTCATTGTCTTCCCGCGCAGGCTTTCCGTGGCATGGTAGGCATGCTA

ATGACTTTGGTATTTTCATGCAGTTATAACTATGATGTGTCTTTGTTTGTTGTTAAAATAA

AAAACATGAACTCTAGCTAGGTGCATGTGTGTGTTTTTAATCTTGTCTACTAAGTTTGGTG

TTTTGTAATGGATTTCTGACTTTATGGAGCAATGTATTGTAACTCTACTAAGAAGTGTAAC

ATTTTATTTCTCCCTCTCTAAGGATTGTATAAGAACCTCTTATTTTCAGACTCTACTTAAT

CCTATTTTCTATGTCTGTATGATTTTTATATTTCTAGGACAATCAAATTGGCTTGTAGAAG

CTCAAAAGCATGCTCACAAAGTAGGTACTTATGTAGGGAACTTCGTACTCTAATAATAACT

GGTTATTAACGTTATAATTAAATGCAAAATTTGATAAGTAGTAGGGTTTGGTAAGTAATAT

AACAACACCATGTTGGCTTGGGATTCTGAATTGTGTTATTAGAGTACAACTTTTAAATAAT

GATCGTAATGTAAATAATTTAAATTTGAAGTTACTAGATAAAAAGATAATATTATGTAATA

TATTATCAAATATGTACTAAAAATGTAATTTTCATCTATCTCAATTGAAAAAATATCTGAG

TTTTGAGTCTATTGTCATGGACGA

>HM014

AAAAAAATAAAGGTTCTATACTCATTAAACAAAGTGCCAAATTCAGAGATACTACTTCCT

TTGGATAATTTGTATCGTCACCAACGATCTTTGAGTTATTTCAAAAATCACATTCTCAATT

TTGTGGTCCAACATTCACAAAATGGTTTGTCGGGCTTTAAGTCTAGTAATTGTGAAACATG

CATATGCATGAGGTAATTAATTGATAATCTGAAAAGGTTAAATAGTTAATCAATTGTACTT

TGGTTCTTTTTAATTTGTTCTCTATAATTTTTCAGTCAAAATTGAATTTGCTTTAACCATT

TAGTGTAGTAGTATGATATTACTTGCTCTACATTCTTGGATCTAAAGGTCTAATTAGTATC

GTATAAAAAATATTCTAGTTAATTAAGATACTTTTGTTCATTCTAATTATTCTTGGATTAC

TTGTGAAACTTTTAGGGATGAACAAGAAAGTCCACTAAAGAGGCTTCTTTTTGCTAGGAGT

CACTAGTTAGTTTTTTTTTTTTTTGACAAAAGTTAACCATTTTTTTTTATCGCATGGTAGG

AAAAAAAAAATAGAGATTTCCTTTGGTACTCATAAAAAAAAAAGTTGGATGGATAATGGAT

TAATCATAACTTATTTAACATAAAATGAACTAAGAGAACATTTCATATTTGGGTTTATTTT

GTAACATAAATATTAATATCAATGTTTGAAAGAGTTTTTAACAATAATTTGTAAAAGAAAG

CATAATATATCTATCAAAATAATTATAAAAGAAAAAATTTCTTTTGGTGGTATGACATGGA

AATATAATTAATTCTTATTAGATGAAAGTGTAAAATTAATTTGCATTAACAAAGCATATTT

CATTAAACTCCTTTTAGTTTTTGTTGGAAAAAATAAATTTATTGTTACTCAAAAAATAAAA

GTGTACAATTAATATATTTACTCAAAAGTCCCCTAAATGGGATGTAATTATCAAGGGATGT

GAGCGGTTTAACTTTAAGTAATGAATCAACTGTTGCATAATCACTTTGAAAGAAACTTGAG

ATTCTTCAATTAGCAAAATCTTGAATAGAATAATGAGATTATTCCTAATGCTAATGATACC

CACCTCTCCTATAAGTTGATGGGGCAGAACAAATTATGCACTCAGGACAACTTTTCTCAAA

TTTTGAAAACTTACCACACGAAGAACACACTTCCACTTGCACAAGTAAGAATATAGTATAA

CAATAAATTTACCACTCATAGCTCAATGGATATTAAAATATTGCTAATCTTTTATAGACAG

ATGTTGGCAAATTAGTATAAATTAGTTATTATATTAGTTTTTAAGGTTCAATATACTTTTT

AAAACTCTTATTAAGTTGTTTAGCTTGCCAACCAAAGACTAAATTATCTTTATTTGTTAGG

AAATAAAAGCACTTATTTTATTTTCTCCATATTTGTTTCCTCTTTGTTCCATTACTAAAAA

AATTATAATATATTAATAACAAAAAAATTTGGGGAGGGAATGTTATTTTTAAAAAAGATGT

TTTATTTATATTTAAGTGTTTTATTGAAATTCCTCGTGAGTTTAGCTCAATTGGTATGGAC

AATACATAATATATGCAAAATTCGAGGTTCAAACCTTGAGCACCACAAAAAAAAGTGTTTT

ATTGAAAAATATCAAGAACTTGAAAACAAATTTCCTTTTTATGAACTAGATATTGTCACAT

AGACAACTTAAGAATTGACTTTTTTTTTAACACTATTGTATTTTCTGAACTCGACTTTAAC

TCGAGATCAATGAGTAAACTAAAAAAACTCGTATTATTTTATCTAAGTGTTCTTAGTGTGA

GAAAACATTATAATTGTTTGCTTAGTCTATTTCTCCCTATATTCTCACCCTCATTCTTGGT

TTGCACATAGAAAGGAGAAAAAAAAAATTAGAGAAGAGTTGAATGGAACAGAAAGAAAGAG

CAAGCAAAATAATCACTAAAAAAAATTCCCCTAAAAGAAAATCACTGAAAAATATTATCCA

CCTCTATTTTAAATAATAATAATAATGAAAACAACAATATATTTTTTTATAGATTTTCTCT

ACTAGAGGTAATTTATTTTAAACACTAAATGTCCACACCTCTAACAAAAAAAATGCATAAT

ATTATAATAGATTATTGTAAGAAAGAGTGACAAATGTTGAATTAGTTATGGAATGAAGAGT

GAGAGAGATTATACAAATAAATAAATATAAATATAAATATAAATAAAGAATAGAGAAGAAC

ATAGTACTTGAATGAAGAGATTTGGCTTGTACTTCCATTACACTTGTAAGCTTTCTACTTC

CTTCACATTCCTTCTCTTTCTCTATCTTTCCTTTTATGATATGTGTACTTTGTTATTGCTT

CATTTACTACCGACATGACTCCAACCTATTAGGGCTTTATCTGAATAATGTTAGTTATTTT

CTCTCATTTTCTTTGCTTCTTCTTATTCTTGGAACTTATAAAGGGTCTTGTCAATTCTATC

GTTCTCTTTTGTATACTTACAAATATAAATATTCTCTTTAGCTTCATTTTCATGTCTTCTT

CTACTAATAAGCTATGGAAATTGAAGTTTTTGTTTTCATGTTTATTTTTCACTTCAGCTCC

TCTTTTTTGATGTTGATGCTGAAGATTGAATAAAAACTGAGGTGGTATGTATGAGAACAAG

AACATGTTTCATTCATCTCTGTTATTTGAACATCTAAATTTTAAACAACTATCGCGACAAT

TTTTTAAGTGGTTGAAGTATTTTAAATAGGTGGTTAATTACATTAAGTAGATAGTTAATTG

ATATATCAGGTGTCAAAACTCATTAACCATCAACATAACTTCATTAGCCATAAATTTTTGA

CGTGATTAACCAAATATTGTAGATGCTCAATTACATGTAGATTTGATGGAGTTGTCCACAA

TTTCTCATATATATATTAACTAATAACCATCTACCGAATATAATTAACCATATATTAGAAT

ATCATTAACCTCTAAAACAGTTGTCGCGAAAATTGGTTGTAAAAAAATCATTTCTATTTTT

TACGCCACTAGTTCTCTTGTTATTTCTCTTTGTTGGAAAGTTGTTGTTTTTAACTTTGAGC

TTAGTCTTATTAATTAACTTGTAATGGTAACAACATTTTTCTATCTTTGTTTGAAGGAAGT

AGTTACTGTGTCAACTTTAGTAATTAATTTGGATCATTTTGGAGGCAAAATATGGCTATGC

AACCTGTTTATTTTAAAGAACATGAAGGAAATGTCCACAATTCTGTTGGACAGTTTTCATC

TGTGACTTCAGCACCATGGTGGAGTAATGCCTATGGATCTCAACCTTTTTATGGAGGAGAC

TCTTGTGGCCAAATGAAACCTTTTTCACTAGAGCTTTCCAACTACATAGACCAACTTGCTC

CGAGTAAGAACTTAGTTCGAGGAGTTGAACAATTGTTTGATAAAGGGCATACAAACCAATT

CACCATCTTTCCAGGTACTTGTTATTCAATATAATTCCGGTTTTGAATGAATTGATTTTTG

TTAAGTTGGTTCTGGATAAACGTGCGTTAAATATAATATGATTTATGTTTGGATACGTTTA

TGTTAAAGTGAGTTGAACATAAATTGGAGACTAAATATCAATTGCAGAGGCAAAAGCTTCA

AATTCTAATTTCAAGTTAGAATCAAATCAATTCTACTCGTAAGCATCTAAATGTTTCAAAA

CCAATTCTACATCTCTAGAACTAAATTTGCATTCAAACATTTTCTGCTACAGATGATTGTA

AGATGTCAGTTGATACACCAAATCATCAAGCAACCTTATCCCTGCAATCACCATTTGCTGC

CGAGCCACTTAATCGATTTGAGCTAGGTTTTAACCAGTCTATGGTAAATCTTCTTATTAAA

GCAATCCCTTTGATCATGTTTTAGTATTTTACTTTTGATGTACATAAATTCATTATGTTTT

TATTATCCTTTCCATGCTAGATCTGTGCAAAATATCCGTATATAGATCAATTTTACGGGCT

CTTCTCGACTTATGGACCTCAAATCTCGGTTTGTCTTCACCTCGGCTATTTTATTAATCTT

TGTATATATCATGCAATGTCTATAAGTACTTGAATGTGAATTTGTTTTATTTACAGTGTGT

ATGCTTACATGAGAAAGCCAGAAATCTGTTTACAACTATCTTGTTAGTATTCTTCTAAATC

TTTCAAATGTAGAGTTGATTATGAAATTTGTTGTTAATGAGTTTTCGGCTTTATGAATGAA

TTGTAAGACAAGTAATGAAACAAAAACTAGAAGAAAATTGAATTATATGATGCAATCTGTG

TTGAAGTTTTTGCATGTATTCAAGTTCTGCTTTTAGTTACTATTTGTTTATCTGCTGCAAT

ATTTCATCAATTATTATTCATGTATGTACCAATATTTAATAAATTAGGGGCGAATTATGCT

TCCGCTTAGCATGACATCTGACGACGGACCAACATACGTGAATGCTAAGCAATACCATGGA

ATCATCAGACGCAGGCATTCTCGTGCCAAAGCTGTGCTTCAGAATAAATTGATAAAGCGTA

ACAAGGTATGAAACTGAGTATTTTTCCTCACAACAATGTTCGAAAACTTGTGAAATAGTAT

ATTTTTCCTTTTACCCTTTTTATACCTATATTAAGGTTTTGTATTTGTCTTGCAGCCATAT

ATGCACGAATCGCGTCATCTACATGCAATGCGTAGACCAAGAGGATGCGGTGGTCGTTTCT

TGAACACAAAAGTTTCTGCTAATGGAAACGGTAAAAGTGGGAGTGAAGAGAACGGAAACAT

TGGTGGCCTACAGCTGCAGTCCAGTGGTTCTCAGAGTTCCGAAGTCTTACAATCTGAGGTT

GGAACTTTAAATTCGTCGAAGGAGACAAACGGAGGCAGTCCAAATGTCTCGGGGTCAGAGG

TGACTAGCATGTATACACAGGGAGGTCTTGATAGCTTTACTGTCAATCATATCGGATCTAC

TGTCCACTCTTTGGGAGACATGATCGATACTGGACACGGTATCGTCATGCCAACAAAATGG

TTTGCAGCAGCTGGCAGACAGCTGCTGGAACCATAAGTTTCGATTCAGAAAGGAAACAAGT

GGGTTTGGTACAATGTGAAATATTTTGCACCAAACTCATCCTTTCCGAGACCAGATGAAGA

AGCTATGTTTCAGTTTGTTGTGTTTACTACGACAAATTTAGTTTCGGAAGACTACTTTTCC

ATCTGGTGCTCAGGCAACTCATTCTTGGCTTATTCTCAGGAAACTCATCCTTGGCTCGTAA

TATTTAGTAGTATTGTCATTGTCTTCCCGCGCAGGCTTTCCGTGGCATGGTAGGCATGCTA

ATGACTTTGGTATTTTCATGCAGTTATAACTATGATGTGTCTTTGTTTGTTGTTAAAATAA

AAAACATGAACTCTAGCTAGGTGCATGTGTGTGTTTTTAATCTTGTCTACTAAGTTTGGTG

TTTTGTAATGGATTTCTGACTTTATGGAGCAATGTATTGTAACTCTACTAAGAAGTGTAAC

ATTTTATTTCTCCCTCTCTAAGGATTGTATAAGAACCTCTTATTTTCAGACTCTACTTAAT

CCTATTTTCTATGTCTGTATGATTTTTATATTTCTAGGACAATCAAATTGGCTTGTAGAAG

CTCAAAAGCATGCTCACAAAGTAGGTACTTATGTAGGGAACTTCGTACTCTAATAATAACT

GGTTATTAACGTTATAATTAAATGCAAAATTTGATAAGTAGTAGGGTTTGGTAAGTAATAT

AACAACACCATGTTGGCTTGGGATTCTGAATTGTGTTATTAGAGTACAACTCTTAAATAAT

GATCGTAATGTAAATAATTTAAATTTGAAGTTACTAGATAAAAAGATAATATTATGTAATA

TATTATCAAATATGTACTAAAAATGTAATTTTCATCTATCTCAATTGAAAAAATATCTGAG

TTTTGAGTCTATTGTCATGGACGA

>HM015

AAAAAAATAAAGGTTCTATACTCATTAAACAAAGTGCCAAATTCAGAGATACTACTTCCT

TTGGATAATTTGTATCGTCACCAACGATCTTTGAGTTATTTCAAAAATCACATTCTCAATT

TTGTGGTCCAACATTCACAAAATGGTTTGTCGGGCTTTAAGTCTAGTAATTGTGAAACATG

CATATGCATGAGGTAATTAATTGATAATCTGAAAAGGTTAAATAGTTAATCAATTGTACTT

TGGTTCTTTTTAATTTGTTCTCTATAATTTTTCAGTCAAAATTGAATTTGCTTTAACCATT

TAGTGTAGTAGTATGATATTACTTGCTCTACATTCTTGGATCTAAAGGTCTAATTAGTATC

GTATAAAAAATATTCTAGTTAATTAAGATACTTTTGTTCATTCTAATTATTCTTGGATTAC

TTGTGAAACTTTTAGGGATGAACAAGAAAGTCCACTAAAGAGGCTTCTTTTTGCTAGGAGT

CACTAGTTAGTTTTTTTTTTTTTTGACAAAAGTTAACCATTTTTTTTTATCGCATGGTAGG

AAAAAAAAAATAGAGATTTCCTTTGGTACTCATAAAAAAAAAAGTTGGATGGATAATGGAT

TAATCATAACTTATTTAACATAAAATGAACTAAGAGAACATTTCATATTTGGGTTTATTTT

GTAACATAAATATTAATATCAATGTTTGAAAGAGTTTTTAACAATAATTTGTAAAAGAAAG

CATAATATATCTATCAAAATAATTATAAAAGAAAAAATTTCTTTTGGTGGTATGACATGGA

AATATAATTAATTCTTATTAGATGAAAGTGTAAAATTAATTTGCATTAACAAAGCATATTT

CATTAAACTCCTTTTAGTTTTTGTTGGAAAAAATAAATTTATTGTTACTCAAAAAATAAAA

GTGTACAATTAATATATTTACTCAAAAGTCCCCTAAATGGGATGTAATTATCAAGGGATGT

GAGCGGTTTAACTTTAAGTAATGAATCAACTGTTGCATAATCACTTTGAAAGAAACTTGAG

ATTCTTCAATTAGCAAAATCTTGAATAGAATAATGAGATTATTCCTAATGCTAATGATACC

CACCTCTCCTATAAGTTGATGGGGCAGAACAAATTATGCACTCAGGACAACTTTTCTCAAA

TTTTGAAAACTTACCACACGAAGAACACACTTCCACTTGCACAAGTAAGAATATAGTATAA

CAATAAATTTACCACTCATAGCTCAATGGATATTAAAATATTGCTAATCTTTTATAGACAG

ATGTTGGCAAATTAGTATAAATTAGTTATTATATTAGTTTTTAAGGTTCAATATACTTTTT

AAAACTCTTATTAAGTTGTTTAGCTTGCCAACCAAAGACTAAATTATCTTTATTTGTTAGG

AAATAAAAGCACTTATTTTATTTTCTCCATATTTGTTTCCTCTTTGTTCCATTACTAAAAA

AATTATAATATATTAATAACAAAAAAATTTGGGGAGGGAATGTTATTTTTAAAAAAGATGT

TTTATTTATATTTAAGTGTTTTATTGAAATTCCTCGTGAGTTTAGCTCAATTGGTATGGAC

AATACATAATATATGCAAAATTCGAGGTTCAAACCTTGAGCACCACAAAAAAAAGTGTTTT

ATTGAAAAATATCAAGAACTTGAAAACAAATTTCCTTTTTATGAACTAGATATTGTCACAT

AGACAACTTAAGAATTGACTTTTTTTTTAACACTATTGTATTTTCTGAACTCGACTTTAAC

TCGAGATCAATGAGTAAACTAAAAAAACTCGTATTATTTTATCTAAGTGTTCTTAGTGTGA

GAAAACATTATAATTGTTTGCTTAGTCTATTTCTCCCTATATTCTCACCCTCATTCTTGGT

TTGCACATAGAAAGGAGAAAAAAAAAATTAGAGAAGAGTTGAATGGAACAGAAAGAAAGAG

CAAGCAAAATAATCACTAAAAAAAATTCCCCTAAAAGAAAATCACTGAAAAATATTATCCA

CCTCTATTTTAAATAATAATAATAATGAAAACAACAATATATTTTTTTATAGATTTTCTCT

ACTAGAGGTAATTTATTTTAAACACTAAATGTCCACACCTCTAACAAAAAAAATGCATAAT

ATTATAATAGATTATTGTAAGAAAGAGTGACAAATGTTGAATTAGTTATGGAATGAAGAGT

GAGAGAGATTATACAAATAAATAAATATAAATATAAATATAAATAAAGAATAGAGAAGAAC

ATAGTACTTGAATGAAGAGATTTGGCTTGTACTTCCATTACACTTGTAAGCTTTCTACTTC

CTTCACATTCCTTCTCTTTCTCTATCTTTCCTTTTATGATATGTGTACTTTGTTATTGCTT

CATTTACTACCGACATGACTCCAACCTATTAGGGCTTTATCTGAATAATGTTAGTTATTTT

CTCTCATTTTCTTTGCTTCTTCTTATTCTTGGAACTTATAAAGGGTCTTGTCAATTCTATC

GTTCTCTTTTGTATACTTACAAATATAAATATTCTCTTTAGCTTCATTTTCATGTCTTCTT

CTACTAATAAGCTATGGAAATTGAAGTTTTTGTTTTCATGTTTATTTTTCACTTCAGCTCC

TCTTTTTTGATGTTGATGCTGAAGATTGAATAAAAACTGAGGTGGTATGTATGAGAACAAG

AACATGTTTCATTCATCTCTGTTATTTGAACATCTAAATTTTAAACAACTATCGCGACAAT

TTTTTAAGTGGTTGAAGTATTTTAAATAGGTGGTTAATTACATTAAGTAGATAGTTAATTG

ATATATCAGGTGTCAAAACTCATTAACCATCAACATAACTTCATTAGCCATAAATTTTTGA

CGTGATTAACCAAATATTGTAGATGCTCAATTACATGTAGATTTGATGGAGTTGTCCACAA

TTTCTCATATATATATTAACTAATAACCATCTACCGAATATAATTAACCATATATTAGAAT

ATCATTAACCTCTAAAACAGTTGTCGCGAAAATTGGTTGTAAAAAAATCATTTCTATTTTT

TACGCCACTAGTTCTCTTGTTATTTCTCTTTGTTGGAAAGTTGTTGTTTTTAACTTTGAGC

TTAGTCTTATTAATTAACTTGTAATGGTAACAACATTTTTCTATCTTTGTTTGAAGGAAGT

AGTTACTGTGTCAACTTTAGTAATTAATTTGGATCATTTTGGAGGCAAAATATGGCTATGC

AACCTGTTTATTTTAAAGAACATGAAGGAAATGTCCACAATTCTGTTGGACAGTTTTCATC

TGTGACTTCAGCACCATGGTGGAGTAATGCCTATGGATCTCAACCTTTTTATGGAGGAGAC

TCTTGTGGCCAAATGAAACCTTTTTCACTAGAGCTTTCCAACTACATAGACCAACTTGCTC

CGAGTAAGAACTTAGTTCGAGGAGTTGAACAATTGTTTGATAAAGGGCATACAAACCAATT

CACCATCTTTCCAGGTACTTGTTATTCAATATAATTCCGGTTTTGAATGAATTGATTTTTG

TTAAGTTGGTTCTGGATAAACGTGCGTTAAATATAATATGATTTATGTTTGGATACGTTTA

TGTTAAAGTGAGTTGAACATAAATTGGAGACTAAATATCAATTGCAGAGGCAAAAGCTTCA

AATTCTAATTTCAAGTTAGAATCAAATCAATTCTACTCGTAAGCATCTAAATGTTTCAAAA

CCAATTCTACATCTCTAGAACTAAATTTGCATTCAAACATTTTCTGCTACAGATGATTGTA

AGATGTCAGTTGATACACCAAATCATCAAGCAACCTTATCCCTGCAATCACCATTTGCTGC

CGAGCCACTTAATCGATTTGAGCTAGGTTTTAACCAGTCTATGGTAAATCTTCTTATTAAA

GCAATCCCTTTGATCATGTTTTAGTATTTTACTTTTGATGTACATAAATTCATTATGTTTT

TATTATCCTTTCCATGCTAGATCTGTGCAAAATATCCGTATATAGATCAATTTTACGGGCT

CTTCTCGACTTATGGACCTCAAATCTCGGTTTGTCTTCACCTCGGCTATTTTATTAATCTT

TGTATATATCATGCAATGTCTATAAGTACTTGAATGTGAATTTGTTTTATTTACAGTGTGT

ATGCTTACATGAGAAAGCCAGAAATCTGTTTACAACTATCTTGTTAGTATTCTTCTAAATC

TTTCAAATGTAGAGTTGATTATGAAATTTGTTGTTAATGAGTTTTCGGCTTTATGAATGAA

TTGTAAGACAAGTAATGAAACAAAAACTAGAAGAAAATTGAATTATATGATGCAATCTGTG

TTGAAGTTTTTGCATGTATTCAAGTTCTGCTTTTAGTTACTATTTGTTTATCTGCTGCAAT

ATTTCATCAATTATTATTCATGTATGTACCAATATTTAATAAATTAGGGGCGAATTATGCT

TCCGCTTAGCATGACATCTGACGACGGACCAACATACGTGAATGCTAAGCAATACCATGGA

ATCATCAGACGCAGGCATTCTCGTGCCAAAGCTGTGCTTCAGAATAAATTGATAAAGCGTA

ACAAGGTATGAAACTGAGTATTTTTCCTCACAACAATGTTCGAAAACTTGTGAAATAGTAT

ATTTTTCCTTTTACCCTTTTTATACCTATATTAAGGTTTTGTATTTGTCTTGCAGCCATAT

ATGCACGAATCGCGTCATCTACATGCAATGCGTAGACCAAGAGGATGCGGTGGTCGTTTCT

TGAACACAAAAGTTTCTGCTAATGGAAACGGTAAAAGTGGGAGTGAAGAGAACGGAAACAT

TGGTGGCCTACAGCTGCAGTCCAGTGGTTCTCAGAGTTCCGAAGTCTTACAATCTGAGGTT

GGAACTTTAAATTCGTCGAAGGAGACAAACGGAGGCAGTCCAAATGTCTCGGGGTCAGAGG

TGACTAGCATGTATACACAGGGAGGTCTTGATAGCTTTACTGTCAATCATATCGGATCTAC

TGTCCACTCTTTGGGAGACATGATCGATACTGGACACGGTATCGTCATGCCAACAAAATGG

TTTGCAGCAGCTGGCAGACAGCTGCTGGAACCATAAGTTTCGATTCAGAAAGGAAACAAGT

GGGTTTGGTACAATGTGAAATATTTTGCACCAAACTCATCCTTTCCGAGACCAGATGAAGA

AGCTATGTTTCAGTTTGTTGTGTTTACTACGACAAATTTAGTTTCGGAAGACTACTTTTCC

ATCTGGTGCTCAGGCAACTCATTCTTGGCTTATTCTCAGGAAACTCATCCTTGGCTCGTAA

TATTTAGTAGTATTGTCATTGTCTTCCCGCGCAGGCTTTCCGTGGCATGGTAGGCATGCTA

ATGACTTTGGTATTTTCATGCAGTTATAACTATGATGTGTCTTTGTTTGTTGTTAAAATAA

AAAACATGAACTCTAGCTAGGTGCATGTGTGTGTTTTTAATCTTGTCTACTAAGTTTGGTG

TTTTGTAATGGATTTCTGACTTTATGGAGCAATGTATTGTAACTCTACTAAGAAGTGTAAC

ATTTTATTTCTCCCTCTCTAAGGATTGTATAAGAACCTCTTATTTTCAGACTCTACTTAAT

CCTATTTTCTATGTCTGTATGATTTTTATATTTCTAGGACAATCAAATTGGCTTGTAGAAG

CTCAAAAGCATGCTCACAAAGTAGGTACTTATGTAGGGAACTTCGTACTCTAATAATAACT

GGTTATTAACGTTATAATTAAATGCAAAATTTGATAAGTAGTAGGGTTTGGTAAGTAATAT

AACAACACCATGTTGGCTTGGGATTCTGAATTGTGTTATTAGAGTACAACTCTTAAATAAT

GATCGTAATGTAAATAATTTAAATTTGAAGTTACTAGATAAAAAGATAATATTATGTAATA

TATTATCAAATATGTACTAAAAATGTAATTTTCATCTATCTCAATTGAAAAAATATCTGAG

TTTTGAGTCTATTGTCATGGACGA

>HM016

AAAAAAATAAAGGTTCTATACTCATTAAACAAAGTGCCAAATTCAGAGATACTACTTCCT

TTGGATAATTTGTATCGTCACCAACGATCTTTGAGTTATTTCAAAAATCACATTCTCAATT

TTGTGGTCCAACATTCACAAAATGGTTTGTCGGGCTTTAAGTCTAGTAATTGTGAAACATG

CATATGCATGAGGTAATTAATTGATAATCTGAAAAGGTTAAATAGTTAATCAATTGTACTT

TGGTTCTTTTTAATTTGTTCTCTATAATTTTTCAGTCAAAATTGAATTTGCTTTAACCATT

TAGTGTAGTAGTATGATATTACTTGCTCTACATTCTTGGATCTAAAGGTCTAATTAGTATC

GTATAAAAAATATTCTAGTTAATTAAGATACTTTTGTTCATTCTAATTATTCTTGGATTAC

TTGTGAAACTTTTAGGGATGAACAAGAAAGTCCACTAAAGAGGCTTCTTTTTGCTAGGAGT

CACTAGTTAGTTTTTTTTTTTTTTGACAAAAGTTAACCATTTTTTTTTATCGCATGGTAGG

AAAAAAAAAATAGAGATTTCCTTTGGTACTCATAAAAAAAAAAGTTGGATGGATAATGGAT

TAATCATAACTTATTTAACATAAAATGAACTAAGAGAACATTTCATATTTGGGTTTATTTT

GTAACATAAATATTAATATCAATGTTTGAAAGAGTTTTTAACAATAATTTGTAAAAGAAAG

CATAATATATCTATCAAAATAATTATAAAAGAAAAAATTTCTTTTGGTGGTATGACATGGA

AATATAATTAATTCTTATTAGATGAAAGTGTAAAATTAATTTGCATTAACAAAGCATATTT

CATTAAACTCCTTTTAGTTTTTGTTGGAAAAAATAAATTTATTGTTACTCAAAAAATAAAA

GTGTACAATTAATATATTTACTCAAAAGTCCCCTAAATGGGATGTAATTATCAAGGGATGT

GAGCGGTTTAACTTTAAGTAATGAATCAACTGTTGCATAATCACTTTGAAAGAAACTTGAG

ATTCTTCAATTAGCAAAATCTTGAATAGAATAATGAGATTATTCCTAATGCTAATGATACC

CACCTCTCCTATAAGTTGATGGGGCAGAACAAATTATGCACTCAGGACAACTTTTCTCAAA

TTTTGAAAACTTACCACACGAAGAACACACTTCCACTTGCACAAGTAAGAATATAGTATAA

CAATAAATTTACCACTCATAGCTCAATGGATATTAAAATATTGCTAATCTTTTATAGACAG

ATGTTGGCAAATTAGTATAAATTAGTTATTATATTAGTTTTTAAGGTTCAATATACTTTTT

AAAACTCTTATTAAGTTGTTTAGCTTGCCAACCAAAGACTAAATTATCTTTATTTGTTAGG

AAATAAAAGCACTTATTTTATTTTCTCCATATTTGTTTCCTCTTTGTTCCATTACTAAAAA

AATTATAATATATTAATAACAAAAAAATTTGGGGAGGGAATGTTATTTTTAAAAAAGATGT

TTTATTTATATTTAAGTGTTTTATTGAAATTCCTCGTGAGTTTAGCTCAATTGGTATGGAC

AATACATAATATATGCAAAATTCGAGGTTCAAACCTTGAGCACCACAAAAAAAAGTGTTTT

ATTGAAAAATATCAAGAACTTGAAAACAAATTTCCTTTTTATGAACTAGATATTGTCACAT

AGACAACTTAAGAATTGACTTTTTTTTTAACACTATTGTATTTTCTGAACTCGACTTTAAC

TCGAGATCAATGAGTAAACTAAAAAAACTCGTATTATTTTATCTAAGTGTTCTTAGTGTGA

GAAAACATTATAATTGTTTGCTTAGTCTATTTCTCCCTATATTCTCACCCTCATTCTTGGT

TTGCACATAGAAAGGAGAAAAAAAAAATTAGAGAAGAGTTGAATGGAACAGAAAGAAAGAG

CAAGCAAAATAATCACTAAAAAAAATTCCCCTAAAAGAAAATCACTGAAAAATATTATCCA

CCTCTATTTTAAATAATAATAATAATGAAAACAACAATATATTTTTTTATAGATTTTCTCT

ACTAGAGGTAATTTATTTTAAACACTAAATGTCCACACCTCTAACAAAAAAAATGCATAAT

ATTATAATAGATTATTGTAAGAAAGAGTGACAAATGTTGAATTAGTTATGGAATGAAGAGT

GAGAGAGATTATACAAATAAATAAATATAAATATAAATATAAATAAAGAATAGAGAAGAAC

ATAGTACTTGAATGAAGAGATTTGGCTTGTACTTCCATTACACTTGTAAGCTTTCTACTTC

CTTCACATTCCTTCTCTTTCTCTATCTTTCCTTTTATGATATGTGTACTTTGTTATTGCTT

CATTTACTACCGACATGACTCCAACCTATTAGGGCTTTATCTGAATAATGTTAGTTATTTT

CTCTCATTTTCTTTGCTTCTTCTTATTCTTGGAACTTATAAAGGGTCTTGTCAATTCTATC

GTTCTCTTTTGTATACTTACAAATATAAATATTCTCTTTAGCTTCATTTTCATGTCTTCTT

CTACTAATAAGCTATGGAAATTGAAGTTTTTGTTTTCATGTTTATTTTTCACTTCAGCTCC

TCTTTTTTGATGTTGATGCTGAAGATTGAATAAAAACTGAGGTGGTATGTATGAGAACAAG

AACATTTTTCATTCATCTCTGTTATTTGAACATCTAAATTTTAAACAACTATCGCGACAAT

TTTTTAAGTGGTTGAAGTATTTTAAATAGGTGGTTAATTACATTAAGTAGATAGTTAATTG

ATATATCAGGTGTCAAAACTCATTAACCATCAACATAACTTCATTAGCCATAAATTTTTGA

CGTGATTAACCAAATATTGTAGATGCTCAATTACATGTAGATTTGATGGAGTTGTCCACAA

TTTCTCATATATATATTAACTAATAACCATCTACCGAATATAATTAACCATATATTAGAAT

ATCATTAACCTCTAAAACAGTTGTCGCGAAAATTGGTTGTAAAAAAATCATTTCTATTTTT

TACGCCACTAGTTCTCTTGTTATTTCTCTTTGTTGGAAAGTTGTTGTTTTTAACTTTGAGC

TTAGTCTTATTAATTAACTTGTAATGGTAACAACATTTTTCTATCTTTGTTTGAAGGAAGT

AGTTACTGTGTCAACTTTAGTAATTAATTTGGATCATTTTGGAGGCAAAATATGGCTATGC

AACCTGTTTATTTTAAAGAACATGAAGGAAATGTCCACAATTCTGTTGGACAGTTTTCATC

TGTGACTTCAGCACCATGGTGGAGTAATGCCTATGGATCTCAACCTTTTTATGGAGGAGAC

TCTTGTGGCCAAATGAAACCTTTTTCACTAGAGCTTTCCAACTACATAGACCAACTTGCTC

CGAGTAAGAACTTAGTTCGAGGAGTTGAACAATTGTTTGATAAAGGGCATACAAACCAATT

CACCATCTTTCCAGGTACTTGTTATTCAATATAATTCCGGTTTTGAATGAATTGATTTTTG

TTAAGTTGGTTCTGGATAAACGTGCGTTAAATATAATATGATTTATGTTTGGATACGTTTA

TGTTAAAGTGAGTTGAACATAAATTGGAGACTAAATATCAATTGCAGAGGCAAAAGCTTCA

AATTCTAATTTCAAGTTAGAATCAAATCAATTCTACTCGTAAGCATCTAAATGTTTCAAAA

CCAATTCTACATCTCTAGAACTAAATTTGCATTCAAACATTTTCTGCTACAGATGATTGTA

AGATGTCAGTTGATACACCAAATCATCAAGCAACCTTATCCCTGCAATCACCATTTGCTGC

CGAGCCACTTAATCGATTTGAGCTAGGTTTTAACCAGTCTATGGTAAATCTTCTTATTAAA

GCAATCCCTTTGATCATGTTTTAGTATTTTACTTTTGATGTACATAAATTCATTATGTTTT

TATTATCCTTTCCATGCTAGATCTGTGCAAAATATCCGTATATAGATCAATTTTACGGGCT

CTTCTCGACTTATGGACCTCAAATCTCGGTTTGTCTTCACCTCGGCTATTTTATTAATCTT

TGTATATATCATGCAATGTCTATAAGTACTTGAATGTGAATTTGTTTTATTTACAGTGTGT

ATGCTTACATGAGAAAGCCAGAAATCTGTTTACAACTATCTTGTTAGTATTCTTCTAAATC

TTTCAAATGTAGAGTTGATTATGAAATTTGTTGTTAATGAGTTTTCGGCTTTATGAATGAA

TTGTAAGACAAGTAATGAAACAAAAACTAGAAGAAAATTGAATTATATGATGCAATCTGTG

TTGAAGTTTTTGCATGTATTCAAGTTCTGCTTTTAGTTACTATTTGTTTATCTGCTGCAAT

ATTTCATCAATTATTATTCATGTATGTACCAATATTTAATAAATTAGGGGCGAATTATGCT

TCCGCTTAGCATGACATCTGACGACGGACCAACATACGTGAATGCTAAGCAATACCATGGA

ATCATCAGACGCAGGCATTCTCGTGCCAAAGCTGTGCTTCAGAATAAATTGATAAAGCGTA

ACAAGGTATGAAACTGAGTATTTTTCCTCACAACAATGTTCGAAAACTTGTGAAATAGTAT

ATTTTTCCTTTTACCCTTTTTATACCTATATTAAGGTTTTGTATTTGTCTTGCAGCCATAT

ATGCACGAATCGCGTCATCTACATGCAATGCGTAGACCAAGAGGATGCGGTGGTCGTTTCT

TGAACACAAAAGTTTCTGCTAATGGAAACGGTAAAAGCGGGAGTGAAGAGAACGGAAACAT

TGGTGGCCTACAGCTGCAGTCCAGTGGTTCTCAGAGTTCCGAAGTCTTACAATCTGAGGTT

GGAACTTTAAATTCGTCGAAGGAGACAAACGGAGGCAGTCCAAATGTCTCGGGGTCAGAGG

TGACTAGCATGTATACACAGGGAGGTCTTGATAGCTTTACTGTCAATCATATCGGATCTAC

TGTCCACTCTTTGGGAGACATGATCGATACTGGACACGGTATCGTCATGCCAACAAAATGG

TTTGCAGCAGCTGGCAGACAGCTGCTGGAACCATAAGTTTCGATTCAGAAAGGAAACAAGT

GGGTTTGGTACAATGTGAAATATTTTGCACCAAACTCATCCTTTCCGAGACCAGATGAAGA

AGCTATGTTTCAGTTTGTTGTGTTTACTACGACAAATTTAGTTTCGGAAGACTACTTTTCC

ATCTGGTGCTCAGGCAACTCATTCTTGGCTTATTCTCAGGAAACTCATCCTTGGCTCGTAA

TATTTAGTAGTATTGTCATTGTCTTCCCGCGCAGGCTTTCCGTGGCATGGTAGGCATGCTA

ATGACTTTGGTATTTTCATGCAGTTATAACTATGATGTGTCTTTGTTTGTTGTTAAAATAA

AAAACATGAACTCTAGCTAGGTGCATGTGTGTGTTTTTAATCTTGTCTACTAAGTTTGGTG

TTTTGTAATGGATTTCTGACTTTATGGAGCAATGTATTGTAACTCTACTAAGAAGTGTAAC

ATTTTATTTCTCCCTCTCTAAGGATTGTATAAGAACCTCTTATTTTCAGACTCTACTTAAT

CCTATTTTCTATGTCTGTATGATTTTTATATTTCTAGGACAATCAAATTGGCTTGTAGAAG

CTCAAAAGCATGCTCACAAAGTAGGTACTTATGTAGGGAACTTCGTACTCTAATAATAACT

GGTTATTAACGTTATAATTAAATGCAAAATTTGATAAGTAGTAGGGTTTGGTAAGTAATAT

AACAACACCATGTTGGCTTGGGATTCTGAATTGTGTTATTAGAGTACAACTCTTAAATAAT

GATCGTAATGTAAATAATTTAAATTTGAAGTTACTAGATAAAAAGATAATATTATGTAATA

TATTATCAAATATGTACTAAAAATGTAATTTTCATCTATCTCAATTGAAAAAATATCTGAG

TTTTGAGTCTATTGTCATGGACGA

>HM019

AAAAAAATAAAGGTTCTATACTCATTAAACAAAGTGCCAAATTCAGAGATACTACTTCCT

TTGGATAATTTGTATCGTCACCAACGATCTTTGAGTTATTTCAAAAATCACATTCTCAATT

TTGTGGTCCAACATTCACAAAATGGTTTGTCGGGCTTTAAGTCTAGTAATTGTGAAACATG

CATATGCATGAGGTAATTAATTGATAATCTGAAAAGGTTAAATAGTTAATCAATTGTACTT

TGGTTCTTTTTAATTTGTTCTCTATAATTTTTCAGTCAAAATTGAATTTGCTTTAACCATT

TAGTGTAGTAGTATGATATTACTTGCTCTACATTCTTGGATCTAAAGGTCTAATTAGTATC

GTATAAAAAATATTCTAGTTAATTAAGATACTTTTGTTCATTCTAATTATTCTTGGATTAC

TTGTGAAACTTTTAGGGATGAACAAGAAAGTCCACTAAAGAGGCTTCTTTTTGCTAGGAGT

CACTAGTTAGTTTTTTTTTTTTTTGACAAAAGTTAACCATTTTTTTTTATCGCATGGTAGG

AAAAAAAAAATAGAGATTTCCTTTGGTACTCATAAAAAAAAAAGTTGGATGGATAATGGAT

TAATCATAACTTATTTAACATAAAATGAACTAAGAGAACATTTCATATTTGGGTTTATTTT

GTAACATAAATATTAATATCAATGTTTGAAAGAGTTTTTAACAATAATTTGTAAAAGAAAG

CATAATATATCTATCAAAATAATTATAAAAGAAAAAATTTCTTTTGGTGGTATGACATGGA

AATATAATTAATTCTTATTAGATGAAAGTGTAAAATTAATTTGCATTAACAAAGCATATTT

CATTAAACTCCTTTTAGTTTTTGTTGGAAAAAATAAATTTATTGTTACTCAAAAAATAAAA

GTGTACAATTAATATATTTACTCAAAAGTCCCCTAAATGGGATGTAATTATCAAGGGATGT

GAGCGGTTTAACTTTAAGTAATGAATCAACTGTTGCATAATCACTTTGAAAGAAACTTGAG

ATTCTTCAATTAGCAAAATCTTGAATAGAATAATGAGATTATTCCTAATGCTAATGATACC

CACCTCTCCTATAAGTTGATGGGGCAGAACAAATTATGCACTCAGGACAACTTTTCTCAAA

TTTTGAAAACTTACCACACGAAGAACACACTTCCACTTGCACAAGTAAGAATATAGTATAA

CAATAAATTTACCACTCATAGCTCAATGGATATTAAAATATTGCTAATCTTTTATAGACAG

ATGTTGGCAAATTAGTATAAATTAGTTATTATATTAGTTTTTAAGGTTCAATATACTTTTT

AAAACTCTTATTAAGTTGTTTAGCTTGCCAACCAAAGACTAAATTATCTTTATTTGTTAGG

AAATAAAAGCACTTATTTTATTTTCTCCATATTTGTTTCCTCTTTGTTCCATTACTAAAAA

AATTATAATATATTAATAACAAAAAAATTTGGGGAGGGAATGTTATTTTTAAAAAAGATGT

TTTATTTATATTTAAGTGTTTTATTGAAATTCCTCGTGAGTTTAGCTCAATTGGTATGGAC

AATACATAATATATGCAAAATTCGAGGTTCAAACCTTGAGCACCACAAAAAAAAGTGTTTT

ATTGAAAAATATCAAGAACTTGAAAACAAATTTCCTTTTTATGAACTAGATATTGTCACAT

AGACAACTTAAGAATTGACTTTTTTTTTAACACTATTGTATTTTCTGAACTCGACTTTAAC

TCGAGATCAATGAGTAAACTAAAAAAACTCGTATTATTTTATCTAAGTGTTCTTAGTGTGA

GAAAACATTATAATTGTTTGCTTAGTCTATTTCTCCCTATATTCTCACCCTCATTCTTGGT

TTGCACATAGAAAGGAGAAAAAAAAAATTAGAGAAGAGTTGAATGGAACAGAAAGAAAGAG

CAAGCAAAATAATCACTAAAAAAAATTCCCCTAAAAGAAAATCACTGAAAAATATTATCCA

CCTCTATTTTAAATAATAATAATAATGAAAACAACAATATATTTTTTTATAGATTTTCTCT

ACTAGAGGTAATTTATTTTAAACACTAAATGTCCACACCTCTAACAAAAAAAATGCATAAT

ATTATAATAGATTATTGTAAGAAAGAGTGACAAATGTTGAATTAGTTATGGAATGAAGAGT

GAGAGAGATTATACAAATAAATAAATATAAATATAAATATAAATAAAGAATAGAGAAGAAC

ATAGTACTTGAATGAAGAGATTTGGCTTGTACTTCCATTACACTTGTAAGCTTTCTACTTC

CTTCACATTCCTTCTCTTTCTCTATCTTTCCTTTTATGATATGTGTACTTTGTTATTGCTT

CATTTACTACCGACATGACTCCAACCTATTAGGGCTTTATCTGAATAATGTTAGTTATTTT

CTCTCATTTTCTTTGCTTCTTCTTATTCTTGGAACTTATAAAGGGTCTTGTCAATTCTATC

GTTCTCTTTTGTATACTTACAAATATAAATATTCTCTTTAGCTTCATTTTCATGTCTTCTT

CTACTAATAAGCTATGGAAATTGAAGTTTTTGTTTTCATGTTTATTTTTCACTTCAGCTCC

TCTTTTTTGATGTTGATGCTGAAGATTGAATAAAAACTGAGGTGGTATGTATGAGAACAAG

AACATTTTTCATTCATCTCTGTTATTTGAACATCTAAATTTTAAACAACTATCGCGACAAT

TTTTTAAGTGGTTGAAGTATTTTAAATAGGTGGTTAATTACATTAAGTAGATAGTTAATTG

ATATATCAGGTGTCAAAACTCATTAACCATCAACATAACTTCATTAGCCATAAATTTTTGA

CGTGATTAACCAAATATTGTAGATGCTCAATTACATGTAGATTTGATGGAGTTGTCCACAA

TTTCTCATATATATATTAACTAATAACCATCTACCGAATATAATTAACCATATATTAGAAT

ATCATTAACCTCTAAAACAGTTGTCGCGAAAATTGGTTGTAAAAAAATCATTTCTATTTTT

TACGCCACTAGTTCTCTTGTTATTTCTCTTTGTTGGAAAGTTGTTGTTTTTAACTTTGAGC

TTAGTCTTATTAATTAACTTGTAATGGTAACAACATTTTTCTATCTTTGTTTGAAGGAAGT

AGTTACTGTGTCAACTTTAGTAATTAATTTGGATCATTTTGGAGGCAAAATATGGCTATGC

AACCTGTTTATTTTAAAGAACATGAAGGAAATGTCCACAATTCTGTTGGACAGTTTTCATC

TGTGACTTCAGCACCATGGTGGAGTAATGCCTATGGATCTCAACCTTTTTATGGAGGAGAC

TCTTGTGGCCAAATGAAACCTTTTTCACTAGAGCTTTCCAACTACATAGACCAACTTGCTC

CGAGTAAGAACTTAGTTCGAGGAGTTGAACAATTGTTTGATAAAGGGCATACAAACCAATT

CACCATCTTTCCAGGTACTTGTTATTCAATATAATTCCGGTTTTGAATGAATTGATTTTTG

TTAAGTTGGTTCTGGATAAACGTGCGTTAAATATAATATGATTTATGTTTGGATACGTTTA

TGTTAAAGTGAGTTGAACATAAATTGGAGACTAAATATCAATTGCAGAGGCAAAAGCTTCA

AATTCTAATTTCAAGTTAGAATCAAATCAATTCTACTCGTAAGCATCTAAATGTTTCAAAA

CCAATTCTACATCTCTAGAACTAAATTTGCATTCAAACATTTTCTGCTACAGATGATTGTA

AGATGTCAGTTGATACACCAAATCATCAAGCAACCTTATCCCTGCAATCACCATTTGCTGC

CGAGCCACTTAATCGATTTGAGCTAGGTTTTAACCAGTCTATGGTAAATCTTCTTATTAAA

GCAATCCCTTTGATCATGTTTTAGTATTTTACTTTTGATGTACATAAATTCATTATGTTTT

TATTATCCTTTCCATGCTAGATCTGTGCAAAATATCCGTATATAGATCAATTTTACGGGCT

CTTCTCGACTTATGGACCTCAAATCTCGGTTTGTCTTCACCTCGGCTATTTTATTAATCTT

TGTATATATCATGCAATGTCTATAAGTACTTGAATGTGAATTTGTTTTATTTACAGTGTGT

ATGCTTACATGAGAAAGCCAGAAATCTGTTTACAACTATCTTGTTAGTATTCTTCTAAATC

TTTCAAATGTAGAGTTGATTATGAAATTTGTTGTTAATGAGTTTTCGGCTTTATGAATGAA

TTGTAAGACAAGTAATGAAACAAAAACTAGAAGAAAATTGAATTATATGATGCAATCTGTG

TTGAAGTTTTTGCATGTATTCAAGTTCTGCTTTTAGTTACTATTTGTTTATCTGCTGCAAT

ATTTCATCAATTATTATTCATGTATGTACCAATATTTAATAAATTAGGGGCGAATTATGCT

TCCGCTTAGCATGACATCTGACGACGGACCAACATACGTGAATGCTAAGCAATACCATGGA

ATCATCAGACGCAGGCATTCTCGTGCCAAAGCTGTGCTTCAGAATAAATTGATAAAGCGTA

ACAAGGTATGAAACTGAGTATTTTTCCTCACAACAATGTTCGAAAACTTGTGAAATAGTAT

ATTTTTCCTTTTACCCTTTTTATACCTATATTAAGGTTTTGTATTTGTCTTGCAGCCATAT

ATGCACGAATCGCGTCATCTACATGCAATGCGTAGACCAAGAGGATGCGGTGGTCGTTTCT

TGAACACAAAAGTTTCTGCTAATGGAAACGGTAAAAGCGGGAGTGAAGAGAACGGAAACAT

TGGTGGCCTACAGCTGCAGTCCAGTGGTTCTCAGAGTTCCGAAGTCTTACAATCTGAGGTT

GGAACTTTAAATTCGTCGAAGGAGACAAACGGAGGCAGTCCAAATGTCTCGGGGTCAGAGG

TGACTAGCATGTATACACAGGGAGGTCTTGATAGCTTTACTGTCAATCATATCGGATCTAC

TGTCCACTCTTTGGGAGACATGATCGATACTGGACACGGTATCGTCATGCCAACAAAATGG

TTTGCAGCAGCTGGCAGACAGCTGCTGGAACCATAAGTTTCGATTCAGAAAGGAAACAAGT

GGGTTTGGTACAATGTGAAATATTTTGCACCAAACTCATCCTTTCCGAGACCAGATGAAGA

AGCTATGTTTCAGTTTGTTGTGTTTACTACGACAAATTTAGTTTCGGAAGACTACTTTTCC

ATCTGGTGCTCAGGCAACTCATTCTTGGCTTATTCTCAGGAAACTCATCCTTGGCTCGTAA

TATTTAGTAGTATTGTCATTGTCTTCCCGCGCAGGCTTTCCGTGGCATGGTAGGCATGCTA

ATGACTTTGGTATTTTCATGCAGTTATAACTATGATGTGTCTTTGTTTGTTGTTAAAATAA

AAAACATGAACTCTAGCTAGGTGCATGTGTGTGTTTTTAATCTTGTCTACTAAGTTTGGTG

TTTTGTAATGGATTTCTGACTTTATGGAGCAATGTATTGTAACTCTACTAAGAAGTGTAAC

ATTTTATTTCTCCCTCTCTAAGGATTGTATAAGAACCTCTTATTTTCAGACTCTACTTAAT

CCTATTTTCTATGTCTGTATGATTTTTATATTTCTAGGACAATCAAATTGGCTTGTAGAAG

CTCAAAAGCATGCTCACAAAGTAGGTACTTATGTAGGGAACTTCGTACTCTAATAATAACT

GGTTATTAACGTTATAATTAAATGCAAAATTTGATAAGTAGTAGGGTTTGGTAAGTAATAT

AACAACACCATGTTGGCTTGGGATTCTGAATTGTGTTATTAGAGTACAACTCTTAAATAAT

GATCGTAATGTAAATAATTTAAATTTGAAGTTACTAGATAAAAAGATAATATTATGTAATA

TATTATCAAATATGTACTAAAAATGTAATTTTCATCTATCTCAATTGAAAAAATATCTGAG

TTTTGAGTCTATTGTCATGGACGA

>HM020

AAAAAAATAAAGGTTCTATACTCATTAAACAAAGTGCCAAATTCAGAGATACTACTTCCT

TTGGATAATTTGTATCGTCACCAACGATCTTTGAGTTATTTCAAAAATCACATTCTCAATT

TTGTGGTCCAACATTCACAAAATGGTTTGTCGGGCTTTAAGTCTAGTAATTGTGAAACATG

CATATGCATGAGGTAATTAATTGATAATCTGAAAAGGTTAAATAGTTAATCAATTGTACTT

TGGTTCTTTTTAATTTGTTCTCTATAATTTTTCAGTCAAAATTGAATTTGCTTTAACCATT

TAGTGTAGTAGTATGATATTACTTGCTCTACATTCTTGGATCTAAAGGTCTAATTAGTATC

GTATAAAAAATATTCTAGTTAATTAAGATACTTTTGTTCATTCTAATTATTCTTGGATTAC

TTGTGAAACTTTTAGGGATGAACAAGAAAGTCCACTAAAGAGGCTTCTTTTTGCTAGGAGT

CACTAGTTAGTTTTTTTTTTTTTTGACAAAAGTTAACCATTTTTTTTTATCGCATGGTAGG

AAAAAAAAAATAGAGATTTCCTTTGGTACTCATAAAAAAAAAAGTTGGATGGATAATGGAT

TAATCATAACTTATTTAACATAAAATGAACTAAGAGAACATTTCATATTTGGGTTTATTTT

GTAACATAAATATTAATATCAATGTTTGAAAGAGTTTTTAACAATAATTTGTAAAAGAAAG

CATAATATATCTATCAAAATAATTATAAAAGAAAAAATTTCTTTTGGTGGTATGACATGGA

AATATAATTAATTCTTATTAGATGAAAGTGTAAAATTAATTTGCATTAACAAAGCATATTT

CATTAAACTCCTTTTAGTTTTTGTTGGAAAAAATAAATTTATTGTTACTCAAAAAATAAAA

GTGTACAATTAATATATTTACTCAAAAGTCCCCTAAATGGGATGTAATTATCAAGGGATGT

GAGCGGTTTAACTTTAAGTAATGAATCAACTGTTGCATAATCACTTTGAAAGAAACTTGAG

ATTCTTCAATTAGCAAAATCTTGAATAGAATAATGAGATTATTCCTAATGCTAATGATACC

CACCTCTCCTATAAGTTGATGGGGCAGAACAAATTATGCACTCAGGACAACTTTTCTCAAA

TTTTGAAAACTTACCACACGAAGAACACACTTCCACTTGCACAAGTAAGAATATAGTATAA

CAATAAATTTACCACTCATAGCTCAATGGATATTAAAATATTGCTAATCTTTTATAGACAG

ATGTTGGCAAATTAGTATAAATTAGTTATTATATTAGTTTTTAAGGTTCAATATACTTTTT

AAAACTCTTATTAAGTTGTTTAGCTTGCCAACCAAAGACTAAATTATCTTTATTTGTTAGG

AAATAAAAGCACTTATTTTATTTTCTCCATATTTGTTTCCTCTTTGTTCCATTACTAAAAA

AATTATAATATATTAATAACAAAAAAATTTGGGGAGGGAATGTTATTTTTAAAAAAGATGT

TTTATTTATATTTAAGTGTTTTATTGAAATTCCTCGTGAGTTTAGCTCAATTGGTATGGAC

AATACATAATATATGCAAAATTCGAGGTTCAAACCTTGAGCACCACAAAAAAAAGTGTTTT

ATTGAAAAATATCAAGAACTTGAAAACAAATTTCCTTTTTATGAACTAGATATTGTCACAT

AGACAACTTAAGAATTGACTTTTTTTTTAACACTATTGTATTTTCTGAACTCGACTTTAAC

TCGAGATCAATGAGTAAACTAAAAAAACTCGTATTATTTTATCTAAGTGTTCTTAGTGTGA

GAAAACATTATAATTGTTTGCTTAGTCTATTTCTCCCTATATTCTCACCCTCATTCTTGGT

TTGCACATAGAAAGGAGAAAAAAAAAATTAGAGAAGAGTTGAATGGAACAGAAAGAAAGAG

CAAGCAAAATAATCACTAAAAAAAATTCCCCTAAAAGAAAATCACTGAAAAATATTATCCA

CCTCTATTTTAAATAATAATAATAATGAAAACAACAATATATTTTTTTATAGATTTTCTCT

ACTAGAGGTAATTTATTTTAAACACTAAATGTCCACACCTCTAACAAAAAAAATGCATAAT

ATTATAATAGATTATTGTAAGAAAGAGTGACAAATGTTGAATTAGTTATGGAATGAAGAGT

GAGAGAGATTATACAAATAAATAAATATAAATATAAATATAAATAAAGAATAGAGAAGAAC

ATAGTACTTGAATGAAGAGATTTGGCTTGTACTTCCATTACACTTGTAAGCTTTCTACTTC

CTTCACATTCCTTCTCTTTCTCTATCTTTCCTTTTATGATATGTGTACTTTGTTATTGCTT

CATTTACTACCGACATGACTCCAACCTATTAGGGCTTTATCTGAATAATGTTAGTTATTTT

CTCTCATTTTCTTTGCTTCTTCTTATTCTTGGAACTTATAAAGGGTCTTGTCAATTCTATC

ATTCTCTTTTGTATACTTACAAATATAAATATTCTCTTTAGCTTCATTTTCATGTCTTCTT

CTACTAATAAGCTATGGAAATTGAAGTTTTTGTTTTCATGTTTATTTTTCACTTCAGCTCC

TCTTTTTTGATGTTGATGCTGAAGATTGAATAAAAACTGAGGTGGTATGTATGAGAACAAG

AACATTTTTCATTCATCTCTGTTATTTGAACATCTAAATTTTAAACAACTATCGCGACAAT

TTTTTAAGTGGTTGAAGTATTTTAAATAGGTGGTTAATTACATTAAGTAGATAGTTAATTG

ATATATCAGGTGTCAAAACTCATTAACCATCAACATAACTTCATTAGCCATAAATTTTTGA

CGTGATTAACCAAATATTGTAGATGCTCAATTACATGTAGATTTGATGGAGTTGTCCACAA

TTTCTCATATATATATTAACTAATAACCATCTACCGAATATAATTAACCATATATTAGAAT

ATCATTAACCTCTAAAACAGTTGTCGCGAAAATTGGTTGTAAAAAAATCATTTCTATTTTT

TACGCCACTAGTTCTCTTGTTATTTCTCTTTGTTGGAAAGTTGTTGTTTTTAACTTTGAGC

TTAGTCTTATTAATTAACTTGTAATGGTAACAACATTTTTCTATCTTTGTTTGAAGGAAGT

AGTTACTGTGTCAACTTTAGTAATTAATTTGGATCATTTTGGAGGCAAAATATGGCTATGC

AACCTGTTTATTTTAAAGAACATGAAGGAAATGTCCACAATTCTGTTGGACAGTTTTCATC

TGTGACTTCAGCACCATGGTGGAGTAATGCCTATGGATCTCAACCTTTTTATGGAGGAGAC

TCTTGTGGCCAAATGAAACCTTTTTCACTAGAGCTTTCCAACTACATAGACCAACTTGCTC

CGAGTAAGAACTTAGTTCGAGGAGTTGAACAATTGTTTGATAAAGGGCATACAAACCAATT

CACCATCTTTCCAGGTACTTGTTATTCAATATAATTCCGGTTTTGAATGAATTGATTTTTG

TTAAGTTGGTTCTGGATAAACGTGCGTTAAATATAATATGATTTATGTTTGGATACGTTTA

TGTTAAAGTGAGTTGAACATAAATTGGAGACTAAATATCAATTGCAGAGGCAAAAGCTTCA

AATTCTAATTTCAAGTTAGAATCAAATCAATTCTACTCGTAAGCATCTAAATGTTTCAAAA

CCAATTCTACATCTCTAGAACTAAATTTGCATTCAAACATTTTCTGCTACAGATGATTGTA

AGATGTCAGTTGATACACCAAATCATCAAGCAACCTTATCCCTGCAATCACCATTTGCTGC

CGAGCCACTTAATCGATTTGAGCTAGGTTTTAACCAGTCTATGGTAAATCTTCTTATTAAA

GCAATCCCTTTGATCATGTTTTAGTATTTTACTTTTGATGTACATAAATTCATTATGTTTT

TATTATCCTTTCCATGCTAGATCTGTGCAAAATATCCGTATATAGATCAATTTTACGGGCT

CTTCTCGACTTATGGACCTCAAATCTCGGTTTGTCTTCACCTCGGCTATTTTATTAATCTT

TGTATATATCATGCAATGTCTATAAGTACTTGAATGTGAATTTGTTTTATTTACAGTGTGT

ATGCTTACATGAGAAAGCCAGAAATCTGTTTACAACTATCTTGTTAGTATTCTTCTAAATC

TTTCAAATGTAGAGTTGATTATGAAATTTGTTGTTAATGAGTTTTCGGCTTTATGAATGAA

TTGTAAGACAAGTAATGAAACAAAAACTAGAAGAAAATTGAATTATATGATGCAATCTGTG

TTGAAGTTTTTGCATGTATTCAAGTTCTGCTTTTAGTTACTATTTGTTTATCTGCTGCAAT

ATTTCATCAATTATTATTCATGTATGTACCAATATTTAATAAATTAGGGGCGAATTATGCT

TCCGCTTAGCATGACATCTGACGACGGACCAACATACGTGAATGCTAAGCAATACCATGGA

ATCATCAGACGCAGGCATTCTCGTGCCAAAGCTGTGCTTCAGAATAAATTGATAAAGCGTA

ACAAGGTATGAAACTGAGTATTTTTCCTCACAACAATGTTCGAAAACTTGTGAAATAGTAT

ATTTTTCCTTTTACCCTTTTTATACCTATATTAAGGTTTTGTATTTGTCTTGCAGCCATAT

ATGCACGAATCGCGTCATCTACATGCAATGCGTAGACCAAGAGGATGCGGTGGTCGTTTCT

TGAACACAAAAGTTTCTGCTAATGGAAACGGTAAAAGCGGGAGTGAAGAGAACGGAAACAT

TGGTGGCCTACAGCTGCAGTCCAGTGGTTCTCAGAGTTCCGAAGTCTTACAATCTGAGGTT

GGAACTTTAAATTCGTCGAAGGAGACAAACGGAGGCAGTCCAAATGTCTCGGGGTCAGAGG

TGACTAGCATGTATACACAGGGAGGTCTTGATAGCTTTACTGTCAATCATATCGGATCTAC

TGTCCACTCTTTGGGAGACATGATCGATACTGGACACGGTATCGTCATGCCAACAAAATGG

TTTGCAGCAGCTGGCAGACAGCTGCTGGAACCATAAGTTTCGATTCAGAAAGGAAACAAGT

GGGTTTGGTACAATGTGAAATATTTTGCACCAAACTCATCCTTTCCGAGACCAGATGAAGA

AGCTATGTTTCAGTTTGTTGTGTTTACTACGACAAATTTAGTTTCGGAAGACTACTTTTCC

ATCTGGTGCTCAGGCAACTCATTCTTGGCTTATTCTCAGGAAACTCATCCTTGGCTCGTAA

TATTTAGTAGTATTGTCATTGTCTTCCCGCGCAGGCTTTCCGTGGCATGGTAGGCATGCTA

ATGACTTTGGTATTTTCATGCAGTTATAACTATGATGTGTCTTTGTTTGTTGTTAAAATAA

AAAACATGAACTCTAGCTAGGTGCATGTGTGTGTTTTTAATCTTGTCTACTAAGTTTGGTG

TTTTGTAATGGATTTCTGACTTTATGGAGCAATGTATTGTAACTCTACTAAGAAGTGTAAC

ATTTTATTTCTCCCTCTCTAAGGATTGTATAAGAACCTCTTATTTTCAGACTCTACTTAAT

CCTATTTTCTATGTCTGTATGATTTTTATATTTCTAGGACAATCAAATTGGCTTGTAGAAG

CTCAAAAGCATGCTCACAAAGTAGGTACTTATGTAGGGAACTTCGTACTCTAATAATAACT

GGTTATTAACGTTATAATTAAATGCAAAATTTGATAAGTAGTAGGGTTTGGTAAGTAATAT

AACAACACCATGTTGGCTTGGGATTCTGAATTGTGTTATTAGAGTACAACTCTTAAATAAT

GATCGTAATGTAAATAATTTAAATTTGAAGTTACTAGATAAAAAGATAACATTATGTAATA

TATTATCAAATATGTACTAAAAATGTAATTTTCATCTATCTCAATTGAAAAAATATCTGAG

TTTTGAGTCTATTGTCATGGACGA

>HM021

AAAAAAATAAAGGTTCTATACTCATTAAACAAAGTGCCAAATTCAGAGATACTACTTCCT

TTGGATAATTTGTATCGTCACCAACGATCTTTGAGTTATTTCAAAAATCACATTCTCAATT

TTGTGGTCCAACATTCACAAAATGGTTTGTCGGGCTTTAAGTCTAGTAATTGTGAAACATG

CATATGCATGAGGTAATTAATTGATAATCTGAAAAGGTTAAATAGTTAATCAATTGTACTT

TGGTTATTTTTAATTTGTTCTCTATAATTTTTCAGTCAAAATTGAATTTGCTTTAACCATT

TAGTGTAGTAGTATGATATTACTTGCTCTACATTCTTGGATCTAAAGGTCTAATTAGTATC

GTATAAAAAATATTCTAGTTAATTAAGATACTTTTGTTCATTCTAATTATTCTTGGATTAC

TTGTGAAACTTTTAGGGATGAACAAGAAAGTCCACTAAAGAGGCTTCTTTTTGCTAGGAGT

CACTAGTTAGTTTTTTTTTTTTTTGACAAAAGTTAACCATTTTTTTTTATCGCATGGTAGG

AAAAAAAAAATAGAGATTTCCTTTGGTACTCATAAAAAAAAAAGTTGGATGGATAATGGAT

TAATCATAACTTATTTAACATAAAATGAACTAAGAGAACATTTCATATTTGGGTTTATTTT

GTAACATAAATATTAATATCAATGTTTGAAAGAGTTTTTAACAATAATTTGTAAAAGAAAG

CATAATATATCTATCAAAATAATTATAAAAGAAAAAATTTCTTTTGGTGGTATGACATGGA

AATATAATTAATTCTTATTAGATGAAAGTGTAAAATTAATTTGCATTAACAAAGCATATTT

CATTAAACTCCTTTTAGTTTTTGTTGGAAAAAATAAATTTATTGTTACTCAAAAAATAAAA

GTGTACAATTAATATATTTACTCAAAAGTCCCCTAAATGGGATGTAATTATCAAGGGATGT

GAGCGGTTTAACTTTAAGTAATGAATCAACTGTTGCATAATCACTTTGAAAGAAACTTGAG

ATTCTTCAATTAGCAAAATCTTGAATAGAATAATGAGATTATTCCTAATGCTAATGATACC

CACCTCTCCTATAAGTTGATGGGGCAGAACAAATTATGCACTCAGGACAACTTTTCTCAAA

TTTTGAAAACTTACCACACGAAGAACACACTTCCACTTGCACAAGTAAGAATATAGTATAA

CAATAAATTTACCACTCATAGCTCAATGGATATTAAAATATTGCTAATCTTTTATAGACAG

ATGTTGGCAAATTAGTATAAATTAGTTATTATATTAGTTTTTAAGGTTCAATATACTTTTT

AAAACTCTTATTAAGTTGTTTAGCTTGCCAACCAAAGACTAAATTATCTTTATTTGTTAGG

AAATAAAAGCACTTATTTTATTTTCTCCATATTTGTTTCCTCTTTGTTCCATTACTAAAAA

AATTATAATATATTAATAACAAAAAAATTTGGGGAGGGAATGTTATTTTTAAAAAAGATGT

TTTATTTATATTTAAGTGTTTTATTGAAATTCCTCGTGAGTTTAGCTCAATTGGTATGGAC

AATACATAATATATGCAAAATTCGAGGTTCAAACCTTGAGCACCACAAAAAAAAGTGTTTT

ATTGAAAAATATCAAGAACTTGAAAACAAATTTCCTTTTTATGAACTAGATATTGTCACAT

AGACAACTTAAGAATTGACTTTTTTTTTAACACTATTGTATTTTCTGAACTCGACTTTAAC

TCGAGATCAATGAGTAAACTAAAAAAACTCGTATTATTTTATCTAAGTGTTCTTAGTGTGA

GAAAACATTATAATTGTTTGCTTAGTCTATTTCTCCCTATATTCTCACCCTCATTCTTGGT

TTGCACATAGAAAGGAGAAAAAAAAAATTAGAGAAGAGTTGAATGGAACAGAAAGAAAGAG

CAAGCAAAATAATCACTAAAAAAAATTCCCCTAAAAGAAAATCACTGAAAAATATTATCCA

CCTCTATTTTAAATAATAATAATAATGAAAACAACAATATATTTTTTTATAGATTTTCTCT

ACTAGAGGTAATTTATTTTAAACACTAAATGTCCACACCTCTAACAAAAAAAATGCATAAT

ATTATAATAGATTATTGTAAGAAAGAGTGACAAATGTTGAATTAGTTATGGAATGAAGAGT

GAGAGAGATTATACAAATAAATAAATATAAATATAAATATAAATAAAGAATAGAGAAGAAC

ATAGTACTTGAATGAAGAGATTTGGCTTGTACTTCCATTACACTTGTAAGCTTTCTACTTC

CTTCACATTCCTTCTCTTTCTCTATCTTTCCTTTTATGATATGTGTACTTTGTTATTGCTT

CATTTACTACCGACATGACTCCAACCTATTAGGGCTTTATCTGAATAATGTTAGTTATTTT

CTCTCATTTTCTTTGCTTCTTCTTATTCTTGGAACTTATAAAGGGTCTTGTCAATTCTATC

ATTCTCTTTTGTATACTTACAAATATAAATATTCTCTTTAGCTTCATTTTCATGTCTTCTT

CTACTAATAAGCTATGGAAATTGAAGTTTTTGTTTTCATGTTTATTTTTCACTTCAGCTCC

TCTTTTTTGATGTTGATGCTGAAGATTGAATAAAAACTGAGGTGGTATGTATGAGAACAAG

AACATTTTTCATTCATCTCTGTTATTTGAACATCTAAATTTTAAACAACTATCGCGACAAT

TTTTTAAGTGGTTGAAGTATTTTAAATAGGTGGTTAATTACATTAAGTAGATAGTTAATTG

ATATATCAGGTGTCAAAACTCATTAACCATCAACATAACTTCATTAGCCATAAATTTTTGA

CGTGATTAACCAAATATTGTAGATGCTCAATTACATGTAGATTTGATGGAGTTGTCCACAA

TTTCTCATATATATATTAACTAATAACCATCTACCGAATATAATTAACCATATATTAGAAT

ATCATTAACCTCTAAAACAGTTGTCGCGAAAATTGGTTGTAAAAAAATCATTTCTATTTTT

TACGCCACTAGTTCTCTTGTTATTTCTCTTTGTTGGAAAGTTGTTGTTTTTAACTTTGAGC

TTAGTCTTATTAATTAACTTGTAATGGTAACAACATTTTTCTATCTTTGTTTGAAGGAAGT

AGTTACTGTGTCAACTTTAGTAATTAATTTGGATCATTTTGGAGGCAAAATATGGCTATGC

AACCTGTTTATTTTAAAGAACATGAAGGAAATGTCCACAATTCTGTTGGACAGTTTTCATC

TGTGACTTCAGCACCATGGTGGAGTAATGCCTATGGATCTCAACCTTTTTATGGAGGAGAC

TCTTGTGGCCAAATGAAACCTTTTTCACTAGAGCTTTCCAACTACATAGACCAACTTGCTC

CGAGTAAGAACTTAGTTCGAGGAGTTGAACAATTGTTTGATAAAGGGCATACAAACCAATT

CACCATCTTTCCAGGTACTTGTTATTCAATATAATTCCGGTTTTGAATGAATTGATTTTTG

TTAAGTTGGTTCTGGATAAACGTGCGTTAAATATAATATGATTTATGTTTGGATACGTTTA

TGTTAAAGTGAGTTGAACATAAATTGGAGACTAAATATCAATTGCAGAGGCAAAAGCTTCA

AATTCTAATTTCAAGTTAGAATCAAATCAATTCTACTCGTAAGCATCTAAATGTTTCAAAA

CCAATTCTACATCTCTAGAACTAAATTTGCATTCAAACATTTTCTGCTACAGATGATTGTA

AGATGTCAGTTGATACACCAAATCATCAAGCAACCTTATCCCTGCAATCACCATTTGCTGC

CGAGCCACTTAATCGATTTGAGCTAGGTTTTAACCAGTCTATGGTAAATCTTCTTATTAAA

GCAATCCCTTTGATCATGTTTTAGTATTTTACTTTTGATGTACATAAATTCATTATGTTTT

TATTATCCTTTCCATGCTAGATCTGTGCAAAATATCCGTATATAGATCAATTTTACGGGCT

CTTCTCGACTTATGGACCTCAAATCTCGGTTTGTCTTCACCTCGGCTATTTTATTAATCTT

TGTATATATCATGCAATGTCTATAAGTACTTGAATGTGAATTTGTTTTATTTACAGTGTGT

ATGCTTACATGAGAAAGCCAGAAATCTGTTTACAACTATCTTGTTAGTATTCTTCTAAATC

TTTCAAATGTAGAGTTGATTATGAAATTTGTTGTTAATGAGTTTTCGGCTTTATGAATGAA

TTGTAAGACAAGTAATGAAACAAAAACTAGAAGAAAATTGAATTATATGATGCAATCTGTG

TTGAAGTTTTTGCATGTATTCAAGTTCTGCTTTTAGTTACTATTTGTTTATCTGCTGCAAT

ATTTCATCAATTATTATTCATGTATGTACCAATATTTAATAAATTAGGGGCGAATTATGCT

TCCGCTTAGCATGACATCTGACGACGGACCAACATACGTGAATGCTAAGCAATACCATGGA

ATCATCAGACGCAGGCATTCTCGTGCCAAAGCTGTGCTTCAGAATAAATTGATAAAGCGTA

ACAAGGTATGAAACTGAGTATTTTTCCTCACAACAATGTTCGAAAACTTGTGAAATAGTAT

ATTTTTCCTTTTACCCTTTTTATACCTATATTAAGGTTTTGTATTTGTCTTGCAGCCATAT

ATGCACGAATCGCGTCATCTACATGCAATGCGTAGACCAAGAGGATGCGGTGGTCGTTTCT

TGAACACAAAAGTTTCTGCTAATGGAAACGGTAAAAGCGGGAGTGAAGAGAACGGAAACAT

TGGTGGCCTACAGCTGCAGTCCAGTGGTTCTCAGAGTTCCGAAGTCTTACAATCTGAGGTT

GGAACTTTAAATTCGTCGAAGGAGACAAACGGAGGCAGTCCAAATGTCTCGGGGTCAGAGG

TGACTAGCATGTATACACAGGGAGGTCTTGATAGCTTTACTGTCAATCATATCGGATCTAC

TGTCCACTCTTTGGGAGACATGATCGATACTGGACACGGTATCGTCATGCCAACAAAATGG

TTTGCAGCAGCTGGCAGACAGCTGCTGGAACCATAAGTTTCGATTCAGAAAGGAAACAAGT

GGGTTTGGTACAATGTGAAATATTTTGCACCAAACTCATCCTTTCCGAGACCAGATGAAGA

AGCTATGTTTCAGTTTGTTGTGTTTACTACGACAAATTTAGTTTCGGAAGACTACTTTTCC

ATCTGGTGCTCAGGCAACTCATTCTTGGCTTATTCTCAGGAAACTCATCCTTGGCTCGTAA

TATTTAGTAGTATTGTCATTGTCTTCCCGCGCAGGCTTTCCGTGGCATGGTAGGCATGCTA

ATGACTTTGGTATTTTCATGCAGTTATAACTATGATGTGTCTTTGTTTGTTGTTAAAATAA

AAAACATGAACTCTAGCTAGGTGCATGTGTGTGTTTTTAATCTTGTCTACTAAGTTTGGTG

TTTTGTAATGGATTTCTGACTTTATGGAGCAATGTATTGTAACTCTACTAAGAAGTGTAAC

ATTTTATTTCTCCCTCTCTAAGGATTGTATAAGAACCTCTTATTTTCAGACTCTACTTAAT

CCTATTTTCTATGTCTGTATGATTTTTATATTTCTAGGACAATCAAATTGGCTTGTAGAAG

CTCAAAAGCATGCTCACAAAGTAGGTACTTATGTAGGGAACTTCGTACTCTAATAATAACT

GGTTATTAACGTTATAATTAAATGCAAAATTTGATAAGTAGTAGGGTTTGGTAAGTAATAT

AACAACACCATGTTGGCTTGGGATTCTGAATTGTGTTATTAGAGTACAACTCTTAAATAAT

GATCGTGATGTAAATAATTTAAATTTGAAGTTACTAGATAAAAAGATAACATTATGTAATA

TATTATCAAATATGTACTAAAAATGTAATTTTCATCTATCTCAATTGAAAAAATATCTGAG

TTTTGAGTCTATTGTCATGGACGA

>HM023

AAAAAAATAAAGGTTCTATACTCATTAAACAAAGTGCCAAATTCAGAGATACTACTTCCT

TTGGATAATTTGTATCGTCACCAACGATCTTTGAGTTATTTCAAAAATCACATTCTCAATT

TTGTGGTCCAACATTCACAAAATGGTTTGTCGGGCTTTAAGTCTAGTAATTGTGAAACATG

CATATGCATGAGGTAATTAATTGATAATCTGAAAAGGTTAAATAGTTAATCAATTGTACTT

TGGTTATTTTTAATTTGTTCTCTATAATTTTTCAGTCAAAATTGAATTTGCTTTAACCATT

TAGTGTAGTAGTATGATATTACTTGCTCTACATTCTTGGATCTAAAGGTCTAATTAGTATC

GTATAAAAAATATTCTAGTTAATTAAGATACTTTTGTTCATTCTAATTATTCTTGGATTAC

TTGTGAAACTTTTAGGGATGAACAAGAAAGTCCACTAAAGAGGCTTCTTTTTGCTAGGAGT

CACTAGTTAGTTTTTTTTTTTTTTGACAAAAGTTAACCATTTTTTTTTATCGCATGGTAGG

AAAAAAAAAATAGAGATTTCCTTTGGTACTCATAAAAAAAAAAGTTGGATGGATAATGGAT

TAATCATAACTTATTTAACATAAAATGAACTAAGAGAACATTTCATATTTGGGTTTATTTT

GTAACATAAATATTAATATCAATGTTTGAAAGAGTTTTTAACAATAATTTGTAAAAGAAAG

CATAATATATCTATCAAAATAATTATAAAAGAAAAAATTTCTTTTGGTGGTATGACATGGA

AATATAATTAATTCTTATTAGATGAAAGTGTAAAATTAATTTGCATTAACAAAGCATATTT

CATTAAACTCCTTTTAGTTTTTGTTGGAAAAAATAAATTTATTGTTACTCAAAAAATAAAA

GTGTACAATTAATATATTTACTCAAAAGTCCCCTAAATGGGATGTAATTATCAAGGGATGT

GAGCGGTTTAACTTTAAGTAATGAATCAACTGTTGCATAATCACTTTGAAAGAAACTTGAG

ATTCTTCAATTAGCAAAATCTTGAATAGAATAATGAGATTATTCCTAATGCTAATGATACC

CACCTCTCCTATAAGTTGATGGGGCAGAACAAATTATGCACTCAGGACAACTTTTCTCAAA

TTTTGAAAACTTACCACACGAAGAACACACTTCCACTTGCACAAGTAAGAATATAGTATAA

CAATAAATTTACCACTCATAGCTCAATGGATATTAAAATATTGCTAATCTTTTATAGACAG

ATGTTGGCAAATTAGTATAAATTAGTTATTATATTAGTTTTTAAGGTTCAATATACTTTTT

AAAACTCTTATTAAGTTGTTTAGCTTGCCAACCAAAGACTAAATTATCTTTATTTGTTAGG

AAATAAAAGCACTTATTTTATTTTCTCCATATTTGTTTCCTCTTTGTTCCATTACTAAAAA

AATTATAATATATTAATAACAAAAAAATTTGGGGAGGGAATGTTATTTTTAAAAAAGATGT

TTTATTTATATTTAAGTGTTTTATTGAAATTCCTCGTGAGTTTAGCTCAATTGGTATGGAC

AATACATAATATATGCAAAATTCGAGGTTCAAACCTTGAGCACCACAAAAAAAAGTGTTTT

ATTGAAAAATATCAAGAACTTGAAAACAAATTTCCTTTTTATGAACTAGATATTGTCACAT

AGACAACTTAAGAATTGACTTTTTTTTTAACACTATTGTATTTTCTGAACTCGACTTTAAC

TCGAGATCAATGAGTAAACTAAAAAAACTCGTATTATTTTATCTAAGTGTTCTTAGTGTGA

GAAAACATTATAATTGTTTGCTTAGTCTATTTCTCCCTATATTCTCACCCTCATTCTTGGT

TTGCACATAGAAAGGAGAAAAAAAAAATTAGAGAAGAGTTGAATGGAACAGAAAGAAAGAG

CAAGCAAAATAATCACTAAAAAAAATTCCCCTAAAAGAAAATCACTGAAAAATATTATCCA

CCTCTATTTTAAATAATAATAATAATGAAAACAACAATATATTTTTTTATAGATTTTCTCT

ACTAGAGGTAATTTATTTTAAACACTAAATGTCCACACCTCTAACAAAAAAAATGCATAAT

ATTATAATAGATTATTGTAAGAAAGAGTGACAAATGTTGAATTAGTTATGGAATGAAGAGT

GAGAGAGATTATACAAATAAATAAATATAAATATAAATATAAATAAAGAATAGAGAAGAAC

ATAGTACTTGAATGAAGAGATTTGGCTTGTACTTCCATTACACTTGTAAGCTTTCTACTTC

CTTCACATTCCTTCTCTTTCTCTATCTTTCCTTTTATGATATGTGTACTTTGTTATTGCTT

CATTTACTACCGACATGACTCCAACCTATTAGGGCTTTATCTGAATAATGTTAGTTATTTT

CTCTCATTTTCTTTGCTTCTTCTTATTCTTGGAACTTATAAAGGGTCTTGTCAATTCTATC

ATTCTCTTTTGTATACTTACAAATATAAATATTCTCTTTAGCTTCATTTTCATGTCTTCTT

CTACTAATAAGCTATGGAAATTGAAGTTTTTGTTTTCATGTTTATTTTTCACTTCAGCTCC

TCTTTTTTGATGTTGATGCTGAAGATTGAATAAAAACTGAGGTGGTATGTATGAGAACAAG

AACATTTTTCATTCATCTCTGTTATTTGAACATCTAAATTTTAAACAACTATCGCGACAAT

TTTTTAAGTGGTTGAAGTATTTTAAATAGGTGGTTAATTACATTAAGTAGATAGTTAATTG

ATATATCAGGTGTCAAAACTCATTAACCATCAACATAACTTCATTAGCCATAAATTTTTGA

CGTGATTAACCAAATATTGTAGATGCTCAATTACATGTAGATTTGATGGAGTTGTCCACAA

TTTCTCATATATATATTAACTAATAACCATCTACCGAATATAATTAACCATATATTAGAAT

ATCATTAACCTCTAAAACAGTTGTCGCGAAAATTGGTTGTAAAAAAATCATTTCTATTTTT

TACGCCACTAGTTCTCTTGTTATTTCTCTTTGTTGGAAAGTTGTTGTTTTTAACTTTGAGC

TTAGTCTTATTAATTAACTTGTAATGGTAACAACATTTTTCTATCTTTGTTTGAAGGAAGT

AGTTACTGTGTCAACTTTAGTAATTAATTTGGATCATTTTGGAGGCAAAATATGGCTATGC

AACCTGTTTATTTTAAAGAACATGAAGGAAATGTCCACAATTCTGTTGGACAGTTTTCATC

TGTGACTTCAGCACCATGGTGGAGTAATGCCTATGGATCTCAACCTTTTTATGGAGGAGAC

TCTTGTGGCCAAATGAAACCTTTTTCACTAGAGCTTTCCAACTACATAGACCAACTTGCTC

CGAGTAAGAACTTAGTTCGAGGAGTTGAACAATTGTTTGATAAAGGGCATACAAACCAATT

CACCATCTTTCCAGGTACTTGTTATTCAATATAATTCCGGTTTTGAATGAATTGATTTTTG

TTAAGTTGGTTCTGGATAAACGTGCGTTAAATATAATATGATTTATGTTTGGATACGTTTA

TGTTAAAGTGAGTTGAACATAAATTGGAGACTAAATATCAATTGCAGAGGCAAAAGCTTCA

AATTCTAATTTCAAGTTAGAATCAAATCAATTCTACTCGTAAGCATCTAAATGTTTCAAAA

CCAATTCTACATCTCTAGAACTAAATTTGCATTCAAACATTTTCTGCTACAGATGATTGTA

AGATGTCAGTTGATACACCAAATCATCAAGCAACCTTATCCCTGCAATCACCATTTGCTGC

CGAGCCACTTAATCGATTTGAGCTAGGTTTTAACCAGTCTATGGTAAATCTTCTTATTAAA

GCAATCCCTTTGATCATGTTTTAGTATTTTACTTTTGATGTACATAAATTCATTATGTTTT

TATTATCCTTTCCATGCTAGATCTGTGCAAAATATCCGTATATAGATCAATTTTACGGGCT

CTTCTCGACTTATGGACCTCAAATCTCGGTTTGTCTTCACCTCGGCTATTTTATTAATCTT

TGTATATATCATGCAATGTCTATAAGTACTTGAATGTGAATTTGTTTTATTTACAGTGTGT

ATGCTTACATGAGAAAGCCAGAAATCTGTTTACAACTATCTTGTTAGTATTCTTCTAAATC

TTTCAAATGTAGAGTTGATTATGAAATTTGTTGTTAATGAGTTTTCGGCTTTATGAATGAA

TTGTAAGACAAGTAATGAAACAAAAACTAGAAGAAAATTGAATTATATGATGCAATCTGTG

TTGAAGTTTTTGCATGTATTCAAGTTCTGCTTTTAGTTACTATTTGTTTATCTGCTGCAAT

ATTTCATCAATTATTATTCATGTATGTACCAATATTTAATAAATTAGGGGCGAATTATGCT

TCCGCTTAGCATGACATCTGACGACGGACCAACATACGTGAATGCTAAGCAATACCATGGA

ATCATCAGACGCAGGCATTCTCGTGCCAAAGCTGTGCTTCAGAATAAATTGATAAAGCGTA

ACAAGGTATGAAACTGAGTATTTTTCCTCACAACAATGTTCGAAAACTTGTGAAATAGTAT

ATTTTTCCTTTTACCCTTTTTATACCTATATTAAGGTTTTGTATTTGTCTTGCAGCCATAT

ATGCACGAATCGCGTCATCTACATGCAATGCGTAGACCAAGAGGATGCGGTGGTCGTTTCT

TGAACACAAAAGTTTCTGCTAATGGAAACGGTAAAAGCGGGAGTGAAGAGAACGGAAACAT

TGGTGGCCTACAGCTGCAGTCCAGTGGTTCTCAGAGTTCCGAAGTCTTACAATCTGAGGTT

GGAACTTTAAATTCGTCGAAGGAGACAAACGGAGGCAGTCCAAATGTCTCGGGGTCAGAGG

TGACTAGCATGTATACACAGGGAGGTCTTGATAGCTTTACTGTCAATCATATCGGATCTAC

TGTCCACTCTTTGGGAGACATGATCGATACTGGACACGGTATCGTCATGCCAACAAAATGG

TTTGCAGCAGCTGGCAGACAGCTGCTGGAACCATAAGTTTCGATTCAGAAAGGAAACAAGT

GGGTTTGGTACAATGTGAAATATTTTGCACCAAACTCATCCTTTCCGAGACCAGATGAAGA

AGCTATGTTTCAGTTTGTTGTGTTTACTACGACAAATTTAGTTTCGGAAGACTACTTTTCC

ATCTGGTGCTCAGGCAACTCATTCTTGGCTTATTCTCAGGAAACTCATCCTTGGCTCGTAA

TATTTAGTAGTATTGTCATTGTCTTCCCGCGCAGGCTTTCCGTGGCATGGTAGGCATGCTA

ATGACTTTGGTATTTTCATGCAGTTATAACTATGATGTGTCTTTGTTTGTTGTTAAAATAA

AAAACATGAACTCTAGCTAGGTGCATGTGTGTGTTTTTAATCTTGTCTACTAAGTTTGGTG

TTTTGTAATGGATTTCTGACTTTATGGAGCAATGTATTGTAACTCTACTAAGAAGTGTAAC

ATTTTATTTCTCCCTCTCTAAGGATTGTATAAGAACCTCTTATTTTCAGACTCTACTTAAT

CCTATTTTCTATGTCTGTATGATTTTTATATTTCTAGGACAATCAAATTGGCTTGTAGAAG

CTCAAAAGCATGCTCACAAAGTAGGTACTTATGTAGGGAACTTCGTACTCTAATAATAACT

GGTTATTAACGTTATAATTAAATGCAAAATTTGATAAGTAGTAGGGTTTGGTAAGTAATAT

AACAACACCATGTTGGCTTGGGATTCTGAATTGTGTTATTAGAGTACAACTCTTAAATAAT

GATCGTGATGTAAATAATTTAAATTTGAAGTTACTAGATAAAAAGATAACATTATGTAATA

TATTATCAAATATGTACTAAAAATATAATTTTCATCTATCTCAATTGAAAAAATATCTGGG

TTTTGAGTCTATTGTCATGGACGA

>HM024

AAAAAAATAAAGGTTCTATACTCATTAAACAAAGTGCCAAATTCAGAGATACTACTTCCT

TTGGATAATTTGTATCGTCACCAACGATCTTTGAGTTATTTCAAAAATCACATTCTCAATT

TTGTGGTCCAACATTCACAAAATGGTTTGTCGGGCTTTAAGTCTAGTAATTGTGAAACATG

CATATGCATGAGGTAATTAATTGATAATCTGAAAAGGTTAAATAGTTAATCAATTGTACTT

TGGTTATTTTTAATTTGTTCTCTATAATTTTTCAGTCAAAATTGAATTTGCTTTAACCATT

TAGTGTAGTAGTATGATATTACTTGCTCTACATTCTTGGATCTAAAGGTCTAATTAGTATC

GTATAAAAAATATTCTAGTTAATTAAGATACTTTTGTTCATTCTAATTATTCTTGGATTAC

TTGTGAAACTTTTAGGGATGAACAAGAAAGTCCACTAAAGAGGCTTCTTTTTGCTAGGAGT

CACTAGTTAGTTTTTTTTTTTTTTGACAAAAGTTAACCATTTTTTTTTATCGCATGGTAGG

AAAAAAAAAATAGAGATTTCCTTTGGTACTCATAAAAAAAAAAGTTGGATGGATAATGGAT

TAATCATAACTTATTTAACATAAAATGAACTAAGAGAACATTTCATATTTGGGTTTATTTT

GTAACATAAATATTAATATCAATGTTTGAAAGAGTTTTTAACAATAATTTGTAAAAGAAAG

CATAATATATCTATCAAAATAATTGTAAAAGAAAAAATTTCTTTTGGTGGTATGACATGGA

AAAATAATTAATTCTTATTAGATGAAAGTGTAAAATTAATTTGCATTAACAAAGCATATTT

CATTAAACTCCTTTTAGTTTTTGTTGGAAAAAATAAATTTATTGTTACTCAAAAAATAAAA

GTGTACAATTAATATATTTACTCAAAAGTCCCCTAAATGGGATGTAATTATCAAGGGATGT

GAGCGGTTTAACTTTAAGTAATGAATCAACTGTTGCATAATCACTTTGAAAGAAACTTGAG

ATTCTTCAATTAGCAAAATCTTGAATAGAATAATGAGATTATTCCTAATGCTAATGATACC

CACCTCTCCTATAAGTTGATGGGGCAGAACAAATTATGCACTCAGGACAACTTTTCTCAAA

TTTTGAAAACTTACCACACGAAGAACACACTTCCACTTGCACAAGTAAGAATATAGTATAA

CAATAAATTTACCACTCATAGCTCAATGGATATTAAAATATTGCTAATCTTTTATAGACAG

ATGTTGGCAAATTAGTATAAATTAGTTATTATATTAGTTTTTAAGGTTCAATATACTTTTT

AAAACTCTTATTAAGTTGTTTAGCTTGCCAACCAAAGACTAAATTATCTTTATTTGTTAGG

AAATAAAAGCACTTATTTTATTTTCTCCATATTTGTTTCCTCTTTGTTCCATTACTAAAAA

AATTATAATATATTAATAACAAAAAAATTTGGGGAGGGAATGTTATTTTTAAAAAAGATGT

TTTATTTATATTTAAGTGTTTTATTGAAATTCCTCGTGAGTTTAGCTCAATTGGTATGGAC

AATACATAATATATGCAAAATTCGAGGTTCAAACCTTGAGCACCACAAAAAAAAGTGTTTT

ATTGAAAAATATCAAGAACTTGAAAACAAATTTCCTTTTTATGAACTAGATATTGTCACAT

AGACAACTTAAGAATTGACTTTTTTTTTAACACTATTGTATTTTCTGAACTCGACTTTAAC

TCGAGATCAATGAGTAAACTAAAAAAACTCGTATTATTTTATCTAAGTGTTCTTAGTGTGA

GAAAACATTATAATTGTTTGCTTAGTCTATTTCTCCCTATATTCTCACCCTCATTCTTGGT

TTGCACATAGAAAGGAGAAAAAAAAAATTAGAGAAGAGTTGAATGGAACAGAAAGAAAGAG

CAAGCAAAATAATCACTAAAAAAAATTCCCCTAAAAGAAAATCACTGAAAAATATTATCCA

CCTCTATTTTAAATAATAATAATAATGAAAACAACAATATATTTTTTTATAGATTTTCTCT

ACTAGAGGTAATTTATTTTAAACACTAAATGTCCACACCTCTAACAAAAAAAATGCATAAT

ATTATAATAGATTATTGTAAGAAAGAGTGACAAATGTTGAATTAGTTATGGAATGAAGAGT

GAGAGAGATTATACAAATAAATAAATATAAATATAAATATAAATAAAGAATAGAGAAGAAC

ATAGTACTTGAATGAAGAGATTTGGCTTGTACTTCCATTACACTTGTAAGCTTTCTACTTC

CTTCACATTCCTTCTCTTTCTCTATCTTTCCTTTTATGATATGTGTACTTTGTTATTGCTT

CATTTACTACCGACATGACTCCAACCTATTAGGGCTTTATCTGAATAATGTTAGTTATTTT

CTCTCATTTTCTTTGCTTCTTCTTATTCTTGGAACTTATAAAGGGTCTTGTCAATTCTATC

ATTCTCTTTTGTATACTTACAAATATAAATATTCTCTTTAGCTTCATTTTCATGTCTTCTT

CTACTAATAAGCTATGGAAATTGAAGTTTTTGTTTTCATGTTTATTTTTCACTTCAGCTCC

TCTTTTTTGATGTTGATGCTGAAGATTGAATAAAAACTGAGGTGGTATGTATGAGAACAAG

AACATTTTTCATTCATCTCTGTTATTTGAACATCTAAATTTTAAACAACTATCGCGACAAT

TTTTTAAGTGGTTGAAGTATTTTAAATAGGTGGTTAATTACATTAAGTAGATAGTTAATTG

ATATATCAGGTGTCAAAACTCATTAACCATCAACATAACTTCATTAGCCATAAATTTTTGA

CGTGATTAACCAAATATTGTAGATGCTCAATTACATGTAGATTTGATGGAGTTGTCCACAA

TTTCTCATATATATATTAACTAATAACCATCTACCGAATATAATTAACCATATATTAGAAT

ATCATTAACCTCTAAAACAGTTGTCGCGAAAATTGGTTGTAAAAAAATCATTTCTATTTTT

TACGCCACTAGTTCTCTTGTTATTTCTCTTTGTTGGAAAGTTGTTGTTTTTAACTTTGAGC

TTAGTCTTATTAATTAACTTGTAATGGTAACAACATTTTTCTATCTTTGTTTGAAGGAAGT

AGTTACTGTGTCAACTTTAGTAATTAATTTGGATCATTTTGGAGGCAAAATATGGCTATGC

AACCTGTTTATTTTAAAGAACATGAAGGAAATGTCCACAATTCTGTTGGACAGTTTTCATC

TGTGACTTCAGCACCATGGTGGAGTAATGCCTATGGATCTCAACCTTTTTATGGAGGAGAC

TCTTGTGGCCAAATGAAACCTTTTTCACTAGAGCTTTCCAACTACATAGACCAACTTGCTC

CGAGTAAGAACTTAGTTCGAGGAGTTGAACAATTGTTTGATAAAGGGCATACAAACCAATT

CACCATCTTTCCAGGTACTTGTTATTCAATATAATTCCGGTTTTGAATGAATTGATTTTTG

TTAAGTTGGTTCTGGATAAACGTGCGTTAAATATAATATGATTTATGTTTGGATACGTTTA

TGTTAAAGTGAGTTGAACATAAATTGGAGACTAAATATCAATTGCAGAGGCAAAAGCTTCA

AATTCTAATTTCAAGTTAGAATCAAATCAATTCTACTCGTAAGCATCTAAATGTTTCAAAA

CCAATTCTACATCTCTAGAACTAAATTTGCATTCAAACATTTTCTGCTACAGATGATTGTA

AGATGTCAGTTGATACACCAAATCATCAAGCAACCTTATCCCTGCAATCACCATTTGCTGC

CGAGCCACTTAATCGATTTGAGCTAGGTTTTAACCAGTCTATGGTAAATCTTCTTATTAAA

GCAATCCCTTTGATCATGTTTTAGTATTTTACTTTTGATGTACATAAATTCATTATGTTTT

TATTATCCTTTCCATGCTAGATCTGTGCAAAATATCCGTATATAGATCAATTTTACGGGCT

CTTCTCGACTTATGGACCTCAAATCTCGGTTTGTCTTCACCTCGGCTATTTTATTAATCTT

TGTATATATCATGCAATGTCTATAAGTACTTGAATGTGAATTTGTTTTATTTACAGTGTGT

ATGCTTACATGAGAAAGCCAGAAATCTGTTTACAACTATCTTGTTAGTATTCTTCTAAATC

TTTCAAATGTAGAGTTGATTATGAAATTTGTTGTTAATGAGTTTTCGGCTTTATGAATGAA

TTGTAAGACAAGTAATGAAACAAAAACTAGAAGAAAATTGAATTATATGATGCAATCTGTG

TTGAAGTTTTTGCATGTATTCAAGTTCTGCTTTTAGTTACTATTTGTTTATCTGCTGCAAT

ATTTCATCAATTATTATTCATGTATGTACCAATATTTAATAAATTAGGGGCGAATTATGCT

TCCGCTTAGCATGACATCTGACGACGGACCAACATACGTGAATGCTAAGCAATACCATGGA

ATCATCAGACGCAGGCATTCTCGTGCCAAAGCTGTGCTTCAGAATAAATTGATAAAGCGTA

ACAAGGTATGAAACTGAGTATTTTTCCTCACAACAATGTTCGAAAACTTGTGAAATAGTAT

ATTTTTCCTTTTACCCTTTTTATACCTATATTAAGGTTTTGTATTTGTCTTGCAGCCATAT

ATGCACGAATCGCGTCATCTACATGCAATGCGTAGACCAAGAGGATGCGGTGGTCGTTTCT

TGAACACAAAAGTTTCTGCTAATGGAAACGGTAAAAGCGGGAGTGAAGAGAACGGAAACAT

TGGTGGCCTACAGCTGCAGTCCAGTGGTTCTCAGAGTTCCGAAGTCTTACAATCTGAGGTT

GGAACTTTAAATTCGTCGAAGGAGACAAACGGAGGCAGTCCAAATGTCTCGGGGTCAGAGG

TGACTAGCATGTATACACAGGGAGGTCTTGATAGCTTTACTGTCAATCATATCGGATCTAC

TGTCCACTCTTTGGGAGACATGATCGATACTGGACACGGTATCGTCATGCCAACAAAATGG

TTTGCAGCAGCTGGCAGACAGCTGCTGGAACCATAAGTTTCGATTCAGAAAGGAAACAAGT

GGGTTTGGTACAATGTGAAATATTTTGCACCAAACTCATCCTTTCCGAGACCAGATGAAGA

AGCTATGTTTCAGTTTGTTGTGTTTACTACGACAAATTTAGTTTCGGAAGACTACTTTTCC

ATCTGGTGCTCAGGCAACTCATTCTTGGCTTATTCTCAGGAAACTCATCCTTGGCTCGTAA

TATTTAGTAGTATTGTCATTGTCTTCCCGCGCAGGCTTTCCGTGGCATGGTAGGCATGCTA

ATGACTTTGGTATTTTCATGCAGTTATAACTATGATGTGTCTTTGTTTGTTGTTAAAATAA

AAAACATGAACTCTAGCTAGGTGCATGTGTGTGTTTTTAATCTTGTCTACTAAGTTTGGTG

TTTTGTAATGGATTTCTGACTTTATGGAGCAATGTATTGTAACTCTACTAAGAAGTGTAAC

ATTTTATTTCTCCCTCTCTAAGGATTGTATAAGAACCTCTTATTTTCAGACTCTACTTAAT

CCTATTTTCTATGTCTGTATGATTTTTATATTTCTAGGACAATCAAATTGGCTTGTAGAAG

CTCAAAAGCATGCTCACAAAGTAGGTACTTATGTAGGGAACTTCGTACTCTAATAATAACT

GGTTATTAACGTTATAATTAAATGCAAAATTTGATAAGTAGTAGGGTTTGGTAAGTAATAT

AACAACACCATGTTGGCTTGGGATTCTGAATTGTGTTATTAGAGTACAACTCTTAAATAAT

GATCGTGATGTAAATAATTTAAATTTGAAGTTACTAGATAAAAAGATAACATTATGTAATA

TATTATCAAATATATACTAAAAATATAATTTTCATCTATCTCAATTGAAAAAATATCTGGG

TTTTGAGTCTATTGTCATGGACGA

>HM025

AAAAAAATAAAGGTTCTATACTCATTAAACAAAGTGCCAAATTCAGAGATACTACTTCCT

TTGGATAATTTGTATCGTCACCAACGATCTTTGAGTTATTTCAAAAATCACATTCTCAATT

TTGTGGTCCAACATTCACAAAATGGTTTGTCGGGCTTTAAGTCTAGTAATTGTGAAACATG

CATATGCATGAGGTAATTAATTGATAATCTGAAAAGGTTAAATAGTTAATCAATTGTACTT

TGGTTATTTTTAATTTGTTCTCTATAATTTTTCAGTCAAAATTGAATTTGCTTTAACCATT

TAGTGTAGTAGTATGATATTACTTGCTCTACATTCTTGGATCTAAAGGTCTAATTAGTATC

GTATAAAAAATATTCTAGTTAATTAAGATACTTTTGTTCATTCTAATTATTCTTGGATTAC

TTGTGAAACTTTTAGGGATGAACAAGAAAGTCCACTAAAGAGGCTTCTTTTTGCTAGGAGT

CACTAGTTAGTTTTTTTTTTTTTTGACAAAAGTTAACCATTTTTTTTTATCGCATGGTAGG

AAAAAAAAAATAGAGATTTCCTTTGGTACTCATAAAAAAAAAAGTTGGATGGATAATGGAT

TAATCATAACTTATTTAACATAAAATGAACTAAGAGAACATTTCATATTTGGGTTTATTTT

GTAACATAAATATTAATATCAATGTTTGAAAGAGTTTTTAACAATAATTTGTAAAAGAAAG

CATAATATATCTATCAAAATAATTGTAAAAGAAAAAATTTCTTTTGGTGGTATGACATGGA

AAAATAATTAATTCTTATTAGATGAAAGTGTAAAATTAATTTGCATTAACAAAGCATATTT

CATTAAACTCCTTTTAGTTTTTGTTGGAAAAAATAAATTTATTGTTACTCAAAAAATAAAA

GTGTACAATTAATATATTTACTCAAAAGTCCCCTAAATGGGATGTAATTATCAAGGGATGT

GAGCGGTTTAACTTTAAGTAATGAATCAACTGTTGCATAATCACTTTGAAAGAAACTTGAG

ATTCTTCAATTAGCAAAATCTTGAATAGAATAATGAGATTATTCCTAATGCTAATGATACC

CACCTCTCCTATAAGTTGATGGGGCAGAACAAATTATGCACTCAGGACAACTTTTCTCAAA

TTTTGAAAACTTACCACACGAAGAACACACTTCCACTTGCACAAGTAAGAATATAGTATAA

CAATAAATTTACCACTCATAGCTCAATGGATATTAAAATATTGCTAATCTTTTATAGACAG

ATGTTGGCAAATTAGTATAAATTAGTTATTATATTAGTTTTTAAGGTTCAATATACTTTTT

AAAACTCTTATTAAGTTGTTTAGCTTGCCAACCAAAGACTAAATTATCTTTATTTGTTAGG

AAATAAAAGCACTTATTTTATTTTCTCCATATTTGTTTCCTCTTTGTTCCATTACTAAAAA

AATTATAATATATTAATAACAAAAAAATTTGGGGAGGGAATGTTATTTTTAAAAAAGATGT

TTTATTTATATTTAAGTGTTTTATTGAAATTCCTCGTGAGTTTAGCTCAATTGGTATGGAC

AATACATAATATATGCAAAATTCGAGGTTCAAACCTTGAGCACCACAAAAAAAAGTGTTTT

ATTGAAAAATATCAAGAACTTGAAAACAAATTTCCTTTTTATGAACTAGATATTGTCACAT

AGACAACTTAAGAATTGACTTTTTTTTTAACACTATTGTATTTTCTGAACTCGACTTTAAC

TCGAGATCAATGAGTAAACTAAAAAAACTCGTATTATTTTATCTAAGTGTTCTTAGTGTGA

GAAAACATTATAATTGTTTGCTTAGTCTATTTCTCCCTATATTCTCACCCTCATTCTTGGT

TTGCACATAGAAAGGAGAAAAAAAAAATTAGAGAAGAGTTGAATGGAACAGAAAGAAAGAG

CAAGCAAAATAATCACTAAAAAAAATTCCCCTAAAAGAAAATCACTGAAAAATATTATCCA

CCTCTATTTTAAATAATAATAATAATGAAAACAACAATATATTTTTTTATAGATTTTCTCT

ACTAGAGGTAATTTATTTTAAACACTAAATGTCCACACCTCTAACAAAAAAAATGCATAAT

ATTATAATAGATTATTGTAAGAAAGAGTGACAAATGTTGAATTAGTTATGGAATGAAGAGT

GAGAGAGATTATACAAATAAATAAATATAAATATAAATATAAATAAAGAATAGAGAAGAAC

ATAGTACTTGAATGAAGAGATTTGGCTTGTACTTCCATTACACTTGTAAGCTTTCTACTTC

CTTCACATTCCTTCTCTTTCTCTATCTTTCCTTTTATGATATGTGTACTTTGTTATTGCTT

CATTTACTACCGACATGACTCCAACCTATTAGGGCTTTATCTGAATAATGTTAGTTATTTT

CTCTCATTTTCTTTGCTTCTTCTTATTCTTGGAACTTATAAAGGGTCTTGTCAATTCTATC

ATTCTCTTTTGTATACTTACAAATATAAATATTCTCTTTAGCTTCATTTTCATGTCTTCTT

CTACTAATAAGCTATGGAAATTGAAGTTTTTGTTTTCATGTTTATTTTTCACTTCAGCTCC

TCTTTTTTGATGTTGATGCTGAAGATTGAATAAAAACTGAGGTGGTATGTATGAGAACAAG

AACATTTTTCATTCATCTCTGTTATTTGAACATCTAAATTTTAAACAACTATCGCGACAAT

TTTTTAAGTGGTTGAAGTATTTTAAATAGGTGGTTAATTACATTAAGTAGATAGTTAATTG

ATATATCAGGTGTCAAAACTCATTAACCATCAACATAACTTCATTAGCCATAAATTTTTGA

CGTGATTAACCAAATATTGTAGATGCTCAATTACATGTAGATTTGATGGAGTTGTCCACAA

TTTCTCATATATATATTAACTAATAACCATCTACCGAATATAATTAACCATATATTAGAAT

ATCATTAACCTCTAAAACAGTTGTCGCGAAAATTGGTTGTAAAAAAATCATTTCTATTTTT

TACGCCACTAGTTCTCTTGTTATTTCTCTTTGTTGGAAAGTTGTTGTTTTTAACTTTGAGC

TTAGTCTTATTAATTAACTTGTAATGGTAACAACATTTTTCTATCTTTGTTTGAAGGAAGT

AGTTACTGTGTCAACTTTAGTAATTAATTTGGATCATTTTGGAGGCAAAATATGGCTATGC

AACCTGTTTATTTTAAAGAACATGAAGGAAATGTCCACAATTCTGTTGGACAGTTTTCATC

TGTGACTTCAGCACCATGGTGGAGTAATGCCTATGGATCTCAACCTTTTTATGGAGGAGAC

TCTTGTGGCCAAATGAAACCTTTTTCACTAGAGCTTTCCAACTACATAGACCAACTTGCTC

CGAGTAAGAACTTAGTTCGAGGAGTTGAACAATTGTTTGATAAAGGGCATACAAACCAATT

CACCATCTTTCCAGGTACTTGTTATTCAATATAATTCCGGTTTTGAATGAATTGATTTTTG

TTAAGTTGGTTCTGGATAAACGTGCGTTAAATATAATATGATTTATGTTTGGATACGTTTA

TGTTAAAGTGAGTTGAACATAAATTGGAGACTAAATATCAATTGCAGAGGCAAAAGCTTCA

AATTCTAATTTCAAGTTAGAATCAAATCAATTCTACTCGTAAGCATCTAAATGTTTCAAAA

CCAATTCTACATCTCTAGAACTAAATTTGCATTCAAACATTTTCTGCTACAGATGATTGTA

AGATGTCAGTTGATACACCAAATCATCAAGCAACCTTATCCCTGCAATCACCATTTGCTGC

CGAGCCACTTAATCGATTTGAGCTAGGTTTTAACCAGTCTATGGTAAATCTTCTTATTAAA

GCAATCCCTTTGATCATGTTTTAGTATTTTACTTTTGATGTACATAAATTCATTATGTTTT

TATTATCCTTTCCATGCTAGATCTGTGCAAAATATCCGTATATAGATCAATTTTACGGGCT

CTTCTCGACTTATGGACCTCAAATCTCGGTTTGTCTTCACCTCGGCTATTTTATTAATCTT

TGTATATATCATGCAATGTCTATAAGTACTTGAATGTGAATTTGTTTTATTTACAGTGTGT

ATGCTTACATGAGAAAGCCAGAAATCTGTTTACAACTATCTTGTTAGTATTCTTCTAAATC

TTTCAAATGTAGAGTTGATTATGAAATTTGTTGTTAATGAGTTTTCGGCTTTATGAATGAA

TTGTAAGACAAGTAATGAAACAAAAACTAGAAGAAAATTGAATTATATGATGCAATCTGTG

TTGAAGTTTTTGCATGTATTCAAGTTCTGCTTTTAGTTACTATTTGTTTATCTGCTGCAAT

ATTTCATCAATTATTATTCATGTATGTACCAATATTTAATAAATTAGGGGCGAATTATGCT

TCCGCTTAGCATGACATCTGACGACGGACCAACATACGTGAATGCTAAGCAATACCATGGA

ATCATCAGACGCAGGCATTCTCGTGCCAAAGCTGTGCTTCAGAATAAATTGATAAAGCGTA

ACAAGGTATGAAACTGAGTATTTTTCCTCACAACAATGTTCGAAAACTTGTGAAATAGTAT

ATTTTTCCTTTTACCCTTTTTATACCTATATTAAGGTTTTGTATTTGTCTTGCAGCCATAT

ATGCACGAATCGCGTCATCTACATGCAATGCGTAGACCAAGAGGATGCGGTGGTCGTTTCT

TGAACACAAAAGTTTCTGCTAATGGAAACGGTAAAAGCGGGAGTGAAGAGAACGGAAACAT

TGGTGGCCTACAGCTGCAGTCCAGTGGTTCTCAGAGTTCCGAAGTCTTACAATCTGAGGTT

GGAACTTTAAATTCGTCGAAGGAGACAAACGGAGGCAGTCCAAATGTCTCGGGGTCAGAGG

TGACTAGCATGTATACACAGGGAGGTCTTGATAGCTTTACTGTCAATCATATCGGATCTAC

TGTCCACTCTTTGGGAGACATGATCGATACTGGACACGGTATCGTCATGCCAACAAAATGG

TTTGCAGCAGCTGGCAGACAGCTGCTGGAACCATAAGTTTCGATTCAGAAAGGAAACAAGT

GGGTTTGGTACAATGTGAAATATTTTGCACCAAACTCATCCTTTCCGAGACCAGATGAAGA

AGCTATGTTTCAGTTTGTTGTGTTTACTACGACAAATTTAGTTTCGGAAGACTACTTTTCC

ATCTGGTGCTCAGGCAACTCATTCTTGGCTTATTCTCAGGAAACTCATCCTTGGCTCGTAA

TATTTAGTAGTATTGTCATTGTCTTCCCGCGCAGGCTTTCCGTGGCATGGTAGGCATGCTA

ATGACTTTGGTATTTTCATGCAGTTATAACTATGATGTGTCTTTGTTTGTTGTTAAAATAA

AAAACATGAACTCTAGCTAGGTGCATGTGTGTGTTTTTAATCTTGTCTACTAAGTTTGGTG

TTTTGTAATGGATTTCTGACTTTATGGAGCAATGTATTGTAACTCTACTAAGAAGTGTAAC

ATTTTATTTCTCCCTCTCTAAGGATTGTATAAGAACCTCTTATTTTCAGACTCTACTTAAT

CCTATTTTCTATGTCTGTATGATTTTTATATTTCTAGGACAATCAAATTGGCTTGTAGAAG

CTCAAAAGCATGCTCACAAAGTAGGTACTTATGTAGGGAACTTCGTACTCTAATAATAACT

GGTTATTAACGTTATAATTAAATGCAAAATTTGATAAGTAGTAGGGTTTGGTAAGTAATAT

AACAACACCATGTTGGCTTGGGATTCTGAATTGTGTTATTAGAGTACAACTCTTAAATAAT

GATCGTGATGTAAATAATTTAAATTTGAAGTTACTAGATAAAAAGATAACATTATGTAATA

TATTATCAAATATATACTAAAAATATAATTTTCATCTATCTCAATTGAAAAAATATCTGGG

TTTTGAGTCTATTGTCATGGACGA

>HM026

AAAAAAATAAAGGTTCTATACTCATTAAACAAAGTGCCAAATTCAGAGATACTACTTCCT

TTGGATAATTTGTATCGTCACCAACGATCTTTGAGTTATTTCAAAAATCACATTCTCAATT

TTGTGGTCCAACATTCACAAAATGGTTTGTCGGGCTTTAAGTCTAGTAATTGTGAAACATG

CATATGCATGAGGTAATTAATTGATAATCTGAAAAGGTTAAATAGTTAATCAATTGTACTT

TGGTTATTTTTAATTTGTTCTCTATAATTTTTCAGTCAAAATTGAATTTGCTTTAACCATT

TAGTGTAGTAGTATGATATTACTTGCTCTACATTCTTGGATCTAAAGGTCTAATTAGTATC

GTATAAAAAATATTCTAGTTAATTAAGATACTTTTGTTCATTCTAATTATTCTTGGATTAC

TTGTGAAACTTTTAGGGATGAACAAGAAAGTCCACTAAAGAGGCTTCTTTTTGCTAGGAGT

CACTAGTTAGTTTTTTTTTTTTTTGACAAAAGTTAACCATTTTTTTTTATCGCATGGTAGG

AAAAAAAAAATAGAGATTTCCTTTGGTACTCATAAAAAAAAAAGTTGGATGGATAATGGAT

TAATCATAACTTATTTAACATAAAATGAACTAAGAGAACATTTCATATTTGGGTTTATTTT

GTAACATAAATATTAATATCAATGTTTGAAAGAGTTTTTAACAATAATTTGTAAAAGAAAG

CATAATATATCTATCAAAATAATTGTAAAAGAAAAAATTTCTTTTGGTGGTATGACATGGA

AAAATAATTAATTCTTATTAGATGAAAGTGTAAAATTAATTTGCATTAACAAAGCATATTT

CATTAAACTCCTTTTAGTTTTTGTTGGAAAAAATAAATTTATTGTTACTCAAAAAATAAAA

GTGTACAATTAATATATTTACTCAAAAGTCCCCTAAATGGGATGTAATTATCAAGGGATGT

GAGCGGTTTAACTTTAAGTAATGAATCAACTGTTGCATAATCACTTTGAAAGAAACTTGAG

ATTCTTCAATTAGCAAAATCTTGAATAGAATAATGAGATTATTCCTAATGCTAATGATACC

CACCTCTCCTATAAGTTGATGGGGCAGAACAAATTATGCACTCAGGACAACTTTTCTCAAA

TTTTGAAAACTTACCACACGAAGAACACACTTCCACTTGCACAAGTAAGAATATAGTATAA

CAATAAATTTACCACTCATAGCTCAATGGATATTAAAATATTGCTAATCTTTTATAGACAG

ATGTTGGCAAATTAGTATAAATTAGTTATTATATTAGTTTTTAAGGTTCAATCTACTTTTT

AAAACTCTTATTAAGTTGTTTAGCTTGCCAACCAAAGACTAAATTATCTTTATTTGTTAGG

AAATAAAAGCACTTATTTTATTTTCTCCATATTTGTTTCCTCTTTGTTCCATTACTAAAAA

AATTATAATATATTAATAACAAAAAAATTTGGGGAGGGAATGTTATTTTTAAAAAAGATGT

TTTATTTATATTTAAGTGTTTTATTGAAATTCCTCGTGAGTTTAGCTCAATTGGTATGGAC

AATACATAATATATGCAAAATTCGAGGTTCAAACCTTGAGCACCACAAAAAAAAGTGTTTT

ATTGAAAAATATCAAGAACTTGAAAACAAATTTCCTTTTTATGAACTAGATATTGTCACAT

AGACAACTTAAGAATTGACATTTTTTTTAACACTATTGTATTTTCTGAACTCGACTTTAAC

TCGAGATCAATGAGTAAACTAAAAAAACTCGTATTATTTTATCTAAGTGTTCTTAGTGTGA

GAAAACATTATAATTGTTTGCTTAGTCTATTTCTCCCTATATTCTCACCCTCATTCTTGGT

TTGCACATAGAAAGGAGAAAAAAAAAATTAGAGAAGAGTTGAATGGAACAGAAAGAAAGAG

CAAGCAAAATAATCACTAAAAAAAATTCCCCTAAAAGAAAATCACTGAAAAATATTATCCA

CCTCTATTTTAAATAATAATAATAATGAAAACAACAATATATTTTTTTATAGATTTTCTCT

ACTAGAGGTAATTTATTTTAAACACTAAATGTCCACACCTCTAACAAAAAAAATGCATAAT

ATTATAATAGATTATTGTAAGAAAGAGTGACAAATGTTGAATTAGTTATGGAATGAAGAGT

GAGAGAGATTATACAAATAAATAAATATAAATATAAATATAAATAAAGAATAGAGAAGAAC

ATAGTACTTGAATGAAGAGATTTGGCTTGTACTTCCATTACACTTGTAAGCTTTCTACTTC

CTTCACATTCCTTCTCTTTCTCTATCTTTCCTTTTATGATATGTGTACTTTGTTATTGCTT

CATTTACTACCGACATGACTCCAACCTATTAGGGCTTTATCTGAATAATGTTAGTTATTTT

CTCTCATTTTCTTTGCTTCTTCTTATTCTTGGAACTTATAAAGGGTCTTGTCAATTCTATC

ATTCTCTTTTGTATACTTACAAATATAAATATTCTCTTTAGCTTCATTTTCATGTCTTCTT

CTACTAATAAGCTATGGAAATTGAAGTTTTTGTTTTCATGTTTATTTTTCACTTCAGCTCC

TCTTTTTTGATGTTGATGCTGAAGATTGAATAAAAACTGAGGTGGTATGTATGAGAACAAG

AACATTTTTCATTCATCTCTGTTATTTGAACATCTAAATTTTAAACAACTATCGCGACAAT

TTTTTAAGTGGTTGAAGTATTTTAAATAGGTGGTTAATTACATTAAGTAGATAGTTAATTG

ATATATCAGGTGTCAAAACTCATTAACCATCAACATAACTTCATTAGCCATAAATTTTTGA

CGTGATTAACCAAATATTGTAGATGCTCAATTACATGTAGATTTGATGGAGTTGTCCACAA

TTTCTCATATATATATTAACTAATAACCATCTACCGAATATAATTAACCATATATTAGAAT

ATCATTAACCTCTAAAACAGTTGTCGCGAAAATTGGTTGTAAAAAAATCATTTCTATTTTT

TACGCCACTAGTTCTCTTGTTATTTCTCTTTGTTGGAAAGTTGTTGTTTTTAACTTTGAGC

TTAGTCTTATTAATTAACTTGTAATGGTAACAACATTTTTCTATCTTTGTTTGAAGGAAGT

AGTTACTGTGTCAACTTTAGTAATTAATTTGGATCATTTTGGAGGCAAAATATGGCTATGC

AACCTGTTTATTTTAAAGAACATGAAGGAAATGTCCACAATTCTGTTGGACAGTTTTCATC

TGTGACTTCAGCACCATGGTGGAGTAATGCCTATGGATCTCAACCTTTTTATGGAGGAGAC

TCTTGTGGCCAAATGAAACCTTTTTCACTAGAGCTTTCCAACTACATAGACCAACTTGCTC

CGAGTAAGAACTTAGTTCGAGGAGTTGAACAATTGTTTGATAAAGGGCATACAAACCAATT

CACCATCTTTCCAGGTACTTGTTATTCAATATAATTCCGGTTTTGAATGAATTGATTTTTG

TTAAGTTGGTTCTGGATAAACGTGCGTTAAATATAATATGATTTATGTTTGGATACGTTTA

TGTTAAAGTGAGTTGAACATAAATTGGAGACTAAATATCAATTGCAGAGGCAAAAGCTTCA

AATTCTAATTTCAAGTTAGAATCAAATCAATTCTACTCGTAAGCATCTAAATGTTTCAAAA

CCAATTCTACATCTCTAGAACTAAATTTGCATTCAAACATTTTCTGCTACAGATGATTGTA

AGATGTCAGTTGATACACCAAATCATCAAGCAACCTTATCCCTGCAATCACCATTTGCTGC

CGAGCCACTTAATCGATTTGAGCTAGGTTTTAACCAGTCTATGGTAAATCTTCTTATTAAA

GCAATCCCTTTGATCATGTTTTAGTATTTTACTTTTGATGTACATAAATTCATTATGTTTT

TATTATCCTTTCCATGCTAGATCTGTGCAAAATATCCGTATATAGATCAATTTTACGGGCT

CTTCTCGACTTATGGACCTCAAATCTCGGTTTGTCTTCACCTCGGCTATTTTATTAATCTT

TGTATATATCATGCAATGTCTATAAGTACTTGAATGTGAATTTGTTTTATTTACAGTGTGT

ATGCTTACATGAGAAAGCCAGAAATCTGTTTACAACTATCTTGTTAGTATTCTTCTAAATC

TTTCAAATGTAGAGTTGATTATGAAATTTGTTGTTAATGAGTTTTCGGCTTTATGAATGAA

TTGTAAGACAAGTAATGAAACAAAAACTAGAAGAAAATTGAATTATATGATGCAATCTGTG

TTGAAGTTTTTGCATGTATTCAAGTTCTGCTTTTAGTTACTATTTGTTTATCTGCTGCAAT

ATTTCATCAATTATTATTCATGTATGTACCAATATTTAATAAATTAGGGGCGAATTATGCT

TCCGCTTAGCATGACATCTGACGACGGACCAACATACGTGAATGCTAAGCAATACCATGGA

ATCATCAGACGCAGGCATTCTCGTGCCAAAGCTGTGCTTCAGAATAAATTGATAAAGCGTA

ACAAGGTATGAAACTGAGTATTTTTCCTCACAACAATGTTCGAAAACTTGTGAAATAGTAT

ATTTTTCCTTTTACCCTTTTTATACCTATATTAAGGTTTTGTATTTGTCTTGCAGCCATAT

ATGCACGAATCGCGTCATCTACATGCAATGCGTAGACCAAGAGGATGCGGTGGTCGTTTCT

TGAACACAAAAGTTTCTGCTAATGGAAACGGTAAAAGCGGGAGTGAAGAGAACGGAAACAT

TGGTGGCCTACAGCTGCAGTCCAGTGGTTCTCAGAGTTCCGAAGTCTTACAATCTGAGGTT

GGAACTTTAAATTCGTCGAAGGAGACAAACGGAGGCAGTCCAAATGTCTCGGGGTCAGAGG

TGACTAGCATGTATACACAGGGAGGTCTTGATAGCTTTACTGTCAATCATATCGGATCTAC

TGTCCACTCTTTGGGAGACATGATCGATACTGGACACGGTATCGTCATGCCAACAAAATGG

TTTGCAGCAGCTGGCAGACAGCTGCTGGAACCATAAGTTTCGATTCAGAAAGGAAACAAGT

GGGTTTGGTACAATGTGAAATATTTTGCACCAAACTCATCCTTTCCGAGACCAGATGAAGA

AGCTATGTTTCAGTTTGTTGTGTTTACTACGACAAATTTAGTTTCGGAAGACTACTTTTCC

ATCTGGTGCTCAGGCAACTCATTCTTGGCTTATTCTCAGGAAACTCATCCTTGGCTCGTAA

TATTTAGTAGTATTGTCATTGTCTTCCCGCGCAGGCTTTCCGTGGCATGGTAGGCATGCTA

ATGACTTTGGTATTTTCATGCAGTTATAACTATGATGTGTCTTTGTTTGTTGTTAAAATAA

AAAACATGAACTCTAGCTAGGTGCATGTGTGTGTTTTTAATCTTGTCTACTAAGTTTGGTG

TTTTGTAATGGATTTCTGACTTTATGGAGCAATGTATTGTAACTCTACTAAGAAGTGTAAC

ATTTTATTTCTCCCTCTCTAAGGATTGTATAAGAACCTCTTATTTTCAGACTCTACTTAAT

CCTATTTTCTATGTCTGTATGATTTTTATATTTCTAGGACAATCAAATTGGCTTGTAGAAG

CTCAAAAGCATGCTCACAAAGTAGGTACTTATGTAGGGAACTTCGTACTCTAATAATAACT

GGTTATTAACGTTATAATTAAATGCAAAATTTGATAAGTAGTAGGGTTTGGTAAGTAATAT

AACAACACCATGTTGGCTTGGGATTCTGAATTGTGTTATTAGAGTACAACTCTTAAATAAT

GATCGTGATGTAAATAATTTAAATTTGAAGTTACTAGATAAAAAGATAACATTATGTAATA

TATTATCAAATATATACTAAAAATATAATTTTCATCTATCTCAATTGAAAAAATATCTGGG

TTTTGAGTCTATTGTCATGGACGA

>HM027

AAAAAAATAAAGGTTCTATACTCATTAAACAAAGTGCCAAATTCAGAGATACTACTTCCT

TTGGATAATTTGTATCGTCACCAACGATCTTTGAGTTATTTCAAAAATCACATTCTCAATT

TTGTGGTCCAACATTCACAAAATGGTTTGTCGGGCTTTAAGTCTAGTAATTGTGAAACATG

CATATGCATGAGGTAATTAATTGATAATCTGAAAAGGTTAAATAGTTAATCAATTGTACTT

TGGTTATTTTTAATTTGTTCTCTATAATTTTTCAGTCAAAATTGAATTTGCTTTAACCATT

TAGTGTAGTAGTATGATATTACTTGCTCTACATTCTTGGATCTAAAAGTCTAATTAGTATC

GTATAAAAAATATTCTAGTTAATTAAGATACTTTTGTTCATTCTAATTATTCTTGGATTAC

TTGTGAAACTTTTAGGGATGAACAAGAAAGTCCACTAAAGAGGCTTCTTTTTGCTAGGAGT

CACTAGTTAGTTTTTTTTTTTTTTGACAAAAGTTAACCATTTTTTTTTATCGCATGGTAGG

AAAAAAAAAATAGAGATTTCCTTTGGTACTCATAAAAAAAAAAGTTGGATGGATAATGGAT

TAATCATAACTTATTTAACATAAAATGAACTAAGAGAACATTTCATATTTGGGTTTATTTT

GTAACATAAATATTAATATCAATGTTTGAAAGAGTTTTTAACAATAATTTGTAAAAGAAAG

CATAATATATCTATCAAAATAATTGTAAAAGAAAAAATTTCTTTTGGTGGTATGACATGGA

AAAATAATTAATTCTTATTAGATGAAAGTGTAAAATTAATTTGCATTAACAAAGCATATTT

CATTAAACTCCTTTTAGTTTTTGTTGGAAAAAATAAATTTATTGTTACTCAAAAAATAAAA

GTGTACAATTAATATATTTACTCAAAAGTCCCCTAAATGGGATGTAATTATCAAGGGATGT

GAGCGGTTTAACTTTAAGTAATGAATCAACTGTTGCATAATCACTTTGAAAGAAACTTGAG

ATTCTTCAATTAGCAAAATCTTGAATAGAATAATGAGATTATTCCTAATGCTAATGATACC

CACCTCTCCTATAAGTTGATGGGGCAGAACAAATTATGCACTCAGGACAACTTTTCTCAAA

TTTTGAAAACTTACCACACGAAGAACACACTTCCACTTGCACAAGTAAGAATATAGTATAA

CAATAAATTTACCACTCATAGCTCAATGGATATTAAAATATTGCTAATCTTTTATAGACAG

ATGTTGGCAAATTAGTATAAATTAGTTATTATATTAGTTTTTAAGGTTCAATCTACTTTTT

AAAACTCTTATTAAGTTGTTTAGCTTGCCAACCAAAGACTAAATTATCTTTATTTGTTAGG

AAATAAAAGCACTTATTTTATTTTCTCCATATTTGTTTCCTCTTTGTTCCATTACTAAAAA

AATTATAATATATTAATAACAAAAAAATTTGGGGAGGGAATGTTATTTTTAAAAAAGATGT

TTTATTTATATTTAAGTGTTTTATTGAAATTCCTCGTGAGTTTAGCTCAATTGGTATGGAC

AATACATAATATATGCAAAATTCGAGGTTCAAACCTTGAGCACCAAAAAAAAAAGTGTTTT

ATTGAAAAATATCAAGAACTTGAAAACAAATTTCCTTTTTATGAACTAGATATTGTCACAT

AGACAACTTAAGAATTGACATTTTTTTTAACACTATTGTATTTTCTGAACTCGACTTTAAC

TCGAGATCAATGAGTAAACTAAAAAAACTCGTATTATTTTATCTAAGTGTTCTTAGTGTGA

GAAAACATTATAATTGTTTGCTTAGTCTATTTCTCCCTATATTCTCACCCTCATTCTTGGT

TTGCACATAGAAAGGAGAAAAAAAAAATTAGAGAAGAGTTGAATGGAACAGAAAGAAAGAG

CAAGCAAAATAATCACTAAAAAAAATTCCCCTAAAAGAAAATCACTGAAAAATATTATCCA

CCTCTATTTTAAATAATAATAATAATGAAAACAACAATATATTTTTTTATAGATTTTCTCT

ACTAGAGGTAATTTATTTTAAACACTAAATGTCCACACCTCTAACAAAAAAAATGCATAAT

ATTATAATAGATTATTGTAAGAAAGAGTGACAAATGTTGAATTAGTTATGGAATGAAGAGT

GAGAGAGATTATACAAATAAATAAATATAAATATAAATATAAATAAAGAATAGAGAAGAAC

ATAGTACTTGAATGAAGAGATTTGGCTTGTACTTCCATTACACTTGTAAGCTTTCTACTTC

CTTCACATTCCTTCTCTTTCTCTATCTTTCCTTTTATGATATGTGTACTTTGTTATTGCTT

CATTTACTACCGACATGACTCCAACCTATTAGGGCTTTATCTGAATAATGTTAGTTATTTT

CTCTCATTTTCTTTGCTTCTTCTTATTCTTGGAACTTATAAAGGGTCTTGTCAATTCTATC

ATTCTCTTTTGTATACTTACAAATATAAATATTCTCTTTAGCTTCATTTTCATGTCTTCTT

CTACTAATAAGCTATGGAAATTGAAGTTTTTGTTTTCATGTTTATTTTTCACTTCAGCTCC

TCTTTTTTGATGTTGATGCTGAAGATTGAATAAAAACTGAGGTGGTATGTATGAGAACAAG

AACATTTTTCATTCATCTCTGTTATTTGAACATCTAAATTTTAAACAACTATCGCGACAAT

TTTTTAAGTGGTTGAAGTATTTTAAATAGGTGGTTAATTACATTAAGTAGATAGTTAATTG

ATATATCAGGTGTCAAAACTCATTAACCATCAACATAACTTCATTAGCCATAAATTTTTGA

CGTGATTAACCAAATATTGTAGATGCTCAATTACATGTAGATTTGATGGAGTTGTCCACAA

TTTCTCATATATATATTAACTAATAACCATCTACCGAATATAATTAACCATATATTAGAAT

ATCATTAACCTCTAAAACAGTTGTCGCGAAAATTGGTTGTAAAAAAATCATTTCTATTTTT

TACGCCACTAGTTCTCTTGTTATTTCTCTTTGTTGGAAAGTTGTTGTTTTTAACTTTGAGC

TTAGTCTTATTAATTAACTTGTAATGGTAACAACATTTTTCTATCTTTGTTTGAAGGAAGT

AGTTACTGTGTCAACTTTAGTAATTAATTTGGATCATTTTGGAGGCAAAATATGGCTATGC

AACCTGTTTATTTTAAAGAACATGAAGGAAATGTCCACAATTCTGTTGGACAGTTTTCATC

TGTGACTTCAGCACCATGGTGGAGTAATGCCTATGGATCTCAACCTTTTTATGGAGGAGAC

TCTTGTGGCCAAATGAAACCTTTTTCACTAGAGCTTTCCAACTACATAGACCAACTTGCTC

CGAGTAAGAACTTAGTTCGAGGAGTTGAACAATTGTTTGATAAAGGGCATACAAACCAATT

CACCATCTTTCCAGGTACTTGTTATTCAATATAATTCCGGTTTTGAATGAATTGATTTTTG

TTAAGTTGGTTCTGGATAAACGTGCGTTAAATATAATATGATTTATGTTTGGATACGTTTA

TGTTAAAGTGAGTTGAACATAAATTGGAGACTAAATATCAATTGCAGAGGCAAAAGCTTCA

AATTCTAATTTCAAGTTAGAATCAAATCAATTCTACTCGTAAGCATCTAAATGTTTCAAAA

CCAATTCTACATCTCTAGAACTAAATTTGCATTCAAACATTTTCTGCTACAGATGATTGTA

AGATGTCAGTTGATACACCAAATCATCAAGCAACCTTATCCCTGCAATCACCATTTGCTGC

CGAGCCACTTAATCGATTTGAGCTAGGTTTTAACCAGTCTATGGTAAATCTTCTTATTAAA

GCAATCCCTTTGATCATGTTTTAGTATTTTACTTTTGATGTACATAAATTCATTATGTTTT

TATTATCCTTTCCATGCTAGATCTGTGCAAAATATCCGTATATAGATCAATTTTACGGGCT

CTTCTCGACTTATGGACCTCAAATCTCGGTTTGTCTTCACCTCGGCTATTTTATTAATCTT

TGTATATATCATGCAATGTCTATAAGTACTTGAATGTGAATTTGTTTTATTTACAGTGTGT

ATGCTTACATGAGAAAGCCAGAAATCTGTTTACAACTATCTTGTTAGTATTCTTCTAAATC

TTTCAAATGTAGAGTTGATTATGAAATTTGTTGTTAATGAGTTTTCGGCTTTATGAATGAA

TTGTAAGACAAGTAATGAAACAAAAACTAGAAGAAAATTGAATTATATGATGCAATCTGTG

TTGAAGTTTTTGCATGTATTCAAGTTCTGCTTTTAGTTACTATTTGTTTATCTGCTGCAAT

ATTTCATCAATTATTATTCATGTATGTACCAATATTTAATAAATTAGGGGCGAATTATGCT

TCCGCTTAGCATGACATCTGACGACGGACCAACATACGTGAATGCTAAGCAATACCATGGA

ATCATCAGACGCAGGCATTCTCGTGCCAAAGCTGTGCTTCAGAATAAATTGATAAAGCGTA

ACAAGGTATGAAACTGAGTATTTTTCCTCACAACAATGTTCGAAAACTTGTGAAATAGTAT

ATTTTTCCTTTTACCCTTTTTATACCTATATTAAGGTTTTGTATTTGTCTTGCAGCCATAT

ATGCACGAATCGCGTCATCTACATGCAATGCGTAGACCAAGAGGATGCGGTGGTCGTTTCT

TGAACACAAAAGTTTCTGCTAATGGAAACGGTAAAAGCGGGAGTGAAGAGAACGGAAACAT

TGGTGGCCTACAGCTGCAGTCCAGTGGTTCTCAGAGTTCCGAAGTCTTACAATCTGAGGTT

GGAACTTTAAATTCGTCGAAGGAGACAAACGGAGGCAGTCCAAATGTCTCGGGGTCAGAGG

TGACTAGCATGTATACACAGGGAGGTCTTGATAGCTTTACTGTCAATCATATCGGATCTAC

TGTCCACTCTTTGGGAGACATGATCGATACTGGACACGGTATCGTCATGCCAACAAAATGG

TTTGCAGCAGCTGGCAGACAGCTGCTGGAACCATAAGTTTCGATTCAGAAAGGAAACAAGT

GGGTTTGGTACAATGTGAAATATTTTGCACCAAACTCATCCTTTCCGAGACCAGATGAAGA

AGCTATGTTTCAGTTTGTTGTGTTTACTACGACAAATTTAGTTTCGGAAGACTACTTTTCC

ATCTGGTGCTCAGGCAACTCATTCTTGGCTTATTCTCAGGAAACTCATCCTTGGCTCGTAA

TATTTAGTAGTATTGTCATTGTCTTCCCGCGCAGGCTTTCCGTGGCATGGTAGGCATGCTA

ATGACTTTGGTATTTTCATGCAGTTATAACTATGATGTGTCTTTGTTTGTTGTTAAAATAA

AAAACATGAACTCTAGCTAGGTGCATGTGTGTGTTTTTAATCTTGTCTACTAAGTTTGGTG

TTTTGTAATGGATTTCTGACTTTATGGAGCAATGTATTGTAACTCTACTAAGAAGTGTAAC

ATTTTATTTCTCCCTCTCTAAGGATTGTATAAGAACCTCTTATTTTCAGACTCTACTTAAT

CCTATTTTCTATGTCTGTATGATTTTTATATTTCTAGGACAATCAAATTGGCTTGTAGAAG

CTCAAAAGCATGCTCACAAAGTAGGTACTTATGTAGGGAACTTCGTACTCTAATAATAACT

GGTTATTAACGTTATAATTAAATGCAAAATTTGATAAGTAGTAGGGTTTGGTAAGTAATAT

AACAACACCATGTTGGCTTGGGATTCTGAATTGTGTTATTAGAGTACAACTCTTAAATAAT

GATCGTGATGTAAATAATTTAAATTTGAAGTTACTAGATAAAAAGATAACATTATGTAATA

TATTATCAAATATATACTAAAAATATAATTTTCATCTATCTCAATTGAAAAAATATCTGGG

TTTTGAGTCTATTGTCATGGACGA

>HM028

AAAAAAATAAAGGTTCTATACTCATTAAACAAAGTGCCAAATTCAGAGATACTACTTCCT

TTGGATAATTTGTATCGTCACCAACGATCTTTGAGTTATTTCAAAAATCACATTCTCAATT

TTGTGGTCCAACATTCACAAAATGGTTTGTCGGGCTTTAAGTCTAGTAATTGTGAAACATG

CATATGCATGAGGTAATTAATTGATAATCTGAAAAGGTTAAATAGTTAATCAATTGTACTT

TGGTTATTTTTAATTTGTTCTCTATAATTTTTCAGTCAAAATTGAATTTGCTTTAACCATT

TAGTGTAGTAGTATGATATTACTTGCTCTACATTCTTGGATCTAAAAGTCTAATTAGTATC

GTATAAAAAATATTCTAGTTAATTAAGATACTTTTGTTCATTCTAATTATTCTTGGATTAC

TTGTGAAACTTTTAGGGATGAACAAGAAAGTCCACTAAAGAGGCTTCTTTTTGCTAGGAGT

CACTAGTTAGTTTTTTTTTTTTTTGACAAAAGTTAACCATTTTTTTTTATCGCATGGTAGG

AAAAAAAAAATAGAGATTTCCTTTGGTACTCATAAAAAAAAAAGTTGGATGGATAATGGAT

TAATCATAACTTATTTAACATAAAATGAACTAAGAGAACATTTCATATTTGGGTTTATTTT

GTAACATAAATATTAATATCAATGTTTGAAAGAGTTTTTAACAATAATTTGTAAAAGAAAG

CATAATATATCTATCAAAATAATTGTAAAAGAAAAAATTTCTTTTGGTGGTATGACATGGA

AAAATAATTAATTCTTATTAGATGAAAGTGTAAAATTAATTTGCATTAACAAAGCATATTT

CATTAAACTCCTTTTAGTTTTTGTTGGAAAAAATAAATTTATTGTTACTCAAAAAATAAAA

GTGTACAATTAATATATTTACTCAAAAGTCCCCTAAATGGGATGTAATTATCAAGGGATGT

GAGCGGTTTAACTTTAAGTAATGAATCAACTGTTGCATAATCACTTTGAAAGAAACTTGAG

ATTCTTCAATTAGCAAAATCTTGAATAGAATAATGAGATTATTCCTAATGCTAATGATACC

CACCTCTCCTATAAGTTGATGGGGCAGAACAAATTATGCACTCAGGACAACTTTTCTCAAA

TTTTGAAAACTTACCACACGAAGAACACACTTCCACTTGCACAAGTAAGAATATAGTATAA

CAATAAATTTACCACTCATAGCTCAATGGATATTAAAATATTGCTAATCTTTTATAGACAG

ATGTTGGCAAATTAGTATAAATTAGTTATTATATTAGTTTTTAAGGTTCAATCTACTTTTT

AAAACTCTTATTAAGTTGTTTAGCTTGCCAACCAAAGACTAAATTATCTTTATTTGTTAGG

AAATAAAAGCACTTATTTTATTTTCTCCATATTTGTTTCCTCTTTGTTCCATTACTAAAAA

AATTATAATATATTAATAACAAAAAAATTTGGGGAGGGAATGTTATTTTTAAAAAAGATGT

TTTATTTATATTTAAGTGTTTTATTGAAATTCCTCGTGAGTTTAGCTCAATTGGTATGGAC

AATACATAATATATGCAAAATTCGAGGTTCAAACCTTGAGCACCAAAAAAAAAAGTGTTTT

ATTGAAAAATATCAAGAACTTGAAAACAAATTTCCTTTTTATGAACTAGATATTGTCACAT

AGACAACTTAAGAATTGACATTTTTTTTAACACTATTGTATTTTCTGAACTCGACTTTAAC

TCGAGATCAATGAGTAAACTAAAAAAACTCGTATTATTTTATCTAAGTGTTCTTAGTGTGA

GAAAACATTATAATTGTTTGCTTAGTCTATTTCTCCCTATATTCTCACCCTCATTCTTGGT

TTGCACATAGAAAGGAGAAAAAAAAAATTAGAGAAGAGTTGAATGGAACAGAAAGAAAGAG

CAAGCAAAATAATCACTAAAAAAAATTCCCCTAAAAGAAAATCACTGAAAAATATTATCCA

CCTCTATTTTAAATAATAATAATAATGAAAACAACAATATATTTTTTTATAGATTTTCTCT

ACTAGAGGTAATTTATTTTAAACACTAAATGTCCACACCTCTAACAAAAAAAATGCATAAT

ATTATAATAGATTATTGTAAGAAAGAGTGACAAATGTTGAATTAGTTATGGAATGAAGAGT

GAGAGAGATTATACAAATAAATAAATATAAATATAAATATAAATAAAGAATAGAGAAGAAC

ATAGTACTTGAATGAAGAGATTTGGCTTGTACTTCCATTACACTTGTAAGCTTTCTACTTC

CTTCACATTCCTTCTCTTTCTCTATCTTTCCTTTTATGATATGTGTACTTTGTTATTGCTT

CATTTACTACCGACATGACTCCAACCTATTAGGGCTTTATCTGAATAATGTTAGTTATTTT

CTCTCATTTTCTTTGCTTCTTCTTATTCTTGGAACTTATAAAGGGTCTTGTCAATTCTATC

ATTCTCTTTTGTATACTTACAAATATAAATATTCTCTTTAGCTTCATTTTCATGTCTTCTT

CTACTAATAAGCTATGGAAATTGAAGTTTTTGTTTTCATGTTTATTTTTCACTTCAGCTCC

TCTTTTTTGATGTTGATGCTGAAGATTGAATAAAAACTGAGGTGGTATGTATGAGAACAAG

AACATTTTTCATTCATCTCTGTTATTTGAACATCTAAATTTTAAACAACTATCGCGACAAT

TTTTTAAGTGGTTGAAGTATTTTAAATAGGTGGTTAATTACATTAAGTAGATAGTTAATTG

ATATATCAGGTGTCAAAACTCATTAACCATCAACATAACTTCATTAGCCATAAATTTTTGA

CGTGATTAACCAAATATTGTAGATGCTCAATTACATGTAGATTTGATGGAGTTGTCCACAA

TTTCTCATATATATATTAACTAATAACCATCTACCGAATATAATTAACCATATATTAGAAT

ATCATTAACCTCTAAAACAGTTGTCGCGAAAATTGGTTGTAAAAAAATCATTTCTATTTTT

TACGCCACTAGTTCTCTTGTTATTTCTCTTTGTTGGAAAGTTGTTGTTTTTAACTTTGAGC

TTAGTCTTATTAATTAACTTGTAATGGTAACAACATTTTTCTATCTTTGTTTGAAGGAAGT

AGTTACTGTGTCAACTTTAGTAATTAATTTGGATCATTTTGGAGGCAAAATATGGCTATGC

AACCTGTTTATTTTAAAGAACATGAAGGAAATGTCCACAATTCTGTTGGACAGTTTTCATC

TGTGACTTCAGCACCATGGTGGAGTAATGCCTATGGATCTCAACCTTTTTATGGAGGAGAC

TCTTGTGGCCAAATGAAACCTTTTTCACTAGAGCTTTCCAACTACATAGACCAACTTGCTC

CGAGTAAGAACTTAGTTCGAGGAGTTGAACAATTGTTTGATAAAGGGCATACAAACCAATT

CACCATCTTTCCAGGTACTTGTTATTCAATATAATTCCGGTTTTGAATGAATTGATTTTTG

TTAAGTTGGTTCTGGATAAACGTGCGTTAAATATAATATGATTTATGTTTGGATACGTTTA

TGTTAAAGTGAGTTGAACATAAATTGGAGACTAAATATCAATTGCAGAGGCAAAAGCTTCA

AATTCTAATTTCAAGTTAGAATCAAATCAATTCTACTCGTAAGCATCTAAATGTTTCAAAA

CCAATTCTACATCTCTAGAACTAAATTTGCATTCAAACATTTTCTGCTACAGATGATTGTA

AGATGTCAGTTGATACACCAAATCATCAAGCAACCTTATCCCTGCAATCACCATTTGCTGC

CGAGCCACTTAATCGATTTGAGCTAGGTTTTAACCAGTCTATGGTAAATCTTCTTATTAAA

GCAATCCCTTTGATCATGTTTTAGTATTTTACTTTTGATGTACATAAATTCATTATGTTTT

TATTATCCTTTCCATGCTAGATCTGTGCAAAATATCCGTATATAGATCAATTTTACGGGCT

CTTCTCGACTTATGGACCTCAAATCTCGGTTTGTCTTCACCTCGGCTATTTTATTAATCTT

TGTATATATCATGCAATGTCTATAAGTACTTGAATGTGAATTTGTTTTATTTACAGTGTGT

ATGCTTACATGAGAAAGCCAGAAATCTGTTTACAACTATCTTGTTAGTATTCTTCTAAATC

TTTCAAATGTAGAGTTGATTATGAAATTTGTTGTTAATGAGTTTTCGGCTTTATGAATGAA

TTGTAAGACAAGTAATGAAACAAAAACTAGAAGAAAATTGAATTATATGATGCAATCTGTG

TTGAAGTTTTTGCATGTATTCAAGTTCTGCTTTTAGTTACTATTTGTTTATCTGCTGCAAT

ATTTCATCAATTATTATTCATGTATGTACCAATATTTAATAAATTAGGGGCGAATTATGCT

TCCGCTTAGCATGACATCTGACGACGGACCAACATACGTGAATGCTAAGCAATACCATGGA

ATCATCAGACGCAGGCATTCTCGTGCCAAAGCTGTGCTTCAGAATAAATTGATAAAGCGTA

ACAAGGTATGAAACTGAGTATTTTTCCTCACAACAATGTTCGAAAACTTGTGAAATAGTAT

ATTTTTCCTTTTACCCTTTTTATACCTATATTAAGGTTTTGTATTTGTCTTGCAGCCATAT

ATGCACGAATCGCGTCATCTACATGCAATGCGTAGACCAAGAGGATGCGGTGGTCGTTTCT

TGAACACAAAAGTTTCTGCTAATGGAAACGGTAAAAGCGGGAGTGAAGAGAACGGAAACAT

TGGTGGCCTACAGCTGCAGTCCAGTGGTTCTCAGAGTTCCGAAGTCTTACAATCTGAGGTT

GGAACTTTAAATTCGTCGAAGGAGACAAACGGAGGCAGTCCAAATGTCTCGGGGTCAGAGG

TGACTAGCATGTATACACAGGGAGGTCTTGATAGCTTTACTGTCAATCATATCGGATCTAC

TGTCCACTCTTTGGGAGACATGATCGATACTGGACACGGTATCGTCATGCCAACAAAATGG

TTTGCAGCAGCTGGCAGACAGCTGCTGGAACCATAAGTTTCGATTCAGAAAGGAAACAAGT

GGGTTTGGTACAATGTGAAATATTTTGCACCAAACTCATCCTTTCCGAGACCAGATGAAGA

AGCTATGTTTCAGTTTGTTGTGTTTACTACGACAAATTTAGTTTCGGAAGACTACTTTTCC

ATCTGGTGCTCAGGCAACTCATTCTTGGCTTATTCTCAGGAAACTCATCCTTGGCTCGTAA

TATTTAGTAGTATTGTCATTGTCTTCCCGCGCAGGCTTTCCGTGGCATGGTAGGCATGCTA

ATGACTTTGGTATTTTCATGCAGTTATAACTATGATGTGTCTTTGTTTGTTGTTAAAATAA

AAAACATGAACTCTAGCTAGGTGCATGTGTGTGTTTTTAATCTTGTCTACTAAGTTTGGTG

TTTTGTAATGGATTTCTGACTTTATGGAGCAATGTATTGTAACTCTACTAAGAAGTGTAAC

ATTTTATTTCTCCCTCTCTAAGGATTGTATAAGAACCTCTTATTTTCAGACTCTACTTAAT

CCTATTTTCTATGTCTGTATGATTTTTATATTTCTAGGACAATCAAATTGGCTTGTAGAAG

CTCAAAAGCATGCTCACAAAGTAGGTACTTATGTAGGGAACTTCGTACTCTAATAATAACT

GGTTATTAACGTTATAATTAAATGCAAAATTTGATAAGTAGTAGGGTTTGGTAAGTAATAT

AACAACACCATGTTGGCTTGGGATTCTGAATTGTGTTATTAGAGTACAACTCTTAAATAAT

GATCGTGATGTAAATAATTTAAATTTGAAGTTACTAGATAAAAAGATAACATTATGTAATA

TATTATCAAATATATACTAAAAATATAATTTTCATCTATCTCAATTGAAAAAATATCTGGG

TTTTGAGTCTATTGTCATTGACGA

>HM031

AAAAAAATAAAGGTTCTATACTCATTAAACAAAGTGCCAAATTCAGAGATACTACTTCCT

TTGGATAATTTGTATCGTCACCAACGATCTTTGAGTTATTTCAAAAATCACATTCTCAATT

TTGTGGTCCAACATTCACAAAATGGTTTGTCGGGCTTTAAGTCTAGTAATTGTGAAACATG

CATATGCATGAGGTAATTAATTGATAATCTGAAAAGGTTAAATAGTTAATCAATTGTACTT

TGGTTATTTTTAATTTGTTCTCTATAATTTTTCAGTCAAAATTGAATTTGCTTTAACCATT

TAGTGTAGTAGTATGATATTACTTGCTCTACATTCTTGGATCTAAAAGTCTAATTAGTATC

GTATAAAAAATATTCTAGTTAATTAAGATACTTTTGTTCATTCTAATTATTCTTGGATTAC

TTGTGAAACTTTTAGGGATGAACAAGAAAGTCCACTAAAGAGGCTTCTTTTTGCTAGGAGT

CACTAGTTAGTTTTTTTTTTTTTTGACAAAAGTTAACCATTTTTTTTTATCGCATGGTAGG

AAAAAAAAAATAGAGATTTCCTTTGGTACTCATAAAAAAAAAAGTTGGATGGATAATGGAT

TAATCATAACTTATTTAACATAAAATGAACTAAGAGAACATTTCATATTTGGGTTTATTTT

GTAACATAAATATTAATATCAATGTTTGAAAGAGTTTTTAACAATAATTTGTAAAAGAAAG

CATAATATATCTATCAAAATAATTGTAAAAGAAAAAATTTCTTTTGGTGGTATGACATGGA

AAAATAATTAATTCTTATTAGATGAAAGTGTAAAATTAATTTGCATTAACAAAGCATATTT

CATTAAACTCCTTTTAGTTTTTGTTGGAAAAAATAAATTTATTGTTACTCAAAAAATAAAA

GTGTACAATTAATATATTTACTCAAAAGTCCCCTAAATGGGATGTAATTATCAAGGGATGT

GAGCGGTTTAACTTTAAGTAATGAATCAACTGTTGCATAATCACTTTGAAAGAAACTTGAG

ATTCTTCAATTAGCAAAATCTTGAATAGAATAATGAGATTATTCCTAATGCTAATGATACC

CACCTCTCCTATAAGTTGATGGGGCAGAACAAATTATGCACTCAGGACAACTTTTCTCAAA

TTTTGAAAACTTACCACACGAAGAACACACTTCCACTTGCACAAGTAAGAATATAGTATAA

CAATAAATTTACCACTCATAGCTCAATGGATATTAAAATATTGCTAATCTTTTATAGACAG

ATGTTGGCAAATTAGTATAAATTAGTTATTATATTAGTTTTTAAGGTTCAATCTACTTTTT

AAAACTCTTATTAAGTTGTTTAGCTTGCCAACCAAAGACTAAATTATCTTTATTTGTTAGG

AAATAAAAGCACTTATTTTATTTTCTCCATATTTGTTTCCTCTTTGTTCCATTACTAAAAA

AATTATAATATATTAATAACAAAAAAATTTGGGGAGGGAATGTTATTTTTAAAAAAGATGT

TTTATTTATATTTAAGTGTTTTATTGAAATTCCTCGTGAGTTTAGCTCAATTGGTATGGAC

AATACATAATATATGCAAAATTCGAGGTTCAAACCTTGAGCACCAAAAAAAAAAGTGTTTT

ATTGAAAAATATCAAGAACTTGAAAACAAATTTCCTTTTTATGAACTAGATATTGTCACAT

AGACAACTTAAGAATTGACATTTTTTTTAACACTATTGTATTTTCTGAACTCGACTTTAAC

TCGAGATCAATGAGTAAACTAAAAAAACTCGTATTATTTTATCTAAGTGTTCTTAGTGTGA

GAAAACATTATAATTGTTTGCTTAGTCTATTTCTCCCTATATTCTCACCCTCATTCTTGGT

TTGCACATAGAAAGGAGAAAAAAAAAATTAGAGAAGAGTTGAATGGAACAGAAAGAAAGAG

CAAGCAAAATAATCACTAAAAAAAATTCCCCTAAAAGAAAATCACTGAAAAATATTATCCA

CCTCTATTTTAAATAATAATAATAATGAAAACAACAATATATTTTTTTATAGATTTTCTCT

ACTAGAGGTAATTTATTTTAAACACTAAATGTCCACACCTCTAACAAAAAAAATGCATAAT

ATTATAATAGATTATTGTAAGAAAGAGTGACAAATGTTGAATTAGTTATGGAATGAAGAGT

GAGAGAGATTATACAAATAAATAAATATAAATATAAATATAAATAAAGAATAGAGAAGAAC

ATAGTACTTGAATGAAGAGATTTGGCTTGTACTTCCATTACACTTGTAAGCTTTCTACTTC

CTTCACATTCCTTCTCTTTCTCTATCTTTCCTTTTATGATATGTGTACTTTGTTATTGCTT

CATTTACTACCGACATGACTCCAACCTATTAGGGCTTTATCTGAATAATGTTAGTTATTTT

CTCTCATTTTCTTTGCTTCTTCTTATTCTTGGAACTTATAAAGGGTCTTGTCAATTCTATC

ATTCTCTTTTGTATACTTACAAATATAAATATTCTCTTTAGCTTCATTTTCATGTCTTCTT

CTACTAATAAGCTATGGAAATTGAAGTTTTTGTTTTCATGTTTATTTTTCACTTCAGCTCC

TCTTTTTTGATGTTGATGCTGAAGATTGAATAAAAACTGAGGTGGTATGTATGAGAACAAG

AACATTTTTCATTCATCTCTGTTATTTGAACATCTAAATTTTAAACAACTATCGCGACAAT

TTTTTAAGTGGTTGAAGTATTTTAAATAGGTGGTTAATTACATTAAGTAGATAGTTAATTG

ATATATCAGGTGTCAAAACTCATTAACCATCAACATAACTTCATTAGCCATAAATTTTTGA

CGTGATTAACCAAATATTGTAGATGCTCAATTACATGTAGATTTGATGGAGTTGTCCACAA

TTTCTCATATATCTATTAACTAATAACCATCTACCGAATATAATTAACCATATATTAGAAT

ATCATTAACCTCTAAAACAGTTGTCGCGAAAATTGGTTGTAAAAAAATCATTTCTATTTTT

TACGCCACTAGTTCTCTTGTTATTTCTCTTTGTTGGAAAGTTGTTGTTTTTAACTTTGAGC

TTAGTCTTATTAATTAACTTGTAATGGTAACAACATTTTTCTATCTTTGTTTGAAGGAAGT

AGTTACTGTGTCAACTTTAGTAATTAATTTGGATCATTTTGGAGGCAAAATATGGCTATGC

AACCTGTTTATTTTAAAGAACATGAAGGAAATGTCCACAATTCTGTTGGACAGTTTTCATC

TGTGACTTCAGCACCATGGTGGAGTAATGCCTATGGATCTCAACCTGTTTATGGAGGAGAC

TCTTGTGGCCAAATGAAACCTTTTTCACTAGAGCTTTCCAACTACATAGACCAACTTGCTC

CGAGTAAGAACTTAGTTCGAGGAGTTGAACAATTGTTTGATAAAGGGCATACAAACCAATT

CACCATCTTTCCAGGTACTTGTTATTCAATATAATTCCGGTTTTGAATGAATTGATTTTTG

TTAAGTTGGTTCTGGATAAACGTGCGTTAAATATAATATGATTTATGTTTGGATACGTTTA

TGTTAAAGTGAGTTGAACATAAATTGGAGACTAAATATCAATTGCAGAGGCAAAAGCTTCA

AATTCTAATTTCAAGTTAGAATCAAATCAATTCTACTCGTAAGCATCTAAATGTTTCAAAA

CCAATTCTACATCTCTAGAACTAAATTTGCATTCAAACATTTTCTGCTACAGATGATTGTA

AGATGTCAGTTGATACACCAAATCATCAAGCAACCTTATCCCTGCAATCACCATTTGCTGC

CGAGCCACTTAATCGATTTGAGCTAGGTTTTAACCAGTCTATGGTAAATCTTCTTATTAAA

GCAATCCCTTTGATCATGTTTTAGTATTTTACTTTTGATGTACATAAATTCATTATGTTTT

TATTATCCTTTCCATGCTAGATCTGTGCAAAATATCCGTATATAGATCAATTTTACGGGCT

CTTCTCGACTTATGGACCTCAAATCTCGGTTTGTCTTCACCTCGGCTATTTTATTAATCTT

TGTATATATCATGCAATGTCTATAAGTACTTGAATGTGAATTTGTTTTATTTACAGTGTGT

ATGCTTACATGAGAAAGCCAGAAATCTGTTTACAACTATCTTGTTAGTATTCTTCTAAATC

TTTCAAATGTAGAGTTGATTATGAAATTTGTTGTTAATGAGTTTTCGGCTTTATGAATGAA

TTGTAAGACAAGTAATGAAACAAAAACTAGAAGAAAATTGAATTATATGATGCAATCTGTG

TTGAAGTTTTTGCATGTATTCAAGTTCTGCTTTTAGTTACTATTTGTTTATCTGCTGCAAT

ATTTCATCAATTATTATTCATGTATGTACCAATATTTAATAAATTAGGGGCGAATTATGCT

TCCGCTTAGCATGACATCTGACGACGGACCAACATACGTGAATGCTAAGCAATACCATGGA

ATCATCAGACGCAGGCATTCTCGTGCCAAAGCTGTGCTTCAGAATAAATTGATAAAGCGTA

ACAAGGTATGAAACTGAGTATTTTTCCTCACAACAATGTTCGAAAACTTGTGAAATAGTAT

ATTTTTCCTTTTACCCTTTTTATACCTATATTAAGGTTTTGTATTTGTCTTGCAGCCATAT

ATGCACGAATCGCGTCATCTACATGCAATGCGTAGACCAAGAGGATGCGGTGGTCGTTTCT

TGAACACAAAAGTTTCTGCTAATGGAAACGGTAAAAGCGGGAGTGAAGAGAACGGAAACAT

TGGTGGCCTACAGCTGCAGTCCAGTGGTTCTCAGAGTTCCGAAGTCTTACAATCTGAGGTT

GGAACTTTAAATTCGTCGAAGGAGACAAACGGAGGCAGTCCAAATGTCTCGGGGTCAGAGG

TGACTAGCATGTATACACAGGGAGGTCTTGATAGCTTTACTGTCAATCATATCGGATCTAC

TGTCCACTCTTTGGGAGACATGATCGATACTGGACACGGTATCGTCATGCCAACAAAATGG

TTTGCAGCAGCTGGCAGACAGCTGCTGGAACCATAAGTTTCGATTCAGAAAGGAAACAAGT

GGGTTTGGTACAATGTGAAATATTTTGCACCAAACTCATCCTTTCCGAGACCAGATGAAGA

AGCTATGTTTCAGTTTGTTGTGTTTACTACGACAAATTTAGTTTCGGAAGACTACTTTTCC

ATCTGGTGCTCAGGCAACTCATTCTTGGCTTATTCTCAGGAAACTCATCCTTGGCTCGTAA

TATTTAGTAGTATTGTCATTGTCTTCCCGCGCAGGCTTGCCGTGGCATGGTAGGCATGCTA

ATGACTTTGGTATTTTCATGCAGTTATAACTATGATGTGTCTTTGTTTGTTGTTAAAATAA

AAAACATGAACTCTAGCTAGGTGCATGTGTGTGTTTTTAATCTTGTCTACTAAGTTTGGTG

TTTTGTAATGGATTTCTGACTTTATGGAGCAATGTATTGTAACTCTACTAAGAAGTGTAAC

ATTTTATTTCTCCCTCTCTAAGGATTGTATAAGAACCTCTTATTTTCAGACTCTACTTAAT

CCTATTTTCTATGTCTGTATGATTTTTATATTTCTAGGACAATCAAATTGGCTTGTAGAAG

CTCAAAAGCATGCTCACAAAGTAGGTACTTATGTAGGGAACTTCGTACTCTAATAATAACT

GGTTATTAACGTTATAATTAAATGCAAAATTTGATAAGTAGTAGGGTTTGGTAAGTAATAT

AACAACACCATGTTGGCTTGGGATTCTGAATTGTGTTATTAGAGTACAACTCTTAAATAAT

GATCGTGATGTAAATAATTTAAATTTGAAGTTACTAGATAAAAAGATAACATTATGTAATA

TATTATCAAATATATACTAAAAATATAATTTTCATCTATCTCAATTGAAAAAATATCCGGG

TTTTGAGTCTATTGTCAATGACGA

>HM032

AAAAAAATAAAGGTTCTATACTCATTAAACAAAGTGCCAAATTCAGAGATACTACTTCCT

TTGGATAATTTGTATCGTCACCAACGATCTTTGAGTTATTTCAAAAATCACATTCTCAATT

TTGTGGTCCAACATTCACAAAATGGTTTGTCGGGCTTTAAGTCTAGTAATTGTGAAACATG

CATATGCATGAGGTAATTAATTGATAATCTGAAAAGGTTAAATAGTTAATCAATTGTACTT

TGGTTATTTTTAATTTGTTCTCTATAATTTTTCAGTCAAAATTGAATTTGCTTTAACCATT

TAGTGTAGTAGTATGATATTACTTGCTCTACATTCTTGGATCTAAAAGTCTAATTAGTATC

GTATAAAAAATATTCTAGTTAATTAAGATACTTTTGTTCATTCTAATTATTCTTGGATTAC

TTGTGAAACTTTTAGGGATGAACAAGAAAGTCCACTAAAGAGGCTTCTTTTTGCTAGGAGT

CACTAGTTAGTTTTTTTTTTTTTTGACAAAAGTTAACCATTTTTTTTTATCGCATGGTAGG

AAAAAAAAAATAGAGATTTCCTTTGGTACTCATAAAAAAAAAAGTTGGATGGATAATGGAT

TAATCATAACTTATTTAACATAAAATGAACTAAGAGAACATTTGATATTTGGGTTTATTTT

GTAACATAAATATTAATATCAATGTTTGAAAGAGTTTTTAACAATAATTTGTAAAAGAAAG

CATAATATATCTATCAAAATAATTGTAAAAGAAAAAATTTCTTTTGGTGGTATGACATGGA

AAAATAATTAATTCTTATTAGATGAAAGTGTAAAATTAATTTGCATTAACAAAGCATATTT

CATTAAACTCCTTTTAGTTTTTGTTGGAAAAAATAAATTTATTGTTACTCAAAAAATAAAA

GTGTACAATTAATATATTTACTCAAAAGTCCCCTAAATGGGATGTAATTATCAAGGGATGT

GAGCGGTTTAACTTTAAGTAATGAATCAACTGTTGCATAATCACTTTGAAAGAAACTTGAG

ATTCTTCAATTAGCAAAATCTTGAATAGAATAATGAGATTATTCCTAATGCTAATGATACC

CACCTCTCCTATAAGTTGATGGGGCAGAACAAATTATGCACTCAGGACAACTTTTCTCAAA

TTTTGAAAACTTACCACACGAAGAACACACTTCCACTTGCACAAGTAAGAATATAGTATAA

CAATAAATTTACCACTCATAGCTCAATGGATATTAAAATATTGCTAATCTTTTATAGACAG

ATGTTGGCAAATTAGTATAAATTAGTTATTATATTAGTTTTTAAGGTTCAATCTACTTTTT

AAAACTCTTATTAAGTTGTTTAGCTTGCCAACCAAAGACTAAATTATCTTTATTTGTTAGG

AAATAAAAGCACTTATTTTATTTTCTCCATATTTGTTTCCTCTTTGTTCCATTACTAAAAA

AATTATAATATATTAATAACAAAAAAATTTGGGGAGGGAATGTTATTTTTAAAAAAGATGT

TTTATTTATATTTAAGTGTTTTATTGAAATTCCTCGTGAGTTTAGCTCAATTGGTATGGAC

AATACATAATATATGCAAAATTCGAGGTTCAAACCTTGAGCACCAAAAAAAAAAGTGTTTT

ATTGAAAAATATCAAGAACTTGAAAACAAATTTCCTTTTTATGAACTAGATATTGTCACAT

AGACAACTTAAGAATTGACATTTTTTTTAACACTATTGTATTTTCTGAACTCGACTTTAAC

TCGAGATCAATGAGTAAACTAAAAAAACTCGTATTATTTTATCTAAGTGTTCTTAGTGTGA

GAAAACATTATAATTGTTTGCTTAGTCTATTTCTCCCTATATTCTCACCCTCATTCTTGGT

TTGCACATAGAAAGGAGAAAAAAAAAATTAGAGAAGAGTTGAATGGAACAGAAAGAAAGAG

CAAGCAAAATAATCACTAAAAAAAATTCCCCTAAAAGAAAATCACTGAAAAATATTATCCA

CCTCTATTTTAAATAATAATAATAATGAAAACAACAATATATTTTTTTATAGATTTTCTCT

ACTAGAGGTAATTTATTTTAAACACTAAATGTCCACACCTCTAACAAAAAAAATGCATAAT

ATTATAATAGATTATTGTAAGAAAGAGTGACAAATGTTGAATTAGTTATGGAATGAAGAGT

GAGAGAGATTATACAAATAAATAAATATAAATATAAATATAAATAAAGAATAGAGAAGAAC

ATAGTACTTGAATGAAGAGATTTGGCTTGTACTTCCATTACACTTGTAAGCTTTCTACTTC

CTTCACATTCCTTCTCTTTCTCTATCTTTCCTTTTATGATATGTGTACTTTGTTATTGCTT

CATTTACTACCGACATGACTCCAACCTATTAGGGCTTTATCTGAATAATGTTAGTTATTTT

CTCTCATTTTCTTTGCTTCTTCTTATTCTTGGAACTTATAAAGGGTCTTGTCAATTCTATC

ATTCTCTTTTGTATACTTACAAATATAAATATTCTCTTTAGCTTCATTTTCATGTCTTCTT

CTACTAATAAGCTATGGAAATTGAAGTTTTTGTTTTCATGTTTATTTTTCACTTCAGCTCC

TCTTTTTTGATGTTGATGCTGAAGATTGAATAAAAACTGAGGTGGTATGTATGAGAACAAG

AACATTTTTCATTCATCTCTGTTATTTGAACATCTAAATTTTAAACAACTATCGCGACAAT

TTTTTAAGTGGTTGAAGTATTTTAAATAGGTGGTTAATTACATTAAGTAGATAGTTAATTG

ATATATCAGGTGTCAAAACTCATTAACCATCAACATAACTTCATTAGCCATAAATTTTTGA

CGTGATTAACCAAATATTGTAGATGCTCAATTACATGTAGATTTGATGGAGTTGTCCACAA

TTTCTCATATATCTATTAACTAATAACCATCTACCGAATATAATTAACCATATATTAGAAT

ATCATTAACCTCTAAAACAGTTGTCGCGAAAATTGGTTGTAAAAAAATCATTTCTATTTTT

TACGCCACTAGTTCTCTTGTTATTTCTCTTTGTTGGAAAGTTGTTGTTTTTAACTTTGAGC

TTAGTCTTATTAATTAACTTGTAATGGTAACAACATTTTTCTATCTTTGTTTGAAGGAAGT

AGTTACTGTGTCAACTTTAGTAATTAATTTGGATCATTTTGGAGGCAAAATATGGCTATGC

AACCTGTTTATTTTAAAGAACATGAAGGAAATGTCCACAATTCTGTTGGACAGTTTTCATC

TGTGACTTCAGCACCATGGTGGAGTAATGCCTATGGATCTCAACCTGTTTATGGAGGAGAC

TCTTGTGGCCAAATGAAACCTTTTTCACTAGAGCTTTCCAACTACATAGACCAACTTGCTC

CGAGTAAGAACTTAGTTCGAGGAGTTGAACAATTGTTTGATAAAGGGCATACAAACCAATT

CACCATCTTTCCAGGTACTTGTTATTCAATATAATTCCGGTTTTGAATGAATTGATTTTTG

TTAAGTTGGTTCTGGATAAACGTGCGTTAAATATAATATGATTTATGTTTGGATACGTTTA

TGTTAAAGTGAGTTGAACATAAATTGGAGACTAAATATCAATTGCAGAGGCAAAAGCTTCA

AATTCTAATTTCAAGTTAGAATCAAATCAATTCTACTCGTAAGCATCTAAATGTTTCAAAA

CCAATTCTACATCTCTAGAACTAAATTTGCATTCAAACATTTTCTGCTACAGATGATTGTA

AGATGTCAGTTGATACACCAAATCATCAAGCAACCTTATCCCTGCAATCACCATTTGCTGC

CGAGCCACTTAATCGATTTGAGCTAGGTTTTAACCAGTCTATGGTAAATCTTCTTATTAAA

GCAATCCCTTTGATCATGTTTTAGTATTTTACTTTTGATGTACATAAATTCATTATGTTTT

TATTATCCTTTCCATGCTAGATCTGTGCAAAATATCCGTATATAGATCAATTTTACGGGCT

CTTCTCGACTTATGGACCTCAAATCTCGGTTTGTCTTCACCTCGGCTATTTTATTAATCTT

TGTATATATCATGCAATGTCTATAAGTACTTGAATGTGAATTTGTTTTATTTACAGTGTGT

ATGCTTACATGAGAAAGCCAGAAATCTGTTTACAACTATCTTGTTAGTATTCTTCTAAATC

TTTCAAATGTAGAGTTGATTATGAAATTTGTTGTTAATGAGTTTTCGGCTTTATGAATGAA

TTGTAAGACAAGTAATGAAACAAAAACTAGAAGAAAATTGAATTATATGATGCAATCTGTG

TTGAAGTTTTTGCATGTATTCAAGTTCTGCTTTTAGTTACTATTTGTTTATCTGCTGCAAT

ATTTCATCAATTATTATTCATGTATGTACCAATATTTAATAAATTAGGGGCGAATTATGCT

TCCGCTTAGCATGACATCTGACGACGGACCAACATACGTGAATGCTAAGCAATACCATGGA

ATCATCAGACGCAGGCATTCTCGTGCCAAAGCTGTGCTTCAGAATAAATTGATAAAGCGTA

ACAAGGTATGAAACTGAGTATTTTTCCTCACAACAATGTTCGAAAACTTGTGAAATAGTAT

ATTTTTCCTTTTACCCTTTTTATACCTATATTAAGGTTTTGTATTTGTCTTGCAGCCATAT

ATGCACGAATCGCGTCATCTACATGCAATGCGTAGACCAAGAGGATGCGGTGGTCGTTTCT

TGAACACAAAAGTTTCTGCTAATGGAAACGGTAAAAGCGGGAGTGAAGAGAACGGAAACAT

TGGTGGCCTACAGCTGCAGTCCAGTGGTTCTCAGAGTTCCGAAGTCTTACAATCTGAGGTT

GGAACTTTAAATTCGTCGAAGGAGACAAACGGAGGCAGTCCAAATGTCTCGGGGTCAGAGG

TGACTAGCATGTATACACAGGGAGGTCTTGATAGCTTTACTGTCAATCATATCGGATCTAC

TGTCCACTCTTTGGGAGACATGATCGATACTGGACACGGTATCGTCATGCCAACAAAATGG

TTTGCAGCAGCTGGCAGACAGCTGCTGGAACCATAAGTTTCGATTCAGAAAGGAAACAAGT

GGGTTTGGTACAATGTGAAATATTTTGCACCAAACTCATCCTTTCCGAGACCAGATGAAGA

AGCTATGTTTCAGTTTGTTGTGTTTACTACGACAAATTTAGTTTCGGAAGACTACTTTTCC

ATCTGGTGCTCAGGCAACTCATTCTTGGCTTATTCTCAGGAAACTCATCCTTGGCTCGTAA

TATTTAGTAGTATTGTCATTGTCTTCCCGCGCAGGCTTGCCGTGGCATGGTAGGCATGCTA

ATGACTTTGGTATTTTCATGCAGTTATAACTATGATGTGTCTTTGTTTGTTGTTAAAATAA

AAAACATGAACTCTAGCTAGGTGCATGTGTGTGTTTTTAATCTTGTCTACTAAGTTTGGTG

TTTTGTAATGGATTTCTGACTTTATGGAGCAATGTATTGTAACTCTACTAAGAAGTGTAAC

ATTTTATTTCTCCCTCTCTAAGGATTGTATAAGAACCTCTTATTTTCAGACTCTACTTAAT

CCTATTTTCTATGTCTGTATGATTTTTATATTTCTAGGACAATCAAATTGGCTTGTAGAAG

CTCAAAAGCATGCTCACAAAGTAGGTACTTATGTAGGGAACTTCGTACTCTAATAATAACT

GGTTATTAACGTTATAATTAAATGCAAAATTTGATAAGTAGTAGGGTTTGGTAAGTAATAT

AACAACACCATGTTGGCTTGGGATTCTGAATTGTGTTATTAGAGTACAACTCTTAAATAAT

GATCGTGATGTAAATAATTTAAATTTGAAGTTACTAGATAAAAAGATAACATTATGTAATA

TATTATCAAATATATACTAAAAATATAATTTTCATCTATCTCAATTGAAAAAATATCCGGG

TTTTGAGTCTATTGTCAATGACGA

>HM033

AAAAAAATAAAGGTTCTATACTCATTAAACAAAGTGCCAAATTCAGAGATACTACTTCCT

TTGGATAATTTGTATCGTCACCAACGATCTTTGAGTTATTTCAAAAATCACATTCTCAATT

TTGTGGTCCAACATTCACAAAATGGTTTGTCGGGCTTTAAGTCTAGTAATTGTGAAACATG

CATATGCATGAGGTAATTAATTGATAATCTGAAAAGGTTAAATAGTTAATCAATTGTACTT

TGGTTATTTTTAATTTGTTCTCTATAATTTTTCAGTCAAAATTGAATTTGCTTTAACCATT

TAGTGTAGTAGTATGATATTACTTGCTCTACATTCTTGGATCTAAAAGTCTAATTAGTATC

GTATAAAAAATATTCTAGTTAATTAAGATACTTTTGTTCATTCTAATTATTCTTGGATTAC

TTGTGAAACTTTTAGGGATGAACAAGAAAGTCCACTAAAGAGGCTTCTTTTTGCTAGGAGT

CACTAGTTAGTTTTTTTTTTTTTTGACAAAAGTTAACCATTTTTTTTTATCGCATGGTAGG

AAAAAAAAAATAGAGATTTCCTTTGGTACTCATAAAAAAAAAAGTTGGATGGATAATGGAT

TAATCATAACTTATTTAACATAAAATGAACTAAGAGAACATTTGATATTTGGGTTTATTTT

GTAACATAAATATTAATATCAATGTTTGAAAGAGTTTTTAACAATAATTTGTAAAAGAAAG

CATAATATATCTATCAAAATAATTGTAAAAGAAAAAATTTCTTTTGGTGGTATGACATGGA

AAAATAATTAATTCTTATTAGATGAAAGTGTAAAATTAATTTGCATTAACAAAGCATATTT

CATTAAACTCCTTTTAGTTTTTGTTGGAAAAAATAAATTTATTGTTACTCAAAAAATAAAA

GTGTACAATTAATATATTTACTCAAAAGTCCCCTAAATGGGATGTAATTATCAAGGGATGT

GAGCGGTTTAACTTTAAGTAATGAATCAACTGTTGCATAATCACTTTGAAAGAAACTTGAG

ATTCTTCAATTAGCAAAATCTTGAATAGAATAATGAGATTATTCCTAATGCTAATGATACC

CACCTCTCCTATAAGTTGATGGGGCAGAACAAATTATGCACTCAGGACAACTTTTCTCAAA

TTTTGAAAACTTACCACACGAAGAACACACTTCCACTTGCACAAGTAAGAATATAGTATAA

CAATAAATTTACCACTCATAGCTCAATGGATATTAAAATATTGCTAATCTTTTATAGACAG

ATGTTGGCAAATTAGTATAAATTAGTTATTATATTAGTTTTTAAGGTTCAATCTACTTTTT

AAAACTCTTATTAAGTTGTTTAGCTTGCCAACCAAAGACTAAATTATCTTTATTTGTTAGG

AAATAAAAGCACTTATTTTATTTTCTCCATATTTGTTTCCTCTTTGTTCCATTACTAAAAA

AATTATAATATATTAATAACAAAAAAATTTGGGGAGGGAATGTTATTTTTAAAAAAGATGT

TTTATTTATATTTAAGTGTTTTATTGAAATTCCTCGTGAGTTTAGCTCAATTGGTATGGAC

AATACATAATATATGCAAAATTCGAGGTTCAAACCTTGAGCACCAAAAAAAAAAGTGTTTT

ATTGAAAAATATCAAGAACTTGAAAACAAATTTCCTTTTTATGAACTAGATATTGTCACAT

AGACAACTTAAGAATTGACATTTTTTTTAACACTATTGTATTTTCTGAACTCGACTTTAAC

TCGAGATCAATGAGTAAACTAAAAAAACTCGTATTATTTTATCTAAGTGTTCTTAGTGTGA

GAAAACATTATAATTGTTTGCTTAGTCTATTTCTCCCTATATTCTCACCCTCATTCTTGGT

TTGCACATAGAAAGGAGAAAAAAAAAATTAGAGAAGAGTTGAATGGAACAGAAAGAAAGAG

CAAGCAAAATAATCACTAAAAAAAATTCCCCTAAAAGAAAATCACTGAAAAATATTATCCA

CCTCTATTTTAAATAATAATAATAATGAAAACAACAATATATTTTTTTATAGATTTTCTCT

ACTAGAGGTAATTTATTTTAAACACTAAATGTCCACACCTCTAACAAAAAAAATGCATAAT

ATTATAATAGATTATTGTAAGAAAGAGTGACAAATGTTGAATTAGTTATGGAATGAAGAGT

GAGAGAGATTATACAAATAAATAAATATAAATATAAATATAAATAAAGAATAGAGAAGAAC

ATAGTACTTGAATGAAGAGATTTGGCTTGTACTTCCATTACACTTGTAAGCTTTCTACTTC

CTTCACATTCCTTCTCTTTCTCTATCTTTCCTTTTATGATATGTGTACTTTGTTATTGCTT

CATTTACTACCGACATGACTCCAACCTATTAGGGCTTTATCTGAATAATGTTAGTTATTTT

CTCTCATTTTCTTTGCTTCTTCTTATTCTTGGAACTTATAAAGGGTCTTGTCAATTCTATC

ATTCTCTTTTGTATACTTACAAATATAAATATTCTCTTTAGCTTCATTTTCATGTCTTCTT

CTACTAATAAGCTATGGAAATTGAAGTTTTTGTTTTCATGTTTATTTTTCACTTCAGCTCC

TCTTTTTTGATGTTGATGCTGAAGATTGAATAAAAACTGAGGTGGTATGTATGAGAACAAG

AACATTTTTCATTCATCTCTGTTATTTGAACATCTAAATTTTAAACAACTATCGCGACAAT

TTTTTAAGTGGTTGAAGTATTTTAAATAGGTGGTTAATTACATTAAGTAGATAGTTAATTG

ATATATCAGGTGTCAAAACTCATTAACCATCAACATAACTTCATTAGCCATAAATTTTTGA

CGTGATTAACCAAATATTGTAGATGCTCAATTACATGTAGATTTGATGGAGTTGTCCACAA

TTTCTCATATATCTATTAACTAATAACCATCTACCGAATATAATTAACCATATATTAGAAT

ATCATTAACCTCTAAAACAGTTGTCGCGAAAATTGGTTGTAAAAAAATCATTTCTATTTTT

TACGCCACTAGTTCTCTTGTTATTTCTCTTTGTTGGAAAGTTGTTGTTTTTAACTTTGAGC

TTAGTCTTATTAATTAACTTGTAATGGTAACAACATTTTTCTATCTTTGTTTGAAGGAAGT

AGTTACTGTGTCAACTTTAGTAATTAATTTGGATCATTTTGGAGGCAAAATATGGCTATGC

AACCTGTTTATTTTAAAGAACATGAAGGAAATGTCCACAATTCTGTTGGACAGTTTTCATC

TGTGACTTCAGCACCATGGTGGAGTAATGCCTATGGATCTCAACCTGTTTATGGAGGAGAC

TCTTGTGGCCAAATGAAACCTTTTTCACTAGAGCTTTCCAACTACATAGACCAACTTGCTC

CGAGTAAGAACTTAGTTCGAGGAGTTGAACAATTGTTTGATAAAGGGCATACAAACCAATT

CACCATCTTTCCAGGTACTTGTTATTCAATATAATTCCGGTTTTGAATGAATTGATTTTTG

TTAAGTTGGTTCTGGATAAACGTGCGTTAAATATAATATGATTTATGTTTGGATACGTTTA

TGTTAAAGTGAGTTGAACATAAATTGGAGACTAAATATCAATTGCAGAGGCAAAAGCTTCA

AATTCTAATTTCAAGTTAGAATCAAATCAATTCTACTCGTAAGCATCTAAATATTTCAAAA

CCAATTCTACATCTCTAGAACTAAATTTGCATTCAAACATTTTCTGCTACAGATGATTGTA

AGATGTCAGTTGATACACCAAATCATCAAGCAACCTTATCCCTGCAATCACCATTTGCTGC

CGAGCCACTTAATCGATTTGAGCTAGGTTTTAACCAGTCTATGGTAAATCTTCTTATTAAA

GCAATCCCTTTGATCATGTTTTAGTATTTTACTTTTGATGTACATAAATTCATTATGTTTT

TATTATCCTTTCCATGCTAGATCTGTGCAAAATATCCGTATATAGATCAATTTTACGGGCT

CTTCTCGACTTATGGACCTCAAATCTCGGTTTGTCTTCACCTCGGCTATTTTATTAATCTT

TGTATATATCATGCAATGTCTATAAGTACTTGAATGTGAATTTGTTTTATTTACAGTGTGT

ATGCTTACATGAGAAAGCCAGAAATCTGTTTACAACTATCTTGTTAGTATTCTTCTAAATC

TTTCAAATGTAGAGTTGATTATGAAATTTGTTGTTAATGAGTTTTCGGCTTTATGAATGAA

TTGTAAGACAAGTAATGAAACAAAAACTAGAAGAAAATTGAATTATATGATGCAATCTGTG

TTGAAGTTTTTGCATGTATTCAAGTTCTGCTTTTAGTTACTATTTGTTTATCTGCTGCAAT

ATTTCATCAATTATTATTCATGTATGTACCAATATTTAATAAATTAGGGGCGAATTATGCT

TCCGCTTAGCATGACATCTGACGACGGACCAACATACGTGAATGCTAAGCAATACCATGGA

ATCATCAGACGCAGGCATTCTCGTGCCAAAGCTGTGCTTCAGAATAAATTGATAAAGCGTA

ACAAGGTATGAAACTGAGTATTTTTCCTCACAACAATGTTCGAAAACTTGTGAAATAGTAT

ATTTTTCCTTTTACCCTTTTTATACCTATATTAAGGTTTTGTATTTGTCTTGCAGCCATAT

ATGCACGAATCGCGTCATCTACATGCAATGCGTAGACCAAGAGGATGCGGTGGTCGTTTCT

TGAACACAAAAGTTTCTGCTAATGGAAACGGTAAAAGCGGGAGTGAAGAGAACGGAAACAT

TGGTGGCCTACAGCTGCAGTCCAGTGGTTCTCAGAGTTCCGAAGTCTTACAATCTGAGGTT

GGAACTTTAAATTCGTCGAAGGAGACAAACGGAGGCAGTCCAAATGTCTCGGGGTCAGAGG

TGACTAGCATGTATACACAGGGAGGTCTTGATAGCTTTACTGTCAATCATATCGGATCTAC

TGTCCACTCTTTGGGAGACATGATCGATACTGGACACGGTATCGTCATGCCAACAAAATGG

TTTGCAGCAGCTGGCAGACAGCTGCTGGAACCATAAGTTTCGATTCAGAAAGGAAACAAGT

GGGTTTGGTACAATGTGAAATATTTTGCACCAAACTCATCCTTTCCGAGACCAGATGAAGA

AGCTATGTTTCAGTTTGTTGTGTTTACTACGACAAATTTAGTTTCGGAAGACTACTTTTCC

ATCTGGTGCTCAGGCAACTCATTCTTGGCTTATTCTCAGGAAACTCATCCTTGGCTCGTAA

TATTTAGTAGTATTGTCATTGTCTTCCCGCGCAGGCTTGCCGTGGCATGGTAGGCATGCTA

ATGACTTTGGTATTTTCATGCAGTTATAACTATGATGTGTCTTTGTTTGTTGTTAAAATAA

AAAACATGAACTCTAGCTAGGTGCATGTGTGTGTTTTTAATCTTGTCTACTAAGTTTGGTG

TTTTGTAATGGATTTCTGACTTTATGGAGCAATGTATTGTAACTCTACTAAGAAGTGTAAC

ATTTTATTTCTCCCTCTCTAAGGATTGTATAAGAACCTCTTATTTTCAGACTCTACTTAAT

CCTATTTTCTATGTCTGTATGATTTTTATATTTCTAGGACAATCAAATTGGCTTGTAGAAG

CTCAAAAGCATGCTCACAAAGTAGGTACTTATGTAGGGAACTTCGTACTCTAATAATAACT

GGTTATTAACGTTATAATTAAATGCAAAATTTGATAAGTAGTAGGGTTTGGTAAGTAATAT

AACAACACCATGTTGGCTTGGGATTCTGAATTGTGTTATTAGAGTACAACTCTTAAATAAT

GATCGTGATGTAAATAATTTAAATTTGAAGTTACTAGATAAAAAGATAACATTATGTAATA

TATTATCAAATATATACTAAAAATATAATTTTCATCTATCTCAATTGAAAAAATATCCGGG

TTTTGAGTCTATTGTCAATGACGA

>HM034

AAAAAAATAAAGGTTCTATACTCATTAAACAAAGTGCCAAATTCAGAGATACTACTTCCT

TTGGATAATTTGTATCGTCACCAACGATCTTTGAGTTATTTCAAAAATCACATTCTCAATT

TTGTGGTCCAACATTCACAAAATGGTTTGTCGGGCTTTAAGTCTAGTAATTGTGAAACATG

CATATGCATGAGGTAATTAATTGATAATCTGAAAAGGTTAAATAGTTAATCAATTGTACTT

TGGTTATTTTTAATTTGTTCTCTATAATTTTTCAGTCAAAATTGAATTTGCTTTAACCATT

TAGTGTAGTAGTATGATATTACTTGCTCTACATTCTTGGATCTAAAAGTCTAATTAGTATC

GTATAAAAAATATTCTAGTTAATTAAGATACTTTTGTTCATTCTAATTATTCTTGGATTAC

TTGTGAAACTTTTAGGGATGAACAAGAAAGTCCACTAAAGAGGCTTCTTTTTGCTAGGAGT

CACTAGTTAGTTTTTTTTTTTTTTGACAAAAGTTAACCATTTTTTTTTATCGCATGGTAGG

AAAAAAAAAATAGAGATTTCCTTTGGTACTCATAAAAAAAAAAGTTGGATGGATAATGGAT

TAATCATAACTTATTTAACATAAAATGAATTAAGAGAACATTTGATATTTGGGTTTATTTT

GTAACATAAATATTAATATCAATGTTTGAAAGAGTTTTTAACAATAATTTGTAAAAGAAAG

CATAATATATCTATCAAAATAATTGTAAAAGAAAAAATTTCTTTTGGTGGTATGACATGGA

AAAATAATTAATTCTTATTAGATGAAAGTGTAAAATTAATTTGCATTAACAAAGCATATTT

CATTAAACTCCTTTTAGTTTTTGTTGGAAAAAATAAATTTATTGTTACTCAAAAAATAAAA

GTGTACAATTAATATATTTACTCAAAAGTCCCCTAAATGGGATGTAATTATCAAGGGATGT

GAGCGGTTTAACTTTAAGTAATGAATCAACTGTTGCATAATCACTTTGAAAGAAACTTGAG

ATTCTTCAATTAGCAAAATCTTGAATAGAATAATGAGATTATTCCTAATGCTAATGATACC

CACCTCTCCTATAAGTTGATGGGGCAGAACAAATTATGCACTCAGGACAACTTTTCTCAAA

TTTTGAAAACTTACCACACGAAGAACACACTTCCACTTGCACAAGTAAGAATATAGTATAA

CAATAAATTTACCACTCATAGCTCAATGGATATTAAAATATTGCTAATCTTTTATAGACAG

ATGTTGGCAAATTAGTATAAATTAGTTATTATATTAGTTTTTAAGGTTCAATCTACTTTTT

AAAACTCTTATTAAGTTGTTTAGCTTGCCAACCAAAGACTAAATTATCTTTATTTGTTAGG

AAATAAAAGCACTTATTTTATTTTCTCCATATTTGTTTCCTCTTTGTTCCATTACTAAAAA

AATTATAATATATTAATAACAAAAAAATTTGGGGAGGGAATGTTATTTTTAAAAAAGATGT

TTTATTTATATTTAAGTGTTTTATTGAAATTCCTCGTGAGTTTAGCTCAATTGGTATGGAC

AATACATAATATATGCAAAATTCGAGGTTCAAACCTTGAGCACCAAAAAAAAAAGTGTTTT

ATTGAAAAATATCAAGAACTTGAAAACAAATTTCCTTTTTATGAACTAGATATTGTCACAT

AGACAACTTAAGAATTGACATTTTTTTTAACACTATTGTATTTTCTGAACTCGACTTTAAC

TCGAGATCAATGAGTAAACTAAAAAAACTCGTATTATTTTATCTAAGTGTTCTTAGTGTGA

GAAAACATTATAATTGTTTGCTTAGTCTATTTCTCCCTATATTCTCACCCTCATTCTTGGT

TTGCACATAGAAAGGAGAAAAAAAAAATTAGAGAAGAGTTGAATGGAACAGAAAGAAAGAG

CAAGCAAAATAATCACTAAAAAAAATTCCCCTAAAAGAAAATCACTGAAAAATATTATCCA

CCTCTATTTTAAATAATAATAATAATGAAAACAACAATATATTTTTTTATAGATTTTCTCT

ACTAGAGGTAATTTATTTTAAACACTAAATGTCCACACCTCTAACAAAAAAAATGCATAAT

ATTATAATAGATTATTGTAAGAAAGAGTGACAAATGTTGAATTAGTTATGGAATGAAGAGT

GAGAGAGATTATACAAATAAATAAATATAAATATAAATATAAATAAAGAATAGAGAAGAAC

ATAGTACTTGAATGAAGAGATTTGGCTTGTACTTCCATTACACTTGTAAGCTTTCTACTTC

CTTCACATTCCTTCTCTTTCTCTATCTTTCCTTTTATGATATGTGTACTTTGTTATTGCTT

CATTTACTACCGACATGACTCCAACCTATTAGGGCTTTATCTGAATAATGTTAGTTATTTT

CTCTCATTTTCTTTGCTTCTTCTTATTCTTGGAACTTATAAAGGGTCTTGTCAATTCTATC

ATTCTCTTTTGTATACTTACAAATATAAATATTCTCTTTAGCTTCATTTTCATGTCTTCTT

CTACTAATAAGCTATGGAAATTGAAGTTTTTGTTTTCATGTTTATTTTTCACTTCAGCTCC

TCTTTTTTGATGTTGATGCTGAAGATTGAATAAAAACTGAGGTGGTATGTATGAGAACAAG

AACATTTTTCATTCATCTCTGTTATTTGAACATCTAAATTTTAAACAACTATCGCGACAAT

TTTTTAAGTGGTTGAAGTATTTTAAATAGGTGGTTAATTACATTAAGTAGATAGTTAATTG

ATATATCAGGTGTCAAAACTCATTAACCATCAACATAACTTCATTAGCCATAAATTTTTGA

CGTGATTAACCAAATATTGTAGATGCTCAATTACATGTAGATTTGATGGAGTTGTCCACAA

TTTCTCATATATCTATTAACTAATAACCATCTACCGAATATAATTAACCATATATTAGAAT

ATCATTAACCTCTAAAACAGTTGTCGCGAAAATTGGTTGTAAAAAAATCATTTCTATTTTT

TACGCCACTAGTTCTCTTGTTATTTCTCTTTGTTGGAAAGTTGTTGTTTTTAACTTTGAGC

TTAGTCTTATTAATTAACTTGTAATGGTAACAACATTTTTCTATCTTTGTTTGAAGGAAGT

AGTTACTGTGTCAACTTTAGTAATTAATTTGGATCATTTTGGAGGCAAAATATGGCTATGC

AACCTGTTTATTTTAAAGAACATGAAGGAAATGTCCACAATTCTGTTGGACAGTTTTCATC

TGTGACTTCAGCACCATGGTGGAGTAATGCCTATGGATCTCAACCTGTTTATGGAGGAGAC

TCTTGTGGCCAAATGAAACCTTTTTCACTAGAGCTTTCCAACTACATAGACCAACTTGCTC

CGAGTAAGAACTTAGTTCGAGGAGTTGAACAATTGTTTGATAAAGGGCATACAAACCAATT

CACCATCTTTCCAGGTACTTGTTATTCAATATAATTCCGGTTTTGAATGAATTGATTTTTG

TTAAGTTGGTTCTGGATAAACGTGCGTTAAATATAATATGATTTATGTTTGGATACGTTTA

TGTTAAAGTGAGTTGAACATAAATTGGAGACTAAATATCAATTGCAGAGGCAAAAGCTTCA

AATTCTAATTTCAAGTTAGAATCAAATCAATTCTACTCGTAAGCATCTAAATATTTCAAAA

CCAATTCTACATCTCTAGAACTAAATTTGCATTCAAACATTTTCTGCTACAGATGATTGTA

AGATGTCAGTTGATACACCAAATCATCAAGCAACCTTATCCCTGCAATCACCATTTGCTGC

CGAGCCACTTAATCGATTTGAGCTAGGTTTTAACCAGTCTATGGTAAATCTTCTTATTAAA

GCAATCCCTTTGATCATGTTTTAGTATTTTACTTTTGATGTACATAAATTCATTATGTTTT

TATTATCCTTTCCATGCTAGATCTGTGCAAAATATCCGTATATAGATCAATTTTACGGGCT

CTTCTCGACTTATGGACCTCAAATCTCGGTTTGTCTTCACCTCGGCTATTTTATTAATCTT

TGTATATATCATGCAATGTCTATAAGTACTTGAATGTGAATTTGTTTTATTTACAGTGTGT

ATGCTTACATGAGAAAGCCAGAAATCTGTTTACAACTATCTTGTTAGTATTCTTCTAAATC

TTTCAAATGTAGAGTTGATTATGAAATTTGTTGTTAATGAGTTTTCGGCTTTATGAATGAA

TTGTAAGACAAGTAATGAAACAAAAACTAGAAGAAAATTGAATTATATGATGCAATCTGTG

TTGAAGTTTTTGCATGTATTCAAGTTCTGCTTTTAGTTACTATTTGTTTATCTGCTGCAAT

ATTTCATCAATTATTATTCATGTATGTACCAATATTTAATAAATTAGGGGCGAATTATGCT

TCCGCTTAGCATGACATCTGACGACGGACCAACATACGTGAATGCTAAGCAATACCATGGA

ATCATCAGACGCAGGCATTCTCGTGCCAAAGCTGTGCTTCAGAATAAATTGATAAAGCGTA

ACAAGGTATGAAACTGAGTATTTTTCCTCACAACAATGTTCGAAAACTTGTGAAATAGTAT

ATTTTTCCTTTTACCCTTTTTATACCTATATTAAGGTTTTGTATTTGTCTTGCAGCCATAT

ATGCACGAATCGCGTCATCTACATGCAATGCGTAGACCAAGAGGATGCGGTGGTCGTTTCT

TGAACACAAAAGTTTCTGCTAATGGAAACGGTAAAAGCGGGAGTGAAGAGAACGGAAACAT

TGGTGGCCTACAGCTGCAGTCCAGTGGTTCTCAGAGTTCCGAAGTCTTACAATCTGAGGTT

GGAACTTTAAATTCGTCGAAGGAGACAAACGGAGGCAGTCCAAATGTCTCGGGGTCAGAGG

TGACTAGCATGTATACACAGGGAGGTCTTGATAGCTTTACTGTCAATCATATCGGATCTAC

TGTCCACTCTTTGGGAGACATGATCGATACTGGACACGGTATCGTCATGCCAACAAAATGG

TTTGCAGCAGCTGGCAGACAGCTGCTGGAACCATAAGTTTCGATTCAGAAAGGAAACAAGT

GGGTTTGGTACAATGTGAAATATTTTGCACCAAACTCATCCTTTCCGAGACCAGATGAAGA

AGCTATGTTTCAGTTTGTTGTGTTTACTACGACAAATTTAGTTTCGGAAGACTACTTTTCC

ATCTGGTGCTCAGGCAACTCATTCTTGGCTTATTCTCAGGAAACTCATCCTTGGCTCGTAA

TATTTAGTAGTATTGTCATTGTCTTCCCGCGCAGGCTTGCCGTGGCATGGTAGGCATGCTA

ATGACTTTGGTATTTTCATGCAGTTATAACTATGATGTGTCTTTGTTTGTTGTTAAAATAA

AAAACATGAACTCTAGCTAGGTGCATGTGTGTGTTTTTAATCTTGTCTACTAAGTTTGGTG

TTTTGTAATGGATTTCTGACTTTATGGAGCAATGTATTGTAACTCTACTAAGAAGTGTAAC

ATTTTATTTCTCCCTCTCTAAGGATTGTATAAGAACCTCTTATTTTCAGACTCTACTTAAT

CCTATTTTCTATGTCTGTATGATTTTTATATTTCTAGGACAATCAAATTGGCTTGTAGAAG

CTCAAAAGCATGCTCACAAAGTAGGTACTTATGTAGGGAACTTCGTACTCTAATAATAACT

GGTTATTAACGTTATAATTAAATGCAAAATTTGATAAGTAGTAGGGTTTGGTAAGTAATAT

AACAACACCATGTTGGCTTGGGATTCTGAATTGTGTTATTAGAGTACAACTCTTAAATAAT

GATCGTGATGTAAATAATTTAAATTTGAAGTTACTAGATAAAAAGATAACATTATGTAATA

TATTATCAAATATATACTAAAAATATAATTTTCATCTATCTCAATTGAAAAAATATCCGGG

TTTTGAGTCTATTGTCAATGACGA

>HM035

AAAAAAATAAAGGTTCTATACTCATTAAACAAAGTGCCAAATTCAGAGATACTACTTCCT

TTGGATAATTTGTATCGTCACCAACGATCTTTGAGTTATTTCAAAAATCACATTCTCAATT

TTGTGGTCCAACATTCACAAAATGGTTTGTCGGGCTTTAAGTCTAGTAATTGTGAAACATG

CATATGCATGAGGTAATTAATTGATAATCTGAAAAGGTTAAATAGTTAATCAATTGTACTT

TGGTTATTTTTAATTTGTTCTCTATAATTTTTCAGTCAAAATTGAATTTGCTTTAACCATT

TAGTGTAGTAGTATGATATTACTTGCTCTACATTCTTGGATCTAAAAGTCTAATTAGTATC

GTATAAAAAATATTCTAGTTAATTAAGATACTTTTGTTCATTCTAATTATTCTTGGATTAC

TTGTGAAACTTTTAGGGATGAACAAGAAAGTCCACTAAAGAGGCTTCTTTTTGCTAGGAGT

CACTAGTTAGTTTTTTTTTTTTTTGACAAAAGTTAACCATTTTTTTTTATCGCATGGTAGG

AAAAAAAAAATAGAGATTTCCTTTGGTACTCATAAAAAAAAAAGTTGGATGGATAATGGAT

TAATCATAACTTATTTAACATAAAATGAATTAAGAGAACATTTGATATTTGGGTTTATTTT

GTAACATAAATATTAATATCAATGTTTGAAAGAGTTTTTAACAATAATTTGTAAAAGAAAG

CATAATATATCTATCAAAATAATTGTAAAAGAAAAAATTTCTTTTGGTGGTATGACATGGA

AAAATAATTAATTCTTATTAGATGAAAGTGTAAAATTAATTTGCATTAACAAAGCATATTT

CATTAAACTCCTTTTAGTTTTTGTTGGAAAAAATAAATTTATTGTTACTCAAAAAATAAAA

GTGTACAATTAATATATTTACTCAAAAGTCCCCTAAATGGGATGTAATTATCAAGGGATGT

GAGCGGTTTAACTTTAAGTAATGAATCAACTGTTGCATAATCACTTTGAAAGAAACTTGAG

ATTCTTCAATTAGCAAAATCTTGAATAGAATAATGAGATTATTCCTAATGCTAATGATACC

CACCTCTCCTATAAGTTGATGGGGCAGAACAAATTATGCACTCAGGACAACTTTTCTCAAA

TTTTGAAAACTTACCACACGAAGAACACACTTCCACTTGCACAAGTAAGAATATAGTATAA

CAATAAATTTACCACTCATAGCTCAATGGATATTAAAATATTGCTAATCTTTTATAGACAG

ATGTTGGCAAATTAGTATAAATTAGTTATTATATTAGTTTTTAAGGTTCAATCTACTTTTT

AAAACTCTTATTAAGTTGTTTAGCTTGCCAACCAAAGACTAAATTATCTTTATTTGTTAGG

AAATAAAAGCACTTATTTTATTTTCTCCATATTTGTTTCCTCTTTGTTCCATTACTAAAAA

AATTATAATATATTAATAACAAAAAAATTTGGGGAGGGAATGTTATTTTTAAAAAAGATGT

TTTATTTATATTTAAGTGTTTTATTGAAATTCCTCGTGAGTTTAGCTCAATTGGTATGGAC

AATACATAATATATGCAAAATTCGAGGTTCAAACCTTGAGCACCAAAAAAAAAAGTGTTTT

ATTGAAAAATATCAAGAACTTGAAAACAAATTTCCTTTTTATGAACTAGATATTGTCACAT

AGACAACTTAAGAATTGACATTTTTTTTAACACTATTGTATTTTCTGAACTCGACTTTAAC

TCGAGATCAATGAGTAAACTAAAAAAACTCGTATTATTTTATCTAAGTGTTCTTAGTGTGA

GAAAACATTATAATTGTTTGCTTAGTCTATTTCTCCCTATATTCTCACCCTCATTCTTGGT

TTGCACATAGAAAGGAGAAAAAAAAAATTAGAGAAGAGTTGAATGGAACAGAAAGAAAGAG

CAAGCAAAATAATCACTAAAAAAAATTCCCCTAAAAGAAAATCACTGAAAAATATTATCCA

CCTCTATTTTAAATAATAATAATAATGAAAACAACAATATATTTTTTTATAGATTTTCTCT

ACTAGAGGTAATTTATTTTAAACACTAAATGTCCACACCTCTAACAAAAAAAATGCATAAT

ATTATAATAGATTATTGTAAGAAAGAGTGACAAATGTTGAATTAGTTATGGAATGAAGAGT

GAGAGAGATTATACAAATAAATAAATATAAATATAAATATAAATAAAGAATAGAGAAGAAC

ATAGTACTTGAATGAAGAGATTTGGCTTGTACTTCCATTACACTTGTAAGCTTTCTACTTC

CTTCACATTCCTTCTCTTTCTCTATCTTTCCTTTTATGATATGTGTACTTTGTTATTGCTT

CATTTACTACCGACATGACTCCAACCTATTAGGGCTTTATCTGAATAATGTTAGTTATTTT

CTCTCATTTTCTTTGCTTCTTCTTATTCTTGGAACTTATAAAGGGTCTTGTCAATTCTATC

ATTCTCTTTTGTATACTTACAAATATAAATATTCTCTTTAGCTTCATTTTCATGTCTTCTT

CTACTAATAAGCTATGGAAATTGAAGTTTTTGTTTTCATGTTTATTTTTCACTTCAGCTCC

TCTTTTTTGATGTTGATGCTGAAGATTGAATAAAAACTGAGGTGGTATGTATGAGAACAAG

AACATTTTTCATTCATCTCTGTTATTTGAACATCTAAATTTTAAACAACTATCGCGACAAT

TTTTTAAGTGGTTGAAGTATTTTAAATAGGTGGTTAATTACATTAAGTAGATAGTTAATTG

ATATATCAGGTGTCAAAACTCATTAACCATCAACATAACTTCATTAGCCATAAATTTTTGA

CGTGATTAACCAAATATTGTAGATGCTCAATTACATGTAGATTTGATGGAGTTGTCCACAA

TTTCTCATATATCTATTAACTAATAACCATCTACCGAATATAATTAACCATATATTAGAAT

ATCATTAACCTCTAAAACAGTTGTCGCGAAAGTTGGTTGTAAAAAAATCATTTCTATTTTT

TACGCCACTAGTTCTCTTGTTATTTCTCTTTGTTGGAAAGTTGTTGTTTTTAACTTTGAGC

TTAGTCTTATTAATTAACTTGTAATGGTAACAACATTTTTCTATCTTTGTTTGAAGGAAGT

AGTTACTGTGTCAACTTTAGTAATTAATTTGGATCATTTTGGAGGCAAAATATGGCTATGC

AACCTGTTTATTTTAAAGAACATGAAGGAAATGTCCACAATTCTGTTGGACAGTTTTCATC

TGTGACTTCAGCACCATGGTGGAGTAATGCCTATGGATCTCAACCTGTTTATGGAGGAGAC

TCTTGTGGCCAAATGAAACCTTTTTCACTAGAGCTTTCCAACTACATAGACCAACTTGCTC

CGAGTAAGAACTTAGTTCGAGGAGTTGAACAATTGTTTGATAAAGGGCATACAAACCAATT

CACCATCTTTCCAGGTACTTGTTATTCAATATAATTCCGGTTTTGAATGAATTGATTTTTG

TTAAGTTGGTTCTGGATAAACGTGCGTTAAATATAATATGATTTATGTTTGGATACGTTTA

TGTTAAAGTGAGTTGAACATAAATTGGAGACTAAATATCAATTGCAGAGGCAAAAGCTTCA

AATTCTAATTTCAAGTTAGAATCAAATCAATTCTACTCGTAAGCATCTAAATATTTCAAAA

CCAATTCTACATCTCTAGAACTAAATTTGCATTCAAACATTTTCTGCTACAGATGATTGTA

AGATGTCAGTTGATACACCAAATCATCAAGCAACCTTATCCCTGCAATCACCATTTGCTGC

CGAGCCACTTAATCGATTTGAGCTAGGTTTTAACCAGTCTATGGTAAATCTTCTTATTAAA

GCAATCCCTTTGATCATGTTTTAGTATTTTACTTTTGATGTACATAAATTCATTATGTTTT

TATTATCCTTTCCATGCTAGATCTGTGCAAAATATCCGTATATAGATCAATTTTACGGGCT

CTTCTCGACTTATGGACCTCAAATCTCGGTTTGTCTTCACCTCGGCTATTTTATTAATCTT

TGTATATATCATGCAATGTCTATAAGTACTTGAATGTGAATTTGTTTTATTTACAGTGTGT

ATGCTTACATGAGAAAGCCAGAAATCTGTTTACAACTATCTTGTTAGTATTCTTCTAAATC

TTTCAAATGTAGAGTTGATTATGAAATTTGTTGTTAATGAGTTTTCGGCTTTATGAATGAA

TTGTAAGACAAGTAATGAAACAAAAACTAGAAGAAAATTGAATTATATGATGCAATCTGTG

TTGAAGTTTTTGCATGTATTCAAGTTCTGCTTTTAGTTACTATTTGTTTATCTGCTGCAAT

ATTTCATCAATTATTATTCATGTATGTACCAATATTTAATAAATTAGGGGCGAATTATGCT

TCCGCTTAGCATGACATCTGACGACGGACCAACATACGTGAATGCTAAGCAATACCATGGA

ATCATCAGACGCAGGCATTCTCGTGCCAAAGCTGTGCTTCAGAATAAATTGATAAAGCGTA

ACAAGGTATGAAACTGAGTATTTTTCCTCACAACAATGTTCGAAAACTTGTGAAATAGTAT

ATTTTTCCTTTTACCCTTTTTATACCTATATTAAGGTTTTGTATTTGTCTTGCAGCCATAT

ATGCACGAATCGCGTCATCTACATGCAATGCGTAGACCAAGAGGATGCGGTGGTCGTTTCT

TGAACACAAAAGTTTCTGCTAATGGAAACGGTAAAAGCGGGAGTGAAGAGAACGGAAACAT

TGGTGGCCTACAGCTGCAGTCCAGTGGTTCTCAGAGTTCCGAAGTCTTACAATCTGAGGTT

GGAACTTTAAATTCGTCGAAGGAGACAAACGGAGGCAGTCCAAATGTCTCGGGGTCAGAGG

TGACTAGCATGTATACACAGGGAGGTCTTGATAGCTTTACTGTCAATCATATCGGATCTAC

TGTCCACTCTTTGGGAGACATGATCGATACTGGACACGGTATCGTCATGCCAACAAAATGG

TTTGCAGCAGCTGGCAGACAGCTGCTGGAACCATAAGTTTCGATTCAGAAAGGAAACAAGT

GGGTTTGGTACAATGTGAAATATTTTGCACCAAACTCATCCTTTCCGAGACCAGATGAAGA

AGCTATGTTTCAGTTTGTTGTGTTTACTACGACAAATTTAGTTTCGGAAGACTACTTTTCC

ATCTGGTGCTCAGGCAACTCATTCTTGGCTTATTCTCAGGAAACTCATCCTTGGCTCGTAA

TATTTAGTAGTATTGTCATTGTCTTCCCGCGCAGGCTTGCCGTGGCATGGTAGGCATGCTA

ATGACTTTGGTATTTTCATGCAGTTATAACTATGATGTGTCTTTGTTTGTTGTTAAAATAA

AAAACATGAACTCTAGCTAGGTGCATGTGTGTGTTTTTAATCTTGTCTACTAAGTTTGGTG

TTTTGTAATGGATTTCTGACTTTATGGAGCAATGTATTGTAACTCTACTAAGAAGTGTAAC

ATTTTATTTCTCCCTCTCTAAGGATTGTATAAGAACCTCTTATTTTCAGACTCTACTTAAT

CCTATTTTCTATGTCTGTATGATTTTTATATTTCTAGGACAATCAAATTGGCTTGTAGAAG

CTCAAAAGCATGCTCACAAAGTAGGTACTTATGTAGGGAACTTCGTACTCTAATAATAACT

GGTTATTAACGTTATAATTAAATGCAAAATTTGATAAGTAGTAGGGTTTGGTAAGTAATAT

AACAACACCATGTTGGCTTGGGATTCTGAATTGTGTTATTAGAGTACAACTCTTAAATAAT

GATCGTGATGTAAATAATTTAAATTTGAAGTTACTAGATAAAAAGATAACATTATGTAATA

TATTATCAAATATATACTAAAAATATAATTTTCATCTATCTCAATTGAAAAAATATCCGGG

TTTTGAGTCTATTGTCAATGACGA

>HM036

AAAAAAATAAAGGTTCTATACTCATTAAACAAAGTGCCAAATTCAGAGATACTACTTCCT

TTGGATAATTTGTATCGTCACCAACGATCTTTGAGTTATTTCAAAAATCACATTCTCAATT

TTGTGGTCCAACATTCACAAAATGGTTTGTCGGGCTTTAAGTCTAGTAATTGTGAAACATG

CATATGCATGAGGTAATTAATTGATAATCTGAAAAGGTTAAATAGTTAATCAATTGTACTT

TGGTTATTTTTAATTTGTTCTCTATAATTTTTCAGTCAAAATTGAATTTGCTTTAACCATT

TAGTGTAGTAGTATGATATTACTTGCTCTACATTCTTGGATCTAAAAGTCTAATTAGTATC

GTATAAAAAATATTCTAGTTAATTAAGATACTTTTGTTCATTCTAATTATTCTTGGATTAC

TTGTGAAACTTTTAGGGATGAACAAGAAAGTCCACTAAAGAGGCTTCTTTTTGCTAGGAGT

CACTAGTTAGTTTTTTTTTTTTTTGACAAAAGTTAACCATTTTTTTTTATCGCATGGTAGG

AAAAAAAAAATAGAGATTTCCTTTGGTACTCATAAAAAAAAAAGTTGGATGGATAATGGAT

TAATCATAACTTATTTAACATAAAATGAATTAAGAGAACATTTGATATTTGGGTTTATTTT

GTAACATAAATATTAATATCAATGTTTGAAAGAGTTTTTAACAATAATTTGTAAAAGAAAG

CATAATATATCTATCAAAATAATTGTAAAAGAAAAAATTTCTTTTGGTGGTATGACATGGA

AAAATAATTAATTCTTATTAGATGAAAGTGTAAAATTAATTTGCATTAACAAAGCATATTT

CATTAAACTCCTTTTAGTTTTTGTTGGAAAAAATAAATTTATTGTTACTCAAAAAATAAAA

GTGTACAATTAATATATTTACTCAAAAGTCCCCTAAATGGGATGTAATTATCAAGGGATGT

GAGCGGTTTAACTTTAAGTAATGAATCAACTGTTGCATAATCACTTTGAAAGAAACTTGAG

ATTCTTCAATTAGCAAAATCTTGAATAGAATAATGAGATTATTCCTAATGCTAATGATACC

CACCTCTCCTATAAGTTGATGGGGCAGAACAAATTATGCACTCAGGACAACTTTTCTCAAA

TTTTGAAAACTTACCACACGAAGAACACACTTCCACTTGCACAAGTAAGAATATAGTATAA

CAATAAATTTACCACTCATAGCTCAATGGATATTAAAATATTGCTAATCTTTTATAGACAG

ATGTTGGCAAATTAGTATAAATTAGTTATTATATTAGTTTTTAAGGTTCAATCTACTTTTT

AAAACTCTTATTAAGTTGTTTAGCTTGCCAACCAAAGACTAAATTATCTTTATTTGTTAGG

AAATAAAAGCACTTATTTTATTTTCTCCATATTTGTTTCCTCTTTGTTCCATTACTAAAAA

AATTATAATATATTAATAACAAAAAAATTTGGGGAGGGAATGTTATTTTTAAAAAAGATGT

TTTATTTATATTTAAGTGTTTTATTGAAATTCCTCGTGAGTTTAGCTCAATTGGTATGGAC

AATACATAATATATGCAAAATTCGAGGTTCAAACCTTGAGCACCAAAAAAAAAAGTGTTTT

ATTGAAAAATATCAAGAACTTGAAAACAAATTTCCTTTTTATGAACTAGATATTGTCACAT

AGACAACTTAAGAATTGACATTTTTTTTAACACTATTGTATTTTCTGAACTCGACTTTAAC

TCGAGATCAATGAGTAAACTAAAAAAACTCGTATTATTTTATCTAAGTGTTCTTAGTGTGA

GAAAACATTATAATTGTTTGCTTAGTCTATTTCTCCCTATATTCTCACCCTCATTCTTGGT

TTGCACATAGAAAGGAGAAAAAAAAAATTAGAGAAGAGTTGAATGGAACAGAAAGAAAGAG

CAAGCAAAATAATCACTAAAAAAAATTCCCCTAAAAGAAAATCACTGAAAAATATTATCCA

CCTCTATTTTAAATAATAATAATAATGAAAACAACAATATATTTTTTTATAGATTTTCTCT

ACTAGAGGTAATTTATTTTAAACACTAAATGTCCACACCTCTAACAAAAAAAATGCATAAT

ATTATAATAGATTATTGTAAGAAAGAGTGACAAATGTTGAATTAGTTATGGAATGAAGAGT

GAGAGAGATTATACAAATAAATAAATATAAATATAAATATAAATAAAGAATAGAGAAGAAC

ATAGTACTTGAATGAAGAGATTTGGCTTGTACTTCCATTACACTTGTAAGCTTTCTACTTC

CTTCACATTCCTTCTCTTTCTCTATCTTTCCTTTTATGATATGTGTACTTTGTTATTGCTT

CATTTACTACCGACATGACTCCAACCTATTAGGGCTTTATCTGAATAATGTTAGTTATTTT

CTCTCATTTTCTTTGCTTCTTCTTATTCTTGGAACTTATAAAGGGTCTTGTCAATTCTATC

ATTCTCTTTTGTATACTTACAAATATAAATATTCTCTTTAGCTTCATTTTCATGTCTTCTT

CTACTAATAAGCTATGGAAATTGAAGTTTTTGTTTTCATGTTTATTTTTCACTTCAGCTCC

TCTTTTTTGATGTTGATGCTGAAGATTGAATAAAAACTGAGGTGGTATGTATGAGAACAAG

AACATTTTTCATTCATCTCTGTTATTTGAACATCTAAATTTTAAACAACTATCGCGACAAT

TTTTTAAGTGGTTGAAGTATTTTAAATAGGTGGTTAATTACATTAAGTAGATAGTTAATTG

ATATATCAGGTGTCAAAACTCATTAACCATCAACATAACTTCATTAGCCATAAATTTTTGA

CGTGATTAACCAAATATTGTAGATGCTCAATTACATGTAGATTTGATGGAGTTGTCCACAA

TTTCTCATATATCTATTAACTAATAACCATCTACCGAATATAATTAACCATATATTAGAAT

ATCATTAACCTCTAAAACAGTTGTCGCGAAAGTTGGTTGTAAAAAAATCATTTCTATTTTT

TACGCCACTAGTTCTCTTGTTATTTCTCTTTGTTGGAAAGTTGTTGTTTTTAACTTTGAGC

TTAGTCTTATTAATTAACTTGTAATGGTAACAACATTTTTCTATCTTTGTTTGAAGGAAGT

AGTTACTGTGTCAACTTTAGTAATTAATTTGGATCATTTTGGAGGCAAAATATGGCTATGC

AACCTGTTTATTTTAAAGAACATGAAGGAAATGTCCACAATTCTGTTGGACAGTTTTCATC

TGTGACTTCAGCACCATGGTGGAGTAATGCCTATGGATCTCAACCTGTTTATGGAGGAGAC

TCTTGTGGCCAAATGAAACCTTTTTCACTAGAGCTTTCCAACTACATAGACCAACTTGCTC

CGAGTAAGAACTTAGTTCGAGGAGTTGAACAATTGTTTGATAAAGGGCATACAAACCAATT

CACCATCTTTCCAGGTACTTGTTATTCAATATAATTCCGGTTTTGAATGAATTGATTTTTG

TTAAGTTGGTTCTGGATAAACGTGCGTTAAATATAATATGATTTATGTTTGGATACGTTTA

TGTTAAAGTGAGTTGAACATAAATTGGAGACTAAATATCAATTGCAGAGGCAAAAGCTTCA

AATTCTAATTTCAAGTTAGAATCAAATCAATTCTACTCGTAAGCATCTAAATATTTCAAAA

CCAATTCTACATCTCTAGAACTAAATTTGCATTCAAACATTTTCTGCTACAGATGATTGTA

AGATGTCAGTTGATACACCAAATCATCAAGCAACCTTATCCCTGCAATCACCATTTGCTGC

CGAGCCACTTAATCGATTTGAGCTAGGTTTTAACCAGTCTATGGTAAATCTTCTTATTAAA

GCAATCCCTTTGATCATGTTTTAGTATTTTACTTTTGATGTACATAAATTCATTATGTTTT

TATTATCCTTTCCATGCTAGATCTGTGCAAAATATCCGTATATAGATCAATTTTACGGGCT

CTTCTCGACTTATGGACCTCAAATCTCGGTTTGTCTTCACCTCGGCTATTTTATTAATCTT

TGTATATATCATGCAATGTCTATAAGTACTTGAATGTGAATTTGTTTTATTTACAGTGTGT

ATGCTTACATGAGAAAGCCAGAAATCTGTTTACAACTATCTTGTTAGTATTCTTCTAAATC

TTTCAAATGTAGAGTTGATTATGAAATTTGTTGTTAATGAGTTTTCGGCTTTATGAATGAA

TTGTAAGACAAGTAATGAAACAAAAACTAGAAGAAAATTGAATTATATGATGCAATCTGTG

TTGAAGTTTTTGCATGTATTCAAGTTCTGCTTTTAGTTACTATTTGTTTATCTGCTGCAAT

ATTTCATCAATTATTATTCATGTATGTACCAATATTTAATAAATTAGGGGCGAATTATGCT

TCCGCTTAGCATGACATCTGACGACGGACCAACATACGTGAATGCTAAGCAATACCATGGA

ATCATCAGACGCAGGCATTCTCGTGCCAAAGCTGTGCTTCAGAATAAATTGATAAAGCGTA

ACAAGGTATGAAACTGAGTATTTTTCCTCACAACAATGTTCGAAAACTTGTGAAATAGTAT

ATTTTTCCTTTTACCCTTTTTATACCTATATTAAGGTTTTGTATTTGTCTTGCAGCCATAT

ATGCACGAATCGCGTCATCTACATGCAATGCGTAGACCAAGAGGATGCGGTGGTCGTTTCT

TGAACACAAAAGTTTCTGCTAATGGAAACGGTAAAAGCGGGAGTGAAGAGAACGGAAACAT

TGGTGGCCTACAGCTGCAGTCCAGTGGTTCTCAGAGTTCCGAAGTCTTACAATCTGAGGTT

GGAACTTTAAATTCGTCGAAGGAGACAAACGGAGGCAGTCCAAATGTCTCGGGGTCAGAGG

TGACTAGCATGTATACACAGGGAGGTCTTGATAGCTTTACTGTCAATCATATCGGATCTAC

TGTCCACTCTTTGGGAGACATGATCGATACTGGACACGGTATCGTCATGCCAACAAAATGG

TTTGCAGCAGCTGGCAGACAGCTGCTGGAACCATAAGTTTCGATTCAGAAAGGAAACAAGT

GGGTTTGGTACAATGTGAAATATTTTGCACCAAACTCATCCTTTCCGAGACCAGATGAAGA

AGCTATGTTTCAGTTTGTTGTGTTTACTACGACAAATTTAGTTTCGGAAGACTACTTTTCC

ATCTGGTGCTCAGGCAACTCATTCTTGGCTTATTCTCAGGAAACTCATCCTTGGCTCGTAA

TATTTAGTAGTATTGTCATTGTCTTCCCGCGCAGGCTTGCCGTGGCATGGTAGGCATGCTA

ATGACTTTGGTATTTTCATGCAGTTATAACTATGATGTGTCTTTGTTTGTTGTTAAAATAA

AAAACATGAACTCTAGCTAGGTGCATGTGTGTGTTTTTAATCTTGTCTACTAAGTTTGGTG

TTTTGTAATGGATTTCTGACTTTATGGAGCAATGTATTGTAACTCTACTAAGAAGTGTAAC

ATTTTATTTCTCCCTCTCTAAGGATTGTATAAGAACCTCTTATTTTCAGACTCTACTTAAT

CCTATTTTCTATGTCTGTATGATTTTTATATTTCTAGGACAATCAAATTGGCTTGTAGAAG

CTCAAAAGCATGCTCACAAAGTAGGTACTTATGTAGGGAACTTCGTACTCTAATAATAACT

GGTTATTAACGTTATAATTAAATGCAAAATTTGATAAGTAGTAGGGTTTGGTAAGTAATAT

AACAACACCATGTTGGCTTGGGATTCTGAATTGTGTTATTAGAGTACAACTCTTAAATAAT

GATCGTGATGTAAATAATTTAAATTTGAAGTTACTAGATAAAAAGATAACATTATGTAATA

TATTATCAAATATATACTAAAAATATAATTTTCATCTATCTCAATTGAAAAAATATCCGGG

TTTTGAGTCTATTGTCAATGACGA

>HM037

AAAAAAATAAAGGTTCTATACTCATTAAACAAAGTGCCAAATTCAGAGATACTACTTCCT

TTGGATAATTTGTATCGTCACCAACGATCTTTGAGTTATTTCAAAAATCACATTCTCAATT

TTGTGGTCCAACATTCACAAAATGGTTTGTCGGGCTTTAAGTCTAGTAATTGTGAAACATG

CATATGCATGAGGTAATTAATTGATAATCTGAAAAGGTTAAATAGTTAATCAATTGTACTT

TGGTTATTTTTAATTTGTTCTCTATAATTTTTCAGTCAAAATTGAATTTGCTTTAACCATT

TAGTGTAGTAGTATGATATTACTTGCTCTACATTCTTGGATCTAAAAGTCTAATTAGTATC

GTATAAAAAATATTCTAGTTAATTAAGATACTTTTGTTCATTCTAATTATTCTTGGATTAC

TTGTGAAACTTTTAGGGATGAACAAGAAAGTCCACTAAAGAGGCTTCTTTTTGCTAGGAGT

CACTAGTTAGTTTTTTTTTTTTTTGACAAAAGTTAACCATTTTTTTTTATCGCATGGTAGG

AAAAAAAAAATAGAGATTTCCTTTGGTACTCATAAAAAAAAAAGTTGGATGGATAATGGAT

TAATCATAACTTATTTAACATAAAATGAATTAAGAGAACATTTGATATTTGGGTTTATTTT

GTAACATAAATATTAATATCAATGTTTGAAAGAGTTTTTAACAATAATTTGTAAAAGAAAG

CATAATATATCTATCAAAATAATTGTAAAAGAAAAAATTTCTTTTGGTGGTATGACATGGA

AAAATAATTAATTCTTATTAGATGAAAGTGTAAAATTAATTTGCATTAACAAAGCATATTT

CATTAAACTCCTTTTAGTTTTTGTTGGAAAAAATAAATTTATTGTTACTCAAAAAATAAAA

GTGTACAATTAATATATTTACTCAAAAGTCCCCTAAATGGGATGTAATTATCAAGGGATGT

GAGCGGTTTAACTTTAAGTAATGAATCAACTGTTGCATAATCACTTTGAAAGAAACTTGAG

ATTCTTCAATTAGCAAAATCTTGAATAGAATAATGAGATTATTCCTAATGCTAATGATACC

CACCTCTCCTATAAGTTGATGGGGCAGAACAAATTATGCACTCAGGACAACTTTTCTCAAA

TTTTGAAAACTTACCACACGAAGAACACACTTCCACTTGCACAAGTAAGAATATAGTATAA

CAATAAATTTACCACTCATAGCTCAATGGATATTAAAATATTGCTAATCTTTTATAGACAG

ATGTTGGCAAATTAGTATAAATTAGTTATTATATTAGTTTTTAAGGTTCAATCTACTTTTT

AAAACTCTTATTAAGTTGTTTAGCTTGCCAACCAAAGACTAAATTATCTTTATTTGTTAGG

AAATAAAAGCACTTATTTTATTTTCTCCATATTTGTTTCCTCTTTGTTCCATTACTAAAAA

AATTATAATATATTAATAACAAAAAAATTTGGGGAGGGAATGTTATTTTTAAAAAAGATGT

TTTATTTATATTTAAGTGTTTTATTGAAATTCCTCGTGAGTTTAGCTCAATTGGTATGGAC

AATACATAATATATGCAAAATTCGAGGTTCAAACCTTGAGCACCAAAAAAAAAAGTGTTTT

ATTGAAAAATATCAAGAACTTGAAAACAAATTTCCTTTTTATGAACTAGATATTGTCACAT

AGACAACTTAAGAATTGACATTTTTTTTAACACTATTGTATTTTCTGAACTCGACTTTAAC

TCGAGATCAATGAGTAAACTAAAAAAACTCGTATTATTTTATCTAAGTGTTCTTAGTGTGA

GAAAACATTATAATTGTTTGCTTAGTCTATTTCTCCCTATATTCTCACCCTCATTCTTGGT

TTGCACATAGAAAGGAGAAAAAAAAAATTAGAGAAGAGTTGAATGGAACAGAAAGAAAGAG

CAAGCAAAATAATCACTAAAAAAAATTCCCCTAAAAGAAAATCACTGAAAAATATTATCCA

CCTCTATTTTAAATAATAATAATAATGAAAACAACAATATATTTTTTTATAGATTTTCTCT

ACTAGAGGTAATTTATTTTAAACACTAAATGTCCACACCTCTAACAAAAAAAATGCATAAT

ATTATAATAGATTATTGTAAGAAAGAGTGACAAATGTTGAATTAGTTATGGAATGAAGAGT

GAGAGAGATTATACAAATAAATAAATATAAATATAAATATAAATAAAGAATAGAGAAGAAC

ATAGTACTTGAATGAAGAGATTTGGCTTGTACTTCCATTACACTTGTAAGCTTTCTACTTC

CTTCACATTCCTTCTCTTTCTCTATCTTTCCTTTTATGATATGTGTACTTTGTTATTGCTT

CATTTACTACCGACATGACTCCAACCTATTAGGGCTTTATCTGAATAATGTTAGTTATTTT

CTCTCATTTTCTTTGCTTCTTCTTATTCTTGGAACTTATAAAGGGTCTTGTCAATTCTATC

ATTCTCTTTTGTATACTTACAAATATAAATATTCTCTTTAGCTTCATTTTCATGTCTTCTT

CTACTAATAAGCTATGGAAATTGAAGTTTTTGTTTTCATGTTTATTTTTCACTTCAGCTCC

TCTTTTTTGATGTTGATGCTGAAGATTGAATAAAAACTGAGGTGGTATGTATGAGAACAAG

AACATTTTTCATTCATCTCTGTTATTTGAACATCTAAATTTTAAACAACTATCGCGACAAT

TTTTTAAGTGGTTGAAGTATTTTAAATAGGTGGTTAATTACATTAAGTAGATAGTTAATTG

ATATATCAGGTGTCAAAACTCATTAACCATCAACATAACTTCATTAGCCATAAATTTTTGA

CGTGATTAACCAAATATTGTAGATGCTCAATTACATGTAGATTTGATGGAGTTGTCCACAA

TTTCTCATATATCTATTAACTAATAACCATCTACCGAATATAATTAACCATATATTAGAAT

ATCATTAACCTCTAAAACAGTTGTCGCGAAAGTTGGTTGTAAAAAAATCATTTCTATTTTT

TACGCCACTAGTTCTCTTGTTATTTCTCTTTGTTGGAAAGTTGTTGTTTTTAACTTTGAGC

TTAGTCTTATTAATTAACTTGTAATGGTAACAACATTTTTCTATCTTTGTTTGAAGGAAGT

AGTTACTGTGTCAACTTTAGTAATTAATTTGGATCATTTTGGAGGCAAAATATGGCTATGC

AACCTGTTTATTTTAAAGAACATGAAGGAAATGTCCACAATTCTGTTGGACAGTTTTCATC

TGTGACTTCAGCACCATGGTGGAGTAATGCCTATGGATCTCAACCTGTTTATGGAGGAGAC

TCTTGTGGCCAAATGAAACCTTTTTCACTAGAGCTTTCCAACTACATAGACCAACTTGCTC

CGAGTAAGAACTTAGTTCGAGGAGTTGAACAATTGTTTGATAAAGGGCATACAAACCAATT

CACCATCTTTCCAGGTACTTGTTATTCAATATAATTCCGGTTTTGAATGAATTGATTTTTG

TTAAGTTGGTTCTGGATAAACGTGCGTTAAATATAATATGATTTATGTTTGGATACGTTTA

TGTTAAAGTGAGTTGAACATAAATTGGAGACTAAATATCAATTGCAGAGGCAAAAGCTTCA

AATTCTAATTTCAAGTTAGAATCAAATCAATTCTACTCGTAAGCATCTAAATATTTCAAAA

CCAATTCTACATCTCTAGAACTAAATTTGCATTCAAACATTTTCTGCTACAGATGATTGTA

AGATGTCAGTTGATACACCAAATCATCAAGCAACCTTATCCCTGCAATCACCATTTGCTGC

CGAGCCACTTAATCGATTTGAGCTAGGTTTTAACCAGTCTATGGTAAATCTTCTTATTAAA

GCAATCCCTTTGATCATGTTTTAGTATTTTACTTTTGATGTACATAAATTCATTATGTTTT

TATTATCCTTTCCATGCTAGATCTGTGCAAAATATCCGTATATAGATCAATTTTACGGGCT

CTTCTCGACTTATGGACCTCAAATCTCGGTTTGTCTTCACCTCGGCTATTTTATTAATCTT

TGTATATATCATGCAATGTCTATAAGTACTTGAATGTGAATTTGTTTTATTTACAGTGTGT

ATGCTTACATGAGAAAGCCAGAAATCTGTTTACAACTATCTTGTTAGTATTCTTCTAAATC

TTTCAAATGTAGAGTTGATTATGAAATTTGTTGTTAATGAGTTTTCGGCTTTATGAATGAA

TTGTAAGACAAGTAATGAAACAAAAACTAGAAGAAAATTGAATTATATGATGCAATCTGTG

TTGAAGTTTTTGCATGTATTCAAGTTCTGCTTTTAGTTACTATTTGTTTATCTGCTGCAAT

ATTTCATCAATTATTATTCATGTATGTACCAATATTTAATAAATTAGGGGCGAATTATGCT

TCCGCTTAGCATGACATCTGACGACGGACCAACATACGTGAATGCTAAGCAATACCATGGA

ATCATCAGACGCAGGCATTCTCGTGCCAAAGCTGTGCTTCAGAATAAATTGATAAAGCGTA

ACAAGGTATGAAACTGAGTATTTTTCCTCACAACAATGTTCGAAAACTTGTGAAATAGTAT

ATTTTTCCTTTTACCCTTTTTATACCTATATTAAGGTTTTGTATTTGTCTTGCAGCCATAT

ATGCACGAATCGCGTCATCTACATGCAATGCGTAGACCAAGAGGATGCGGTGGTCGTTTCT

TGAACACAAAAGTTTCTGCTAATGGAAACGGTAAAAGCGGGAGTGAAGAGAACGGAAACAT

TGGTGGCCTACAGCTGCAGTCCAGTGGTTCTCAGAGTTCCGAAGTCTTACAATCTGAGGTT

GGAACTTTAAATTCGTCGAAGGAGACAAACGGAGGCAGTCCAAATGTCTCGGGGTCAGAGG

TGACTAGCATGTATACACAGGGAGGTCTTGATAGCTTTACTGTCAATCATATCGGATCTAC

TGTCCACTCTTTGGGAGACATGATCGATACTGGACACGGTATCGTCATGCCAACAAAATGG

TTTGCAGCAGCTGGCAGACAGCTGCTGGAACCATAAGTTTCGATTCAGAAAGGAAACAAGT

GGGTTTGGTACAATGTGAAATATTTTGCACCAAACTCATCCTTTCCGAGACCAGATGAAGA

AGCTATGTTTCAGTTTGTTGTGTTTACTACGACAAATTTAGTTTCGGAAGACTACTTTTCC

ATCTGGTGCTCAGGCAACTCATTCTTGGCTTATTCTCAGGAAACTCATCCTTGGCTCGTAA

TATTTAGTAGTATTGTCATTGTCTTCCCGCGCAGGCTTGCCGTGGCATGGTAGGCATGCTA

ATGACTTTGGTATTTTCATGCAGTTATAACTATGATGTGTCTTTGTTTGTTGTTAAAATAA

AAAACATGAACTCTAGCTAGGTGCATGTGTGTGTTTTTAATCTTGTCTACTAAGTTTGGTG

TTTTGTAATGGATTTCTGACTTTATGGAGCAATGTATTGTAACTCTACTAAGAAGTGTAAC

ATTTTATTTCTCCCTCTCTAAGGATTGTATAAGAACCTCTTATTTTCAGACTCTACTTAAT

CCTATTTTCTATGTCTGTATGATTTTTATATTTCTAGGACAATCAAATTGGCTTGTAGAAG

CTCAAAAGCATGCTCACAAAGTAGGTACTTATGTAGGGAACTTCGTACTCTAATAATAACT

GGTTATTAACGTTATAATTAAATGCAAAATTTGATAAGTAGTAGGGTTTGGTAAGTAATAT

AACAACACCATGTTGGCTTGGGATTCTGAATTGTGTTATTAGAGTACAACTCTTAAATAAT

GATCGTGATGTAAATAATTTAAATTTGAAGTTACTAGATAAAAAGATAACATTATGTAATA

TATTATCAAATATATACTAAAAATATAATTTTCATCTATCTCAATTGAAAAAATATCCGGG

TTTTGAGTCTATTGTCAATGACGA

>HM038

AAAAAAATAAAGGTTCTATACTCATTAAACAAAGTGCCAAATTCAGAGATACTACTTCCT

TTGGATAATTTGTATCGTCACCAACGATCTTTGAGTTATTTCAAAAATCACATTCTCAATT

TTGTGGTCCAACATTCACAAAATGGTTTGTCGGGCTTTAAGTCTAGTAATTGTGAAACATG

CATATGCATGAGGTAATTAATTGATAATCTGAAAAGGTTAAATAGTTAATCAATTGTACTT

TGGTTATTTTTAATTTGTTCTCTATAATTTTTCAGTCAAAATTGAATTTGCTTTAACCATT

TAGTGTAGTAGTATGATATTACTTGCTCTACATTCTTGGATCTAAAAGTCTAATTAGTATC

GTATAAAAAATATTCTAGTTAATTAAGATACTTTTGTTCATTCTAATTATTCTTGGATTAC

TTGTGAAACTTTTAGGGATGAACAAGAAAGTCCACTAAAGAGGCTTCTTTTTGCTAGGAGT

CACTAGTTAGTTTTTTTTTTTTTTGACAAAAGTTAACCATTTTTTTTTATCGCATGGTAGG

AAAAAAAAAATAGAGATTTCCTTTGGTACTCATAAAAAAAAAAGTTGGATGGATAATGGAT

TAATCATAACTTATTTAACATAAAATGAATTAAGAGAACATTTGATATTTGGGTTTATTTT

GTAACATAAATATTAATATCAATGTTTGAAAGAGTTTTTAACAATAATTTGTAAAAGAAAG

CATAATATATCTATCAAAATAATTGTAAAAGAAAAAATTTCTTTTGGTGGTATGACATGGA

AAAATAATTAATTCTTATTAGATGAAAGTGTAAAATTAATTTGCATTAACAAAGCATATTT

CATTAAACTCCTTTTAGTTTTTGTTGGAAAAAATAAATTTATTGTTACTCAAAAAATAAAA

GTGTACAATTAATATATTTACTCAAAAGTCCCCTAAATGGGATGTAATTATCAAGGGATGT

GAGCGGTTTAACTTTAAGTAATGAATCAACTGTTGCATAATCACTTTGAAAGAAACTTGAG

ATTCTTCAATTAGCAAAATCTTGAATAGAATAATGAGATTATTCCTAATGCTAATGATACC

CACCTCTCCTATAAGTTGATGGGGCAGAACAAATTATGCACTCAGGACAACTTTTCTCAAA

TTTTGAAAACTTACCACACGAAGAACACACTTCCACTTGCACAAGTAAGAATATAGTATAA

CAATAAATTTACCACTCATAGCTCAATGGATATTAAAATATTGCTAATCTTTTATAGACAG

ATGTTGGCAAATTAGTATAAATTAGTTATTATATTAGTTTTTAAGGTTCAATCTACTTTTT

AAAACTCTTATTAAGTTGTTTAGCTTGCCAACCAAAGACTAAATTATCTTTATTTGTTAGG

AAATAAAAGCACTTATTTTATTTTCTCCATATTTGTTTCCTCTTTGTTCCATTACTAAAAA

AATTATAATATATTAATAACAAAAAAATTTGGGGAGGGAATGTTATTTTTAAAAAAGATGT

TTTATTTATATTTAAGTGTTTTATTGAAATTCCTCGTGAGTTTAGCTCAATTGGTATGGAC

AATACATAATATATGCAAAATTCGAGGTTCAAACCTTGAGCACCAAAAAAAAAAGTGTTTT

ATTGAAAAATATCAAGAACTTGAAAACAAATTTCCTTTTTATGAACTAGATATTGTCACAT

AGACAACTTAAGAATTGACATTTTTTTTAACACTATTGTATTTTCTGAACTCGACTTTAAC

TCGAGATCAATGAGTAAACTAAAAAAACTCGTATTATTTTATCTAAGTGTTCTTAGTGTGA

GAAAACATTATAATTGTTTGCTTAGTCTATTTCTCCCTATATTCTCACCCTCATTCTTGGT

TTGCACATAGAAAGGAGAAAAAAAAAATTAGAGAAGAGTTGAATGGAACAGAAAGAAAGAG

CAAGCAAAATAATCACTAAAAAAAATTCCCCTAAAAGAAAATCACTGAAAAATATTATCCA

CCTCTATTTTAAATAATAATAATAATGAAAACAACAATATATTTTTTTATAGATTTTCTCT

ACTAGAGGTAATTTATTTTAAACACTAAATGTCCACACCTCTAACAAAAAAAATGCATAAT

ATTATAATAGATTATTGTAAGAAAGAGTGACAAATGTTGAATTAGTTATGGAATGAAGAGT

GAGAGAGATTATACAAATAAATAAATATAAATATAAATATAAATAAAGAATAGAGAAGAAC

ATAGTACTTGAATGAAGAGATTTGGCTTGTACTTCCATTACACTTGTAAGCTTTCTACTTC

CTTCACATTCCTTCTCTTTCTCTATCTTTCCTTTTATGATATGTGTACTTTGTTATTGCTT

CATTTACTACCGACATGACTCCAACCTATTAGGGCTTTATCTGAATAATGTTAGTTATTTT

CTCTCATTTTCTTTGCTTCTTCTTATTCTTGGAACTTATAAAGGGTCTTGTCAATTCTATC

ATTCTCTTTTGTATACTTACAAATATAAATATTCTCTTTAGCTTCATTTTCATGTCTTCTT

CTACTAATAAGCTATGGAAATTGAAGTTTTTGTTTTCATGTTTATTTTTCACTTCAGCTCC

TCTTTTTTGATGTTGATGCTGAAGATTGAATAAAAACTGAGGTGGTATGTATGAGAACAAG

AACATTTTTCATTCATCTCTGTTATTTGAACATCTAAATTTTAAACAACTATCGCGACAAT

TTTTTAAGTGGTTGAAGTATTTTAAATAGGTGGTTAATTACATTAAGTAGATAGTTAATTG

ATATATCAGGTGTCAAAACTCATTAACCATCAACATAACTTCATTAGCCATAAATTTTTGA

CGTGATTAACCAAATATTGTAGATGCTCAATTACATGTAGATTTGATGGAGTTGTCCACAA

TTTCTCATATATCTATTAACTAATAACCATCTACCGAATATAATTAACCATATATTAGAAT

ATCATTAACCTCTAAAACAGTTGTCGCGAAAGTTGGTTGTACAAAAATCATTTCTATTTTT

TACGCCACTAGTTCTCTTGTTATTTCTCTTTGTTGGAAAGTTGTTGTTTTTAACTTTGAGC

TTAGTCTTATTAATTAACTTGTAATGGTAACAACATTTTTCTATCTTTGTTTGAAGGAAGT

AGTTACTGTGTCAACTTTAGTAATTAATTTGGATCATTTTGGAGGCAAAATATGGCTATGC

AACCTGTTTATTTTAAAGAACATGAAGGAAATGTCCACAATTCTGTTGGACAGTTTTCATC

TGTGACTTCAGCACCATGGTGGAGTAATGCCTATGGATCTCAACCTGTTTATGGAGGAGAC

TCTTGTGGCCAAATGAAACCTTTTTCACTAGAGCTTTCCAACTACATAGACCAACTTGCTC

CGAGTAAGAACTTAGTTCGAGGAGTTGAACAATTGTTTGATAAAGGGCATACAAACCAATT

CACCATCTTTCCAGGTACTTGTTATTCAATATAATTCCGGTTTTGAATGAATTGATTTTTG

TTAAGTTGGTTCTGGATAAACGTGCGTTAAATATAATATGATTTATGTTTGGATACGTTTA

TGTTAAAGTGAGTTGAACATAAATTGGAGACTAAATATCAATTGCAGAGGCAAAAGCTTCA

AATTCTAATTTCAAGTTAGAATCAAATCAATTCTACTCGTAAGCATCTAAATATTTCAAAA

CCAATTCTACATCTCTAGAACTAAATTTGCATTCAAACATTTTCTGCTACAGATGATTGTA

AGATGTCAGTTGATACACCAAATCATCAAGCAACCTTATCCCTGCAATCACCATTTGCTGC

CGAGCCACTTAATCGATTTGAGCTAGGTTTTAACCAGTCTATGGTAAATCTTCTTATTAAA

GCAATCCCTTTGATCATGTTTTAGTATTTTACTTTTGATGTACATAAATTCATTATGTTTT

TATTATCCTTTCCATGCTAGATCTGTGCAAAATATCCGTATATAGATCAATTTTACGGGCT

CTTCTCGACTTATGGACCTCAAATCTCGGTTTGTCTTCACCTCGGCTATTTTATTAATCTT

TGTATATATCATGCAATGTCTATAAGTACTTGAATGTGAATTTGTTTTATTTACAGTGTGT

ATGCTTACATGAGAAAGCCAGAAATCTGTTTACAACTATCTTGTTAGTATTCTTCTAAATC

TTTCAAATGTAGAGTTGATTATGAAATTTGTTGTTAATGAGTTTTCGGCTTTATGAATGAA

TTGTAAGACAAGTAATGAAACAAAAACTAGAAGAAAATTGAATTATATGATGCAATCTGTG

TTGAAGTTTTTGCATGTATTCAAGTTCTGCTTTTAGTTACTATTTGTTTATCTGCTGCAAT

ATTTCATCAATTATTATTCATGTATGTACCAATATTTAATAAATTAGGGGCGAATTATGCT

TCCGCTTAGCATGACATCTGACGACGGACCAACATACGTGAATGCTAAGCAATACCATGGA

ATCATCAGACGCAGGCATTCTCGTGCCAAAGCTGTGCTTCAGAATAAATTGATAAAGCGTA

ACAAGGTATGAAACTGAGTATTTTTCCTCACAACAATGTTCGAAAACTTGTGAAATAGTAT

ATTTTTCCTTTTACCCTTTTTATACCTATATTAAGGTTTTGTATTTGTCTTGCAGCCATAT

ATGCACGAATCGCGTCATCTACATGCAATGCGTAGACCAAGAGGATGCGGTGGTCGTTTCT

TGAACACAAAAGTTTCTGCTAATGGAAACGGTAAAAGCGGGAGTGAAGAGAACGGAAACAT

TGGTGGCCTACAGCTGCAGTCCAGTGGTTCTCAGAGTTCCGAAGTCTTACAATCTGAGGTT

GGAACTTTAAATTCGTCGAAGGAGACAAACGGAGGCAGTCCAAATGTCTCGGGGTCAGAGG

TGACTAGCATGTATACACAGGGAGGTCTTGATAGCTTTACTGTCAATCATATCGGATCTAC

TGTCCACTCTTTGGGAGACATGATCGATACTGGACACGGTATCGTCATGCCAACAAAATGG

TTTGCAGCAGCTGGCAGACAGCTGCTGGAACCATAAGTTTCGATTCAGAAAGGAAACAAGT

GGGTTTGGTACAATGTGAAATATTTTGCACCAAACTCATCCTTTCCGAGACCAGATGAAGA

AGCTATGTTTCAGTTTGTTGTGTTTACTACGACAAATTTAGTTTCGGAAGACTACTTTTCC

ATCTGGTGCTCAGGCAACTCATTCTTGGCTTATTCTCAGGAAACTCATCCTTGGCTCGTAA

TATTTAGTAGTATTGTCATTGTCTTCCCGCGCAGGCTTGCCGTGGCATGGTAGGCATGCTA

ATGACTTTGGTATTTTCATGCAGTTATAACTATGATGTGTCTTTGTTTGTTGTTAAAATAA

AAAACATGAACTCTAGCTAGGTGCATGTGTGTGTTTTTAATCTTGTCTACTAAGTTTGGTG

TTTTGTAATGGATTTCTGACTTTATGGAGCAATGTATTGTAACTCTACTAAGAAGTGTAAC

ATTTTATTTCTCCCTCTCTAAGGATTGTATAAGAACCTCTTATTTTCAGACTCTACTTAAT

CCTATTTTCTATGTCTGTATGATTTTTATATTTCTAGGACAATCAAATTGGCTTGTAGAAG

CTCAAAAGCATGCTCACAAAGTAGGTACTTATGTAGGGAACTTCGTACTCTAATAATAACT

GGTTATTAACGTTATAATTAAATGCAAAATTTGATAAGTAGTAGGGTTTGGTAAGTAATAT

AACAACACCATGTTGGCTTGGGATTCTGAATTGTGTTATTAGAGTACAACTCTTAAATAAT

GATCGTGATGTAAATAATTTAAATTTGAAGTTACTAGATAAAAAGATAACATTATGTAATA

TATTATCAAATATATACTAAAAATATAATTTTCATCTATCTCAATTGAAAAAATATCCGGG

TTTTGAGTCTATTGTCAATGACGA

>HM039

AAAAAAATAAAGGTTCTATACTCATTAAACAAAGTGCCAAATTCAGAGATACTACTTCCT

TTGGATAATTTGTATCGTCACCAACGATCTTTGAGTTATTTCAAAAATCACATTCTCAATT

TTGTGGTCCAACATTCACAAAATGGTTTGTCGGGCTTTAAGTCTAGTAATTGTGAAACATG

CATATGCATGAGGTAATTAATTGATAATCTGAAAAGGTTAAATAGTTAATCAATTGTACTT

TGGTTATTTTTAATTTGTTCTCTATAATTTTTCAGTCAAAATTGAATTTGCTTTAACCATT

TAGTGTAGTAGTATGATATTACTTGCTCTACATTCTTGGATCTAAAAGTCTAATTAGTATC

GTATAAAAAATATTCTAGTTAATTAAGATACTTTTGTTCATTCTAATTATTCTTGGATTAC

TTGTGAAACTTTTAGGGATGAACAAGAAAGTCCACTAAAGAGGCTTCTTTTTGCTAGGAGT

CACTAGTTAGTTTTTTTTTTTTTTGACAAAAGTTAACCATTTTTTTTTATCGCATGGTAGG

AAAAAAAAAATAGAGATTTCCTTTGGTACTCATAAAAAAAAAAGTTGGATGGATAATGGAT

TAATCATAACTTATTTAACATAAAATGAATTAAGAGAACATTTGATATTTGGGTTTATTTT

GTAACATAAATATTAATATCAATGTTTGAAAGAGTTTTTAACAATAATTTGTAAAAGAAAG

CATAATATATCTATCAAAATAATTGTAAAAGAAAAAATTTCTTTTGGTGGTATGACATGGA

AAAATAATTAATTCTTATTAGATGAAAGTGTAAAATTAATTTGCATTAACAAAGCATATTT

CATTAAACTCCTTTTAGTTTTTGTTGGAAAAAATAAATTTATTGTTACTCAAAAAATAAAA

GTGTACAATTAATATATTTACTCAAAAGTCCCCTAAATGGGATGTAATTATCAAGGGATGT

GAGCGGTTTAACTTTAAGTAATGAATCAACTGTTGCATAATCACTTTGAAAGAAACTTGAG

ATTCTTCAATTAGCAAAATCTTGAATAGAATAATGAGATTATTCCTAATGCTAATGATACC

CACCTCTCCTATAAGTTGATGGGGCAGAACAAATTATGCACTCAGGACAACTTTTCTCAAA

TTTTGAAAACTTACCACACGAAGAACACACTTCCACTTGCACAAGTAAGAATATAGTATAA

CAATAAATTTACCACTCATAGCTCAATGGATATTAAAATATTGCTAATCTTTTATAGACAG

ATGTTGGCAAATTAGTATAAATTAGTTATTATATTAGTTTTTAAGGTTCAATCTACTTTTT

AAAACTCTTATTAAGTTGTTTAGCTTGCCAACCAAAGACTAAATTATCTTTATTTGTTAGG

AAATAAAAGCACTTATTTTATTTTCTCCATATTTGTTTCCTCTTTGTTCCATTACTAAAAA

AATTATAATATATTAATAACAAAAAAATTTGGGGAGGGAATGTTATTTTTAAAAAAGATGT

TTTATTTATATTTAAGTGTTTTATTGAAATTCCTCGTGAGTTTAGCTCAATTGGTATGGAC

AATACATAATATATGCAAAATTCGAGGTTCAAACCTTGAGCACCAAAAAAAAAAGTGTTTT

ATTGAAAAATATCAAGAACTTGAAAACAAATTTCCTTTTTATGAACTAGATATTGTCACAT

AGACAACTTAAGAATTGACATTTTTTTTAACACTATTGTATTTTCTGAACTCGACTTTAAC

TCGAGATCAATGAGTAAACTAAAAAAACTCGTATTATTTTATCTAAGTGTTCTTAGTGTGA

GAAAACATTATAATTGTTTGCTTAGTCTATTTCTCCCTATATTCTCACCCTCATTCTTGGT

TTGCACATAGAAAGGAGAAAAAAAAAATTAGAGAAGAGTTGAATGGAACAGAAAGAAAGAG

CAAGCAAAATAATCACTAAAAAAAATTCCCCTAAAAGAAAATCACTGAAAAATATTATCCA

CCTCTATTTTAAATAATAATAATAATGAAAACAACAATATATTTTTTTATAGATTTTCTCT

ACTAGAGGTAATTTATTTTAAACACTAAATGTCCACACCTCTAACAAAAAAAATGCATAAT

ATTATAATAGATTATTGTAAGAAAGAGTGACAAATGTTGAATTAGTTATGGAATGAAGAGT

GAGAGAGATTATACAAATAAATAAATATAAATATAAATATAAATAAAGAATAGAGAAGAAC

ATAGTACTTGAATGAAGAGATTTGGCTTGTACTTCCATTACACTTGTAAGCTTTCTACTTC

CTTCACATTCCTTCTCTTTCTCTATCTTTCCTTTTATGATATGTGTACTTTGTTATTGCTT

CATTTACTACCGACATGACTCCAACCTATTAGGGCTTTATCTGAATAATGTTAGTTATTTT

CTCTCATTTTCTTTGCTTCTTCTTATTCTTGGAACTTATAAAGGGTCTTGTCAATTCTATC

ATTCTCTTTTGTATACTTACAAATATAAATATTCTCTTTAGCTTCATTTTCATGTCTTCTT

CTACTAATAAGCTATGGAAATTGAAGTTTTTGTTTTCATGTTTATTTTTCACTTCAGCTCC

TCTTTTTTGATGTTGATGCTGAAGATTGAATAAAAACTGAGGTGGTATGTATGAGAACAAG

AACATTTTTCATTCATCTCTGTTATTTGAACATCTAAATTTTAAACAACTATCGCGACAAT

TTTTTAAGTGGTTGAAGTATTTTAAATAGGTGGTTAATTACATTAAGTAGATAGTTAATTG

ATATATCAGGTGTCAAAACTCATTAACCATCAACATAACTTCATTAGCCATGAATTTTTGA

CGTGATTAACCAAATATTGTAGATGCTCAATTACATGTAGATTTGATGGAGTTGTCCACAA

TTTCTCATATATCTATTAACTAATAACCATCTACCGAATATAATTAACCATATATTAGAAT

ATCATTAACCTCTAAAACAGTTGTCGCGAAAGTTGGTTGTACAAAAATCATTTCTATTTTT

TACGCCACTAGTTCTCTTGTTATTTCTCTTTGTTGGAAAGTTGTTGTTTTTAACTTTGAGC

TTAGTCTTATTAATTAACTTGTAATGGTAACAACATTTTTCTATCTTTGTTTGAAGGAAGT

AGTTACTGTGTCAACTTTAGTAATTAATTTGGATCATTTTGGAGGCAAAATATGGCTATGC

AACCTGTTTATTTTAAAGAACATGAAGGAAATGTCCACAATTCTGTTGGACAGTTTTCATC

TGTGACTTCAGCACCATGGTGGAGTAATGCCTATGGATCTCAACCTGTTTATGGAGGAGAC

TCTTGTGGCCAAATGAAACCTTTTTCACTAGAGCTTTCCAACTACATAGACCAACTTGCTC

CGAGTAAGAACTTAGTTCGAGGAGTTGAACAATTGTTTGATAAAGGGCATACAAACCAATT

CACCATCTTTCCAGGTACTTGTTATTCAATATAATTCCGGTTTTGAATGAATTGATTTTTG

TTAAGTTGGTTCTGGATAAACGTGCGTTAAATATAATATGATTTATGTTTGGATACGTTTA

TGTTAAAGTGAGTTGAACATAAATTGGAGACTAAATATCAATTGCAGAGGCAAAAGCTTCA

AATTCTAATTTCAAGTTAGAATCAAATCAATTCTACTCGTAAGCATCTAAATATTTCAAAA

CCAATTCTACATCTCTAGAACTAAATTTGCATTCAAACATTTTCTGCTACAGATGATTGTA

AGATGTCAGTTGATACACCAAATCATCAAGCAACCTTATCCCTGCAATCACCATTTGCTGC

CGAGCCACTTAATCGATTTGAGCTAGGTTTTAACCAGTCTATGGTAAATCTTCTTATTAAA

GCAATCCCTTTGATCATGTTTTAGTATTTTACTTTTGATGTACATAAATTCATTATGTTTT

TATTATCCTTTCCATGCTAGATCTGTGCAAAATATCCGTATATAGATCAATTTTACGGGCT

CTTCTCGACTTATGGACCTCAAATCTCGGTTTGTCTTCACCTCGGCTATTTTATTAATCTT

TGTATATATCATGCAATGTCTATAAGTACTTGAATGTGAATTTGTTTTATTTACAGTGTGT

ATGCTTACATGAGAAAGCCAGAAATCTGTTTACAACTATCTTGTTAGTATTCTTCTAAATC

TTTCAAATGTAGAGTTGATTATGAAATTTGTTGTTAATGAGTTTTCGGCTTTATGAATGAA

TTGTAAGACAAGTAATGAAACAAAAACTAGAAGAAAATTGAATTATATGATGCAATCTGTG

TTGAAGTTTTTGCATGTATTCAAGTTCTGCTTTTAGTTACTATTTGTTTATCTGCTGCAAT

ATTTCATCAATTATTATTCATGTATGTACCAATATTTAATAAATTAGGGGCGAATTATGCT

TCCGCTTAGCATGACATCTGACGACGGACCAACATACGTGAATGCTAAGCAATACCATGGA

ATCATCAGACGCAGGCATTCTCGTGCCAAAGCTGTGCTTCAGAATAAATTGATAAAGCGTA

ACAAGGTATGAAACTGAGTATTTTTCCTCACAACAATGTTCGAAAACTTGTGAAATAGTAT

ATTTTTCCTTTTACCCTTTTTATACCTATATTAAGGTTTTGTATTTGTCTTGCAGCCATAT

ATGCACGAATCGCGTCATCTACATGCAATGCGTAGACCAAGAGGATGCGGTGGTCGTTTCT

TGAACACAAAAGTTTCTGCTAATGGAAACGGTAAAAGCGGGAGTGAAGAGAACGGAAACAT

TGGTGGCCTACAGCTGCAGTCCAGTGGTTCTCAGAGTTCCGAAGTCTTACAATCTGAGGTT

GGAACTTTAAATTCGTCGAAGGAGACAAACGGAGGCAGTCCAAATGTCTCGGGGTCAGAGG

TGACTAGCATGTATACACAGGGAGGTCTTGATAGCTTTACTGTCAATCATATCGGATCTAC

TGTCCACTCTTTGGGAGACATGATCGATACTGGACACGGTATCGTCATGCCAACAAAATGG

TTTGCAGCAGCTGGCAGACAGCTGCTGGAACCATAAGTTTCGATTCAGAAAGGAAACAAGT

GGGTTTGGTACAATGTGAAATATTTTGCACCAAACTCATCCTTTCCGAGACCAGATGAAGA

AGCTATGTTTCAGTTTGTTGTGTTTACTACGACAAATTTAGTTTCGGAAGACTACTTTTCC

ATCTGGTGCTCAGGCAACTCATTCTTGGCTTATTCTCAGGAAACTCATCCTTGGCTCGTAA

TATTTAGTAGTATTGTCATTGTCTTCCCGCGCAGGCTTGCCGTGGCATGGTAGGCATGCTA

ATGACTTTGGTATTTTCATGCAGTTATAACTATGATGTGTCTTTGTTTGTTGTTAAAATAA

AAAACATGAACTCTAGCTAGGTGCATGTGTGTGTTTTTAATCTTGTCTACTAAGTTTGGTG

TTTTGTAATGGATTTCTGACTTTATGGAGCAATGTATTGTAACTCTACTAAGAAGTGTAAC

ATTTTATTTCTCCCTCTCTAAGGATTGTATAAGAACCTCTTATTTTCAGACTCTACTTAAT

CCTATTTTCTATGTCTGTATGATTTTTATATTTCTAGGACAATCAAATTGGCTTGTAGAAG

CTCAAAAGCATGCTCACAAAGTAGGTACTTATGTAGGGAACTTCGTACTCTAATAATAACT

GGTTATTAACGTTATAATTAAATGCAAAATTTGATAAGTAGTAGGGTTTGGTAAGTAATAT

AACAACACCATGTTGGCTTGGGATTCTGAATTGTGTTATTAGAGTACAACTCTTAAATAAT

GATCGTGATGTAAATAATTTAAATTTGAAGTTACTAGATAAAAAGATAACATTATGTAATA

TATTATCAAATATATACTAAAAATATAATTTTCATCTATCTCAATTGAAAAAATATCCGGG

TTTTGAGTCTATTGTCAATGACGA

>HM040

AAAAAAATAAAGGTTCTATACTCATTAAACAAAGTGCCAAATTCAGAGATACTACTTCCT

TTGGATAATTTGTATCGTCACCAACGATCTTTGAGTTATTTCAAAAATCACATTCTCAATT

TTGTGGTCCAACATTCACAAAATGGTTTGTCGGGCTTTAAGTCTAGTAATTGTGAAACATG

CATATGCATGAGGTAATTAATTGATAATCTGAAAAGGTTAAATAGTTAATCAATTGTACTT

TGGTTATTTTTAATTTGTTCTCTATAATTTTTCAGTCAAAATTGAATTTGCTTTAACCATT

TAGTGTAGTAGTATGATATTACTTGCTCTACATTCTTGGATCTAAAAGTCTAATTAGTATC

GTATAAAAAATATTCTAGTTAATTAAGATACTTTTGTTCATTCTAATTATTCTTGGATTAC

TTGTGAAACTTTTAGGGATGAACAAGAAAGTCCACTAAAGAGGCTTCTTTTTGCTAGGAGT

CACTAGTTAGTTTTTTTTTTTTTTGACAAAAGTTAACCATTTTTTTTTATCGCATGGTAGG

AAAAAAAAAATAGAGATTTCCTTTGGTACTCATAAAAAAAAAAGTTGGATGGATAATGGAT

TAATCATAACTTATTTAACATAAAATGAATTAAGAGAACATTTGATATTTGGGTTTATTTT

GTAACATAAATATTAATATCAATGTTTGAAAGAGTTTTTAACAATAATTTGTAAAAGAAAG

CATAATATATCTATCAAAATAATTGTAAAAGAAAAAATTTCTTTTGGTGGTATGACATGGA

AAAATAATTAATTCTTATTAGATGAAAGTGTAAAATTAATTTGCATTAACAAAGCATATTT

CATTAAACTCCTTTTAGTTTTTGTTGGAAAAAATAAATTTATTGTTACTCAAAAAATAAAA

GTGTACAATTAATATATTTACTCAAAAGTCCCCTAAATGGGATGTAATTATCAAGGGATGT

GAGCGGTTTAACTTTAAGTAATGAATCAACTGTTGCATAATCACTTTGAAAGAAACTTGAG

ATTCTTCAATTAGCAAAATCTTGAATAGAATAATGAGATTATTCCTAATGCTAATGATACC

CACCTCTCCTATAAGTTGATGGGGCAGAACAAATTATGCACTCAGGACAACTTTTCTCAAA

TTTTGAAAACTTACCACACGAAGAACACACTTCCACTTGCACAAGTAAGAATATAGTATAA

CAATAAATTTACCACTCATAGCTCAATGGATATTAAAATATTGCTAATCTTTTATAGACAG

ATGTTGGCAAATTAGTATAAATTAGTTATTATATTAGTTTTTAAGGTTCAATCTACTTTTT

AAAACTCTTATTAAGTTGTTTAGCTTGCCAACCAAAGACTAAATTATCTTTATTTGTTAGG

AAATAAAAGCACTTATTTTATTTTCTCCATATTTGTTTCCTCTTTGTTCCATTACTAAAAA

AATTATAATATATTAATAACAAAAAAATTTGGGGAGGGAATGTTATTTTTAAAAAAGATGT

TTTATTTATATTTAAGTGTTTTATTGAAATTCCTCGTGAGTTTAGCTCAATTGGTATGGAC

AATACATAATATATGCAAAATTCGAGGTTCAAACCTTGAGCACCAAAAAAAAAAGTGTTTT

ATTGAAAAATATCAAGAACTTGAAAACAAATTTCCTTTTTATGAACTAGATATTGTCACAT

AGACAACTTAAGAATTGACATTTTTTTTAACACTATTGTATTTTCTGAACTCGACTTTAAC

TCGAGATCAATGAGTAAACTAAAAAAACTCGTATTATTTTATCTAAGTGTTCTTAGTGTGA

GAAAACATTATAATTGTTTGCTTAGTCTATTTCTCCCTATATTCTCACCCTCATTCTTGGT

TTGCACATAGAAAGGAGAAAAAAAAAATTAGAGAAGAGTTGAATGGAACAGAAAGAAAGAG

CAAGCAAAATAATCACTAAAAAAAATTCCCCTAAAAGAAAATCACTGAAAAATATTATCCA

CCTCTATTTTAAATAATAATAATAATGAAAACAACAATATATTTTTTTATAGATTTTCTCT

ACTAGAGGTAATTTATTTTAAACACTAAATGTCCACACCTCTAACAAAAAAAATGCATAAT

ATTATAATAGATTATTGTAAGAAAGAGTGACAAATGTTGAATTAGTTATGGAATGAAGAGT

GAGAGAGATTATACAAATAAATAAATATAAATATAAATATAAATAAAGAATAGAGAAGAAC

ATAGTACTTGAATGAAGAGATTTGGCTTGTACTTCCATTACACTTGTAAGCTTTCTACTTC

CTTCACATTCCTTCTCTTTCTCTATCTTTCCTTTTATGATATGTGTACTTTGTTATTGCTT

CATTTACTACCGACATGACTCCAACCTATTAGGGCTTTATCTGAATAATGTTAGTTATTTT

CTCTCATTTTCTTTGCTTCTTCTTATTCTTGGAACTTATAAAGGGTCTTGTCAATTCTATC

ATTCTCTTTTGTATACTTACAAATATAAATATTCTCTTTAGCTTCATTTTCATGTCTTCTT

CTACTAATAAGCTATGGAAATTGAAGTTTTTGTTTTCATGTTTATTTTTCACTTCAGCTCC

TCTTTTTTGATGTTGATGCTGAAGATTGAATAAAAACTGAGGTGGTATGTATGAGAACAAG

AACATTTTTCATTCATCTCTGTTATTTGAACATCTAAATTTTAAACAACTATCGCGACAAT

TTTTTAAGTGGTTGAAGTATTTTAAATAGGTGGTTAATTACATTAAGTAGATAGTTAATTG

ATATATCAGGTGTCAAAACTCATTAACCATCAACATAACTTCATTAGCCATGAATTTTTGA

CGTGATTAACCAAATATTGTAGATGCTCAATTACATGTAGATTTGATGGAGTTGTCCACAA

TTTCTCATATATCTATTAACTAATAACCATCTACCGAATATAATTAACCATATATTAGAAT

ATCATTAACCTCTAAAACAGTTGTCGCGAAAGTTGGTTGTACAAAAATCATTTCTATTTTT

TACGCCACTAGTTCTCTTGTTATTTCTCTTTGTTGGAAAGTTGTTGTTTTTAACTTTGAGC

TTAGTCTTATTAATTAACTTGTAATGGTAACAACATTTTTCTATCTTTGTTTGAAGGAAGT

AGTTACTGTGTCAACTTTAGTAATTAATTTGGATCATTTTGGAGGCAAAATATGGCTATGC

AACCTGTTTATTTTAAAGAACATGAAGGAAATGTCCACAATTCTGTTGGACAGTTTTCATC

TGTGACTTCAGCACCATGGTGGAGTAATGCCTATGGATCTCAACCTGTTTATGGAGGAGAC

TCTTGTGGCCAAATGAAACCTTTTTCACTAGAGCTTTCCAACTACATAGACCAACTTGCTC

CGAGTAAGAACTTAGTTCGAGGAGTTGAACAATTGTTTGATAAAGGGCATACAAACCAATT

CACCATCTTTCCAGGTACTTGTTATTCAATATAATTCCGGTTTTGAATGAATTGATTTTTG

TTAAGTTGGTTCTGGATAAACGTGCGTTAAATATAATATGATTTATGTTTGGATACGTTTA

TGTTAAAGTGAGTTGAACATAAATTGGAGACTAAATATCAATTGCAGAGGCAAAAGCTTCA

AATTCTAATTTCAAGTTAGAATCAAATCAATTCTACTCGTAAGCATCTAAATATTTCAAAA

CCAATTCTACATCTCTAGAACTAAATTTGCATTCAAACATTTTCTGCTACAGATGATTGTA

AGATGTCAGTTGATACACCAAATCATCAAGCAACCTTATCCCTGCAATCACCATTTGCTGC

CGAGCCACTTAATCGATTTGAGCTAGGTTTTAACCAGTCTATGGTAAATCTTCTTATTAAA

GCAATCCCTTTGATCATGTTTTAGTATTTTACTTTTGATGTACATAAATTCATTATGTTTT

TATTATCCTTTCCATGCTAGATCTGTGCAAAATATCCGTATATAGATCAATTTTACGGGCT

CTTCTCGACTTATGGACCTCAAATCTCGGTTTGTCTTCACCTCGGCTATTTTATTAATCTT

TGTATATATCATGCAATGTCTATAAGTACTTGAATGTGAATTTGTTTTATTTACAGTGTGT

ATGCTTACATGAGAAAGCCAGAAATCTGTTTACAACTATCTTGTTAGTATTCTTCTAAATC

TTTCAAATGTAGAGTTGATTATGAAATTTGTTGTTAATGAGTTTTCGGCTTTATGAATGAA

TTGTAAGACAAGTAATGAAACAAAAACTAGAAGAAAATTGAATTATATGATGCAATCTGTG

TTGAAGTTTTTGCATGTATTCAAGTTCTGCTTTTAGTTACTATTTGTTTATCTGCTGCAAT

ATTTCATCAATTATTATTCATGTATGTACCAATATTTAATAAATTAGGGGCGAATTATGCT

TCCGCTTAGCATGACATCTGACGACGGACCAACATACGTGAATGCTAAGCAATACCATGGA

ATCATCAGACGCAGGCATTCTCGTGCCAAAGCTGTGCTTCAGAATAAATTGATAAAGCGTA

ACAAGGTATGAAACTGAGTATTTTTCCTCACAACAATGTTCGAAAACTTGTGAAATAGTAT

ATTTTTCCTTTTACCCTTTTTATACCTATATTAAGGTTTTGTATTTGTCTTGCAGCCATAT

ATGCACGAATCGCGTCATCTACATGCAATGCGTAGACCAAGAGGATGCGGTGGTCGTTTCT

TGAACACAAAAGTTTCTGCTAATGGAAACGGTAAAAGCGGGAGTGAAGAGAACGGAAACAT

TGGTGGCCTACAGCTGCAGTCCAGTGGTTCTCAGAGTTCCGAAGTCTTACAATCTGAGGTT

GGAACTTTAAATTCGTCGAAGGAGACAAACGGAGGCAGTCCAAATGTCTCGGGGTCAGAGG

TGACTAGCATGTATACACAGGGAGGTCTTGATAGCTTTACTGTCAATCATATCGGATCTAC

TGTCCACTCTTTGGGAGACATGATCGATACTGGACACGGTATCGTCATGCCAACAAAATGG

TTTGCAGCAGCTGGCAGACAGCTGCTGGAACCATAAGTTTCGATTCAGAAAGGAAACAAGT

GGGTTTGGTACAATGTGAAATATTTTGCACCAAACTCATCCTTTCCGAGACCAGATGAAGA

AGCTATGTTTCAGTTTGTTGTGTTTACTACGACAAATTTAGTTTCGGAAGACTACTTTTCC

ATCTGGTGCTCAGGCAACTCATTCTTGGCTTATTCTCAGGAAACTCATCCTTGGCTCGTAA

TATTTAGTAGTATTGTCATTGTCTTCCCGCGCAGGCTTGCCGTGGCATGGTAGGCATGCTA

ATGACTTTGGTATTTTCATGCAGTTATAACTATGATGTGTCTTTGTTTGTTGTTAAAATAA

AAAACATGAACTCTAGCTAGGTGCATGTGTGTGTTTTTAATCTTGTCTACTAAGTTTGGTG

TTTTGTAATGGATTTCTGACTTTATGGAGCAATGTATTGTAACTCTACTAAGAAGTGTAAC

ATTTTATTTCTCCCTCTCTAAGGATTGTATAAGAACCTCTTATTTTCAGACTCTACTTAAT

CCTATTTTCTATGTCTGTATGATTTTTATATTTCTAGGACAATCAAATTGGCTTGTAGAAG

CTCAAAAGCATGCTCACAAAGTAGGTACTTATGTAGGGAACTTCGTACTCTAATAATAACT

GGTTATTAACGTTATAATTAAATGCAAAATTTGATAAGTAGTAGGGTTTGGTAAGTAATAT

AACAACACCATGTTGGCTTGGGATTCTGAATTGTGTTATTAGAGTACAACTCTTAAATAAT

GATCGTGATGTAAATAATTTAAATTTGAAGTTACTAGATAAAAAGATAACATTATGTAATA

TATTATCAAATATATACTAAAAATATAATTTTCATCTATCTCAATTGAAAAAATATCCGGG

TTTTGAGTCTATTGTCAATGACGA

>HM041

AAAAAAATAAAGGTTCTATACTCATTAAACAAAGTGCCAAATTCAGAGATACTACTTCCT

TTGGATAATTTGTATCGTCACCAACGATCTTTGAGTTATTTCAAAAATCACATTCTCAATT

TTGTGGTCCAACATTCACAAAATGGTTTGTCGGGCTTTAAGTCTAGTAATTGTGAAACATG

CATATGCATGAGGTAATTAATTGATAATCTGAAAAGGTTAAATAGTTAATCAATTGTACTT

TGGTTATTTTTAATTTGTTCTCTATAATTTTTCAGTCAAAATTGAATTTGCTTTAACCATT

TAGTGTAGTAGTATGATATTACTTGCTCTACATTCTTGGATCTAAAAGTCTAATTAGTATC

GTATAAAAAATATTCTAGTTAATTAAGATACTTTTGTTCATTCTAATTATTCTTGGATTAC

TTGTGAAACTTTTAGGGATGAACAAGAAAGTCCACTAAAGAGGCTTCTTTTTGCTAGGAGT

CACTAGTTAGTTTTTTTTTTTTTTGACAAAAGTTAACCATTTTTTTTTATCGCATGGTAGG

AAAAAAAAAATAGAGATTTCCTTTGGTACTCATAAAAAAAAAAGTTGGATGGATAATGGAT

TAATCATAACTTATTTAACATAAAATGAATTAAGAGAACATTTGATATTTGGGTTTATTTT

GTAACATAAATATTAATATCAATGTTTGAAAGAGTTTTTAACAATAATTTGTAAAAGAAAG

CATAATATATCTATCAAAATAATTGTAAAAGAAAAAATTTCTTTTGGTGGTATGACATGGA

AAAATAATTAATTCTTATTAGATGAAAGTGTAAAATTAATTTGCATTAACAAAGCATATTT

CATTAAACTCCTTTTAGTTTTTGTTGGAAAAAATAAATTTATTGTTACTCAAAAAATAAAA

GTGTACAATTAATATATTTACTCAAAAGTCCCCTAAATGGGATGTAATTATCAAGGGATGT

GAGCGGTTTAACTTTAAGTAATGAATCAACTGTTGCATAATCACTTTGAAAGAAACTTGAG

ATTCTTCAATTAGCAAAATCTTGAATAGAATAATGAGATTATTCCTAATGCTAATGATACC

CACCTCTCCTATAAGTTGATGGGGCAGAACAAATTATGCACTCAGGACAACTTTTCTCAAA

TTTTGAAAACTTACCACACGAAGAACACACTTCCACTTGCACAAGTAAGAATATAGTATAA

CAATAAATTTACCACTCATAGCTCAATGGATATTAAAATATTGCTAATCTTTTATAGACAG

ATGTTGGCAAATTAGTATAAATTAGTTATTATATTAGTTTTTAAGGTTCAATCTACTTTTT

AAAACTCTTATTAAGTTGTTTAGCTTGCCAACCAAAGACTAAATTATCTTTATTTGTTAGG

AAATAAAAGCACTTATTTTATTTTCTCCATATTTGTTTCCTCTTTGTTCCATTACTAAAAA

AATTATAATATATTAGTAACAAAAAAATTTGGGGAGGGAATGTTATTTTTAAAAAAGATGT

TTTATTTATATTTAAGTGTTTTATTGAAATTCCTCGTGAGTTTAGCTCAATTGGTATGGAC

AATACATAATATATGCAAAATTCGAGGTTCAAACCTTGAGCACCAAAAAAAAAAGTGTTTT

ATTGAAAAATATCAAGAACTTGAAAACAAATTTCCTTTTTATGAACTAGATATTGTCACAT

AGACAACTTAAGAATTGACATTTTTTTTAACACTATTGTATTTTCTGAACTCGACTTTAAC

TCGAGATCAATGAGTAAACTAAAAAAACTCGTATTATTTTATCTAAGTGTTCTTAGTGTGA

GAAAACATTATAATTGTTTGCTTAGTCTATTTCTCCCTATATTCTCACCCTCATTCTTGGT

TTGCACATAGAAAGGAGAAAAAAAAAATTAGAGAAGAGTTGAATGGAACAGAAAGAAAGAG

CAAGCAAAATAATCACTAAAAAAAATTCCCCTAAAAGAAAATCACTGAAAAATATTATCCA

CCTCTATTTTAAATAATAATAATAATGAAAACAACAATATATTTTTTTATAGATTTTCTCT

ACTAGAGGTAATTTATTTTAAACACTAAATGTCCACACCTCTAACAAAAAAAATGCATAAT

ATTATAATAGATTATTGTAAGAAAGAGTGACAAATGTTGAATTAGTTATGGAATGAAGAGT

GAGAGAGATTATACAAATAAATAAATATAAATATAAATATAAATAAAGAATAGAGAAGAAC

ATAGTACTTGAATGAAGAGATTTGGCTTGTACTTCCATTACACTTGTAAGCTTTCTACTTC

CTTCACATTCCTTCTCTTTCTCTATCTTTCCTTTTATGATATGTGTACTTTGTTATTGCTT

CATTTACTACCGACATGACTCCAACCTATTAGGGCTTTATCTGAATAATGTTAGTTATTTT

CTCTCATTTTCTTTGCTTCTTCTTATTCTTGGAACTTATAAAGGGTCTTGTCAATTCTATC

ATTCTCTTTTGTATACTTACAAATATAAATATTCTCTTTAGCTTCATTTTCATGTCTTCTT

CTACTAATAAGCTATGGAAATTGAAGTTTTTGTTTTCATGTTTATTTTTCACTTCAGCTCC

TCTTTTTTGATGTTGATGCTGAAGATTGAATAAAAACTGAGGTGGTATGTATGAGAACAAG

AACATTTTTCATTCATCTCTGTTATTTGAACATCTAAATTTTAAACAACTATCGCGACAAT

TTTTTAAGTGGTTGAAGTATTTTAAATAGGTGGTTAATTACATTAAGTAGATAGTTAATTG

ATATATCAGGTGTCAAAACTCATTAACCATCAACATAACTTCATTAGCCATGAATTTTTGA

CGTGATTAACCAAATATTGTAGATGCTCAATTACATGTAGATTTGATGGAGTTGTCCACAA

TTTCTCATATATCTATTAACTAATAACCATCTACCGAATATAATTAACCATATATTAGAAT

ATCATTAACCTCTAAAACAGTTGTCGCGAAAGTTGGTTGTACAAAAATCATTTCTATTTTT

TACGCCACTAGTTCTCTTGTTATTTCTCTTTGTTGGAAAGTTGTTGTTTTTAACTTTGAGC

TTAGTCTTATTAATTAACTTGTAATGGTAACAACATTTTTCTATCTTTGTTTGAAGGAAGT

AGTTACTGTGTCAACTTTAGTAATTAATTTGGATCATTTTGGAGGCAAAATATGGCTATGC

AACCTGTTTATTTTAAAGAACATGAAGGAAATGTCCACAATTCTGTTGGACAGTTTTCATC

TGTGACTTCAGCACCATGGTGGAGTAATGCCTATGGATCTCAACCTGTTTATGGAGGAGAC

TCTTGTGGCCAAATGAAACCTTTTTCACTAGAGCTTTCCAACTACATAGACCAACTTGCTC

CGAGTAAGAACTTAGTTCGAGGAGTTGAACAATTGTTTGATAAAGGGCATACAAACCAATT

CACCATCTTTCCAGGTACTTGTTATTCAATATAATTCCGGTTTTGAATGAATTGATTTTTG

TTAAGTTGGTTCTGGATAAACGTGCGTTAAATATAATATGATTTATGTTTGGATACGTTTA

TGTAAAAGTGAGTTGAACATAAATTGGAGACTAAATATCAATTGCAGAGGCAAAAGCTTCA

AATTCTAATTTCAAGTTAGAATCAAATCAATTCTACTCGTAAGCATCTAAATATTTCAAAA

CCAATTCTACATCTCTAGAACTAAATTTGCATTCAAACATTTTCTGCTACAGATGATTGTA

AGATGTCAGTTGATACACCAAATCATCAAGCAACCTTATCCCTGCAATCACCATTTGCTGC

CGAGCCACTTAATCGATTTGAGCTAGGTTTTAACCAGTCTATGGTAAATCTTCTTATTAAA

GCAATCCCTTTGATCATGTTTTAGTATTTTACTTTTGATGTACATAAATTCATTATGTTTT

TATTATCCTTTCCATGCTAGATCTGTGCAAAATATCCGTATATAGATCAATTTTACGGGCT

CTTCTCGACTTATGGACCTCAAATCTCGGTTTGTCTTCACCTCGGCTATTTTATTAATCTT

TGTATATATCATGCAATGTCTATAAGTACTTGAATGTGAATTTGTTTTATTTACAGTGTGT

ATGCTTACATGAGAAAGCCAGAAATCTGTTTACAACTATCTTGTTAGTATTCTTCTAAATC

TTTCAAATGTAGAGTTGATTATGAAATTTGTTGTTAATGAGTTTTCGGCTTTATGAATGAA

TTGTAAGACAAGTAATGAAACAAAAACTAGAAGAAAATTGAATTATATGATGCAATCTGTG

TTGAAGTTTTTGCATGTATTCAAGTTCTGCTTTTAGTTACTATTTGTTTATCTGCTGCAAT

ATTTCATCAATTATTATTCATGTATGTACCAATATTTAATAAATTAGGGGCGAATTATGCT

TCCGCTTAGCATGACATCTGACGACGGACCAACATACGTGAATGCTAAGCAATACCATGGA

ATCATCAGACGCAGGCATTCTCGTGCCAAAGCTGTGCTTCAGAATAAATTGATAAAGCGTA

ACAAGGTATGAAACTGAGTATTTTTCCTCACAACAATGTTCGAAAACTTGTGAAATAGTAT

ATTTTTCCTTTTACCCTTTTTATACCTATATTAAGGTTTTGTATTTGTCTTGCAGCCATAT

ATGCACGAATCGCGTCATCTACATGCAATGCGTAGACCAAGAGGATGCGGTGGTCGTTTCT

TGAACACAAAAGTTTCTGCTAATGGAAACGGTAAAAGCGGGAGTGAAGAGAACGGAAACAT

TGGTGGCCTACAGCTGCAGTCCAGTGGTTCTCAGAGTTCCGAAGTCTTACAATCTGAGGTT

GGAACTTTAAATTCGTCGAAGGAGACAAACGGAGGCAGTCCAAATGTCTCGGGGTCAGAGG

TGACTAGCATGTATACACAGGGAGGTCTTGATAGCTTTACTGTCAATCATATCGGATCTAC

TGTCCACTCTTTGGGAGACATGATCGATACTGGACACGGTATCGTCATGCCAACAAAATGG

TTTGCAGCAGCTGGCAGACAGCTGCTGGAACCATAAGTTTCGATTCAGAAAGGAAACAAGT

GGGTTTGGTACAATGTGAAATATTTTGCACCAAACTCATCCTTTCCGAGACCAGATGAAGA

AGCTATGTTTCAGTTTGTTGTGTTTACTACGACAAATTTAGTTTCGGAAGACTACTTTTCC

ATCTGGTGCTCAGGCAACTCATTCTTGGCTTATTCTCAGGAAACTCATCCTTGGCTCGTAA

TATTTAGTAGTATTGTCATTGTCTTCCCGCGCAGGCTTGCCGTGGCATGGTAGGCATGCTA

ATGACTTTGGTATTTTCATGCAGTTATAACTATGATGTGTCTTTGTTTGTTGTTAAAATAA

AAAACATGAACTCTAGCTAGGTGCATGTGTGTGTTTTTAATCTTGTCTACTAAGTTTGGTG

TTTTGTAATGGATTTCTGACTTTATGGAGCAATGTATTGTAACTCTACTAAGAAGTGTAAC

ATTTTATTTCTCCCTCTCTAAGGATTGTATAAGAACCTCTTATTTTCAGACTCTACTTAAT

CCTATTTTCTATGTCTGTATGATTTTTATATTTCTAGGACAATCAAATTGGCTTGTAGAAG

CTCAAAAGCATGCTCACAAAGTAGGTACTTATGTAGGGAACTTCGTACTCTAATAATAACT

GGTTATTAACGTTATAATTAAATGCAAAATTTGATAAGTAGTAGGGTTTGGTAAGTAATAT

AACAACACCATGTTGGCTTGGGATTCTGAATTGTGTTATTAGAGTACAACTCTTAAATAAT

GATCGTGATGTAAATAATTTAAATTTGAAGTTACTAGATAAAAAGATAACATTATGTAATA

TATTATCAAATATATACTAAAAATATAATTTTCATCTATCTCAATTGAAAAAATATCCGGG

TTTTGAGTCTATTGTCAATGACGA

>HM042

AAAAAAATAAAGGTTCTATACTCATTAAACAAAGTGCCAAATTCAGAGATACTACTTCCT

TTGGATAATTTGTATCGTCACCAACGATCTTTGAGTTATTTCAAAAATCACATTCTCAATT

TTGTGGTCCAACATTCACAAAATGGTTTGTCGGGCTTTAAGTCTAGTAATTGTGAAACATG

CATATGCATGAGGTAATTAATTGATAATCTGAAAAGGTTAAATAGTTAATCAATTGTACTT

TGGTTATTTTTAATTTGTTCTCTATAATTTTTCAGTCAAAATTGAATTTGCTTTAACCATT

TAGTGTAGTAGTATGATATTACTTGCTCTACATTCTTGGATCTAAAAGTCTAATTAGTATC

GTATAAAAAATATTCTAGTTAATTAAGATACTTTTGTTCATTCTAATTATTCTTGGATTAC

TTGTGAAACTTTTAGGGATGAACAAGAAAGTCCACTAAAGAGGCTTCTTTTTGCTAGGAGT

CACTAGTTAGTTTTTTTTTTTTTTGACAAAAGTTAACCATTTTTTTTTATCGCATGGTAGG

AAAAAAAAAATAGAGATTTCCTTTGGTACTCATAAAAAAAAAAGTTGGATGGATAATGGAT

TAATCATAACTTATTTAACATAAAATGAATTAAGAGAACATTTGATATTTGGGTTTATTTT

GTAACATAAATATTAATATCAATGTTTGAAAGAGTTTTTAACAATAATTTGTAAAAGAAAG

CATAATATATCTATCAAAATAATTGTAAAAGAAAAAATTTCTTTTGGTGGTATGACATGGA

AAAATAATTAATTCTTATTAGATGAAAGTGTAAAATTAATTTGCATTAACAAAGCATATTT

CATTAAACTCCTTTTAGTTTTTGTTGGAAAAAATAAATTTATTGTTACTCAAAAAATAAAA

GTGTACAATTAATATATTTACTCAAAAGTCCCCTAAATGGGATGTAATTATCAAGGGATGT

GAGCGGTTTAACTTTAAGTAATGAATCAACTGTTGCATAATCACTTTGAAAGAAACTTGAG

ATTCTTCAATTAGCAAAATCTTGAATAGAATAATGAGATTATTCCTAATGCTAATGATACC

CACCTCTCCTATAAGTTGATGGGGCAGAACAAATTATGCACTCAGGACAACTTTTCTCAAA

TTTTGAAAACTTACCACACGAAGAACACACTTCCACTTGCACAAGTAAGAATATAGTATAA

CAATAAATTTACCACTCATAGCTCAATGGATATTAAAATATTGCTAATCTTTTATAGACAG

ATGTTGGCAAATTAGTATAAATTAGTTATTATATTAGTTTTTAAGGTTCAATCTACTTTTT

AAAACTCTTATTAAGTTGTTTAGCTTGCCAACCAAAGACTAAATTATCTTTATTTGTTAGG

AAATAAAAGCACTTATTTTATTTTCTCCATATTTGTTTCCTCTTTGTTCCATTACTAAAAA

AATTATAATATATTAGTAACAAAAAAATTTGGGGAGGGAATGTTATTTTTAAAAAAGATGT

TTTATTTATATTTAAGTGTTTTATTGAAATTCCTCGTGAGTTTAGCTCAATTGGTATGGAC

AATACATAATATATGCAAAATTCGAGGTTCAAACCTTGAGCACCAAAAAAAAAAGTGTTTT

ATTGAAAAATATCAAGAACTTGAAAACAAATTTCCTTTTTATGAACTAGATATTGTCACAT

AGACAACTTAAGAATTGACATTTTTTTTAACACTATTGTATTTTCTGAACTCGACTTTAAC

TCGAGATCAATGAGTAAACTAAAAAAACTCGTATTATTTTATCTAAGTGTTCTTAGTGTGA

GAAAACATTATAATTGTTTGCTTAGTCTATTTCTCCCTATATTCTCACCCTCATTCTTGGT

TTGCACATAGAAAGGAGAAAAAAAAAATTAGAGAAGAGTTGAATGGAACAGAAAGAAAGAG

CAAGCAAAATAATCACTAAAAAAAATTCCCCTAAAAGAAAATCACTGAAAAATATTATCCA

CCTCTATTTTAAATAATAATAATAATGAAAACAACAATATATTTTTTTATAGATTTTCTCT

ACTAGAGGTAATTTATTTTAAACACTAAATGTCCACACCTCTAACAAAAAAAATGCATAAT

ATTATAATAGATTATTGTAAGAAAGAGTGACAAATGTTGAATTAGTTATGGAATGAAGAGT

GAGAGAGATTATACAAATAAATAAATATAAATATAAATATAAATAAAGAATAGAGAAGAAC

ATAGTACTTGAATGAAGAGATTTGGCTTGTACTTCCATTACACTTGTAAGCTTTCTACTTC

CTTCACATTCCTTCTCTTTCTCTATCTTTCCTTTTATGATATGTGTACTTTGTTATTGCTT

CATTTACTACCGACATGACTCCAACCTATTAGGGCTTTATCTGAATAATGTTAGTTATTTT

CTCTCATTTTCTTTGCTTCTTCTTATTCTTGGAACTTATAAAGGGTCTTGTCAATTCTATC

ATTCTCTTTTGTATACTTACAAATATAAATATTCTCTTTAGCTTCATTTTCATGTCTTCTT

CTACTAATAAGCTATGGAAATTGAAGTTTTTGTTTTCATGTTTATTTTTCACTTCAGCTCC

TCTTTTTTGATGTTGATGCTGAAGATTGAATAAAAACTGAGGTGGTATGTATGAGAACAAG

AACATTTTTCATTCATCTCTGTTATTTGAACATCTAAATTTTAAACAACTATCGCGACAAT

TTTTTAAGTGGTTGAAGTATTTTAAATAGGTGGTTAATTACATTAAGTAGATAGTTAATTG

ATATATCAGGTGTCAAAACTCATTAACCATCAACATAACTTCATTAGCCATGAATTTTTGA

CGTGATTAACCAAATATTGTAGATGCTCAATTACATGTAGATTTGATGGAGTTGTCCACAA

TTTCTCATATATCTATTAACTAATAACCATCTACCGAATATAATTAACCATATATTAGAAT

ATCATTAACCTCTAAAACAGTTGTCGCGAAAGTTGGTTGTACAAAAATCATTTCTATTTTT

TACGCCACTAGTTCTCTTGTTATTTCTCTTTGTTGGAAAGTTGTTGTTTTTAACTTTGAGC

TTAGTCTTATTAATTAACTTGTAATGGTAACAACATTTTTCTATCTTTGTTTGAAGGAAGT

AGTTACTGTGTCAACTTTAGTAATTAATTTGGATCATTTTGGAGGCAAAATATGGCTATGC

AACCTGTTTATTTTAAAGAACATGAAGGAAATGTCCACAATTCTGTTGGACAGTTTTCATC

TGTGACTTCAGCACCATGGTGGAGTAATGCCTATGGATCTCAACCTGTTTATGGAGGAGAC

TCTTGTGGCCAAATGAAACCTTTTTCACTAGAGCTTTCCAACTACATAGACCAACTTGCTC

CGAGTAAGAACTTAGTTCGAGGAGTTGAACAATTGTTTGATAAAGGGCATACAAACCAATT

CACCATCTTTCCAGGTACTTGTTATTCAATATAATTCCGGTTTTGAATGAATTGATTTTTG

TTAAGTTGGTTCTGGATAAACGTGCGTTAAATATAATATGATTTATGTTTGGATACGTTTA

TGTAAAAGTGAGTTGAACATAAATTGGAGACTAAATATCAATTGCAGAGGCAAAAGCTTCA

AATTCTAATTTCAAGTTAGAATCAAATCAATTCTACTCGTAAGCATCTAAATATTTCAAAA

CCAATTCTACATCTCTAGAACTAAATTTGCATTCAAACATTTTCTGCTACAGATGATTGTA

AGATGTCAGTTGATACACCAAATCATCAAGCAACCTTATCCCTGCAATCACCATTTGCTGC

CGAGCCACTTAATCGATTTGAGCTAGGTTTTAACCAGTCTATGGTAAATCTTCTTATTAAA

GCAATCCCTTTGATCATGTTTTAGTATTTTACTTTTGATGTACATAAATTCATTATGTTTT

TATTATCCTTTCCATGCTAGATCTGTGCAAAATATCCGTATATAGATCAATTTTACGGGCT

CTTCTCGACTTATGGACCTCAAATCTCGGTTTGTCTTCACCTCGGCTATTTTATTAATCTT

TGTATATATCATGCAATGTCTATAAGTACTTGAATGTGAATTTGTTTTATTTACAGTGTGT

ATGCTTACATGAGAAAGCCAGAAATCTGTTTACAACTATCTTGTTAGTATTCTTCTAAATC

TTTCAAATGTAGAGTTGATTATGAAATTTGTTGTTAATGAGTTTTCGGCTTTATGAATGAA

TTGTAAGACAAGTAATGAAACAAAAACTAGAAGAAAATTGAATTATATGATGCAATCTGTG

TTGAAGTTTTTGCATGTATTCAAGTTCTGCTTTTAGTTACTATTTGTTTATCTGCTGCAAT

ATTTCATCAATTATTATTCATGTATGTACCAATATTTAATAAATTAGGGGCGAATTATGCT

TCCGCTTAGCATGACATCTGACGACGGACCAACATACGTGAATGCTAAGCAATACCATGGA

ATCATCAGACGCAGGCATTCTCGTGCCAAAGCTGTGCTTCAGAATAAATTGATAAAGCGTA

ACAAGGTATGAAACTGAGTATTTTTCCTCACAACAATGTTCGAAAACTTGTGAAATAGTAT

ATTTTTCCTTTTACCCTTTTTATACCTATATTAAGGTTTTGTATTTGTCTTGCAGCCATAT

ATGCACGAATCGCGTCATCTACATGCAATGCGTAGACCAAGAGGATGCGGTGGTCGTTTCT

TGAACACAAAAGTTTCTGCTAATGGAAACGGTAAAAGCGGGAGTGAAGAGAACGGAAACAT

TGGTGGCCTACAGCTGCAGTCCAGTGGTTCTCAGAGTTCCGAAGTCTTACAATCTGAGGTT

GGAACTTTAAATTCGTCGAAGGAGACAAACGGAGGCAGTCCAAATGTCTCGGGGTCAGAGG

TGACTAGCATGTATACACAGGGAGGTCTTGATAGCTTTACTGTCAATCATATCGGATCTAC

TGTCCACTCTTTGGGAGACATGATCGATACTGGACACGGTATCGTCATGCCAACAAAATGG

TTTGCAGCAGCTGGCAGACAGCTGCTGGAACCATAAGTTTCGATTCAGAAAGGAAACAAGT

GGGTTTGGTACAATGTGAAATATTTTGCACCAAACTCATCCTTTCCGAGACCAGATGAAGA

AGCTATGTTTCAGTTTGTTGTGTTTACTACGACAAATTTAGTTTCGGAAGACTACTTTTCC

ATCTGGTGCTCAGGCAACTCATTCTTGGCTTATTCTCAGGAAACTCATCCTTGGCTCGTAA

TATTTAGTAGTATTGTCATTGTCTTTCCGCGCAGGCTTGCCGTGGCATGGTAGGCATGCTA

ATGACTTTGGTATTTTCATGCAGTTATAACTATGATGTGTCTTTGTTTGTTGTTAAAATAA

AAAACATGAACTCTAGCTAGGTGCATGTGTGTGTTTTTAATCTTGTCTACTAAGTTTGGTG

TTTTGTAATGGATTTCTGACTTTATGGAGCAATGTATTGTAACTCTACTAAGAAGTGTAAC

ATTTTATTTCTCCCTCTCTAAGGATTGTATAAGAACCTCTTATTTTCAGACTCTACTTAAT

CCTATTTTCTATGTCTGTATGATTTTTATATTTCTAGGACAATCAAATTGGCTTGTAGAAG

CTCAAAAGCATGCTCACAAAGTAGGTACTTATGTAGGGAACTTCGTACTCTAATAATAACT

GGTTATTAACGTTATAATTAAATGCAAAATTTGATAAGTAGTAGGGTTTGGTAAGTAATAT

AACAACACCATGTTGGCTTGGGATTCTGAATTGTGTTATTAGAGTACAACTCTTAAATAAT

GATCGTGATGTAAATAATTTAAATTTGAAGTTACTAGATAAAAAGATAACATTATGTAATA

TATTATCAAATATATACTAAAAATATAATTTTCATCTATCTCAATTGAAAAAATATCCGGG

TTTTGAGTCTATTGTCAATGACGA

>HM043

AAAAAAATAAAGGTTCTATACTCATTAAACAAAGTGCCAAATTCAGAGATACTACTTCCT

TTGGATAATTTGTATCGTCACCAACGATCTTTGAGTTATTTCAAAAATCACATTCTCAATT

TTGTGGTCCAACATTCACAAAATGGTTTGTCGGGCTTTAAGTCTAGTAATTGTGAAACATG

CATATGCATGAGGTAATTAATTGATAATCTGAAAAGGTTAAATAGTTAATCAATTGTACTT

TGGTTATTTTTAATTTGTTCTCTATAATTTTTCAGTCAAAATTGAATTTGCTTTAACCATT

TAGTGTAGTAGTATGATATTACTTGCTCTACATTCTTGGATCTAAAAGTCTAATTAGTATC

GTATAAAAAATATTCTAGTTAATTAAGATACTTTTGTTCATTCTAATTATTCTTGGATTAC

TTGTGAAACTTTTAGGGATGAACAAGAAAGTCCACTAAAGAGGCTTCTTTTTGCTAGGAGT

CACTAGTTAGTTTTTTTTTTTTTTGACAAAAGTTAACCATTTTTTTTTATCGCATGGTAGG

AAAAAAAAAATAGAGATTTCCTTTGGTACTCATAAAAAAAAAAGTTGGATGGATAATGGAT

TAATCATAACTTATTTAACATAAAATGAATTAAGAGAACATTTGATATTTGGGTTTATTTT

GTAACATAAATATTAATATCAATGTTTGAAAGAGTTTTTAACAATAATTTGTAAAAGAAAG

CATAATATATCTATCAAAATAATTGTAAAAGAAAAAATTTCTTTTGGTGGTATGACATGGA

AAAATAATTAATTCTTATTAGATGAAAGTGTAAAATTAATTTGCATTAACAAAGCATATTT

CATTAAACTCCTTTTAGTTTTTGTTGGAAAAAATAAATTTATTGTTACTCAAAAAATAAAA

GTGTACAATTAATATATTTACTCAAAAGTCCCCTAAATGGGATGTAATTATCAAGGGATGT

GAGCGGTTTAACTTTAAGTAATGAATCAACTGTTGCATAATCACTTTGAAAGAAACTTGAG

ATTCTTCAATTAGCAAAATCTTGAATAGAATAATGAGATTATTCCTAATGCTAATGATACC

CACCTCTCCTATAAGTTGATGGGGCAGAACAAATTATGCACTCAGGACAACTTTTCTCAAA

TTTTGAAAACTTACCACACGAAGAACACACTTCCACTTGCACAAGTAAGAATATAGTATAA

CAATAAATTTACCACTCATAGCTCAATGGATATTAAAATATTGCTAATCTTTTATAGACAG

ATGTTGGCAAATTAGTATAAATTAGTTATTATATTAGTTTTTAAGGTTCAATCTACTTTTT

AAAACTCTTATTAAGTTGTTTAGCTTGCCAACCAAAGACTAAATTATCTTTATTTGTTAGG

AAATAAAAGCACTTATTTTATTTTCTCCATATTTGTTTCCTCTTTGTTCCATTACTAAAAA

AATTATAATATATTAGTAACAAAAAAATTTGGGGAGGGAATGTTATTTTTAAAAAAGATGT

TTTATTTATATTTAAGTGTTTTATTGAAATTCCTCGTGAGTTTAGCTCAATTGGTATGGAC

AATACATAATATATGCAAAATTCGAGGTTCAAACCTTGAGCACCAAAAAAAAAAGTGTTTT

ATTGAAAAATATCAAGAACTTGAAAACAAATTTCCTTTTTATGAACTAGATATTGTCACAT

AGACAACTTAAGAATTGACATTTTTTTTAACACTATTGTATTTTCTGAACTCGACTTTAAC

TCGAGATCAATGAGTAAACTAAAAAAACTCGTATTATTTTATCTAAGTGTTCTTAGTGTGA

GAAAACATTATAATTGTTTGCTTAGTCTATTTCTCCCTATATTCTCACCCTCATTCTTGGT

TTGCACATAGAAAGGAGAAAAAAAAAATTAGAGAAGAGTTGAATGGAACAGAAAGAAAGAG

CAAGCAAAATAATCACTAAAAAAAATTCCCCTAAAAGAAAATCACTGAAAAATATTATCCA

CCTCTATTTTAAATAATAATAATAATGAAAACAACAATATATTTTTTTATAGATTTTCTCT

ACTAGAGGTAATTTATTTTAAACACTAAATGTCCACACCTCTAACAAAAAAAATGCATAAT

ATTATAATAGATTATTGTAAGAAAGAGTGACAAATGTTGAATTAGTTATGGAATGAAGAGT

GAGAGAGATTATACAAATAAATAAATATAAATATAAATATAAATAAAGAATAGAGAAGAAC

ATAGTACTTGAATGAAGAGATTTGGCTTGTACTTCCATTACACTTGTAAGCTTTCTACTTC

CTTCACATTCCTTCTCTTTCTCTATCTTTCCTTTTATGATATGTGTACTTTGTTATTGCTT

CATTTACTACCGACATGACTCCAACCTATTAGGGCTTTATCTGAATAATGTTAGTTATTTT

CTCTCATTTTCTTTGCTTCTTCTTATTCTTGGAACTTATAAAGGGTCTTGTCAATTCTATC

ATTCTCTTTTGTATACTTACAAATATAAATATTCTCTTTAGCTTCATTTTCATGTCTTCTT

CTACTAATAAGCTATGGAAATTGAAGTTTTTGTTTTCATGTTTATTTTTCACTTCAGCTCC

TCTTTTTTGATGTTGATGCTGAAGATTGAATAAAAACTGAGGTGGTATGTATGAGAACAAG

AACATTTTTCATTCATCTCTGTTATTTGAACATCTAAATTTTAAACAACTATCGCGACAAT

TTTTTAAGTGGTTGAAGTATTTTAAATAGGTGGTTAATTACATTAAGTAGATAGTTAATTG

ATATATCAGGTGTCAAAACTCATTAACCATCAACATAACTTCATTAGCCATGAATTTTTGA

CGTGATTAACCAAATATTGTAGATGCTCAATTACATGTAGATTTGATGGAGTTGTCCACAA

TTTCTCATATATCTATTAACTAATAACCATCTACCGAATATAATTAACCATATATTAGAAT

ATCATTAACCTCTAAAACAGTTGTCGCGAAAGTTGGTTGTACAAAAATCATTTCTATTTTT

TACGCCACTAGTTCTCTTGTTATTTCTCTTTGTTGGAAAGTTGTTGTTTTTAACTTTGAGC

TTAGTCTTATTAATTAACTTGTAATGGTAACAACATTTTTCTATCTTTGTTTGAAGGAAGT

AGTTACTGTGTCAACTTTAGTAATTAATTTGGATCATTTTGGAGGCAAAATATGGCTATGC

AACCTGTTTATTTTAAAGAACATGAAGGAAATGTCCACAATTCTGTTGGACAGTTTTCATC

TGTGACTTCAGCACCATGGTGGAGTAATGCCTATGGATCTCAACCTGTTTATGGAGGAGAC

TCTTGTGGCCAAATGAAACCTTTTTCACTAGAGCTTTCCAACTACATAGACCAACTTGCTC

CGAGTAAGAACTTAGTTCGAGGAGTTGAACAATTGTTTGATAAAGGGCATACAAACCAATT

CACCATCTTTCCAGGTACTTGTTATTCAATATAATTCCGGTTTTGAATGAATTGATTTTTG

TTAAGTTGGTTCTGGATAAACGTGCGTTAAATATAATATGATTTATGTTTGGATACGTTTA

TGTAAAAGTGAGTTGAACATAAATTGGAGACTAAATATCAATTGCAGAGGCAAAAGCTTCA

AATTCTAATTTCAAGTTAGAATCAAATCAATTCTACTCGTAAGCATCTAAATATTTCAAAA

CCAATTCTACATCTCTAGAACTAAATTTGCATTCAAACATTTTCTGCTACAGATGATTGTA

AGATGTCAGTTGATACACCAAATCATCAAGCAACCTTATCCCTGCAATCACCATTTGCTGC

CGAGCCACTTAATCGATTTGAGCTAGGTTTTAACCAGTCTATGGTAAATCTTCTTATTAAA

GCAATCCCTTTGATCATGTTTTAGTATTTTACTTTTGATGTACATAAATTCATTATGTTTT

TATTATCCTTTCCATGCTAGATCTGTGCAAAATATCCGTATATAGATCAATTTTACGGGCT

CTTCTCGACTTATGGACCTCAAATCTCGGTTTGTCTTCACCTCGGCTATTTTATTAATCTT

TGTATATATCATGCAATGTCTATAAGTACTTGAATGTGAATTTGTTTTATTTACAGTGTGT

ATGCTTACATGAGAAAGCCAGAAATCTGTTTACAACTATCTTGTTAGTATTCTTCTAAATC

TTTCAAATGTAGAGTTGATTATGAAATTTGTTGTTAATGAGTTTTCGGCTTTATGAATGAA

TTGTAAGACAAGTAATGAAACAAAAACTAGAAGAAAATTGAATTATATGATGCAATCTGTG

TTGAAGTTTTTGCATGTATTCAAGTTCTGCTTTTAGTTACTATTTGTTTATCTGCTGCAAT

ATTTCATCAATTATTATTCATGTATGTACCAATATTTAATAAATTAGGGGCGAATTATGCT

TCCGCTTAGCATGACATCTGACGACGGACCAACATACGTGAATGCTAAGCAATACCATGGA

ATCATCAGACGCAGGCATTCTCGTGCCAAAGCTGTGCTTCAGAATAAATTGATAAAGCGTA

ACAAGGTATGAAACTGAGTATTTTTCCTCACAACAATGTTCGAAAACTTGTGAAATAGTAT

ATTTTTCCTTTTACCCTTTTTATACCTATATTAAGGTTTTGTATTTGTCTTGCAGCCATAT

ATGCACGAATCGCGTCATCTACATGCAATGCGTAGACCAAGAGGATGCGGTGGTCGTTTCT

TGAACACAAAAGTTTCTGCTAATGGAAACGGTAAAAGCGGGAGTGAAGAGAACGGAAACAT

TGGTGGCCTACAGCTGCAGTCCAGTGGTTCTCAGAGTTCCGAAGTCTTACAATCTGAGGTT

GGAACTTTAAATTCGTCGAAGGAGACAAACGGAGGCAGTCCAAATGTCTCGGGGTCAGAGG

TGACTAGCATGTATACACAGGGAGGTCTTGATAGCTTTACTGTCAATCATATCGGATCTAC

TGTCCACTCTTTGGGAGACATGATCGATACTGGACACGGTATCGTCATGCCAACAAAATGG

TTTGCAGCAGCTGGCAGACAGCTGCTGGAACCATAAGTTTCGATTCAGAAAGGAAACAAGT

GGGTTTGGTACAATGTGAAATATTTTGCACCAAACTCATCCTTTCCGAGACCAGATGAAGA

AGCTATGTTTCAGTTTGTTGTGTTTACTACGACAAATTTAGTTTCGGAAGACTACTTTTCC

ATCTGGTGCTCAGGCAACTCATTCTTGGCTTATTCTCAGGAAACTCATCCTTGGCTCGTAA

TATTTAGTAGTATTGTCATTGTCTTTCCGCGCAGGCTTGCCGTGGCATGGTAGGCATGCTA

ATGACTTTGGTATTTTCATGCAGTTATAACTATGATGTGTCTTTGTTTGTTGTTAAAATAA

AAAACATGAACTCTAGCTAGGTGCATGTGTGTGTTTTTAATCTTGTCTACTAAGTTTGGTG

TTTTGTAATGGATTTCTGACTTTATGGAGCAATGTATTGTAACTCTACTAAGAAGTGTAAC

ATTTTATTTCTCCCTCTCTAAGGATTGTATAAGAACCTCTTATTTTCAGACTCTACTTAAT

CCTATTTTCTATGTCTGTATGATTTTTATATTTCTAGGACAATCAAATTGGCTTGTAGAAG

CTCAAAAGCATGCTCACAAAGTAGGTACTTATGTAGGGAACTTCGTACTCTAATAATAACT

GGTTATTAACGTTATAATTAAATGCAAAATTTGATAAGTAGTAGGGTTTGGTAAGTAATAT

AACAACACCATGTTGGCTTGGGATTCTGAATTGTGTTATTAGAGTACAACTCTTAAATAAT

GATCGTGATGTAAATAATTTAAATTTGAAGTTACTAGATAAAAAGATAACATTATGTAATA

TATTATCAAATATATACTAAAAATATAATTTTCATCTATCTCAATTGAAAAAATATCCGGG

TTTTGAGTCTATTGTCAATGACGA

>HM044

AAAAAAATAAAGGTTCTATACTCATTAAACAAAGTGCCAAATTCAGAGATACTACTTCCT

TTGGATAATTTGTATCGTCACCAACGATCTTTGAGTTATTTCAAAAATCACATTCTCAATT

TTGTGGTCCAACATTCACAAAATGGTTTGTCGGGCTTTAAGTCTAGTAATTGTGAAACATG

CATATGCATGAGGTAATTAATTGATAATCTGAAAAGGTTAAATAGTTAATCAATTGTACTT

TGGTTATTTTTAATTTGTTCTCTATAATTTTTCAGTCAAAATTGAATTTGCTTTAACCATT

TAGTGTAGTAGTATGATATTACTTGCTCTACATTCTTGGATCTAAAAGTCTAATTAGTATC

GTATAAAAAATATTCTAGTTAATTAAGATACTTTTGTTCATTCTAATTATTCTTGGATTAC

TTGTGAAACTTTTAGGGATGAACAAGAAAGTCCACTAAAGAGGCTTCTTTTTGCTAGGAGT

CACTAGTTAGTTTTTTTTTTTTTTGACAAAAGTTAACCATTTTTTTTTATCGCATGGTAGG

AAAAAAAAAATAGAGATTTCCTTTGGTACTCATAAAAAAAAAAGTTGGATGGATAATGGAT

TAATCATAACTTATTTAACATAAAATGAATTAAGAGAACATTTGATATTTGGGTTTATTTT

GTAACATAAATATTAATATCAATGTTTGAAAGAGTTTTTAACAATAATTTGTAAAAGAAAG

CATAATATATCTATCAAAATAATTGTAAAAGAAAAAATTTCTTTTGGTGGTATGACATGGA

AAAATAATTAATTCTTATTAGATGAAAGTGTAAAATTAATTTGCATTAACAAAGCATATTT

CATTAAACTCCTTTTAGTTTTTGTTGGAAAAAATAAATTTATTGTTACTCAAAAAATAAAA

GTGTACAATTAATATATTTACTCAAAAGTCCCCTAAATGGGATGTAATTATCAAGGGATGT

GAGCGGTTTAACTTTAAGTAATGAATCAACTGTTGCATAATCACTTTGAAAGAAACTTGAG

ATTCTTCAATTAGCAAAATCTTGAATAGAATAATGAGATTATTCCTAATGCTAATGATACC

CACCTCTCCTATAAGTTGATGGGGCAGAACAAATTATGCACTCAGGACAACTTTTCTCAAA

TTTTGAAAACTTACCACACGAAGAACACACTTCCACTTGCACAAGTAAGAATATAGTATAA

CAATAAATTTACCACTCATAGCTCAATGGATATTAAAATATTGCTAATCTTTTATAGACAG

ATGTTGGCAAATTAGTATAAATTAGTTATTATATTAGTTTTTAAGGTTCAATCTACTTTTT

AAAACTCTTATTAAGTTGTTTAGCTTGCCAACCAAAGACTAAATTATCTTTATTTGTTAGG

AAATAAAAGCACTTATTTTATTTTCTCCATATTTGTTTCCTCTTTGTTCCATTACTAAAAA

AATTATAATATATTAGTAACAAAAAAATTTGGGGAGGGAATGTTATTTTTAAAAAAGATGT

TTTATTTATATTTAAGTGTTTTATTGAAATTCCTCGTGAGTTTAGCTCAATTGGTATGGAC

AATACATAATATATGCAAAATTCGAGGTTCAAACCTTGAGCACCAAAAAAAAAAGTGTTTT

ATTGAAAAATATCAAGAACTTGAAAACAAATTTCCTTTTTATGAACTAGATATTGTCACAT

AGACAACTTAAGAATTGACATTTTTTTTAACACTATTGTATTTTCTGAACTCGACTTTAAC

TCGAGATCAATGAGTAAACTAAAAAAACTCGTATTATTTTATCTAAGTGTTCTTAGTGTGA

GAAAACATTATAATTGTTTGCTTAGTCTATTTCTCCCTATATTCTCACCCTCATTCTTGGT

TTGCACATAGAAAGGAGAAAAAAAAAATTAGAGAAGAGTTGAATGGAACAGAAAGAAAGAG

CAAGCAAAATAATCACTAAAAAAAATTCCCCTAAAAGAAAATCACTGAAAAATATTATCCA

CCTCTATTTTAAATAATAATAATAATGAAAACAACAATATATTTTTTTATAGATTTTCTCT

ACTAGAGGTAATTTATTTTAAACACTAAATGTCCACACCTCTAACAAAAAAAATGCATAAT

ATTATAATAGATTATTGTAAGAAAGAGTGACAAATGTTGAATTAGTTATGGAATGAAGAGT

GAGAGAGATTATACAAATAAATAAATATAAATATAAATATAAATAAAGAATAGAGAAGAAC

ATAGTACTTGAATGAAGAGATTTGGCTTGTACTTCCATTACACTTGTAAGCTTTCTACTTC

CTTCACATTCCTTCTCTTTCTCTATCTTTCCTTTTATGATATGTGTACTTTGTTATTGCTT

CATTTACTACCGACATGACTCCAACCTATTAGGGCTTTATCTGAATAATGTTAGTTATTTT

CTCTCATTTTCTTTGCTTCTTCTTATTCTTGGAACTTATAAAGGGTCTTGTCAATTCTATC

ATTCTCTTTTGTATACTTACAAATATAAATATTCTCTTTAGCTTCATTTTCATGTCTTCTT

CTACTAATAAGCTATGGAAATTGAAGTTTTTGTTTTCATGTTTATTTTTCACTTCAGCTCC

TCTTTTTTGATGTTGATGCTGAAGATTGAATAAAAACTGAGGTGGTATGTATGAGAACAAG

AACATTTTTCATTCATCTCTGTTATTTGAACATCTAAATTTTAAACAACTATCGCGACAAT

TTTTTAAGTGGTTGAAGTATTTTAAATAGGTGGTTAATTACATTAAGTAGATAGTTAATTG

ATATATCAGGTGTCAAAACTCATTAACCATCAACATAACTTCATTAGCCATGAATTTTTGA

CGTGATTAACCAAATATTGTAGATGCTCAATTACATGTAGATTTGATGGAGTTGTCCACAA

TTTCTCATATATCTATTAACTAATAACCATCTACCGAATATAATTAACCATATATTAGAAT

ATCATTAACCTCTAAAACAGTTGTCGCGAAAGTTGGTTGTACAAAAATCATTTCTATTTTT

TACGCCACTAGTTCTCTTGTTATTTCTCTTTGTTGGAAAGTTGTTGTTTTTAACTTTGAGC

TTAGTCTTATTAATTAACTTGTAATGGTAACAACATTTTTCTATCTTTGTTTGAAGGAAGT

AGTTACTGTGTCAACTTTAGTAATTAATTTGGATCATTTTGGAGGCAAAATATGGCTATGC

AACCTGTTTATTTTAAAGAACATGAAGGAAATGTCCACAATTCTGTTGGACAGTTTTCATC

TGTGACTTCAGCACCATGGTGGAGTAATGCCTATGGATCTCAACCTGTTTATGGAGGAGAC

TCTTGTGGCCAAATGAAACCTTTTTCACTAGAGCTTTCCAACTACATAGACCAACTTGCTC

CGAGTAAGAACTTAGTTCGAGGAGTTGAACAATTGTTTGATAAAGGGCATACAAACCAATT

CACCATCTTTCCAGGTACTTGTTATTCAATATAATTCCGGTTTTGAATGAATTGATTTTTG

TTAAGTTGGTTCTGGATAAACGTGCGTTAAATATAATATGATTTATGTTTGGATACGTTTA

TGTAAAAGTGAGTTGAACATAAATTGGAGACTAAATATCAATTGCAGAGGCAAAAGCTTCA

AATTCTAATTTCAAGTTAGAATCAAATCAATTCTACTCGTAAGCATCTAAATATTTCAAAA

CCAATTCTACATCTCTAGAACTAAATTTGCATTCAAACATTTTCTGCTACAGATGATTGTA

AGATGTCAGTTGATACACCAAATCATCAAGCAACCTTATCCCTGCAATCACCATTTGCTGC

CGAGCCACTTAATCGATTTGAGCTAGGTTTTAACCAGTCTATGGTAAATCTTCTTATTAAA

GCAATCCCTTTGATCATGTTTTAGTATTTTACTTTTGATGTACATAAATTCATTATGTTTT

TATTATCCTTTCCATGCTAGATCTGTGCAAAATATCCGTATATAGATCAATTTTACGGGCT

CTTCTCGACTTATGGACCTCAAATCTCGGTTTGTCTTCACCTCGGCTATTTTATTAATCTT

TGTATATATCATGCAATGTCTATAAGTACTTGAATGTGAATTTGTTTTATTTACAGTGTGT

ATGCTTACATGAGAAAGCCAGAAATCTGTTTACAACTATCTTGTTAGTATTCTTCTAAATC

TTTCAAATGTAGAGTTGATTATGAAATTTGTTGTTAATGAGTTTTCGGCTTTATGAATGAA

TTGTAAGACAAGTAATGAAACAAAAACTAGAAGAAAATTGAATTATATGATGCAATCTGTG

TTGAAGTTTTTGCATGTATTCAAGTTCTGCTTTTAGTTACTATTTGTTTATCTGCTGCAAT

ATTTCATCAATTATTATTCATGTATGTACCAATATTTAATAAATTAGGGGCGAATTATGCT

TCCGCTTAGCATGACATCTGACGACGGACCAACATACGTGAATGCTAAGCAATACCATGGA

ATCATCAGACGCAGGCATTCTCGTGCCAAAGCTGTGCTTCAGAATAAATTGATAAAGCGTA

ACAAGGTATGAAACTGAGTATTTTTCCTCACAACAATGTTCGAAAACTTGTGAAATAGTAT

ATTTTTCCTTTTACCCTTTTTATACCTATATTAAGGTTTTGTATTTGTCTTGCAGCCATAT

ATGCACGAATCGCGTCATCTACATGCAATGCGTAGACCAAGAGGATGCGGTGGTCGTTTCT

TGAACACAAAAGTTTCTGCTAATGGAAACGGTAAAAGCGGGAGTGAAGAGAACGGAAACAT

TGGTGGCCTACAGCTGCAGTCCAGTGGTTCTCAGAGTTCCGAAGTCTTACAATCTGAGGTT

GGAACTTTAAATTCGTCGAAGGAGACAAACGGAGGCAGTCCAAATGTCTCGGGGTCAGAGG

TGACTAGCATGTATACACAGGGAGGTCTTGATAGCTTTACTGTCAATCATATCGGATCTAC

TGTCCACTCTTTGGGAGACATGATCGATACTGGACACGGTATCGTCATGCCAACAAAATGG

TTTGCAGCAGCTGGCAGACAGCTGCTGGAACCATAAGTTTCGATTCAGAAAGGAAACAAGT

GGGTTTGGTACAATGTGAAATATTTTGCACCAAACTCATCCTTTCCGAGACCAGATGAAGA

AGCTATGTTTCAGTTTGTTGTGTTTACTACGACAAATTTAGTTTCGGAAGACTACTTTTCC

ATCTGGTGCTCAGGCAACTCATTCTTGGCTTATTCTCAGGAAACTCATCCTTGGCTCGTAA

TATTTAGTAGTATTGTCATTGTCTTTCCGCGCAGGCTTGCCGTGGCATGGTAGGCATGCTA

ATGACTTTGGTATTTTCATGCAGTTATAACTATGATGTGTCTTTGTTTGTTGTTAAAATAA

AAAACATGAACTCTAGCTAGGTGCATGTGTGTGTTTTTAATCTTGTCTACTAAGTTTGGTG

TTTTGTAATGGATTTCTGACTTTATGGAGCAATGTATTGTAACTCTACTAAGAAGTGTAAC

ATTTTATTTCTCCCTCTCTAAGGATTGTATAAGAACCTCTTATTTTCAGACTCTACTTAAT

CCTATTTTCTATGTCTGTATGATTTTTATATTTCTAGGACAATCAAATTGGCTTGTAGAAG

CTCAAAAGCATGCTCACAAAGTAGGTACTTATGTAGGGAACTTCGTACTCTAATAATAACT

GGTTATTAACGTTATAATTAAATGCAAAATTTGATAAGTAGTAGGGTTTGGTAAGTAATAT

AACAACACCATGTTGGCTTGGGATTCTGAATTGTGTTATTGGAGTACAACTCTTAAATAAT

GATCGTGATGTAAATAATTTAAATTTGAAGTTACTAGATAAAAAGATAACATTATGTAATA

TATTATCAAATATATACTAAAAATATAATTTTCATCTATCTCAATTGAAAAAATATCCGGG

TTTTGAGTCTATTGTCAATGACGA

>HM045

AAAAAAATAAAGGTTCTATACTCATTAAACAAAGTGCCAAATTCAGAGATACTACTTCCT

TTGGATAATTTGTATCGTCACCAACGATCTTTGAGTTATTTCAAAAATCACATTCTCAATT

TTGTGGTCCAACATTCACAAAATGGTTTGTCGGGCTTTAAGTCTAGTAATTGTGAAACATG

CATATGCATGAGGTAATTAATTGATAATCTGAAAAGGTTAAATAGTTAATCAATTGTACTT

TGGTTATTTTTAATTTGTTCTCTATAATTTTTCAGTCAAAATTGAATTTGCTTTAACCATT

TAGTGTAGTAGTATGATATTACTTGCTCTACATTCTTGGATCTAAAAGTCTAATTAGTATC

GTATAAAAAATATTCTAGTTAATTAAGATACTTTTGTTCATTCTAATTATTCTTGGATTAC

TTGTGAAACTTTTAGGGATGAACAAGAAAGTCCACTAAAGAGGCTTCTTTTTGCTAGGAGT

CACTAGTTAGTTTTTTTTTTTTTTGACAAAAGTTAACCATTTTTTTTTATCGCATGGTAGG

AAAAAAAAAATAGAGATTTCCTTTGGTACTCATAAAAAAAAAAGTTGGATGGATAATGGAT

TAATCATAACTTATTTAACATAAAATGAATTAAGAGAACATTTGATATTTGGGTTTATTTT

GTAACATAAATATTAATATCAATGTTTGAAAGAGTTTTTAACAATAATTTGTAAAAGAAAG

CATAATATATCTATCAAAATAATTGTAAAAGAAAAAATTTCTTTTGGTGGTATGACATGGA

AAAATAATTAATTCTTATTAGATGAAAGTGTAAAATTAATTTGCATTAACAAAGCATATTT

CATTAAACTCCTTTTAGTTTTTGTTGGAAAAAATAAATTTATTGTTACTCAAAAAATAAAA

GTGTACAATTAATATATTTACTCAAAAGTCCCCTAAATGGGATGTAATTATCAAGGGATGT

GAGCGGTTTAACTTTAAGTAATGAATCAACTGTTGCATAATCACTTTGAAAGAAACTTGAG

ATTCTTCAATTAGCAAAATCTTGAATAGAATAATGAGATTATTCCTAATGCTAATGATACC

CACCTCTCCTATAAGTTGATGGGGCAGAACAAATTATGCACTCAGGACAACTTTTCTCAAA

TTTTGAAAACTTACCACACGAAGAACACACTTCCACTTGCACAAGTAAGAATATAGTATAA

CAATAAATTTACCACTCATAGCTCAATGGATATTAAAATATTGCTAATCTTTTATAGACAG

ATGTTGGCAAATTAGTATAAATTAGTTATTATATTAGTTTTTAAGGTTCAATCTACTTTTT

AAAACTCTTATTAAGTTGTTTAGCTTGCCAACCAAAGACTAAATTATCTTTATTTGTTAGG

AAATAAAAGCACTTATTTTATTTTCTCCATATTTGTTTCCTCTTTGTTCCATTACTAAAAA

AATTATAATATATTAGTAACAAAAAAATTTGGGGAGGGAATGTTATTTTTAAAAAAGATGT

TTTATTTATATTTAAGTGTTTTATTGAAATTCCTCGTGAGTTTAGCTCAATTGGTATGGAC

AATACATAATATATGCAAAATTCGAGGTTCAAACCTTGAGCACCAAAAAAAAAAGTGTTTT

ATTGAAAAATATCAAGAACTTGAAAACAAATTTCCTTTTTATGAACTAGATATTGTCACAT

AGACAACTTAAGAATTGACATTTTTTTTAACACTATTGTATTTTCTGAACTCGACTTTAAC

TCGAGATCAATGAGTAAACTAAAAAAACTCGTATTATTTTATCTAAGTGTTCTTAGTGTGA

GAAAACATTATAATTGTTTGCTTAGTCTATTTCTCCCTATATTCTCACCCTCATTCTTGGT

TTGCACATAGAAAGGAGAAAAAAAAAATTAGAGAAGAGTTGAATGGAACAGAAAGAAAGAG

CAAGCAAAATAATCACTAAAAAAAATTCCCCTAAAAGAAAATCACTGAAAAATATTATCCA

CCTCTATTTTAAATAATAATAATAATGAAAACAACAATATATTTTTTTATAGATTTTCTCT

ACTAGAGGTAATTTATTTTAAACACTAAATGTCCACACCTCTAACAAAAAAAATGCATAAT

ATTATAATAGATTATTGTAAGAAAGAGTGACAAATGTTGAATTAGTTATGGAATGAAGAGT

GAGAGAGATTATACAAATAAATAAATATAAATATAAATATAAATAAAGAATAGAGAAGAAC

ATAGTACTTGAATGAAGAGATTTGGCTTGTACTTCCATTACACTTGTAAGCTTTCTACTTC

CTTCACATTCCTTCTCTTTCTCTATCTTTCCTTTTATGATATGTGTACTTTGTTATTGCTT

CATTTACTACCGACATGACTCCAACCTATTAGGGCTTTATCTGAATAATGTTAGTTATTTT

CTCTCATTTTCTTTGCTTCTTCTTATTCTTGGAACTTATAAAGGGTCTTGTCAATTCTATC

ATTCTCTTTTGTATACTTACAAATATAAATATTCTCTTTAGCTTCATTTTCATGTCTTCTT

CTACTAATAAGCTATGGAAATTGAAGTTTTTGTTTTCATGTTTATTTTTCACTTCAGCTCC

TCTTTTTTGATGTTGATGCTGAAGATTGAATAAAAACTGAGGTGGTATGTATGAGAACAAG

AACATTTTTCATTCATCTCTGTTATTTGAACATCTAAATTTTAAACAACTATCGCGACAAT

TTTTTAAGTGGTTGAAGTATTTTAAATAGGTGGTTAATTACATTAAGTAGATAGTTAATTG

ATATATCAGGTGTCAAAACTCATTAACCATCAACATAACTTCATTAGCCATGAATTTTTGA

CGTGATTAACCAAATATTGTAGATGCTCAATTACATGTAGATTTGATGGAGTTGTCCACAA

TTTCTCATATATCTATTAACTAATAACCATCTACCGAATATAATTAACCATATATTAGAAT

ATCATTAACCTCTAAAACAGTTGTCGCGAAAGTTGGTTGTACAAAAATCATTTCTATTTTT

TACGCCACTAGTTCTCTTGTTATTTCTCTTTGTTGGAAAGTTGTTGTTTTTAACTTTGAGC

TTAGTCTTATTAATTAACTTGTAATGGTAACAACATTTTTCTATCTTTGTTTGAAGGAAGT

AGTTACTGTGTCAACTTTAGTAATTAATTTGGATCATTTTGGAGGCAAAATATGGCTATGC

AACCTGTTTATTTTAAAGAACATGAAGGAAATGTCCACAATTCTGTTGGACAGTTTTCATC

TGTGACTTCAGCACCATGGTGGAGTAATGCCTATGGATCTCAACCTGTTTATGGAGGAGAC

TCTTGTGGCCAAATGAAACCTTTTTCACTAGAGCTTTCCAACTACATAGACCAACTTGCTC

CGAGTAAGAACTTAGTTCGAGGAGTTGAACAATTGTTTGATAAAGGGCATACAAACCAATT

CACCATCTTTCCAGGTACTTGTTATTCAATATAATTCCGGTTTTGAATGAATTGATTTTTG

TTAAGTTGGTTCTGGATAAACGTGCGTTAAATATAATATGATTTATGTTTGGATACGTTTA

TGTAAAAGTGAGTTGAACATAAATTGGAGACTAAATATCAATTGCAGAGGCAAAAGCTTCA

AATTCTAATTTCAAGTTAGAATCAAATCAATTCTACTCGTAAGCATCTAAATATTTCAAAA

CCAATTCTACATCTCTAGAACTAAATTTGCATTCAAACATTTTCTGCTACAGATGATTGTA

AGATGTCAGTTGATACACCAAATCATCAAGCAACCTTATCCCTGCAATCACCATTTGCTGC

CGAGCCACTTAATCGATTTGAGCTAGGTTTTAACCAGTCTATGGTAAATCTTCTTATTAAA

GCAATCCCTTTGATCATGTTTTAGTATTTTACTTTTGATGTACATAAATTCATTATGTTTT

TATTATCCTTTCCATGCTAGATCTGTGCAAAATATCCGTATATAGATCAATTTTACGGGCT

CTTCTCGACTTATGGACCTCAAATCTCGGTTTGTCTTCACCTCGGCTATTTTATTAATCTT

TGTATATATCATGCAATGTCTATAAGTACTTGAATGTGAATTTGTTTTATTTACAGTGTGT

ATGCTTACATGAGAAAGCCAGAAATCTGTTTACAACTATCTTGTTAGTATTCTTCTAAATC

TTTCAAATGTAGAGTTGATTATGAAATTTGTTGTTAATGAGTTTTCGGCTTTATGAATGAA

TTGTAAGACAAGTAATGAAACAAAAACTAGAAGAAAATTGAATTATATGATGCAATCTGTG

TTGAAGTTTTTGCATGTATTCAAGTTCTGCTTTTAGTTACTATTTGTTTATCTGCTGCAAT

ATTTCATCAATTATTATTCATGTATGTACCAATATTTAATAAATTAGGGGCGAATTATGCT

TCCGCTTAGCATGACATCTGACGACGGACCAACATACGTGAATGCTAAGCAATACCATGGA

ATCATCAGACGCAGGCATTCTCGTGCCAAAGCTGTGCTTCAGAATAAATTGATAAAGCGTA

ACAAGGTATGAAACTGAGTATTTTTCCTCACAACAATGTTCGAAAACTTGTGAAATAGTAT

ATTTTTCCTTTTACCCTTTTTATACCTATATTAAGGTTTTGTATTTGTCTTGCAGCCATAT

ATGCACGAATCGCGTCATCTACATGCAATGCGTAGACCAAGAGGATGCGGTGGTCGTTTCT

TGAACACAAAAGTTTCTGCTAATGGAAACGGTAAAAGCGGGAGTGAAGAGAACGGAAACAT

TGGTGGCCTACAGCTGCAGTCCAGTGGTTCTCAGAGTTCCGAAGTCTTACAATCTGAGGTT

GGAACTTTAAATTCGTCGAAGGAGACAAACGGAGGCAGTCCAAATGTCTCGGGGTCAGAGG

TGACTAGCATGTATACACAGGGAGGTCTTGATAGCTTTACTGTCAATCATATCGGATCTAC

TGTCCACTCTTTGGGAGACATGATCGATACTGGACACGGTATCGTCATGCCAACAAAATGG

TTTGCAGCAGCTGGCAGACAGCTGCTGGAACCATAAGTTTCGATTCAGAAAGGAAACAAGT

GGGTTTGGTACAATGTGAAATATTTTGCACCAAACTCATCCTTTCCGAGACCAGATGAAGA

AGCTATGTTTCAGTTTGTTGTGTTTACTACGACAAATTTAGTTTCGGAAGACTACTTTTCC

ATCTGGTGCTCAGGCAACTCATTCTTGGCTTATTCTCAGGAAACTCATCCTTGGCTCGTAA

TATTTAGTAGTATTGTCATTGTCTTTCCGCGCAGGCTTGCCGTGGCATGGTAGGCATGCTA

ATGACTTTGGTATTTTCATGCAGTTATAACTATGATGTGTCTTTGTTTGTTGTTAAAATAA

AAAACATGAACTCTAGCTAGGTGCATGTGTGTGTTTTTAATCTTGTCTACTAAGTTTGGTG

TTTTGTAATGGATTTCTGACTTTATGGAGCAATGTATTGTAACTCTACTAAGAAGTGTAAC

ATTTTATTTCTCCCTCTCTAAGGATTGTATAAGAACCTCTTATTTTCAGACTCTACTTAAT

CCTATTTTCTATGTCTGTATGATTTTTATATTTCTAGGACAATCAAATTGGCTTGTAGAAG

CTCAAAAGCATGCTCACAAAGTAGGTACTTATGTAGGGAACTTCGTACTCTAATAATAACT

GGTTATTAACGTTATAATTAAATGCAAAATTTGATAAGTAGTAGGGTTTGGTAAGTAATAT

AACAACACCATGTTGGCTTGGGATTCTGAATTGTGTTATTGGAGTACAACTCTTAAATAAT

GATCGTGATGTAAATAATTTAAATTTGAAGTTACTAGATAAAAAGATAACATTATGTAATA

TATTATCAAATATATACTAAAAATATAATTTTCATCTATCTCAATTGAAAAAATATCCGGG

TTTTGAGTCTATTGTCAATGACGA

>HM046

AAAAAAATAAAGGTTCTATACTCATTAAACAAAGTGCCAAATTCAGAGATACTACTTCCT

TTGGATAATTTGTATCGTCACCAACGATCTTTGAGTTATTTCAAAAATCACATTCTCAATT

TTGTGGTCCAACATTCACAAAATGGTTTGTCGGGCTTTAAGTCTAGTAATTGTGAAACATG

CATATGCATGAGGTAATTAATTGATAATCTGAAAAGGTTAAATAGTTAATCAATTGTACTT

TGGTTATTTTTAATTTGTTCTCTATAATTTTTCAGTCAAAATTGAATTTGCTTTAACCATT

TAGTGTAGTAGTATGATATTACTTGCTCTACATTCTTGGATCTAAAAGTCTAATTAGTATC

GTATAAAAAATATTCTAGTTAATTAAGATACTTTTGTTCATTCTAATTATTCTTGGATTAC

TTGTGAAACTTTTAGGGATGAACAAGAAAGTCCACTAAAGAGGCTTCTTTTTGCTAGGAGT

CACTAGTTAGTTTTTTTTTTTTTTGACAAAAGTTAACCATTTTTTTTTATCGCATGGTAGG

AAAAAAAAAATAGAGATTTCCTTTGGTACTCATAAAAAAAAAAGTTGGATGGATAATGGAT

TAATCATAACTTATTTAACATAAAATGAATTAAGAGAACATTTGATATTTGGGTTTATTTT

GTAACATAAATATTAATATCAATGTTTGAAAGAGTTTTTAACAATAATTTGTAAAAGAAAG

CATAATATATCTATCAAAATAATTGTAAAAGAAAAAATTTCTTTTGGTGGTATGACATGGA

AAAATAATTAATTCTTATTAGATGAAAGTGTAAAATTAATTTGCATTAACAAAGCATATTT

CATTAAACTCCTTTTAGTTTTTGTTGGAAAAAATAAATTTATTGTTACTCAAAAAATAAAA

GTGTACAATTAATATATTTACTCAAAAGTCCCCTAAATGGGATGTAATTATCAAGGGATGT

GAGCGGTTTAACTTTAAGTAATGAATCAACTGTTGCATAATCACTTTGAAAGAAACTTGAG

ATTCTTCAATTAGCAAAATCTTGAATAGAATAATGAGATTATTCCTAATGCTAATGATACC

CACCTCTCCTATAAGTTGATGGGGCAGAACAAATTATGCACTCAGGACAACTTTTCTCAAA

TTTTGAAAACTTACCACACGAAGAACACACTTCCACTTGCACAAGTAAGAATATAGTATAA

CAATAAATTTACCACTCATAGCTCAATGGATATTAAAATATTGCTAATCTTTTATAGACAG

ATGTTGGCAAATTAGTATAAATTAGTTATTATATTAGTTTTTAAGGTTCAATCTACTTTTT

AAAACTCTTATTAAGTTGTTTAGCTTGCCAACCAAAGACTAAATTATCTTTATTTGTTAGG

AAATAAAAGCACTTATTTTATTTTCTCCATATTTGTTTCCTCTTTGTTCCATTACTAAAAA

AATTATAATATATTAGTAACAAAAAAATTTGGGGAGGGAATGTTATTTTTAAAAAAGATGT

TTTATTTATATTTAAGTGTTTTATTGAAATTCCTCGTGAGTTTAGCTCAATTGGTATGGAC

AATACATAATATATGCAAAATTCGAGGTTCAAACCTTGAGCACCAAAAAAAAAAGTGTTTT

ATTGAAAAATATCAAGAACTTGAAAACAAATTTCCTTTTTATGAACTAGATATTGTCACAT

AGACAACTTAAGAATTGACATTTTTTTTAACACTATTGTATTTTCTGAACTCGACTTTAAC

TCGAGATCAATGAGTAAACTAAAAAAACTCGTATTATTTTATCTAAGTGTTCTTAGTGTGA

GAAAACATTATAATTGTTTGCTTAGTCTATTTCTCCCTATATTCTCACCCTCATTCTTGGT

TTGCACATAGAAAGGAGAAAAAAAAAATTAGAGAAGAGTTGAATGGAACAGAAAGAAAGAG

CAAGCAAAATAATCACTAAAAAAAATTCCCCTAAAAGAAAATCACTGAAAAATATTATCCA

CCTCTATTTTAAATAATAATAATAATGAAAACAACAATATATTTTTTTATAGATTTTCTCT

ACTAGAGGTAATTTATTTTAAACACTAAATGTCCACACCTCTAACAAAAAAAATGCATAAT

ATTATAATAGATTATTGTAAGAAAGAGTGACAAATGTTGAATTAGTTATGGAATGAAGAGT

GAGAGAGATTATACAAATAAATAAATATAAATATAAATATAAATAAAGAATAGAGAAGAAC

ATAGTACTTGAATGAAGAGATTTGGCTTGTACTTCCATTACACTTGTAAGCTTTCTACTTC

CTTCACATTCCTTCTCTTTCTCTATCTTTCCTTTTATGATATGTGTACTTTGTTATTGCTT

CATTTACTACCGACATGACTCCAACCTATTAGGGCTTTATCTGAATAATGTTAGTTATTTT

CTCTCATTTTCTTTGCTTCTTCTTATTCTTGGAACTTATAAAGGGTCTTGTCAATTCTATC

ATTCTCTTTTGTATACTTACAAATATAAATATTCTCTTTAGCTTCATTTTCATGTCTTCTT

CTACTAATAAGCTATGGAAATTGAAGTTTTTGTTTTCATGTTTATTTTTCACTTCAGCTCC

TCTTTTTTGATGTTGATGCTGAAGATTGAATAAAAACTGAGGTGGTATGTATGAGAACAAG

AACATTTTTCATTCATCTCTGTTATTTGAACATCTAAATTTTAAACAACTATCGCGACAAT

TTTTTAAGTGGTTGAAGTATTTTAAATAGGTGGTTAATTACATTAAGTAGATAGTTAATTG

ATATATCAGGTGTCAAAACTCATTAACCATCAACATAACTTCATTAGCCATGAATTTTTGA

CGTGATTAACCAAATATTGTAGATGCTCAATTACATGTAGATTTGATGGAGTTGTCCACAA

TTTCTCATATATCTATTAACTAATAACCATCTACCGAATATAATTAACCATATATTAGAAT

ATCATTAACCTCTAAAACAGTTGTCGCGAAAGTTGGTTGTACAAAAATCATTTCTATTTTT

TACGCCACTAGTTCTCTTGTTATTTCTCTTTGTTGGAAAGTTGTTGTTTTTAACTTTGAGC

TTAGTCTTATTAATTAACTTGTAATGGTAACAACATTTTTCTATCTTTGTTTGAAGGAAGT

AGTTACTGTGTCAACTTTAGTAATTAATTTGGATCATTTTGGAGGCAAAATATGGCTATGC

AACCTGTTTATTTTAAAGAACATGAAGGAAATGTCCACAATTCTGTTGGACAGTTTTCATC

TGTGACTTCAGCACCATGGTGGAGTAATGCCTATGGATCTCAACCTGTTTATGGAGGAGAC

TCTTGTGGCCAAATGAAACCTTTTTCACTAGAGCTTTCCAACTACATAGACCAACTTGCTC

CGAGTAAGAACTTAGTTCGAGGAGTTGAACAATTGTTTGATAAAGGGCATACAAACCAATT

CACCATCTTTCCAGGTACTTGTTATTCAATATAATTCCGGTTTTGAATGAATTGATTTTTG

TTAAGTTGGTTCTGGATAAACGTGCGTTAAATATAATATGATTTATGTTTGGATACGTTTA

TGTAAAAGTGAGTTGAACATAAATTGGAGACTAAATATCAATTGCAGAGGCAAAAGCTTCA

AATTCTAATTTCAAGTTAGAATCAAATCAATTCTACTCGTAAGCATCTAAATATTTCAAAA

CCAATTCTACATCTCTAGAACTAAATTTGCATTCAAACATTTTCTGCTACAGATGATTGTA

AGATGTCAGTTGATACACCAAATCATCAAGCAACCTTATCCCTGCAATCACCATTTGCTGC

CGAGCCACTTAATCGATTTGAGCTAGGTTTTAACCAGTCTATGGTAAATCTTCTTATTAAA

GCAATCCCTTTGATCATGTTTTAGTATTTTACTTTTGATGTACATAAATTCATTATGTTTT

TATTATCCTTTCCATGCTAGATCTGTGCAAAATATCCGTATATAGATCAATTTTACGGGCT

CTTCTCGACTTATGGACCTCAAATCTCGGTTTGTCTTCACCTCGGCTATTTTATTAATCTT

TGTATATATCATGCAATGTCTATAAGTACTTGAATGTGAATTTGTTTTATTTACAGTGTGT

ATGCTTACATGAGAAAGCCAGAAATCTGTTTACAACTATCTTGTTAGTATTCTTCTAAATC

TTTCAAATGTAGAGTTGATTATGAAATTTGTTGTTAATGAGTTTTCGGCTTTATGAATGAA

TTGTAAGACAAGTAATGAAACAAAAACTAGAAGAAAATTGAATTATATGATGCAATCTGTG

TTGAAGTTTTTGCATGTATTCAAGTTCTGCTTTTAGTTACTATTTGTTTATCTGCTGCAAT

ATTTCATCAATTATTATTCATGTATGTACCAATATTTAATAAATTAGGGGCGAATTATGCT

TCCGCTTAGCATGACATCTGACGACGGACCAACATACGTGAATGCTAAGCAATACCATGGA

ATCATCAGACGCAGGCATTCTCGTGCCAAAGCTGTGCTTCAGAATAAATTGATAAAGCGTA

ACAAGGTATGAAACTGAGTATTTTTCCTCACAACAATGTTCGAAAACTTGTGAAATAGTAT

ATTTTTCCTTTTACCCTTTTTATACCTATATTAAGGTTTTGTATTTGTCTTGCAGCCATAT

ATGCACGAATCGCGTCATCTACATGCAATGCGTAGACCAAGAGGATGCGGTGGTCGTTTCT

TGAACACAAAAGTTTCTGCTAATGGAAACGGTAAAAGCGGGAGTGAAGAGAACGGAAACAT

TGGTGGCCTACAGCTGCAGTCCAGTGGTTCTCAGAGTTCCGAAGTCTTACAATCTGAGGTT

GGAACTTTAAATTCGTCGAAGGAGACAAACGGAGGCAGTCCAAATGTCTCGGGGTCAGAGG

TGACTAGCATGTATACACAGGGAGGTCTTGATAGCTTTACTGTCAATCATATCGGATCTAC

TGTCCACTCTTTGGGAGACATGATCGATACTGGACACGGTATCGTCATGCCAACAAAATGG

TTTGCAGCAGCTGGCAGACAGCTGCTGGAACCATAAGTTTCGATTCAGAAAGGAAACAAGT

GGGTTTGGTACAATGTGAAATATTTTGCACCAAACTCATCCTTTCCGAGACCAGATGAAGA

AGCTATGTTTCAGTTTGTTGTGTTTACTACGACAAATTTAGTTTCGGAAGACTACTTTTCC

ATCTGGTGCTCAGGCAACTCATTCTTGGCTTATTCTCAGGAAACTCATCCTTGGCTCGTAA

TATTTAGTAGTATTGTCATTGTCTTTCCGCGCAGGCTTGCCGTGGCATGGTAGGCATGCTA

ATGACTTTGGTATTTTCATGCAGTTATAACTATGATGTGTCTTTGTTTGTTGTTAAAATAA

AAAACATGAACTCTAGCTAGGTGCATGTGTGTGTTTTTAATCTTGTCTACTAAGTTTGGTG

TTTTGTAATGGATTTCTGACTTTATGGAGCAATGTATTGTAACTCTACTAAGAAGTGTAAC

ATTTTATTTCTCCCTCTCTAAGGATTGTATAAGAACCTCTTATTTTCAGACTCTACTTAAT

CCTATTTTCTATGTCTGTATGATTTTTATATTTCTAGGACAATCAAATTGGCTTGTAGAAG

CTCAAAAGCATGCTCACAAAGTAGGTACTTATGTAGGGAACTTCGTACTCTAATAATAACT

GGTTATTAACGTTATAATTAAATGCAAAATTTGATAAGTAGTAGGGTTTGGTAAGTAATAT

AACAACACCATGTTGGCTTGGGATTCTGAATTGTGTTATTGGAGTACAACTCTTAAATAAT

GATCGTGATGTAAATAATTTAAATTTGAAGTTACTAGATAAAAAGATAACATTATGTAATA

TATTATCAAATATATACTAAAAATATAATTTTCATCTATCTCAATTGAAAAAATATCCGGG

TTTTGAGTCTATTGTCAATGACGA

>HM047

AAAAAAATAAAGGTTCTATACTCATTAAACAAAGTGCCAAATTCAGAGATACTACTTCCT

TTGGATAATTTGTATCGTCACCAACGATCTTTGAGTTATTTCAAAAATCACATTCTCAATT

TTGTGGTCCAACATTCACAAAATGGTTTGTCGGGCTTTAAGTCTAGTAATTGTGAAACATG

CATATGCATGAGGTAATTAATTGATAATCTGAAAAGGTTAAATAGTTAATCAATTGTACTT

TGGTTATTTTTAATTTGTTCTCTATAATTTTTCAGTCAAAATTGAATTTGCTTTAACCATT

TAGTGTAGTAGTATGATATTACTTGCTCTACATTCTTGGATCTAAAAGTCTAATTAGTATC

GTATAAAAAATATTCTAGTTAATTAAGATACTTTTGTTCATTCTAATTATTCTTGGATTAC

TTGTGAAACTTTTAGGGATGAACAAGAAAGTCCACTAAAGAGGCTTCTTTTTGCTAGGAGT

CACTAGTTAGTTTTTTTTTTTTTTGACAAAAGTTAACCATTTTTTTTTATCGCATGGTAGG

AAAAAAAAAATAGAGATTTCCTTTGGTACTCATAAAAAAAAAAGTTGGATGGATAATGGAT

TAATCATAACTTATTTAACATAAAATGAATTAAGAGAACATTTGATATTTGGGTTTATTTT

GTAACATAAATATTAATATCAATGTTTGAAAGAGTTTTTAACAATAATTTGTAAAAGAAAG

CATAATATATCTATCAAAATAATTGTAAAAGAAAAAATTTCTTTTGGTGGTATGACATGGA

AAAATAATTAATTCTTATTAGATGAAAGTGTAAAATTAATTTGCATTAACAAAGCATATTT

CATTAAACTCCTTTTAGTTTTTGTTGGAAAAAATAAATTTATTGTTACTCAAAAAATAAAA

GTGTACAATTAATATATTTACTCAAAAGTCCCCTAAATGGGATGTAATTATCAAGGGATGT

GAGCGGTTTAACTTTAAGTAATGAATCAACTGTTGCATAATCACTTTGAAAGAAACTTGAG

ATTCTTCAATTAGCAAAATCTTGAATAGAATAATGAGATTATTCCTAATGCTAATGATACC

CACCTCTCCTATAAGTTGATGGGGCAGAACAAATTATGCACTCAGGACAACTTTTCTCAAA

TTTTGAAAACTTACCACACGAAGAACACACTTCCACTTGCACAAGTAAGAATATAGTATAA

CAATAAATTTACCACTCATAGCTCAATGGATATTAAAATATTGCTAATCTTTTATAGACAG

ATGTTGGCAAATTAGTATAAATTAGTTATTATATTAGTTTTTAAGGTTCAATCTACTTTTT

AAAACTCTTATTAAGTTGTTTAGCTTGCCAACCAAAGACTAAATTATCTTTATTTGTTAGG

AAATAAAAGCACTTATTTTATTTTCTCCATATTTGTTTCCTCTTTGTTCCATTACTAAAAA

AATTATAATATATTAGTAACAAAAAAATTTGGGGAGGGAATGTTATTTTTAAAAAAGATGT

TTTATTTATATTTAAGTGTTTTATTGAAATTCCTCGTGAGTTTAGCTCAATTGGTATGGAC

AATACATAATATATGCAAAATTCGAGGTTCAAACCTTGAGCACCAAAAAAAAAAGTGTTTT

ATTGAAAAATATCAAGAACTTGAAAACAAATTTCCTTTTTATGAACTAGATATTGTCACAT

AGACAACTTAAGAATTGACATTTTTTTTAACACTATTGTATTTTCTGAACTCGACTTTAAC

TCGAGATCAATGAGTAAACTAAAAAAACTCGTATTATTTTATCTAAGTGTTCTTAGTGTGA

GAAAACATTATAATTGTTTGCTTAGTCTATTTCTCCCTATATTCTCACCCTCATTCTTGGT

TTGCACATAGAAAGGAGAAAAAAAAAATTAGAGAAGAGTTGAATGGAACAGAAAGAAAGAG

CAAGCAAAATAATCACTAAAAAAAATTCCCCTAAAAGAAAATCACTGAAAAATATTATCCA

CCTCTATTTTAAATAATAATAATAATGAAAACAACAATATATTTTTTTATAGATTTTCTCT

ACTAGAGGTAATTTATTTTAAACACTAAATGTCCACACCTCTAACAAAAAAAATGCATAAT

ATTATAATAGATTATTGTAAGAAAGAGTGACAAATGTTGAATTAGTTATGGAATGAAGAGT

GAGAGAGATTATACAAATAAATAAATATAAATATAAATATAAATAAAGAATAGAGAAGAAC

ATAGTACTTGAATGAAGAGATTTGGCTTGTACTTCCATTACACTTGTAAGCTTTCTACTTC

CTTCACATTCCTTCTCTTTCTCTATCTTTCCTTTTATGATATGTGTACTTTGTTATTGCTT

CATTTACTACCGACATGACTCCAACCTATTAGGGCTTTATCTGAATAATGTTAGTTATTTT

CTCTCATTTTCTTTGCTTCTTCTTATTCTTGGAACTTATAAAGGGTCTTGTCAATTCTATC

ATTCTCTTTTGTATACTTACAAATATAAATATTCTCTTTAGCTTCATTTTCATGTCTTCTT

CTACTAATAAGCTATGGAAATTGAAGTTTTTGTTTTCATGTTTATTTTTCACTTCAGCTCC

TCTTTTTTGATGTTGATGCTGAAGATTGAATAAAAACTGAGGTGGTATGTATGAGAACAAG

AACATTTTTCATTCATCTCTGTTATTTGAACATCTAAATTTTAAACAACTATCGCGACAAT

TTTTTAAGTGGTTGAAGTATTTTAAATAGGTGGTTAATTACATTAAGTAGATAGTTAATTG

ATATATCAGGTGTCAAAACTCATTAACCATCAACATAACTTCATTAGCCATGAATTTTTGA

CGTGATTAACCAAATATTGTAGATGCTCAATTACATGTAGATTTGATGGAGTTGTCCACAA

TTTCTCATATATCTATTAACTAATAACCATCTACCGAATATAATTAACCATATATTAGAAT

ATCATTAACCTCTAAAACAGTTGTCGCGAAAGTTGGTTGTACAAAAATCATTTCTATTTTT

TACGCCACTAGTTCTCTTGTTATTTCTCTTTGTTGGAAAGTTGTTGTTTTTAACTTTGAGC

TTAGTCTTATTAATTAACTTGTAATGGTAACAACATTTTTCTATCTTTGTTTGAAGGAAGT

AGTTACTGTGTCAACTTTAGTAATTAATTTGGATCATTTTGGAGGCAAAATATGGCTATGC

AACCTGTTTATTTTAAAGAACATGAAGGAAATGTCCACAATTCTGTTGGACAGTTTTCATC

TGTGACTTCAGCACCATGGTGGAGTAATGCCTATGGATCTCAACCTGTTTATGGAGGAGAC

TCTTGTGGCCAAATGAAACCTTTTTCACTAGAGCTTTCCAACTACATAGACCAACTTGCTC

CGAGTAAGAACTTAGTTCGAGGAGTTGAACAATTGTTTGATAAAGGGCATACAAACCAATT

CACCATCTTTCCAGGTACTTGTTATTCAATATAATTCCGGTTTTGAATGAATTGATTTTTG

TTAAGTTGGTTCTGGATAAACGTGCGTTAAATATAATATGATTTATGTTTGGATACGTTTA

TGTAAAAGTGAGTTGAACATAAATTGGAGACTAAATATCAATTGCAGAGGCAAAAGCTTCA

AATTCTAATTTCAAGTTAGAATCAAATCAATTCTACTCGTAAGCATCTAAATATTTCAAAA

CCAATTCTACATCTCTAGAACTAAATTTGCATTCAAACATTTTCTGCTACAGATGATTGTA

AGATGTCAGTTGATACACCAAATCATCAAGCAACCTTATCCCTGCAATCACCATTTGCTGC

CGAGCCACTTAATCGATTTGAGCTAGGTTTTAACCAGTCTATGGTAAATCTTCTTATTAAA

GCAATCCCTTTGATCATGTTTTAGTATTTTACTTTTGATGTACATAAATTCATTATGTTTT

TATTATCCTTTCCATGCTAGATCTGTGCAAAATATCCGTATATAGATCAATTTTACGGGCT

CTTCTCGACTTATGGACCTCAAATCTCGGTTTGTCTTCACCTCGGCTATTTTATTAATCTT

TGTATATATCATGCAATGTCTATAAGTACTTGAATGTGAATTTGTTTTATTTACAGTGTGT

ATGCTTACATGAGAAAGCCAGAAATCTGTTTACAACTATCTTGTTAGTATTCTTCTAAATC

TTTCAAATGTAGAGTTGATTATGAAATTTGTTGTTAATGAGTTTTCGGCTTTATGAATGAA

TTGTAAGACAAGTAATGAAACAAAAACTAGAAGAAAATTGAATTATATGATGCAATCTGTG

TTGAAGTTTTTGCATGTATTCAAGTTCTGCTTTTAGTTACTATTTGTTTATCTGCTGCAAT

ATTTCATCAATTATTATTCATGTATGTACCAATATTTAATAAATTAGGGGCGAATTATGCT

TCCGCTTAGCATGACATCTGACGACGGACCAACATACGTGAATGCTAAGCAATACCATGGA

ATCATCAGACGCAGGCATTCTCGTGCCAAAGCTGTGCTTCAGAATAAATTGATAAAGCGTA

ACAAGGTATGAAACTGAGTATTTTTCCTCACAACAATGTTCGAAAACTTGTGAAATAGTAT

ATTTTTCCTTTTACCCTTTTTATACCTATATTAAGGTTTTGTATTTGTCTTGCAGCCATAT

ATGCACGAATCGCGTCATCTACATGCAATGCGTAGACCAAGAGGATGCGGTGGTCGTTTCT

TGAACACAAAAGTTTCTGCTAATGGAAACGGTAAAAGCGGGAGTGAAGAGAACGGAAACAT

TGGTGGCCTACAGCTGCAGTCCAGTGGTTCTCAGAGTTCCGAAGTCTTACAATCTGAGGTT

GGAACTTTAAATTCGTCGAAGGAGACAAACGGAGGCAGTCCAAATGTCTCGGGGTCAGAGG

TGACTAGCATGTATACACAGGGAGGTCTTGATAGCTTTACTGTCAATCATATCGGATCTAC

TGTCCACTCTTTGGGAGACATGATCGATACTGGACACGGTATCGTCATGCCAACAAAATGG

TTTGCAGCAGCTGGCAGACAGCTGCTGGAACCATAAGTTTCGATTCAGAAAGGAAACAAGT

GGGTTTGGTACAATGTGAAATATTTTGCACCAAACTCATCCTTTCCGAGACCAGATGAAGA

AGCTATGTTTCAGTTTGTTGTGTTTACTACGACAAATTTAGTTTCGGAAGACTACTTTTCC

ATCTGGTGCTCAGGCAACTCATTCTTGGCTTATTCTCAGGAAACTCATCCTTGGCTCGTAA

TATTTAGTAGTATTGTCATTGTCTTTCCGCGCAGGCTTGCCGTGGCATGGTAGGCATGCTA

ATGACTTTGGTATTTTCATGCAGTTATAACTATGATGTGTCTTTGTTTGTTGTTAAAATAA

AAAACATGAACTCTAGCTAGGTGCATGTGTGTGTTTTTAATCTTGTCTACTAAGTTTGGTG

TTTTGTAATGGATTTCTGACTTTATGGAGCAATGTATTGTAACTCTACTAAGAAGTGTAAC

ATTTTATTTCTCCCTCTCTAAGGATTGTATAAGAACCTCTTATTTTCAGACTCTACTTAAT

CCTATTTTCTATGTCTGTATGATTTTTATATTTCTAGGACAATCAAATTGGCTTGTAGAAG

CTCAAAAGCATGCTCACAAAGTAGGTACTTATGTAGGGAACTTCGTACTCTAATAATAACT

GGTTATTAACGTTATAATTAAATGCAAAATTTGATAAGTAGTAGGGTTTGGTAAGTAATAT

AACAACACCATGTTGGCTTGGGATTCTGAATTGTGTTATTGGAGTACAACTCTTAAATAAT

GATCGTGATGTAAATAATTTAAATTTGAAGTTACTAGATAAAAAGATAACATTATGTAATA

TATTATCAAATATATACTAAAAATATAATTTTCATCTATCTCAATTGAAAAAATATCCGGG

TTTTGAGTCTATTGTCAATGACGA

>HM048

AAAAAAATAAAGGTTCTATACTCATTAAACAAAGTGCCAAATTCAGAGATACTACTTCCT

TTGGATAATTTGTATCGTCACCAACGATCTTTGAGTTATTTCAAAAATCACATTCTCAATT

TTGTGGTCCAACATTCACAAAATGGTTTGTCGGGCTTTAAGTCTAGTAATTGTGAAACATG

CATATGCATGAGGTAATTAATTGATAATCTGAAAAGGTTAAATAGTTAATCAATTGTACTT

TGGTTATTTTTAATTTGTTCTCTATAATTTTTCAGTCAAAATTGAATTTGCTTTAACCATT

TAGTGTAGTAGTATGATATTACTTGCTCTACATTCTTGGATCTAAAAGTCTAATTAGTATC

GTATAAAAAATATTCTAGTTAATTAAGATACTTTTGTTCATTCTAATTATTCTTGGATTAC

TTGTGAAACTTTTAGGGATGAACAAGAAAGTCCACTAAAGAGGCTTCTTTTTGCTAGGAGT

CACTAGTTAGTTTTTTTTTTTTTTGACAAAAGTTAACCATTTTTTTTTATCGCATGGTAGG

AAAAAAAAAATAGAGATTTCCTTTGGTACTCATAAAAAAAAAAGTTGGATGGATAATGGAT

TAATCATAACTTATTTAACATAAAATGAATTAAGAGAACATTTGATATTTGGGTTTATTTT

GTAACATAAATATTAATATCAATGTTTGAAAGAGTTTTTAACAATAATTTGTAAAAGAAAG

CATAATATATCTATCAAAATAATTGTAAAAGAAAAAATTTCTTTTGGTGGTATGACATGGA

AAAATAATTAATTCTTATTAGATGAAAGTGTAAAATTAATTTGCATTAACAAAGCATATTT

CATTAAACTCCTTTTAGTTTTTGTTGGAAAAAATAAATTTATTGTTACTCAAAAAATAAAA

GTGTACAATTAATATATTTACTCAAAAGTCCCCTAAATGGGATGTAATTATCAAGGGATGT

GAGCGGTTTAACTTTAAGTAATGAATCAACTGTTGCATAATCACTTTGAAAGAAACTTGAG

ATTCTTCAATTAGCAAAATCTTGAATAGAATAATGAGATTATTCCTAATGCTAATGATACC

CACCTCTCCTATAAGTTGATGGGGCAGAACAAATTATGCACTCAGGACAACTTTTCTCAAA

TTTTGAAAACTTACCACACGAAGAACACACTTCCACTTGCACAAGTAAGAATATAGTATAA

CAATAAATTTACCACTCATAGCTCAATGGATATTAAAATATTGCTAATCTTTTATAGACAG

ATGTTGGCAAATTAGTATAAATTAGTTATTATATTAGTTTTTAAGGTTCAATCTACTTTTT

AAAACTCTTATTAAGTTGTTTAGCTTGCCAACCAAAGACTAAATTATCTTTATTTGTTAGG

AAATAAAAGCACTTATTTTATTTTCTCCATATTTGTTTCCTCTTTGTTCCATTACTAAAAA

AATTATAATATATTAGTAACAAAAAAATTTGGGGAGGGAATGTTATTTTTAAAAAAGATGT

TTTATTTATATTTAAGTGTTTTATTGAAATTCCTCGTGAGTTTAGCTCAATTGGTATGGAC

AATACATAATATATGCAAAATTCGAGGTTCAAACCTTGAGCACCAAAAAAAAAAGTGTTTT

ATTGAAAAATATCAAGAACTTGAAAACAAATTTCCTTTTTATGAACTAGATATTGTCACAT

AGACAACTTAAGAATTGACATTTTTTTTAACACTATTGTATTTTCTGAACTCGACTTTAAC

TCGAGATCAATGAGTAAACTAAAAAAACTCGTATTATTTTATCTAAGTGTTCTTAGTGTGA

GAAAACATTATAATTGTTTGCTTAGTCTATTTCTCCCTATATTCTCACCCTCATTCTTGGT

TTGCACATAGAAAGGAGAAAAAAAAAATTAGAGAAGAGTTGAATGGAACAGAAAGAAAGAG

CAAGCAAAATAATCACTAAAAAAAATTCCCCTAAAAGAAAATCACTGAAAAATATTATCCA

CCTCTATTTTAAATAATAATAATAATGAAAACAACAATATATTTTTTTATAGATTTTCTCT

ACTAGAGGTAATTTATTTTAAACACTAAATGTCCACACCTCTAACAAAAAAAATGCATAAT

ATTATAATAGATTATTGTAAGAAAGAGTGACAAATGTTGAATTAGTTATGGAATGAAGAGT

GAGAGAGATTATACAAATAAATAAATATAAATATAAATATAAATAAAGAATAGAGAAGAAC

ATAGTACTTGAATGAAGAGATTTGGCTTGTACTTCCATTACACTTGTAAGCTTTCTACTTC

CTTCACATTCCTTCTCTTTCTCTATCTTTCCTTTTATGATATGTGTACTTTGTTATTGCTT

CATTTACTACCGACATGACTCCAACCTATTAGGGCTTTATCTGAATAATGTTAGTTATTTT

CTCTCATTTTCTTTGCTTCTTCTTATTCTTGGAACTTATAAAGGGTCTTGTCAATTCTATC

ATTCTCTTTTGTATACTTACAAATATAAATATTCTCTTTAGCTTCATTTTCATGTCTTCTT

CTACTAATAAGCTATGGAAATTGAAGTTTTTGTTTTCATGTTTATTTTTCACTTCAGCTCC

TCTTTTTTGATGTTGATGCTGAAGATTGAATAAAAACTGAGGTGGTATGTATGAGAACAAG

AACATTTTTCATTCATCTCTGTTATTTGAACATCTAAATTTTAAACAACTATCGCGACAAT

TTTTTAAGTGGTTGAAGTATTTTAAATAGGTGGTTAATTACATTAAGTAGATAGTTAATTG

ATATATCAGGTGTCAAAACTCATTAACCATCAACATAACTTCATTAGCCATGAATTTTTGA

CGTGATTAACCAAATATTGTAGATGCTCAATTACATGTAGATTTGATGGAGTTGTCCACAA

TTTCTCATATATCTATTAACTAATAACCATCTACCGAATATAATTAACCATATATTAGAAT

ATCATTAACCTCTAAAACAGTTGTCGCGAAAGTTGGTTGTACAAAAATCATTTCTATTTTT

TACGCCACTAGTTCTCTTGTTATTTCTCTTTGTTGGAAAGTTGTTGTTTTTAACTTTGAGC

TTAGTCTTATTAATTAACTTGTAATGGTAACAACATTTTTCTATCTTTGTTTGAAGGAAGT

AGTTACTGTGTCAACTTTAGTAATTAATTTGGATCATTTTGGAGGCAAAATATGGCTATGC

AACCTGTTTATTTTAAAGAACATGAAGGAAATGTCCACAATTCTGTTGGACAGTTTTCATC

TGTGACTTCAGCACCATGGTGGAGTAATGCCTATGGATCTCAACCTGTTTATGGAGGAGAC

TCTTGTGGCCAAATGAAACCTTTTTCACTAGAGCTTTCCAACTACATAGACCAACTTGCTC

CGAGTAAGAACTTAGTTCGAGGAGTTGAACAATTGTTTGATAAAGGGCATACAAACCAATT

CACCATCTTTCCAGGTACTTGTTATTCAATATAATTCCGGTTTTGAATGAATTGATTTTTG

TTAAGTTGGTTCTGGATAAACGTGCGTTAAATATAATATGATTTATGTTTGGATACGTTTA

TGTAAAAGTGAGTTGAACATAAATTGGAGACTAAATATCAATTGCAGAGGCAAAAGCTTCA

AATTCTAATTTCAAGTTAGAATCAAATCAATTCTACTCGTAAGCATCTAAATATTTCAAAA

CCAATTCTACATCTCTAGAACTAAATTTGCATTCAAACATTTTCTGCTACAGATGATTGTA

AGATGTCAGTTGATACACCAAATCATCAAGCAACCTTATCCCTGCAATCACCATTTGCTGC

CGAGCCACTTAATCGATTTGAGCTAGGTTTTAACCAGTCTATGGTAAATCTTCTTATTAAA

GCAATCCCTTTGATCATGTTTTAGTATTTTACTTTTGATGTACATAAATTCATTATGTTTT

TATTATCCTTTCCATGCTAGATCTGTGCAAAATATCCGTATATAGATCAATTTTACGGGCT

CTTCTCGACTTATGGACCTCAAATCTCGGTTTGTCTTCACCTCGGCTATTTTATTAATCTT

TGTATATATCATGCAATGTCTATAAGTACTTGAATGTGAATTTGTTTTATTTACAGTGTGT

ATGCTTACATGAGAAAGCCAGAAATCTGTTTACAACTATCTTGTTAGTATTCTTCTAAATC

TTTCAAATGTAGAGTTGATTATGAAATTTGTTGTTAATGAGTTTTCGGCTTTATGAATGAA

TTGTAAGACAAGTAATGAAACAAAAACTAGAAGAAAATTGAATTATATGATGCAATCTGTG

TTGAAGTTTTTGCATGTATTCAAGTTCTGCTTTTAGTTACTATTTGTTTATCTGCTGCAAT

ATTTCATCAATTATTATTCATGTATGTACCAATATTTAATAAATTAGGGGCGAATTATGCT

TCCGCTTAGCATGACATCTGACGACGGACCAACATACGTGAATGCTAAGCAATACCATGGA

ATCATCAGACGCAGGCATTCTCGTGCCAAAGCTGTGCTTCAGAATAAATTGATAAAGCGTA

ACAAGGTATGAAACTGAGTATTTTTCCTCACAACAATGTTCGAAAACTTGTGAAATAGTAT

ATTTTTCCTTTTACCCTTTTTATACCTATATTAAGGTTTTGTATTTGTCTTGCAGCCATAT

ATGCACGAATCGCGTCATCTACATGCAATGCGTAGACCAAGAGGATGCGGTGGTCGTTTCT

TGAACACAAAAGTTTCTGCTAATGGAAACGGTAAAAGCGGGAGTGAAGAGAACGGAAACAT

TGGTGGCCTACAGCTGCAGTCCAGTGGTTCTCAGAGTTCCGAAGTCTTACAATCTGAGGTT

GGAACTTTAAATTCGTCGAAGGAGACAAACGGAGGCAGTCCAAATGTCTCGGGGTCAGAGG

TGACTAGCATGTATACACAGGGAGGTCTTGATAGCTTTACTGTCAATCATATCGGATCTAC

TGTCCACTCTTTGGGAGACATGATCGATACTGGACACGGTATCGTCATGCCAACAAAATGG

TTTGCAGCAGCTGGCAGACAGCTGCTGGAACCATAAGTTTCGATTCAGAAAGGAAACAAGT

GGGTTTGGTACAATGTGAAATATTTTGCACCAAACTCATCCTTTCCGAGACCAGATGAAGA

AGCTATGTTTCAGTTTGTTGTGTTTACTACGACAAATTTAGTTTCGGAAGACTACTTTTCC

ATCTGGTGCTCAGGCAACTCATTCTTGGCTTATTCTCAGGAAACTCATCCTTGGCTCGTAA

TATTTAGTAGTATTGTCATTGTCTTTCCGCGCAGGCTTGCCGTGGCATGGTAGGCATGCTA

ATGACTTTGGTATTTTCATGCAGTTATAACTATGATGTGTCTTTGTTTGTTGTTAAAATAA

AAAACATGAACTCTAGCTAGGTGCATGTGTGTGTTTTTAATCTTGTCTACTAAGTTTGGTG

TTTTGTAATGGATTTCTGACTTTATGGAGCAATGTATTGTAACTCTACTAAGAAGTGTAAC

ATTTTATTTCTCCCTCTCTAAGGATTGTATAAGAACCTCTTATTTTCAGACTCTACTTAAT

CCTATTTTCTATGTCTGTATGATTTTTATATTTCTAGGACAATCAAATTGGCTTGTAGAAG

CTCAAAAGCATGCTCACAAAGTAGGTACTTATGTAGGGAACTTCGTACTCTAATAATAACT

GGTTATTAACGTTATAATTAAATGCAAAATTTGATAAGTAGTAGGGTTTGGTAAGTAATAT

AACAACACCATGTTGGCTTGGGATTCTGAATTGTGTTATTGGAGTACAACTCTTAAATAAT

GATCGTGATGTAAATAATTTAAATTTGAAGTTACTAGATAAAAAGATAACATTATGTAATA

TATTATCAAATATATACTAAAAATATAATTTTCATCTATCTCAATTGAAAAAATATCCGGG

TTTTGAGTCTATTGTCAATGACGA

>HM049

AAAAAAATAAAGGTTCTATACTCATTAAACAAAGTGCCAAATTCAGAGATACTACTTCCT

TTGGATAATTTGTATCGTCACCAACGATCTTTGAGTTATTTCAAAAATCACATTCTCAATT

TTGTGGTCCAACATTCACAAAATGGTTTGTCGGGCTTTAAGTCTAGTAATTGTGAAACATG

CATATGCATGAGGTAATTAATTGATAATCTGAAAAGGTTAAATAGTTAATCAATTGTACTT

TGGTTATTTTTAATTTGTTCTCTATAATTTTTCAGTCAAAATTGAATTTGCTTTAACCATT

TAGTGTAGTAGTATGATATTACTTGCTCTACATTCTTGGATCTAAAAGTCTAATTAGTATC

GTATAAAAAATATTCTAGTTAATTAAGATACTTTTGTTCATTCTAATTATTCTTGGATTAC

TTGTGAAACTTTTAGGGATGAACAAGAAAGTCCACTAAAGAGGCTTCTTTTTGCTAGGAGT

CACTAGTTAGTTTTTTTTTTTTTTGACAAAAGTTAACCATTTTTTTTTATCGCATGGTAGG

AAAAAAAAAATAGAGATTTCCTTTGGTACTCATAAAAAAAAAAGTTGGATGGATAATGGAT

TAATCATAACTTATTTAACATAAAATGAATTAAGAGAACATTTGATATTTGGGTTTATTTT

GTAACATAAATATTAATATCAATGTTTGAAAGAGTTTTTAACAATAATTTGTAAAAGAAAG

CATAATATATCTATCAAAATAATTGTAAAAGAAAAAATTTCTTTTGGTGGTATGACATGGA

AAAATAATTAATTCTTATTAGATGAAAGTGTAAAATTAATTTGCATTAACAAAGCATATTT

CATTAAACTCCTTTTAGTTTTTGTTGGAAAAAATAAATTTATTGTTACTCAAAAAATAAAA

GTGTACAATTAATATATTTACTCAAAAGTCCCCTAAATGGGATGTAATTATCAAGGGATGT

GAGCGGTTTAACTTTAAGTAATGAATCAACTGTTGCATAATCACTTTGAAAGAAACTTGAG

ATTCTTCAATTAGCAAAATCTTGAATAGAATAATGAGATTATTCCTAATGCTAATGATACC

CACCTCTCCTATAAGTTGATGGGGCAGAACAAATTATGCACTCAGGACAACTTTTCTCAAA

TTTTGAAAACTTACCACACGAAGAACACACTTCCACTTGCACAAGTAAGAATATAGTATAA

CAATAAATTTACCACTCATAGCTCAATGGATATTAAAATATTGCTAATCTTTTATAGACAG

ATGTTGGCAAATTAGTATAAATTAGTTATTATATTAGTTTTTAAGGTTCAATCTACTTTTT

AAAACTCTTATTAAGTTGTTTAGCTTGCCAACCAAAGACTAAATTATCTTTATTTGTTAGG

AAATAAAAGCACTTATTTTATTTTCTCCATATTTGTTTCCTCTTTGTTCCATTACTAAAAA

AATTATAATATATTAGTAACAAAAAAATTTGGGGAGGGAATGTTATTTTTAAAAAAGATGT

TTTATTTATATTTAAGTGTTTTATTGAAATTCCTCGTGAGTTTAGCTCAATTGGTATGGAC

AATACATAATATATGCAAAATTCGAGGTTCAAACCTTGAGCACCAAAAAAAAAAGTGTTTT

ATTGAAAAATATCAAGAACTTGAAAACAAATTTCCTTTTTATGAACTAGATATTGTCACAT

AGACAACTTAAGAATTGACATTTTTTTTAACACTATTGTATTTTCTGAACTCGACTTTAAC

TCGAGATCAATGAGTAAACTAAAAAAACTCGTATTATTTTATCTAAGTGTTCTTAGTGTGA

GAAAACATTATAATTGTTTGCTTAGTCTATTTCTCCCTATATTCTCACCCTCATTCTTGGT

TTGCACATAGAAAGGAGAAAAAAAAAATTAGAGAAGAGTTGAATGGAACAGAAAGAAAGAG

CAAGCAAAATAATCACTAAAAAAAATTCCCCTAAAAGAAAATCACTGAAAAATATTATCCA

CCTCTATTTTAAATAATAATAATAATGAAAACAACAATATATTTTTTTATAGATTTTCTCT

ACTAGAGGTAATTTATTTTAAACACTAAATGTCCACACCTCTAACAAAAAAAATGCATAAT

ATTATAATAGATTATTGTAAGAAAGAGTGACAAATGTTGAATTAGTTATGGAATGAAGAGT

GAGAGAGATTATACAAATAAATAAATATAAATATAAATATAAATAAAGAATAGAGAAGAAC

ATAGTACTTGAATGAAGAGATTTGGCTTGTACTTCCATTACACTTGTAAGCTTTCTACTTC

CTTCACATTCCTTCTCTTTCTCTATCTTTCCTTTTATGATATGTGTACTTTGTTATTGCTT

CATTTACTACCGACATGACTCCAACCTATTAGGGCTTTATCTGAATAATGTTAGTTATTTT

CTCTCATTTTCTTTGCTTCTTCTTATTCTTGGAACTTATAAAGGGTCTTGTCAATTCTATC

ATTCTCTTTTGTATACTTACAAATATAAATATTCTCTTTAGCTTCATTTTCATGTCTTCTT

CTACTAATAAGCTATGGAAATTGAAGTTTTTGTTTTCATGTTTATTTTTCACTTCAGCTCC

TCTTTTTTGATGTTGATGCTGAAGATTGAATAAAAACTGAGGTGGTATGTATGAGAACAAG

AACATTTTTCATTCATCTCTGTTATTTGAACATCTAAATTTTAAACAACTATCGCGACAAT

TTTTTAAGTGGTTGAAGTATTTTAAATAGGTGGTTAATTACATTAAGTAGATAGTTAATTG

ATATATCAGGTGTCAAAACTCATTAACCATCAACATAACTTCATTAGCCATGAATTTTTGA

CGTGATTAACCAAATATTGTAGATGCTCAATTACATGTAGATTTGATGGAGTTGTCCACAA

TTTCTCATATATCTATTAACTAATAACCATCTACCGAATATAATTAACCATATATTAGAAT

ATCATTAACCTCTAAAACAGTTGTCGCGAAAGTTGGTTGTACAAAAATCATTTCTATTTTT

TACGCCACTAGTTCTCTTGTTATTTCTCTTTGTTGGAAAGTTGTTGTTTTTAACTTTGAGC

TTAGTCTTATTAATTAACTTGTAATGGTAACAACATTTTTCTATCTTTGTTTGAAGGAAGT

AGTTACTGTGTCAACTTTAGTAATTAATTTGGATCATTTTGGAGGCAAAATATGGCTATGC

AACCTGTTTATTTTAAAGAACATGAAGGAAATGTCCACAATTCTGTTGGACAGTTTTCATC

TGTGACTTCAGCACCATGGTGGAGTAATGCCTATGGATCTCAACCTGTTTATGGAGGAGAC

TCTTGTGGCCAAATGAAACCTTTTTCACTAGAGCTTTCCAACTACATAGACCAACTTGCTC

CGAGTAAGAACTTAGTTCGAGGAGTTGAACAATTGTTTGATAAAGGGCATACAAACCAATT

CACCATCTTTCCAGGTACTTGTTATTCAATATAATTCCGGTTTTGAATGAATTGATTTTTG

TTAAGTTGGTTCTGGATAAACGTGCGTTAAATATAATATGATTTATGTTTGGATACGTTTA

TGTAAAAGTGAGTTGAACATAAATTGGAGACTAAATATCAATTGCAGAGGCAAAAGCTTCA

AATTCTAATTTCAAGTTAGAATCAAATCAATTCTACTCGTAAGCATCTAAATATTTCAAAA

CCAATTCTACATCTCTAGAACTAAATTTGCATTCAAACATTTTCTGCTACAGATGATTGTA

AGATGTCAGTTGATACACCAAATCATCAAGCAACCTTATCCCTGCAATCACCATTTGCTGC

CGAGCCACTTAATCGATTTGAGCTAGGTTTTAACCAGTCTATGGTAAATCTTCTTATTAAA

GCAATCCCTTTGATCATGTTTTAGTATTTTACTTTTGATGTACATAAATTCATTATGTTTT

TATTATCCTTTCCATGCTAGATCTGTGCAAAATATCCGTATATAGATCAATTTTACGGGCT

CTTCTCGACTTATGGACCTCAAATCTCGGTTTGTCTTCACCTCGGCTATTTTATTAATCTT

TGTATATATCATGCAATGTCTATAAGTACTTGAATGTGAATTTGTTTTATTTACAGTGTGT

ATGCTTACATGAGAAAGCCAGAAATCTGTTTACAACTATCTTGTTAGTATTCTTCTAAATC

TTTCAAATGTAGAGTTGATTATGAAATTTGTTGTTAATGAGTTTTCGGCTTTATGAATGAA

TTGTAAGACAAGTAATGAAACAAAAACTAGAAGAAAATTGAATTATATGATGCAATCTGTG

TTGAAGTTTTTGCATGTATTCAAGTTCTGCTTTTAGTTACTATTTGTTTATCTGCTGCAAT

ATTTCATCAATTATTATTCATGTATGTACCAATATTTAATAAATTAGGGGCGAATTATGCT

TCCGCTTAGCATGACATCTGACGACGGACCAACATACGTGAATGCTAAGCAATACCATGGA

ATCATCAGACGCAGGCATTCTCGTGCCAAAGCTGTGCTTCAGAATAAATTGATAAAGCGTA

ACAAGGTATGAAACTGAGTATTTTTCCTCACAACAATGTTCGAAAACTTGTGAAATAGTAT

ATTTTTCCTTTTACCCTTTTTATACCTATATTAAGGTTTTGTATTTGTCTTGCAGCCATAT

ATGCACGAATCGCGTCATCTACATGCAATGCGTAGACCAAGAGGATGCGGTGGTCGTTTCT

TGAACACAAAAGTTTCTGCTAATGGAAACGGTAAAAGCGGGAGTGAAGAGAACGGAAACAT

TGGTGGCCTACAGCTGCAGTCCAGTGGTTCTCAGAGTTCCGAAGTCTTACAATCTGAGGTT

GGAACTTTAAATTCGTCGAAGGAGACAAACGGAGGCAGTCCAAATGTCTCGGGGTCAGAGG

TGACTAGCATGTATACACAGGGAGGTCTTGATAGCTTTACTGTCAATCATATCGGATCTAC

TGTCCACTCTTTGGGAGACATGATCGATACTGGACACGGTATCGTCATGCCAACAAAATGG

TTTGCAGCAGCTGGCAGACAGCTGCTGGAACCATAAGTTTCGATTCAGAAAGGAAACAAGT

GGGTTTGGTACAATGTGAAATATTTTGCACCAAACTCATCCTTTCCGAGACCAGATGAAGA

AGCTATGTTTCAGTTTGTTGTGTTTACTACGACAAATTTAGTTTCGGAAGACTACTTTTCC

ATCTGGTGCTCAGGCAACTCATTCTTGGCTTATTCTCAGGAAACTCATCCTTGGCTCGTAA

TATTTAGTAGTATTGTCATTGTCTTTCCGCGCAGGCTTGCCGTGGCATGGTAGGCATGCTA

ATGACTTTGGTATTTTCATGCAGTTATAACTATGATGTGTCTTTGTTTGTTGTTAAAATAA

AAAACATGAACTCTAGCTAGGTGCATGTGTGTGTTTTTAATCTTGTCTACTAAGTTTGGTG

TTTTGTAATGGATTTCTGACTTTATGGAGCAATGTATTGTAACTCTACTAAGAAGTGTAAC

ATTTTATTTCTCCCTCTCTAAGGATTGTATAAGAACCTCTTATTTTCAGACTCTACTTAAT

CCTATTTTCTATGTCTGTATGATTTTTATATTTCTAGGACAATCAAATTGGCTTGTAGAAG

CTCAAAAGCATGCTCACAAAGTAGGTACTTATGTAGGGAACTTCGTACTCTAATAATAACT

GGTTATTAACGTTATAATTAAATGCAAAATTTGATAAGTAGTAGGGTTTGGTAAGTAATAT

AACAACACCATGTTGGCTTGGGATTCTGAATTGTGTTATTGGAGTACAACTCTTAAATAAT

GATCGTGATGTAAATAATTTAAATTTGAAGTTACTAGATAAAAAGATAACATTATGTAATA

TATTATCAAATATATACTAAAAATATAATTTTCATCTATCTCAATTGAAAAAATATCCGGG

TTTTGAGTCTATTGTCAATGACGA

>HM050

AAAAAAATAAAGGTTCTATACTCATTAAACAAAGTGCCAAATTCAGAGATACTACTTCCT

TTGGATAATTTGTATCGTCACCAACGATCTTTGAGTTATTTCAAAAATCACATTCTCAATT

TTGTGGTCCAACATTCACAAAATGGTTTGTCGGGCTTTAAGTCTAGTAATTGTGAAACATG

CATATGCATGAGGTAATTAATTGATAATCTGAAAAGGTTAAATAGTTAATCAATTGTACTT

TGGTTATTTTTAATTTGTTCTCTATAATTTTTCAGTCAAAATTGAATTTGCTTTAACCATT

TAGTGTAGTAGTATGATATTACTTGCTCTACATTCTTGGATCTAAAAGTCTAATTAGTATC

GTATAAAAAATATTCTAGTTAATTAAGATACTTTTGTTCATTCTAATTATTCTTGGATTAC

TTGTGAAACTTTTAGGGATGAACAAGAAAGTCCACTAAAGAGGCTTCTTTTTGCTAGGAGT

CACTAGTTAGTTTTTTTTTTTTTTGACAAAAGTTAACCATTTTTTTTTATCGCATGGTAGG

AAAAAAAAAATAGAGATTTCCTTTGGTACTCATAAAAAAAAAAGTTGGATGGATAATGGAT

TAATCATAACTTATTTAACATAAAATGAATTAAGAGAACATTTGATATTTGGGTTTATTTT

GTAACATAAATATTAATATCAATGTTTGAAAGAGTTTTTAACAATAATTTGTAAAAGAAAG

CATAATATATCTATCAAAATAATTGTAAAAGAAAAAATTTCTTTTGGTGGTATGACATGGA

AAAATAATTAATTCTTATTAGATGAAAGTGTAAAATTAATTTGCATTAACAAAGCATATTT

CATTAAACTCCTTTTAGTTTTTGTTGGAAAAAATAAATTTATTGTTACTCAAAAAATAAAA

GTGTACAATTAATATATTTACTCAAAAGTCCCCTAAATGGGATGTAATTATCAAGGGATGT

GAGCGGTTTAACTTTAAGTAATGAATCAACTGTTGCATAATCACTTTGAAAGAAACTTGAG

ATTCTTCAATTAGCAAAATCTTGAATAGAATAATGAGATTATTCCTAATGCTAATGATACC

CACCTCTCCTATAAGTTGATGGGGCAGAACAAATTATGCACTCAGGACAACTTTTCTCAAA

TTTTGAAAACTTACCACACGAAGAACACACTTCCACTTGCACAAGTAAGAATATAGTATAA

CAATAAATTTACCACTCATAGCTCAATGGATATTAAAATATTGCTAATCTTTTATAGACAG

ATGTTGGCAAATTAGTATAAATTAGTTATTATATTAGTTTTTAAGGTTCAATCTACTTTTT

AAAACTCTTATTAAGTTGTTTAGCTTGCCAACCAAAGACTAAATTATCTTTATTTGTTAGG

AAATAAAAGCACTTATTTTATTTTCTCCATATTTGTTTCCTCTTTGTTCCATTACTAAAAA

AATTATAATATATTAGTAACAAAAAAATTTGGGGAGGGAATGTTATTTTTAAAAAAGATGT

TTTATTTATATTTAAGTGTTTTATTGAAATTCCTCGTGAGTTTAGCTCAATTGGTATGGAC

AATACATAATATATGCAAAATTCGAGGTTCAAACCTTGAGCACCAAAAAAAAAAGTGTTTT

ATTGAAAAATATCAAGAACTTGAAAACAAATTTCCTTTTTATGAACTAGATATTGTCACAT

AGACAACTTAAGAATTGACATTTTTTTTAACACTATTGTATTTTCTGAACTCGACTTTAAC

TCGAGATCAATGAGTAAACTAAAAAAACTCGTATTATTTTATCTAAGTGTTCTTAGTGTGA

GAAAACATTATAATTGTTTGCTTAGTCTATTTCTCCCTATATTCTCACCCTCATTCTTGGT

TTGCACATAGAAAGGAGAAAAAAAAAATTAGAGAAGAGTTGAATGGAACAGAAAGAAAGAG

CAAGCAAAATAATCACTAAAAAAAATTCCCCTAAAAGAAAATCACTGAAAAATATTATCCA

CCTCTATTTTAAATAATAATAATAATGAAAACAACAATATATTTTTTTATAGATTTTCTCT

ACTAGAGGTAATTTATTTTAAACACTAAATGTCCACACCTCTAACAAAAAAAATGCATAAT

ATTATAATAGATTATTGTAAGAAAGAGTGACAAATGTTGAATTAGTTATGGAATGAAGAGT

GAGAGAGATTATACAAATAAATAAATATAAATATAAATATAAATAAAGAATAGAGAAGAAC

ATAGTACTTGAATGAAGAGATTTGGCTTGTACTTCCATTACACTTGTAAGCTTTCTACTTC

CTTCACATTCCTTCTCTTTCTCTATCTTTCCTTTTATGATATGTGTACTTTGTTATTGCTT

CATTTACTACCGACATGACTCCAACCTATTAGGGCTTTATCTGAATAATGTTAGTTATTTT

CTCTCATTTTCTTTGCTTCTTCTTATTCTTGGAACTTATAAAGGGTCTTGTCAATTCTATC

ATTCTCTTTTGTATACTTACAAATATAAATATTCTCTTTAGCTTCATTTTCATGTCTTCTT

CTACTAATAAGCTATGGAAATTGAAGTTTTTGTTTTCATGTTTATTTTTCACTTCAGCTCC

TCTTTTTTGATGTTGATGCTGAAGATTGAATAAAAACTGAGGTGGTATGTATGAGAACAAG

AACATTTTTCATTCATCTCTGTTATTTGAACATCTAAATTTTAAACAACTATCGCGACAAT

TTTTTAAGTGGTTGAAGTATTTTAAATAGGTGGTTAATTACATTAAGTAGATAGTTAATTG

ATATATCAGGTGTCAAAACTCATTAACCATCAACATAACTTCATTAGCCATGAATTTTTGA

CGTGATTAACCAAATATTGTAGATGCTCAATTACATGTAGATTTGATGGAGTTGTCCACAA

TTTCTCATATATCTATTAACTAATAACCATCTACCGAATATAATTAACCATATATTAGAAT

ATCATTAACCTCTAAAACAGTTGTCGCGAAAGTTGGTTGTACAAAAATCATTTCTATTTTT

TACGCCACTAGTTCTCTTGTTATTTCTCTTTGTTGGAAAGTTGTTGTTTTTAACTTTGAGC

TTAGTCTTATTAATTAACTTGTAATGGTAACAACATTTTTCTATCTTTGTTTGAAGGAAGT

AGTTACTGTGTCAACTTTAGTAATTAATTTGGATCATTTTGGAGGCAAAATATGGCTATGC

AACCTGTTTATTTTAAAGAACATGAAGGAAATGTCCACAATTCTGTTGGACAGTTTTCATC

TGTGACTTCAGCACCATGGTGGAGTAATGCCTATGGATCTCAACCTGTTTATGGAGGAGAC

TCTTGTGGCCAAATGAAACCTTTTTCACTAGAGCTTTCCAACTACATAGACCAACTTGCTC

CGAGTAAGAACTTAGTTCGAGGAGTTGAACAATTGTTTGATAAAGGGCATACAAACCAATT

CACCATCTTTCCAGGTACTTGTTATTCAATATAATTCCGGTTTTGAATGAATTGATTTTTG

TTAAGTTGGTTCTGGATAAACGTGCGTTAAATATAATATGATTTATGTTTGGATACGTTTA

TGTAAAAGTGAGTTGAACATAAATTGGAGACTAAATATCAATTGCAGAGGCAAAAGCTTCA

AATTCTAATTTCAAGTTAGAATCAAATCAATTCTACTCGTAAGCATCTAAATATTTCAAAA

CCAATTCTACATCTCTAGAACTAAATTTGCATTCAAACATTTTCTGCTACAGATGATTGTA

AGATGTCAGTTGATACACCAAATCATCAAGCAACCTTATCCCTGCAATCACCATTTGCTGC

CGAGCCACTTAATCGATTTGAGCTAGGTTTTAACCAGTCTATGGTAAATCTTCTTATTAAA

GCAATCCCTTTGATCATGTTTTAGTATTTTACTTTTGATGTACATAAATTCATTATGTTTT

TATTATCCTTTCCATGCTAGATCTGTGCAAAATATCCGTATATAGATCAATTTTACGGGCT

CTTCTCGACTTATGGACCTCAAATCTCGGTTTGTCTTCACCTCGGCTATTTTATTAATCTT

TGTATATATCATGCAATGTCTATAAGTACTTGAATGTGAATTTGTTTTATTTACAGTGTGT

ATGCTTACATGAGAAAGCCAGAAATCTGTTTACAACTATCTTGTTAGTATTCTTCTAAATC

TTTCAAATGTAGAGTTGATTATGAAATTTGTTGTTAATGAGTTTTCGGCTTTATGAATGAA

TTGTAAGACAAGTAATGAAACAAAAACTAGAAGAAAATTGAATTATATGATGCAATCTGTG

TTGAAGTTTTTGCATGTATTCAAGTTCTGCTTTTAGTTACTATTTGTTTATCTGCTGCAAT

ATTTCATCAATTATTATTCATGTATGTACCAATATTTAATAAATTAGGGGCGAATTATGCT

TCCGCTTAGCATGACATCTGACGACGGACCAACATACGTGAATGCTAAGCAATACCATGGA

ATCATCAGACGCAGGCATTCTCGTGCCAAAGCTGTGCTTCAGAATAAATTGATAAAGCGTA

ACAAGGTATGAAACTGAGTATTTTTCCTCACAACAATGTTCGAAAACTTGTGAAATAGTAT

ATTTTTCCTTTTACCCTTTTTATACCTATATTAAGGTTTTGTATTTGTCTTGCAGCCATAT

ATGCACGAATCGCGTCATCTACATGCAATGCGTAGACCAAGAGGATGCGGTGGTCGTTTCT

TGAACACAAAAGTTTCTGCTAATGGAAACGGTAAAAGCGGGAGTGAAGAGAACGGAAACAT

TGGTGGCCTACAGCTGCAGTCCAGTGGTTCTCAGAGTTCCGAAGTCTTACAATCTGAGGTT

GGAACTTTAAATTCGTCGAAGGAGACAAACGGAGGCAGTCCAAATGTCTCGGGGTCAGAGG

TGACTAGCATGTATACACAGGGAGGTCTTGATAGCTTTACTGTCAATCATATCGGATCTAC

TGTCCACTCTTTGGGAGACATGATCGATACTGGACACGGTATCGTCATGCCAACAAAATGG

TTTGCAGCAGCTGGCAGACAGCTGCTGGAACCATAAGTTTCGATTCAGAAAGGAAACAAGT

GGGTTTGGTACAATGTGAAATATTTTGCACCAAACTCATCCTTTCCGAGACCAGATGAAGA

AGCTATGTTTCAGTTTGTTGTGTTTACTACGACAAATTTAGTTTCGGAAGACTACTTTTCC

ATCTGGTGCTCAGGCAACTCATTCTTGGCTTATTCTCAGGAAACTCATCCTTGGCTCGTAA

TATTTAGTAGTATTGTCATTGTCTTTCCGCGCAGGCTTGCCGTGGCATGGTAGGCATGCTA

ATGACTTTGGTATTTTCATGCAGTTATAACTATGATGTGTCTTTGTTTGTTGTTAAAATAA

AAAACATGAACTCTAGCTAGGTGCATGTGTGTGTTTTTAATCTTGTCTACTAAGTTTGGTG

TTTTGTAATGGATTTCTGACTTTATGGAGCAATGTATTGTAACTCTACTAAGAAGTGTAAC

ATTTTATTTCTCCCTCTCTAAGGATTGTATAAGAACCTCTTATTTTCAGACTCTACTTAAT

CCTATTTTCTATGTCTGTATGATTTTTATATTTCTAGGACAATCAAATTGGCTTGTAGAAG

CTCAAAAGCATGCTCACAAAGTAGGTACTTATGTAGGGAACTTCGTACTCTAATAATAACT

GGTTATTAACGTTATAATTAAATGCAAAATTTGATAAGTAGTAGGGTTTGGTAAGTAATAT

AACAACACCATGTTGGCTTGGGATTCTGAATTGTGTTATTGGAGTACAACTCTTAAATAAT

GATCGTGATGTAAATAATTTAAATTTGAAGTTACTAGATAAAAAGATAACATTATGTAATA

TATTATCAAATATATACTAAAAATATAATTTTCATCTATCTCAATTGAAAAAATATCCGGG

TTTTGAGTCTATTGTCAATGACGA

>HM051

AAAAAAATAAAGGTTCTATACTCATTAAACAAAGTGCCAAATTCAGAGATACTACTTCCT

TTGGATAATTTGTATCGTCACCAACGATCTTTGAGTTATTTCAAAAATCACATTCTCAATT

TTGTGGTCCAACATTCACAAAATGGTTTGTCGGGCTTTAAGTCTAGTAATTGTGAAACATG

CATATGCATGAGGTAATTAATTGATAATCTGAAAAGGTTAAATAGTTAATCAATTGTACTT

TGGTTATTTTTAATTTGTTCTCTATAATTTTTCAGTCAAAATTGAATTTGCTTTAACCATT

TAGTGTAGTAGTATGATATTACTTGCTCTACATTCTTGGATCTAAAAGTCTAATTAGTATC

GTATAAAAAATATTCTAGTTAATTAAGATACTTTTGTTCATTCTAATTATTCTTGGATTAC

TTGTGAAACTTTTAGGGATGAACAAGAAAGTCCACTAAAGAGGCTTCTTTTTGCTAGGAGT

CACTAGTTAGTTTTTTTTTTTTTTGACAAAAGTTAACCATTTTTTTTTATCGCATGGTAGG

AAAAAAAAAATAGAGATTTCCTTTGGTACTCATAAAAAAAAAAGTTGGATGGATAATGGAT

TAATCATAACTTATTTAACATAAAATGAATTAAGAGAACATTTGATATTTGGGTTTATTTT

GTAACATAAATATTAATATCAATGTTTGAAAGAGTTTTTAACAATAATTTGTAAAAGAAAG

CATAATATATCTATCAAAATAATTGTAAAAGAAAAAATTTCTTTTGGTGGTATGACATGGA

AAAATAATTAATTCTTATTAGATGAAAGTGTAAAATTAATTTGCATTAACAAAGCATATTT

CATTAAACTCCTTTTAGTTTTTGTTGGAAAAAATAAATTTATTGTTACTCAAAAAATAAAA

GTGTACAATTAATATATTTACTCAAAAGTCCCCTAAATGGGATGTAATTATCAAGGGATGT

GAGCGGTTTAACTTTAAGTAATGAATCAACTGTTGCATAATCACTTTGAAAGAAACTTGAG

ATTCTTCAATTAGCAAAATCTTGAATAGAATAATGAGATTATTCCTAATGCTAATGATACC

CACCTCTCCTATAAGTTGATGGGGCAGAACAAATTATGCACTCAGGACAACTTTTCTCAAA

TTTTGAAAACTTACCACACGAAGAACACACTTCCACTTGCACAAGTAAGAATATAGTATAA

CAATAAATTTACCACTCATAGCTCAATGGATATTAAAATATTGCTAATCTTTTATAGACAG

ATGTTGGCAAATTAGTATAAATTAGTTATTATATTAGTTTTTAAGGTTCAATCTACTTTTT

AAAACTCTTATTAAGTTGTTTAGCTTGCCAACCAAAGACTAAATTATCTTTATTTGTTAGG

AAATAAAAGCACTTATTTTATTTTCTCCATATTTGTTTCCTCTTTGTTCCATTACTAAAAA

AATTATAATATATTAGTAACAAAAAAATTTGGGGAGGGAATGTTATTTTTAAAAAAGATGT

TTTATTTATATTTAAGTGTTTTATTGAAATTCCTCGTGAGTTTAGCTCAATTGGTATGGAC

AATACATAATATATGCAAAATTCGAGGTTCAAACCTTGAGCACCAAAAAAAAAAGTGTTTT

ATTGAAAAATATCAAGAACTTGAAAACAAATTTCCTTTTTATGAACTAGATATTGTCACAT

AGACAACTTAAGAATTGACATTTTTTTTAACACTATTGTATTTTCTGAACTCGACTTTAAC

TCGAGATCAATGAGTAAACTAAAAAAACTCGTATTATTTTATCTAAGTGTTCTTAGTGTGA

GAAAACATTATAATTGTTTGCTTAGTCTATTTCTCCCTATATTCTCACCCTCATTCTTGGT

TTGCACATAGAAAGGAGAAAAAAAAAATTAGAGAAGAGTTGAATGGAACAGAAAGAAAGAG

CAAGCAAAATAATCACTAAAAAAAATTCCCCTAAAAGAAAATCACTGAAAAATATTATCCA

CCTCTATTTTAAATAATAATAATAATGAAAACAACAATATATTTTTTTATAGATTTTCTCT

ACTAGAGGTAATTTATTTTAAACACTAAATGTCCACACCTCTAACAAAAAAAATGCATAAT

ATTATAATAGATTATTGTAAGAAAGAGTGACAAATGTTGAATTAGTTATGGAATGAAGAGT

GAGAGAGATTATACAAATAAATAAATATAAATATAAATATAAATAAAGAATAGAGAAGAAC

ATAGTACTTGAATGAAGAGATTTGGCTTGTACTTCCATTACACTTGTAAGCTTTCTACTTC

CTTCACATTCCTTCTCTTTCTCTATCTTTCCTTTTATGATATGTGTACTTTGTTATTGCTT

CATTTACTACCGACATGACTCCAACCTATTAGGGCTTTATCTGAATAATGTTAGTTATTTT

CTCTCATTTTCTTTGCTTCTTCTTATTCTTGGAACTTATAAAGGGTCTTGTCAATTCTATC

ATTCTCTTTTGTATACTTACAAATATAAATATTCTCTTTAGCTTCATTTTCATGTCTTCTT

CTACTAATAAGCTATGGAAATTGAAGTTTTTGTTTTCATGTTTATTTTTCACTTCAGCTCC

TCTTTTTTGATGTTGATGCTGAAGATTGAATAAAAACTGAGGTGGTATGTATGAGAACAAG

AACATTTTTCATTCATCTCTGTTATTTGAACATCTAAATTTTAAACAACTATCGCGACAAT

TTTTTAAGTGGTTGAAGTATTTTAAATAGGTGGTTAATTACATTAAGTAGATAGTTAATTG

ATATATCAGGTGTCAAAACTCATTAACCATCAACATAACTTCATTAGCCATGAATTTTTGA

CGTGATTAACCAAATATTGTAGATGCTCAATTACATGTAGATTTGATGGAGTTGTCCACAA

TTTCTCATATATCTATTAACTAATAACCATCTACCGAATATAATTAACCATATATTAGAAT

ATCATTAACCTCTAAAACAGTTGTCGCGAAAGTTGGTTGTACAAAAATCATTTCTATTTTT

TACGCCACTAGTTCTCTTGTTATTTCTCTTTGTTGGAAAGTTGTTGTTTTTAACTTTGAGC

TTAGTCTTATTAATTAACTTGTAATGGTAACAACATTTTTCTATCTTTGTTTGAAGGAAGT

AGTTACTGTGTCAACTTTAGTAATTAATTTGGATCATTTTGGAGGCAAAATATGGCTATGC

AACCTGTTTATTTTAAAGAACATGAAGGAAATGTCCACAATTCTGTTGGACAGTTTTCATC

TGTGACTTCAGCACCATGGTGGAGTAATGCCTATGGATCTCAACCTGTTTATGGAGGAGAC

TCTTGTGGCCAAATGAAACCTTTTTCACTAGAGCTTTCCAACTACATAGACCAACTTGCTC

CGAGTAAGAACTTAGTTCGAGGAGTTGAACAATTGTTTGATAAAGGGCATACAAACCAATT

CACCATCTTTCCAGGTACTTGTTATTCAATATAATTCCGGTTTTGAATGAATTGATTTTTG

TTAAGTTGGTTCTGGATAAACGTGCGTTAAATATAATATGATTTATGTTTGGATACGTTTA

TGTAAAAGTGAGTTGAACATAAATTGGAGACTAAATATCAATTGCAGAGGCAAAAGCTTCA

AATTCTAATTTCAAGTTAGAATCAAATCAATTCTACTCGTAAGCATCTAAATATTTCAAAA

CCAATTCTACATCTCTAGAACTAAATTTGCATTCAAACATTTTCTGCTACAGATGATTGTA

AGATGTCAGTTGATACACCAAATCATCAAGCAACCTTATCCCTGCAATCACCATTTGCTGC

CGAGCCACTTAATCGATTTGAGCTAGGTTTTAACCAGTCTATGGTAAATCTTCTTATTAAA

GCAATCCCTTTGATCATGTTTTAGTATTTTACTTTTGATGTACATAAATTCATTATGTTTT

TATTATCCTTTCCATGCTAGATCTGTGCAAAATATCCGTATATAGATCAATTTTACGGGCT

CTTCTCGACTTATGGACCTCAAATCTCGGTTTGTCTTCACCTCGGCTATTTTATTAATCTT

TGTATATATCATGCAATGTCTATAAGTACTTGAATGTGAATTTGTTTTATTTACAGTGTGT

ATGCTTACATGAGAAAGCCAGAAATCTGTTTACAACTATCTTGTTAGTATTCTTCTAAATC

TTTCAAATGTAGAGTTGATTATGAAATTTGTTGTTAATGAGTTTTCGGCTTTATGAATGAA

TTGTAAGACAAGTAATGAAACAAAAACTAGAAGAAAATTGAATTATATGATGCAATCTGTG

TTGAAGTTTTTGCATGTATTCAAGTTCTGCTTTTAGTTACTATTTGTTTATCTGCTGCAAT

ATTTCATCAATTATTATTCATGTATGTACCAATATTTAATAAATTAGGGGCGAATTATGCT

TCCGCTTAGCATGACATCTGACGACGGACCAACATACGTGAATGCTAAGCAATACCATGGA

ATCATCAGACGCAGGCATTCTCGTGCCAAAGCTGTGCTTCAGAATAAATTGATAAAGCGTA

ACAAGGTATGAAACTGAGTATTTTTCCTCACAACAATGTTCGAAAACTTGTGAAATAGTAT

ATTTTTCCTTTTACCCTTTTTATACCTATATTAAGGTTTTGTATTTGTCTTGCAGCCATAT

ATGCACGAATCGCGTCATCTACATGCAATGCGTAGACCAAGAGGATGCGGTGGTCGTTTCT

TGAACACAAAAGTTTCTGCTAATGGAAACGGTAAAAGCGGGAGTGAAGAGAACGGAAACAT

TGGTGGCCTACAGCTGCAGTCCAGTGGTTCTCAGAGTTCCGAAGTCTTACAATCTGAGGTT

GGAACTTTAAATTCGTCGAAGGAGACAAACGGAGGCAGTCCAAATGTCTCGGGGTCAGAGG

TGACTAGCATGTATACACAGGGAGGTCTTGATAGCTTTACTGTCAATCATATCGGATCTAC

TGTCCACTCTTTGGGAGACATGATCGATACTGGACACGGTATCGTCATGCCAACAAAATGG

TTTGCAGCAGCTGGCAGACAGCTGCTGGAACCATAAGTTTCGATTCAGAAAGGAAACAAGT

GGGTTTGGTACAATGTGAAATATTTTGCACCAAACTCATCCTTTCCGAGACCAGATGAAGA

AGCTATGTTTCAGTTTGTTGTGTTTACTACGACAAATTTAGTTTCGGAAGACTACTTTTCC

ATCTGGTGCTCAGGCAACTCATTCTTGGCTTATTCTCAGGAAACTCATCCTTGGCTCGTAA

TATTTAGTAGTATTGTCATTGTCTTTCCGCGCAGGCTTGCCGTGGCATGGTAGGCATGCTA

ATGACTTTGGTATTTTCATGCAGTTATAACTATGATGTGTCTTTGTTTGTTGTTAAAATAA

AAAACATGAACTCTAGCTAGGTGCATGTGTGTGTTTTTAATCTTGTCTACTAAGTTTGGTG

TTTTGTAATGGATTTCTGACTTTATGGAGCAATGTATTGTAACTCTACTAAGAAGTGTAAC

ATTTTATTTCTCCCTCTCTAAGGATTGTATAAGAACCTCTTATTTTCAGACTCTACTTAAT

CCTATTTTCTATGTCTGTATGATTTTTATATTTCTAGGACAATCAAATTGGCTTGTAGAAG

CTCAAAAGCATGCTCACAAAGTAGGTACTTATGTAGGGAACTTCGTACTCTAATAATAACT

GGTTATTAACGTTATAATTAAATGCAAAATTTGATAAGTAGTAGGGTTTGGTAAGTAATAT

AACAACACCATGTTGGCTTGGGATTCTGAATTGTGTTATTGGAGTACAACTCTTAAATAAT

GATCGTGATGTAAATAATTTAAATTTGAAGTTACTAGATAAAAAGATAACATTATGTAATA

TATTATCAAATATATACTAAAAATATAATTTTCATCTATCTCAATTGAAAAAATATCCGGG

TTTTGAGTCTATTGTCAATGACGA

>HM052

AAAAAAATAAAGGTTCTATACTCATTAAACAAAGTGCCAAATTCAGAGATACTACTTCCT

TTGGATAATTTGTATCGTCACCAACGATCTTTGAGTTATTTCAAAAATCACATTCTCAATT

TTGTGGTCCAACATTCACAAAATGGTTTGTCGGGCTTTAAGTCTAGTAATTGTGAAACATG

CATATGCATGAGGTAATTAATTGATAATCTGAAAAGGTTAAATAGTTAATCAATTGTACTT

TGGTTATTTTTAATTTGTTCTCTATAATTTTTCAGTCAAAATTGAATTTGCTTTAACCATT

TAGTGTAGTAGTATGATATTACTTGCTCTACATTCTTGGATCTAAAAGTCTAATTAGTATC

GTATAAAAAATATTCTAGTTAATTAAGATACTTTTGTTCATTCTAATTATTCTTGGATTAC

TTGTGAAACTTTTAGGGATGAACAAGAAAGTCCACTAAAGAGGCTTCTTTTTGCTAGGAGT

CACTAGTTAGTTTTTTTTTTTTTTGACAAAAGTTAACCATTTTTTTTTATCGCATGGTAGG

AAAAAAAAAATAGAGATTTCCTTTGGTACTCATAAAAAAAAAAGTTGGATGGATAATGGAT

TAATCATAACTTATTTAACATAAAATGAATTAAGAGAACATTTGATATTTGGGTTTATTTT

GTAACATAAATATTAATATCAATGTTTGAAAGAGTTTTTAACAATAATTTGTAAAAGAAAG

CATAATATATCTATCAAAATAATTGTAAAAGAAAAAATTTCTTTTGGTGGTATGACATGGA

AAAATAATTAATTCTTATTAGATGAAAGTGTAAAATTAATTTGCATTAACAAAGCATATTT

CATTAAACTCCTTTTAGTTTTTGTTGGAAAAAATAAATTTATTGTTACTCAAAAAATAAAA

GTGTACAATTAATATATTTACTCAAAAGTCCCCTAAATGGGATGTAATTATCAAGGGATGT

GAGCGGTTTAACTTTAAGTAATGAATCAACTGTTGCATAATCACTTTGAAAGAAACTTGAG

ATTCTTCAATTAGCAAAATCTTGAATAGAATAATGAGATTATTCCTAATGCTAATGATACC

CACCTCTCCTATAAGTTGATGGGGCAGAACAAATTATGCACTCAGGACAACTTTTCTCAAA

TTTTGAAAACTTACCACACGAAGAACACACTTCCACTTGCACAAGTAAGAATATAGTATAA

CAATAAATTTACCACTCATAGCTCAATGGATATTAAAATATTGCTAATCTTTTATAGACAG

ATGTTGGCAAATTAGTATAAATTAGTTATTATATTAGTTTTTAAGGTTCAATCTACTTTTT

AAAACTCTTATTAAGTTGTTTAGCTTGCCAACCAAAGACTAAATTATCTTTATTTGTTAGG

AAATAAAAGCACTTATTTTATTTTCTCCATATTTGTTTCCTCTTTGTTCCATTACTAAAAA

AATTATAATATATTAGTAACAAAAAAATTTGGGGAGGGAATGTTATTTTTAAAAAAGATGT

TTTATTTATATTTAAGTGTTTTATTGAAATTCCTCGTGAGTTTAGCTCAATTGGTATGGAC

AATACATAATATATGCAAAATTCGAGGTTCAAACCTTGAGCACCAAAAAAAAAAGTGTTTT

ATTGAAAAATATCAAGAACTTGAAAACAAATTTCCTTTTTATGAACTAGATATTGTCACAT

AGACAACTTAAGAATTGACATTTTTTTTAACACTATTGTATTTTCTGAACTCGACTTTAAC

TCGAGATCAATGAGTAAACTAAAAAAACTCGTATTATTTTATCTAAGTGTTCTTAGTGTGA

GAAAACATTATAATTGTTTGCTTAGTCTATTTCTCCCTATATTCTCACCCTCATTCTTGGT

TTGCACATAGAAAGGAGAAAAAAAAAATTAGAGAAGAGTTGAATGGAACAGAAAGAAAGAG

CAAGCAAAATAATCACTAAAAAAAATTCCCCTAAAAGAAAATCACTGAAAAATATTATCCA

CCTCTATTTTAAATAATAATAATAATGAAAACAACAATATATTTTTTTATAGATTTTCTCT

ACTAGAGGTAATTTATTTTAAACACTAAATGTCCACACCTCTAACAAAAAAAATGCATAAT

ATTATAATAGATTATTGTAAGAAAGAGTGACAAATGTTGAATTAGTTATGGAATGAAGAGT

GAGAGAGATTATACAAATAAATAAATATAAATATAAATATAAATAAAGAATAGAGAAGAAC

ATAGTACTTGAATGAAGAGATTTGGCTTGTACTTCCATTACACTTGTAAGCTTTCTACTTC

CTTCACATTCCTTCTCTTTCTCTATCTTTCCTTTTATGATATGTGTACTTTGTTATTGCTT

CATTTACTACCGACATGACTCCAACCTATTAGGGCTTTATCTGAATAATGTTAGTTATTTT

CTCTCATTTTCTTTGCTTCTTCTTATTCTTGGAACTTATAAAGGGTCTTGTCAATTCTATC

ATTCTCTTTTGTATACTTACAAATATAAATATTCTCTTTAGCTTCATTTTCATGTCTTCTT

CTACTAATAAGCTATGGAAATTGAAGTTTTTGTTTTCATGTTTATTTTTCACTTCAGCTCC

TCTTTTTTGATGTTGATGCTGAAGATTGAATAAAAACTGAGGTGGTATGTATGAGAACAAG

AACATTTTTCATTCATCTCTGTTATTTGAACATCTAAATTTTAAACAACTATCGCGACAAT

TTTTTAAGTGGTTGAAGTATTTTAAATAGGTGGTTAATTACATTAAGTAGATAGTTAATTG

ATATATCAGGTGTCAAAACTCATTAACCATCAACATAACTTCATTAGCCATGAATTTTTGA

CGTGATTAACCAAATATTGTAGATGCTCAATTACATGTAGATTTGATGGAGTTGTCCACAA

TTTCTCATATATCTATTAACTAATAACCATCTACCGAATATAATTAACCATATATTAGAAT

ATCATTAACCTCTAAAACAGTTGTCGCGAAAGTTGGTTGTACAAAAATCATTTCTATTTTT

TACGCCACTAGTTCTCTTGTTATTTCTCTTTGTTGGAAAGTTGTTGTTTTTAACTTTGAGC

TTAGTCTTATTAATTAACTTGTAATGGTAACAACATTTTTCTATCTTTGTTTGAAGGAAGT

AGTTACTGTGTCAACTTTAGTAATTAATTTGGATCATTTTGGAGGCAAAATATGGCTATGC

AACCTGTTTATTTTAAAGAACATGAAGGAAATGTCCACAATTCTGTTGGACAGTTTTCATC

TGTGACTTCAGCACCATGGTGGAGTAATGCCTATGGATCTCAACCTGTTTATGGAGGAGAC

TCTTGTGGCCAAATGAAACCTTTTTCACTAGAGCTTTCCAACTACATAGACCAACTTGCTC

CGAGTAAGAACTTAGTTCGAGGAGTTGAACAATTGTTTGATAAAGGGCATACAAACCAATT

CACCATCTTTCCAGGTACTTGTTATTCAATATAATTCCGGTTTTGAATGAATTGATTTTTG

TTAAGTTGGTTCTGGATAAACGTGCGTTAAATATAATATGATTTATGTTTGGATACGTTTA

TGTAAAAGTGAGTTGAACATAAATTGGAGACTAAATATCAATTGCAGAGGCAAAAGCTTCA

AATTCTAATTTCAAGTTAGAATCAAATCAATTCTACTCGTAAGCATCTAAATATTTCAAAA

CCAATTCTACATCTCTAGAACTAAATTTGCATTCAAACATTTTCTGCTACAGATGATTGTA

AGATGTCAGTTGATACACCAAATCATCAAGCAACCTTATCCCTGCAATCACCATTTGCTGC

CGAGCCACTTAATCGATTTGAGCTAGGTTTTAACCAGTCTATGGTAAATCTTCTTATTAAA

GCAATCCCTTTGATCATGTTTTAGTATTTTACTTTTGATGTACATAAATTCATTATGTTTT

TATTATCCTTTCCATGCTAGATCTGTGCAAAATATCCGTATATAGATCAATTTTACGGGCT

CTTCTCGACTTATGGACCTCAAATCTCGGTTTGTCTTCACCTCGGCTATTTTATTAATCTT

TGTATATATCATGCAATGTCTATAAGTACTTGAATGTGAATTTGTTTTATTTACAGTGTGT

ATGCTTACATGAGAAAGCCAGAAATCTGTTTACAACTATCTTGTTAGTATTCTTCTAAATC

TTTCAAATGTAGAGTTGATTATGAAATTTGTTGTTAATGAGTTTTCGGCTTTATGAATGAA

TTGTAAGACAAGTAATGAAACAAAAACTAGAAGAAAATTGAATTATATGATGCAATCTGTG

TTGAAGTTTTTGCATGTATTCAAGTTCTGCTTTTAGTTACTATTTGTTTATCTGCTGCAAT

ATTTCATCAATTATTATTCATGTATGTACCAATATTTAATAAATTAGGGGCGAATTATGCT

TCCGCTTAGCATGACATCTGACGACGGACCAACATACGTGAATGCTAAGCAATACCATGGA

ATCATCAGACGCAGGCATTCTCGTGCCAAAGCTGTGCTTCAGAATAAATTGATAAAGCGTA

ACAAGGTATGAAACTGAGTATTTTTCCTCACAACAATGTTCGAAAACTTGTGAAATAGTAT

ATTTTTCCTTTTACCCTTTTTATACCTATATTAAGGTTTTGTATTTGTCTTGCAGCCATAT

ATGCACGAATCGCGTCATCTACATGCAATGCGTAGACCAAGAGGATGCGGTGGTCGTTTCT

TGAACACAAAAGTTTCTGCTAATGGAAACGGTAAAAGCGGGAGTGAAGAGAACGGAAACAT

TGGTGGCCTACAGCTGCAGTCCAGTGGTTCTCAGAGTTCCGAAGTCTTACAATCTGAGGTT

GGAACTTTAAATTCGTCGAAGGAGACAAACGGAGGCAGTCCAAATGTCTCGGGGTCAGAGG

TGACTAGCATGTATACACAGGGAGGTCTTGATAGCTTTACTGTCAATCATATCGGATCTAC

TGTCCACTCTTTGGGAGACATGATCGATACTGGACACGGTATCGTCATGCCAACAAAATGG

TTTGCAGCAGCTGGCAGACAGCTGCTGGAACCATAAGTTTCGATTCAGAAAGGAAACAAGT

GGGTTTGGTACAATGTGAAATATTTTGCACCAAACTCATCCTTTCCGAGACCAGATGAAGA

AGCTATGTTTCAGTTTGTTGTGTTTACTACGACAAATTTAGTTTCGGAAGACTACTTTTCC

ATCTGGTGCTCAGGCAACTCATTCTTGGCTTATTCTCAGGAAACTCATCCTTGGCTCGTAA

TATTTAGTAGTATTGTCATTGTCTTTCCGCGCAGGCTTGCCGTGGCATGGTAGGCATGCTA

ATGACTTTGGTATTTTCATGCAGTTATAACTATGATGTGTCTTTGTTTGTTGTTAAAATAA

AAAACATGAACTCTAGCTAGGTGCATGTGTGTGTTTTTAATCTTGTCTACTAAGTTTGGTG

TTTTGTAATGGATTTCTGACTTTATGGAGCAATGTATTGTAACTCTACTAAGAAGTGTAAC

ATTTTATTTCTCCCTCTCTAAGGATTGTATAAGAACCTCTTATTTTCAGACTCTACTTAAT

CCTATTTTCTATGTCTGTATGATTTTTATATTTCTAGGACAATCAAATTGGCTTGTAGAAG

CTCAAAAGCATGCTCACAAAGTAGGTACTTATGTAGGGAACTTCGTACTCTAATAATAACT

GGTTATTAACGTTATAATTAAATGCAAAATTTGATAAGTAGTAGGGTTTGGTAAGTAATAT

AACAACACCATGTTGGCTTGGGATTCTGAATTGTGTTATTGGAGTACAACTCTTAAATAAT

GATCGTGATGTAAATAATTTAAATTTGAAGTTACTAGATAAAAAGATAACATTATGTAATA

TATTATCAAATATATACTAAAAATATAATTTTCATCTATCTCAATTGAAAAAATATCCGGG

TTTTGAGTCTATTGTCAATGACGA

>HM053

AAAAAAATAAAGGTTCTATACTCATTAAACAAAGTGCCAAATTCAGAGATACTACTTCCT

TTGGATAATTTGTATCGTCACCAACGATCTTTGAGTTATTTCAAAAATCACATTCTCAATT

TTGTGGTCCAACATTCACAAAATGGTTTGTCGGGCTTTAAGTCTAGTAATTGTGAAACATG

CATATGCATGAGGTAATTAATTGATAATCTGAAAAGGTTAAATAGTTAATCAATTGTACTT

TGGTTATTTTTAATTTGTTCTCTATAATTTTTCAGTCAAAATTGAATTTGCTTTAACCATT

TAGTGTAGTAGTATGATATTACTTGCTCTACATTCTTGGATCTAAAAGTCTAATTAGTATC

GTATAAAAAATATTCTAGTTAATTAAGATACTTTTGTTCATTCTAATTATTCTTGGATTAC

TTGTGAAACTTTTAGGGATGAACAAGAAAGTCCACTAAAGAGGCTTCTTTTTGCTAGGAGT

CACTAGTTAGTTTTTTTTTTTTTTGACAAAAGTTAACCATTTTTTTTTATCGCATGGTAGG

AAAAAAAAAATAGAGATTTCCTTTGGTACTCATAAAAAAAAAAGTTGGATGGATAATGGAT

TAATCATAACTTATTTAACATAAAATGAATTAAGAGAACATTTGATATTTGGGTTTATTTT

GTAACATAAATATTAATATCAATGTTTGAAAGAGTTTTTAACAATAATTTGTAAAAGAAAG

CATAATATATCTATCAAAATAATTGTAAAAGAAAAAATTTCTTTTGGTGGTATGACATGGA

AAAATAATTAATTCTTATTAGATGAAAGTGTAAAATTAATTTGCATTAACAAAGCATATTT

CATTAAACTCCTTTTAGTTTTTGTTGGAAAAAATAAATTTATTGTTACTCAAAAAATAAAA

GTGTACAATTAATATATTTACTCAAAAGTCCCCTAAATGGGATGTAATTATCAAGGGATGT

GAGCGGTTTAACTTTAAGTAATGAATCAACTGTTGCATAATCACTTTGAAAGAAACTTGAG

ATTCTTCAATTAGCAAAATCTTGAATAGAATAATGAGATTATTCCTAATGCTAATGATACC

CACCTCTCCTATAAGTTGATGGGGCAGAACAAATTATGCACTCAGGACAACTTTTCTCAAA

TTTTGAAAACTTACCACACGAAGAACACACTTCCACTTGCACAAGTAAGAATATAGTATAA

CAATAAATTTACCACTCATAGCTCAATGGATATTAAAATATTGCTAATCTTTTATAGACAG

ATGTTGGCAAATTAGTATAAATTAGTTATTATATTAGTTTTTAAGGTTCAATCTACTTTTT

AAAACTCTTATTAAGTTGTTTAGCTTGCCAACCAAAGACTAAATTATCTTTATTTGTTAGG

AAATAAAAGCACTTATTTTATTTTCTCCATATTTGTTTCCTCTTTGTTCCATTACTAAAAA

AATTATAATATATTAGTAACAAAAAAATTTGGGGAGGGAATGTTATTTTTAAAAAAGATGT

TTTATTTATATTTAAGTGTTTTATTGAAATTCCTCGTGAGTTTAGCTCAATTGGTATGGAC

AATACATAATATATGCAAAATTCGAGGTTCAAACCCTGAGCACCAAAAAAAAAAGTGTTTT

ATTGAAAAATATCAAGAACTTGAAAACAAATTTCCTTTTTATGAACTAGATATTGTCACAT

AGACAACTTAAGAATTGACATTTTTTTTAACACTATTGTATTTTCTGAACTCGACTTTAAC

TCGAGATCAATGAGTAAACTAAAAAAACTCGTATTATTTTATCTAAGTGTTCTTAGTGTGA

GAAAACATTATAATTGTTTGCTTAGTCTATTTCTCCCTATATTCTCACCCTCATTCTTGGT

TTGCACATAGAAAGGAGAAAAAAAAAATTAGAGAAGAGTTGAATGGAACAGAAAGAAAGAG

CAAGCAAAATAATCACTAAAAAAAATTCCCCTAAAAGAAAATCACTGAAAAATATTATCCA

CCTCTATTTTAAATAATAATAATAATGAAAACAACAATATATTTTTTTATAGATTTTCTCT

ACTAGAGGTAATTTATTTTAAACACTAAATGTCCACACCTCTAACAAAAAAAATGCATAAT

ATTATAATAGATTATTGTAAGAAAGAGTGACAAATGTTGAATTAGTTATGGAATGAAGAGT

GAGAGAGATTATACAAATAAATAAATATAAATATAAATATAAATAAAGAATAGAGAAGAAC

ATAGTACTTGAATGAAGAGATTTGGCTTGTACTTCCATTACACTTGTAAGCTTTCTACTTC

CTTCACATTCCTTCTCTTTCTCTATCTTTCCTTTTATGATATGTGTACTTTGTTATTGCTT

CATTTACTACCGACATGACTCCAACCTATTAGGGCTTTATCTGAATAATGTTAGTTATTTT

CTCTCATTTTCTTTGCTTCTTCTTATTCTTGGAACTTATAAAGGGTCTTGTCAATTCTATC

ATTCTCTTTTGTATACTTACAAATATAAATATTCTCTTTAGCTTCATTTTCATGTCTTCTT

CTACTAATAAGCTATGGAAATTGAAGTTTTTGTTTTCATGTTTATTTTTCACTTCAGCTCC

TCTTTTTTGATGTTGATGCTGAAGATTGAATAAAAACTGAGGTGGTATGTATGAGAACAAG

AACATTTTTCATTCATCTCTGTTATTTGAACATCTAAATTTTAAACAACTATCGCGACAAT

TTTTTAAGTGGTTGAAGTATTTTAAATAGGTGGTTAATTACATTAAGTAGATAGTTAATTG

ATATATCAGGTGTCAAAACTCATTAACCATCAACATAACTTCATTAGCCATGAATTTTTGA

CGTGATTAACCAAATATTGTAGATGCTCAATTACATGTAGATTTGATGGAGTTGTCCACAA

TTTCTCATATATCTATTAACTAATAACCATCTACCGAATATAATTAACCATATATTAGAAT

ATCATTAACCTCTAAAACAGTTGTCGCGAAAGTTGGTTGTACAAAAATCATTTCTATTTTT

TACGCCACTAGTTCTCTTGTTATTTCTCTTTGTTGGAAAGTTGTTGTTTTTAACTTTGAGC

TTAGTCTTATTAATTAACTTGTAATGGTAACAACATTTTTCTATCTTTGTTTGAAGGAAGT

AGTTACTGTGTCAACTTTAGTAATTAATTTGGATCATTTTGGAGGCAAAATATGGCTATGC

AACCTGTTTATTTTAAAGAACATGAAGGAAATGTCCACAATTCTGTTGGACAGTTTTCATC

TGTGACTTCAGCACCATGGTGGAGTAATGCCTATGGATCTCAACCTGTTTATGGAGGAGAC

TCTTGTGGCCAAATGAAACCTTTTTCACTAGAGCTTTCCAACTACATAGACCAACTTGCTC

CGAGTAAGAACTTAGTTCGAGGAGTTGAACAATTGTTTGATAAAGGGCATACAAACCAATT

CACCATCTTTCCAGGTACTTGTTATTCAATATAATTCCGGTTTTGAATGAATTGATTTTTG

TTAAGTTGGTTCTGGATAAACGTGCGTTAAATATAATATGATTTATGTTTGGATACGTTTA

TGTAAAAGTGAGTTGAACATAAATTGGAGACTAAATATCAATTGCAGAGGCAAAAGCTTCA

AATTCTAATTTCAAGTTAGAATCAAATCAATTCTACTCGTAAGCATCTAAATATTTCAAAA

CCAATTCTACATCTCTAGAACTAAATTTGCATTCAAACATTTTCTGCTACAGATGATTGTA

AGATGTCAGTTGATACACCAAATCATCAAGCAACCTTATCCCTGCAATCACCATTTGCTGC

CGAGCCACTTAATCGATTTGAGCTAGGTTTTAACCAGTCTATGGTAAATCTTCTTATTAAA

GCAATCCCTTTGATCATGTTTTAGTATTTTACTTTTGATGTACATAAATTCATTATGTTTT

TATTATCCTTTCCATGCTAGATCTGTGCAAAATATCCGTATATAGATCAATTTTACGGGCT

CTTCTCGACTTATGGACCTCAAATCTCGGTTTGTCTTCACCTCGGCTATTTTATTAATCTT

TGTATATATCATGCAATGTCTATAAGTACTTGAATGTGAATTTGTTTTATTTACAGTGTGT

ATGCTTACATGAGAAAGCCAGAAATCTGTTTACAACTATCTTGTTAGTATTCTTCTAAATC

TTTCAAATGTAGAGTTGATTATGAAATTTGTTGTTAATGAGTTTTCGGCTTTATGAATGAA

TTGTAAGACAAGTAATGAAACAAAAACTAGAAGAAAATTGAATTATATGATGCAATCTGTG

TTGAAGTTTTTGCATGTATTCAAGTTCTGCTTTTAGTTACTATTTGTTTATCTGCTGCAAT

ATTTCATCAATTATTATTCATGTATGTACCAATATTTAATAAATTAGGGGCGAATTATGCT

TCCGCTTAGCATGACATCTGACGACGGACCAACATACGTGAATGCTAAGCAATACCATGGA

ATCATCAGACGCAGGCATTCTCGTGCCAAAGCTGTGCTTCAGAATAAATTGATAAAGCGTA

ACAAGGTATGAAACTGAGTATTTTTCCTCACAACAATGTTCGAAAACTTGTGAAATAGTAT

ATTTTTCCTTTTACCCTTTTTATACCTATATTAAGGTTTTGTATTTGTCTTGCAGCCATAT

ATGCACGAATCGCGTCATCTACATGCAATGCGTAGACCAAGAGGATGCGGTGGTCGTTTCT

TGAACACAAAAGTTTCTGCTAATGGAAACGGTAAAAGCGGGAGTGAAGAGAACGGAAACAT

TGGTGGCCTACAGCTGCAGTCCAGTGGTTCTCAGAGTTCCGAAGTCTTACAATCTGAGGTT

GGAACTTTAAATTCGTCGAAGGAGACAAACGGAGGCAGTCCAAATGTCTCGGGGTCAGAGG

TGACTAGCATGTATACACAGGGAGGTCTTGATAGCTTTACTGTCAATCATATCGGATCTAC

TGTCCACTCTTTGGGAGACATGATCGATACTGGACACGGTATCGTCATGCCAACAAAATGG

TTTGCAGCAGCTGGCAGACAGCTGCTGGAACCATAAGTTTCGATTCAGAAAGGAAACAAGT

GGGTTTGGTACAATGTGAAATATTTTGCACCAAACTCATCCTTTCCGAGACCAGATGAAGA

AGCTATGTTTCAGTTTGTTGTGTTTACTACGACAAATTTAGTTTCGGAAGACTACTTTTCC

ATCTGGTGCTCAGGCAACTCATTCTTGGCTTATTCTCAGGAAACTCATCCTTGGCTCGTAA

TATTTAGTAGTATTGTCATTGTCTTTCCGCGCAGGCTTGCCGTGGCATGGTAGGCATGCTA

ATGACTTTGGTATTTTCATGCAGTTATAACTATGATGTGTCTTTGTTTGTTGTTAAAATAA

AAAACATGAACTCTAGCTAGGTGCATGTGTGTGTTTTTAATCTTGTCTACTAAGTTTGGTG

TTTTGTAATGGATTTCTGACTTTATGGAGCAATGTATTGTAACTCTACTAAGAAGTGTAAC

ATTTTATTTCTCCCTCTCTAAGGATTGTATAAGAACCTCTTATTTTCAGACTCTACTTAAT

CCTATTTTCTATGTCTGTATGATTTTTATATTTCTAGGACAATCAAATTGGCTTGTAGAAG

CTCAAAAGCATGCTCACAAAGTAGGTACTTATGTAGGGAACTTCGTACTCTAATAATAACT

GGTTATTAACGTTATAATTAAATGCAAAATTTGATAAGTAGTAGGGTTTGGTAAGTAATAT

AACAACACCATGTTGGCTTGGGATTCTGAATTGTGTTATTGGAGTACAACTCTTAAATAAT

GATCGTGATGTAAATAATTTAAATTTGAAGTTACTAGATAAAAAGATAACATTATGTAATA

TATTATCAAATATATACTAAAAATATAATTTTCATCTATCTCAATTGAAAAAATATCCGGG

TTTTGAGTCTATTGTCAATGACGA

>HM054

AAAAAAATAAAGGTTCTATACTCATTAAACAAAGTGCCAAATTCAGAGATACTACTTCCT

TTGGATAATTTGTATCGTCACCAACGATCTTTGAGTTATTTCAAAAATCACATTCTCAATT

TTGTGGTCCAACATTCACAAAATGGTTTGTCGGGCTTTAAGTCTAGTAATTGTGAAACATG

CATATGCATGAGGTAATTAATTGATAATCTGAAAAGGTTAAATAGTTAATCAATTGTACTT

TGGTTATTTTTAATTTGTTCTCTATAATTTTTCAGTCAAAATTGAATTTGCTTTAACCATT

TAGTGTAGTAGTATGATATTACTTGCTCTACATTCTTGGATCTAAAAGTCTAATTAGTATC

GTATAAAAAATATTCTAGTTAATTAAGATACTTTTGTTCATTCTAATTATTCTTGGATTAC

TTGTGAAACTTTTAGGGATGAACAAGAAAGTCCACTAAAGAGGCTTCTTTTTGCTAGGAGT

CACTAGTTAGTTTTTTTTTTTTTTGACAAAAGTTAACCATTTTTTTTTATCGCATGGTAGG

AAAAAAAAAATAGAGATTTCCTTTGGTACTCATAAAAAAAAAAGTTGGATGGATAATGGAT

TAATCATAACTTATTTAACATAAAATGAATTAAGAGAACATTTGATATTTGGGTTTATTTT

GTAACATAAATATTAATATCAATGTTTGAAAGAGTTTTTAACAATAATTTGTAAAAGAAAG

CATAATATATCTATCAAAATAATTGTAAAAGAAAAAATTTCTTTTGGTGGTATGACATGGA

AAAATAATTAATTCTTATTAGATGAAAGTGTAAAATTAATTTGCATTAACAAAGCATATTT

CATTAAACTCCTTTTAGTTTTTGTTGGAAAAAATAAATTTATTGTTACTCAAAAAATAAAA

GTGTACAATTAATATATTTACTCAAAAGTCCCCTAAATGGGATGTAATTATCAAGGGATGT

GAGCGGTTTAACTTTAAGTAATGAATCAACTGTTGCATAATCACTTTGAAAGAAACTTGAG

ATTCTTCAATTAGCAAAATCTTGAATAGAATAATGAGATTATTCCTAATGCTAATGATACC

CACCTCTCCTATAAGTTGATGGGGCAGAACAAATTATGCACTCAGGACAACTTTTCTCAAA

TTTTGAAAACTTACCACACGAAGAACACACTTCCACTTGCACAAGTAAGAATATAGTATAA

CAATAAATTTACCACTCATAGCTCAATGGATATTAAAATATTGCTAATCTTTTATAGACAG

ATGTTGGCAAATTAGTATAAATTAGTTATTATATTAGTTTTTAAGGTTCAATCTACTTTTT

AAAACTCTTATTAAGTTGTTTAGCTTGCCAACCAAAGACTAAATTATCTTTATTTGTTAGG

AAATAAAAGCACTTATTTTATTTTCTCCATATTTGTTTCCTCTTTGTTCCATTACTAAAAA

AATTATAATATATTAGTAACAAAAAAATTTGGGGAGGGAATGTTATTTTTAAAAAAGATGT

TTTATTTATATTTAAGTGTTTTATTGAAATTCCTCGTGAGTTTAGCTCAATTGGTATGGAC

AATACATAATATATGCAAAATTCGAGGTTCAAACCCTGAGCACCAAAAAAAAAAGTGTTTT

ATTGAAAAATATCAAGAACTTGAAAACAAATTTCCTTTTTATGAACTAGATATTGTCACAT

AGACAACTTAAGAATTGACATTTTTTTTAACACTATTGTATTTTCTGAACTCGACTTTAAC

TCGAGATCAATGAGTAAACTAAAAAAACTCGTATTATTTTATCTAAGTGTTCTTAGTGTGA

GAAAACATTATAATTGTTTGCTTAGTCTATTTCTCCCTATATTCTCACCCTCATTCTTGGT

TTGCACATAGAAAGGAGAAAAAAAAAATTAGAGAAGAGTTGAATGGAACAGAAAGAAAGAG

CAAGCAAAATAATCACTAAAAAAAATTCCCCTAAAAGAAAATCACTGAAAAATATTATCCA

CCTCTATTTTAAATAATAATAATAATGAAAACAACAATATATTTTTTTATAGATTTTCTCT

ACTAGAGGTAATTTATTTTAAACACTAAATGTCCACACCTCTAACAAAAAAAATGCATAAT

ATTATAATAGATTATTGTAAGAAAGAGTGACAAATGTTGAATTAGTTATGGAATGAAGAGT

GAGAGAGATTATACAAATAAATAAATATAAATATAAATATAAATAAAGAATAGAGAAGAAC

ATAGTACTTGAATGAAGAGATTTGGCTTGTACTTCCATTACACTTGTAAGCTTTCTACTTC

CTTCACATTCCTTCTCTTTCTCTATCTTTCCTTTTATGATATGTGTACTTTGTTATTGCTT

CATTTACTACCGACATGACTCCAACCTATTAGGGCTTTATCTGAATAATGTTAGTTATTTT

CTCTCATTTTCTTTGCTTCTTCTTATTCTTGGAACTTATAAAGGGTCTTGTCAATTCTATC

ATTCTCTTTTGTATACTTACAAATATAAATATTCTCTTTAGCTTCATTTTCATGTCTTCTT

CTACTAATAAGCTATGGAAATTGAAGTTTTTGTTTTCATGTTTATTTTTCACTTCAGCTCC

TCTTTTTTGATGTTGATGCTGAAGATTGAATAAAAACTGAGGTGGTATGTATGAGAACAAG

AACATTTTTCATTCATCTCTGTTATTTGAACATCTAAATTTTAAACAACTATCGCGACAAT

TTTTTAAGTGGTTGAAGTATTTTAAATAGGTGGTTAATTACATTAAGTAGATAGTTAATTG

ATATATCAGGTGTCAAAACTCATTAACCATCAACATAACTTCATTAGCCATGAATTTTTGA

CGTGATTAACCAAATATTGTAGATGCTCAATTACATGTAGATTTGATGGAGTTGTCCACAA

TTTCTCATATATCTATTAACTAATAACCATCTACCGAATATAATTAACCATATATTAGAAT

ATCATTAACCTCTAAAACAGTTGTCGCGAAAGTTGGTTGTACAAAAATCATTTCTATTTTT

TACGCCACTAGTTCTCTTGTTATTTCTCTTTGTTGGAAAGTTGTTGTTTTTAACTTTGAGC

TTAGTCTTATTAATTAACTTGTAATGGTAACAACATTTTTCTATCTTTGTTTGAAGGAAGT

AGTTACTGTGTCAACTTTAGTAATTAATTTGGATCATTTTGGAGGCAAAATATGGCTATGC

AACCTGTTTATTTTAAAGAACATGAAGGAAATGTCCACAATTCTGTTGGACAGTTTTCATC

TGTGACTTCAGCACCATGGTGGAGTAATGCCTATGGATCTCAACCTGTTTATGGAGGAGAC

TCTTGTGGCCAAATGAAACCTTTTTCACTAGAGCTTTCCAACTACATAGACCAACTTGCTC

CGAGTAAGAACTTAGTTCGAGGAGTTGAACAATTGTTTGATAAAGGGCATACAAACCAATT

CACCATCTTTCCAGGTACTTGTTATTCAATATAATTCCGGTTTTGAATGAATTGATTTTTG

TTAAGTTGGTTCTGGATAAACGTGCGTTAAATATAATATGATTTATGTTTGGATACGTTTA

TGTAAAAGTGAGTTGAACATAAATTGGAGACTAAATATCAATTGCAGAGGCAAAAGCTTCA

AATTCTAATTTCAAGTTAGAATCAAATCAATTCTACTCGTAAGCATCTAAATATTTCAAAA

CCAATTCTACATCTCTAGAACTAAATTTGCATTCAAACATTTTCTGCTACAGATGATTGTA

AGATGTCAGTTGATACACCAAATCATCAAGCAACCTTATCCCTGCAATCACCATTTGCTGC

CGAGCCACTTAATCGATTTGAGCTAGGTTTTAACCAGTCTATGGTAAATCTTCTTATTAAA

GCAATCCCTTTGATCATGTTTTAGTATTTTACTTTTGATGTACATAAATTCATTATGTTTT

TATTATCCTTTCCATGCTAGATCTGTGCAAAATATCCGTATATAGATCAATTTTACGGGCT

CTTCTCGACTTATGGACCTCAAATCTCGGTTTGTCTTCACCTCGGCTATTTTATTAATCTT

TGTATATATCATGCAATGTCTATAAGTACTTGAATGTGAATTTGTTTTATTTACAGTGTGT

ATGCTTACATGAGAAAGCCAGAAATCTGTTTACAACTATCTTGTTAGTATTCTTCTAAATC

TTTCAAATGTAGAGTTGATTATGAAATTTGTTGTTAATGAGTTTTCGGCTTTATGAATGAA

TTGTAAGACAAGTAATGAAACAAAAACTAGAAGAAAATTGAATTATATGATGCAATCTGTG

TTGAAGTTTTTGCATGTATTCAAGTTCTGCTTTTAGTTACTATTTGTTTATCTGCTGCAAT

ATTTCATCAATTATTATTCATGTATGTACCAATATTTAATAAATTAGGGGCGAATTATGCT

TCCGCTTAGCATGACATCTGACGACGGACCAACATACGTGAATGCTAAGCAATACCATGGA

ATCATCAGACGCAGGCATTCTCGTGCCAAAGCTGTGCTTCAGAATAAATTGATAAAGCGTA

ACAAGGTATGAAACTGAGTATTTTTCCTCACAACAATGTTCGAAAACTTGTGAAATAGTAT

ATTTTTCCTTTTACCCTTTTTATACCTATATTAAGGTTTTGTATTTGTCTTGCAGCCATAT

ATGCACGAATCGCGTCATCTACATGCAATGCGTAGACCAAGAGGATGCGGTGGTCGTTTCT

TGAACACAAAAGTTTCTGCTAATGGAAACGGTAAAAGCGGGAGTGAAGAGAACGGAAACAT

TGGTGGCCTACAGCTGCAGTCCAGTGGTTCTCAGAGTTCCGAAGTCTTACAATCTGAGGTT

GGAACTTTAAATTCGTCGAAGGAGACAAACGGAGGCAGTCCAAATGTCTCGGGGTCAGAGG

TGACTAGCATGTATACACAGGGAGGTCTTGATAGCTTTACTGTCAATCATATCGGATCTAC

TGTCCACTCTTTGGGAGACATGATCGATACTGGACACGGTATCGTCATGCCAACAAAATGG

TTTGCAGCAGCTGGCAGACAGCTGCTGGAACCATAAGTTTCGATTCAGAAAGGAAACAAGT

GGGTTTGGTACAATGTGAAATATTTTGCACCAAACTCATCCTTTCCGAGACCAGATGAAGA

AGCTATGTTTCAGTTTGTTGTGTTTACTACGACAAATTTAGTTTCGGAAGACTACTTTTCC

ATCTGGTGCTCAGGCAACTCATTCTTGGCTTATTCTCAGGAAACTCATCCTTGGCTCGTAA

TATTTAGTAGTATTGTCATTGTCTTTCCGCGCAGGCTTGCCGTGGCATGGTAGGCATGCTA

ATGACTTTGGTATTTTCATGCAGTTATAACTATGATGTGTCTTTGTTTGTTGTTAAAATAA

AAAACATGAACTCTAGCTAGGTGCATGTGTGTGTTTTTAATCTTGTCTACTAAGTTTGGTG

TTTTGTAATGGATTTCTGACTTTATGGAGCAATGTATTGTAACTCTACTAAGAAGTGTAAC

ATTTTATTTCTCCCTCTCTAAGGATTGTATAAGAACCTCTTATTTTCAGACTCTACTTAAT

CCTATTTTCTATGTCTGTATGATTTTTATATTTCTAGGACAATCAAATTGGCTTGTAGAAG

CTCAAAAGCATGCTCACAAAGTAGGTACTTATGTAGGGAACTTCGTACTCTAATAATAACT

GGTTATTAACGTTATAATTAAATGCAAAATTTGATAAGTAGTAGGGTTTGGTAAGTAATAT

AACAACACCATGTTGGCTTGGGATTCTGAATTGTGTTATTGGAGTACAACTCTTAAATAAT

GATCGTGATGTAAATAATTTAAATTTGAAGTTACTAGATAAAAAGATAACATTATGTAATA

TATTATCAAATATATACTAAAAATATAATTTTCATCTATCTCAATTGAAAAAATATCCGGG

TTTTGAGTCTATTGTCAATGACGA

>HM055

AAAAAAATAAAGGTTCTATACTCATTAAACAAAGTGCCAAATTCAGAGATACTACTTCCT

TTGGATAATTTGTATCGTCACCAACGATCTTTGAGTTATTTCAAAAATCACATTCTCAATT

TTGTGGTCCAACATTCACAAAATGGTTTGTCGGGCTTTAAGTCTAGTAATTGTGAAACATG

CATATGCATGAGGTAATTAATTGATAATCTGAAAAGGTTAAATAGTTAATCAATTGTACTT

TGGTTATTTTTAATTTGTTCTCTATAATTTTTCAGTCAAAATTGAATTTGCTTTAACCATT

TAGTGTAGTAGTATGATATTACTTGCTCTACATTCTTGGATCTAAAAGTCTAATTAGTATC

GTATAAAAAATATTCTAGTTAATTAAGATACTTTTGTTCATTCTAATTATTCTTGGATTAC

TTGTGAAACTTTTAGGGATGAACAAGAAAGTCCACTAAAGAGGCTTCTTTTTGCTAGGAGT

CACTAGTTAGTTTTTTTTTTTTTTGACAAAAGTTAACCATTTTTTTTTATCGCATGGTAGG

AAAAAAAAAATAGAGATTTCCTTTGGTACTCATAAAAAAAAAAGTTGGATGGATAATGGAT

TAATCATAACTTATTTAACATAAAATGAATTAAGAGAACATTTGATATTTGGGTTTATTTT

GTAACATAAATATTAATATCAATGTTTGAAAGAGTTTTTAACAATAATTTGTAAAAGAAAG

CATAATATATCTATCAAAATAATTGTAAAAGAAAAAATTTCTTTTGGTGGTATGACATGGA

AAAATAATTAATTCTTATTAGATGAAAGTGTAAAATTAATTTGCATTAACAAAGCATATTT

CATTAAACTCCTTTTAGTTTTTGTTGGAAAAAATAAATTTATTGTTACTCAAAAAATAAAA

GTGTACAATTAATATATTTACTCAAAAGTCCCCTAAATGGGATGTAATTATCAAGGGATGT

GAGCGGTTTAACTTTAAGTAATGAATCAACTGTTGCATAATCACTTTGAAAGAAACTTGAG

ATTCTTCAATTAGCAAAATCTTGAATAGAATAATGAGATTATTCCTAATGCTAATGATACC

CACCTCTCCTATAAGTTGATGGGGCAGAACAAATTATGCACTCAGGACAACTTTTCTCAAA

TTTTGAAAACTTACCACACGAAGAACACACTTCCACTTGCACAAGTAAGAATATAGTATAA

CAATAAATTTACCACTCATAGCTCAATGGATATTAAAATATTGCTAATCTTTTATAGACAG

ATGTTGGCAAATTAGTATAAATTAGTTATTATATTAGTTTTTAAGGTTCAATCTACTTTTT

AAAACTCTTATTAAGTTGTTTAGCTTGCCAACCAAAGACTAAATTATCTTTATTTGTTAGG

AAATAAAAGCACTTATTTTATTTTCTCCATATTTGTTTCCTCTTTGTTCCATTACTAAAAA

AATTATAATATATTAGTAACAAAAAAATTTGGGGAGGGAATGTTATTTTTAAAAAAGATGT

TTTATTTATATTTAAGTGTTTTATTGAAATTCCTCGTGAGTTTAGCTCAATTGGTATGGAC

AATACATAATATATGCAAAATTCGAGGTTCAAACCCTGAGCACCAAAAAAAAAAGTGTTTT

ATTGAAAAATATCAAGAACTTGAAAACAAATTTCCTTTTTATGAACTAGATATTGTCACAT

AGACAACTTAAGAATTGACATTTTTTTTAACACTATTGTATTTTCTGAACTCGACTTTAAC

TCGAGATCAATGAGTAAACTAAAAAAACTCGTATTATTTTATCTAAGTGTTCTTAGTGTGA

GAAAACATTATAATTGTTTGCTTAGTCTATTTCTCCCTATATTCTCACCCTCATTCTTGGT

TTGCACATAGAAAGGAGAAAAAAAAAATTAGAGAAGAGTTGAATGGAACAGAAAGAAAGAG

CAAGCAAAATAATCACTAAAAAAAATTCCCCTAAAAGAAAATCACTGAAAAATATTATCCA

CCTCTATTTTAAATAATAATAATAATGAAAACAACAATATATTTTTTTATAGATTTTCTCT

ACTAGAGGTAATTTATTTTAAACACTAAATGTCCACACCTCTAACAAAAAAAATGCATAAT

ATTATAATAGATTATTGTAAGAAAGAGTGACAAATGTTGAATTAGTTATGGAATGAAGAGT

GAGAGAGATTATACAAATAAATAAATATAAATATAAATATAAATAAAGAATAGAGAAGAAC

ATAGTACTTGAATGAAGAGATTTGGCTTGTACTTCCATTACACTTGTAAGCTTTCTACTTC

CTTCACATTCCTTCTCTTTCTCTATCTTTCCTTTTATGATATGTGTACTTTGTTATTGCTT

CATTTACTACCGACATGACTCCAACCTATTAGGGCTTTATCTGAATAATGTTAGTTATTTT

CTCTCATTTTCTTTGCTTCTTCTTATTCTTGGAACTTATAAAGGGTCTTGTCAATTCTATC

ATTCTCTTTTGTATACTTACAAATATAAATATTCTCTTTAGCTTCATTTTCATGTCTTCTT

CTACTAATAAGCTATGGAAATTGAAGTTTTTGTTTTCATGTTTATTTTTCACTTCAGCTCC

TCTTTTTTGATGTTGATGCTGAAGATTGAATAAAAACTGAGGTGGTATGTATGAGAACAAG

AACATTTTTCATTCATCTCTGTTATTTGAACATCTAAATTTTAAACAACTATCGCGACAAT

TTTTTAAGTGGTTGAAGTATTTTAAATAGGTGGTTAATTACATTAAGTAGATAGTTAATTG

ATATATCAGGTGTCAAAACTCATTAACCATCAACATAACTTCATTAGCCATGAATTTTTGA

CGTGATTAACCAAATATTGTAGATGCTCAATTACATGTAGATTTGATGGAGTTGTCCACAA

TTTCTCATATATCTATTAACTAATAACCATCTACCGAATATAATTAACCATATATTAGAAT

ATCATTAACCTCTAAAACAGTTGTCGCGAAAGTTGGTTGTACAAAAATCATTTCTATTTTT

TACGCCACTAGTTCTCTTGTTATTTCTCTTTGTTGGAAAGTTGTTGTTTTTAACTTTGAGC

TTAGTCTTATTAATTAACTTGTAATGGTAACAACATTTTTCTATCTTTGTTTGAAGGAAGT

AGTTACTGTGTCAACTTTAGTAATTAATTTGGATCATTTTGGAGGCAAAATATGGCTATGC

AACCTGTTTATTTTAAAGAACATGAAGGAAATGTCCACAATTCTGTTGGACAGTTTTCATC

TGTGACTTCAGCACCATGGTGGAGTAATGCCTATGGATCTCAACCTGTTTATGGAGGAGAC

TCTTGTGGCCAAATGAAACCTTTTTCACTAGAGCTTTCCAACTACATAGACCAACTTGCTC

CGAGTAAGAACTTAGTTCGAGGAGTTGAACAATTGTTTGATAAAGGGCATACAAACCAATT

CACCATCTTTCCAGGTACTTGTTATTCAATATAATTCCGGTTTTGAATGAATTGATTTTTG

TTAAGTTGGTTCTGGATAAACGTGCGTTAAATATAATATGATTTATGTTTGGATACGTTTA

TGTAAAAGTGAGTTGAACATAAATTGGAGACTAAATATCAATTGCAGAGGCAAAAGCTTCA

AATTCTAATTTCAAGTTAGAATCAAATCAATTCTACTCGTAAGCATCTAAATATTTCAAAA

CCAATTCTACATCTCTAGAACTAAATTTGCATTCAAACATTTTCTGCTACAGATGATTGTA

AGATGTCAGTTGATACACCAAATCATCAAGCAACCTTATCCCTGCAATCACCATTTGCTGC

CGAGCCACTTAATCGATTTGAGCTAGGTTTTAACCAGTCTATGGTAAATCTTCTTATTAAA

GCAATCCCTTTGATCATGTTTTAGTATTTTACTTTTGATGTACATAAATTCATTATGTTTT

TATTATCCTTTCCATGCTAGATCTGTGCAAAATATCCGTATATAGATCAATTTTACGGGCT

CTTCTCGACTTATGGACCTCAAATCTCGGTTTGTCTTCACCTCGGCTATTTTATTAATCTT

TGTATATATCATGCAATGTCTATAAGTACTTGAATGTGAATTTGTTTTATTTACAGTGTGT

ATGCTTACATGAGAAAGCCAGAAATCTGTTTACAACTATCTTGTTAGTATTCTTCTAAATC

TTTCAAATGTAGAGTTGATTATGAAATTTGTTGTTAATGAGTTTTCGGCTTTATGAATGAA

TTGTAAGACAAGTAATGAAACAAAAACTAGAAGAAAATTGAATTATATGATGCAATCTGTG

TTGAAGTTTTTGCATGTATTCAAGTTCTGCTTTTAGTTACTATTTGTTTATCTGCTGCAAT

ATTTCATCAATTATTATTCATGTATGTACCAATATTTAATAAATTAGGGGCGAATTATGCT

TCCGCTTAGCATGACATCTGACGACGGACCAACATACGTGAATGCTAAGCAATACCATGGA

ATCATCAGACGCAGGCATTCTCGTGCCAAAGCTGTGCTTCAGAATAAATTGATAAAGCGTA

ACAAGGTATGAAACTGAGTATTTTTCCTCACAACAATGTTCGAAAACTTGTGAAATAGTAT

ATTTTTCCTTTTACCCTTTTTATACCTATATTAAGGTTTTGTATTTGTCTTGCAGCCATAT

ATGCACGAATCGCGTCATCTACATGCAATGCGTAGACCAAGAGGATGCGGTGGTCGTTTCT

TGAACACAAAAGTTTCTGCTAATGGAAACGGTAAAAGCGGGAGTGAAGAGAACGGAAACAT

TGGTGGCCTACAGCTGCAGTCCAGTGGTTCTCAGAGTTCCGAAGTCTTACAATCTGAGGTT

GGAACTTTAAATTCGTCGAAGGAGACAAACGGAGGCAGTCCAAATGTCTCGGGGTCAGAGG

TGACTAGCATGTATACACAGGGAGGTCTTGATAGCTTTACTGTCAATCATATCGGATCTAC

TGTCCACTCTTTGGGAGACATGATCGATACTGGACACGGTATCGTCATGCCAACAAAATGG

TTTGCAGCAGCTGGCAGACAGCTGCTGGAACCATAAGTTTCGATTCAGAAAGGAAACAAGT

GGGTTTGGTACAATGTGAAATATTTTGCACCAAACTCATCCTTTCCGAGACCAGATGAAGA

AGCTATGTTTCAGTTTGTTGTGTTTACTACGACAAATTTAGTTTCGGAAGACTACTTTTCC

ATCTGGTGCTCAGGCAACTCATTCTTGGCTTATTCTCAGGAAACTCATCCTTGGCTCGTAA

TATTTAGTAGTATTGTCATTGTCTTTCCGCGCAGGCTTGCCGTGGCATGGTAGGCATGCTA

ATGACTTTGGTATTTTCATGCAGTTATAACTATGATGTGTCTTTGTTTGTTGTTAAAATAA

AAAACATGAACTCTAGCTAGGTGCATGTGTGTGTTTTTAATCTTGTCTACTAAGTTTGGTG

TTTTGTAATGGATTTCTGACTTTATGGAGCAATGTATTGTAACTCTACTAAGAAGTGTAAC

ATTTTATTTCTCCCTCTCTAAGGATTGTATAAGAACCTCTTATTTTCAGACTCTACTTAAT

CCTATTTTCTATGTCTGTATGATTTTTATATTTCTAGGACAATCAAATTGGCTTGTAGAAG

CTCAAAAGCATGCTCACAAAGTAGGTACTTATGTAGGGAACTTCGTACTCTAATAATAACT

GGTTATTAACGTTATAATTAAATGCAAAATTTGATAAGTAGTAGGGTTTGGTAAGTAATAT

AACAACACCATGTTGGCTTGGGATTCTGAATTGTGTTATTGGAGTACAACTCTTAAATAAT

GATCGTGATGTAAATAATTTAAATTTGAAGTTACTAGATAAAAAGATAACATTATGTAATA

TATTATCAAATATATACTAAAAATATAATTTTCATCTATCTCAATTGAAAAAATATCCGGG

TTTTGAGTCTATTGTCAATGACGA

>HM056

AAAAAAATAAAGGTTCTATACTCATTAAACAAAGTGCCAAATTCAGAGATACTACTTCCT

TTGGATAATTTGTATCGTCACCAACGATCTTTGAGTTATTTCAAAAATCACATTCTCAATT

TTGTGGTCCAACATTCACAAAATGGTTTGTCGGGCTTTAAGTCTAGTAATTGTGAAACATG

CATATGCATGAGGTAATTAATTGATAATCTGAAAAGGTTAAATAGTTAATCAATTGTACTT

TGGTTATTTTTAATTTGTTCTCTATAATTTTTCAGTCAAAATTGAATTTGCTTTAACCATT

TAGTGTAGTAGTATGATATTACTTGCTCTACATTCTTGGATCTAAAAGTCTAATTAGTATC

GTATAAAAAATATTCTAGTTAATTAAGATACTTTTGTTCATTCTAATTATTCTTGGATTAC

TTGTGAAACTTTTAGGGATGAACAAGAAAGTCCACTAAAGAGGCTTCTTTTTGCTAGGAGT

CACTAGTTAGTTTTTTTTTTTTTTGACAAAAGTTAACCATTTTTTTTTATCGCATGGTAGG

AAAAAAAAAATAGAGATTTCCTTTGGTACTCATAAAAAAAAAAGTTGGATGGATAATGGAT

TAATCATAACTTATTTAACATAAAATGAATTAAGAGAACATTTGATATTTGGGTTTATTTT

GTAACATAAATATTAATATCAATGTTTGAAAGAGTTTTTAACAATAATTTGTAAAAGAAAG

CATAATATATCTATCAAAATAATTGTAAAAGAAAAAATTTCTTTTGGTGGTATGACATGGA

AAAATAATTAATTCTTATTAGATGAAAGTGTAAAATTAATTTGCATTAACAAAGCATATTT

CATTAAACTCCTTTTAGTTTTTGTTGGAAAAAATAAATTTATTGTTACTCAAAAAATAAAA

GTGTACAATTAATATATTTACTCAAAAGTCCCCTAAATGGGATGTAATTATCAAGGGATGT

GAGCGGTTTAACTTTAAGTAATGAATCAACTGTTGCATAATCACTTTGAAAGAAACTTGAG

ATTCTTCAATTAGCAAAATCTTGAATAGAATAATGAGATTATTCCTAATGCTAATGATACC

CACCTCTCCTATAAGTTGATGGGGCAGAACAAATTATGCACTCAGGACAACTTTTCTCAAA

TTTTGAAAACTTACCACACGAAGAACACACTTCCACTTGCACAAGTAAGAATATAGTATAA

CAATAAATTTACCACTCATAGCTCAATGGATATTAAAATATTGCTAATCTTTTATAGACAG

ATGTTGGCAAATTAGTATAAATTAGTTATTATATTAGTTTTTAAGGTTCAATCTACTTTTT

AAAACTCTTATTAAGTTGTTTAGCTTGCCAACCAAAGACTAAATTATCTTTATTTGTTAGG

AAATAAAAGCACTTATTTTATTTTCTCCATATTTGTTTCCTCTTTGTTCCATTACTAAAAA

AATTATAATATATTAGTAACAAAAAAATTTGGGGAGGGAATGTTATTTTTAAAAAAGATGT

TTTATTTATATTTAAGTGTTTTATTGAAATTCCTCGTGAGTTTAGCTCAATTGGTATGGAC

AATACATAATATATGCAAAATTCGAGGTTCAAACCCTGAGCACCAAAAAAAAAAGTGTTTT

ATTGAAAAATATCAAGAACTTGAAAACAAATTTCCTTTTTATGAACTAGATATTGTCACAT

AGACAACTTAAGAATTGACATTTTTTTTAACACTATTGTATTTTCTGAACTCGACTTTAAC

TCGAGATCAATGAGTAAACTAAAAAAACTCGTATTATTTTATCTAAGTGTTCTTAGTGTGA

GAAAACATTATAATTGTTTGCTTAGTCTATTTCTCCCTATATTCTCACCCTCATTCTTGGT

TTGCACATAGAAAGGAGAAAAAAAAAATTAGAGAAGAGTTGAATGGAACAGAAAGAAAGAG

CAAGCAAAATAATCACTAAAAAAAATTCCCCTAAAAGAAAATCACTGAAAAATATTATCCA

CCTCTATTTTAAATAATAATAATAATGAAAACAACAATATATTTTTTTATAGATTTTCTCT

ACTAGAGGTAATTTATTTTAAACACTAAATGTCCACACCTCTAACAAAAAAAATGCATAAT

ATTATAATAGATTATTGTAAGAAAGAGTGACAAATGTTGAATTAGTTATGGAATGAAGAGT

GAGAGAGATTATACAAATAAATAAATATAAATATAAATATAAATAAAGAATAGAGAAGAAC

ATAGTACTTGAATGAAGAGATTTGGCTTGTACTTCCATTACACTTGTAAGCTTTCTACTTC

CTTCACATTCCTTCTCTTTCTCTATCTTTCCTTTTATGATATGTGTACTTTGTTATTGCTT

CATTTACTACCGACATGACTCCAACCTATTAGGGCTTTATCTGAATAATGTTAGTTATTTT

CTCTCATTTTCTTTGCTTCTTCTTATTCTTGGAACTTATAAAGGGTCTTGTCAATTCTATC

ATTCTCTTTTGTATACTTACAAATATAAATATTCTCTTTAGCTTCATTTTCATGTCTTCTT

CTACTAATAAGCTATGGAAATTGAAGTTTTTGTTTTCATGTTTATTTTTCACTTCAGCTCC

TCTTTTTTGATGTTGATGCTGAAGATTGAATAAAAACTGAGGTGGTATGTATGAGAACAAG

AACATTTTTCATTCATCTCTGTTATTTGAACATCTAAATTTTAAACAACTATCGCGACAAT

TTTTTAAGTGGTTGAAGTATTTTAAATAGGTGGTTAATTACATTAAGTAGATAGTTAATTG

ATATATCAGGTGTCAAAACTCATTAACCATCAACATAACTTCATTAGCCATGAATTTTTGA

CGTGATTAACCAAATATTGTAGATGCTCAATTACATGTAGATTTGATGGAGTTGTCCACAA

TTTCTCATATATCTATTAACTAATAACCATCTACCGAATATAATTAACCATATATTAGAAT

ATCATTAACCTCTAAAACAGTTGTCGCGAAAGTTGGTTGTACAAAAATCATTTCTATTTTT

TACGCCACTAGTTCTCTTGTTATTTCTCTTTGTTGGAAAGTTGTTGTTTTTAACTTTGAGC

TTAGTCTTATTAATTAACTTGTAATGGTAACAACATTTTTCTATCTTTGTTTGAAGGAAGT

AGTTACTGTGTCAACTTTAGTAATTAATTTGGATCATTTTGGAGGCAAAATATGGCTATGC

AACCTGTTTATTTTAAAGAACATGAAGGAAATGTCCACAATTCTGTTGGACAGTTTTCATC

TGTGACTTCAGCACCATGGTGGAGTAATGCCTATGGATCTCAACCTGTTTATGGAGGAGAC

TCTTGTGGCCAAATGAAACCTTTTTCACTAGAGCTTTCCAACTACATAGACCAACTTGCTC

CGAGTAAGAACTTAGTTCGAGGAGTTGAACAATTGTTTGATAAAGGGCATACAAACCAATT

CACCATCTTTCCAGGTACTTGTTATTCAATATAATTCCGGTTTTGAATGAATTGATTTTTG

TTAAGTTGGTTCTGGATAAACGTGCGTTAAATATAATATGATTTATGTTTGGATACGTTTA

TGTAAAAGTGAGTTGAACATAAATTGGAGACTAAATATCAATTGCAGAGGCAAAAGCTTCA

AATTCTAATTTCAAGTTAGAATCAAATCAATTCTACTCGTAAGCATCTAAATATTTCAAAA

CCAATTCTACATCTCTAGAACTAAATTTGCATTCAAACATTTTCTGCTACAGATGATTGTA

AGATGTCAGTTGATACACCAAATCATCAAGCAACCTTATCCCTGCAATCACCATTTGCTGC

CGAGCCACTTAATCGATTTGAGCTAGGTTTTAACCAGTCTATGGTAAATCTTCTTATTAAA

GCAATCCCTTTGATCATGTTTTAGTATTTTACTTTTGATGTACATAAATTCATTATGTTTT

TATTATCCTTTCCATGCTAGATCTGTGCAAAATATCCGTATATAGATCAATTTTACGGGCT

CTTCTCGACTTATGGACCTCAAATCTCGGTTTGTCTTCACCTCGGCTATTTTATTAATCTT

TGTATATATCATGCAATGTCTATAAGTACTTGAATGTGAATTTGTTTTATTTACAGTGTGT

ATGCTTACATGAGAAAGCCAGAAATCTGTTTACAACTATCTTGTTAGTATTCTTCTAAATC

TTTCAAATGTAGAGTTGATTATGAAATTTGTTGTTAATGAGTTTTCGGCTTTATGAATGAA

TTGTAAGACAAGTAATGAAACAAAAACTAGAAGAAAATTGAATTATATGATGCAATCTGTG

TTGAAGTTTTTGCATGTATTCAAGTTCTGCTTTTAGTTACTATTTGTTTATCTGCTGCAAT

ATTTCATCAATTATTATTCATGTATGTACCAATATTTAATAAATTAGGGGCGAATTATGCT

TCCGCTTAGCATGACATCTGACGACGGACCAACATACGTGAATGCTAAGCAATACCATGGA

ATCATCAGACGCAGGCATTCTCGTGCCAAAGCTGTGCTTCAGAATAAATTGATAAAGCGTA

ACAAGGTATGAAACTGAGTATTTTTCCTCACAACAATGTTCGAAAACTTGTGAAATAGTAT

ATTTTTCCTTTTACCCTTTTTATACTTATATTAAGGTTTTGTATTTGTCTTGCAGCCATAT

ATGCACGAATCGCGTCATCTACATGCAATGCGTAGACCAAGAGGATGCGGTGGTCGTTTCT

TGAACACAAAAGTTTCTGCTAATGGAAACGGTAAAAGCGGGAGTGAAGAGAACGGAAACAT

TGGTGGCCTACAGCTGCAGTCCAGTGGTTCTCAGAGTTCCGAAGTCTTACAATCTGAGGTT

GGAACTTTAAATTCGTCGAAGGAGACAAACGGAGGCAGTCCAAATGTCTCGGGGTCAGAGG

TGACTAGCATGTATACACAGGGAGGTCTTGATAGCTTTACTGTCAATCATATCGGATCTAC

TGTCCACTCTTTGGGAGACATGATCGATACTGGACACGGTATCGTCATGCCAACAAAATGG

TTTGCAGCAGCTGGCAGACAGCTGCTGGAACCATAAGTTTCGATTCAGAAAGGAAACAAGT

GGGTTTGGTACAATGTGAAATATTTTGCACCAAACTCATCCTTTCCGAGACCAGATGAAGA

AGCTATGTTTCAGTTTGTTGTGTTTACTACGACAAATTTAGTTTCGGAAGACTACTTTTCC

ATCTGGTGCTCAGGCAACTCATTCTTGGCTTATTCTCAGGAAACTCATCCTTGGCTCGTAA

TATTTAGTAGTATTGTCATTGTCTTTCCGCGCAGGCTTGCCGTGGCATGGTAGGCATGCTA

ATGACTTTGGTATTTTCATGCAGTTATAACTATGATGTGTCTTTGTTTGTTGTTAAAATAA

AAAACATGAACTCTAGCTAGGTGCATGTGTGTGTTTTTAATCTTGTCTACTAAGTTTGGTG

TTTTGTAATGGATTTCTGACTTTATGGAGCAATGTATTGTAACTCTACTAAGAAGTGTAAC

ATTTTATTTCTCCCTCTCTAAGGATTGTATAAGAACCTCTTATTTTCAGACTCTACTTAAT

CCTATTTTCTATGTCTGTATGATTTTTATATTTCTAGGACAATCAAATTGGCTTGTAGAAG

CTCAAAAGCATGCTCACAAAGTAGGTACTTATGTAGGGAACTTCGTACTCTAATAATAACT

GGTTATTAACGTTATAATTAAATGCAAAATTTGATAAGTAGTAGGGTTTGGTAAGTAATAT

AACAACACCATGTTGGCTTGGGATTCTGAATTGTGTTATTGGAGTACAACTCTTAAATAAT

GATCGTGATGTAAATAATTTAAATTTGAAGTTACTAGATAAAAAGATAACATTATGTAATA

TATTATCAAATATATACTAAAAATATAATTTTCATCTATCTCAATTGAAAAAATATCCGGG

TTTTGAGTCTATTGTCAATGACGA

>HM057

AAAAAAATAAAGGTTCTATACTCATTAAACAAAGTGCCAAATTCAGAGATACTACTTCCT

TTGGATAATTTGTATCGTCACCAACGATCTTTGAGTTATTTCAAAAATCACATTCTCAATT

TTGTGGTCCAACATTCACAAAATGGTTTGTCGGGCTTTAAGTCTAGTAATTGTGAAACATG

CATATGCATGAGGTAATTAATTGATAATCTGAAAAGGTTAAATAGTTAATCAATTGTACTT

TGGTTATTTTTAATTTGTTCTCTATAATTTTTCAGTCAAAATTGAATTTGCTTTAACCATT

TAGTGTAGTAGTATGATATTACTTGCTCTACATTCTTGGATCTAAAAGTCTAATTAGTATC

GTATAAAAAATATTCTAGTTAATTAAGATACTTTTGTTCATTCTAATTATTCTTGGATTAC

TTGTGAAACTTTTAGGGATGAACAAGAAAGTCCACTAAAGAGGCTTCTTTTTGCTAGGAGT

CACTAGTTAGTTTTTTTTTTTTTTGACAAAAGTTAACCATTTTTTTTTATCGCATGGTAGG

AAAAAAAAAATAGAGATTTCCTTTGGTACTCATAAAAAAAAAAGTTGGATGGATAATGGAT

TAATCATAACTTATTTAACATAAAATGAATTAAGAGAACATTTGATATTTGGGTTTATTTT

GTAACATAAATATTAATATCAATGTTTGAAAGAGTTTTTAACAATAATTTGTAAAAGAAAG

CATAATATATCTATCAAAATAATTGTAAAAGAAAAAATTTCTTTTGGTGGTATGACATGGA

AAAATAATTAATTCTTATTAGATGAAAGTGTAAAATTAATTTGCATTAACAAAGCATATTT

CATTAAACTCCTTTTAGTTTTTGTTGGAAAAAATAAATTTATTGTTACTCAAAAAATAAAA

GTGTACAATTAATATATTTACTCAAAAGTCCCCTAAATGGGATGTAATTATCAAGGGATGT

GAGCGGTTTAACTTTAAGTAATGAATCAACTGTTGCATAATCACTTTGAAAGAAACTTGAG

ATTCTTCAATTAGCAAAATCTTGAATAGAATAATGAGATTATTCCTAATGCTAATGATACC

CACCTCTCCTATAAGTTGATGGGGCAGAACAAATTATGCACTCAGGACAACTTTTCTCAAA

TTTTGAAAACTTACCACACGAAGAACACACTTCCACTTGCACAAGTAAGAATATAGTATAA

CAATAAATTTACCACTCATAGCTCAATGGATATTAAAATATTGCTAATCTTTTATAGACAG

ATGTTGGCAAATTAGTATAAATTAGTTATTATATTAGTTTTTAAGGTTCAATCTACTTTTT

AAAACTCTTATTAAGTTGTTTAGCTTGCCAACCAAAGACTAAATTATCTTTATTTGTTAGG

AAATAAAAGCACTTATTTTATTTTCTCCATATTTGTTTCCTCTTTGTTCCATTACTAAAAA

AATTATAATATATTAGTAACAAAAAAATTTGGGGAGGGAATGTTATTTTTAAAAAAGATGT

TTTATTTATATTTAAGTGTTTTATTGAAATTCCTCGTGAGTTTAGCTCAATTGGTATGGAC

AATACATAATATATGCAAAATTCGAGGTTCAAACCCTGAGCACCAAAAAAAAAAGTGTTTT

ATTGAAAAATATCAAGAACTTGAAAACAAATTTCCTTTTTATGAACTAGATATTGTCACAT

AGACAACTTAAGAATTGACATTTTTTTTAACACTATTGTATTTTCTGAACTCGACTTTAAC

TCGAGATCAATGAGTAAACTAAAAAAACTCGTATTATTTTATCTAAGTGTTCTTAGTGTGA

GAAAACATTATAATTGTTTGCTTAGTCTATTTCTCCCTATATTCTCACCCTCATTCTTGGT

TTGCACATAGAAAGGAGAAAAAAAAAATTAGAGAAGAGTTGAATGGAACAGAAAGAAAGAG

CAAGCAAAATAATCACTAAAAAAAATTCCCCTAAAAGAAAATCACTGAAAAATATTATCCA

CCTCTATTTTAAATAATAATAATAATGAAAACAACAATATATTTTTTTATAGATTTTCTCT

ACTAGAGGTAATTTATTTTAAACACTAAATGTCCACACCTCTAACAAAAAAAATGCATAAT

ATTATAATAGATTATTGTAAGAAAGAGTGACAAATGTTGAATTAGTTATGGAATGAAGAGT

GAGAGAGATTATACAAATAAATAAATATAAATATAAATATAAATAAAGAATAGAGAAGAAC

ATAGTACTTGAATGAAGAGATTTGGCTTGTACTTCCATTACACTTGTAAGCTTTCTACTTC

CTTCACATTCCTTCTCTTTCTCTATCTTTCCTTTTATGATATGTGTACTTTGTTATTGCTT

CATTTACTACCGACATGACTCCAACCTATTAGGGCTTTATCTGAATAATGTTAGTTATTTT

CTCTCATTTTCTTTGCTTCTTCTTATTCTTGGAACTTATAAAGGGTCTTGTCAATTCTATC

ATTCTCTTTTGTATACTTACAAATATAAATATTCTCTTTAGCTTCATTTTCATGTCTTCTT

CTACTAATAAGCTATGGAAATTGAAGTTTTTGTTTTCATGTTTATTTTTCACTTCAGCTCC

TCTTTTTTGATGTTGATGCTGAAGATTGAATAAAAACTGAGGTGGTATGTATGAGAACAAG

AACATTTTTCATTCATCTCTGTTATTTGAACATCTAAATTTTAAACAACTATCGCGACAAT

TTTTTAAGTGGTTGAAGTATTTTAAATAGGTGGTTAATTACATTAAGTAGATAGTTAATTG

ATATATCAGGTGTCAAAACTCATTAACCATCAACATAACTTCATTAGCCATGAATTTTTGA

CGTGATTAACCAAATATTGTAGATGCTCAATTACATGTAGATTTGATGGAGTTGTCCACAA

TTTCTCATATATCTATTAACTAATAACCATCTACCGAATATAATTAACCATATATTAGAAT

ATCATTAACCTCTAAAACAGTTGTCGCGAAAGTTGGTTGTACAAAAATCATTTCTATTTTT

TACGCCACTAGTTCTCTTGTTATTTCTCTTTGTTGGAAAGTTGTTGTTTTTAACTTTGAGC

TTAGTCTTATTAATTAACTTGTAATGGTAACAACATTTTTCTATCTTTGTTTGAAGGAAGT

AGTTACTGTGTCAACTTTAGTAATTAATTTGGATCATTTTGGAGGCAAAATATGGCTATGC

AACCTGTTTATTTTAAAGAACATGAAGGAAATGTCCACAATTCTGTTGGACAGTTTTCATC

TGTGACTTCAGCACCATGGTGGAGTAATGCCTATGGATCTCAACCTGTTTATGGAGGAGAC

TCTTGTGGCCAAATGAAACCTTTTTCACTAGAGCTTTCCAACTACATAGACCAACTTGCTC

CGAGTAAGAACTTAGTTCGAGGAGTTGAACAATTGTTTGATAAAGGGCATACAAACCAATT

CACCATCTTTCCAGGTACTTGTTATTCAATATAATTCCGGTTTTGAATGAATTGATTTTTG

TTAAGTTGGTTCTGGCTAAACGTGCGTTAAATATAATATGATTTATGTTTGGATACGTTTA

TGTAAAAGTGAGTTGAACATAAATTGGAGACTAAATATCAATTGCAGAGGCAAAAGCTTCA

AATTCTAATTTCAAGTTAGAATCAAATCAATTCTACTCGTAAGCATCTAAATATTTCAAAA

CCAATTCTACATCTCTAGAACTAAATTTGCATTCAAACATTTTCTGCTACAGATGATTGTA

AGATGTCAGTTGATACACCAAATCATCAAGCAACCTTATCCCTGCAATCACCATTTGCTGC

CGAGCCACTTAATCGATTTGAGCTAGGTTTTAACCAGTCTATGGTAAATCTTCTTATTAAA

GCAATCCCTTTGATCATGTTTTAGTATTTTACTTTTGATGTACATAAATTCATTATGTTTT

TATTATCCTTTCCATGCTAGATCTGTGCAAAATATCCGTATATAGATCAATTTTACGGGCT

CTTCTCGACTTATGGACCTCAAATCTCGGTTTGTCTTCACCTCGGCTATTTTATTAATCTT

TGTATATATCATGCAATGTCTATAAGTACTTGAATGTGAATTTGTTTTATTTACAGTGTGT

ATGCTTACATGAGAAAGCCAGAAATCTGTTTACAACTATCTTGTTAGTATTCTTCTAAATC

TTTCAAATGTAGAGTTGATTATGAAATTTGTTGTTAATGAGTTTTCGGCTTTATGAATGAA

TTGTAAGACAAGTAATGAAACAAAAACTAGAAGAAAATTGAATTATATGATGCAATCTGTG

TTGAAGTTTTTGCATGTATTCAAGTTCTGCTTTTAGTTACTATTTGTTTATCTGCTGCAAT

ATTTCATCAATTATTATTCATGTATGTACCAATATTTAATAAATTAGGGGCGAATTATGCT

TCCGCTTAGCATGACATCTGACGACGGACCAACATACGTGAATGCTAAGCAATACCATGGA

ATCATCAGACGCAGGCATTCTCGTGCCAAAGCTGTGCTTCAGAATAAATTGATAAAGCGTA

ACAAGGTATGAAACTGAGTATTTTTCCTCACAACAATGTTCGAAAACTTGTGAAATAGTAT

ATTTTTCCTTTTACCCTTTTTATACTTATATTAAGGTTTTGTATTTGTCTTGCAGCCATAT

ATGCACGAATCGCGTCATCTACATGCAATGCGTAGACCAAGAGGATGCGGTGGTCGTTTCT

TGAACACAAAAGTTTCTGCTAATGGAAACGGTAAAAGCGGGAGTGAAGAGAACGGAAACAT

TGGTGGCCTACAGCTGCAGTCCAGTGGTTCTCAGAGTTCCGAAGTCTTACAATCTGAGGTT

GGAACTTTAAATTCGTCGAAGGAGACAAACGGAGGCAGTCCAAATGTCTCGGGGTCAGAGG

TGACTAGCATGTATACACAGGGAGGTCTTGATAGCTTTACTGTCAATCATATCGGATCTAC

TGTCCACTCTTTGGGAGACATGATCGATACTGGACACGGTATCGTCATGCCAACAAAATGG

TTTGCAGCAGCTGGCAGACAGCTGCTGGAACCATAAGTTTCGATTCAGAAAGGAAACAAGT

GGGTTTGGTACAATGTGAAATATTTTGCACCAAACTCATCCTTTCCGAGACCAGATGAAGA

AGCTATGTTTCAGTTTGTTGTGTTTACTACGACAAATTTAGTTTCGGAAGACTACTTTTCC

ATCTGGTGCTCAGGCAACTCATTCTTGGCTTATTCTCAGGAAACTCATCCTTGGCTCGTAA

TATTTAGTAGTATTGTCATTGTCTTTCCGCGCAGGCTTGCCGTGGCATGGTAGGCATGCTA

ATGACTTTGGTATTTTCATGCAGTTATAACTATGATGTGTCTTTGTTTGTTGTTAAAATAA

AAAACATGAACTCTAGCTAGGTGCATGTGTGTGTTTTTAATCTTGTCTACTAAGTTTGGTG

TTTTGTAATGGATTTCTGACTTTATGGAGCAATGTATTGTAACTCTACTAAGAAGTGTAAC

ATTTTATTTCTCCCTCTCTAAGGATTGTATAAGAACCTCTTATTTTCAGACTCTACTTAAT

CCTATTTTCTATGTCTGTATGATTTTTATATTTCTAGGACAATCAAATTGGCTTGTAGAAG

CTCAAAAGCATGCTCACAAAGTAGGTACTTATGTAGGGAACTTCGTACTCTAATAATAACT

GGTTATTAACGTTATAATTAAATGCAAAATTTGATAAGTAGTAGGGTTTGGTAAGTAATAT

AACAACACCATGTTGGCTTGGGATTCTGAATTGTGTTATTGGAGTACAACTCTTAAATAAT

GATCGTGATGTAAATAATTTAAATTTGAAGTTACTAGATAAAAAGATAACATTATGTAATA

TATTATCAAATATATACTAAAAATATAATTTTCATCTATCTCAATTGAAAAAATATCCGGG

TTTTGAGTCTATTGTCAATGACGA

>HM058

AAAAAAATAAAGGTTCTATACTCATTAAACAAAGTGCCAAATTCAGAGATACTACTTCCT

TTGGATAATTTGTATCGTCACCAACGATCTTTGAGTTATTTCAAAAATCACATTCTCAATT

TTGTGGTCCAACATTCACAAAATGGTTTGTCGGGCTTTAAGTCTAGTAATTGTGAAACATG

CATATGCATGAGGTAATTAATTGATAATCTGAAAAGGTTAAATAGTTAATCAATTGTACTT

TGGTTATTTTTAATTTGTTCTCTATAATTTTTCAGTCAAAATTGAATTTGCTTTAACCATT

TAGTGTAGTAGTATGATATTACTTGCTCTACATTCTTGGATCTAAAAGTCTAATTAGTATC

GTATAAAAAATATTCTAGTTAATTAAGATACTTTTGTTCATTCTAATTATTCTTGGATTAC

TTGTGAAACTTTTAGGGATGAACAAGAAAGTCCACTAAAGAGGCTTCTTTTTGCTAGGAGT

CACTAGTTAGTTTTTTTTTTTTTTGACAAAAGTTAACCATTTTTTTTTATCGCATGGTAGG

AAAAAAAAAATAGAGATTTCCTTTGGTACTCATAAAAAAAAAAGTTGGATGGATAATGGAT

TAATCATAACTTATTTAACATAAAATGAATTAAGAGAACATTTGATATTTGGGTTTATTTT

GTAACATAAATATTAATATCAATGTTTGAAAGAGTTTTTAACAATAATTTGTAAAAGAAAG

CATAATATATCTATCAAAATAATTGTAAAAGAAAAAATTTCTTTTGGTGGTATGACATGGA

AAAATAATTAATTCTTATTAGATGAAAGTGTAAAATTAATTTGCATTAACAAAGCATATTT

CATTAAACTCCTTTTAGTTTTTGTTGGAAAAAATAAATTTATTGTTACTCAAAAAATAAAA

GTGTACAATTAATATATTTACTCAAAAGTCCCCTAAATGGGATGTAATTATCAAGGGATGT

GAGCGGTTTAACTTTAAGTAATGAATCAACTGTTGCATAATCACTTTGAAAGAAACTTGAG

ATTCTTCAATTAGCAAAATCTTGAATAGAATAATGAGATTATTCCTAATGCTAATGATACC

CACCTCTCCTATAAGTTGATGGGGCAGAACAAATTATGCACTCAGGACAACTTTTCTCAAA

TTTTGAAAACTTACCACACGAAGAACACACTTCCACTTGCACAAGTAAGAATATAGTATAA

CAATAAATTTACCACTCATAGCTCAATGGATATTAAAATATTGCTAATCTTTTATAGACAG

ATGTTGGCAAATTAGTATAAATTAGTTATTATATTAGTTTTTAAGGTTCAATCTACTTTTT

AAAACTCTTATTAAGTTGTTTAGCTTGCCAACCAAAGACTAAATTATCTTTATTTGTTAGG

AAATAAAAGCACTTATTTTATTTTCTCCATATTTGTTTCCTCTTTGTTCCATTACTAAAAA

AATTATAATATATTAGTAACAAAAAAATTTGGGGAGGGAATGTTATTTTTAAAAAAGATGT

TTTATTTATATTTAAGTGTTTTATTGAAATTCCTCGTGAGTTTAGCTCAATTGGTATGGAC

AATACATAATATATGCAAAATTCGAGGTTCAAACCCTGAGCACCAAAAAAAAAAGTGTTTT

ATTGAAAAATATCAAGAACTTGAAAACAAATTTCCTTTTTATGAACTAGATATTGTCACAT

AGACAACTTAAGAATTGACATTTTTTTTAACACTATTGTATTTTCTGAACTCGACTTTAAC

TCGAGATCAATGAGTAAACTAAAAAAACTCGTATTATTTTATCTAAGTGTTCTTAGTGTGA

GAAAACATTATAATTGTTTGCTTAGTCTATTTCTCCCTATATTCTCACCCTCATTCTTGGT

TTGCACATAGAAAGGAGAAAAAAAAAATTAGAGAAGAGTTGAATGGAACAGAAAGAAAGAG

CAAGCAAAATAATCACTAAAAAAAATTCCCCTAAAAGAAAATCACTGAAAAATATTATCCA

CCTCTATTTTAAATAATAATAATAATGAAAACAACAATATATTTTTTTATAGATTTTCTCT

ACTAGAGGTAATTTATTTTAAACACTAAATGTCCACACCTCTAACAAAAAAAATGCATAAT

ATTATAATAGATTATTGTAAGAAAGAGTGACAAATGTTGAATTAGTTATGGAATGAAGAGT

GAGAGAGATTATACAAATAAATAAATATAAATATAAATATAAATAAAGAATAGAGAAGAAC

ATAGTACTTGAATGAAGAGATTTGGCTTGTACTTCCATTACACTTGTAAGCTTTCTACTTC

CTTCACATTCCTTCTCTTTCTCTATCTTTCCTTTTATGATATGTGTACTTTGTTATTGCTT

CATTTACTACCGACATGACTCCAACCTATTAGGGCTTTATCTGAATAATGTTAGTTATTTT

CTCTCATTTTCTTTGCTTCTTCTTATTCTTGGAACTTATAAAGGGTCTTGTCAATTCTATC

ATTCTCTTTTGTATACTTACAAATATAAATATTCTCTTTAGCTTCATTTTCATGTCTTCTT

CTACTAATAAGCTATGGAAATTGAAGTTTTTGTTTTCATGTTTATTTTTCACTTCAGCTCC

TCTTTTTTGATGTTGATGCTGAAGATTGAATAAAAACTGAGGTGGTATGTATGAGAACAAG

AACATTTTTCATTCATCTCTGTTATTTGAACATCTAAATTTTAAACAACTATCGCGACAAT

TTTTTAAGTGGTTGAAGTATTTTAAATAGGTGGTTAATTACATTAAGTAGATAGTTAATTG

ATATATCAGGTGTCAAAACTCATTAACCATCAACATAACTTCATTAGCCATGAATTTTTGA

CGTGATTAACCAAATATTGTAGATGCTCAATTACATGTAGATTTGATGGAGTTGTCCACAA

TTTCTCATATATCTATTAACTAATAACCATCTACCGAATATAATTAACCATATATTAGAAT

ATCATTAACCTCTAAAACAGTTGTCGCGAAAGTTGGTTGTACAAAAATCATTTCTATTTTT

TACGCCACTAGTTCTCTTGTTATTTCTCTTTGTTGGAAAGTTGTTGTTTTTAACTTTGAGC

TTAGTCTTATTAATTAACTTGTAATGGTAACAACATTTTTCTATCTTTGTTTGAAGGAAGT

AGTTACTGTGTCAACTTTAGTAATTAATTTGGATCATTTTGGAGGCAAAATATGGCTATGC

AACCTGTTTATTTTAAAGAACATGAAGGAAATGTCCACAATTCTGTTGGACAGTTTTCATC

TGTGACTTCAGCACCATGGTGGAGTAATGCCTATGGATCTCAACCTGTTTATGGAGGAGAC

TCTTGTGGCCAAATGAAACCTTTTTCACTAGAGCTTTCCAACTACATAGACCAACTTGCTC

CGAGTAAGAACTTAGTTCGAGGAGTTGAACAATTGTTTGATAAAGGGCATACAAACCAATT

CACCATCTTTCCAGGTACTTGTTATTCAATATAATTCCGGTTTTGAATGAATTGATTTTTG

TTAAGTTGGTTCTGGCTAAACGTGCGTTAAATATAATATGATTTATGTTTGGATACGTTTA

TGTAAAAGTGAGTTGAACATAAATTGGAGACTAAATATCAATTGCAGAGGCAAAAGCTTCA

AATTCTAATTTCAAGTTAGAATCAAATCAATTCTACTCGTAAGCATCTAAATATTTCAAAA

CCAATTCTACATCTCTAGAACTAAATTTGCATTCAAACATTTTCTGCTACAGATGATTGTA

AGATGTCAGTTGATACACCAAATCATCAAGCAACCTTATCCCTGCAATCACCATTTGCTGC

CGAGCCACTTAATCGATTTGAGCTAGGTTTTAACCAGTCTATGGTAAATCTTCTTATTAAA

GCAATCCCTTTGATCATGTTTTAGTATTTTACTTTTGATGTACATAAATTCATTATGTTTT

TATTATCCTTTCCATGCTAGATCTGTGCAAAATATCCGTATATAGATCAATTTTACGGGCT

CTTCTCGACTTATGGACCTCAAATCTCGGTTTGTCTTCACCTCGGCTATTTTATTAATCTT

TGTATATATCATGCAATGTCTATAAGTACTTGAATGTGAATTTGTTTTATTTACAGTGTGT

ATGCTTACATGAGAAAGCCAGAAATCTGTTTACAACTATCTTGTTAGTATTCTTCTAAATC

TTTCAAATGTAGAGTTGATTATGAAATTTGTTGTTAATGAGTTTTCGGCTTTATGAATGAA

TTGTAAGACAAGTAATGAAACAAAAACTAGAAGAAAATTGAATTATATGATGCAATCTGTG

TTGAAGTTTTTGCATGTATTCAAGTTCTGCTTTTAGTTACTATTTGTTTATCTGCTGCAAT

ATTTCATCAATTATTATTCATGTATGTACCAATATTTAATAAATTAGGGGCGAATTATGCT

TCCGCTTAGCATGACATCTGACGACGGACCAACATACGTGAATGCTAAGCAATACCATGGA

ATCATCAGACGCAGGCATTCTCGTGCCAAAGCTGTGCTTCAGAATAAATTGATAAAGCGTA

ACAAGGTATGAAACTGAGTATTTTTCCTCACAACAATGTTCGAAAACTTGTGAAATAGTAT

ATTTTTCCTTTTACCCTTTTTATACTTATATTAAGGTTTTGTATTTGTCTTGCAGCCATAT

ATGCACGAATCGCGTCATCTACATGCAATGCGTAGACCAAGAGGATGCGGTGGTCGTTTCT

TGAACACAAAAGTTTCTGCTAATGGAAACGGTAAAAGCGGGAGTGAAGAGAACGGAAACAT

TGGTGGCCTACAGCTGCAGTCCAGTGGTTCTCAGAGTTCCGAAGTCTTACAATCTGAGGTT

GGAACTTTAAATTCGTCGAAGGAGACAAACGGAGGCAGTCCAAATGTCTCGGGGTCAGAGG

TGACTAGCATGTATACACAGGGAGGTCTTGATAGCTTTACTGTCAATCATATCGGATCTAC

TGTCCACTCTTTGGGAGACATGATCGATACTGGACACGGTATCGTCATGCCAACAAAATGG

TTTGCAGCAGCTGGCAGACAGCTGCTGGAACCATAAGTTTCGATTCAGAAAGGAAACAAGT

GGGTTTGGTACAATGTGAAATATTTTGCACCAAACTCATCCTTTCCGAGACCAGATGAAGA

AGCTATGTTTCAGTTTGTTGTGTTTACTACGACAAATTTAGTTTCGGAAGACTACTTTTCC

ATCTGGTGCTCAGGCAACTCATTCTTGGCTTATTCTCAGGAAACTCATCCTTGGCTCGTAA

TATTTAGTAGTATTGTCATTGTCTTTCCGCGCAGGCTTGCCGTGGCATGGTAGGCATGCTA

ATGACTTTGGTATTTTCATGCAGTTATAACTATGATGTGTCTTTGTTTGTTGTTAAAATAA

AAAACATGAACTCTAGCTAGGTGCATGTGTGTGTTTTTAATCTTGTCTACTAAGTTTGGTG

TTTTGTAATGGATTTCTGACTTTATGGAGCAATGTATTGTAACTCTACTAAGAAGTGTAAC

ATTTTATTTCTCCCTCTCTAAGGATTGTATAAGAACCTCTTATTTTCAGACTCTACTTAAT

CCTATTTTCTATGTCTGTATGATTTTTATATTTCTAGGACAATCAAATTGGCTTGTAGAAG

CTCAAAAGCATGCTCACAAAGTAGGTACTTATGTAGGGAACTTCGTACTCTAATAATAACT

GGTTATTAACGTTATAATTAAATGCAAAATTTGATAAGTAGTAGGGTTTGGTAAGTAATAT

AACAACACCATGTTGGCTTGGGATTCTGAATTGTGTTATTGGAGTACAACTCTTAAATAAT

GATCGTGATGTAAATAATTTAAATTTGAAGTTACTAGATAAAAAGATAACATTATGTAATA

TATTATCAAATATATACTAAAAATATAATTTTCATCTATCTCAATTGAAAAAATATCCGGG

TTTTGAGTCTATTGTCAATGACGA

>HM059

AAAAAAATAAAGGTTCTATACTCATTAAACAAAGTGCCAAATTCAGAGATACTACTTCCT

TTGGATAATTTGTATCGTCACCAACGATCTTTGAGTTATTTCAAAAATCACATTCTCAATT

TTGTGGTCCAACATTCACAAAATGGTTTGTCGGGCTTTAAGTCTAGTAATTGTGAAACATG

CATATGCATGAGGTAATTAATTGATAATCTGAAAAGGTTAAATAGTTAATCAATTGTACTT

TGGTTATTTTTAATTTGTTCTCTATAATTTTTCAGTCAAAATTGAATTTGCTTTAACCATT

TAGTGTAGTAGTATGATATTACTTGCTCTACATTCTTGGATCTAAAAGTCTAATTAGTATC

GTATAAAAAATATTCTAGTTAATTAAGATACTTTTGTTCATTCTAATTATTCTTGGATTAC

TTGTGAAACTTTTAGGGATGAACAAGAAAGTCCACTAAAGAGGCTTCTTTTTGCTAGGAGT

CACTAGTTAGTTTTTTTTTTTTTTGACAAAAGTTAACCATTTTTTTTTATCGCATGGTAGG

AAAAAAAAAATAGAGATTTCCTTTGGTACTCATAAAAAAAAAAGTTGGATGGATAATGGAT

TAATCATAACTTATTTAACATAAAATGAATTAAGAGAACATTTGATATTTGGGTTTATTTT

GTAACATAAATATTAATATCAATGTTTGAAAGAGTTTTTAACAATAATTTGTAAAAGAAAG

CATAATATATCTATCAAAATAATTGTAAAAGAAAAAATTTCTTTTGGTGGTATGACATGGA

AAAATAATTAATTCTTATTAGATGAAAGTGTAAAATTAATTTGCATTAACAAAGCATATTT

CATTAAACTCCTTTTAGTTTTTGTTGGAAAAAATAAATTTATTGTTACTCAAAAAATAAAA

GTGTACAATTAATATATTTACTCAAAAGTCCCCTAAATGGGATGTAATTATCAAGGGATGT

GAGCGGTTTAACTTTAAGTAATGAATCAACTGTTGCATAATCACTTTGAAAGAAACTTGAG

ATTCTTCAATTAGCAAAATCTTGAATAGAATAATGAGATTATTCCTAATGCTAATGATACC

CACCTCTCCTATAAGTTGATGGGGCAGAACAAATTATGCACTCAGGACAACTTTTCTCAAA

TTTTGAAAACTTACCACACGAAGAACACACTTCCACTTGCACAAGTAAGAATATAGTATAA

CAATAAATTTACCACTCATAGCTCAATGGATATTAAAATATTGCTAATCTTTTATAGACAG

ATGTTGGCAAATTAGTATAAATTAGTTATTATATTAGTTTTTAAGGTTCAATCTACTTTTT

AAAACTCTTATTAAGTTGTTTAGCTTGCCAACCAAAGACTAAATTATCTTTATTTGTTAGG

AAATAAAAGCACTTATTTTATTTTCTCCATATTTGTTTCCTCTTTGTTCCATTACTAAAAA

AATTATAATATATTAGTAACAAAAAAATTTGGGGAGGGAATGTTATTTTTAAAAAAGATGT

TTTATTTATATTTAAGTGTTTTATTGAAATTCCTCGTGAGTTTAGCTCAATTGGTATGGAC

AATACATAATATATGCAAAATTCGAGGTTCAAACCCTGAGCACCAAAAAAAAAAGTGTTTT

ATTGAAAAATATCAAGAACTTGAAAACAAATTTCCTTTTTATGAACTAGATATTGTCACAT

AGACAACTTAAGAATTGACATTTTTTTTAACACTATTGTATTTTCTGAACTCGACTTTAAC

TCGAGATCAATGAGTAAACTAAAAAAACTCGTATTATTTTATCTAAGTGTTCTTAGTGTGA

GAAAACATTATAATTGTTTGCTTAGTCTATTTCTCCCTATATTCTCACCCTCATTCTTGGT

TTGCACATAGAAAGGAGAAAAAAAAAATTAGAGAAGAGTTGAATGGAACAGAAAGAAAGAG

CAAGCAAAATAATCACTAAAAAAAATTCCCCTAAAAGAAAATCACTGAAAAATATTATCCA

CCTCTATTTTAAATAATAATAATAATGAAAACAACAATATATTTTTTTATAGATTTTCTCT

ACTAGAGGTAATTTATTTTAAACACTAAATGTCCACACCTCTAACAAAAAAAATGCATAAT

ATTATAATAGATTATTGTAAGAAAGAGTGACAAATGTTGAATTAGTTATGGAATGAAGAGT

GAGAGAGATTATACAAATAAATAAATATAAATATAAATATAAATAAAGAATAGAGAAGAAC

ATAGTACTTGAATGAAGAGATTTGGCTTGTACTTCCATTACACTTGTAAGCTTTCTACTTC

CTTCACATTCCTTCTCTTTCTCTATCTTTCCTTTTATGATATGTGTACTTTGTTATTGCTT

CATTTACTACCGACATGACTCCAACCTATTAGGGCTTTATCTGAATAATGTTAGTTATTTT

CTCTCATTTTCTTTGCTTCTTCTTATTCTTGGAACTTATAAAGGGTCTTGTCAATTCTATC

ATTCTCTTTTGTATACTTACAAATATAAATATTCTCTTTAGCTTCATTTTCATGTCTTCTT

CTACTAATAAGCTATGGAAATTGAAGTTTTTGTTTTCATGTTTATTTTTCACTTCAGCTCC

TCTTTTTTGATGTTGATGCTGAAGATTGAATAAAAACTGAGGTGGTATGTATGAGAACAAG

AACATTTTTCATTCATCTCTGTTATTTGAACATCTAAATTTTAAACAACTATCGCGACAAT

TTTTTAAGTGGTTGAAGTATTTTAAATAGGTGGTTAATTACATTAAGTAGATAGTTAATTG

ATATATCAGGTGTCAAAACTCATTAACCATCAACATAACTTCATTAGCCATGAATTTTTGA

CGTGATTAACCAAATATTGTAGATGCTCAATTACATGTAGATTTGATGGAGTTGTCCACAA

TTTCTCATATATCTATTAACTAATAACCATCTACCGAATATAATTAACCATATATTAGAAT

ATCATTAACCTCTAAAACAGTTGTCGCGAAAGTTGGTTGTACAAAAATCATTTCTATTTTT

TACGCCACTAGTTCTCTTGTTATTTCTCTTTGTTGGAAAGTTGTTGTTTTTAACTTTGAGC

TTAGTCTTATTAATTAACTTGTAATGGTAACAACATTTTTCTATCTTTGTTTGAAGGAAGT

AGTTACTGTGTCAACTTTAGTAATTAATTTGGATCATTTTGGAGGCAAAATATGGCTATGC

AACCTGTTTATTTTAAAGAACATGAAGGAAATGTCCACAATTCTGTTGGACAGTTTTCATC

TGTGACTTCAGCACCATGGTGGAGTAATGCCTATGGATCTCAACCTGTTTATGGAGGAGAC

TCTTGTGGCCAAATGAAACCTTTTTCACTAGAGCTTTCCAACTACATAGACCAACTTGCTC

CGAGTAAGAACTTAGTTCGAGGAGTTGAACAATTGTTTGATAAAGGGCATACAAACCAATT

CACCATCTTTCCAGGTACTTGTTATTCAATATAATTCCGGTTTTGAATGAATTGATTTTTG

TTAAGTTGGTTCTGGCTAAACGTGCGTTAAATATAATATGATTTATGTTTGGATACGTTTA

TGTAAAAGTGAGTTGAACATAAATTGGAGACTAAATATCAATTGCAGAGGCAAAAGCTTCA

AATTCTAATTTCAAGTTAGAATCAAATCAATTCTACTCGTAAGCATCTAAATATTTCAAAA

CCAATTCTACATCTCTAGAACTAAATTTGCATTCAAACATTTTCTGCTACAGATGATTGTA

AGATGTCAGTTGATACACCAAATCATCAAGCAACCTTATCCCTGCAATCACCATTTGCTGC

CGAGCCACTTAATCGATTTGAGCTAGGTTTTAACCAGTCTATGGTAAATCTTCTTATTAAA

GCAATCCCTTTGATCATGTTTTAGTATTTTACTTTTGATGTACATAAATTCATTATGTTTT

TATTATCCTTTCCATGCTAGATCTGTGCAAAATATCCGTATATAGATCAATTTTACGGGCT

CTTCTCGACTTATGGACCTCAAATCTCGGTTTGTCTTCACCTCGGCTATTTTATTAATCTT

TGTATATATCATGCAATGTCTATAAGTACTTGAATGTGAATTTGTTTTATTTACAGTGTGT

ATGCTTACATGAGAAAGCCAGAAATCTGTTTACAACTATCTTGTTAGTATTCTTCTAAATC

TTTCAAATGTAGAGTTGATTATGAAATTTGTTGTTAATGAGTTTTCGGCTTTATGAATGAA

TTGTAAGACAAGTAATGAAACAAAAACTAGAAGAAAATTGAATTATATGATGCAATCTGTG

TTGAAGTTTTTGCATGTATTCAAGTTCTGCTTTTAGTTACTATTTGTTTATCTGCTGCAAT

ATTTCATCAATTATTATTCATGTATGTACCAATATTTAATAAATTAGGGGCGAATTATGCT

TCCGCTTAGCATGACATCTGACGACGGACCAACATACGTGAATGCTAAGCAATACCATGGA

ATCATCAGACGCAGGCATTCTCGTGCCAAAGCTGTGCTTCAGAATAAATTGATAAAGCGTA

ACAAGGTATGAAACTGAGTATTTTTCCTCACAACAATGTTCGAAAACTTGTGAAATAGTAT

ATTTTTCCTTTTACCCTTTTTATACTTATATTAAGGTTTTGTATTTGTCTTGCAGCCATAT

ATGCACGAATCGCGTCATCTACATGCAATGCGTAGACCAAGAGGATGCGGTGGTCGTTTCT

TGAACACAAAAGTTTCTGCTAATGGAAACGGTAAAAGCGGGAGTGAAGAGAACGGAAACAT

TGGTGGCCTACAGCTGCAGTCCAGTGGTTCTCAGAGTTCCGAAGTCTTACAATCTGAGGTT

GGAACTTTAAATTCGTCGAAGGAGACAAACGGAGGCAGTCCAAATGTCTCGGGGTCAGAGG

TGACTAGCATGTATACACAGGGAGGTCTTGATAGCTTTACTGTCAATCATATCGGATCTAC

TGTCCACTCTTTGGGAGACATGATCGATACTGGACACGGTATCGTCATGCCAACAAAATGG

TTTGCAGCAGCTGGCAGACAGCTGCTGGAACCATAAGTTTCGATTCAGAAAGGAAACAAGT

GGGTTTGGTACAATGTGAAATATTTTGCACCAAACTCATCCTTTCCGAGACCAGATGAAGA

AGCTATGTTTCAGTTTGTTGTGTTTACTACGACAAATTTAGTTTCGGAAGACTACTTTTCC

ATCTGGTGCTCAGGCAACTCATTCTTGGCTTATTCTCAGGAAACTCATCCTTGGCTCGTAA

TATTTAGTAGTATTGTCATTGTCTTTCCGCGCAGGCTTGCCGTGGCATGGTAGGCATGCTA

ATGACTTTGGTATTTTCATGCAGTTATAACTATGATGTGTCTTTGTTTGTTGTTAAAATAA

AAAACATGAACTCTAGCTAGGTGCATGTGTGTGTTTTTAATCTTGTCTACTAAGTTTGGTG

TTTTGTAATGGATTTCTGACTTTATGGAGCAATGTATTGTAACTCTACTAAGAAGTGTAAC

ATTTTATTTCTCCCTCTCTAAGGATTGTATAAGAACCTCTTATTTTCAGACTCTACTTAAT

CCTATTTTCTATGTCTGTATGATTTTTATATTTCTAGGACAATCAAATTGGCTTGTAGAAG

CTCAAAAGCATGCTCACAAAGTAGGTACTTATGTAGGGAACTTCGTACTCTAATAATAACT

GGTTATTAACGTTATAATTAAATGCAAAATTTGATAAGTAGTAGGGTTTGGTAAGTAATAT

AACAACACCATGTTGGCTTGGGATTCTGAATTGTGTTATTGGAGTACAACTCTTAAATAAT

GATCGTGATGTAAATAATTTAAATTTGAAGTTACTAGATAAAAAGATAACATTATGTAATA

TATTATCAAATATATACTAAAAATATAATTTTCATCTATCTCAATTGAAAAAATATCCGGG

TTTTGAGTCTATTGTCAATGACGA

>HM060

AAAAAAATAAAGGTTCTATACTCATTAAACAAAGTGCCAAATTCAGAGATACTACTTCCT

TTGGATAATTTGTATCGTCACCAACGATCTTTGAGTTATTTCAAAAATCACATTCTCAATT

TTGTGGTCCAACATTCACAAAATGGTTTGTCGGGCTTTAAGTCTAGTAATTGTGAAACATG

CATATGCATGAGGTAATTAATTGATAATCTGAAAAGGTTAAATAGTTAATCAATTGTACTT

TGGTTATTTTTAATTTGTTCTCTATAATTTTTCAGTCAAAATTGAATTTGCTTTAACCATT

TAGTGTAGTAGTATGATATTACTTGCTCTACATTCTTGGATCTAAAAGTCTAATTAGTATC

GTATAAAAAATATTCTAGTTAATTAAGATACTTTTGTTCATTCTAATTATTCTTGGATTAC

TTGTGAAACTTTTAGGGATGAACAAGAAAGTCCACTAAAGAGGCTTCTTTTTGCTAGGAGT

CACTAGTTAGTTTTTTTTTTTTTTGACAAAAGTTAACCATTTTTTTTTATCGCATGGTAGG

AAAAAAAAAATAGAGATTTCCTTTGGTACTCATAAAAAAAAAAGTTGGATGGATAATGGAT

TAATCATAACTTATTTAACATAAAATGAATTAAGAGAACATTTGATATTTGGGTTTATTTT

GTAACATAAATATTAATATCAATGTTTGAAAGAGTTTTTAACAATAATTTGTAAAAGAAAG

CATAATATATCTATCAAAATAATTGTAAAAGAAAAAATTTCTTTTGGTGGTATGACATGGA

AAAATAATTAATTCTTATTAGATGAAAGTGTAAAATTAATTTGCATTAACAAAGCATATTT

CATTAAACTCCTTTTAGTTTTTGTTGGAAAAAATAAATTTATTGTTACTCAAAAAATAAAA

GTGTACAATTAATATATTTACTCAAAAGTCCCCTAAATGGGATGTAATTATCAAGGGATGT

GAGCGGTTTAACTTTAAGTAATGAATCAACTGTTGCATAATCACTTTGAAAGAAACTTGAG

ATTCTTCAATTAGCAAAATCTTGAATAGAATAATGAGATTATTCCTAATGCTAATGATACC

CACCTCTCCTATAAGTTGATGGGGCAGAACAAATTATGCACTCAGGACAACTTTTCTCAAA

TTTTGAAAACTTACCACACGAAGAACACACTTCCACTTGCACAAGTAAGAATATAGTATAA

CAATAAATTTACCACTCATAGCTCAATGGATATTAAAATATTGCTAATCTTTTATAGACAG

ATGTTGGCAAATTAGTATAAATTAGTTATTATATTAGTTTTTAAGGTTCAATCTACTTTTT

AAAACTCTTATTAAGTTGTTTAGCTTGCCAACCAAAGACTAAATTATCTTTATTTGTTAGG

AAATAAAAGCACTTATTTTATTTTCTCCATATTTGTTTCCTCTTTGTTCCATTACTAAAAA

AATTATAATATATTAGTAACAAAAAAATTTGGGGAGGGAATGTTATTTTTAAAAAAGATGT

TTTATTTATATTTAAGTGTTTTATTGAAATTCCTCGTGAGTTTAGCTCAATTGGTATGGAC

AATACATAATATATGCAAAATTCGAGGTTCAAACCCTGAGCACCAAAAAAAAAAGTGTTTT

ATTGAAAAATATCAAGAACTTGAAAACAAATTTCCTTTTTATGAACTAGATATTGTCACAT

AGACAACTTAAGAATTGACATTTTTTTTAACACTATTGTATTTTCTGAACTCGACTTTAAC

TCGAGATCAATGAGTAAACTAAAAAAACTCGTATTATTTTATCTAAGTGTTCTTAGTGTGA

GAAAACATTATAATTGTTTGCTTAGTCTATTTCTCCCTATATTCTCACCCTCATTCTTGGT

TTGCACATAGAAAGGAGAAAAAAAAAATTAGAGAAGAGTTGAATGGAACAGAAAGAAAGAG

CAAGCAAAATAATCACTAAAAAAAATTCCCCTAAAAGAAAATCACTGAAAAATATTATCCA

CCTCTATTTTAAATAATAATAATAATGAAAACAACAATATATTTTTTTATAGATTTTCTCT

ACTAGAGGTAATTTATTTTAAACACTAAATGTCCACACCTCTAACAAAAAAAATGCATAAT

ATTATAATAGATTATTGTAAGAAAGAGTGACAAATGTTGAATTAGTTATGGAATGAAGAGT

GAGAGAGATTATACAAATAAATAAATATAAATATAAATATAAATAAAGAATAGAGAAGAAC

ATAGTACTTGAATGAAGAGATTTGGCTTGTACTTCCATTACACTTGTAAGCTTTCTACTTC

CTTCACATTCCTTCTCTTTCTCTATCTTTCCTTTTATGATATGTGTACTTTGTTATTGCTT

CATTTACTACCGACATGACTCCAACCTATTAGGGCTTTATCTGAATAATGTTAGTTATTTT

CTCTCATTTTCTTTGCTTCTTCTTATTCTTGGAACTTATAAAGGGTCTTGTCAATTCTATC

ATTCTCTTTTGTATACTTACAAATATAAATATTCTCTTTAGCTTCATTTTCATGTCTTCTT

CTACTAATAAGCTATGGAAATTGAAGTTTTTGTTTTCATGTTTATTTTTCACTTCAGCTCC

TCTTTTTTGATGTTGATGCTGAAGATTGAATAAAAACTGAGGTGGTATGTATGAGAACAAG

AACATTTTTCATTCATCTCTGTTATTTGAACATCTAAATTTTAAACAACTATCGCGACAAT

TTTTTAAGTGGTTGAAGTATTTTAAATAGGTGGTTAATTACATTAAGTAGATAGTTAATTG

ATATATCAGGTGTCAAAACTCATTAACCATCAACATAACTTCATTAGCCATGAATTTTTGA

CGTGATTAACCAAATATTGTAGATGCTCAATTACATGTAGATTTGATGGAGTTGTCCACAA

TTTCTCATATATCTATTAACTAATAACCATCTACCGAATATAATTAACCATATATTAGAAT

ATCATTAACCTCTAAAACAGTTGTCGCGAAAGTTGGTTGTACAAAAATCATTTCTATTTTT

TACGCCACTAGTTCTCTTGTTATTTCTCTTTGTTGGAAAGTTGTTGTTTTTAACTTTGAGC

TTAGTCTTATTAATTAACTTGTAATGGTAACAACATTTTTCTATCTTTGTTTGAAGGAAGT

AGTTACTGTGTCAACTTTAGTAATTAATTTGGATCATTTTGGAGGCAAAATATGGCTATGC

AACCTGTTTATTTTAAAGAACATGAAGGAAATGTCCACAATTCTGTTGGACAGTTTTCATC

TGTGACTTCAGCACCATGGTGGAGTAATGCCTATGGATCTCAACCTGTTTATGGAGGAGAC

TCTTGTGGCCAAATGAAACCTTTTTCACTAGAGCTTTCCAACTACATAGACCAACTTGCTC

CGAGTAAGAACTTAGTTCGAGGAGTTGAACAATTGTTTGATAAAGGGCATACAAACCAATT

CACCATCTTTCCAGGTACTTGTTATTCAATATAATTCCGGTTTTGAATGAATTGATTTTTG

TTAAGTTGGTTCTGGCTAAACGTGCGTTAAATATAATATGATTTATGTTTGGATACGTTTA

TGTAAAAGTGAGTTGAACATAAATTGGAGACTAAATATCAATTGCAGAGGCAAAAGCTTCA

AATTCTAATTTCAAGTTAGAATCAAATCAATTCTACTCGTAAGCATCTAAATATTTCAAAA

CCAATTCTACATCTCTAGAACTAAATTTGCATTCAAACATTTTCTGCTACAGATGATTGTA

AGATGTCAGTTGATACACCAAATCATCAAGCAACCTTATCCCTGCAATCACCATTTGCTGC

CGAGCCACTTAATCGATTTGAGCTAGGTTTTAACCAGTCTATGGTAAATCTTCTTATTAAA

GCAATCCCTTTGATCATGTTTTAGTATTTTACTTTTGATGTACATAAATTCATTATGTTTT

TATTATCCTTTCCATGCTAGATCTGTGCAAAATATCCGTATATAGATCAATTTTACGGGCT

CTTCTCGACTTATGGACCTCAAATCTCGGTTTGTCTTCACCTCGGCTATTTTATTAATCTT

TGTATATATCATGCAATGTCTATAAGTACTTGAATGTGAATTTGTTTTATTTACAGTGTGT

ATGCTTACATGAGAAAGCCAGAAATCTGTTTACAACTATCTTGTTAGTATTCTTCTAAATC

TTTCAAATGTAGAGTTGATTATGAAATTTGTTGTTAATGAGTTTTCGGCTTTATGAATGAA

TTGTAAGACAAGTAATGAAACAAAAACTAGAAGAAAATTGAATTATATGATGCAATCTGTG

TTGAAGTTTTTGCATGTATTCAAGTTCTGCTTTTAGTTACTATTTGTTTATCTGCTGCAAT

ATTTCATCAATTATTATTCATGTATGTACCAATATTTAATAAATTAGGGGCGAATTATGCT

TCCGCTTAGCATGACATCTGACGACGGACCAACATACGTGAATGCTAAGCAATACCATGGA

ATCATCAGACGCAGGCATTCTCGTGCCAAAGCTGTGCTTCAGAATAAATTGATAAAGCGTA

ACAAGGTATGAAACTGAGTATTTTTCCTCACAACAATGTTCGAAAACTTGTGAAATAGTAT

ATTTTTCCTTTTACCCTTTTTATACTTATATTAAGGTTTTGTATTTGTCTTGCAGCCATAT

ATGCACGAATCGCGTCATCTACATGCAATGCGTAGACCAAGAGGATGCGGTGGTCGTTTCT

TGAACACAAAAGTTTCTGCTAATGGAAACGGTAAAAGCGGGAGTGAAGAGAACGGAAACAT

TGGTGGCCTACAGCTGCAGTCCAGTGGTTCTCAGAGTTCCGAAGTCTTACAATCTGAGGTT

GGAACTTTAAATTCGTCGAAGGAGACAAACGGAGGCAGTCCAAATGTCTCGGGGTCAGAGG

TGACTAGCATGTATACACAGGGAGGTCTTGATAGCTTTACTGTCAATCATATCGGATCTAC

TGTCCACTCTTTGGGAGACATGATCGATACTGGACACGGTATCGTCATGCCAACAAAATGG

TTTGCAGCAGCTGGCAGACAGCTGCTGGAACCATAAGTTTCGATTCAGAAAGGAAACAAGT

GGGTTTGGTACAATGTGAAATATTTTGCACCAAACTCATCCTTTCCGAGACCAGATGAAGA

AGCTATGTTTCAGTTTGTTGTGTTTACTACGACAAATTTAGTTTCGGAAGACTACTTTTCC

ATCTGGTGCTCAGGCAACTCATTCTTGGCTTATTCTCAGGAAACTCATCCTTGGCTCGTAA

TATTTAGTAGTATTGTCATTGTCTTTCCGCGCAGGCTTGCCGTGGCATGGTAGGCATGCTA

ATGACTTTGGTATTTTCATGCAGTTATAACTATGATGTGTCTTTGTTTGTTGTTAAAATAA

AAAACATGAACTCTAGCTAGGTGCATGTGTGTGTTTTTAATCTTGTCTACTAAGTTTGGTG

TTTTGTAATGGATTTCTGACTTTATGGAGCAATGTATTGTAACTCTACTAAGAAGTGTAAC

ATTTTATTTCTCCCTCTCTAAGGATTGTATAAGAACCTCTTATTTTCAGACTCTACTTAAT

CCTATTTTCTATGTCTGTATGATTTTTATATTTCTAGGACAATCAAATTGGCTTGTAGAAG

CTCAAAAGCATGCTCACAAAGTAGGTACTTATGTAGGGAACTTCGTACTCTAATAATAACT

GGTTATTAACGTTATAATTAAATGCAAAATTTGATAAGTAGTAGGGTTTGGTAAGTAATAT

AACAACACCATGTTGGCTTGGGATTCTGAATTGTGTTATTGGAGTACAACTCTTAAATAAT

GATCGTGATGTAAATAATTTAAATTTGAAGTTACTAGATAAAAAGATAACATTATGTAATA

TATTATCAAATATATACTAAAAATATAATTTTCATCTATCTCAATTGAAAAAATATCCGGG

TTTTGAGTCTATTGTCAATGACGA

>HM061

AAAAAAATAAAGGTTCTATACTCATTAAACAAAGTGCCAAATTCAGAGATACTACTTCCT

TTGGATAATTTGTATCGTCACCAACGATCTTTGAGTTATTTCAAAAATCACATTCTCAATT

TTGTGGTCCAACATTCACAAAATGGTTTGTCGGGCTTTAAGTCTAGTAATTGTGAAACATG

CATATGCATGAGGTAATTAATTGATAATCTGAAAAGGTTAAATAGTTAATCAATTGTACTT

TGGTTATTTTTAATTTGTTCTCTATAATTTTTCAGTCAAAATTGAATTTGCTTTAACCATT

TAGTGTAGTAGTATGATATTACTTGCTCTACATTCTTGGATCTAAAAGTCTAATTAGTATC

GTATAAAAAATATTCTAGTTAATTAAGATACTTTTGTTCATTCTAATTATTCTTGGATTAC

TTGTGAAACTTTTAGGGATGAACAAGAAAGTCCACTAAAGAGGCTTCTTTTTGCTAGGAGT

CACTAGTTAGTTTTTTTTTTTTTTGACAAAAGTTAACCATTTTTTTTTATCGCATGGTAGG

AAAAAAAAAATAGAGATTTCCTTTGGTACTCATAAAAAAAAAAGTTGGATGGATAATGGAT

TAATCATAACTTATTTAACATAAAATGAATTAAGAGAACATTTGATATTTGGGTTTATTTT

GTAACATAAATATTAATATCAATGTTTGAAAGAGTTTTTAACAATAATTTGTAAAAGAAAG

CATAATATATCTATCAAAATAATTGTAAAAGAAAAAATTTCTTTTGGTGGTATGACATGGA

AAAATAATTAATTCTTATTAGATGAAAGTGTAAAATTAATTTGCATTAACAAAGCATATTT

CATTAAACTCCTTTTAGTTTTTGTTGGAAAAAATAAATTTATTGTTACTCAAAAAATAAAA

GTGTACAATTAATATATTTACTCAAAAGTCCCCTAAATGGGATGTAATTATCAAGGGATGT

GAGCGGTTTAACTTTAAGTAATGAATCAACTGTTGCATAATCACTTTGAAAGAAACTTGAG

ATTCTTCAATTAGCAAAATCTTGAATAGAATAATGAGATTATTCCTAATGCTAATGATACC

CACCTCTCCTATAAGTTGATGGGGCAGAACAAATTATGCACTCAGGACAACTTTTCTCAAA

TTTTGAAAACTTACCACACGAAGAACACACTTCCACTTGCACAAGTAAGAATATAGTATAA

CAATAAATTTACCACTCATAGCTCAATGGATATTAAAATATTGCTAATCTTTTATAGACAG

ATGTTGGCAAATTAGTATAAATTAGTTATTATATTAGTTTTTAAGGTTCAATCTACTTTTT

AAAACTCTTATTAAGTTGTTTAGCTTGCCAACCAAAGACTAAATTATCTTTATTTGTTAGG

AAATAAAAGCACTTATTTTATTTTCTCCATATTTGTTTCCTCTTTGTTCCATTACTAAAAA

AATTATAATATATTAGTAACAAAAAAATTTGGGGAGGGAATGTTATTTTTAAAAAAGATGT

TTTATTTATATTTAAGTGTTTTATTGAAATTCCTCGTGAGTTTAGCTCAATTGGTATGGAC

AATACATAATATATGCAAAATTCGAGGTTCAAACCCTGAGCACCAAAAAAAAAAGTGTTTT

ATTGAAAAATATCAAGAACTTGAAAACAAATTTCCTTTTTATGAACTAGATATTGTCACAT

AGACAACTTAAGAATTGACATTTTTTTTAACACTATTGTATTTTCTGAACTCGACTTTAAC

TCGAGATCAATGAGTAAACTAAAAAAACTCGTATTATTTTATCTAAGTGTTCTTAGTGTGA

GAAAACATTATAATTGTTTGCTTAGTCTATTTCTCCCTATATTCTCACCCTCATTCTTGGT

TTGCACATAGAAAGGAGAAAAAAAAAATTAGAGAAGAGTTGAATGGAACAGAAAGAAAGAG

CAAGCAAAATAATCACTAAAAAAAATTCCCCTAAAAGAAAATCACTGAAAAATATTATCCA

CCTCTATTTTAAATAATAATAATAATGAAAACAACAATATATTTTTTTATAGATTTTCTCT

ACTAGAGGTAATTTATTTTAAACACTAAATGTCCACACCTCTAACAAAAAAAATGCATAAT

ATTATAATAGATTATTGTAAGAAAGAGTGACAAATGTTGAATTAGTTATGGAATGAAGAGT

GAGAGAGATTATACAAATAAATAAATATAAATATAAATATAAATAAAGAATAGAGAAGAAC

ATAGTACTTGAATGAAGAGATTTGGCTTGTACTTCCATTACACTTGTAAGCTTTCTACTTC

CTTCACATTCCTTCTCTTTCTCTATCTTTCCTTTTATGATATGTGTACTTTGTTATTGCTT

CATTTACTACCGACATGACTCCAACCTATTAGGGCTTTATCTGAATAATGTTAGTTATTTT

CTCTCATTTTCTTTGCTTCTTCTTATTCTTGGAACTTATAAAGGGTCTTGTCAATTCTATC

ATTCTCTTTTGTATACTTACAAATATAAATATTCTCTTTAGCTTCATTTTCATGTCTTCTT

CTACTAATAAGCTATGGAAATTGAAGTTTTTGTTTTCATGTTTATTTTTCACTTCAGCTCC

TCTTTTTTGATGTTGATGCTGAAGATTGAATAAAAACTGAGGTGGTATGTATGAGAACAAG

AACATTTTTCATTCATCTCTGTTATTTGAACATCTAAATTTTAAACAACTATCGCGACAAT

TTTTTAAGTGGTTGAAGTATTTTAAATAGGTGGTTAATTACATTAAGTAGATAGTTAATTG

ATATATCAGGTGTCAAAACTCATTAACCATCAACATAACTTCATTAGCCATGAATTTTTGA

CGTGATTAACCAAATATTGTAGATGCTCAATTACATGTAGATTTGATGGAGTTGTCCACAA

TTTCTCATATATCTATTAACTAATAACCATCTACCGAATATAATTAACCATATATTAGAAT

ATCATTAACCTCTAAAACAGTTGTCGCGAAAGTTGGTTGTACAAAAATCATTTCTATTTTT

TACGCCACTAGTTCTCTTGTTATTTCTCTTTGTTGGAAAGTTGTTGTTTTTAACTTTGAGC

TTAGTCTTATTAATTAACTTGTAATGGTAACAACATTTTTCTATCTTTGTTTGAAGGAAGT

AGTTACTGTGTCAACTTTAGTAATTAATTTGGATCATTTTGGAGGCAAAATATGGCTATGC

AACCTGTTTATTTTAAAGAACATGAAGGAAATGTCCACAATTCTGTTGGACAGTTTTCATC

TGTGACTTCAGCACCATGGTGGAGTAATGCCTATGGATCTCAACCTGTTTATGGAGGAGAC

TCTTGTGGCCAAATGAAACCTTTTTCACTAGAGCTTTCCAACTACATAGACCAACTTGCTC

CGAGTAAGAACTTAGTTCGAGGAGTTGAACAATTGTTTGATAAAGGGCATACAAACCAATT

CACCATCTTTCCAGGTACTTGTTATTCAATATAATTCCGGTTTTGAATGAATTGATTTTTG

TTAAGTTGGTTCTGGCTAAACGTGCGTTAAATATAATATGATTTATGTTTGGATACGTTTA

TGTAAAAGTGAGTTGAACATAAATTGGAGACTAAATATCAATTGCAGAGGCAAAAGCTTCA

AATTCTAATTTCAAGTTAGAATCAAATCAATTCTACTCGTAAGCATCTAAATATTTCAAAA

CCAATTCTACATCTCTAGAACTAAATTTGCATTCAAACATTTTCTGCTACAGATGATTGTA

AGATGTCAGTTGATACACCAAATCATCAAGCAACCTTATCCCTGCAATCACCATTTGCTGC

CGAGCCACTTAATCGATTTGAGCTAGGTTTTAACCAGTCTATGGTAAATCTTCTTATTAAA

GCAATCCCTTTGATCATGTTTTAGTATTTTACTTTTGATGTACATAAATTCATTATGTTTT

TATTATCCTTTCCATGCTAGATCTGTGCAAAATATCCGTATATAGATCAATTTTACGGGCT

CTTCTCGACTTATGGACCTCAAATCTCGGTTTGTCTTCACCTCGGCTATTTTATTAATCTT

TGTATATATCATGCAATGTCTATAAGTACTTGAATGTGAATTTGTTTTATTTACAGTGTGT

ATGCTTACATGAGAAAGCCAGAAATCTGTTTACAACTATCTTGTTAGTATTCTTCTAAATC

TTTCAAATGTAGAGTTGATTATGAAATTTGTTGTTAATGAGTTTTCGGCTTTATGAATGAA

TTGTAAGACAAGTAATGAAACAAAAACTAGAAGAAAATTGAATTATATGATGCAATCTGTG

TTGAAGTTTTTGCATGTATTCAAGTTCTGCTTTTAGTTACTATTTGTTTATCTGCTGCAAT

ATTTCATCAATTATTATTCATGTATGTACCAATATTTAATAAATTAGGGGCGAATTATGCT

TCCGCTTAGCATGACATCTGACGACGGACCAACATACGTGAATGCTAAGCAATACCATGGA

ATCATCAGACGCAGGCATTCTCGTGCCAAAGCTGTGCTTCAGAATAAATTGATAAAGCGTA

ACAAGGTATGAAACTGAGTATTTTTCCTCACAACAATGTTCGAAAACTTGTGAAATAGTAT

ATTTTTCCTTTTACCCTTTTTATACTTATATTAAGGTTTTGTATTTGTCTTGCAGCCATAT

ATGCACGAATCGCGTCATCTACATGCAATGCGTAGACCAAGAGGATGCGGTGGTCGTTTCT

TGAACACAAAAGTTTCTGCTAATGGAAACGGTAAAAGCGGGAGTGAAGAGAACGGAAACAT

TGGTGGCCTACAGCTGCAGTCCAGTGGTTCTCAGAGTTCCGAAGTCTTACAATCTGAGGTT

GGAACTTTAAATTCGTCGAAGGAGACAAACGGAGGCAGTCCAAATGTCTCGGGGTCAGAGG

TGACTAGCATGTATACACAGGGAGGTCTTGATAGCTTTACTGTCAATCATATCGGATCTAC

TGTCCACTCTTTGGGAGACATGATCGATACTGGACACGGTATCGTCATGCCAACAAAATGG

TTTGCAGCAGCTGGCAGACAGCTGCTGGAACCATAAGTTTCGATTCAGAAAGGAAACAAGT

GGGTTTGGTACAATGTGAAATATTTTGCACCAAACTCATCCTTTCCGAGACCAGATGAAGA

AGCTATGTTTCAGTTTGTTGTGTTTACTACGACAAATTTAGTTTCGGAAGACTACTTTTCC

ATCTGGTGCTCAGGCAACTCATTCTTGGCTTATTCTCAGGAAACTCATCCTTGGCTCGTAA

TATTTAGTAGTATTGTCATTGTCTTTCCGCGCAGGCTTGCCGTGGCATGGTAGGCATGCTA

ATGACTTTGGTATTTTCATGCAGTTATAACTATGATGTGTCTTTGTTTGTTGTTAAAATAA

AAAACATGAACTCTAGCTAGGTGCATGTGTGTGTTTTTAATCTTGTCTACTAAGTTTGGTG

TTTTGTAATGGATTTCTGACTTTATGGAGCAATGTATTGTAACTCTACTAAGAAGTGTAAC

ATTTTATTTCTCCCTCTCTAAGGATTGTATAAGAACCTCTTATTTTCAGACTCTACTTAAT

CCTATTTTCTATGTCTGTATGATTTTTATATTTCTAGGACAATCAAATTGGCTTGTAGAAG

CTCAAAAGCATGCTCACAAAGTAGGTACTTATGTAGGGAACTTCGTACTCTAATAATAACT

GGTTATTAACGTTATAATTAAATGCAAAATTTGATAAGTAGTAGGGTTTGGTAAGTAATAT

AACAACACCATGTTGGCTTGGGATTCTGAATTGTGTTATTGGAGTACAACTCTTAAATAAT

GATCGTGATGTAAATAATTTAAATTTGAAGTTACTAGATAAAAAGATAACATTATGTAATA

TATTATCAAATATATACTAAAAATATAATTTTCATCTATCTCAATTGAAAAAATATCCGGG

TTTTGAGTCTATTGTCAATGACGA

>HM062

AAAAAAATAAAGGTTCTATACTCATTAAACAAAGTGCCAAATTCAGAGATACTACTTCCT

TTGGATAATTTGTATCGTCACCAACGATCTTTGAGTTATTTCAAAAATCACATTCTCAATT

TTGTGGTCCAACATTCACAAAATGGTTTGTCGGGCTTTAAGTCTAGTAATTGTGAAACATG

CATATGCATGAGGTAATTAATTGATAATCTGAAAAGGTTAAATAGTTAATCAATTGTACTT

TGGTTATTTTTAATTTGTTCTCTATAATTTTTCAGTCAAAATTGAATTTGCTTTAACCATT

TAGTGTAGTAGTATGATATTACTTGCTCTACATTCTTGGATCTAAAAGTCTAATTAGTATC

GTATAAAAAATATTCTAGTTAATTAAGATACTTTTGTTCATTCTAATTATTCTTGGATTAC

TTGTGAAACTTTTAGGGATGAACAAGAAAGTCCACTAAAGAGGCTTCTTTTTGCTAGGAGT

CACTAGTTAGTTTTTTTTTTTTTTGACAAAAGTTAACCATTTTTTTTTATCGCATGGTAGG

AAAAAAAAAATAGAGATTTCCTTTGGTACTCATAAAAAAAAAAGTTGGATGGATAATGGAT

TAATCATAACTTATTTAACATAAAATGAATTAAGAGAACATTTGATATTTGGGTTTATTTT

GTAACATAAATATTAATATCAATGTTTGAAAGAGTTTTTAACAATAATTTGTAAAAGAAAG

CATAATATATCTATCAAAATAATTGTAAAAGAAAAAATTTCTTTTGGTGGTATGACATGGA

AAAATAATTAATTCTTATTAGATGAAAGTGTAAAATTAATTTGCATTAACAAAGCATATTT

CATTAAACTCCTTTTAGTTTTTGTTGGAAAAAATAAATTTATTGTTACTCAAAAAATAAAA

GTGTACAATTAATATATTTACTCAAAAGTCCCCTAAATGGGATGTAATTATCAAGGGATGT

GAGCGGTTTAACTTTAAGTAATGAATCAACTGTTGCATAATCACTTTGAAAGAAACTTGAG

ATTCTTCAATTAGCAAAATCTTGAATAGAATAATGAGATTATTCCTAATGCTAATGATACC

CACCTCTCCTATAAGTTGATGGGGCAGAACAAATTATGCACTCAGGACAACTTTTCTCAAA

TTTTGAAAACTTACCACACGAAGAACACACTTCCACTTGCACAAGTAAGAATATAGTATAA

CAATAAATTTACCACTCATAGCTCAATGGATATTAAAATATTGCTAATCTTTTATAGACAG

ATGTTGGCAAATTAGTATAAATTAGTTATTATATTAGTTTTTAAGGTTCAATCTACTTTTT

AAAACTCTTATTAAGTTGTTTAGCTTGCCAACCAAAGACTAAATTATCTTTATTTGTTAGG

AAATAAAAGCACTTATTTTATTTTCTCCATATTTGTTTCCTCTTTGTTCCATTACTAAAAA

AATTATAATATATTAGTAACAAAAAAATTTGGGGAGGGAATGTTATTTTTAAAAAAGATGT

TTTATTTATATTTAAGTGTTTTATTGAAATTCCTCGTGAGTTTAGCTCAATTGGTATGGAC

AATACATAATATATGCAAAATTCGAGGTTCAAACCCTGAGCACCAAAAAAAAAAGTGTTTT

ATTGAAAAATATCAAGAACTTGAAAACAAATTTCCTTTTTATGAACTAGATATTGTCACAT

AGACAACTTAAGAATTGACATTTTTTTTAACACTATTGTATTTTCTGAACTCGACTTTAAC

TCGAGATCAATGAGTAAACTAAAAAAACTCGTATTATTTTATCTAAGTGTTCTTAGTGTGA

GAAAACATTATAATTGTTTGCTTAGTCTATTTCTCCCTATATTCTCACCCTCATTCTTGGT

TTGCACATAGAAAGGAGAAAAAAAAAATTAGAGAAGAGTTGAATGGAACAGAAAGAAAGAG

CAAGCAAAATAATCACTAAAAAAAATTCCCCTAAAAGAAAATCACTGAAAAATATTATCCA

CCTCTATTTTAAATAATAATAATAATGAAAACAACAATATTTTTTTTTATAGATTTTCTCT

ACTAGAGGTAATTTATTTTAAACACTAAATGTCCACACCTCTAACAAAAAAAATGCATAAT

ATTATAATAGATTATTGTAAGAAAGAGTGACAAATGTTGAATTAGTTATGGAATGAAGAGT

GAGAGAGATTATACAAATAAATAAATATAAATATAAATATAAATAAAGAATAGAGAAGAAC

ATAGTACTTGAATGAAGAGATTTGGCTTGTACTTCCATTACACTTGTAAGCTTTCTACTTC

CTTCACATTCCTTCTCTTTCTCTATCTTTCCTTTTATGATATGTGTACTTTGTTATTGCTT

CATTTACTACCGACATGACTCCAACCTATTAGGGCTTTATCTGAATAATGTTAGTTATTTT

CTCTCATTTTCTTTGCTTCTTCTTATTCTTGGAACTTATAAAGGGTCTTGTCAATTCTATC

ATTCTCTTTTGTATACTTACAAATATAAATATTCTCTTTAGCTTCATTTTCATGTCTTCTT

CTACTAATAAGCTATGGAAATTGAAGTTTTTGTTTTCATGTTTATTTTTCACTTCAGCTCC

TCTTTTTTGATGTTGATGCTGAAGATTGAATAAAAACTGAGGTGGTATGTATGAGAACAAG

AACATTTTTCATTCATCTCTGTTATTTGAACATCTAAATTTTAAACAACTATCGCGACAAT

TTTTTAAGTGGTTGAAGTATTTTAAATAGGTGGTTAATTACATTAAGTAGATAGTTAATTG

ATATATCAGGTGTCAAAACTCATTAACCATCAACATAACTTCATTAGCCATGAATTTTTGA

CGTGATTAACCAAATATTGTAGATGCTCAATTACATGTAGATTTGATGGAGTTGTCCACAA

TTTCTCATATATCTATTAACTAATAACCATCTACCGAATATAATTAACCATATATTAGAAT

ATCATTAACCTCTAAAACAGTTGTCGCGAAAGTTGGTTGTACAAAAATCATTTCTATTTTT

TACGCCACTAGTTCTCTTGTTATTTCTCTTTGTTGGAAAGTTGTTGTTTTTAACTTTGAGC

TTAGTCTTATTAATTAACTTGTAATGGTAACAACATTTTTCTATCTTTGTTTGAAGGAAGT

AGTTACTGTGTCAACTTTAGTAATTAATTTGGATCATTTTGGAGGCAAAATATGGCTATGC

AACCTGTTTATTTTAAAGAACATGAAGGAAATGTCCACAATTCTGTTGGACAGTTTTCATC

TGTGACTTCAGCACCATGGTGGAGTAATGCCTATGGATCTCAACCTGTTTATGGAGGAGAC

TCTTGTGGCCAAATGAAACCTTTTTCACTAGAGCTTTCCAACTACATAGACCAACTTGCTC

CGAGTAAGAACTTAGTTCGAGGAGTTGAACAATTGTTTGATAAAGGGCATACAAACCAATT

CACCATCTTTCCAGGTACTTGTTATTCAATATAATTCCGGTTTTGAATGAATTGATTTTTG

TTAAGTTGGTTCTGGCTAAACGTGCGTTAAATATAATATGATTTATGTTTGGATACGTTTA

TGTAAAAGTGAGTTGAACATAAATTGGAGACTAAATATCAATTGCAGAGGCAAAAGCTTCA

AATTCTAATTTCAAGTTAGAATCAAATCAATTCTACTCGTAAGCATCTAAATATTTCAAAA

CCAATTCTACATCTCTAGAACTAAATTTGCATTCAAACATTTTCTGCTACAGATGATTGTA

AGATGTCAGTTGATACACCAAATCATCAAGCAACCTTATCCCTGCAATCACCATTTGCTGC

CGAGCCACTTAATCGATTTGAGCTAGGTTTTAACCAGTCTATGGTAAATCTTCTTATTAAA

GCAATCCCTTTGATCATGTTTTAGTATTTTACTTTTGATGTACATAAATTCATTATGTTTT

TATTATCCTTTCCATGCTAGATCTGTGCAAAATATCCGTATATAGATCAATTTTACGGGCT

CTTCTCGACTTATGGACCTCAAATCTCGGTTTGTCTTCACCTCGGCTATTTTATTAATCTT

TGTATATATCATGCAATGTCTATAAGTACTTGAATGTGAATTTGTTTTATTTACAGTGTGT

ATGCTTACATGAGAAAGCCAGAAATCTGTTTACAACTATCTTGTTAGTATTCTTCTAAATC

TTTCAAATGTAGAGTTGATTATGAAATTTGTTGTTAATGAGTTTTCGGCTTTATGAATGAA

TTGTAAGACAAGTAATGAAACAAAAACTAGAAGAAAATTGAATTATATGATGCAATCTGTG

TTGAAGTTTTTGCATGTATTCAAGTTCTGCTTTTAGTTACTATTTGTTTATCTGCTGCAAT

ATTTCATCAATTATTATTCATGTATGTACCAATATTTAATAAATTAGGGGCGAATTATGCT

TCCGCTTAGCATGACATCTGACGACGGACCAACATACGTGAATGCTAAGCAATACCATGGA

ATCATCAGACGCAGGCATTCTCGTGCCAAAGCTGTGCTTCAGAATAAATTGATAAAGCGTA

ACAAGGTATGAAACTGAGTATTTTTCCTCACAACAATGTTCGAAAACTTGTGAAATAGTAT

ATTTTTCCTTTTACCCTTTTTATACTTATATTAAGGTTTTGTATTTGTCTTGCAGCCATAT

ATGCACGAATCGCGTCATCTACATGCAATGCGTAGACCAAGAGGATGCGGTGGTCGTTTCT

TGAACACAAAAGTTTCTGCTAATGGAAACGGTAAAAGCGGGAGTGAAGAGAACGGAAACAT

TGGTGGCCTACAGCTGCAGTCCAGTGGTTCTCAGAGTTCCGAAGTCTTACAATCTGAGGTT

GGAACTTTAAATTCGTCGAAGGAGACAAACGGAGGCAGTCCAAATGTCTCGGGGTCAGAGG

TGACTAGCATGTATACACAGGGAGGTCTTGATAGCTTTACTGTCAATCATATCGGATCTAC

TGTCCACTCTTTGGGAGACATGATCGATACTGGACACGGTATCGTCATGCCAACAAAATGG

TTTGCAGCAGCTGGCAGACAGCTGCTGGAACCATAAGTTTCGATTCAGAAAGGAAACAAGT

GGGTTTGGTACAATGTGAAATATTTTGCACCAAACTCATCCTTTCCGAGACCAGATGAAGA

AGCTATGTTTCAGTTTGTTGTGTTTACTACGACAAATTTAGTTTCGGAAGACTACTTTTCC

ATCTGGTGCTCAGGCAACTCATTCTTGGCTTATTCTCAGGAAACTCATCCTTGGCTCGTAA

TATTTAGTAGTATTGTCATTGTCTTTCCGCGCAGGCTTGCCGTGGCATGGTAGGCATGCTA

ATGACTTTGGTATTTTCATGCAGTTATAACTATGATGTGTCTTTGTTTGTTGTTAAAATAA

AAAACATGAACTCTAGCTAGGTGCATGTGTGTGTTTTTAATCTTGTCTACTAAGTTTGGTG

TTTTGTAATGGATTTCTGACTTTATGGAGCAATGTATTGTAACTCTACTAAGAAGTGTAAC

ATTTTATTTCTCCCTCTCTAAGGATTGTATAAGAACCTCTTATTTTCAGACTCTACTTAAT

CCTATTTTCTATGTCTGTATGATTTTTATATTTCTAGGACAATCAAATTGGCTTGTAGAAG

CTCAAAAGCATGCTCACAAAGTAGGTACTTATGTAGGGAACTTCGTACTCTAATAATAACT

GGTTATTAACGTTATAATTAAATGCAAAATTTGATAAGTAGTAGGGTTTGGTAAGTAATAT

AACAACACCATGTTGGCTTGGGATTCTGAATTGTGTTATTGGAGTACAACTCTTAAATAAT

GATCGTGATGTAAATAATTTAAATTTGAAGTTACTAGATAAAAAGATAACATTATGTAATA

TATTATCAAATATATACTAAAAATATAATTTTCATCTATCTCAATTGAAAAAATATCCGGG

TTTTGAGTCTATTGTCAATGACGA

>HM063

AAAAAAATAAAGGTTCTATACTCATTAAACAAAGTGCCAAATTCAGAGATACTACTTCCT

TTGGATAATTTGTATCGTCACCAACGATCTTTGAGTTATTTCAAAAATCACATTCTCAATT

TTGTGGTCCAACATTCACAAAATGGTTTGTCGGGCTTTAAGTCTAGTAATTGTGAAACATG

CATATGCATGAGGTAATTAATTGATAATCTGAAAAGGTTAAATAGTTAATCAATTGTACTT

TGGTTATTTTTAATTTGTTCTCTATAATTTTTCAGTCAAAATTGAATTTGCTTTAACCATT

TAGTGTAGTAGTATGATATTACTTGCTCTACATTCTTGGATCTAAAAGTCTAATTAGTATC

GTATAAAAAATATTCTAGTTAATTAAGATACTTTTGTTCATTCTAATTATTCTTGGATTAC

TTGTGAAACTTTTAGGGATGAACAAGAAAGTCCACTAAAGAGGCTTCTTTTTGCTAGGAGT

CACTAGTTAGTTTTTTTTTTTTTTGACAAAAGTTAACCATTTTTTTTTATCGCATGGTAGG

AAAAAAAAAATAGAGATTTCCTTTGGTACTCATAAAAAAAAAAGTTGGATGGATAATGGAT

TAATCATAACTTATTTAACATAAAATGAATTAAGAGAACATTTGATATTTGGGTTTATTTT

GTAACATAAATATTAATATCAATGTTTGAAAGAGTTTTTAACAATAATTTGTAAAAGAAAG

CATAATATATCTATCAAAATAATTGTAAAAGAAAAAATTTCTTTTGGTGGTATGACATGGA

AAAATAATTAATTCTTATTAGATGAAAGTGTAAAATTAATTTGCATTAACAAAGCATATTT

CATTAAACTCCTTTTAGTTTTTGTTGGAAAAAATAAATTTATTGTTACTCAAAAAATAAAA

GTGTACAATTAATATATTTACTCAAAAGTCCCCTAAATGGGATGTAATTATCAAGGGATGT

GAGCGGTTTAACTTTAAGTAATGAATCAACTGTTGCATAATCACTTTGAAAGAAACTTGAG

ATTCTTCAATTAGCAAAATCTTGAATAGAATAATGAGATTATTCCTAATGCTAATGATACC

CACCTCTCCTATAAGTTGATGGGGCAGAACAAATTATGCACTCAGGACAACTTTTCTCAAA

TTTTGAAAACTTACCACACGAAGAACACACTTCCACTTGCACAAGTAAGAATATAGTATAA

CAATAAATTTACCACTCATAGCTCAATGGATATTAAAATATTGCTAATCTTTTATAGACAG

ATGTTGGCAAATTAGTATAAATTAGTTATTATATTAGTTTTTAAGGTTCAATCTACTTTTT

AAAACTCTTATTAAGTTGTTTAGCTTGCCAACCAAAGACTAAATTATCTTTATTTGTTAGG

AAATAAAAGCACTTATTTTATTTTCTCCATATTTGTTTCCTCTTTGTTCCATTACTAAAAA

AATTATAATATATTAGTAACAAAAAAATTTGGGGAGGGAATGTTATTTTTAAAAAAGATGT

TTTATTTATATTTAAGTGTTTTATTGAAATTCCTCGTGAGTTTAGCTCAATTGGTATGGAC

AATACATAATATATGCAAAATTCGAGGTTCAAACCCTGAGCACCAAAAAAAAAAGTGTTTT

ATTGAAAAATATCAAGAACTTGAAAACAAATTTCCTTTTTATGAACTAGATATTGTCACAT

AGACAACTTAAGAATTGACATTTTTTTTAACACTATTGTATTTTCTGAACTCGACTTTAAC

TCGAGATCAATGAGTAAACTAAAAAAACTCGTATTATTTTATCTAAGTGTTCTTAGTGTGA

GAAAACATTATAATTGTTTGCTTAGTCTATTTCTCCCTATATTCTCACCCTCATTCTTGGT

TTGCACATAGAAAGGAGAAAAAAAAAATTAGAGAAGAGTTGAATGGAACAGAAAGAAAGAG

CAAGCAAAATAATCACTAAAAAAAATTCCCCTAAAAGAAAATCACTGAAAAATATTATCCA

CCTCTATTTTAAATAATAATAATAATGAAAACAACAATATTTTTTTTTATAGATTTTCTCT

ACTAGAGGTAATTTATTTTAAACACTAAATGTCCACACCTCTAACAAAAAAAATGCATAAT

ATTATAATAGATTATTGTAAGAAAGAGTGACAAATGTTGAATTAGTTATGGAATGAAGAGT

GAGAGAGATTATACAAATAAATAAATATAAATATAAATATAAATAAAGAATAGAGAAGAAC

ATAGTACTTGAATGAAGAGATTTGGCTTGTACTTCCATTACACTTGTAAGCTTTCTACTTC

CTTCACATTCCTTCTCTTTCTCTATCTTTCCTTTTATGATATGTGTACTTTGTTATTGCTT

CATTTACTACCGACATGACTCCAACCTATTAGGGCTTTATCTGAATAATGTTAGTTATTTT

CTCTCATTTTCTTTGCTTCTTCTTATTCTTGGAACTTATAAAGGGTCTTGTCAATTCTATC

ATTCTCTTTTGTATACTTACAAATATAAATATTCTCTTTAGCTTCATTTTCATGTCTTCTT

CTACTAATAAGCTATGGAAATTGAAGTTTTTGTTTTCATGTTTATTTTTCACTTCAGCTCC

TCTTTTTTGATGTTGATGCTGAAGATTGAATAAAAACTGAGGTGGTATGTATGAGAACAAG

AACATTTTTCATTCATCTCTGTTATTTGAACATCTAAATTTTAAACAACTATCGCGACAAT

TTTTTAAGTGGTTGAAGTATTTTAAATAGGTGGTTAATTACATTAAGTAGATAGTTAATTG

ATATATCAGGTGTCAAAACTCATTAACCATCAACATAACTTCATTAGCCATGAATTTTTGA

CGTGATTAACCAAATATTGTAGATGCTCAATTACATGTAGATTTGATGGAGTTGTCCACAA

TTTCTCATATATCTATTAACTAATAACCATCTACCGAATATAATTAACCATATATTAGAAT

ATCATTAACCTCTAAAACAGTTGTCGCGAAAGTTGGTTGTACAAAAATCATTTCTATTTTT

TACGCCACTAGTTCTCTTGTTATTTCTCTTTGTTGGAAAGTTGTTGTTTTTAACTTTGAGC

TTAGTCTTATTAATTAACTTGTAATGGTAACAACATTTTTCTATCTTTGTTTGAAGGAAGT

AGTTACTGTGTCAACTTTAGTAATTAATTTGGATCATTTTGGAGGCAAAATATGGCTATGC

AACCTGTTTATTTTAAAGAACATGAAGGAAATGTCCACAATTCTGTTGGACAGTTTTCATC

TGTGACTTCAGCACCATGGTGGAGTAATGCCTATGGATCTCAACCTGTTTATGGAGGAGAC

TCTTGTGGCCAAATGAAACCTTTTTCACTAGAGCTTTCCAACTACATAGACCAACTTGCTC

CGAGTAAGAACTTAGTTCGAGGAGTTGAACAATTGTTTGATAAAGGGCATACAAACCAATT

CACCATCTTTCCAGGTACTTGTTATTCAATATAATTCCGGTTTTGAATGAATTGATTTTTG

TTAAGTTGGTTCTGGCTAAACGTGCGTTAAATATAATATGATTTATGTTTGGATACGTTTA

TGTAAAAGTGAGTTGAACATAAATTGGAGACTAAATATCAATTGCAGAGGCAAAAGCTTCA

AATTCTAATTTCAAGTTAGAATCAAATCAATTCTACTCGTAAGCATCTAAATATTTCAAAA

CCAATTCTACATCTCTAGAACTAAATTTGCATTCAAACATTTTCTGCTACAGATGATTGTA

AGATGTCAGTTGATACACCAAATCATCAAGCAACCTTATCCCTGCAATCACCATTTGCTGC

CGAGCCACTTAATCGATTTGAGCTAGGTTTTAACCAGTCTATGGTAAATCTTCTTATTAAA

GCAATCCCTTTGATCATGTTTTAGTATTTTACTTTTGATGTACATAAATTCATTATGTTTT

TATTATCCTTTCCATGCTAGATCTGTGCAAAATATCCGTATATAGATCAATTTTACGGGCT

CTTCTCGACTTATGGACCTCAAATCTCGGTTTGTCTTCACCTCGGCTATTTTATTAATCTT

TGTATATATCATGCAATGTCTATAAGTACTTGAATGTGAATTTGTTTTATTTACAGTGTGT

ATGCTTACATGAGAAAGCCAGAAATCTGTTTACAACTATCTTGTTAGTATTCTTCTAAATC

TTTCAAATGTAGAGTTGATTATGAAATTTGTTGTTAATGAGTTTTCGGCTTTATGAATGAA

TTGTAAGACAAGTAATGAAACAAAAACTAGAAGAAAATTGAATTATATGATGCAATCTGTG

TTGAAGTTTTTGCATGTATTCAAGTTCTGCTTTTAGTTACTATTTGTTTATCTGCTGCAAT

ATTTCATCAATTATTATTCATGTATGTACCAATATTTAATAAATTAGGGGCGAATTATGCT

TCCGCTTAGCATGACATCTGACGACGGACCAACATACGTGAATGCTAAGCAATACCATGGA

ATCATCAGACGCAGGCATTCTCGTGCCAAAGCTGTGCTTCAGAATAAATTGATAAAGCGTA

ACAAGGTATGAAACTGAGTATTTTTCCTCACAACAATGTTCGAAAACTTGTGAAATAGTAT

ATTTTTCCTTTTACCCTTTTTATACTTATATTAAGGTTTTGTATTTGTCTTGCAGCCATAT

ATGCACGAATCGCGTCATCTACATGCAATGCGTAGACCAAGAGGATGCGGTGGTCGTTTCT

TGAACACAAAAGTTTCTGCTAATGGAAACGGTAAAAGCGGGAGTGAAGAGAACGGAAACAT

TGGTGGCCTACAGCTGCAGTCCAGTGGTTCTCAGAGTTCCGAAGTCTTACAATCTGAGGTT

GGAACTTTAAATTCGTCGAAGGAGACAAACGGAGGCAGTCCAAATGTCTCGGGGTCAGAGG

TGACTAGCATGTATACACAGGGAGGTCTTGATAGCTTTACTGTCAATCATATCGGATCTAC

TGTCCACTCTTTGGGAGACATGATCGATACTGGACACGGTATCGTCATGCCAACAAAATGG

TTTGCAGCAGCTGGCAGACAGCTGCTGGAACCATAAGTTTCGATTCAGAAAGGAAACAAGT

GGGTTTGGTACAATGTGAAATATTTTGCACCAAACTCATCCTTTCCGAGACCAGATGAAGA

AGCTATGTTTCAGTTTGTTGTGTTTACTACGACAAATTTAGTTTCGGAAGACTACTTTTCC

ATCTGGTGCTCAGGCAACTCATTCTTGGCTTATTCTCAGGAAACTCATCCTTGGCTCGTAA

TATTTAGTAGTATTGTCATTGTCTTTCCGCGCAGGCTTGCCGTGGCATGGTAGGCATGCTA

ATGACTTTGGTATTTTCATGCAGTTATAACTATGATGTGTCTTTGTTTGTTGTTAAAATAA

AAAACATGAACTCTAGCTAGGTGCATGTGTGTGTTTTTAATCTTGTCTACTAAGTTTGGTG

TTTTGTAATGGATTTCTGACTTTATGGAGCAATGTATTGTAACTCTACTAAGAAGTGTAAC

ATTTTATTTCTCCCTCTCTAAGGATTGTATAAGAACCTCTTATTTTCAGACTCTACTTAAT

CCTATTTTCTATGTCTGTATGATTTTTATATTTCTAGGACAATCAAATTGGCTTGTAGAAG

CTCAAAAGCATGCTCACAAAGTAGGTACTTATGTAGGGAACTTCGTACTCTAATAATAACT

GGTTATTAACGTTATAATTAAATGCAAAATTTGATAAGTAGTAGGGTTTGGTAAGTAATAT

AACAACACCATGTTGGCTTGGGATTCTGAATTGTGTTATTGGAGTACAACTCTTAAATAAT

GATCGTGATGTAAATAATTTAAATTTGAAGTTACTAGATAAAAAGATAACATTATGTAATA

TATTATCAAATATATACTAAAAATATAATTTTCATCTATCTCAATTGAAAAAATATCCGGG

TTTTGAGTCTATTGTCAATGACGA

>HM064

AAAAAAATAAAGGTTCTATACTCATTAAACAAAGTGCCAAATTCAGAGATACTACTTCCT

TTGGATAATTTGTATCGTCACCAACGATCTTTGAGTTATTTCAAAAATCACATTCTCAATT

TTGTGGTCCAACATTCACAAAATGGTTTGTCGGGCTTTAAGTCTAGTAATTGTGAAACATG

CATATGCATGAGGTAATTAATTGATAATCTGAAAAGGTTAAATAGTTAATCAATTGTACTT

TGGTTATTTTTAATTTGTTCTCTATAATTTTTCAGTCAAAATTGAATTTGCTTTAACCATT

TAGTGTAGTAGTATGATATTACTTGCTCTACATTCTTGGATCTAAAAGTCTAATTAGTATC

GTATAAAAAATATTCTAGTTAATTAAGATACTTTTGTTCATTCTAATTATTCTTGGATTAC

TTGTGAAACTTTTAGGGATGAACAAGAAAGTCCACTAAAGAGGCTTCTTTTTGCTAGGAGT

CACTAGTTAGTTTTTTTTTTTTTTGACAAAAGTTAACCATTTTTTTTTATCGCATGGTAGG

AAAAAAAAAATAGAGATTTCCTTTGGTACTCATAAAAAAAAAAGTTGGATGGATAATGGAT

TAATCATAACTTATTTAACATAAAATGAATTAAGAGAACATTTGATATTTGGGTTTATTTT

GTAACATAAATATTAATATCAATGTTTGAAAGAGTTTTTAACAATAATTTGTAAAAGAAAG

CATAATATATCTATCAAAATAATTGTAAAAGAAAAAATTTCTTTTGGTGGTATGACATGGA

AAAATAATTAATTCTTATTAGATGAAAGTGTAAAATTAATTTGCATTAACAAAGCATATTT

CATTAAACTCCTTTTAGTTTTTGTTGGAAAAAATAAATTTATTGTTACTCAAAAAATAAAA

GTGTACAATTAATATATTTACTCAAAAGTCCCCTAAATGGGATGTAATTATCAAGGGATGT

GAGCGGTTTAACTTTAAGTAATGAATCAACTGTTGCATAATCACTTTGAAAGAAACTTGAG

ATTCTTCAATTAGCAAAATCTTGAATAGAATAATGAGATTATTCCTAATGCTAATGATACC

CACCTCTCCTATAAGTTGATGGGGCAGAACAAATTATGCACTCAGGACAACTTTTCTCAAA

TTTTGAAAACTTACCACACGAAGAACACACTTCCACTTGCACAAGTAAGAATATAGTATAA

CAATAAATTTACCACTCATAGCTCAATGGATATTAAAATATTGCTAATCTTTTATAGACAG

ATGTTGGCAAATTAGTATAAATTAGTTATTATATTAGTTTTTAAGGTTCAATCTACTTTTT

AAAACTCTTATTAAGTTGTTTAGCTTGCCAACCAAAGACTAAATTATCTTTATTTGTTAGG

AAATAAAAGCACTTATTTTATTTTCTCCATATTTGTTTCCTCTTTGTTCCATTACTAAAAA

AATTATAATATATTAGTAACAAAAAAATTTGGGGAGGGAATGTTATTTTTAAAAAAGATGT

TTTATTTATATTTAAGTGTTTTATTGAAATTCCTCGTGAGTTTAGCTCAATTGGTATGGAC

AATACATAATATATGCAAAATTCGAGGTTCAAACCCTGAGCACCAAAAAAAAAAGTGTTTT

ATTGAAAAATATCAAGAACTTGAAAACAAATTTCCTTTTTATGAACTAGATATTGTCACAT

AGACAACTTAAGAATTGACATTTTTTTTAACACTATTGTATTTTCTGAACTCGACTTTAAC

TCGAGATCAATGAGTAAACTAAAAAAACTCGTATTATTTTATCTAAGTGTTCTTAGTGTGA

GAAAACATTATAATTGTTTGCTTAGTCTATTTCTCCCTATATTCTCACCCTCATTCTTGGT

TTGCACATAGAAAGGAGAAAAAAAAAATTAGAGAAGAGTTGAATGGAACAGAAAGAAAGAG

CAAGCAAAATAATCACTAAAAAAAATTCCCCTAAAAGAAAATCACTGAAAAATATTATCCA

CCTCTATTTTAAATAATAATAATAATGAAAACAACAATATTTTTTTTTATAGATTTTCTCT

ACTAGAGGTAATTTATTTTAAACACTAAATGTCCACACCTCTAACAAAAAAAATGCATAAT

ATTATAATAGATTATTGTAAGAAAGAGTGACAAATGTTGAATTAGTTATGGAATGAAGAGT

GAGAGAGATTATACAAATAAATAAATATAAATATAAATATAAATAAAGAATAGAGAAGAAC

ATAGTACTTGAATGAAGAGATTTGGCTTGTACTTCCATTACACTTGTAAGCTTTCTACTTC

CTTCACATTCCTTCTCTTTCTCTATCTTTCCTTTTATGATATGTGTACTTTGTTATTGCTT

CATTTACTACCGACATGACTCCAACCTATTAGGGCTTTATCTGAATAATGTTAGTTATTTT

CTCTCATTTTCTTTGCTTCTTCTTATTCTTGGAACTTATAAAGGGTCTTGTCAATTCTATC

ATTCTCTTTTGTATACTTACAAATATAAATATTCTCTTTAGCTTCATTTTCATGTCTTCTT

CTACTAATAAGCTATGGAAATTGAAGTTTTTGTTTTCATGTTTATTTTTCACTTCAGCTCC

TCTTTTTTGATGTTGATGCTGAAGATTGAATAAAAACTGAGGTGGTATGTATGAGAACAAG

AACATTTTTCATTCATCTCTGTTATTTGAACATCTAAATTTTAAACAACTATCGCGACAAT

TTTTTAAGTGGTTGAAGTATTTTAAATAGGTGGTTAATTACATTAAGTAGATAGTTAATTG

ATATATCAGGTGTCAAAACTCATTAACCATCAACATAACTTCATTAGCCATGAATTTTTGA

CGTGATTAACCAAATATTGTAGATGCTCAATTACATGTAGATTTGATGGAGTTGTCCACAA

TTTCTCATATATCTATTAACTAATAACCATCTACCGAATATAATTAACCATATATTAGAAT

ATCATTAACCTCTAAAACAGTTGTCGCGAAAGTTGGTTGTACAAAAATCATTTCTATTTTT

TACGCCACTAGTTCTCTTGTTATTTCTCTTTGTTGGAAAGTTGTTGTTTTTAACTTTGAGC

TTAGTCTTATTAATTAACTTGTAATGGTAACAACATTTTTCTATCTTTGTTTGAAGGAAGT

AGTTACTGTGTCAACTTTAGTAATTAATTTGGATCATTTTGGAGGCAAAATATGGCTATGC

AACCTGTTTATTTTAAAGAACATGAAGGAAATGTCCACAATTCTGTTGGACAGTTTTCATC

TGTGACTTCAGCACCATGGTGGAGTAATGCCTATGGATCTCAACCTGTTTATGGAGGAGAC

TCTTGTGGCCAAATGAAACCTTTTTCACTAGAGCTTTCCAACTACATAGACCAACTTGCTC

CGAGTAAGAACTTAGTTCGAGGAGTTGAACAATTGTTTGATAAAGGGCATACAAACCAATT

CACCATCTTTCCAGGTACTTGTTATTCAATATAATTCCGGTTTTGAATGAATTGAATTTTG

TTAAGTTGGTTCTGGCTAAACGTGCGTTAAATATAATATGATTTATGTTTGGATACGTTTA

TGTAAAAGTGAGTTGAACATAAATTGGAGACTAAATATCAATTGCAGAGGCAAAAGCTTCA

AATTCTAATTTCAAGTTAGAATCAAATCAATTCTACTCGTAAGCATCTAAATATTTCAAAA

CCAATTCTACATCTCTAGAACTAAATTTGCATTCAAACATTTTCTGCTACAGATGATTGTA

AGATGTCAGTTGATACACCAAATCATCAAGCAACCTTATCCCTGCAATCACCATTTGCTGC

CGAGCCACTTAATCGATTTGAGCTAGGTTTTAACCAGTCTATGGTAAATCTTCTTATTAAA

GCAATCCCTTTGATCATGTTTTAGTATTTTACTTTTGATGTACATAAATTCATTATGTTTT

TATTATCCTTTCCATGCTAGATCTGTGCAAAATATCCGTATATAGATCAATTTTACGGGCT

CTTCTCGACTTATGGACCTCAAATCTCGGTTTGTCTTCACCTCGGCTATTTTATTAATCTT

TGTATATATCATGCAATGTCTATAAGTACTTGAATGTGAATTTGTTTTATTTACAGTGTGT

ATGCTTACATGAGAAAGCCAGAAATCTGTTTACAACTATCTTGTTAGTATTCTTCTAAATC

TTTCAAATGTAGAGTTGATTATGAAATTTGTTGTTAATGAGTTTTCGGCTTTATGAATGAA

TTGTAAGACAAGTAATGAAACAAAAACTAGAAGAAAATTGAATTATATGATGCAATCTGTG

TTGAAGTTTTTGCATGTATTCAAGTTCTGCTTTTAGTTACTATTTGTTTATCTGCTGCAAT

ATTTCATCAATTATTATTCATGTATGTACCAATATTTAATAAATTAGGGGCGAATTATGCT

TCCGCTTAGCATGACATCTGACGACGGACCAACATACGTGAATGCTAAGCAATACCATGGA

ATCATCAGACGCAGGCATTCTCGTGCCAAAGCTGTGCTTCAGAATAAATTGATAAAGCGTA

ACAAGGTATGAAACTGAGTATTTTTCCTCACAACAATGTTCGAAAACTTGTGAAATAGTAT

ATTTTTCCTTTTACCCTTTTTATACTTATATTAAGGTTTTGTATTTGTCTTGCAGCCATAT

ATGCACGAATCGCGTCATCTACATGCAATGCGTAGACCAAGAGGATGCGGTGGTCGTTTCT

TGAACACAAAAGTTTCTGCTAATGGAAACGGTAAAAGCGGGAGTGAAGAGAACGGAAACAT

TGGTGGCCTACAGCTGCAGTCCAGTGGTTCTCAGAGTTCCGAAGTCTTACAATCTGAGGTT

GGAACTTTAAATTCGTCGAAGGAGACAAACGGAGGCAGTCCAAATGTCTCGGGGTCAGAGG

TGACTAGCATGTATACACAGGGAGGTCTTGATAGCTTTACTGTCAATCATATCGGATCTAC

TGTCCACTCTTTGGGAGACATGATCGATACTGGACACGGTATCGTCATGCCAACAAAATGG

TTTGCAGCAGCTGGCAGACAGCTGCTGGAACCATAAGTTTCGATTCAGAAAGGAAACAAGT

GGGTTTGGTACAATGTGAAATATTTTGCACCAAACTCATCCTTTCCGAGACCAGATGAAGA

AGCTATGTTTCAGTTTGTTGTGTTTACTACGACAAATTTAGTTTCGGAAGACTACTTTTCC

ATCTGGTGCTCAGGCAACTCATTCTTGGCTTATTCTCAGGAAACTCATCCTTGGCTCGTAA

TATTTAGTAGTATTGTCATTGTCTTTCCGCGCAGGCTTGCCGTGGCATGGTAGGCATGCTA

ATGACTTTGGTATTTTCATGCAGTTATAACTATGATGTGTCTTTGTTTGTTGTTAAAATAA

AAAACATGAACTCTAGCTAGGTGCATGTGTGTGTTTTTAATCTTGTCTACTAAGTTTGGTG

TTTTGTAATGGATTTCTGACTTTATGGAGCAATGTATTGTAACTCTACTAAGAAGTGTAAC

ATTTTATTTCTCCCTCTCTAAGGATTGTATAAGAACCTCTTATTTTCAGACTCTACTTAAT

CCTATTTTCTATGTCTGTATGATTTTTATATTTCTAGGACAATCAAATTGGCTTGTAGAAG

CTCAAAAGCATGCTCACAAAGTAGGTACTTATGTAGGGAACTTCGTACTCTAATAATAACT

GGTTATTAACGTTATAATTAAATGCAAAATTTGATAAGTAGTAGGGTTTGGTAAGTAATAT

AACAACACCATGTTGGCTTGGGATTCTGAATTGTGTTATTGGAGTACAACTCTTAAATAAT

GATCGTGATGTAAATAATTTAAATTTGAAGTTACTAGATAAAAAGATAACATTATGTAATA

TATTATCAAATATATACTAAAAATATAATTTTCATCTATCTCAATTGAAAAAATATCCGGG

TTTTGAGTCTATTGTCAATGACGA

>HM065

AAAAAAATAAAGGTTCTATACTCATTAAACAAAGTGCCAAATTCAGAGATACTACTTCCT

TTGGATAATTTGTATCGTCACCAACGATCTTTGAGTTATTTCAAAAATCACATTCTCAATT

TTGTGGTCCAACATTCACAAAATGGTTTGTCGGGCTTTAAGTCTAGTAATTGTGAAACATG

CATATGCATGAGGTAATTAATTGATAATCTGAAAAGGTTAAATAGTTAATCAATTGTACTT

TGGTTATTTTTAATTTGTTCTCTATAATTTTTCAGTCAAAATTGAATTTGCTTTAACCATT

TAGTGTAGTAGTATGATATTACTTGCTCTACATTCTTGGATCTAAAAGTCTAATTAGTATC

GTATAAAAAATATTCTAGTTAATTAAGATACTTTTGTTCATTCTAATTATTCTTGGATTAC

TTGTGAAACTTTTAGGGATGAACAAGAAAGTCCACTAAAGAGGCTTCTTTTTGCTAGGAGT

CACTAGTTAGTTTTTTTTTTTTTTGACAAAAGTTAACCATTTTTTTTTATCGCATGGTAGG

AAAAAAAAAATAGAGATTTCCTTTGGTACTCATAAAAAAAAAAGTTGGATGGATAATGGAT

TAATCATAACTTATTTAACATAAAATGAATTAAGAGAACATTTGATATTTGGGTTTATTTT

GTAACATAAATATTAATATCAATGTTTGAAAGAGTTTTTAACAATAATTTGTAAAAGAAAG

CATAATATATCTATCAAAATAATTGTAAAAGAAAAAATTTCTTTTGGTGGTATGACATGGA

AAAATAATTAATTCTTATTAGATGAAAGTGTAAAATTAATTTGCATTAACAAAGCATATTT

CATTAAACTCCTTTTAGTTTTTGTTGGAAAAAATAAATTTATTGTTACTCAAAAAATAAAA

GTGTACAATTAATATATTTACTCAAAAGTCCCCTAAATGGGATGTAATTATCAAGGGATGT

GAGCGGTTTAACTTTAAGTAATGAATCAACTGTTGCATAATCACTTTGAAAGAAACTTGAG

ATTCTTCAATTAGCAAAATCTTGAATAGAATAATGAGATTATTCCTAATGCTAATGATACC

CACCTCTCCTATAAGTTGATGGGGCAGAACAAATTATGCACTCAGGACAACTTTTCTCAAA

TTTTGAAAACTTACCACACGAAGAACACACTTCCACTTGCACAAGTAAGAATATAGTATAA

CAATAAATTTACCACTCATAGCTCAATGGATATTAAAATATTGCTAATCTTTTATAGACAG

ATGTTGGCAAATTAGTATAAATTAGTTATTATATTAGTTTTTAAGGTTCAATCTACTTTTT

AAAACTCTTATTAAGTTGTTTAGCTTGCCAACCAAAGACTAAATTATCTTTATTTGTTAGG

AAATAAAAGCACTTATTTTATTTTCTCCATATTTGTTTCCTCTTTGTTCCATTACTAAAAA

AATTATAATATATTAGTAACAAAAAAATTTGGGGAGGGAATGTTATTTTTAAAAAAGATGT

TTTATTTATATTTAAGTGTTTTATTGAAATTCCTCGTGAGTTTAGCTCAATTGGTATGGAC

AATACATAATATATGCAAAATTCGAGGTTCAAACCCTGAGCACCAAAAAAAAAAGTGTTTT

ATTGAAAAATATCAAGAACTTGAAAACAAATTTCCTTTTTATGAACTAGATATTGTCACAT

AGACAACTTAAGAATTGACATTTTTTTTAACACTATTGTATTTTCTGAACTCGACTTTAAC

TCGAGATCAATGAGTAAACTAAAAAAACTCGTATTATTTTATCTAAGTGTTCTTAGTGTGA

GAAAACATTATAATTGTTTGCTTAGTCTATTTCTCCCTATATTCTCACCCTCATTCTTGGT

TTGCACATAGAAAGGAGAAAAAAAAAATTAGAGAAGAGTTGAATGGAACAGAAAGAAAGAG

CAAGCAAAATAATCACTAAAAAAAATTCCCCTAAAAGAAAATCACTGAAAAATATTATCCA

CCTCTATTTTAAATAATAATAATAATGAAAACAACAATATTTTTTTTTATAGATTTTCTCT

ACTAGAGGTAATTTATTTTAAACACTAAATGTCCACACCTCTAACAAAAAAAATGCATAAT

ATTATAATAGATTATTGTAAGAAAGAGTGACAAATGTTGAATTAGTTATGGAATGAAGAGT

GAGAGAGATTATACAAATAAATAAATATAAATATAAATATAAATAAAGAATAGAGAAGAAC

ATAGTACTTGAATGAAGAGATTTGGCTTGTACTTCCATTACACTTGTAAGCTTTCTACTTC

CTTCACATTCCTTCTCTTTCTCTATCTTTCCTTTTATGATATGTGTACTTTGTTATTGCTT

CATTTACTACCGACATGACTCCAACCTATTAGGGCTTTATCTGAATAATGTTAGTTATTTT

CTCTCATTTTCTTTGCTTCTTCTTATTCTTGGAACTTATAAAGGGTCTTGTCAATTCTATC

ATTCTCTTTTGTATACTTACAAATATAAATATTCTCTTTAGCTTCATTTTCATGTCTTCTT

CTACTAATAAGCTATGGAAATTGAAGTTTTTGTTTTCATGTTTATTTTTCACTTCAGCTCC

TCTTTTTTGATGTTGATGCTGAAGATTGAATAAAAACTGAGGTGGTATGTATGAGAACAAG

AACATTTTTCATTCATCTCTGTTATTTGAACATCTAAATTTTAAACAACTATCGCGACAAT

TTTTTAAGTGGTTGAAGTATTTTAAATAGGTGGTTAATTACATTAAGTAGATAGTTAATTG

ATATATCAGGTGTCAAAACTCATTAACCATCAACATAACTTCATTAGCCATGAATTTTTGA

CGTGATTAACCAAATATTGTAGATGCTCAATTACATGTAGATTTGATGGAGTTGTCCACAA

TTTCTCATATATCTATTAACTAATAACCATCTACCGAATATAATTAACCATATATTAGAAT

ATCATTAACCTCTAAAACAGTTGTCGCGAAAGTTGGTTGTACAAAAATCATTTCTATTTTT

TACGCCACTAGTTCTCTTGTTATTTCTCTTTGTTGGAAAGTTGTTGTTTTTAACTTTGAGC

TTAGTCTTATTAATTAACTTGTAATGGTAACAACATTTTTCTATCTTTGTTTGAAGGAAGT

AGTTACTGTGTCAACTTTAGTAATTAATTTGGATCATTTTGGAGGCAAAATATGGCTATGC

AACCTGTTTATTTTAAAGAACATGAAGGAAATGTCCACAATTCTGTTGGACAGTTTTCATC

TGTGACTTCAGCACCATGGTGGAGTAATGCCTATGGATCTCAACCTGTTTATGGAGGAGAC

TCTTGTGGCCAAATGAAACCTTTTTCACTAGAGCTTTCCAACTACATAGACCAACTTGCTC

CGAGTAAGAACTTAGTTCGAGGAGTTGAACAATTGTTTGATAAAGGGCATACAAACCAATT

CACCATCTTTCCAGGTACTTGTTATTCAATATAATTCCGGTTTTGAATGAATTGAATTTTG

TTAAGTTGGTTCTGGCTAAACGTGCGTTAAATATAATATGATTTATGTTTGGATACGTTTA

TGTAAAAGTGAGTTGAACATAAATTGGAGACTAAATATCAATTGCAGAGGCAAAAGCTTCA

AATTCTAATTTCAAGTTAGAATCAAATCAATTCTACTCGTAAGCATCTAAATATTTCAAAA

CCAATTCTACATCTCTAGAACTAAATTTGCATTCAAACATTTTCTGCTACAGATGATTGTA

AGATGTCAGTTGATACACCAAATCATCAAGCAACCTTATCCCTGCAATCACCATTTGCTGC

CGAGCCACTTAATCGATTTGAGCTAGGTTTTAACCAGTCTATGGTAAATCTTCTTATTAAA

GCAATCCCTTTGATCATGTTTTAGTATTTTACTTTTGATGTACATAAATTCATTATGTTTT

TATTATCCTTTCCATGCTAGATCTGTGCAAAATATCCGTATATAGATCAATTTTACGGGCT

CTTCTCGACTTATGGACCTCAAATCTCGGTTTGTCTTCACCTCGGCTATTTTATTAATCTT

TGTATATATCATGCAATGTCTATAAGTACTTGAATGTGAATTTGTTTTATTTACAGTGTGT

ATGCTTACATGAGAAAGCCAGAAATCTGTTTACAACTATCTTGTTAGTATTCTTCTAAATC

TTTCAAATGTAGAGTTGATTATGAAATTTGTTGTTAATGAGTTTTCGGCTTTATGAATGAA

TTGTAAGACAAGTAATGAAACAAAAACTAGAAGAAAATTGAATTATATGATGCAATCTGTG

TTGAAGTTTTTGCATGTATTCAAGTTCTGCTTTTAGTTACTATTTGTTTATCTGCTGCAAT

ATTTCATCAATTATTATTCATGTATGTACCAATATTTAATAAATTAGGGGCGAATTATGCT

TCCGCTTAGCATGACATCTGACGACGGACCAACATACGTGAATGCTAAGCAATACCATGGA

ATCATCAGACGCAGGCATTCTCGTGCCAAAGCTGTGCTTCAGAATAAATTGATAAAGCGTA

ACAAGGTATGAAACTGAGTATTTTTCCTCACAACAATGTTCGAAAACTTGTGAAATAGTAT

ATTTTTCCTTTTACCCTTTTTATACTTATATTAAGGTTTTGTATTTGTCTTGCAGCCATAT

ATGCACGAATCGCGTCATCTACATGCAATGCGTAGACCAAGAGGATGCGGTGGTCGTTTCT

TGAACACAAAAGTTTCTGCTAATGGAAACGGTAAAAGCGGGAGTGAAGAGAACGGAAACAT

TGGTGGCCTACAGCTGCAGTCCAGTGGTTCTCAGAGTTCCGAAGTCTTACAATCTGAGGTT

GGAACTTTAAATTCGTCGAAGGAGACAAACGGAGGCAGTCCAAATGTCTCGGGGTCAGAGG

TGACTAGCATGTATACACAGGGAGGTCTTGATAGCTTTACTGTCAATCATATCGGATCTAC

TGTCCACTCTTTGGGAGACATGATCGATACTGGACACGGTATCGTCATGCCAACAAAATGG

TTTGCAGCAGCTGGCAGACAGCTGCTGGAACCATAAGTTTCGATTCAGAAAGGAAACAAGT

GGGTTTGGTACAATGTGAAATATTTTGCACCAAACTCATCCTTTCCGAGACCAGATGAAGA

AGCTATGTTTCAGTTTGTTGTGTTTACTACGACAAATTTAGTTTCGGAAGACTACTTTTCC

ATCTGGTGCTCAGGCAACTCATTCTTGGCTTATTCTCAGGAAACTCATCCTTGGCTCGTAA

TATTTAGTAGTATTGTCATTGTCTTTCCGCGCAGGCTTGCCGTGGCATGGTAGGCATGCTA

ATGACTTTGGTATTTTCATGCAGTTATAACTATGATGTGTCTTTGTTTGTTGTTAAAATAA

AAAACATGAACTCTAGCTAGGTGCATGTGTGTGTTTTTAATCTTGTCTACTAAGTTTGGTG

TTTTGTAATGGATTTCTGACTTTATGGAGCAATGTATTGTAACTCTACTAAGAAGTGTAAC

ATTTTATTTCTCCCTCTCTAAGGATTGTATAAGAACCTCTTATTTTCAGACTCTACTTAAT

CCTATTTTCTATGTCTGTATGATTTTTATATTTCTAGGACAATCAAATTGGCTTGTAGAAG

CTCAAAAGCATGCTCACAAAGTAGGTACTTATGTAGGGAACTTCGTACTCTAATAATAACT

GGTTATTAACGTTATAATTAAATGCAAAATTTGATAAGTAGTAGGGTTTGGTAAGTAATAT

AACAACACCATGTTGGCTTGGGATTCTGAATTGTGTTATTGGAGTACAACTCTTAAATAAT

GATCGTGATGTAAATAATTTAAATTTGAAGTTACTAGATAAAAAGATAACATTATGTAATA

TATTATCAAATATATACTAAAAATATAATTTTCATCTATCTCAATTGAAAAAATATCCGGG

TTTTGAGTCTATTGTCAATGACGA

>HM066

AAAAAAATAAAGGTTCTATACTCATTAAACAAAGTGCCAAATTCAGAGATACTACTTCCT

TTGGATAATTTGTATCGTCACCAACGATCTTTGAGTTATTTCAAAAATCACATTCTCAATT

TTGTGGTCCAACATTCACAAAATGGTTTGTCGGGCTTTAAGTCTAGTAATTGTGAAACATG

CATATGCATGAGGTAATTAATTGATAATCTGAAAAGGTTAAATAGTTAATCAATTGTACTT

TGGTTATTTTTAATTTGTTCTCTATAATTTTTCAGTCAAAATTGAATTTGCTTTAACCATT

TAGTGTAGTAGTATGATATTACTTGCTCTACATTCTTGGATCTAAAAGTCTAATTAGTATC

GTATAAAAAATATTCTAGTTAATTAAGATACTTTTGTTCATTCTAATTATTCTTGGATTAC

TTGTGAAACTTTTAGGGATGAACAAGAAAGTCCACTAAAGAGGCTTCTTTTTGCTAGGAGT

CACTAGTTAGTTTTTTTTTTTTTTGACAAAAGTTAACCATTTTTTTTTATCGCATGGTAGG

AAAAAAAAAATAGAGATTTCCTTTGGTACTCATAAAAAAAAAAGTTGGATGGATAATGGAT

TAATCATAACTTATTTAACATAAAATGAATTAAGAGAACATTTGATATTTGGGTTTATTTT

GTAACATAAATATTAATATCAATGTTTGAAAGAGTTTTTAACAATAATTTGTAAAAGAAAG

CATAATATATCTATCAAAATAATTGTAAAAGAAAAAATTTCTTTTGGTGGTATGACATGGA

AAAATAATTAATTCTTATTAGATGAAAGTGTAAAATTAATTTGCATTAACAAAGCATATTT

CATTAAACTCCTTTTAGTTTTTGTTGGAAAAAATAAATTTATTGTTACTCAAAAAATAAAA

GTGTACAATTAATATATTTACTCAAAAGTCCCCTAAATGGGATGTAATTATCAAGGGATGT

GAGCGGTTTAACTTTAAGTAATGAATCAACTGTTGCATAATCACTTTGAAAGAAACTTGAG

ATTCTTCAATTAGCAAAATCTTGAATAGAATAATGAGATTATTCCTAATGCTAATGATACC

CACCTCTCCTATAAGTTGATGGGGCAGAACAAATTATGCACTCAGGACAACTTTTCTCAAA

TTTTGAAAACTTACCACACGAAGAACACACTTCCACTTGCACAAGTAAGAATATAGTATAA

CAATAAATTTACCACTCATAGCTCAATGGATATTAAAATATTGCTAATCTTTTATAGACAG

ATGTTGGCAAATTAGTATAAATTAGTTATTATATTAGTTTTTAAGGTTCAATCTACTTTTT

AAAACTCTTATTAAGTTGTTTAGCTTGCCAACCAAAGACTAAATTATCTTTATTTGTTAGG

AAATAAAAGCACTTATTTTATTTTCTCCATATTTGTTTCCTCTTTGTTCCATTACTAAAAA

AATTATAATATATTAGTAACAAAAAAATTTGGGGAGGGAATGTTATTTTTAAAAAAGATGT

TTTATTTATATTTAAGTGTTTTATTGAAATTCCTCGTGAGTTTAGCTCAATTGGTATGGAC

AATACATAATATATGCAAAATTCGAGGTTCAAACCCTGAGCACCAAAAAAAAAAGTGTTTT

ATTGAAAAATATCAAGAACTTGAAAACAAATTTCCTTTTTATGAACTAGATATTGTCACAT

AGACAACTTAAGAATTGACATTTTTTTTAACACTATTGTATTTTCTGAACTCGACTTTAAC

TCGAGATCAATGAGTAAACTAAAAAAACTCGTATTATTTTATCTAAGTGTTCTTAGTGTGA

GAAAACATTATAATTGTTTGCTTAGTCTATTTCTCCCTATATTCTCACCCTCATTCTTGGT

TTGCACATAGAAAGGAGAAAAAAAAAATTAGAGAAGAGTTGAATGGAACAGAAAGAAAGAG

CAAGCAAAATAATCACTAAAAAAAATTCCCCTAAAAGAAAATCACTGAAAAATATTATCCA

CCTCTATTTTAAATAATAATAATAATGAAAACAACAATATTTTTTTTTATAGATTTTCTCT

ACTAGAGGTAATTTATTTTAAACACTAAATGTCCACACCTCTAACAAAAAAAATGCATAAT

ATTATAATAGATTATTGTAAGAAAGAGTGACAAATGTTGAATTAGTTATGGAATGAAGAGT

GAGAGAGATTATACAAATAAATAAATATAAATATAAATATAAATAAAGAATAGAGAAGAAC

ATAGTACTTGAATGAAGAGATTTGGCTTGTACTTCCATTACACTTGTAAGCTTTCTACTTC

CTTCACATTCCTTCTCTTTCTCTATCTTTCCTTTTATGATATGTGTACTTTGTTATTGCTT

CATTTACTACCGACATGACTCCAACCTATTAGGGCTTTATCTGAATAATGTTAGTTATTTT

CTCTCATTTTCTTTGCTTCTTCTTATTCTTGGAACTTATAAAGGGTCTTGTCAATTCTATC

ATTCTCTTTTGTATACTTACAAATATAAATATTCTCTTTAGCTTCATTTTCATGTCTTCTT

CTACTAATAAGCTATGGAAATTGAAGTTTTTGTTTTCATGTTTATTTTTCACTTCAGCTCC

TCTTTTTTGATGTTGATGCTGAAGATTGAATAAAAACTGAGGTGGTATGTATGAGAACAAG

AACATTTTTCATTCATCTCTGTTATTTGAACATCTAAATTTTAAACAACTATCGCGACAAT

TTTTTAAGTGGTTGAAGTATTTTAAATAGGTGGTTAATTACATTAAGTAGATAGTTAATTG

ATATATCAGGTGTCAAAACTCATTAACCATCAACATAACTTCATTAGCCATGAATTTTTGA

CGTGATTAACCAAATATTGTAGATGCTCAATTACATGTAGATTTGATGGAGTTGTCCACAA

TTTCTCATATATCTATTAACTAATAACCATCTACCGAATATAATTAACCATATATTAGAAT

ATCATTAACCTCTAAAACAGTTGTCGCGAAAGTTGGTTGTACAAAAATCATTTCTATTTTT

TACGCCACTAGTTCTCTTGTTATTTCTCTTTGTTGGAAAGTTGTTGTTTTTAACTTTGAGC

TTAGTCTTATTAATTAACTTGTAATGGTAACAACATTTTTCTATCTTTGTTTGAAGGAAGT

AGTTACTGTGTCAACTTTAGTAATTAATTTGGATCATTTTGGAGGCAAAATATGGCTATGC

AACCTGTTTATTTTAAAGAACATGAAGGAAATGTCCACAATTCTGTTGGACAGTTTTCATC

TGTGACTTCAGCACCATGGTGGAGTAATGCCTATGGATCTCAACCTGTTTATGGAGGAGAC

TCTTGTGGCCAAATGAAACCTTTTTCACTAGAGCTTTCCAACTACATAGACCAACTTGCTC

CGAGTAAGAACTTAGTTCGAGGAGTTGAACAATTGTTTGATAAAGGGCATACAAACCAATT

CACCATCTTTCCAGGTACTTGTTATTCAATATAATTCCGGTTTTGAATGAATTGAATTTTG

TTAAGTTGGTTCTGGCTAAACGTGCGTTAAATATAATATGATTTATGTTTGGATACGTTTA

TGTAAAAGTGAGTTGAACATAAATTGGAGACTAAATATCAATTGCAGAGGCAAAAGCTTCA

AATTCTAATTTCAAGTTAGAATCAAATCAATTCTACTCGTAAGCATCTAAATATTTCAAAA

CCAATTCTACATCTCTAGAACTAAATTTGCATTCAAACATTTTCTGCTACAGATGATTGTA

AGATGTCAGTTGATACACCAAATCATCAAGCAACCTTATCCCTGCAATCACCATTTGCTGC

CGAGCCACTTAATCGATTTGAGCTAGGTTTTAACCAGTCTATGGTAAATCTTCTTATTAAA

GCAATCCCTTTGATCATGTTTTAGTATTTTACTTTTGATGTACATAAATTCATTATGTTTT

TATTATCCTTTCCATGCTAGATCTGTGCAAAATATCCGTATATAGATCAATTTTACGGGCT

CTTCTCGACTTATGGACCTCAAATCTCGGTTTGTCTTCACCTCGGCTATTTTATTAATCTT

TGTATATATCATGCAATGTCTATAAGTACTTGAATGTGAATTTGTTTTATTTACAGTGTGT

ATGCTTACATGAGAAAGCCAGAAATCTGTTTACAACTATCTTGTTAGTATTCTTCTAAATC

TTTCAAATGTAGAGTTGATTATGAAATTTGTTGTTAATGAGTTTTCGGCTTTATGAATGAA

TTGTAAGACAAGTAATGAAACAAAAACTAGAAGAAAATTGAATTATATGATGCAATCTGTG

TTGAAGTTTTTGCATGTATTCAAGTTCTGCTTTTAGTTACTATTTGTTTATCTGCTGCAAT

ATTTCATCAATTATTATTCATGTATGTACCAATATTTAATAAATTAGGGGCGAATTATGCT

TCCGCTTAGCATGACATCTGACGACGGACCAACATACGTGAATGCTAAGCAATACCATGGA

ATCATCAGACGCAGGCATTCTCGTGCCAAAGCTGTGCTTCAGAATAAATTGATAAAGCGTA

ACAAGGTATGAAACTGAGTATTTTTCCTCACAACAATGTTCGAAAACTTGTGAAATAGTAT

ATTTTTCCTTTTACCCTTTTTATACTTATATTAAGGTTTTGTATTTGTCTTGCAGCCATAT

ATGCACGAATCGCGTCATCTACATGCAATGCGTAGACCAAGAGGATGCGGTGGTCGTTTCT

TGAACACAAAAGTTTCTGCTAATGGAAACGGTAAAAGCGGGAGTGAAGAGAACGGAAACAT

TGGTGGCCTACAGCTGCAGTCCAGTGGTTCTCAGAGTTCCGAAGTCTTACAATCTGAGGTT

GGAACTTTAAATTCGTCGAAGGAGACAAACGGAGGCAGTCCAAATGTCTCGGGGTCAGAGG

TGACTAGCATGTATACACAGGGAGGTCTTGATAGCTTTACTGTCAATCATATCGGATCTAC

TGTCCACTCTTTGGGAGACATGATCGATACTGGACACGGTATCGTCATGCCAACAAAATGG

TTTGCAGCAGCTGGCAGACAGCTGCTGGAACCATAAGTTTCGATTCAGAAAGGAAACAAGT

GGGTTTGGTACAATGTGAAATATTTTGCACCAAACTCATCCTTTCCGAGACCAGATGAAGA

AGCTATGTTTCAGTTTGTTGTGTTTACTACGACAAATTTAGTTTCGGAAGACTACTTTTCC

ATCTGGTGCTCAGGCAACTCATTCTTGGCTTATTCTCAGGAAACTCATCCTTGGCTCGTAA

TATTTAGTAGTATTGTCATTGTCTTTCCGCGCAGGCTTGCCGTGGCATGGTAGGCATGCTA

ATGACTTTGGTATTTTCATGCAGTTATAACTATGATGTGTCTTTGTTTGTTGTTAAAATAA

AAAACATGAACTCTAGCTAGGTGCATGTGTGTGTTTTTAATCTTGTCTACTAAGTTTGGTG

TTTTGTAATGGATTTCTGACTTTATGGAGCAATGTATTGTAACTCTACTAAGAAGTGTAAC

ATTTTATTTCTCCCTCTCTAAGGATTGTATAAGAACCTCTTATTTTCAGACTCTACTTAAT

CCTATTTTCTATGTCTGTATGATTTTTATATTTCTAGGACAATCAAATTGGCTTGTAGAAG

CTCAAAAGCATGCTCACAAAGTAGGTACTTATGTAGGGAACTTCGTACTCTAATAATAACT

GGTTATTAACGTTATAATTAAATGCAAAATTTGATAAGTAGTAGGGTTTGGTAAGTAATAT

AACAACACCATGTTGGCTTGGGATTCTGAATTGTGTTATTGGAGTACAACTCTTAAATAAT

GATCGTGATGTAAATAATTTAAATTTGAAGTTACTAGATAAAAAGATAACATTATGTAATA

TATTATCAAATATATACTAAAAATATAATTTTCATCTATCTCAATTGAAAAAATATCCGGG

TTTTGAGTCTATTGTCAATGACGA

>HM067

AAAAAAATAAAGGTTCTATACTCATTAAACAAAGTGCCAAATTCAGAGATACTACTTCCT

TTGGATAATTTGTATCGTCACCAACGATCTTTGAGTTATTTCAAAAATCACATTCTCAATT

TTGTGGTCCAACATTCACAAAATGGTTTGTCGGGCTTTAAGTCTAGTAATTGTGAAACATG

CATATGCATGAGGTAATTAATTGATAATCTGAAAAGGTTAAATAGTTAATCAATTGTACTT

TGGTTATTTTTAATTTGTTCTCTATAATTTTTCAGTCAAAATTGAATTTGCTTTAACCATT

TAGTGTAGTAGTATGATATTACTTGCTCTACATTCTTGGATCTAAAAGTCTAATTAGTATC

GTATAAAAAATATTCTAGTTAATTAAGATACTTTTGTTCATTCTAATTATTCTTGGATTAC

TTGTGAAACTTTTAGGGATGAACAAGAAAGTCCACTAAAGAGGCTTCTTTTTGCTAGGAGT

CACTAGTTAGTTTTTTTTTTTTTTGACAAAAGTTAACCATTTTTTTTTATCGCATGGTAGG

AAAAAAAAAATAGAGATTTCCTTTGGTACTCATAAAAAAAAAAGTTGGATGGATAATGGAT

TAATCATAACTTATTTAACATAAAATGAATTAAGAGAACATTTGATATTTGGGTTTATTTT

GTAACATAAATATTAATATCAATGTTTGAAAGAGTTTTTAACAATAATTTGTAAAAGAAAG

CATAATATATCTATCAAAATAATTGTAAAAGAAAAAATTTCTTTTGGTGGTATGACATGGA

AAAATAATTAATTCTTATTAGATGAAAGTGTAAAATTAATTTGCATTAACAAAGCATATTT

CATTAAACTCCTTTTAGTTTTTGTTGGAAAAAATAAATTTATTGTTACTCAAAAAATAAAA

GTGTACAATTAATATATTTACTCAAAAGTCCCCTAAATGGGATGTAATTATCAAGGGATGT

GAGCGGTTTAACTTTAAGTAATGAATCAACTGTTGCATAATCACTTTGAAAGAAACTTGAG

ATTCTTCAATTAGCAAAATCTTGAATAGAATAATGAGATTATTCCTAATGCTAATGATACC

CACCTCTCCTATAAGTTGATGGGGCAGAACAAATTATGCACTCAGGACAACTTTTCTCAAA

TTTTGAAAACTTACCACACGAAGAACACACTTCCACTTGCACAAGTAAGAATATAGTATAA

CAATAAATTTACCACTCATAGCTCAATGGATATTAAAATATTGCTAATCTTTTATAGACAG

ATGTTGGCAAATTAGTATAAATTAGTTATTATATTAGTTTTTAAGGTTCAATCTACTTTTT

AAAACTCTTATTAAGTTGTTTAGCTTGCCAACCAAAGACTAAATTATCTTTATTTGTTAGG

AAATAAAAGCACTTATTTTATTTTCTCCATATTTGTTTCCTCTTTGTTCCATTACTAAAAA

AATTATAATATATTAGTAACAAAAAAATTTGGGGAGGGAATGTTATTTTTAAAAAAGATGT

TTTATTTATATTTAAGTGTTTTATTGAAATTCCTCGTGAGTTTAGCTCAATTGGTATGGAC

AATACATAATATATGCAAAATTCGAGGTTCAAACCCTGAGCACCAAAAAAAAAAGTGTTTT

ATTGAAAAATATCAAGAACTTGAAAACAAATTTCCTTTTTATGAACTAGATATTGTCACAT

AGACAACTTAAGAATTGACATTTTTTTTAACACTATTGTATTTTCTGAACTCGACTTTAAC

TCGAGATCAATGAGTAAACTAAAAAAACTCGTATTATTTTATCTAAGTGTTCTTAGTGTGA

GAAAACATTATAATTGTTTGCTTAGTCTATTTCTCCCTATATTCTCACCCTCATTCTTGGT

TTGCACATAGAAAGGAGAAAAAAAAAATTAGAGAAGAGTTGAATGGAACAGAAAGAAAGAG

CAAGCAAAATAATCACTAAAAAAAATTCCCCTAAAAGAAAATCACTGAAAAATATTATCCA

CCTCTATTTTAAATAATAATAATAATGAAAACAACAATATTTTTTTTTATAGATTTTCTCT

ACTAGAGGTAATTTATTTTAAACACTAAATGTCCACACCTCTAACAAAAAAAATGCATAAT

ATTATAATAGATTATTGTAAGAAAGAGTGACAAATGTTGAATTAGTTATGGAATGAAGAGT

GAGAGAGATTATACAAATAAATAAATATAAATATAAATATAAATAAAGAATAGAGAAGAAC

ATAGTACTTGAATGAAGAGATTTGGCTTGTACTTCCATTACACTTGTAAGCTTTCTACTTC

CTTCACATTCCTTCTCTTTCTCTATCTTTCCTTTTATGATATGTGTACTTTGTTATTGCTT

CATTTACTACCGACATGACTCCAACCTATTAGGGCTTTATCTGAATAATGTTAGTTATTTT

CTCTCATTTTCTTTGCTTCTTCTTATTCTTGGAACTTATAAAGGGTCTTGTCAATTCTATC

ATTCTCTTTTGTATACTTACAAATATAAATATTCTCTTTAGCTTCATTTTCATGTCTTCTT

CTACTAATAAGCTATGGAAATTGAAGTTTTTGTTTTCATGTTTATTTTTCACTTCAGCTCC

TCTTTTTTGATGTTGATGCTGAAGATTGAATAAAAACTGAGGTGGTATGTATGAGAACAAG

AACATTTTTCATTCATCTCTGTTATTTGAACATCTAAATTTTAAACAACTATCGCGACAAT

TTTTTAAGTGGTTGAAGTATTTTAAATAGGTGGTTAATTACATTAAGTAGATAGTTAATTG

ATATATCAGGTGTCAAAACTCATTAACCATCAACATAACTTCATTAGCCATGAATTTTTGA

CGTGATTAACCAAATATTGTAGATGCTCAATTACATGTAGATTTGATGGAGTTGTCCACAA

TTTCTCATATATCTATTAACTAATAACCATCTACCGAATATAATTAACCATATATTAGAAT

ATCATTAACCTCTAAAACAGTTGTCGCGAAAGTTGGTTGTACAAAAATCATTTCTATTTTT

TACGCCACTAGTTCTCTTGTTATTTCTCTTTGTTGGAAAGTTGTTGTTTTTAACTTTGAGC

TTAGTCTTATTAATTAACTTGTAATGGTAACAACATTTTTCTATCTTTGTTTGAAGGAAGT

AGTTACTGTGTCAACTTTAGTAATTAATTTGGATCATTTTGGAGGCAAAATATGGCTATGC

AACCTGTTTATTTTAAAGAACATGAAGGAAATGTCCACAATTCTGTTGGACAGTTTTCATC

TGTGACTTCAGCACCATGGTGGAGTAATGCCTATGGATCTCAACCTGTTTATGGAGGAGAC

TCTTGTGGCCAAATGAAACCTTTTTCACTAGAGCTTTCCAACTACATAGACCAACTTGCTC

CGAGTAAGAACTTAGTTCGAGGAGTTGAACAATTGTTTGATAAAGGGCATACAAACCAATT

CACCATCTTTCCAGGTACTTGTTATTCAATATAATTCCGGTTTTGAATGAATTGAATTTTG

TTAAGTTGGTTCTGGCTAAACGTGCGTTAAATATAATATGATTTATGTTTGGATACGTTTA

TGTAAAAGTGAGTTGAACATAAATTGGAGACTAAATATCAATTGCAGAGGCAAAAGCTTCA

AATTCTAATTTCAAGTTAGAATCAAATCAATTCTACTCGTAAGCATCTAAATATTTCAAAA

CCAATTCTACATCTCTAGAACTAAATTTGCATTCAAACATTTTCTGCTACAGATGATTGTA

AGATGTCAGTTGATACACCAAATCATCAAGCAACCTTATCCCTGCAATCACCATTTGCTGC

CGAGCCACTTAATCGATTTGAGCTAGGTTTTAACCAGTCTATGGTAAATCTTCTTATTAAA

GCAATCCCTTTGATCATGTTTTAGTATTTTACTTTTGATGTACATAAATTCATTATGTTTT

TATTATCCTTTCCATGCTAGATCTGTGCAAAATATCCGTATATAGATCAATTTTACGGGCT

CTTCTCGACTTATGGACCTCAAATCTCGGTTTGTCTTCACCTCGGCTATTTTATTAATCTT

TGTATATATCATGCAATGTCTATAAGTACTTGAATGTGAATTTGTTTTATTTACAGTGTGT

ATGCTTACATGAGAAAGCCAGAAATCTGTTTACAACTATCTTGTTAGTATTCTTCTAAATC

TTTCAAATGTAGAGTTGATTATGAAATTTGTTGTTAATGAGTTTTCGGCTTTATGAATGAA

TTGTAAGACAAGTAATGAAACAAAAACTAGAAGAAAATTGAATTATATGATGCAATCTGTG

TTGAAGTTTTTGCATGTATTCAAGTTCTGCTTTTAGTTACTATTTGTTTATCTGCTGCAAT

ATTTCATCAATTATTATTCATGTATGTACCAATATTTAATAAATTAGGGGCGAATTATGCT

TCCGCTTAGCATGACATCTGACGACGGACCAACATACGTGAATGCTAAGCAATACCATGGA

ATCATCAGACGCAGGCATTCTCGTGCCAAAGCTGTGCTTCAGAATAAATTGATAAAGCGTA

ACAAGGTATGAAACTGAGTATTTTTCCTCACAACAATGTTCGAAAACTTGTGAAATAGTAT

ATTTTTCCTTTTACCCTTTTTATACTTATATTAAGGTTTTGTATTTGTCTTGCAGCCATAT

ATGCACGAATCGCGTCATCTACATGCAATGCGTAGACCAAGAGGATGCGGTGGTCGTTTCT

TGAACACAAAAGTTTCTGCTAATGGAAACGGTAAAAGCGGGAGTGAAGAGAACGGAAACAT

TGGTGGCCTACAGCTGCAGTCCAGTGGTTCTCAGAGTTCCGAAGTCTTACAATCTGAGGTT

GGAACTTTAAATTCGTCGAAGGAGACAAACGGAGGCAGTCCAAATGTCTCGGGGTCAGAGG

TGACTAGCATGTATACACAGGGAGGTCTTGATAGCTTTACTGTCAATCATATCGGATCTAC

TGTCCACTCTTTGGGAGACATGATCGATACTGGACACGGTATCGTCATGCCAACAAAATGG

TTTGCAGCAGCTGGCAGACAGCTGCTGGAACCATAAGTTTCGATTCAGAAAGGAAACAAGT

GGGTTTGGTACAATGTGAAATATTTTGCACCAAACTCATCCTTTCCGAGACCAGATGAAGA

AGCTATGTTTCAGTTTGTTGTGTTTACTACGACAAATTTAGTTTCGGAAGACTACTTTTCC

ATCTGGTGCTCAGGCAACTCATTCTTGGCTTATTCTCAGGAAACTCATCCTTGGCTCGTAA

TATTTAGTAGTATTGTCATTGTCTTTCCGCGCAGGCTTGCCGTGGCATGGTAGGCATGCTA

ATGACTTTGGTATTTTCATGCAGTTATAACTATGATGTGTCTTTGTTTGTTGTTAAAATAA

AAAACATGAACTCTAGCTAGGTGCATGTGTGTGTTTTTAATCTTGTCTACTAAGTTTGGTG

TTTTGTAATGGATTTCTGACTTTATGGAGCAATGTATTGTAACTCTACTAAGAAGTGTAAC

ATTTTATTTCTCCCTCTCTAAGGATTGTATAAGAACCTCTTATTTTCAGACTCTACTTAAT

CCTATTTTCTATGTCTGTATGATTTTTATATTTCTAGGACAATCAAATTGGCTTGTAGAAG

CTCAAAAGCATGCTCACAAAGTAGGTACTTATGTAGGGAACTTCGTACTCTAATAATAACT

GGTTATTAACGTTATAATTAAATGCAAAATTTGATAAGTAGTAGGGTTTGGTAAGTAATAT

AACAACACCATGTTGGCTTGGGATTCTGAATTGTGTTATTGGAGTACAACTCTTAAATAAT

GATCGTGATGTAAATAATTTAAATTTGAAGTTACTAGATAAAAAGATAACATTATGTAATA

TATTATCAAATATATACTAAAAATATAATTTTCATCTATCTCAATTGAAAAAATATCCGGG

TTTTGAGTCTATTGTCAATGACGA

>HM068

AAAAAAATAAAGGTTCTATACTCATTAAACAAAGTGCCAAATTCAGAGATACTACTTCCT

TTGGATAATTTGTATCGTCACCAACGATCTTTGAGTTATTTCAAAAATCACATTCTCAATT

TTGTGGTCCAACATTCACAAAATGGTTTGTCGGGCTTTAAGTCTAGTAATTGTGAAACATG

CATATGCATGAGGTAATTAATTGATAATCTGAAAAGGTTAAATAGTTAATCAATTGTACTT

TGGTTATTTTTAATTTGTTCTCTATAATTTTTCAGTCAAAATTGAATTTGCTTTAACCATT

TAGTGTAGTAGTATGATATTACTTGCTCTACATTCTTGGATCTAAAAGTCTAATTAGTATC

GTATAAAAAATATTCTAGTTAATTAAGATACTTTTGTTCATTCTAATTATTCTTGGATTAC

TTGTGAAACTTTTAGGGATGAACAAGAAAGTCCACTAAAGAGGCTTCTTTTTGCTAGGAGT

CACTAGTTAGTTTTTTTTTTTTTTGACAAAAGTTAACCATTTTTTTTTATCGCATGGTAGG

AAAAAAAAAATAGAGATTTCCTTTGGTACTCATAAAAAAAAAAGTTGGATGGATAATGGAT

TAATCATAACTTATTTAACATAAAATGAATTAAGAGAACATTTGATATTTGGGTTTATTTT

GTAACATAAATATTAATATCAATGTTTGAAAGAGTTTTTAACAATAATTTGTAAAAGAAAG

CATAATATATCTATCAAAATAATTGTAAAAGAAAAAATTTCTTTTGGTGGTATGACATGGA

AAAATAATTAATTCTTATTAGATGAAAGTGTAAAATTAATTTGCATTAACAAAGCATATTT

CATTAAACTCCTTTTAGTTTTTGTTGGAAAAAATAAATTTATTGTTACTCAAAAAATAAAA

GTGTACAATTAATATATTTACTCAAAAGTCCCCTAAATGGGATGTAATTATCAAGGGATGT

GAGCGGTTTAACTTTAAGTAATGAATCAACTGTTGCATAATCACTTTGAAAGAAACTTGAG

ATTCTTCAATTAGCAAAATCTTGAATAGAATAATGAGATTATTCCTAATGCTAATGATACC

CACCTCTCCTATAAGTTGATGGGGCAGAACAAATTATGCACTCAGGACAACTTTTCTCAAA

TTTTGAAAACTTACCACACGAAGAACACACTTCCACTTGCACAAGTAAGAATATAGTATAA

CAATAAATTTACCACTCATAGCTCAATGGATATTAAAATATTGCTAATCTTTTATAGACAG

ATGTTGGCAAATTAGTATAAATTAGTTATTATATTAGTTTTTAAGGTTCAATCTACTTTTT

AAAACTCTTATTAAGTTGTTTAGCTTGCCAACCAAAGACTAAATTATCTTTATTTGTTAGG

AAATAAAAGCACTTATTTTATTTTCTCCATATTTGTTTCCTCTTTGTTCCATTACTAAAAA

AATTATAATATATTAGTAACAAAAAAATTTGGGGAGGGAATGTTATTTTTAAAAAAGATGT

TTTATTTATATTTAAGTGTTTTATTGAAATTCCTCGTGAGTTTAGCTCAATTGGTATGGAC

AATACATAATATATGCAAAATTCGAGGTTCAAACCCTGAGCACCAAAAAAAAAAGTGTTTT

ATTGAAAAATATCAAGAACTTGAAAACAAATTTCCTTTTTATGAACTAGATATTGTCACAT

AGACAACTTAAGAATTGACATTTTTTTTAACACTATTGTATTTTCTGAACTCGACTTTAAC

TCGAGATCAATGAGTAAACTAAAAAAACTCGTATTATTTTATCTAAGTGTTCTTAGTGTGA

GAAAACATTATAATTGTTTGCTTAGTCTATTTCTCCCTATATTCTCACCCTCATTCTTGGT

TTGCACATAGAAAGGAGAAAAAAAAAATTAGAGAAGAGTTGAATGGAACAGAAAGAAAGAG

CAAGCAAAATAATCACTAAAAAAAATTCCCCTAAAAGAAAATCACTGAAAAATATTATCCA

CCTCTATTTTAAATAATAATAATAATGAAAACAACAATATTTTTTTTTATAGATTTTCTCT

ACTAGAGGTAATTTATTTTAAACACTAAATGTCCACACCTCTAACAAAAAAAATGCATAAT

ATTATAATAGATTATTGTAAGAAAGAGTGACAAATGTTGAATTAGTTATGGAATGAAGAGT

GAGAGAGATTATACAAATAAATAAATATAAATATAAATATAAATAAAGAATAGAGAAGAAC

ATAGTACTTGAATGAAGAGATTTGGCTTGTACTTCCATTACACTTGTAAGCTTTCTACTTC

CTTCACATTCCTTCTCTTTCTCTATCTTTCCTTTTATGATATGTGTACTTTGTTATTGCTT

CATTTACTACCGACATGACTCCAACCTATTAGGGCTTTATCTGAATAATGTTAGTTATTTT

CTCTCATTTTCTTTGCTTCTTCTTATTCTTGGAACTTATAAAGGGTCTTGTCAATTCTATC

ATTCTCTTTTGTATACTTACAAATATAAATATTCTCTTTAGCTTCATTTTCATGTCTTCTT

CTACTAATAAGCTATGGAAATTGAAGTTTTTGTTTTCATGTTTATTTTTCACTTCAGCTCC

TCTTTTTTGATGTTGATGCTGAAGATTGAATAAAAACTGAGGTGGTATGTATGAGAACAAG

AACATTTTTCATTCATCTCTGTTATTTGAACATCTAAATTTTAAACAACTATCGCGACAAT

TTTTTAAGTGGTTGAAGTATTTTAAATAGGTGGTTAATTACATTAAGTAGATAGTTAATTG

ATATATCAGGTGTCAAAACTCATTAACCATCAACATAACTTCATTAGCCATGAATTTTTGA

CGTGATTAACCAAATATTGTAGATGCTCAATTACATGTAGATTTGATGGAGTTGTCCACAA

TTTCTCATATATCTATTAACTAATAACCATCTACCGAATATAATTAACCATATATTAGAAT

ATCATTAACCTCTAAAACAGTTGTCGCGAAAGTTGGTTGTACAAAAATCATTTCTATTTTT

TACGCCACTAGTTCTCTTGTTATTTCTCTTTGTTGGAAAGTTGTTGTTTTTAACTTTGAGC

TTAGTCTTATTAATTAACTTGTAATGGTAACAACATTTTTCTATCTTTGTTTGAAGGAAGT

AGTTACTGTGTCAACTTTAGTAATTAATTTGGATCATTTTGGAGGCAAAATATGGCTATGC

AACCTGTTTATTTTAAAGAACATGAAGGAAATGTCCACAATTCTGTTGGACAGTTTTCATC

TGTGACTTCAGCACCATGGTGGAGTAATGCCTATGGATCTCAACCTGTTTATGGAGGAGAC

TCTTGTGGCCAAATGAAACCTTTTTCACTAGAGCTTTCCAACTACATAGACCAACTTGCTC

CGAGTAAGAACTTAGTTCGAGGAGTTGAACAATTGTTTGATAAAGGGCATACAAACCAATT

CACCATCTTTCCAGGTACTTGTTATTCAATATAATTCCGGTTTTGAATGAATTGAATTTTG

TTAAGTTGGTTCTGGCTAAACGTGCGTTAAATATAATATGATTTATGTTTGGATACGTTTA

TGTAAAAGTGAGTTGAACATAAATTGGAGACTAAATATCAATTGCAGAGGCAAAAGCTTCA

AATTCTAATTTCAAGTTAGAATCAAATCAATTCTACTCGTAAGCATCTAAATATTTCAAAA

CCAATTCTACATCTCTAGAACTAAATTTGCATTCAAACATTTTCTGCTACAGATGATTGTA

AGATGTCAGTTGATACACCAAATCATCAAGCAACCTTATCCCTGCAATCACCATTTGCTGC

CGAGCCACTTAATCGATTTGAGCTAGGTTTTAACCAGTCTATGGTAAATCTTCTTATTAAA

GCAATCCCTTTGATCATGTTTTAGTATTTTACTTTTGATGTACATAAATTCATTATGTTTT

TATTATCCTTTCCATGCTAGATCTGTGCAAAATATCCGTATATAGATCAATTTTACGGGCT

CTTCTCGACTTATGGACCTCAAATCTCGGTTTGTCTTCACCTCGGCTATTTTATTAATCTT

TGTATATATCATGCAATGTCTATAAGTACTTGAATGTGAATTTGTTTTATTTACAGTGTGT

ATGCTTACATGAGAAAGCCAGAAATCTGTTTACAACTATCTTGTTAGTATTCTTCTAAATC

TTTCAAATGTAGAGTTGATTATGAAATTTGTTGTTAATGAGTTTTCGGCTTTATGAATGAA

TTGTAAGACAAGTAATGAAACAAAAACTAGAAGAAAATTGAATTATATGATGCAATCTGTG

TTGAAGTTTTTGCATGTATTCAAGTTCTGCTTTTAGTTACTATTTGTTTATCTGCTGCAAT

ATTTCATCAATTATTATTCATGTATGTACCAATATTTAATAAATTAGGGGCGAATTATGCT

TCCGCTTAGCATGACATCTGACGACGGACCAACATACGTGAATGCTAAGCAATACCATGGA

ATCATCAGACGCAGGCATTCTCGTGCCAAAGCTGTGCTTCAGAATAAATTGATAAAGCGTA

ACAAGGTATGAAACTGAGTATTTTTCCTCACAACAATGTTCGAAAACTTGTGAAATAGTAT

ATTTTTCCTTTTACCCTTTTTATACTTATATTAAGGTTTTGTATTTGTCTTGCAGCCATAT

ATGCACGAATCGCGTCATCTACATGCAATGCGTAGACCAAGAGGATGCGGTGGTCGTTTCT

TGAACACAAAAGTTTCTGCTAATGGAAACGGTAAAAGCGGGAGTGAAGAGAACGGAAACAT

TGGTGGCCTACAGCTGCAGTCCAGTGGTTCTCAGAGTTCCGAAGTCTTACAATCTGAGGTT

GGAACTTTAAATTCGTCGAAGGAGACAAACGGAGGCAGTCCAAATGTCTCGGGGTCAGAGG

TGACTAGCATGTATACACAGGGAGGTCTTGATAGCTTTACTGTCAATCATATCGGATCTAC

TGTCCACTCTTTGGGAGACATGATCGATACTGGACACGGTATCGTCATGCCAACAAAATGG

TTTGCAGCAGCTGGCAGACAGCTGCTGGAACCATAAGTTTCGATTCAGAAAGGAAACAAGT

GGGTTTGGTACAATGTGAAATATTTTGCACCAAACTCATCCTTTCCGAGACCAGATGAAGA

AGCTATGTTTCAGTTTGTTGTGTTTACTACGACAAATTTAGTTTCGGAAGACTACTTTTCC

ATCTGGTGCTCAGGCAACTCATTCTTGGCTTATTCTCAGGAAACTCATCCTTGGCTCGTAA

TATTTAGTAGTATTGTCATTGTCTTTCCGCGCAGGCTTGCCGTGGCATGGTAGGCATGCTA

ATGACTTTGGTATTTTCATGCAGTTATAACTATGATGTGTCTTTGTTTGTTGTTAAAATAA

AAAACATGAACTCTAGCTAGGTGCATGTGTGTGTTTTTAATCTTGTCTACTAAGTTTGGTG

TTTTGTAATGGATTTCTGACTTTATGGAGCAATGTATTGTAACTCTACTAAGAAGTGTAAC

ATTTTATTTCTCCCTCTCTAAGGATTGTATAAGAACCTCTTATTTTCAGACTCTACTTAAT

CCTATTTTCTATGTCTGTATGATTTTTATATTTCTAGGACAATCAAATTGGCTTGTAGAAG

CTCAAAAGCATGCTCACAAAGTAGGTACTTATGTAGGGAACTTCGTACTCTAATAATAACT

GGTTATTAACGTTATAATTAAATGCAAAATTTGATAAGTAGTAGGGTTTGGTAAGTAATAT

AACAACACCATGTTGGCTTGGGATTCTGAATTGTGTTATTGGAGTACAACTCTTAAATAAT

GATCGTGATGTAAATAATTTAAATTTGAAGTTACTAGATAAAAAGATAACATTATGTAATA

TATTATCAAATATATACTAAAAATATAATTTTCATCTATCTCAATTGAAAAAATATCCGGG

TTTTGAGTCTATTGTCAATGACGA

>HM069

AAAAAAATAAAGGTTCTATACTCATTAAACAAAGTGCCAAATTCAGAGATACTACTTCCT

TTGGATAATTTGTATCGTCACCAACGATCTTTGAGTTATTTCAAAAATCACATTCTCAATT

TTGTGGTCCAACATTCACAAAATGGTTTGTCGGGCTTTAAGTCTAGTAATTGTGAAACATG

CATATGCATGAGGTAATTAATTGATAATCTGAAAAGGTTAAATAGTTAATCAATTGTACTT

TGGTTATTTTTAATTTGTTCTCTATAATTTTTCAGTCAAAATTGAATTTGCTTTAACCATT

TAGTGTAGTAGTATGATATTACTTGCTCTACATTCTTGGATCTAAAAGTCTAATTAGTATC

GTATAAAAAATATTCTAGTTAATTAAGATACTTTTGTTCATTCTAATTATTCTTGGATTAC

TTGTGAAACTTTTAGGGATGAACAAGAAAGTCCACTAAAGAGGCTTCTTTTTGCTAGGAGT

CACTAGTTAGTTTTTTTTTTTTTTGACAAAAGTTAACCATTTTTTTTTATCGCATGGTAGG

AAAAAAAAAATAGAGATTTCCTTTGGTACTCATAAAAAAAAAAGTTGGATGGATAATGGAT

TAATCATAACTTATTTAACATAAAATGAATTAAGAGAACATTTGATATTTGGGTTTATTTT

GTAACATAAATATTAATATCAATGTTTGAAAGAGTTTTTAACAATAATTTGTAAAAGAAAG

CATAATATATCTATCAAAATAATTGTAAAAGAAAAAATTTCTTTTGGTGGTATGACATGGA

AAAATAATTAATTCTTATTAGATGAAAGTGTAAAATTAATTTGCATTAACAAAGCATATTT

CATTAAACTCCTTTTAGTTTTTGTTGGAAAAAATAAATTTATTGTTACTCAAAAAATAAAA

GTGTACAATTAATATATTTACTCAAAAGTCCCCTAAATGGGATGTAATTATCAAGGGATGT

GAGCGGTTTAACTTTAAGTAATGAATCAACTGTTGCATAATCACTTTGAAAGAAACTTGAG

ATTCTTCAATTAGCAAAATCTTGAATAGAATAATGAGATTATTCCTAATGCTAATGATACC

CACCTCTCCTATAAGTTGATGGGGCAGAACAAATTATGCACTCAGGACAACTTTTCTCAAA

TTTTGAAAACTTACCACACGAAGAACACACTTCCACTTGCACAAGTAAGAATATAGTATAA

CAATAAATTTACCACTCATAGCTCAATGGATATTAAAATATTGCTAATCTTTTATAGACAG

ATGTTGGCAAATTAGTATAAATTAGTTATTATATTAGTTTTTAAGGTTCAATCTACTTTTT

AAAACTCTTATTAAGTTGTTTAGCTTGCCAACCAAAGACTAAATTATCTTTATTTGTTAGG

AAATAAAAGCACTTATTTTATTTTCTCCATATTTGTTTCCTCTTTGTTCCATTACTAAAAA

AATTATAATATATTAGTAACAAAAAAATTTGGGGAGGGAATGTTATTTTTAAAAAAGATGT

TTTATTTATATTTAAGTGTTTTATTGAAATTCCTCGTGAGTTTAGCTCAATTGGTATGGAC

AATACATAATATATGCAAAATTCGAGGTTCAAACCCTGAGCACCAAAAAAAAAAGTGTTTT

ATTGAAAAATATCAAGAACTTGAAAACAAATTTCCTTTTTATGAACTAGATATTGTCACAT

AGACAACTTAAGAATTGACATTTTTTTTAACACTATTGTATTTTCTGAACTCGACTTTAAC

TCGAGATCAATGAGTAAACTAAAAAAACTCGTATTATTTTATCTAAGTGTTCTTAGTGTGA

GAAAACATTATAATTGTTTGCTTAGTCTATTTCTCCCTATATTCTCACCCTCATTCTTGGT

TTGCACATAGAAAGGAGAAAAAAAAAATTAGAGAAGAGTTGAATGGAACAGAAAGAAAGAG

CAAGCAAAATAATCACTAAAAAAAATTCCCCTAAAAGAAAATCACTGAAAAATATTATCCA

CCTCTATTTTAAATAATAATAATAATGAAAACAACAATATTTTTTTTTATAGATTTTCTCT

ACTAGAGGTAATTTATTTTAAACACTAAATGTCCACACCTCTAACAAAAAAAATGCATAAT

ATTATAATAGATTATTGTAAGAAAGAGTGACAAATGTTGAATTAGTTATGGAATGAAGAGT

GAGAGAGATTATACAAATAAATAAATATAAATATAAATATAAATAAAGAATAGAGAAGAAC

ATAGTACTTGAATGAAGAGATTTGGCTTGTACTTCCATTACACTTGTAAGCTTTCTACTTC

CTTCACATTCCTTCTCTTTCTCTATCTTTCCTTTTATGATATGTGTACTTTGTTATTGCTT

CATTTACTACCGACATGACTCCAACCTATTAGGGCTTTATCTGAATAATGTTAGTTATTTT

CTCTCATTTTCTTTGCTTCTTCTTATTCTTGGAACTTATAAAGGGTCTTGTCAATTCTATC

ATTCTCTTTTGTATACTTACAAATATAAATATTCTCTTTAGCTTCATTTTCATGTCTTCTT

CTACTAATAAGCTATGGAAATTGAAGTTTTTGTTTTCATGTTTATTTTTCACTTCAGCTCC
[truncated: 1,009,024 more chars]
