# Supplementary material for: MtNF-YA1, A Central Transcriptional Regulator of Symbiotic Nodule Development, Is Also a Determinant of Medicago truncatula Susceptibility toward a Root Pathogen
Source: Front Plant Sci. 2016 Dec 5;7:1837. doi: 10.3389/fpls.2016.01837 (PMC5137509; doi:10.3389/fpls.2016.01837)
Supplement: Supplementary file 7 [file Image_1.pdf]

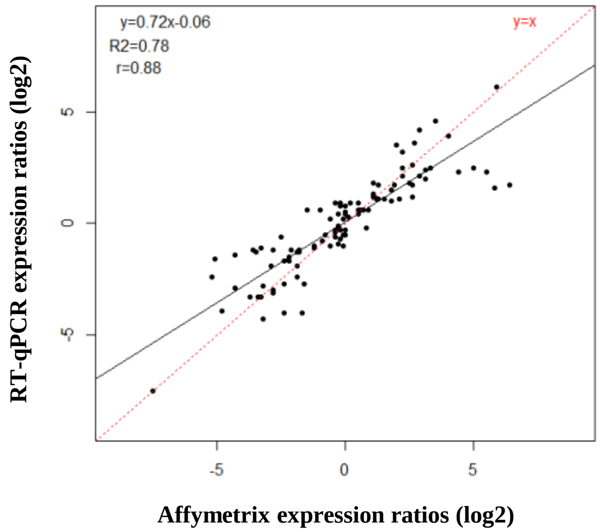

Supplementary Figure 1: Linear regression between gene expression ratios obtained for regulated genes identified wih Affymetrix microarray data (according to the five ratios used for clustering) and those obtained by qPCR with the $2^{-\Delta\Delta Ct}$ method.

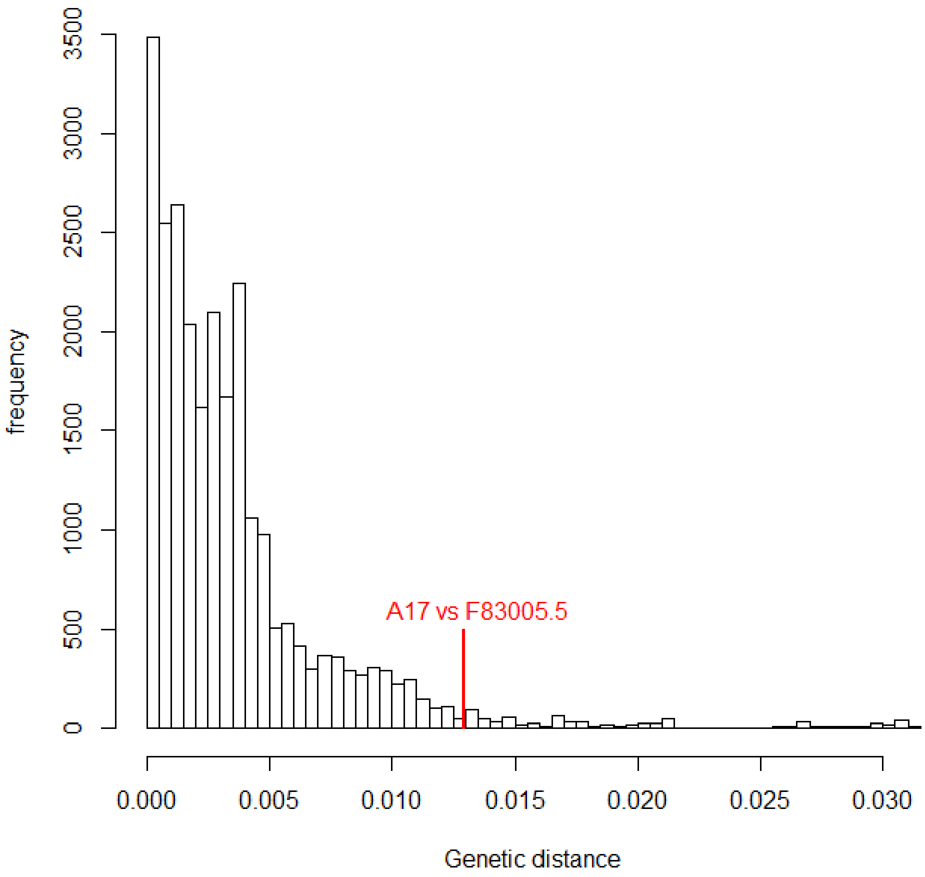

Supplementary Figure 2: Distribution of 25651 genetic distance values across 226 accessions of *M. truncatula*. Estimates were obtained using the Kimura 2-parameters evolutionary model. In red is indicated the genetic distance between A17 and F83005.5 (0.013), which is among the top 3% highest pairwise distance values calculated.

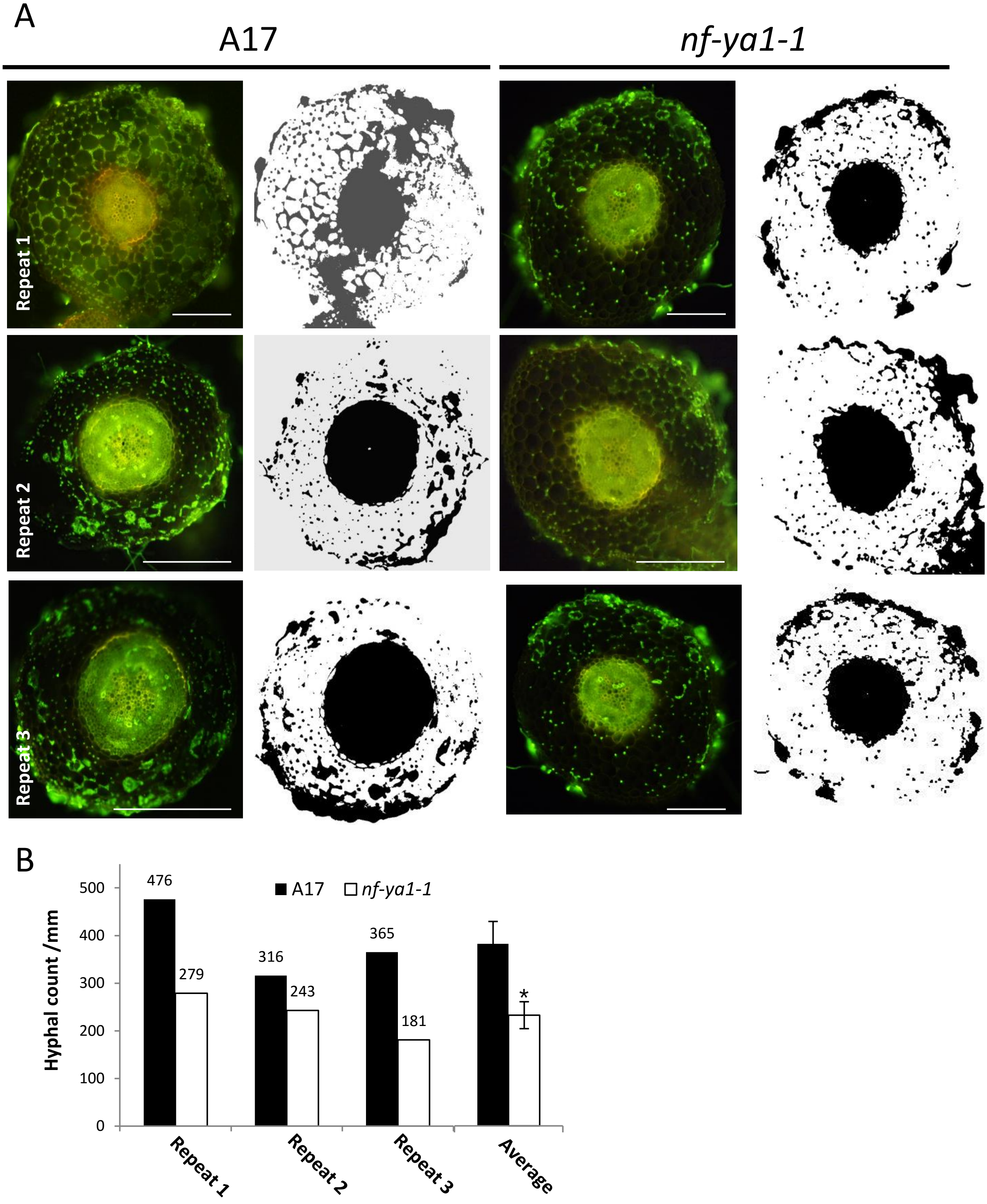

Supplementary Figure 3: Observation of *A. euteiches* hyphal density in root cross sections, 21 days post inoculation (Scale bar =100µm).

A – Observation of root cross sections in three inoculations repeats of the A17 and *nf-ya1-1*. Each picture (left pannel of each genotype) was subjected to a particle analysis (right pannel) to detect the number of hyphae in the root cross section

B– Summary of hyphal count detected in each picture a Mann–Whitney U test was applied to assess significance of *nf-ya1-1* lower colonisation(*: pvalue < 0.05).
